# Evolution in an oncogenic bacterial species with extreme genome plasticity: *Helicobacter pylori *East Asian genomes

**DOI:** 10.1186/1471-2180-11-104

**Published:** 2011-05-16

**Authors:** Mikihiko Kawai, Yoshikazu Furuta, Koji Yahara, Takeshi Tsuru, Kenshiro Oshima, Naofumi Handa, Noriko Takahashi, Masaru Yoshida, Takeshi Azuma, Masahira Hattori, Ikuo Uchiyama, Ichizo Kobayashi

**Affiliations:** 1 Department of Medical Genome Sciences, Graduate School of Frontier Sciences, University of Tokyo, Minato-ku, Tokyo, 108-8639, Japan; 2 Institute of Medical Science, University of Tokyo, Minato-ku, Tokyo, 108-8639, Japan; 3 National Institute for Basic Biology, National Institutes of Natural Sciences, Okazaki, Aichi, 444-8585, Japan; 4 Graduate School of Medicine, Kurume University, Kurume, Fukuoka, 830-0011, Japan; 5 Fujitsu Kyushu Systems LTD, Fukuoka, Fukuoka 814-8589, Japan; 6 Department of Biophysics and Biochemistry, Graduate School of Science, University of Tokyo, Minato-ku, Tokyo, 108-8639, Japan; 7 Department of Computational Biology, Graduate School of Frontier Sciences, University of Tokyo, Kashiwa, Chiba, 277-8561, Japan; 8 Department of Gastroenterology, Graduate School of Medicine, Kobe University, Chuou-ku, Kobe, Hyogo, 650-0017, Japan

## Abstract

**Background:**

The genome of *Helicobacter pylori*, an oncogenic bacterium in the human stomach, rapidly evolves and shows wide geographical divergence. The high incidence of stomach cancer in East Asia might be related to bacterial genotype. We used newly developed comparative methods to follow the evolution of East Asian *H. pylori *genomes using 20 complete genome sequences from Japanese, Korean, Amerind, European, and West African strains.

**Results:**

A phylogenetic tree of concatenated well-defined core genes supported divergence of the East Asian lineage (hspEAsia; Japanese and Korean) from the European lineage ancestor, and then from the Amerind lineage ancestor. Phylogenetic profiling revealed a large difference in the repertoire of outer membrane proteins (including *oipA*, *hopMN*, *babABC*, *sabAB *and *vacA-2*) through gene loss, gain, and mutation. All known functions associated with molybdenum, a rare element essential to nearly all organisms that catalyzes two-electron-transfer oxidation-reduction reactions, appeared to be inactivated. Two pathways linking acetyl~CoA and acetate appeared intact in some Japanese strains. Phylogenetic analysis revealed greater divergence between the East Asian (hspEAsia) and the European (hpEurope) genomes in proteins in host interaction, specifically virulence factors (*tipα*), outer membrane proteins, and lipopolysaccharide synthesis (human Lewis antigen mimicry) enzymes. Divergence was also seen in proteins in electron transfer and translation fidelity (*miaA, tilS*), a DNA recombinase/exonuclease that recognizes genome identity (*addA*), and DNA/RNA hybrid nucleases (*rnhAB*). Positively selected amino acid changes between hspEAsia and hpEurope were mapped to products of *cagA*, *vacA*, *homC *(outer membrane protein), *sotB *(sugar transport), and a translation fidelity factor (*miaA*). Large divergence was seen in genes related to antibiotics: *frxA *(metronidazole resistance), *def *(peptide deformylase, drug target), and *ftsA *(actin-like, drug target).

**Conclusions:**

These results demonstrate dramatic genome evolution within a species, especially in likely host interaction genes. The East Asian strains appear to differ greatly from the European strains in electron transfer and redox reactions. These findings also suggest a model of adaptive evolution through proteome diversification and selection through modulation of translational fidelity. The results define *H. pylori *East Asian lineages and provide essential information for understanding their pathogenesis and designing drugs and therapies that target them.

## Background

Genome sequence comparison within a species can reveal genome evolution processes in detail and provide insights for basic and applied research. For bacteria, this approach has been quite powerful in revealing horizontal gene transfer, gene decay, and genome rearrangements underlying adaptation, such as evolution of virulence [1]. Comparison of many complete genome sequences is feasible through innovations in DNA sequencing.

*Helicobacter pylori *was the first species for which two complete genome sequences were available [2]. This species of ε-proteobacteria causes gastritis, gastric (stomach) ulcer, and duodenal ulcer, and is associated with gastric cancer and mucosa-associated lymphoid tissue (MALT) lymphoma [3,4]. Animal models show a causal link between *H. pylori *and gastric cancer [5,6]. Recent clinical work in Japan suggests that *H. pylori *eradication reduces the risk of new gastric carcinomas in patients with a history of the disease [7].

*H. pylori *shows a high mutation rate and an even higher rate of homologous recombination [8]. Phylogenetic analysis based on several genes revealed geographical differentiation since *H. pylori *left Africa together with *Homo sapiens *[9]. The analysis indicated that the East Asian type (hpEastAsia) is classified into at least three subtypes: East Asian (hspEAsia), Pacific (hspMaori) and native American (hspAmerind) [9,10]. The East Asia subtype (hspEAsia) may be related to the high incidence of gastric cancer in East Asia [4].

*H. pylori *CagA is considered to be a major virulence factor associated with gastric cancer. CagA is delivered into gastric epithelial cells and undergoes phosphorylation by host kinases. Membrane-localized CagA mimics mammalian scaffold proteins, perturbs signaling pathways and promotes transformation. CagA is noted for structural diversity in its C-terminal region, which interacts with host cell proteins. It is classified into Western and East Asian types, with higher activities associated with the latter [11]. The East Asian CagA-positive *H. pylori *infection is more closely associated with gastric cancer [12]. Geographical differences have also been noted for other genes [13-17].

To fully characterize these bacteria (hspEAsia subtype of *H. pylori*) and to study underlying intraspecific (within-species) evolutionary processes in detail at the genome sequence level, we determined the genome sequence of four Japanese strains and compared them to available complete *H. pylori *genome sequences. The sequences of the Japanese strains and two Korean strains were different in gene content from the European and West African genomes and from the Amerind genome. Unexpectedly, divergence was seen in genes related to electron transfer and translation fidelity, as well as virulence and host interaction.

## Results

The complete genome sequences of four *H. pylori *strains (F57, F32, F30 and F16) isolated from different individuals in Fukui, Japan were determined. We compared 20 complete genomes of *H. pylori *(the 4 new genomes and 16 genomes in the public domain; Table 1), focusing on their gene contents.

**Table 1:** Comparison of hspEAsia to other genomes

Strain	Disease	Population	Length	% GC	CDS	Core	*cagA*^(c)^	*vacA*^(d)^	*homAB*	Reference
		subpopulation	(bp)^(a,b)^	content		genes				
F57	Gastric cancer	hpEastAsia hspEAsia	1609006	38.7	1521	1402	ABD	s1a-m1-i1	-/B	This work

F32	Gastric cancer	hpEastAsia hspEAsia	1578824, 2637	38.9	1492	1385	ABD	s1a-m1-i1	-/E^(e)^	This work

F30	Duodenal ulcer	hpEastAsia hspEAsia	1570564, 9129	38.8	1485	1385	ABD	s1a-m1-i1	-/B	This work

F16	Gastritis	hpEastAsia hspEAsia	1575399	38.9	1500	1402	ABD	s1a-m1-i1	-/B	This work

51	Duodenal ulcer	hpEastAsia hspEAsia	1589954	38.8	1509	1424	ABD	s1a-m1-i1	-/B	

52	?	hpEastAsia hspEAsia	1568826	38.9	1496	1383	(A/B)(D/B)D	(s1a)-m1-i1 ^(f)^	-/B	

Shi470	Gastritis	hpEastAsia hspAmerind	1608548	38.9	1517	1401	AB(D/C),CC^(g)^	s1b-m1-i1	-/B	[21]

v225d	Gastritis	hpEastAsia hspAmerind	1588278, 7326	39.0	1506	1377	AB(C/D)(C/D), (tr) ^(g,h)^	s1a-m1-i1	-/B	[22]

Cuz20	?	hpEastAsia hspAmerind	1635449	38.9	1527	1364	AB(D/C)×5(tr) ^(h)^	s1a-m2-i2	-/A	

Sat464	?	hpEastAsia hspAmerind	1629557, 8712	38.9	1465	1376	AB(D/C)	s1b-m1-i1	-/B	

PeCan4	Gastric cancer	hpEastAsia hspAmerind?	1560342, 7228	39.1	1525	1388	A(B/A)BC	s1a-m1-i1	-/B	

26695	Gastritis	hpEurope	1667867	38.9	1575	1411	ABC	s1a-m1-i1	A/-	[28]

HPAG1	Gastritis	hpEurope	1596366, 9370	39.1	1492	1394	A(B/A)C	s1b-m1-i1	B/-	[30]

G27	?	hpEurope	1652982, 10031	38.9	1560	1400	ABCC	s1b-m1-i1	B/-	[56]

P12	Duodenal ulcer	hpEurope	1673813, 10225	38.8	1593	1396	ABCC	s1a-m1-i1	A/-	[49]

B38	MALT lymphoma	hpEurope	1576758	39.2	1493	1388	-	s2-m1-i2	A/-	[51]

B8^(i)^	Gastric ulcer^(i)^	hpEurope	1673997, 6032	38.8	1578	1385	ABC	s1a-m2-i2 ^(j)^	A/A	[57]

SJM180	Gastritis	hpEurope?	1658051	38.9	1515	1381	ABC	s1b-m1-i1	B/B	

J99	Duodenal ulcer	hpAfrica1 hspWAfrica	1643831	39.2	1502	1383	(A/B)C	s1b-m1-i1	A/B	[2]

908^(k)^	Duodenal ulcer	hpAfrica1 hspWAfrica	1549666	39.3	1503	1393	ABC	-s1b-(-)-i1 ^(j,k,l)^	-/-^(k)^	[139]

a) The first number is the length of the chromosome and the second number (when present) is that of the plasmid.

b) Accession numbers are as follows: F57 [DDBJ:AP011945.1 http://getentry.ddbj.nig.ac.jp/cgi-bin/get_entry2.pl?database=ver_ddbj&query=AP011945.1], F32 [DDBJ:AP011943.1 http://getentry.ddbj.nig.ac.jp/cgi-bin/get_entry2.pl?database=ver_ddbj&query=AP011943.1, DDBJ:AP011944.1 http://getentry.ddbj.nig.ac.jp/cgi-bin/get_entry2.pl?database=ver_ddbj&query=AP011944.1], F30 [DDBJ:AP011941.1 http://getentry.ddbj.nig.ac.jp/cgi-bin/get_entry2.pl?database=ver_ddbj&query=AP011941.1, DDBJ: AP011942.1 http://getentry.ddbj.nig.ac.jp/cgi-bin/get_entry2.pl?database=ver_ddbj&query=AP011942.1], F16 [DDBJ:AP011940.1 http://getentry.ddbj.nig.ac.jp/cgi-bin/get_entry2.pl?database=ver_ddbj&query=AP011940.1], 51 [GenBank:CP000012.1], 52 [GenBank:CP001680.1], Shi470 [GenBank:NC_010698.2], v225d [GenBank:CP001582.1, GenBank:CP001583.1], Cuz20 [GenBank:CP002076.1], Sat464 [GenBank:CP002071.1, GenBank:CP002072.1], PeCan4 [GenBank:NC_014555.1, GenBank:NC_014556.1], 26695 [GenBank:NC_000915.1], HPAG1 [GenBank:NC_008086.1, GenBank:NC_008087.1], G27 [GenBank:NC_011333.1, GenBank:NC_011334.1], P12 [GenBank:NC_011498.1, GenBank:NC_011499.1], B38 [GenBank:NC_012973.1], B8 [GenBank:NC_014256.1, GenBank:NC_014257.1], SJM180 [GenBank:NC_014560.1], J99 [GenBank:NC_000921.1], 908 [GenBank:CP002184.1]. Draft sequence of the East Asian strain 98-10 [140]. 98-10, [GenBank:NZ_ABSX01000001.1] - [GenBank:NZ_ABSX01000051.1].

c) Letters in parentheses are the hybrid EPIYA segment. For example, (A/B) is a hybrid of EPIYA-A and EPIYA-B segments [21,22,141].

d) Reference [142,143].

e) Designated as *homE *as it was very different from *homA *or *homB*.

f) "s" region locates outside of the ORF.

g) A second *cagA *gene between *cagM *and *cagP*.

h) (tr), truncation.

i) Mongolian gerbil-adapted, originally from gastric ulcer.

j) *vacA *gene is split.

k) According to a reference [139], the sequence might not represent a complete genome, although it is deposited as a complete circular genome in GenBank.

l) "m" region was not available because of a deletion in the center of the ORF.

### Japanese/Korean core genomes diverged from the European and then the Amerind

A phylogenetic tree was constructed from concatenated seven genes *atpA, efp, mutY, ppa, trpC, ureI *and *yphC*, which were used for multi-locus sequence typing (MLST) [18] and phylogenetic analyses [19,20]) (Additional file 1 (= Figure S1)). The tree showed that the 6 East Asian strains, the 4 Japanese strains (F57, F32, F30 and F16) and the 2 Korean strains (strain 51 and strain 52), are close to the known subpopulation hspEAsia of hpEastAsia, whereas 4 strains (Shi470 [21], v225d [22], Sat464 and Cuz20) are close to another subpopulation of hpEastAsia, hspAmerind. Strains 26695, HPAG1, G27, P12, B38, B8 and SJM180 were assigned to hpEurope. Strains J99 and 908 were assigned to hspWAfrica of hpAfrica1. PeCan4 was tentatively assigned to hspAmerind although it appears to be separate from the above 4 hspAmerind strains and somewhat closer to other subgroups (a subgroup of hpEurope, hspMaori and a group of "unclassified Asia" in the HpyMLST database [18]).

We deduced the common core genome structure of these 20 genomes based on the conservation of gene order using CoreAligner [23] (Table 1). CoreAligner determines the set of core genes among the related genomes not by universal conservation of genes but by conservation of neighborhood relationships between orthologous gene pairs allowing some exceptions. As a result, CoreAligner identified different numbers of core genes among strains (1364-1424), which reflect deletion, duplication and split of the core genes in the individual strains.

**Figure 1:**
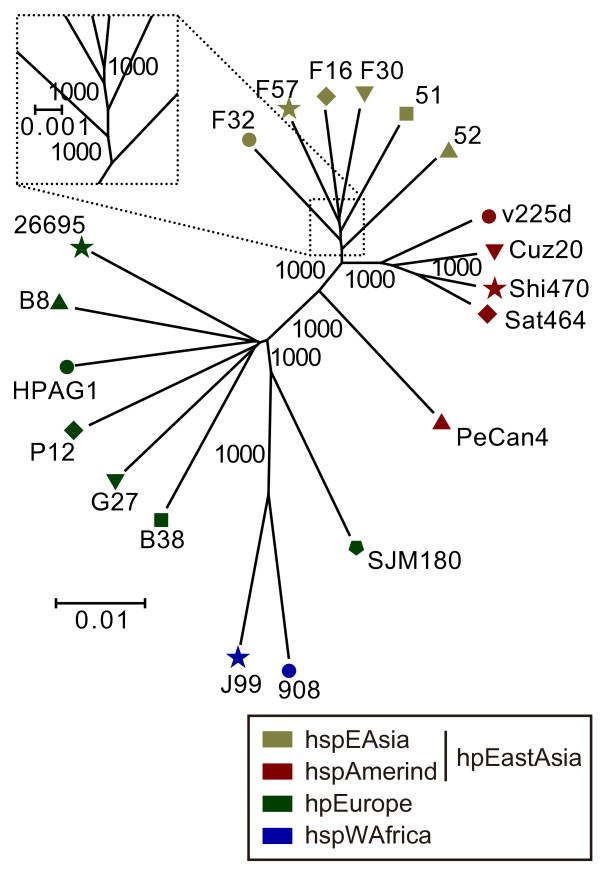
**Phylogenetic tree of 20 *H. pylori *strains based on their well-defined core genes**. Well-defined core OGs were used for neighbor-joining method (see Methods). Numbers indicate bootstrap values. Scale bar indicates substitutions per nucleic acid residue (change/nucleotide site). The assignment of population/subpopulation was based on a phylogenetic tree constructed from the concatenated alignment of fragments of seven genes used in the *H. pylori *MLST database (*atpA, efp, mutY, ppa, trpC, ureI *and *yphC*) [18]. Classification of population/subpopulation was as described [10,19].

For phylogenetic analysis among the strains, we further extracted 1079 *well-defined core orthologous groups *(OGs) as those that were universally conserved, non-domain-separated, and with one-to-one correspondence (see Methods). The concatenated sequence of all well-defined core OGs resulted in a well-resolved phylogenetic tree (Figure 1). The tree was composed of two clusters, one containing the Japanese, Korean and Amerind strains and the other containing the European and West African strains. The tree strongly supported a model in which the Japanese/Korean strains (hspEAsia) and the Amerind strains (hspAmerind) diverged from their common ancestor, which in turn diverged from the ancestor shared by the European strains (hpEurope) long before. This conclusion is robust, as shown by the high bootstrap values of the internal nodes, primarily because the tree is composed of a large quantity of sequence information with approximately 1400 genes. The Japanese and Korean strains were not separated into two clusters. PeCan4 appeared diverged from the other four hspAmerind strains as expected from the result of the phylogenetic analysis based on the 7 genes described above. SJM180 appeared diverged from the other hpEurope strains in the well-defined core gene-based tree.

### Phylogenetic profiling to identify gene contents of hspEAsia

To thoroughly characterize the gene contents specific to the Japanese/Korean (hspEAsia) strains, we conducted phylogenetic profile analysis using the DomClust program [24]. This analysis determines the presence or absence of a domain, rather than a gene, and allows detection of split genes, partially deleted genes and partially duplicated genes (detailed in Methods). Their features will be explained in the next five sections.

### Differences in outer membrane proteins and related proteins in the number of loci of gene families and in alleles at each locus

One of the emerging features of the East Asian (hspEAsia) strains is the change in the number of loci of some of the outer membrane protein (OMP) families. We detected five OMP genes (gene families; *oipA*, *hopMN*, *sabAB*, *babABC *and *vacA-2*) with the number of loci different between the hspEAsia and hpEurope strains (Table 2). In all but one gene family, the difference in the number of locus was the result of gene decay in the East Asian (hspEAsia) strains.

**Table 2:** Characteristic gene contents of East Asian (hspEAsia) *H. pylori*

Population type	Strain	Locus of outer membrane proteins					Periplasmic endonuclease *nucG*^(d)^	Molybdenum- related function
				
		*oipA/oipA-2*	*hopM/hopN*	*babA/babB/babC*^(a)^	*sabA/sabB*^(b)^	*vacA-2*^(c)^		
hspEAsia	F57	A/A^(e)^	+/-	A/B/-	A/-	x	x	-
	
	F32	A/x	+/-	A/B(tr)/-	A/-	x	x	-
	
	F30	A/A	+/-	A/B/-	A/-	x	x	-
	
	F16	A/A	+/-	A/B/-	A/-	x	x	-
	
	51	A/A	+/-	A/B/-	A/-	+	x	-
	
	52	A/A	+/-	A/B/-	A/A	x	x	-

hspAmerind	Shi470	A/x	+/-	A/B/-	A/-	+	+	+
	
	v225d	A/x	+/-	A/B/-	A/-	+	+	+
	
	Cuz20	A/x	+/-	A/B/-	A/-	+	+	+
	
	Sat464	A/x	+/-	A/B/-	A/-	+	+	+
	
	PeCan4	A/A	+/-	A/B/-	A/B	+	+	+
	
hpEurope	26695	A/-	+/+	B/A/C	A/A	+	x	+
	
	HPAG1	A/-	x/+	A/C/B	A/B	+	+	+
	
	G27	A/-	+/x	C/B/A	A/B	+	+	+
	
	P12	A/-	+/+	A/B/B(tr)	B/B	+	+	+?^(f)^
	
	B38	A/-	+/+	A/A(tr)/-	A/-	+	+	+
	
	B8	A/-	+/+	A/A/-	A/Q^(g)^	+	+	+
	
	SJM180	A/-	+/+	B/C/A	A/B	+	+	+

hspWAfrica	J99	A/-	+/+	A/B(tr)/-	A/B	+	+	+
	
	908^(h)^	A/-	-/-^(h)^	A(tr)/B(tr)/-^(h)^	-/-^(h)^	+	+	+

+, present; x, disrupted (nucleotide sequence partly remained); -, absent. See Additional file 2 (= Table S1) for a detailed list.

a) *babA *locus corresponds to HP0896; *babB *locus, HP1243; *babC *locus, HP0317.

b) *sabA *locus corresponds to jhp0662; *sabB *locus, jhp0659.

c) Paralog of *vacA *(HP0289), but not *vacA *itself (HP0887). Another paralog *vacA-4 *(HP0922) is in Table 6.

d) HP1382.

e)/, different loci.

f) One of 12 molybdenum-related genes was truncated.

g) *hopQ *gene. Two *hopQ *copies exist, one at *sabB *locus and the other, as in other strains, at the *hopQ *locus.

h) From the description of the reference [139], the sequence might not represent a complete genome, although it is deposited as a complete circular genome in GenBank. Hence, care should be taken in interpreting the results.

Relevant information about each family from draft sequence of the Japanese strain 98-10 (NZ_ABSX01000001.1- NZ_ABSX01000051.1) [143] are as follows: *oipA/oipA-2*, with at least one copy, although the exact copy number cannot be determined because of a short contig encoded only the *oipA *gene but not the flanking region; *hopM *locus, +? (partial sequence at an end of the contig); *hopN *locus, not applicable because it was at an end of contigs (*hopN *fragment is deposited but the sequence was partial at both ends of the contig, preventing locus assignment); *babA/babB/babC*, A?/?/? (*babA *at *babA *locus but partial at an end of the contig; *babB *and *babC *loci, not applicable because they were at ends of contigs; *babB *sequence was partial at both ends of the contig, preventing locus assignment); *sabA/sabB*, +/-; *vacA-2*, x; *nucG *split as in the other hspEAsia strains; Molybdenum-related function, x.

The notable exception was *oipA*, for which a secondary locus was found in hspEAsia (6/6 strains) and hspAmerind (5/5), but not in hpEurope (0/7) or hspWAfrica (0/2). This increase of the secondary locus can be explained by a novel DNA duplication mechanism associated with inversion [25]. The two *hopMN *loci in hpEurope (7/7 strains) and hspWAfrica (1/2) were reduced to one locus in the hspEAsia (6/6) and hspAmerind (5/5). This loss was likely caused by the same duplication mechanism [25].

For the *babABC *family, the *babC *locus [26] was empty in all the hpEastAsia strains (6/6 hspEAsia and 5/5 hspAmerind) as well as from all the hspWAfrica strains (2/2) and two hpEurope strains (B38 and B8). This is in contrast to the presence of three loci in the other (5/7) European strains (Table 2).

The strain J99 carried a *sabA *gene (jhp0662) at the *sabA *locus and a *sabB *gene (jhp0659) at the *sabB *locus [27]. All the hpEurope strains but the strain B38 (6/7) and this hspWAfrica strain (J99) had these two loci, whereas all the hpEastAsia strains but the strains 52 and PeCan4 (5/6 hspEAsia and 4/5 hspAmerind) lacked *sabB *locus (Table 2). These hpEastAsia strains all carried a *sabA *gene at the *sabA *locus. Genes of hpEurope differed among strains. Three strains (HPAG1, G27 and SJM180) carried a *sabA *gene at the *sabA *locus and a *sabB *gene at the *sabB *locus, as J99. The strain 26695 carried a *sabA *gene at both the *sabA *and *sabB *loci, whereas the strain P12 carried a *sabB *gene at both the loci. The strain B8 carried a *sabA *gene at the *sabA *locus and a *hopQ *gene at the *sabB *locus, along with another *hopQ *gene at the *hopQ *locus.

Some of these genes (*oipA*, *babA *and *babB*) and *homAB *genes were previously reported to diverge between the East Asian and Western strains [13,14,17]. Difference in the number of copies of *homAB *genes between East Asian and Western strains was reported [17].

**Figure 2:**
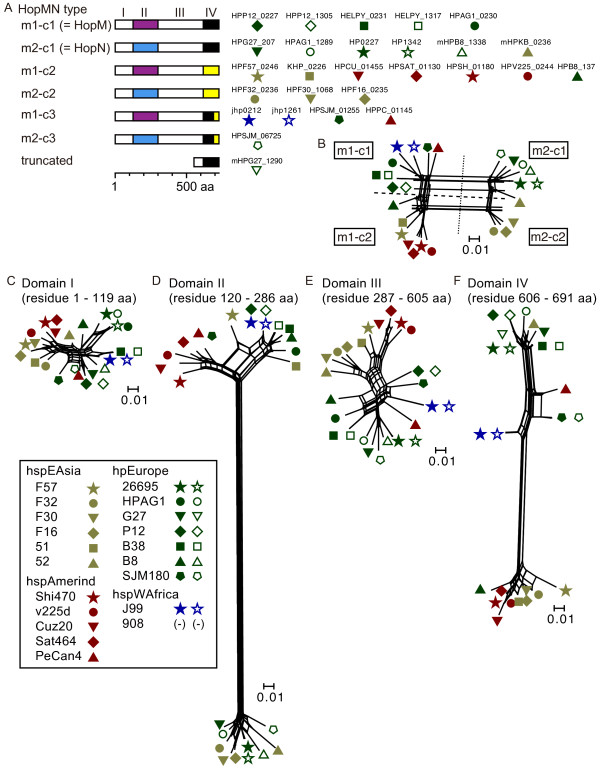
**East Asia-specific sequence at the C-terminus of the putative product of *hopMN***. (A) Four types of *hopMN *genes. Type c3 of m1-c3 and m2-c3 is composed of parts of c1 and c2. The c1-m1 and c2-m1 types correspond to *hopM *and *hopN*, respectively. (B) Phylogenetic network of whole region of proteins. Types m1-c3 and m2-c3 cannot be clearly distinguished from m1-c1 and m2-c1 in this figure. (C)-(F) Phylogenetic networks for the four domains. Scale bar indicates substitutions per amino acid residue (change/amino-acid site). Positions are for HP0227 of strain 26695.

For *hopMN*, two gene types (*hopM *and *hopN*) have been recognized [26,27]. Phylogenetic network analysis revealed two variable regions within the *hopMN *family (region II and IV; Figure 2). Combining the two types of two variable regions defined four main gene types, of which two corresponded to *hopM *and *hopN*. The two types in region II were designated m1 and m2 (m for *m*id). The types in region IV were designated c1 and c2 (c for *C*-terminus); c3 was another variant type in region IV, composed of parts of c1 and c2. In this designation, previous *hopM *and *hopN *genes correspond to *hopMNm1-c1 *and *hopMNm2-c1*, respectively. All hpEastAsia strains except the strains 52 and PeCan4 (9/11) carry sequence type c2 at region IV. The c3 variant is observed in J99, PeCan4 and SJM180 (Figure 2A and 2F).

Three *vacA *paralogs and *vacA *itself were found in 26695 [28]. Those paralogs share the auto-transporter domain at the C-terminus with *vacA *[28]. A large deletion in *vacA-2 *(HP0289) (approximately 2400 amino acids) was found in all the hspEAsia strains except the strain 51 (5/6) (Table 2 and Additional file 2 (= Table S1)).

**Figure 3:**
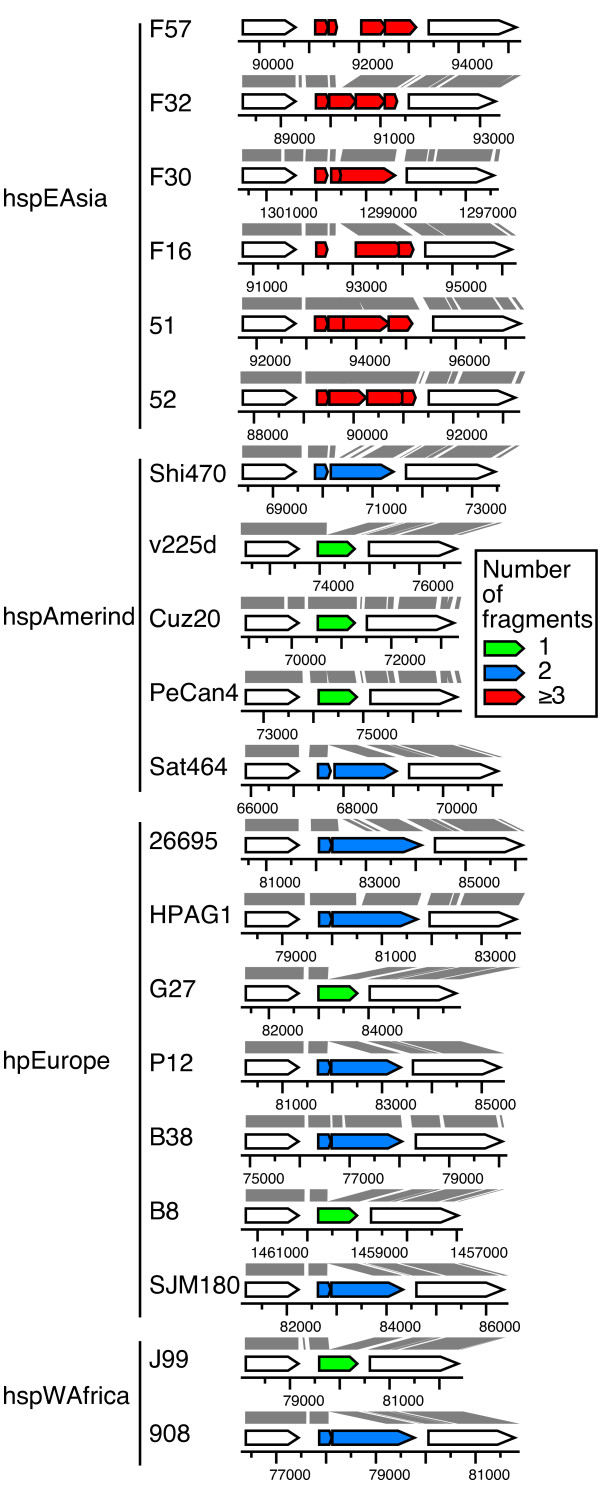
**Fragmentation of *horA *OMP gene through various mutations in the hspEAsia strains**. Genes homologous to *horA *in J99 (jhp0073) are classified by the number of ORFs. Numbers indicate coordinates on the genome sequence. Nucleotide similarity between each pair of strains is indicated by gray parallelogram. The state in strain 98-10 is: two ORFs.

It was described earlier that *horA *OMP locus in 26695 is composed of two open reading frames (ORFs) (HP0078/HP0079) whereas that in J99 is composed of one ORF (jhp0073) [27]. The *horA *locus in all the hspEAsia strains shows apparent gene decay by fragmentation through various mutations (Figure 3). Whether the genes in the other strains are functional is not known.

A putative periplasmic endonuclease gene (*nucG*, HP1382) was split in all the hspEAsia strains examined (Table 2 and Additional file 2 (= Table S1)). Detailed analysis revealed that the split was mediated by recombination between short similar sequences [25].

### Massive decay of molybdenum-related genes for two-electron reduction-oxidation reactions

Unexpectedly, our profiling suggested that functions related to molybdenum (Mo) were lost specifically in the hspEAsia strains (Table 3 and Additional file 2 (= Table S1)). The trace element Mo is essential for nearly all organisms [29]. After transport into the cell as molybdate, it is incorporated into metal cofactors for specific enzymes (molybdo-enzymes) that catalyze reduction-oxidation (redox) reactions mediated by two-electron transfer.

**Table 3:** Decay of molybdenum-related genes

Type	hspEAsia						hspAmerind	hpEurope		hspWAfrica
Strain	F57	F32	F30	F16	51	52	^(a)^	^(b)^	P12	^(c)^

Molybdenum (MoO_4_^2-^) transport										

*modA*	x	x	x	+	+	x	+	+	+	+
*modB*	x	+	+	+	x	x	+	+	+	+
*modC*	x	x	x	x	x	+	+	+	+	+

Molybdenum cofactor synthesis										

*moaA*	x	x	x	x	+	x	+	+	+	+
*moaC*	x	+	+	+	+	+	+	+	+	+
*moaE*	x	+	+	+	+	+	+	+	+	+
*moaD*	+	x	+	+	+	+	+	+	x	+
*moeB*	+	+	+	+	+	+	+	+	+	+
*mogA*	x	+	x	x	x	+	+	+	+	+
*moeA*	x	x	x	x	x	x	+	+	+	+
*mobA*	+	+	+	+	+	x	+	+	+	+

Molybdenum cofactor-containing enzyme										

*bisC*	x	x	x	x	x	x	+	+	+	+

+, present; x, disrupted (nucleotide sequence remained).

a) Strains Shi470, v225d, Cuz20, Sat464 and PeCan4.

b) Strains 26695, HPAG1, G27, B38, B8 and SJM180.

c) Strains J99 and 908

The states in strain 98-10 are: x for *modA*, *modB*, *mobA*, *moaA*, *moeB *and *bisC*; + for *modC*, *moaD*, *moaE*, *mogA*, *moaC *and *moeA*.

**Figure 4:**
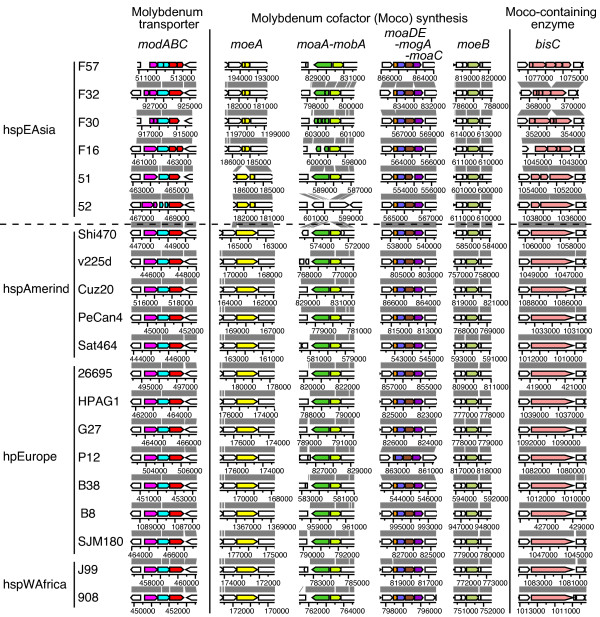
**Decay of Mo-related genes in the hspEAsia strains**. Mo-related genes are indicated by color. Homologs are indicated by the same color. See Additional file 3 (= Table S2) for nucleotide sequences.

In the 20 *H. pylori *genomes, the only gene for molybdo-enzymes identified was *bisC*. At least one gene in each of the three Mo-related functions, Mo transport, Mo cofactor synthesis and a Mo-containing enzyme, decayed in all hspEAsia strains (Table 3 and Figure 4). Detailed analysis of nucleotide sequences revealed a mutation in 10 of 12 Mo-related genes in some of the hspEAsia strains (Table 3 and Additional file 3 (= Table S2)). The occurrence of apparently independent multiple mutations (Additional file 3 (= Table S2)) suggests some selection against use of Mo in the hspEAsia strains. All other strains but P12 possessed all intact genes. The strain P12 had a truncation of *moaD *(Additional file 3 (= Table S2)). Tungsten sometimes substitutes for Mo, but genes for known tungstate/molybdate binding proteins (TupA and WtpA) were not found in the *H. pylori *genomes.

The sequences in the four Japanese strains were confirmed by polymerase chain reaction (PCR) with the primers listed in the Additional file 4 (= Table S3).

The Mo-related genes were in a list of "chronic gastritis-associated" genes [30], primarily because they are absent from three Amerind strains from the Athabaskan people [31]. The 5 Amerind strains analyzed in the present study are different from the three Amerind strains in this respect. This difference could reflect the later migration of the Athabaskans to the Americas [32].

### Two pathways between acetyl~CoA and acetate in some Japanese strains

**Figure 5:**
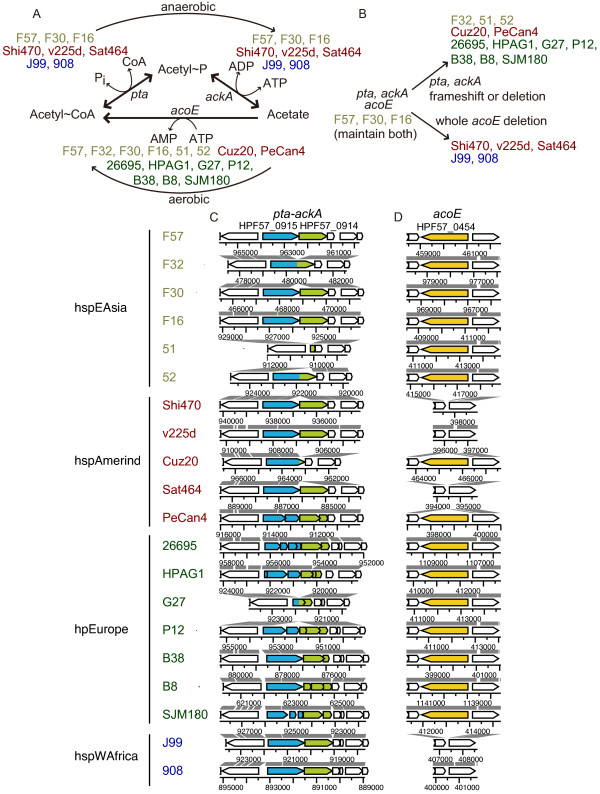
**Variation in genes connecting acetyl-CoA and acetate**. (A) Functional states of three genes in two pathways inferred for 20 strains. (B) Reconstruction of pathway evolution. (C) Genome comparison for the *pta*-*ackA *region. (D) Genome comparison for the *acoE *region. Homologs are indicated by the same color in (C) and (D). The states in strain 98-10 are: *pta*^+ ^*ackA*^+^/*acoE*^+ ^as F57.

Our profiling revealed an important change at the center of energy and carbon metabolism related to acetyl~CoA. Two pathways connect acetyl~CoA and acetate (Figure 5A). In anaerobic fermentation, acetyl~CoA is converted into acetate by phosphoacetyl transferase (*pta *product) and acetyl kinase (*ackA *product) with generation of ATP (anaerobic *pta*-*ackA *pathway) [33]. The intermediate acetyl~P, a high-energy form of phosphate, likely serves as a global signal. Although these reactions are reversible, assimilation of acetate may be irreversibly mediated by acetyl~CoA synthetase (*acoE *product) by the generation of acetyl~CoA, which enters the TCA cycle to generate energy under aerobic conditions (aerobic *acoE *pathway).

It has been suggested that strain 26695 (hpEurope) carries a mutation in *pta *for the former pathway whereas strain J99 (hspWAfrica) lacks *acoE *for the latter [28,34]. All European strains in this study (7/7) had at least one inactivated *pta *and *ackA *gene through a variety of mutations (Figure 5C). Two of five Amerind strains, PeCan4 and Cuz20, also had a mutated *pta *and *ackA*, whereas the other 3/5 Amerind, 2/2 African, and 3/6 hspEAsia strains had a *pta *and *ackA *intact but had a deletion of *acoE*. Exceptions to such apparent incompatibility between the two pathways were found for 3/4 of the Japanese strains (F16, F30 and F57), which had intact genes for both pathways (Figure 5BCD). The sequences in the four Japanese strains were confirmed (see Methods and Additional file 4 (= Table S3)).

### **A gene for an amino acid utilization**

An ortholog of jhp0585 in J99 is absent from 26695 [2]. An ortholog is present in the six other hpEurope strains and both hspWAfrica strains, but absent from all hpEastAsia strains (hspEAsia and hspAmerind) (Additional file 2 (= Table S1)). It encodes a homolog of 3-hydroxy-isobutyrate dehydrogenase and the related beta-hydroxyacid dehydrogenase (COG2084). The 3-hydroxy-isobutyrate dehydrogenase degrades the branched-chain amino acid valine. *H. pylori *requires branched amino acids for growth. It is not known what the substrates or products of reactions catalyzed by this gene product are, or the biological relevance of its distribution.

### Gene contents unique to other groups

Phylogenetic profiling involving four groups (6 hspEAsia, 5 hspAmerind, 7 hpEurope, and 2 hspWAfrica strains) (Additional file 2 (= Table S1), second sheet) revealed the following group-specific genes:

(i) *tas *(HP1193) for aldo-ketoreductase was present in all hpEurope strains except one (HPAG1) and one hspWAfrica strain (J99), but was absent from all hpEastAsia strains (hspEAsia and hspAmerind). Aldo-keto reductases (AKRs) constitute a large protein superfamily of mainly NAD(P)-dependent oxidoreductases involved in carbonyl metabolism [35]. This gene is fragmented in *H. acinonychis *strain Sheeba [36].

(ii) *homB *encoding an outer membrane protein was present in all but two (B8 and SJM180) hpEurope strains (5/7) but absent from the others. This result is in agreement with an earlier study [17].

(iii) *trl *was detected in all hpEastAsia (hspEAsia and hspAmerind) strains and 2/7 hpEurope strains (26695 and HPAG1). It is present between tRNA(Gly) and tRNA(Leu), and co-transcribed with tRNA(Gly) [37]. It is found in roughly half the clinical isolates in Ireland [37]. Its homologs are present at two loci in 26695 [38].

(iv) A part of *xseA *for Exonuclease VII large subunit was duplicated in all the hspAmerind strains but the strain PeCan4. *Escherichia coli *exonuclease VII degrades single-stranded DNA and contributes to DNA damage repair and methyl-directed DNA mismatch repair to avoid mutagenesis [39-41]. This part of *xseA *was present in the neighbor of 3 other genes in these hspAmerind strains. These 4 genes may form a genomic island.

(v) IS*606 *transposase gene was present in all hspAmerind and hspWAfrica strains, and one hpEurope (26695) strain, but was absent from the others.

(vi) Most of *fecA-2 *gene, a *fecA *paralog, was deleted in the hspAmerind strains. The *fecA *gene, for Iron (III) dicitrate transport protein, is important under aerobic conditions [42]. There are several links between iron metabolism and oxidative stress defense in *H. pylori *[43].

(vii) The *hopZ *OMP gene was split in the hspAmerind strains. The *hopZ *gene is involved in adhesion [44].

(viii) The *hopQ *OMP gene decayed in the hpEastAsia strains (hspEAsia and hspAmerind). This observation agrees with an earlier work [45].

(ix) *H. pylori *can ferment pyruvate to ethanol via an alcohol dehydrogenase [46]. Duplication of the alcohol dehydrogenase gene as in J99 (jhp1429) [2] was seen only in the two hspWAfrica strains (J99 and 908).

### Prophage-related genomic islands and other mobile elements

**Figure 6:**
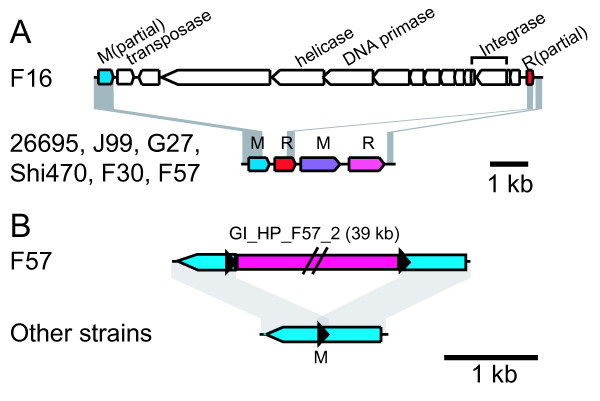
**GIs inserted into restriction-modification systems**. (A) Insertion of a prophage-like GI (GI_HP_F16_1) into a restriction-modification system. (B) Insertion of a GI into a modification gene. (See Table 4 for detail).

**Figure 7:**
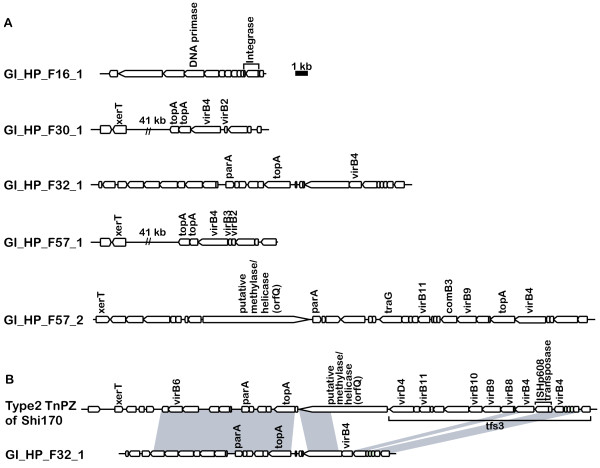
**GIs detected in Japanese *H. pylori *strains**. (A) GIs. (B) Decay of type 2 TnPZ in F32 strain inferred from comparison to the Shi170 strain. The sequence of Type 2 TnPZ of Shi170 is deposited under the accession number [GenBank:EU807988] [48] (Table 4).

Except for the cag pathogenicity island (cagPAI), five genomic islands (GIs) were identified in the genomes of the four Japanese strains (Table 4, Figure 6 and Figure 7). In F32, the cagPAI was flanked by a 44-bp direct repeat, which extended the 22-bp sequence found in the other strains (Table 4). This length of sequence identity would allow homologous recombination [47] leading to the excision of cagPAI flanked by the repeat.

**Table 4:** Genomic islands in the four Japanese *H. pylori *strains

Strain	GI number	Type	Length	Start-end	Flanking repeat (bp)	Secretion system	Left gene (annotation)	Right gene (annotation)
F16	GI_HP_F16_1	prophage-like	12245	471964 - 484208 (HPF16_0465 - HPF16_0478)	N/D^(a)^	N/D^(a)^	HPF16_0464 (IS*605 *transposase (*tnpB*))	HPF16_0479 (*typeIIR*)
	GI_HP_F16_2	cagPAI	36761	871413 - 834651 (HPF16_0834 - HPF16_0810)	22^(b)^	Type IV	HPF16_0835 (hypothetical protein)	HPF16_0809 (glutamate racemase)

F30	GI_HP_F30_1 (left)	type 1b TnPZ partial	7246	1280406 - 1287651 (HPF30_1205 - HPF30_1211)	N/D^(a)^	tfs3b partial	HPF30_1204 (outer membrane protein *horB*)	HPF30_1212 (*rpoD*)
	GI_HP_F30_1 (right)	type 1b TnPZ partial	1655	1237267 - 1238921 (HPF30_1166 - HPF30_1167)	N/D^(a)^	N/D^(a)^	HPF30_1165 (hypothetical protein)	HPF30_1168 (5'-methylthioadenosine/S -adenosylhomocysteine nucleosidase)
	GI_HP_F30_2	cagPAI	37153	867993 - 830839 (HPF30_0803 - HPF30_0778)	22^(c)^	Type IV	HPF30_0804 (hypothetical protein)	HPF30_0777 (glutamate racemase)

F32	GI_HP_F32_1	type 2 TnPZ partial	24283	1058236 - 1082518 (HPF32_0988 - HPF32_1014)	N/D^(a)^	tfs3 partial	HPF32_0987 (hypothetical protein)	HPF32_1015 (hypothetical protein)
	GI_HP_F32_2	cagPAI	36609	534488 - 571096 (HPF32_0500 - HPF32_0524)	44^(d)^	Type IV	HPF32_0499 (hypothetical protein)	HPF32_0525 (glutamate racemase)
F57	GI_HP_F57_1 (left)	type 1b TnPZ partial	7246	103791 - 111036 (HPF57_0102 - HPF57_0109)	N/D^(a)^	tfs3b partial	HPF57_0101 (RNA polymerase sigma factor RpoD)	HPF57_0110 (hypothetical protein)
	GI_HP_F57_1 (right)	type 1b TnPZ partial	1625	152699 - 154323 (HPF57_0147 - HPF57_0148)	N/D^(a)^	N/D^(a)^	HPF57_0146 (5'-methylthioadenosine/S-adenosylhomocysteine nucleosidase)	HPF57_0149 (hypothetical protein)
	GI_HP_F57_2	type 1 TnPZ	38991	284353 - 323343 (HPF57_0279 - HPF57_0311)	8^(e)^	tfs3b partial	HPF57_0278 (*typeIIM*)	HPF57_0312 (type II DNA modification enzyme )
	GI_HP_F57_3	cagPAI	36797	562215 - 599011 (HPF57_0550 - HPF57_0575)	22^(c)^	Type IV	HPF57_0549 (hypothetical protein)	HPF57_0576 (glutamate racemase)

a) N/D, not detected

b) TTATAATTTGAGCCATTATTTA

c) TTTCAATTTGAGCCATTCTTTA

d) TTATAATTTGAGCCATTCTTTAGCTTGTTTTTCTAGCCAAACCA

e) ACATTCTT

A GI found in strain F16 lacked similarity to known GIs of *H. pylori *whereas the other four GIs were homologous to transposable elements TnPZs, as recently reported [48,49]. The GI in F16 appears to be a remnant of a prophage inserted into a restriction-modification system (Figure 6A). It is homologous to the 5'-half of the Hac II prophage found in *H. acinonychis *Sheeba. The F16 GI appeared to have lost its 3'-half, presumably through deletion mediated by the inserted IS*605 *copy. The GI included putative phage integrase genes (HPF16_0475 and HPF16_0476) that suggest the mobility of this region, and a DNA primase gene (HPF16_0468). The gene (HPF16_0469) next to the DNA primase gene had weak sequence similarity to a putative phage helicase gene (ORF35 of bacteriophage phi3626, e-value 5e-5 by TBLASTN against phage nucleotide database), which can be assumed to be the primase-helicase system found in several bacteriophages such as T3, T4, T7 and P4 [50]. Recently, a partial Hac II prophage region was reported for another *H. pylori *strain [51].

The other four GIs in the other three strains had sequence similarity to TnPZs [48]. One GI in F57 was entirely homologous to the type 1 TnPZ inserted into the coding region for a DNA methyltransferase with 8-bp target duplication (5' ACATTCTT) (Figure 6B). The GI in F32 appeared to have been deleted by a type 2 TnPZ (Figure 7B). Among the Korean strains, a Type 2 TnPZ was observed only in strain 51.

The plasmid in F32 (pHPF32) was similar in sequence to known theta replication plasmids with a RepB family (Rep_3 superfamily) replication protein and R3 iterons [52-54].

The plasmid in F30 (pHPF30) was similar to a group of previously characterized *H. pylori *plasmids such as pHel4 in *H. pylori *[52,55]. This carries genes for microcin (7-aa peptide; MKLSYRN), MccB (microcin C7 biosynthesis protein), MccC (microcin C7 secretion protein), MobBCD (for plasmid mobilization), a replication initiator protein, and two relaxases. When compared to other related plasmids, a substitution in *mobB *and a deletion covering several small ORFs were seen. Homologous plasmids are found in G27 (pHPG27 [56]), P12 (HPP12 [49]), and v225d [22]. HPAG1 [30], B8 [57], PeCan4 and Sat464 carry a similar plasmid without the MccBC genes.

Insertion sequences (ISs) were searched for in the Japanese strains using GIB-IS [58]. An apparently intact known IS was detected in two strains: IS*607 *in F16; IS*605 *in F32.

### Divergence of genes between the East Asian (hspEAsia) and the European (hpEurope) strains

**Figure 8:**
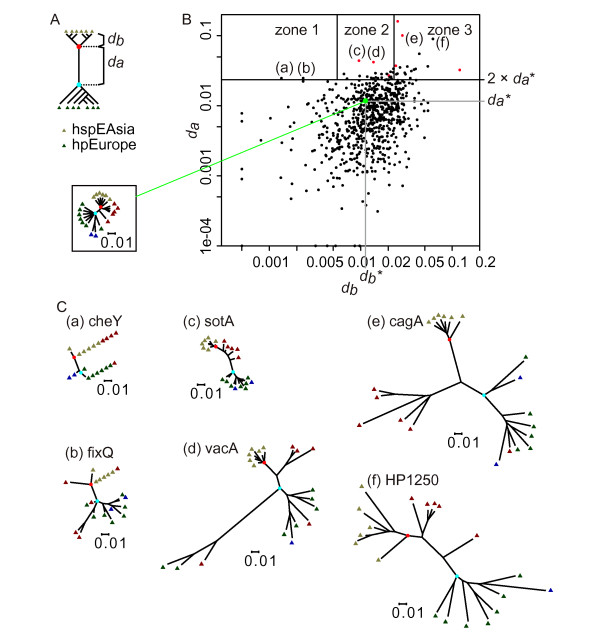
**Genes diverged between East Asian and European strains**. (A) Diagram of phylogenetic tree-based analysis. Black dots, last common ancestors of Eastern and Western strains. *d*_*a*_, length of the branch separating the two; *d*_*b*_, average branch length of the Eastern strains. (B) Plot of gene trees based on the two distance values. Large green dot, well-defined core tree; *d*_a_*, *d*_*a *_for the well-defined core tree; *d*_b_*, *d*_*b *_for the well-defined core tree; inset box, well-defined core tree; zone 1, *d*_*b *_< 0.00550; zone 2, 0.00550 ≤ *d*_*b *_≤ 0.0231; zone 3, *d*_*b *_> 0.0231; red dot, genes with positive selection for amino acid change and with *d*_*a *_> 2 × *d*_a_*, that is, *d*_*a *_*>*0.02324; (a), *cheY*; (b), *fixQ*; (c), *sotA*; (d), *vacA*; (e), *cagA*; (f), HP1250. N = 692 genes. (C) Representative trees with high divergence between hspEAsia and hpEurope strains. Lowest common ancestor (LCA) of hspEAsia (red) and hpEurope (cyan).

We systematically examined the amino acid-based phylogenetic trees of the orthologous genes (gene families) common to the six hspEAsia genomes and the seven hpEurope genomes. Trees of 687 OGs were selected with genes of the hspEAsia strains forming a sub tree with no genes of the hpEurope strains and *vice versa*. Each of the orthologs was plotted according to two distance parameters: *d*_*a *_for the hspEAsia-hpEurope divergence and *d*_*b *_for intra-hspEAsia divergence (Figure 8A). An hspEAsia-hpEurope divergence greater than twice that of the well-defined core tree (*d*_a_*) was seen in 47 gene families (Table 5 and 6; genes of those orthologs in each strain are listed in Additional file 5 (= Table S4)). These genes were further divided by the intra-hspEAsia divergence (*d*_*b*_) into zone 1 (lowest divergence), zone 2 (average divergence) and zone 3 (highest divergence) (Figure 8B). Six typical trees are depicted in Figure 8C. The *cagA *tree (e) (zone 3) has large *d*_*a *_and *d*_*b *_values and a low *d*_*b*_/*d*_*a *_value, primarily because of the divergence in a C-terminal region of the ORF. This region, including sequences known as EPIYA (Gln-Pro-Ile-Tyr-Ala) motif, is involved in host interaction [22,59]. The tree here is consistent with previous results [22].

**Table 5:** Selected genes diverged between East Asian (hspEAsia) and European (hpEurope) *H. pylori*

Function	Genes (classified by divergence within hspEAsia)		
	Conserved^(a)^	Average^(b)^	Diverged^(c)^

Known virulence genes		***vacA***, ***tipα***	***cagA***, ***hcpD***

Outer membrane proteins		***oipA/oipA-2***,*** vacA***, ***vacA-4***	***hpaA-2***, ***homC***,*** hopJ/hopK***, ***horI***
Lipopolysaccharide synthesis (Lewis antigen mimicry)		***agt***	***futA/futB***
Transport		***secG*, *sotB*, *comH****, cvpA*	***yajC***
Motility and chemotaxis	*cheY*	*maf*, *fliT*	***fliK***
Redox	*fixQ*	***hypD***, ***frxA***, *pgl, nuoF*	***fixS, hydE***
Nuclease		*rnhA*	***addA***, ***rnhB, hsdR***
Protein synthesis		***def***, ***prmA***, ***tilS***	***miaA***
Antibiotic-related		***def***, ***frxA****, ftsA*	

Full list and details in Table 6, Additional file 5 (= Table S4) and text. Genes in bold were also extracted in the comparison of 6 hspEAsia *vs*. 5 hpEurope (Additional file 7 (= Table S5)).

(a) *d*_*b *_<*d*_b_*. Zone 1 in Figure 3. (b) *d*_*b *_≈ *d*_b_*. Zone 2. (c) *d*_*b *_>*d*_b_*. Zone 3.

**Table 6:** Genes diverged between East Asian and European *H. pylori*

Gene	Description	Representative of the gene family^(a,b,c)^	Distance *d*_*a*_^(d)^	Distance *d*_*b*_^(e)^	*d*_*b *_zone	Reference
*hpaA-2*	HpaA paralog	HP0492^(f)^	0.1608	0.0253	3	[68]
*cagA*	Cag pathogenicity island protein	HP0547^(f)^	0.1009	0.0285	3	[11]
	Bacterial SH3 domain	HP1250	0.0901	0.0615	3	
*futA, futB*	α-(1,3)-fucosyltransferase	HP0379, HP0651	0.0553	0.0436	3	[15]
*sotB*	Sugar efflux transporter	HP1185	0.0441	0.0095	2	
*vacA*	Vacuolating cytotoxin A	HP0887	0.0420	0.0137	2	[67]
*miaA*	tRNA delta(2)-isopentenylpyrophosphate transferase	mHP1415	0.0373	0.0241	3	[64,144]
	Hypothetical protein	HPAG1_0619	0.0366	0.0540	3	
*hcpD*	Cysteine-rich protein, SLR (Sel1-like repeat) protein	HP0160	0.0363	0.0323	3	[16]
*yajC*	Preprotein translocase subunit YajC	HP1551	0.0353	0.0268	3	[81]
*agt*	β-1,3-N-acetyl-glucosaminyl transferase	HP1105	0.0338	0.0228	2	
*rnhB*	Ribonuclease HII	mHP1323^(f)^	0.0337	0.0398	3	[103,104]
*fliK*	Flagellar hook length control	HP0906	0.0328	0.0382	3	[85]
*homC*	Putative outer membrane protein	HP0373	0.0325	0.1207	3	
*hopJ,hopK*	Outer membrane protein	HP0477, HP0923	0.0313	0.0357	3	[27]
*frxA*	NAD(P)H-flavin oxidoreductase	HP0642	0.0306	0.0212	2	[120]
*secG*	Preprotein translocase subunit SecG	mHP1255	0.0300	0.0226	2	[80]
	Hypothetical protein	HP0384	0.0296	0.0302	3	
*tipα*	Tumor necrosis factor alpha-inducing protein	HP0596	0.0293	0.0145	2	[66]
*hydE*	Membrane-bound, nickel containing, hydrogen uptake hydrogenase	HP0635	0.0288	0.0252	3	[92]
*tilS*	tRNA(Ile) lysidine synthase	HP0728	0.0286	0.0193	2	[96,97]
*comH*	Periplasmic competence protein	HP1527	0.0285	0.0194	2	[82]
*def*	Peptide deformylase	HP0793	0.0285	0.0065	2	[98]
*vacA-4*	Putative vacuolating cytotoxin-like protein	HP0922	0.0284	0.0222	2	
*hypD*	Hydrogenase expression/formation protein	HP0898	0.0284	0.0169	2	[91,145,146]
*addA*	Helicase	HP1553	0.0283	0.0308	3	[100]
*hsdR*	Type I restriction enzyme, R protein	mHP1402	0.0282	0.0245	3	
	Hypothetical protein	mHP0174	0.0268	0.0203	2	
*oipA,oipA-2*	Outer membrane protein OipA	HP0638	0.0267	0.0097	2	[70]
*prmA*	Ribosomal protein L11 methyltransferase	HP1068	0.0261	0.0118	2	[99]
*maf*	Maf family (motility accessory family of flagellin-associated proteins) homolog	HP0465	0.0259	0.0214	2	[86]
	Hypothetical protein	HP0097	0.0257	0.0207	2	
	Hypothetical protein	HP1143	0.0254	0.0146	2	
*cvpA*	Membrane protein required for colicin V production and secretion	mHP0181	0.0252	0.0169	2	[83]
*pgl*	6-phosphogluconolactonase	HP1102	0.0250	0.0130	2	
*horI*	Outer membrane protein Horl	HP1113	0.0248	0.0348	3	
*fixQ*	cbb3-type cytochrome c oxidase subunit Q	mHP0146	0.0248	0.0023	1	
	Hypothetical protein	HP0150	0.0248	0.0154	2	
*cheY*	Chemotaxis effector	HP1067	0.0248	0.0014	1	[84]
*fliT*	Flagellar chaperone	HP0754	0.0245	0.0138	2	[84]
*ftsA*	Cell division protein	HP0978	0.0244	0.0071	2	[105,106]
*rnhA*	Ribonuclease H	HP0661	0.0243	0.0217	2	[103,104]
*ilvE*	Branched-chain amino acid aminotransferase	HP1468	0.0239	0.0136	2	
*fixS*	Cation transport subunit for cbb3-type oxidase	HP1163	0.0237	0.0250	3	[87]
*nuoF*	NADH-ubiquinone oxidoreductase chain F	HP1265	0.0236	0.0202	2	
	Putative thiol:disulfide interchange protein	HP0861	0.0234	0.0185	2	
	Hypothetical protein	HP0806	0.0233	0.0233	3	

(a) m, different assignment of start codon from the RefSeq entry in the GenBank database

(b) All paralogous genes in each orthologous group are counted.

(c) Assignments to gene families are in Additional file 5 (= Table S4).

(d) Distance between the last common ancestor of hspEAsia and the last common ancestor of hpEurope.

(e) Average of distances between the last common ancestor of hspEAsia and each hspEAsia strain.

(f) A homolog in the draft genome sequence of another East Asian strain 98-10 has been reported to be diverged from four Western strains [143]. The other genes listed as diverged in 98-10 [143], HP0806, HP0061, HP1524, HP0519 and HP1322, did not meet the criteria of this study. HP0806 was below the *d*_*a *_threshold; for the others, the hspEAsia genes did not form a separate sub tree from hpEurope.

This tree-based analysis effectively extracted known pathogenesis-related genes (Table 5 and Table 6) as discussed below. The list also included several genes related to antibiotics. Amino acid alignments (Additional file 6) located the divergent sites. The distribution pattern of these sequences suggests a possible relationship between structure and function as detailed below for each protein. The divergence could be related to differential activity and adaptation.

The variable *d*_*a *_for an orthologous group is expected to be sensitive to the presence of a member with an exceptional phylogeny. The strain B8, assigned to hpEurope in this work (Additional file 1 (= Figure S1)), has been adapted to a mongolian gerbil [57]. The strain SJM180, also assigned to hpEurope based on the tree of seven MLST genes (Additional file 1 (= Figure S1)), clustered with hspWAfrica strains rather than with hpEurope strains in the tree of the well-defined core genes (Figure 1). To examine robustness of the above classification into diverged genes, the same analysis was conducted using the 6 hspEAsia strains and 5 hpEurope strains excluding B8 and SJM180 (Additional file 7 (= Table S5)). These two analyses used all the 20 strains, because we expected inclusion of the hspAmerind and hspWAfrica strains may provide better classification of the sub trees. In addition to these two analyses, analysis with the 6 hspEAsia and 7 hpEurope strains or with the 6 hspEAsia and 5 hpEurope strains was carried out, which allowed assignment of a bootstrap value to the branch separating the hspEAsia and hpEurope strains. Comparison of these 4 analyses is summarized in Additional file 7 (= Table S5). The four sets of results agreed rather well, especially for those genes with larger *d*_*a *_value: 34 among the 47 genes in Table 6 were extracted in all the 4 analyses. The bootstrap value supported the separation of hspEAsia and hpEurope well in most cases, with the bootstrap value ≥ 900 in 41 among the 47 genes.

### Positively-selected amino-acid changes between the East Asian (hspEAsia) and European (hpEurope) strains

**Figure 9:**
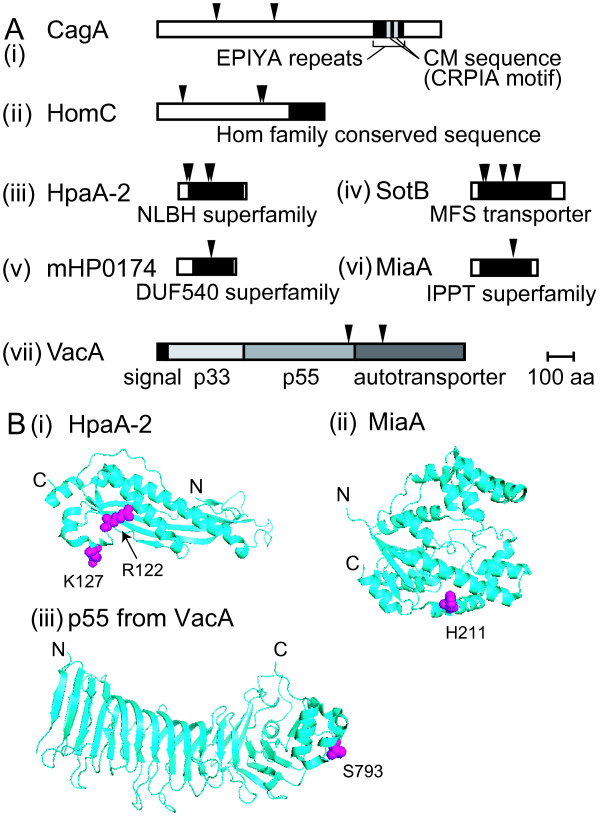
**Genes with positively selected amino acid changes between East Asian and non-East Asian strains**. (A) Position of the positively selected amino-acid residues in ORF (triangles). In (i), EPIYA segments and CM sequences [138] are marked. (B) Position of positively selected amino acids in the three-dimensional structure. (i) HpaA-2 [PDB:2I9I]. (ii) *E. coli *MiaA [PDB:3FOZ] [61] with the residue corresponding to H174 of *H. pylori *MiaA. (iii) p55 fragment of VacA [PDB:2QV3] [61] (Table 7).

Divergence could be adaptive or neutral. We searched for sites where the hspEAsia-hpEurope changes in amino acids were positively selected [60] and found that 7 of 47 genes passed the likelihood test (Table 7; red dots in Figure 8B). These selected sites were mapped on the coding sequences (Figure 9A). For CagA, several sites were found outside the area of EPIYA segments.

**Table 7:** Genes with positively selected amino-acid changes between the East Asian and the European *H. pylori*

Locus tag	Gene	Description	p-value^(a)^	Positively selected sites ^(b,c)^
HP0547	*cagA*	Cag pathogenicity island protein	< 1E-21	V238R (0.994)
				A482Q (0.953)

HP0373	*homC*	Putative outer membrane protein	< 1E-14	E110N (0.978)
				K428H (0.986)
				T437D (0.979)

HP0492	*hpaA-2*	Hpa paralog	< 1E-5	S34V (0.970)
				A46Q (0.993)
				R122F (0.967)
				K127S (0.962)

HP1185	*sotB*	Sugar efflux transporter protein	0.00005	T50S (0.956)
				A57L (0.990)
				N134G (0.983)
				W186Y (0.980)

mHP0174		Hypothetical protein	0.0007	F144W (0.952)

mHP1415	*miaA*	General tRNA delta(2)-isopentenylpyrophosphate transferase	0.0002	H174A (0.992)

HP0887	*vacA*	Vacuolating cytotoxin A	0.002^(d)^	S793A (0.964) ^(d) ^N931A (0.960) ^(d)^

a) Bonferonni adjusted.

b) Posterior probabilities of dN/dS > 1.

c) Positions are for *H. pylori *26695. Residues were aligned at the same site by both Mafft [128] and PRANK [136].

d) Two *vacA *genes (in B38 and B8) were eliminated because they belonged to different subtypes of the gene.

Three-dimensional structure was available for mapping some of the selected sites for three of these genes (Figure 9B). The three-dimensional structure of part of VacA, the p55 fragment, is determined [61]. S793A mapped on the surface of the p55 at its C-terminal region (Figure 9B). Deletion of the p55 region reduces VacA binding to cells [62], so S793A might affect cell binding of the hspEAsia and hpEurope strains. Two selected residues of HpaA-2 were mapped (Figure 9B). The residue (H211) corresponding to the selected residue H174 of *H. pylori *MiaA mapped to the alpha helix 10 of *E. coli *MiaA [63,64] (Figure 9B).

### Diverged genes and possible biological significance

We explored the possible biological significance of the observed divergence in genes in Table 6 using gene and protein properties, as summarized in Table 5.

#### Known virulence genes

Four genes in Table 6, *cagA*, *vacA*, *hcpD *and *tipα*, are virulence genes.

CagA is introduced in the Background section and discussed above in the section "Divergence of genes between the East Asian (hspEAsia) and the European (hpEurope) strains". VacA is another important virulence protein [65]. The *hcpD *(HP0160) is a member of the Hcp (*H. pylori *cysteine-rich protein) family, which contains repeat motifs characteristic to the eukaryotic Sel1 regulatory proteins, is secreted and interacts with the host immune systems [16]. Geographical divergence and positive selection for amino acid changes in this family, including HcpD, are reported [16]. HP0596 encodes tumor necrosis factor alpha-inducing protein (Tipα), a DNA-binding protein [66]. This enters the gastric cells and induces TNF-alpha, an essential cytokine for tumor promotion.

The *cagA *gene is discussed above in the section "Divergence of genes between the East Asian (hspEAsia) and the European (hpEurope) strains". The *vacA *gene showed a qualitatively similar pattern of intra-hspEAsia divergence and overall divergence as *cagA *(Figure 8C (d)). The overall tree pattern was consistent with previous studies (for review, see [67]). Intra-hspEAsia divergence was large for *hcpD*. Positively-selected residues of *cagA *and *vacA *are described above.

#### Outer membrane proteins

Nine genes in Table 6 are outer membrane protein genes (Table 5).

The *vacA *gene is discussed above. *vacA-4 *is a *vacA *paralog. The *hpaA-2 *is of unknown function [68], but is a paralog of *hpaA *[27] which is essential for adhesion [69]. The *homA/B *genes are homologs of *homC *and known to have diverse copy number and genomic localization in Western and East Asian strains (Table 1) [17]. OipA (also known as HopH) induces IL-8 from host cells [70]. Geographical divergence of *oipA *has been reported [14].

The *hpaA-2 *showed a very large hspEAsia-hpEurope divergence (the largest *d*_*a *_value; Figure 8B and Table 6).

Intra-hspEAsia divergence was intermediate for *oipA/oipA-2 *(Table 6).

The *d*_*a *_value (hspEAsia-hpEurope divergence) of *homC *(0.0325) was larger than the threshold distance (Table 6). Moreover, the *homC *genes of all hpEastAsia and hpAfrica1 strains but the strain 52 were greatly diverged from those of the hpEurope strains and the strain 52: distance 0.1387 for this separation was comparable to the largest *d*_*a *_values for *hpaA-2 *and *cagA*. Diverged residues were clustered in a specific region. Positively selected amino-acid changes of the putative *homC *product were identified (Table 7 and Figure 9).

The *hopJ *and *hopK *genes (HP0477 and HP0923) were similar within each strain but different between strains [26,27]. This earlier observation, seen for 26695, J99 and HPAG1, was confirmed with the other genomes except for 908 and B8. This similarity of *hopJ *and *hopK *genes in one strain is likely to be caused by concerted evolution by homologous interaction, possibly with selection.

The *babA *and *alpA *genes were not included in the 687 OGs that showed complete separation between genes of the six hspEAsia strains and those of the seven hpEurope strains on the phylogenetic tree. BabA binds to Lewis b antigens [71,72]. Geographic variation of BabA has been reported [13]. AlpAB proteins are necessary for specific adherence to human gastric tissue [73]. In the East Asian strains but not the Western strains, AlpA activates NF-κB-related pro-inflammatory signaling pathways [74].

The reason that the *babA *is not in Table 6 was mainly because *babA *genes of the hpEurope strains B8 and SJM180 grouped together with the hspEAsia strains (Additional file 7 (= Table S5)). The *alpA *in the hpEurope strain SJM180 grouped with the hspEAsia strains (Additional file 7 (= Table S5)).

#### Lipopolysaccharide synthesis and Lewis antigen mimicry

Three genes in Table 6, *futA*, *futB *and HP1105 (designated here as *agt*), are related to lipopolysaccharide (LPS) synthesis and Lewis antigen mimicry.

The lipopolysaccharides of *H. pylori *are important for host interaction. *H. pylori *can express Lewis and related antigens in the O-chains of its surface lipopolysaccharide that mimic the hosts. O-chains are commonly composed of internal Lewis X units with terminal Lewis X or Lewis Y units or, in some strains, with additional units of Lewis a, Lewis b, Lewis c, sialyl-Lewis X and H-1 antigens, as well as blood groups A and B, producing a mosaic of antigenic units [75]. The activity and specificity of the fucosyltransferases may vary between the two paralogs in one strain, as well as between the orthologs in different strains [76]. Mechanism of these changes is phase variation involving simple repeats and longer repeats [77,78]. Such diversity could be adaptive and related to differences in pathogenicity [79].

The two fucosyltransferase genes (*futA *= HP0379, *futB *= HP0651) showed large hpEurope-hspEAsia divergence (the 4th largest *d*_*a *_value), as reported earlier [15]. Intra-hspEAsia divergence was large for them (in zone 3). HP1105 (*agt*) was β-1,3-N-acetyl-glucosaminyl transferase gene for LPS synthesis. Another transfereaseα-1,6-glucosyltransferase gene (HP0159 = *rfaJ-1*) was in the list of 6 hspEAsia - 5 hpEurope comparison (Additional file 7 (= Table S5)).

#### Transport

Four genes in Table 6, *sotB*, *secG*, *yajC*, *comH *and *cvpA*, are related to motility and chemotaxis.

The *sotB *gene was similar to genes for sugar efflux transporters and multi-drug resistance transporters (COG2814, TIGR00880). SecG forms the machinery for protein translocation across the cytoplasmic membrane [80]. YajC is a member of the preprotein translocase machinery, SecDF-YajC. SecDF-YajC inhibits disulfide bond formation between two SecG molecules [81]. ComH is essential for natural transformation [82]. Its putative N-terminal secretion signal suggests that it is either anchored in the cytoplasmic membrane or exported to the periplasm [82]. The *cvpA *gene of *E. coli *is suggested to encode a membrane protein required for colicin V production/secretion [83].

The *secG *homolog, mHP1255, showed divergence focused around residues 150-160. The nucleotide sequence AAAGAGAAG encoding Lys-Glu-Asn was present once in hpEurope and hspWAfrica strains whereas repeated 2 to 4 times in tandem in all hpEastAsia strains (4 in F16, 3 in Sat464, and 2 in the others).

Positively-selected amino-acid changes of the putative *sotB *product were identified (Table 7). Of these, W186Y lay at the end of a transmembrane helical region away from the substrate tranlocation pores.

#### Motility and chemotaxis

Four genes in Table 6, *fliT*, *fliK*, *maf *and *cheY*, are related to motility and chemotaxis.

The *fliT *product is a flagellar chaperone [84], whereas the *fliK *product controls the hook length of flagella [85]. The *maf *gene encodes a member of motility accessory family of flagellin-associated proteins implicated in flagellar assembly [86]. The *cheY *gene (HP1067) encodes a response regulator of a two-component signal transduction system regulating chemotaxis [84]. CheY does not act as a transcriptional activator. Instead, when activated, it interacts directly with the flagellar motor-switch complex, causing a clockwise rotation of the flagella that results in cell tumbling.

Intra-hspEAsia divergence was very small for *cheY *(Table 6 and Figure 8C (a)). It would be interesting to see whether this divergence is related to differences in chemotaxis.

#### Electron transfer

Seven genes in Table 6, *fixQ*, *fixS*, *frxA*, *hypD*, *hydE*, *pgl *and *nuoF*, are related to electron transfer.

Aerobic respiration in *H. pylori *has been analyzed experimentally and by genome sequences. A cb-type cytochrome c oxidase is the sole terminal oxidase present in *H. pylori *[87]. FixQ (= CcoQ) is a component of the oxidase. The *fixS *gene likely encodes the cation transport subunit of the oxidase [34]. It has been proposed that FixS plays a role in the uptake and metabolism of copper required for oxidase assembly [87]. Aerobic respiration results in production of toxic superoxide at this terminal oxidase, which is involved in bacterial death [88]. The *frxA *gene, NAD(P)H-flavin oxidoreductase, is involved in redox of flavins, which are important electron transfer mediators [89]. Reduced flavins reduce ferric complexes or iron proteins with low redox potential. FrxA is one of the enzymes that make *H. pylori *sensitive to metronidazole [90]. *H. pylori *is capable of hydrogen oxidation [87]. HypD is involved in maturation of the [NiFe] H_2_-uptake hydrogenase, and catalyzes insertion and cyanation of the iron center [91]. The *hydE *gene is also necessary for the hydrogenase activity [92]. The *pgl *gene (HP1102) encodes a 6-phosphogluconolactonase, which catalyzes the second step of the phosphopentose pathway. This phase of the phosphopentose pathway generates reducing power in the form of NADPH and is important in other organisms in defense against reactive oxygen species and oxidative stress response [93,94].

Intra-hspEAsia divergence was very small for *fixQ *(Figure 8C (b), Table 5 and Table 6).

#### Translation

Four genes in Table 6, *miaA*, *tilS*, *def*, and *prmA*, are important for translation.

MiaA and TilS affects translation fidelity [95-97]. MiaA isopentenyl-tRNA transferase modifies the tRNAs that read codons starting with U to minimize peptidyl-tRNA slippage in translation. TilS, the tRNA(Ile2) lysidine synthetase, modifies cytidine to lysidine (2-lysyl-cytidine) at the first anticodon of tRNA(Ile2), thereby switching tRNA(Ile2) from a methionine-specific to an isoleucine-specific tRNA. Def removes a formyl group from the N-terminus of a nascent polypeptide and is a potential drug target [98]. PrmA is a trimethyltransferase that methyates multiple residues in the N-terminal domain of ribosomal protein L11, a universally conserved component of the large ribosomal subunit [99].

There was evidence that divergence in *miaA *was adaptive (Table 7), and the relevant amino acid residue was mapped on the structure (Figure 9B ii), as described above. Intra-hspEAsia divergence was not large for *def *(located in zone 2), whereas large for *miaA *(in zone 3).

#### Nucleases

Four genes in Table 6, *addA*, *rnhA*, *rnhB *and *hsdR*, are nucleases.

AddA (AdnA, PcrA) is a RecB-like helicase that promotes DNA recombination repair and survival during colonization [100]. Upon encounter with a DNA double-strand break, *E. coli *RecBCD enzyme degrades non-self DNA, but repairs self DNA marked by a genomic identification sequence through RecA-mediated homologous recombination. The identification sequence varies among bacterial groups [101] and can be altered by a mutation in RecBCD [102].

The *rnhA *and *rnhB *genes encode RNase HI and RNase HII, which hydrolyze RNA hybridized to DNA. Their biological role remains unclear, although they affect DNA replication, repair and transcription [103,104].

An AT-rich region of the *addA *gene linking the helicase domain and the nuclease domain showed an interesting divergence: the sequence AAAGAAAG(T/C)AAA encoding Lys-Glu-Ser-Lys was repeated in tandem 2 to 8 times in the hspWAfrica and hpEurope strains but was absent or present only once in the hspEAsia strains. The hspAmerind strains have a single copy (4 strains) or two copies (1 strain).

#### Cell division

Gene *ftsA *encodes an actin-like, membrane-associated protein that interacts with the tubulin-like FtsZ protein, helps it assemble into the Z ring, anchors it to the cytoplasmic membrane, and recruits other proteins for cell division [105]. It is a potential drug target [106].

#### Amino acid

The *ilvE *gene (HP1468) encodes a branched-chain amino acid aminotransferase that generates glutamic acid from branched-chain amino acids (valine, leucine, isoleucine) that are essential to *H. pylori*. We do not know whether its divergence is related to loss of jhp0585, encoding a branched-amino-acid dehydrogenase, in all hpEastAsia strains (see above), or whether it is related to a possible geographical divergence in the amino acid content of food.

## Discussion

We closely compared complete genome sequences through phylogenetic profiling, phylogenetic tree construction, and nucleotide sequence analysis. The results distinguished decaying from intact genes and revealed drastic evolutionary changes within the *H. pylori *species. Our results clearly define the *H. pylori *East Asian lineage as distinct at the genome level from the African, European or Amerind lineages (Table 2). The East Asian lineage consists of Japanese and Korean genomes and corresponds to hspEAsia in the phylogenetic tree of the concatenated seven genes used for multi-locus sequence typing. The hspEAsia and hspAmerind lineages form a phylogenetic group hpEastAsia. The outstanding differences are in proteins related to: (i) host-interaction; (ii) electron transfer and redox metabolism; and (iii) translation fidelity.

### Host-interaction proteins

Many of the virulence factors show wide divergence between hspEAsia and hpEurope, most likely because of co-evolution with the host. We anticipate that the list of well-diverged genes (Table 6) is enriched for host-interaction and potential virulence genes. We detected positively-selected amino-acid changes in two virulence factors: *cagA *and *vacA *(Table 7).

Many OMP families showed loss of one of their resident loci (*hopMN*, *babABC*, *sabAB*), whereas one family (*oipA*) showed duplication of its locus. Some OMP genes showed internal deletions (*vacA-2*) or interallelic homologous recombination (*hopMN*). A group-specific repertoire was seen for other OMP genes (*homB*, *hopZ *and *hopQ*), for other criteria. We also found substantial hspEAsia-hpEurope divergence in many OMPs (Table 5). The OMPs play important roles in host interaction such as adhesion to the host cells and induction of immune responses [26]. For example, OipA induces IL-8 from host cells [70]. Systematic decay of OMP genes occurred during adaptation of *H. pylori *to a new host of large felines, generating the new species of *H. acinonychis *[36]. Hence, the above OMP changes might reflect selection and/or fine regulation in host interaction, and more specifically, may help avoid the host immune system. At least two OMPs show evidence for positive selection (Table 7). We do not yet know whether these OMP changes are related to immune response or adhesin activity.

Lewis antigen mimicry is important for gastric colonization and adhesion. The mimicry affects innate immune recognition, inflammatory response, and T-cell polarization. Long-term infection by *H. pylori *might induce autoreactive anti-Lewis antigen antibodies [107]. Divergence in transferase genes for LPS biosynthesis may have resulted from co-evolution with the host immune system and could be related to changes in Lewis antigens in human populations. For example, the Le(a+b+) phenotype is almost absent in Caucasian persons whereas it occurs with a higher frequency in the Asian population [108]. This might be related to differences in pathogenicity and adaptation [109].

Changes in transporter genes, the loss of a putative amino acid utilization gene, divergence in a branched chain amino acid metabolism gene, differences in acetate metabolism genes, and divergence in motility and chemotaxis genes could also be related to host interaction, because these are related to the stomach environment. An interesting question is if these changes are related to variation in human diets.

### Electron transfer

Several key electron transfer components were diverged between hspEAsia and hpEurope. The multiple and drastic changes in redox metabolism were unexpected. The systematic decay of all Mo-related genes through mutations in all (6/6) hspEAsia strains was the most striking. We do not know whether our findings reflect the biased environmental occurrence of Mo or the dietary habits of human populations. The richest sources of Mo include legumes, cereal grains (and baked products), leafy vegetables, milk, beans, liver, and kidney, whereas fruits, stem and root vegetables, and muscle meats are poor Mo sources [110].

The BisC homolog, the only molybdoenzyme found in the *H. pylori *genome, is similar to a number of periplasmic reductases for alternative oxidants such as dimethylsulfoxide or trimethylamine *N*-oxide [87]. Western strains of *H. pylori *might be able to use *N*- and/or *S*-oxide as an electron acceptor in energy metabolism in addition to oxygen and fumarate. One hypothesis about decay of the Mo-related genes is that this anaerobic electron transport system became maladaptive in the East Asian lineage. One possibility is the radical reaction mediated by MoaA in molybdopterin synthesis is dangerous in the presence of oxygen. This could explain the observed changes in oxidative phosphorylation and acetate metabolism.

A candidate for the BisC substrate is an oxidized form of methionine, free or within a protein. Methionine is sensitive to oxidation, which converts it to a racemic mixture of methionine-*S*-sulfoxide (Met-*S*-SO) and methionine-*R*-sulfoxide (Met-*R*-SO) [111]. The reductive repair of oxidized methionine residues performed by methionine sulfoxide reductase is important in many pathogenic bacteria in general, and specifically for *H. pylori *to maintain persistent stomach colonization [112,113]. *H. pylori *methionine sulfoxide reductase (Msr, HP0224 product) is induced under oxidative stress control and can repair methionine-*R*-sulfoxide but not the *S *isomer, even though it is a fusion of an *R*-specific and an *S*-specific enzyme [114]. BisC from other bacteria can reduce and repair the *S *but not the *R *form [111].

If the sole function of BisC is to repair methionine-*S*-sulfoxide, another means to repair methionine-*S*-sulfoxide may have appeared in the East Asian *H. pylori*, for example by higher expression of Msr. In this case, BisC may have been inactivated because Mo-related reactions were no longer necessary. The substitution by a DNA element downstream of the *msr *gene in the hspEAsia strains (5/6, all but strain 52) could be involved in the hypothesized methionine-*S*-sulfoxide repair activity of its product.

Another possibility is decrease of oxidative stress generating methionine-*S*-sulfoxide in the East Asian *H. pylori*. Oxidative stress is induced by acid exposure, and *msr *is among the oxidative stress genes induced by acid [115]. *H. pylori *infection has different effects on acid secretion in Europe and Asia [116]. In Europe, antral-predominant gastritis with increased acid secretion is frequent, whereas in Asia, pan-gastritis and subsequent atrophic gastritis with decreased acid secretion are common. The decrease in acid experienced by East Asian *H. pylori *lineages may have decreased their methionine-*S*-sulfoxide and made its repair by BisC unnecessary.

Downregulation of some of the Mo-related genes in a European strain under acidic conditions may be related to their decay [30]. Downregulation may occur to avoid the possible toxic effects of Mo metabolism under conditions of acid adaptation.

Taken together, our results led us to predict that the East Asian *H. pylori *strains are different from the European strains in electron transfer reactions and responses to oxygen and acid. Possibly related to this alteration in redox is the presence of the two acetate-related pathways in 3 out of 4 Japanese strains. These are expected to be able to switch from acetate fermentation to acetate utilization under aerobic conditions, as seen for *E. coli *[117]. The European strains, some of the hspAmerind strains, and the other hspEAsia strains may be regarded as mutants that lack the *pta-ackA *pathway and the supposedly important acetyl~P signal. Global effects of these defects on chemotaxis, nitrogen and phosphate assimilation, osmo-regulation, flagellar biogenesis, biofilm development, and pathogenicity are expected, based on the various phenotypes of *E. coli *strains defective in these genes [33].

### Translation fidelity

Translational proteins also diverged between hpEurope and hspEAsia strains. MiaA (tRNA delta(2)-isopentenylpyrophosphate transferase) and TilS (tRNA lysidine synthetase) affect accuracy in elongation. The amino-acid change in MiaA turned out to be adaptive (Table 7). TilS affects translation efficiency at various stages. Ambiguity in translation is proposed to be important in the evolution of novel proteins by generating phenotypic and genetic diversity in the proteome for selection [118]. This role of ambiguity is similar to the evolutionary role of genome-wide modulation of mutation rates by genes such as *mutS *[119].

### Implications for medicine

East Asian (Japanese/Korean) *H. pylori *appear to be quite different from European *H. pylori*. Our results provide a solid starting point for understanding the biology, host interaction, and pathogenesis of the East Asian *H. pylori*, which in most previous works were inferred from a European strain. Divergences included virulence, cell surface-related, and drug target genes. These results will affect our strategy in developing effective therapies and drugs. Questions raised by our findings include whether East Asian VacA (Figure 9B) interacts with host cells in the same way as European VacA.

The diverged gene *frxA *is associated with resistance to antibiotics metronidazole [120], which is frequently used in *H. pylori *eradication. The divergence in the *frxA *could affect resistance to this group of drugs in various ways. More generally, if redox metabolism differs between hspEAsia and hpEurope strains, the same drug might produce different effects, depending on intra-bacterial redox reactions.

The diverged genes included two potential drug targets (*def *and *ftsA*), so drugs that target these proteins may have different effects in East Asian and European strains. We do not know, for example, whether anti-*H. pylori *drugs designed from structure of European Def [98] will be as effective against East Asian *H. pylori*.

### Remaining questions

Clearly, many studies are needed to answer these and other questions raised by the genomics results presented here. Phylogenetic analysis in the present study used OGs where genes of hspEAsia were clustered separately from those of hpEurope. Some genes do not share this topology, as suggested above for *acoE *deletion and *hopMN *recombination. We plan to study the distortion in the tree. We focused on differences between a limited numbers of strains from each group. However, there are variations within East Asian strains (Table 5). Further experimental examination of the divergence within hspEAsia, and between hspEAsia and the other strains are necessary to understand their divergence in detail. Such examination might reveal complexity in evolution and will be the subject of a separate study. The mechanisms underlying the variation, such as mutations and rearrangements, will be a subject of a separate study [25].

## Conclusions

Taking advantage of the extreme genome plasticity of *H. pylori*, we demonstrated how drastically a genome can change during evolution within a species. Our results revealed drastic changes in proteins for host interaction and electron transfer and suggested their importance in adaptive evolution. These results define the *H. pylori *East Asian and Western lineages at the genome level, enhance our understanding of their host interaction, and contribute to the design of effective drugs and therapies. The approach of fine comparative analysis of closely-related multiple genomes may reveal subtle but important evolutionary changes in other populations.

## Methods

### *H. pylori *culture

Four strains were isolated from patients with diffuse type gastric cancer, intestinal type gastric cancer, duodenal ulcer, and gastritis (F57 [121], F32, F30 and F16 [122]). The ABO blood groups of the hosts were: F57, B; F32, A; F30, O; F16, B. Studies were performed according to the principles of the Declaration of Helsinki, and consent obtained from each individual after a full description of the nature and protocol of the study.

Gastric biopsy specimens from each patient were inoculated onto a trypticase soy agar (TSA)-II/5% sheep blood plate and cultured under microaerobic conditions (O_2_, 5%; CO_2_, 15%; N_2_, 80%) at 37°C for 5 days. A single colony was picked from each primary culture plate, inoculated onto a fresh TSA-II plate, and cultured under the conditions described above. A few colonies were picked from each plate and transferred into 20 ml of Brucella broth liquid culture medium containing 10% fetal calf serum, and cultured for 3 days under the conditions as described above. A part of the liquid culture sample was stored at -80°C in 0.01 M phosphate-buffered saline (PBS) containing 20% glycerol. DNA from each *H. pylori *isolate was extracted from the culture pellet by the protease/phenol-chloroform method, suspended in 300 μl of TE buffer (10 mM Tris HCl, 1 mM EDTA) and stored at 4°C for PCR analysis and nucleotide sequencing.

### Genome sequencing

The genome sequences of *H. pylori *strains F16, F30, F32 and F57 were determined by a whole-genome shotgun strategy. We constructed small-insert (2 kb) and large-insert (10 kb) plasmid libraries from genomic DNA, and sequenced both ends of the clones to obtain 26,112 (F16 and F57), 30,720 (F30) and 33,792 (F32) sequences using ABI 3730xl sequencers (Applied Biosystems), with coverage of 10.0 (F16)-, 11.5 (F30)-, 12.7 (F32)- and 10.0 (F57)-fold. Sequence reads were assembled with the Phred-Phrap-Consed program, and gaps were closed by direct sequencing of clones that spanned the gaps or with PCR products amplified using oligonucleotide primers designed against the ends of neighboring contigs. The overall accuracy of the finished sequence was estimated to have an error rate of less than 1 per 10,000 bases (Phrap score of ≥40). Sequences of the molybdenum-related genes and the genes in the acetate pathway of the four Japanese strains were verified by resequencing PCR fragments directly amplified from genomic DNA (primers are in Additional file 4 (= Table S3)). The genome sequences of other strains were obtained from National Center for Biotechnology Information (NCBI) [123]. Accession numbers are in Table 1.

### Gene finding and annotation

We used the same protocol to identify genes in the four new strains and 16 other complete genomes (Table 1; gene assignment differences are in Additional file 8 (= Table 6)).

Protein-coding genes were identified by integrating predictions from programs GeneMarkS [124] and GLIMMER3 [125]. All ORFs longer than 10 amino acids were searched using BLASTP [126] against two databases, one composed of genes of 6 *H. pylori *genomes in RefSeq database at NCBI ("close" database), and the other composed of genes of 300 complete prokaryote genomes (one genome per one genus) available at the end of 2008, except for those in the *Helicobacter *genus ("distant" database). When the predicted start position differed in GeneMarkS and GLIMMER3, assignments were made by consensus of hits, with consensus against the "distant" database taking priority over the "close" one. The consensus start position among bidirectional best hits with 50% or more amino acid sequence identity for each matched region for each genome pair was determined by majority rule. Overlap of genes was resolved by comparing the results from four prediction programs. Genes encoding fewer than 100 amino acids and predicted only by Glimmer3 were dropped except for the microcin gene.

tRNA genes were detected using tRNAscan-SE [127]. rRNA genes were identified based on sequence conservation. Putative replication origins were predicted by GC-skew (window size 500 bp, window shift 250 bp).

### Core genome analysis

The common core structure conserved among 20 *H. pylori *genomes was identified based on conservation of gene order among orthologs using the CoreAligner program [23] implemented in the RECOG system. Briefly, CoreAligner identifies the genomic core of the input genomes by taking the longest path of the neighborhood graph that consists of conserved neighborhood gene pairs, which are defined as pairs of OGs that are within a neighborhood of 20 genes in at least half of the genomes. For this analysis, we used as input a set of OGs generated by the DomClust program [24] (see "Phylogenetic profile analysis" section below for details about identification of OGs by DomClust). Absence of a gene in some genomes (at least half of the genomes) in each OGs among the core is allowed. In addition, as identified OGs are at the domain level, if a counterpart of a gene in one genome is split in another genome, different number of genes can participate in the OGs in different genomes. Thus, the number of core genes in each genome can vary. Still, the numbers of core genes varied less (1364-1424; SD = 13.5) than the total number of genes among the strains (1465-1593; SD = 33.9) (Table 1). Among those core OGs, 1079 OGs were universally conserved (conserved in the all genomes), non-domain-separated, with one-to-one correspondence, and designated "well-defined core OGs". Those 1079 OGs were used for phylogenetic analysis (Figure 1). Nucleotide sequences of genes in well-defined core OGs were aligned by the Mafft program [128], from which conserved blocks were extracted by the Gblocks program [129].

### Phylogenetic profile analysis

Phylogenetic profiling was carried out using the set of OGs generated by DomClust [24]. We identified OGs with East Asian-specific features as those whose phylogenetic profiles were highly correlated to the template pattern (taking 1 for hspEAsia and 0 for hpEurope). The DomClust clustering program can identify OGs at the domain level, and was used to identify genes truncated in particular strains. Clustering was performed based on PAM (point accepted mutation) distance rather than score to ensure proper evaluation of evolutionary distances, even if one gene was truncated; in the latter case, scores may underestimate evolutionary relatedness. To clarify differences in gene-splitting patterns among strains, we did not use DomClust options to suppress domain splitting.

To identify genes with characteristic patterns of hspEAsia strains, we constructed a phylogenetic profile for each OG as a vector of examined property values (*e.g*., number of domains or number of duplications). For surveying patterns of gene splitting and deletion, a phylogenetic profile was constructed for each OG using the number of domains for each gene that resulted from the clustering. For surveying patterns of gene duplication, a phylogenetic profile was constructed using the number of duplicated genes (in-paralogs). To find OGs with a characteristic hspEAsia pattern, equality of the medians among different populations was tested by Kruskal-Wallis test. Tests between East Asian and European strains used the six hspEAsia strains and the seven hpEurope strains. Tests among four subpopulations used six hspEAsia, five hspAmerind, seven hpEurope, and two hspWAfrica strains.

Phylogenetic network analysis of the *hopM/N *family was carried out using NeighborNet [130] implemented on SpritsTree [131].

### Analyses of molybdenum-related genes

*H. pylori *protein sequences were searched against the CDD conserved protein domain database, by RPS-BLAST [132]. Protein families extracted from the search results for Mo-cofactor synthesis or binding domain were: PF03404 (Mo-co_dimer), PF03205 (MobB), PF02738 (Ald_Xan_dh_C2), PF01568 (Molydop_binding), PF02730 (AFOR_N), PF02597 (ThiS), PF03454 (MoeA_C), PF06463 (Mob_synth_C), PF03453 (MoeA_N), PF01315 (Ald_Xan_dh_C), PF01493 (GXGXG), PF02579 (Nitro_FeMo-Co, PF01967 (MoaC), PF03459 (TOBE), PF02391 (MoaE), PF00384 (Molybdopterin), PF04879 (Molybdop_Fe4S4), PF02665 (Nitrate_red_gam), PF00174 (Oxidored_molyb), PF00994 (MoCF_biosynth), PF03473 (MOSC), PF02625 (XdhC_CoxI), PF01314 (AFOR_C), PF01547 (SBP_bac_1) (pfam name in parentheses). Homologs of two molybdoproteins [133] that were not detected in the above protein families were absent in the *H. pylori *genomes.

*bisC *was the only molybdoenzyme gene in the 20 *H. pylori *genomes with detected domains PF01568 (Molydop_binding) and PF00384 (Molybdopterin). A multidomain TIGR00509 (bisC_fam) was also detected in *bisC*.

### Analyses of horizontally transferred regions

GIs were detected by searching for regions that fulfilled the conditions of: (i) longer than 5 kb; (ii) continuous ORFs not perfectly conserved in all 20 *H. pylori *strains; and (iii) whole regions assumed as extrinsic by Alien Hunter [134]. Counterparts of detected GIs in Amerind strains were previously reported as TnPZ [48,49].

### Genes with a large distance between East Asian and European strains

OGs diverged between six hspEAsia and seven hpEurope strains were screened based on two values related to their phylogenetic tree. The *d*_*a *_value was the distance between the last common ancestral (LCA) node of hspEAsia and the LCA node of hpEurope. The *d*_*b *_value was the average distance of hspEAsia from its LCA node. OGs with hspEAsia-diverged genes were screened by introducing the following conditions (with hspAmerind omitted): (i) OGs in which all the hspEAsia genes of the OG formed a sub tree without any hpEurope genes in the phylogenetic tree; (ii) OGs universally conserved (not less than 12 of the 13 genomes; not less than 10 among 11 genomes for comparison of 6 hspEAsia and 5 hpEurope strains in Additional file 7 (= Table S5)); (iii) genes with no domain fusion/fission event among the 13 genomes (within ± 20% of the mean length of the OG, measured in amino acid residues); (iv) *d*_*a *_value greater than twice the *d*_*a *_value of the concatenated well-defined core tree (of amino-acid sequences) (denoted as *d*_a_*; with the resulting cutoff of *d*_*a *_> 0.02324; 1079 OGs; see "core genome analysis" section above). Among 1248 OGs that satisfied the criteria (ii) and (iii), 692 OGs satisfied the criteria (i), that is, complete separation of genes of hspEAsia from those of hpEurope. The *d*_b_* ± sd values in logarithmic scale, corresponding to 0.00550 and 0.0231 (*d*_b_* = 0.01128) in the original scale, were used as threshold values for the three zones (N = 687; five OGs with *d*_*b *_= 0 were excluded from 692 OGs satisfying the above criteria (i)-(iii)).

Amino acid sequences of the genes were aligned by the einsi command of the MAFFT program [128], from which a neighbor-joining tree was constructed by the ClustalW program [135].

A branch-site likelihood ratio test of positive selection was carried out using PAML [60] based on the multiple alignment by the einsi command of MAFFT [128]. Only residues aligned at the same site by the einsi command and by PRANK (with codon option) [136] were considered. Positively-selected residues were mapped on the p55 structure of VacA using PyMol).

### Statistics

The equality of means for phylogenetic profiling between East Asian and European strains was tested by Kruskal-Wallis one-way analysis of variance by ranks, a non-parametric method for testing equality of population medians among groups. The tests were conducted using the R statistics package [137].

### Accession Numbers

The accession numbers of the *H. pylori *genome sequences reported in this paper are: F16 [DDBJ:AP011940.1 http://getentry.ddbj.nig.ac.jp/cgi-bin/get_entry2.pl?database=ver_ddbj&query=AP011940.1 ], F30 [DDBJ:AP011941.1 http://getentry.ddbj.nig.ac.jp/cgi-bin/get_entry2.pl?database=ver_ddbj&query=AP011941.1, DDBJ:AP011942.1 http://getentry.ddbj.nig.ac.jp/cgi-bin/get_entry2.pl?database=ver_ddbj&query=AP011942.1], F32 [DDBJ:AP011943.1 http://getentry.ddbj.nig.ac.jp/cgi-bin/get_entry2.pl?database=ver_ddbj&query=AP011943.1, DDBJ:AP011944.1 http://getentry.ddbj.nig.ac.jp/cgi-bin/get_entry2.pl?database=ver_ddbj&query= AP011944.1] and F57 [DDBJ:AP011945.1 http://getentry.ddbj.nig.ac.jp/cgi-bin/get_entry2.pl?database=ver_ddbj&query=AP011945.1].

## List of Abbreviations

bp: base pair(s); hpEastAsia: hpEurope and hpAfrica1, populations of *H. pylori*; hspEAsia and hspAmerind: sub-populations of hpEastAsia; hspWAfrica: a sub-population of hpAfrica1; GI: genomic island; Mo: molybdenum; OG: orthologous group; OMP: outer membrane protein; ORF: open reading frame; redox: reduction-oxidation.

## Competing interests

The authors declare that they have no competing interests.

## Authors' contributions

MK and YF contributed to informatics analysis and wrote the manuscript. YF carried out experimental verification of sequences of molybdenum-related genes and acetate pathway related genes. KY, TT, and IU contributed to informatics analysis. NH and NT contributed to genome DNA preparation. KO and MH contributed to sequencing and assembly. MY and TA provided the strains. I.K. contributed to design, analysis and writing. All the authors discussed the results and commented on the manuscript. All the authors read and approved the final manuscript.

## Author information

Current position of MK: Institute of Biogeosciences, Japan Agency for Marine-Earth Science and Technology, Yokosuka, Kanagawa, 237-0061, Japan

## Supplementary Material

Additional file 1 Phylogenetic tree of *H. pylori *based on MLST genes Click here for file

Additional file 2 **Genes characterizing East Asian strains: domain-based analysis**. Click here for file

Additional file 3 **Mutations in molybdenum-related genes of *H. pylori***. Click here for file

Additional file 4 **Primers for sequence validation**. Click here for file

Additional file 5 **Distance values of 692 genes with complete separation of hspEAsia and hpEurope**. Click here for file

Additional file 6 **Multiple sequence alignments of diverged genes**. Click here for file

Additional file 7 **Examination of robustness of extraction of diverged genes**. Click here for file

Additional file 8 **Differences in gene assignment**. Click here for file
