# Supplementary material for: Evolution in an oncogenic bacterial species with extreme genome plasticity: Helicobacter pylori East Asian genomes
Source: BMC Microbiol. 2011 May 16;11:104. doi: 10.1186/1471-2180-11-104 (PMC3120642; doi:10.1186/1471-2180-11-104)
Supplement: Additional file 1 — Phylogenetic tree of H. pylori based on MLST genes [file 1471-2180-11-104-S1.PDF]

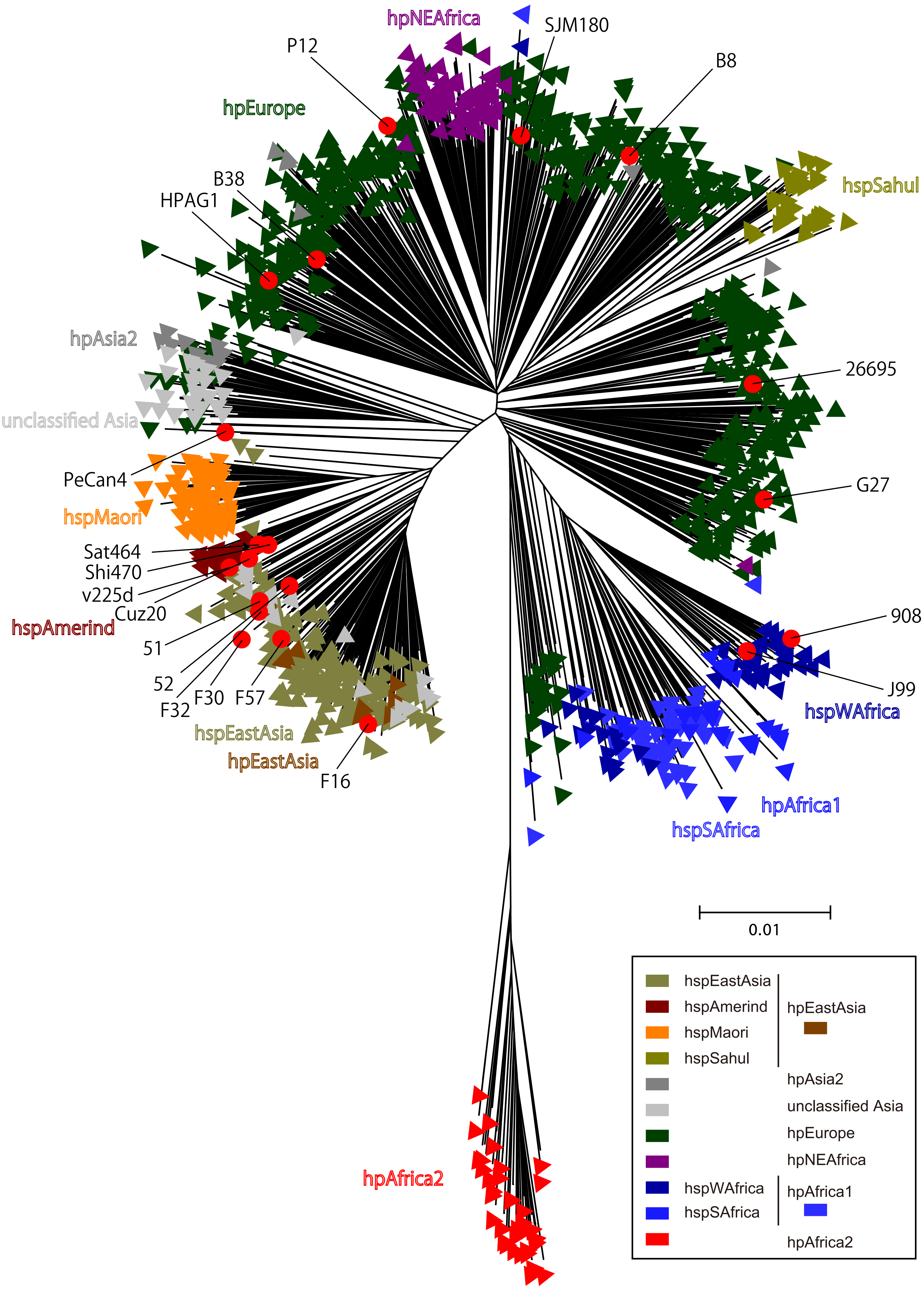
hpNEAfrica
SJM180
P12
B8
hpEurope
hspSahul
B38
HPAG1
hpAsia2
unclassified Asia
26695
PeCan4
hspMaori
G27
Sat464
Shi470
v225d
Cuz20
hspAmerind
908
51
J99
52
F32
F30
F57
hspWAfrica
hspEastAsia
hpEastAsia
F16
hpAfrica1
hspSAfrica
0.01
hpAfrica2
hspEastAsia
hspAmerind
hspMaori
hspSahul
hpEastAsia
hpAsia2
unclassified Asia
hpEurope
hpNEAfrica
hspWAfrica
hspSAfrica
hpAfrica1
hpAfrica2

Figure S1. Phylogenetic tree of *H. pylori* based on MLST genes

The tree was constructed using the neighbor-joining method from the concatenated alignment of fragments of seven genes used in the *H. pylori* MLST database (*atpA, efp, mutY, ppa, trpC, ureI* and *yphC*) [1]. The 20 strains compared are indicated. The color for the labels of typing is based on [2].

1. Adams DW, Errington J: **Bacterial cell division: assembly, maintenance and disassembly of the Z ring.** *Nat Rev Microbiol* 2009, **7:**642-653.
2. Linz B, Balloux F, Moodley Y, Manica A, Liu H, Roumagnac P, Falush D, Stamer C, Prugnolle F, van der Merwe SW, et al: **An African origin for the intimate association between humans and *Helicobacter pylori*.** *Nature* 2007, **445:**915-918.
