# Supplementary material for: Evolution in an oncogenic bacterial species with extreme genome plasticity: Helicobacter pylori East Asian genomes
Source: BMC Microbiol. 2011 May 16;11:104. doi: 10.1186/1471-2180-11-104 (PMC3120642; doi:10.1186/1471-2180-11-104)
Supplement: Additional file 6 — Multiple sequence alignments of diverged genes. [file 1471-2180-11-104-S6.ZIP › Diverged_genes_multiple_seuence_alignments/HP0097.mfa.rtf]

                  1         11        21        31        41        51        61        71        81        91                          |         |         |         |         |         |         |         |         |         |         HB8:HPB8_1469     MKKIVLVAIALLISGCASYKITPEHVTSYNNGIQVMTSTQAKSKVQLEIAQSKLKGLNESPLVLYVAAQVIEGNPVVFSRKAISVSINQTNLPVLSLRQVHB38:HELPY_0094   MKKIVLVAIALLISGCASYKITPEHVTSYNNGIQVMTSTQAKSKVQLEIAQSKLKGLNESPLVLYVAAQVIEGNPVVFSRKAISVSINQTNLPVLSLRQVHSJM:HPSJM_00550  MKKIVLVAVALLMSACASYKITPEHVTSYNNGIQVMTSTQAKSKVQLEIAQSKLKGLNESPLVLYVAAQVIEGNPVVFSRKAISVSINQTNLPVLSLRQVHP12:HPP12_0099   MKKIVLVAIALLMSACASYKITPEHVTSYNNGIQVMTSTQAKSKVQLEIAQSKLKGLNESPLVLYVAAQVIEGNPVVFSRKAISVSINQTNLPVLSLRQVH266:HP0097       MKKIVLVAIALLMSACASYKITPEHVTSYNNGIQVMTSTQAKSKVQLEIAQSKLKGLNESPLVLYVAAQVIEGSPVVFSRKAISVSINQTNLPVLSLRQVHHPA:HPAG1_0097   MKKIVLVAIALLMSACASYKITPEHVTSYNNGIQVMTSTQAKSKVQLEIAQSKLKGLNESPLVLYVAAQVIEGNPVVFSRKAISVSINQTNLPVLSLRQVHG27:mHPG27_89    MKKIVLVAIALLMSACASYKITPEHVTSYNNGIQVMTSTQAKSKVQLEIAQSKLKGLNESPLVLYVAAQVLEGNPVVFSRKAISVSINQTNLPVLSLRQVHF32:HPF32_0109   MKKIILVAIALLMSACASYKITPEHVTSYNNGIQVMTSTQAKSKVQLEIAQSKLKGLSASPLVLYVAAQVLEGNPVAFGRKAISVSINQTNLPVLSLRQVHF16:HPF16_0110   MKRIVLVAIALLMSACASYKITPEHVTSYNNGIQVMTSTQAKSKVQLEIAQSKLKGLSESPLVLYVAVQVLEGNPVAFGRKAISVSINQTNLPVLSLRQVHF30:HPF30_1177   MKRIVLVAIALLMSACASYKITPEHVASYNNGIQVMTSTQAKSKVQLEIAQSKLKGLNASPLVLYVAVQVLEGNPVVFGRKAISVSINQTNLPVLSLRQVHF57:HPF57_0138   MKRIVLVAIALLMSACASYKITPEHVTSYNNGIQVMTSTQAKSKVQLEIAQSKLKGLSESPLVLYVAAQVLEGNPVMFGRKAISVSINQTNLPVLSLRQVH51:KHP_0100      MKRIVLVAIALLMSACASYKITPEHVTSYNNGIQVMTSTQAKSKVQLEIAQSKLKGLSESPLVLYVAAQVLEGNPVAFGRKAISVSINQTNLPVLSLRQVH52:HPKB_0110     MKKIVLVAIALLMSACASYKITPEHVTSYNNGIQVMTSTQAKSKVQLEIAQSKLKGLSESPLVLYVAAQVLEGNPVAFGRKAISVSINQTNLPVLSLRQV                  101       111       121       131       141       151       161       171       181       191                         |         |         |         |         |         |         |         |         |         |         HB8:HPB8_1469     MKSSFDFEGILQSFNISVPTAPIDNINMITPPMFYYGQGSFLAYNGMMYGGMGMYGPGFGMMMMDDVEEQEIMQESRQALKILAINYLKNNTLNVEGKARHB38:HELPY_0094   MKSSFDFEGILQSFNISVPTAPIDNINMITPPMFYYGQGSFLAYNGMMYGGMGMYGPGFGMMMMDDVEEQEVMQESRQALKILAINYLKNNTLNVEGKARHSJM:HPSJM_00550  MKSSFDFEGILQSFNISVPTAPIDNVNMITPPMFYYGQGSFLAYNGMMYGGMGMYGPGFGMMMMDDVEEQEVMQESRQALKILAINYLKNNTLNVEGKARHP12:HPP12_0099   MKSSFDFEGILQSFNISVPTAPIDNINMITPPMFYYGQGSFLAYNGMMYGGMGMYGPGFGMMMMDDVEEQEVMQESRQALKILAINYLKNNTLNVEGKARH266:HP0097       MKSSFDFEGILQSFNIAVPTTPIDNVNMITPPMFYYGQGGFLAYNGMMYGGMGMYGPGFGMMMMDDVEEQEVMQESRQALKILAINYLKNNTLNVESKAKHHPA:HPAG1_0097   MKSSFDFEGILQSFNIAVPTTPIDNVNMITPPMFYYGQGGFLAYNGMMYGGMGMYGPGFGMMMMDDVEEQEVMQESRQALKILAINYLKNNTLNVENKAKHG27:mHPG27_89    MKSSFDFEGILQSFNIAVPTTPIDNVNMITPPMFYYGQGSFLAYNGMMYGGMGMYGPGFGMMMMDDVEEQEVMQESRQALKILAINYLKNNTLNVENKAKHF32:HPF32_0109   MKSSFDFEGILQSFNIAVPTTPIDNVNMITPPMFYYGQGGFLAYD-MMYGGMGLYGPGFGMMMMDDVEEQEIMQESRQALKILAINYLKKNTLNVENKAKHF16:HPF16_0110   MKSSFDFEGILQSFNIAVPTTPIDNVNMITPPMFYYGQGGFLAYD-MMYGGMGLYGPGFGMMMVDDVEEQEIMQESRQALKILAINYLKKNTLNVEGKAKHF30:HPF30_1177   MKSSFDFEGILQSFNIAVPTTPIDNVNMITPPMFYYGQGGFLAYD-MMYGGMGLYGPGFGMMMVDDVEEQEIMQESRQALKILAINYLKKNTLNVEGKAKHF57:HPF57_0138   MKSSFDFEGILQSFNIAVPTTPIDNVNMITPPMFYYGQGGFLAYNGMMYGGMGLYGPGFGMMMVDDVEEQEIMQESRQALKILAINYLKKNTLNVENKAKH51:KHP_0100      MKSSFDFEGILQSFNIAVPTTPIDNVNMITPPMFYYGQGGFLAYNGIMYGGMGLYGPGFGMMMVDDVEEQEIMQESRQALKILAINYLKKNTLNVEGKAKH52:HPKB_0110     MKSSFDFEGILQSFNIAVPTTPIDNVNMITPPMFYYGQGGFLAYD-IMYGGMGLYGPGFGMMMMDDVEEQEVMQESRQALKILAINYLKKNTLNVEGKAK                  201       211       221       231       241                  |         |         |         |         |HB8:HPB8_1469     -----GGFVVVDTKNLKTPGVVVVKVFLEDEIHTFKIDISKMHB38:HELPY_0094   -----GGFVVVDTKNLKTPGVVVVKVFLEDEIHTFKIDISKAHSJM:HPSJM_00550  -----GGFVVVDTKNLKTPGVVVVKVFLEDEIHTFKIDISKMHP12:HPP12_0099   -----GGFVVVDTKNLKTPGVVVVKVFLEDEIHTFKIDISKMH266:HP0097       -----GGFVVVDTKNLKTPGVVVVKVFLEDEIHTFKIDISKMHHPA:HPAG1_0097   -----GGFVVVDTKNLKTPGVVVVKVFLEDEIHTFKIDISKMHG27:mHPG27_89    -----GGFVVVDTKNLKTPGVVVVKVFLEDEIHTFKIDISKAHF32:HPF32_0109   -----GGFVVVDTKNLKTPGVVVVKVFLEDEIHTFKMDISKMHF16:HPF16_0110   -----GGFVVVDTKNLKTPGVVVVKVFLEDEIHTFKMDISKMHF30:HPF30_1177   -----GGFVVVDTKNLKTPGVVVVKVFLEDEIHTFKIDISKMHF57:HPF57_0138   -----GGFVVVDTKNLKTPGVVVVKVFLEDEIHTFKIDISKMH51:KHP_0100      -----GGFVVVDTKNLKTPGVVVVKVFLEDEIHTFKIDISKMH52:HPKB_0110     WRVCGSGY-------------------------------QKP
