# Supplementary material for: Evolution in an oncogenic bacterial species with extreme genome plasticity: Helicobacter pylori East Asian genomes
Source: BMC Microbiol. 2011 May 16;11:104. doi: 10.1186/1471-2180-11-104 (PMC3120642; doi:10.1186/1471-2180-11-104)
Supplement: Additional file 6 — Multiple sequence alignments of diverged genes. [file 1471-2180-11-104-S6.ZIP › Diverged_genes_multiple_seuence_alignments/HP0150.mfa.rtf]

                  1         11        21        31        41        51        61        71        81        91                          |         |         |         |         |         |         |         |         |         |         HB8:HPB8_1415     MKEKNFWPLGIMSVLILGLGIVVFLVVFALKNSPKNDLVYFKGHNEVDLNFNAMLKTYENFKSNYRFSVGLKPLIKSPKTPILPYFSKGTHGDKKLQENLHP12:HPP12_0149   MKEKNFWPLGIMSVLILGLGIVVFLVVFALKNSPKNDLVYFKGHNEVDLNFNAMLKTYENFKSNYRFLVGLKPLIKSPKTPILPYFSKGTHGDKKLQENLHB38:HELPY_0154   MKEKNFWPLGIMSVLILGLGIVVFLVVFALKNSPKNDLVYFKGHNEVDLNFNAMLKTYENFKSNYRFLVGLKPLTKSPKTPILPYFSKGTHGDKKLQETLH266:HP0150       MKEKNFWPLGIMSVLILGLGIVVFLVVFALKNSPKNDLVYFKGHNEVDLNFNAMLKTYENFKSNYRFLVGLKPLIKSPKTPILPYFSKGTHGDKKLQENLHHPA:HPAG1_0148   MKEKNFWPLGIMGVLIFGLGIVVFLVVFALKNSPKNDLVYFKGHNEVDLNFNAMLKTYEDFKANYRFLVGLKPLIKSPKTPILPYFSKGTHGDKKLQENLHF32:HPF32_0159   MKEKNFWPLGIMSVLILGLGIVAFLVVFALKNSPKNDLVYFKSHNEVDLNFNAMLKTYENFKSNYRFLVGLKPLTEGSKTPILPYFSKGTHGDKKLQENLHF57:HPF57_0170   MKEKNFWPLGIMSVLILGLGIVVFLVVFALKNSPKNDLVYFKGHNEVDLNFNAMLKTYENFKSNYRFLVGLKPLTEGSKTPILPYFSKGTHGDKKLQENLH52:HPKB_0158     MKEKNFWPLGIMSVLILGLGIVVFLVVFALKNSPKNDLVYFKGHNEVDLNFNAMLKTYENFKSNYRFLVGLKPLTEGSKTPILPYFSKGTHGDKKLQENLH51:KHP_0149      MKEKNFWPLGIMSVLILGLGIVVFLVVFALKNSPKNDLVYFKGHNEVDLNFNAMLKTYENFKSNYRFLVGLKPLTEGSKTPILPYFSKGTHGDKKLQENLHF30:HPF30_1145   MKEKNFWPLGIMSVLILGLGIVMFLVVFALKNSPKNDLVYFKGHNEVDLNFNAMLKTYENFKSNYRFLVGLKPLTEGSKTPILPYFSKGTHGDKKLQENLHF16:HPF16_0159   MKEKNFWPLGIMSVLILGLGIVAFLVVFALKNSPKNDLVYFKSHNEVDLNFNAMLKTYENFKSNYRFLVGLKPLTEGSKTPILPYFSKGTHGDKKLQENLHSJM:HPSJM_00820  MKEKNFWPLGIMGVLIFGLGIVVFLVVFALKNSPKNDLVYFKGHNEVDLNFNAMLKTYENFKSNYRFLVGLKPLTKSPKTPILPYFSKGTHGDKKIQENL                  101       111       121       131       141       151       161       171       181       191                  |         |         |         |         |         |         |         |         |         |HB8:HPB8_1415     LKNALILEKSNTLYVQLQPLKPALDAPNIQVYLAFYPSPSQPRLLGTLDCRSACEPLKFDLLESDKMGRYKILFKFVFKNKEELILEQLAFFK---HP12:HPP12_0149   LKNALILEKSNTLYAQLQPLKPALDPPNIQVYLAFYPSPSQPRWLGVLDCRSACEPLKFDLLESDKMGRYKILFKFVFKNKEELILEQLAFFK---HB38:HELPY_0154   LNNALILEKSNTLYAQLQPLKPALDSPNIQVYLAFYPSPSQPRLLGTLDCRSACEPLKFDLLESDKRGRYKILFKFVFKNKEELILEQLAFFK---H266:HP0150       LNNALILEKSNTLYAQLQPLKPALDSPNIQVYLAFYPSPSQPRWLGTLDCKNACEPLKFDLLESDKMGRYKILFKFVFKNKEELILEQLAFFKQRIHHPA:HPAG1_0148   LNNALILEKSNTLYAQLQPLKPALDPPNIQVYLAFYPSRSQPRWLGVLDCRSACEPLKFDLLESDKMGRYKILFKFVFKNKEELILEQLAFFKQSIHF32:HPF32_0159   LKNALILEKSNTLYVQLQPLKPALDSPNIQVYLAFYPSPSQPRLLGTLDCEIACEPLRFDLLESDKMGRYKILFKFVFKNKEELILEQLAFFK---HF57:HPF57_0170   LKNALILEKSNTLYAQLQPLKPALDSPNIQVYLAFYPSPSQPRLLGTLDCEIACEPLRFDLLESDKMGRYKILFKFVFKNKEELILEQLAFFK---H52:HPKB_0158     LKNALILEKSNTLHAQLQPLKPALDSPNIQVYLAFYPSPSQPRLLGTLDCEIACEPLRFDLLESDKMGRYKILFKFVFKNKEELILEQLAFFK---H51:KHP_0149      LKNALILEKSNTLHAQLQPLKPALDLPNIQVYLAFYPSPSQPRLLGTLDCEIACEPLRFDLLESDKMGRYKILFKFVFKNKEELILEQLAFFK---HF30:HPF30_1145   LKNALILEKSNTLYAQLQPLKPALDSPNIQVYLAFYPSPSQPRLLGTLDCEIACEPLKFDLLESDKMGRYKILFKFVFKNKEELILEQLAFFK---HF16:HPF16_0159   LKNALILEKSNTLYAQLQPLKPALDLPNIQVYLAFYPSPSQPRLLGTLDYEIACEPLKFDLLESDKMGRYKILFKFVFKNKEELILEQLAFFK---HSJM:HPSJM_00820  LNNALILEKSNTLYVRLQPLKPALDSPNIQVYLAFYPSPSQPRLLGTLDCTNACEPLKFDLLEGDKVGRYKILFKFTFKNKEELILEQLAFFK---
