# Supplementary material for: Evolution in an oncogenic bacterial species with extreme genome plasticity: Helicobacter pylori East Asian genomes
Source: BMC Microbiol. 2011 May 16;11:104. doi: 10.1186/1471-2180-11-104 (PMC3120642; doi:10.1186/1471-2180-11-104)
Supplement: Additional file 6 — Multiple sequence alignments of diverged genes. [file 1471-2180-11-104-S6.ZIP › Diverged_genes_multiple_seuence_alignments/HP0160_hcpD.mfa.rtf]

                   1         11        21        31        41        51        61        71        81        91                           |         |         |         |         |         |         |         |         |         |         HB8:HPB8_1405      --------------MIKSWTKKWFLILFLMASCFGHLVATTGEKYFKMANQALKRGDYHKAVAFYKRSCNLRVGVGCTSLGSMYEDGDGVDQSIPKAVFYHG27:HPG27_147     --------------MIKSWTKKWFLILFLMASCFSHLMATTGEKYFKMANQALKRGDYHRAVAFYKRSCNLRVGVGCTSLGSMYEDGDGVDQSIPKAVFYHB38:HELPY_0164    --------------MIKNWTKKWFLILFLMASCFSYLVATTGEKYFKMANQALKRGDYHRAVAFYKRSCNLRVGVGCTSLGSMYEDGDGVDQNIPKAVFYH266:HP0160        --------------MIKSWTKKWFLILFLMASCSSYLVATTGEKYFKMATQAFKRGDYHKAVAFYKRSCNLRVGVGCTSLGSMYEDGDGVDQNITKAVFYHP12:HPP12_0159    --------------MIKSWTKKWFLILFLMASCSSYLVATAGEKYFKMATQAFKRGDYHKAVAFYKRSCNLRVGVGCTSLGSMYEDGDGVDQNIPKAVFYHHPA:HPAG1_0158    --------------MIKSWTKKWFLILFLMASCSSYLVATTGEKYFKMATQAFKRGDYHKAVAFYKRSCNLRVGVGCASLGSMYEDGDGVDQNVPKAVFYHSJM:mHPSJM_00870  LSLCELWLSFVRNEMIKSWTKKWVLILFLMASCFGHLVATTGEKYFKMANQALKRGDYHRAVAFYKRSCNLRVGVGCTSLGSMYEDGDGVDQNIPKAVFYHF32:HPF32_0169    --------------MIRNWTKKWFLILFLMASCSSYLVATTGEAYFKMATQAFKRGNYHKAVAFYKRSCNLRVGVGCTSLGSMYEDGDGVDQNIPKAVFYHF57:HPF57_0180    --------------MIKSWTKKWFLILFLMASCSSYLVATTGEAYFKMATQAFKRGDYHKAVAFYKRSCNLRVGAGCTSLGSMYEDGDGVDQNIPKAVFYHF16:HPF16_0169    --------------MIKSWTKKWFLILFLMASCSSYLAATTGETYFKMATQAFKRGDYHKAVAFYKRSCNLRVGVGCTSLGSMYEDGDGVDQNIPKAVFYH51:KHP_0159       --------------MIKSWTKKWFLILFLMASCSSYLVATTGEKYFKMATQAFKRGNYHKAVAFYKRSCNLRVGVGCTSLGSMYEDGDGVDQNITKAVFYHF30:HPF30_1135    --------------MIKSWTKKWFLILFLMASCSSYLAATTGETYFKMATQVFKRGNYHKAMAFYKRSCNLRVGAGCTSLGSMYEYGDSVDQNIPKAVFYH52:mHPKB_0168     -----------------------------MASCSSYLVATTGEKYFKMATQAFKRGDYHKAVAFYKRSCNLRVGVSCTSLGSMYEDGDGVDQNIPKAVFY                   101       111       121       131       141       151       161       171       181       191                          |         |         |         |         |         |         |         |         |         |         HB8:HPB8_1405      YRRGCNLRNYLACASLGSMYEDGDGVQKDLPKALYYYRRGCHLKGGVSCGSLGFMYFNGTGVKQNYAKALSLSKYACSLNYGISCNFVGYMYRNAKGVQKHG27:HPG27_147     YRRGCNLRNYLACASLGSMYEDGDGVQKDLPKAIYYYRRGCHLKGGVSCGSLGFMYFNGTGVKQNYAKALSLSKYACSLNYGISCNFAGYMYRNAKGVQKHB38:HELPY_0164    YRRGCNLRDYLACASLGSMYEDGDGVQKNLPKALYYYRRGCHLKGGVSCGSLGFMYFNGTGVKQNYAKALSLSKYACSLNYGISCNFVGYMYRSAKGVQKH266:HP0160        YRRGCNLRNHLACASLGSMYEDGDGVQKNLPKAIYYYRRGCHLKGGVSCGSLGFMYFNGTGVKQNYAKALFLSKYACSLNYGISCNFVGYMYRNAKGVQKHP12:HPP12_0159    YRRGCNLRNYLACASLGSMYEDGDGVQKNIPKALYYYRRGCHLKGGVSCGSLGFMYFNGTGVKQNYAKALSLSKYACSLNYGISCNFVGYIYRNAKGVQKHHPA:HPAG1_0158    YRRGCNLRNHLACASLGSMYEDGDGVQKNLPKAIYYYRRGCHLKGGVSCGSLGFMYFNGTGVKQNYAKALSLSKYACSLNYRISCNFVGYMYRNAKGVQKHSJM:mHPSJM_00870  YRRGCNLRNHLACASLGSMYEDGDGVQKDLPKALYYYRRGCHLKGGVSCGSLGFMYFNGTGVKQNYAKALSLSKYACSLNYGISCNFVGYMYRSAKGVQKHF32:HPF32_0169    YKRGCNLRNHLACASLGSMYEDGDGVQKDLPKAIYYYRRGCHLKGGVSCGSLGFMYFNGIGVKQNYAKALSLSKYACSLDYGISCNFAGYMYRNAKGVEKHF57:HPF57_0180    YRRGCNLRNHLACASLGSMYEDGDGVQKDLPKAIYYYRRGCHLKGGVSCGSLGFMYFNGVGVKQNYAKALSLSKYACSLNYGISCNLAGYMYRNAKGVEKHF16:HPF16_0169    YRRGCNLRNHLACASLGSMYEDGDGVQKDLPKAIYYYRRGCHLKGGVSCGSLGFMYFNGIGVKQNYAKALSLSKYACSLNYGISCNFAGYMYRNAKGAEKH51:KHP_0159       YRRGCNLRNHLACTSLGSMYEDGDGVQKDLPKAIYYYRRGCHLKGGMSCGSLGFMYFNGTGVKQNYAKALSLSKYACSLDYGMSCNFVGYMYRNAKGVEKHF30:HPF30_1135    YKRGCNLRNHLACASLGSMYEDGDGVQKDLPKAIYYYRRGCHLKGGVSCGSLGFMYFNGIGVKQNYAKALSLSKYACSLNYGISCNFAGYMYRNAKGVEKH52:mHPKB_0168     YKRGCNLRNHLACASLGSMYEDGDGVQKDLPKAIYYYRRGCHLKGGVSCGSLGFMYFNGTGVKQNYAKALSLSKYACSLDYGISCNFAGYMYRNAKGVEK                   201       211       221       231       241       251       261       271       281       291                          |         |         |         |         |         |         |         |         |         |         HB8:HPB8_1405      DLKKALANFKRGCHLKDGASCVSLGYMHEAGMNVKQNGEQALNLYKKGCFLKEGSGCHNVAVMYYTGKGTPKDLDKAISYYKKGCTLGFSGSCKVLEEVIHG27:HPG27_147     DLKKALANFKRGCHLKDGASCVSLGYMYEAGMDVKQNGEQALNLYKKGCHLKEGSGCHNVAVMYYTGKDIPKDLDKAVSYYKKGCTLGFSGSCKVLEEVIHB38:HELPY_0164    DLKKALANFKRGCHLKDGASCVSLGYMYEVGMDVKQNGEQALNLYKKGCFLKEGSGCHNVAVMYYTGKGTPKDLDKAISYYKKGCTLGFSGSCKVLEEVIH266:HP0160        DLKKALANFKRGCHLKDGASCVSLGYMYEVGMDVKQNGEQALNLYKKGCYLKRGSGCHNVAVMYYTGKGVPKDLDKAISYYKKGCTLGFSGSCKVLEEVIHP12:HPP12_0159    DLKKALANFKRGCHLKDGASCVSLGYMYETGMDVKQNGEQALNLYKKGCYLKRGSGCHNVAVMYYTGKGAPKDLDKAISYYKKGCTLGFSGSCKVLEEVIHHPA:HPAG1_0158    DLKKALANFKRGCHLKDGASCVSLGYMYEVGMGVKQNGEQALNLYKNGCYLKSGSGCHNVAVMYYTGKGAPKDLDKAISYYKKGCTLGFSGSCKVLEEVIHSJM:mHPSJM_00870  DLKKALANFKRGCHLKDGASCVSLGYMYEVGMDVKQNGEQALNLYKKGCYLKRGSGCHNVAVMYYTGKGAPKDLDKAISYYKKGCTLGFSGSCKVLEEVIHF32:HPF32_0169    DLKKALTNFKRGCHLKDGASCVSLGYMYEAGLYVRQNEEQALNLYKKGCSLKERSGCHNVAVMYYTGKGAPKDLDRATSFYKKGCTLGFSGSCKIL-EVVHF57:HPF57_0180    DLKKALTHFKRGCHLKDGASCVSLGYMYEAGLYVRQNEEQALNLYKKGCSLKEGSGCHNVAVMYYTGKGAPKDLDKATLYYKKGCALGFSGSCKIL-EVVHF16:HPF16_0169    DLKKALTHFKRGCHLKDGASCVSLGYMYEAGLYVRQNEEQALNLYKKGCSLKEGSGCHNVAVMYYMGKGAPKDLDKATSFYKKGCALGFSGSCKIL-EVVH51:KHP_0159       DLKKALTNFKRGCHLKDGASCVSLGYMYEAGLYVRQNEEQALNFYKKGCSLKEGSGCHNVAVMYYTGKGAPKDLDKATSYYKKGCTLGFSGSCKIL-EVVHF30:HPF30_1135    DLKKALTHFKRGCHLKDGASCVSLGYMYETGLYVRQNEEQALNLYKKGCSLKEGSGCHNVAVMYYTGKGASKDLDKATSFYKKGCTLGFSGSCKIL-EVVH52:mHPKB_0168     DLKKALTNFKRGCHLKDGASCVSLGYMYEAGLYVRQNEEQALNLYKKGCSLKEGSGCHNVAVMYYTGKGAPKDLDKAILYYKKGCTLGFSGSCKIL-EVV                   301       311       321                   |         |         |HB8:HPB8_1405      GKKSDDLQDDAQNDTQDDTQHG27:HPG27_147     GKKSDDLQDDAQNDTQDDTQHB38:HELPY_0164    GKKSDNLQDDAQNDTQDDTQH266:HP0160        GKKSDDLQDDAQNDTQDDMQHP12:HPP12_0159    GKKSDNLQDDAQNDTQDDTQHHPA:HPAG1_0158    GKKSDDLQDDAQNDTQDDAQHSJM:mHPSJM_00870  GKKSDNLQDDTQNDTQDDTQHF32:HPF32_0169    GKNSDNLQDDAQNDTQDDTQHF57:HPF57_0180    GKNSDNLQDDAQNDTQDDTQHF16:HPF16_0169    GKKSDNLQDDAQNDTQDDTQH51:KHP_0159       GKKSDNLQDDAQNDTQDDTQHF30:HPF30_1135    GKNSDNLQDDVQNDTQDDTQH52:mHPKB_0168     G-KSDDLQDDAQNDTQDDTQ
