# Supplementary material for: Evolution in an oncogenic bacterial species with extreme genome plasticity: Helicobacter pylori East Asian genomes
Source: BMC Microbiol. 2011 May 16;11:104. doi: 10.1186/1471-2180-11-104 (PMC3120642; doi:10.1186/1471-2180-11-104)
Supplement: Additional file 6 — Multiple sequence alignments of diverged genes. [file 1471-2180-11-104-S6.ZIP › Diverged_genes_multiple_seuence_alignments/HP0373_homC.mfa.rtf]

                  1         11        21        31        41        51        61        71        81        91                          |         |         |         |         |         |         |         |         |         |         HB8:HPB8_421      MLKLVSKTICLSLIGLFNPLEAFQKHQKDGFFIEAGFETGLLEGTQTKEQTITQNTQNTQKIYENPLTH----PQTKEQPKEQNKSDTATPQSAYGKYYIHSJM:HPSJM_05325  MLRLVSKTICLSLIGLFNPLEAFQKNQKDGFFIEAGFETGLLEGAQTKEQTIT---QNTQKVYENPLTH----PQTKEQPKEQNKSDTATPQSVYGRYYIHP12:HPP12_1048   MLRFVSKTICLSLIGLFNPLEAFQKHQKDGFFVEAGFETGLLEGTQTKEETTA---QNTPKIYENPLTH----PQTKEQPKEQNKSDTATPQSAYGKYYIH266:HP0373       MLRFVSKTICLSLIGLFNPLEAFQKHQKDGFFIEAGFETGLLEGTQTKEEVIT-----TQKIYENPLTH----PQTKEQPKEQNKSDTATPQSAYGKYYIHB38:HELPY_1052   MLKLVSKTICLSLIGLFNPLEAFQKNQKDGFFIEAGFETGLLEGTQTKEQTITQNTQNTQKIYENPLTH----PQTKEQPKEQNKSDTATPQSVYGRYYIHHPA:HPAG1_1019   MLRFVSKTICLSLIGLFNPLEAFQKHQKDGFFIEAGFETGLLEGVQTKEEVIT-----TQKIYENPLTH----PQTKEQPKEQNKSDTATPQSAYGKYYIHG27:HPG27_1024   MLRFVSKTICLSLIGLFNPLEAFQKHQKDGFFIEAGFETGLLEGTQTKEEATA---QNIPKIYKNPLTH----PQS----KEQNKSDTATPQSVYGRYYIH52:HPKB_1010     MLRFVSKTICLSLIGLFNPLEAFQKHQKDGFFIEAGFETGLLEGAQTKEEVIT-----TQKIQKNPLTH----PQPNEQPKEQNKSDTATPQSVYGRYYIHF32:HPF32_1019   MLRFVSKTICLSLIGLFNPLEAFQKHQKDGFFIEAGFETGLLEGAQTKEEVI-----TTQKIQKNPLTH----PQPNEQPKEQNKSDTATPQSVYGRYYIHF30:HPF30_0306   MLRFVSKTICLSLIGLFNPLEAFQKHQKDGFFIEAGFETGLLEGAQTKEEVI-----TTQKIQKNPLTY----PQTNEQPKEQNKSDTATPQSVYGRYYIHF57:HPF57_1044   MLRFVSKTICLSLIGLFNPLEAFQKHQKDGFFIEAGFETGLLEGAQTKEEATA---QNTQKIQKNPLTH----LQTNEQPKEQNKSDTATPQSVYGRYYIH51:KHP_0983      MLRFVSKTICLSLIGLFNPLEAFQKHQKDGFFIEAGFETGLLEGAQTKEEATA---QNTQKNPKNPLTH----PQPNEQPKEQNKNDTATPQSVYGRYYIHF16:HPF16_1024   MLRFVSKTICLSLIGLFNPLEAFQKHQKDGFFIEAGFETGLLEGTQTKEEATA---QNTQKIYENHTTHTPSNEQPKEQPKEQNKSDTTTPQSVYGRYYI                  101       111       121       131       141       151       161       171       181       191                         |         |         |         |         |         |         |         |         |         |         HB8:HPB8_421      PQSTILEKATELFTTDNIE-NGLTFYSQNPVYANMV------------NGSVTIQNFLPYNLNNVELSFKDAQGKVVNLGVIETIPKQSQIILPASLFNDHSJM:HPSJM_05325  PQSTILKNATELFTTNNIE-NGLTFYSQSPVYANMV------------NGSVTIQNFLPYNLNNVELSYTDAQGKVVNLGVIETIPKDSQIILPASLFNDHP12:HPP12_1048   PQSTILKNATALFTTNNIE-NGLTFYSQNPVYANMV------------NGSVTIQNFLPYNLNNVELSFKDAQGKVVNLGVIETIPKDSQIILPASLFNDH266:HP0373       PQSTILKNATALFTTDKIE-NGLTFYSQNPVYANMV------------NGSVTIQNFLPYNLNNVELSFKDAQGKVVNLGVIETIPKQSQITLPASLFNDHB38:HELPY_1052   PQSTILKNATALFTTDNIE-NGLTFYSQNPVYANMV------------NGSVTIQNFLPYNLNNVELSFKDAQGKVVNLGVIETIPKDSQIILPASLFNDHHPA:HPAG1_1019   PQSTILKNATALFTTDNIE-NGLTFYSQNPVYANMV------------NGSVTIQNFLPYNLNNVELSFKDAQGKVVNLGVIETIPKDSQIILPASLFNDHG27:HPG27_1024   LQSTILEKATELFTTDNIE-NGLTFYSQNPVYANMV------------NGNVTIQNFLPYNLNNVELSFKDAQGKVVNLGVIETIPKDSQIILPASLFNDH52:HPKB_1010     LQSTILEKATELFTAANINGNGLTFYSQNPVYANMV------------NGSVTIQNFLPYNLNNVELSFKDAQGKVVNLGVIETIPKDSQIILPASLFNDHF32:HPF32_1019   LQSTILEKATELFTAANINGNGLTFYSQNPVYVMAYNKDNAEFEGYGNNSVVVIQNFLPYNLNNIELSYTDAQGKVVNLGVIETIPKDSQIILPASLFNNHF30:HPF30_0306   LQSTILEKATELFTAANINGNGLTFYSQNPVYVMAYNKDNAEFEGYGNNSVVVIQNFLPYNLNNIELSYTDTQGKVVNLGVIETIPKDSQIILPASLFNNHF57:HPF57_1044   LQSTILEKATELFTAANINGNGLTFYSQNPVYVMAYNKDNAEFEGYGNNSVVVIQNFLPYNLNNIELSYTDAQGKVVNLGVIETIPKDSQIILPASLFNNH51:KHP_0983      LQSTILEKATELFTAANINGNGLTFYSQNPVYVMAYNKDNAEFEGYGNNSVVVIQNFLPYNLNNIELSYTDAQGKVVNLGVIETIPKDSQIILPASLFNNHF16:HPF16_1024   LQSTILEKATELFTAANINGNGLTFYSQNPVYVMAYNKDNAEFEGYGNNSVVVIQNFLPYNLNNIELSYTDAQGKVVNLGVIETIPKDSQIILPASLFNN                  201       211       221       231       241       251       261       271       281       291                         |         |         |         |         |         |         |         |         |         |         HB8:HPB8_421      SEFEQADSFN---YQQLQATATQFSDANTQSLFEKLSKITTNVTMSYENAD----TNN-----------------FKGNCHDCVSDFTPQTAEELTNLMLHSJM:HPSJM_05325  SEFEQADSFN---YQQLQATATQFSDANTQSLFEKLGQITTNVVMSYENAD----TNN-----------------FKGNCHDCVSDFTPQTAEELTNLMLHP12:HPP12_1048   SEFEQADSFN---YQQLQATATQFSDANTQSLFEKLSQITTNVTMSYENAD----TNN-----------------FKGNCNDCVSDFTPQTAEELTNLMLH266:HP0373       SEFEQADSFN---YQQLQATATQFSDANTQSLFQKLSKITTNVTMSYENAD----TNN-----------------FKGNCHDCVSDFTPQTAEELTNLMLHB38:HELPY_1052   SEFEQADSFN---YQQLQATATQFSDANTQSLFQKLSQITTNVTMSYENAD----TNN-----------------FKGNCHDCVSDFTPQTAEELTNLMLHHPA:HPAG1_1019   SEFEQADSFN---YQQLQATATQFSDANTQSLFEKLSQITTNVTMSYENAD----TNN-----------------FKGNCNDCVSDFTPQTAEELTNLMLHG27:HPG27_1024   SEFEQADSFN---YQQLQATATQFSDANTQSLFEKISQITTNVTMSYENAD----TNN-----------------FKGNCHDCVSDFTPQTAEELTNLMLH52:HPKB_1010     SEFEQADSFN---YQQLQATATQFSDANTQSLFEKLSKITTNVTMSYENAD----TNN-----------------FKGNCNDCVSDFTPQTAEELTNLMLHF32:HPF32_1019   --FSNDSPFNSDGLQQLQTTTTPFSDANTQSLFEKLSQITTNLQMTYENTDPFSSGNNDPSGPLASPKPHYECPGYKKSCQVASVSFTPQTAEELTNLMLHF30:HPF30_0306   --FSNDSPFNSDGLQQLQTTTTPFSDANTQSLFEKLSQITTNLQMTYENTDPFSSGNNDPSGPLASPKPHYECPGYKKSCQVASVSFTPQTAEELTNLMLHF57:HPF57_1044   --FSNDSPFNSEGLQQLQTTTTPFSDANTQSLFEKLSQITTNLQMTYENTDPFSSGNNDPSGPLASPKPHYECPGYKKSCQVASVSFTPQTAEELTNLMLH51:KHP_0983      --FSNDSPFNSDGLQQLQTTTTPFSDANTQSLFEKLSQITTNLQMTYENTDPFSSGNNDPSGPLASPKPHYECPGYKKSCQVASVSFTPQTAEELTNLMLHF16:HPF16_1024   --FSNDSPFNSDGLQQLQTTTTPFSDANTQNLFEKLSQITTNLQMTYENTDPFSSGNNDPSGPLASPKPHYECPGYKKSCQVASVSFTPQTAEELTNLML                  301       311       321       331       341       351       361       371       381       391                         |         |         |         |         |         |         |         |         |         |         HB8:HPB8_421      DMIAVFDSKSWEEAVLNAPFQFSNSPSECGSDFPKCVNPFNNGRVAPIYEKYVLTPQSVIDAFRRTINLEVNILKS--GFVGLGYELDD---------NDHSJM:HPSJM_05325  DMIAVFDSKSWEEAVLNAPFQFSNSPSECGSDFPKCVNPFNNGRVAPIYEKYVLTPQSVIDAFRRTINLEVNILKS--GFVGLGYELDD---------NDHP12:HPP12_1048   DMIAVFDSKSWEEAVLKAPFQFSNSPSECGSDFPKCVNPFNNGRVAPIYEKYVLTPQSVIDAFRRAINLEVNIMKS--GFLGLGYELDD---------NDH266:HP0373       DMIAVFDSKSWEEAVLNAPFQFSNSSSECGSDFPKCVNPFNNGRVAPIYEKYVLTPQSVIDAFRRTINLEVNILKS--GFVGLGYELDD---------NDHB38:HELPY_1052   DMIAVFDSKSWEEAVLNAPFQFSNSPSECGSDFPKCVNPFNNGRVAPIYEKYVLTPQSVIDAFRRAINLEVNIMKS--GFLGLGYELDD---------NDHHPA:HPAG1_1019   DMIAVFDSKSWEEAVLNAPFQFSNSPSECGSDYPKCVNPFNNGRVAPIYEHYVLTPQSVIDAFRRAINLEVNILKS--GFVGLGYELDD---------NDHG27:HPG27_1024   DMIAVFDSKSWEEAVLNAPFQFSNSPSECGSDFPKCVNPFNNGRVAPIYEKYVLTPQSVIDAFRRTINLEVNILKS--GFVGLGYELDD---------NDH52:HPKB_1010     DMIAVFDSKSWEEAVLNAPFQFSNSPSECGSDFPKCVNPFNNGRVAPIYEKYVLTPQSVINAFRRAINLEVNILKS--GFVGLGYELDD---------NDHF32:HPF32_1019   DMIAVFDSKSWEEAVLNAPFQFSNSPSECGIDYPKCVNPFNNGLVDPKDEKYVLTPEEVINSYRVANELTVNLLNAAKGFLGLGSQLGSANAPGDNGFNQHF30:HPF30_0306   DMIAVFDSKSWEEAVLNAPFQFSNSPSECGIDYPKCVNPFNNGLVDPKDEKYVLTPEEVINSYRVANELTVNLLNAAKGFLGLGSQLGSANAPGDNGFNQHF57:HPF57_1044   DMIAVFDSKSWEEAVLNAPFQFSNSPSECGIDYPKCVNPFNNGLVDPKDEKYVLTPEEVINSYRVANELTVNLLNAAKGFLGLGSQLGSANAPGDNGFNQH51:KHP_0983      DMIAVFDSKSWEEAVLNAPFQFSNSPSECGIDYPKCVNPFNNGLVDPKDEKYVLTPKEVINSYRVANELTVNLLNAAKGFLGLGSQLGSANAPGDNGFNQHF16:HPF16_1024   DMIAVFDSKSWEEAVLNAPFQFSNSPSECGIDYPKCVNPFNNGLVDPKDEKYVLTPEEVINSYRVANELTVNLLNAAKGFLGLGSQLGSANAPGDNGFNQ                  401       411       421       431       441       451       461       471       481       491                         |         |         |         |         |         |         |         |         |         |         HB8:HPB8_421      GNLGIEASALNPEKLFGKTLNKVDIVELRDIIHEFSHTKGYTHNGNMTYQRVRLCQENGGAIQECEGGKEELVNGKEELKFTNGKEVKDQDGYTYDVCSRHSJM:HPSJM_05325  GNLGIAASALNPEKLFGKTLNKVDIVELRDIIHEFSHTKGYTHNGNMTYQRVRLCQENGGAIQECEGGKEELVNGKEELKFTNGKEVKDRDGYTYDVCSFHP12:HPP12_1048   GNLGIAASALNPEKLFGKTLNKVDIVELRDIIHEFSHTKGYTHNGNMTYQRVRLCQENGGAIQECEGGKEELVNGKEELKFTNGKEVKDQDGYTYDVCSFH266:HP0373       GNLGIEASALNPEKLFGKTLNKVDIVELRDIIHEFSHTKGYTHNGNMTYQRVRLCQENGGAIQECEGGKEELVNGKEELKFTNGKEVKDQDGYTYDVCSFHB38:HELPY_1052   GNLGIAASALNPEKLFGKTLNKVDIVELRDIIHEFSHTKGYTHNGNMTYQRVRLCQENGGAIQECEGGKEELVNGKEELKFTNGKEMKDQDGYTYDVCSRHHPA:HPAG1_1019   GNLGIEASALNPEKLFGKTLNKVDIVELRDIIHEFSHTKGYTHNGNMTYQRVRLCQENGGAIQECEGGKEELVNGKEELKFTNGKEVKDQDGYTYNVCSRHG27:HPG27_1024   GNLGIAASALNPEKLFGKTLNKVDIVELRDIIHEFSHTKGYTHNGNMTYQRVRLCQENGGAIQECEGGKEELVNGKEELKFTNGKEVKDQDGYTYNVCSFH52:HPKB_1010     GNLGIEASALNPEKLFGKTLNKVDIVELRDIIHEFSHTKGYTHNGNMTYQRVRLCQEGNGPEVRCEGGHEVEKNGKEELEFSNGHEVKDHDGYDYNVCSRHF32:HPF32_1019   GVLGIAPFALDPEKLFGKNLNKVAILALRDIIHEYGHTLGYTHNGNMTYQRVRLCQEGNGPEVRCEGGHEVEKNGKEELEFSNGHEVRDHDGYDYNVCSRHF30:HPF30_0306   GVLGIAPFALNPEKLFGKNLNKVVILALRDIIHEYGHTLGYTHNGNMTYQRVRLCKEGNGPEVRCEGGHEVEKNGKEELEFSNGHEVRDHDGYDYNVCSRHF57:HPF57_1044   GVLGIAPFALNPEKLFGKNLNKVAILALRDITHEYGHTLGYTHNGNMTYQRVRLCQEGNGPEVRCEGGHEVEKNGKEELEFNNGHEVRDHDGYDYNVCSRH51:KHP_0983      GVLGIAPFALNPEKLFGKNLNKVAILALRDIIHEYGHTLGYTHNGNMTYQRVRLCQEGNGPEVRCEGGREVEKNGKEELEFNNGHEVRDHDGYDYNVCSRHF16:HPF16_1024   GVLGIAPFALNPEKLFGKNLNKVVILALRDIIHEYGHTLGYTHNGNMTYQRVRLCQEGNGPEVRCEGGHEVEKNGKEELEFNNGHEVRDHDGYDYNVCSR                  501       511       521       531       541       551       561       571       581       591                         |         |         |         |         |         |         |         |         |         |         HB8:HPB8_421      FGGKNQPAFPSNYPNSIYTNCAQVPAGLIGVTTAVWQQLINQNALPINFANLNSQANYLDASLNAKAFVGSVFNAFNQNFLTS------SAQQSFRSPILHSJM:HPSJM_05325  YKDNHQVYTAGNYPNSIYTNCAQIPAGLIGVTTAVWQQLINQNALPINFANLSSQANYLDAGLNVQNFATSMVSAIAQNFSTTSTTTYRSSSKNFRSPILHP12:HPP12_1048   YKDNHQVYTAGNYPNSIYTNCAQVPAGLIGVTTAVWQQLINQNALPINFANLSSQANYLNAGLNAQNFATSMISAIAQNFSTTSTTTYRSSSKNFRSPILH266:HP0373       YKDNHQVYTASNYPNSIYTNCAQVPAGLIGVTTAVWQQLINQNALPINFANLNSPTNHLNAGLNAQNFATSIVSAIAQNFSTTSTTTYRSSSKNFRSPILHB38:HELPY_1052   FGGKNQPAFPSNYPNSIYTNCAQVPAGLIGVTTAVWQQLINQNALPINFANLNSQTNHLNAGLNAQNFATSMASAIAQNFSTTSTTTYRSSSKNFRSPILHHPA:HPAG1_1019   FGGKNQPAFPSNYPNSIYTNCAQVPAGLIGVTTAVWQQLINQNALPINFANLNSQTSHLNAGLNAQNFATSMVSAIAQNFSTTSTTTYHSSSKNFRSPILHG27:HPG27_1024   YKDNHQVYTAGNYPNSIYTNCAQVPAGLIGVTTAVWQQLINQNALPINFANLNSPTSHLNAGLNAQNFATSMVNAIAQNFSTTSTTTYRSSNKNFRSPILH52:HPKB_1010     FGGKNQPAFPSNYPNSIYTNCAQVPAGLIGVTSAVWQQLINQNALPINFANLNSQANYLDASLNAQNFATSMVSAIAQNFSTTSTTTYRSSSKNFRSPILHF32:HPF32_1019   FGGKNQSAFPSNYPNSIYTNCAQVPAGLIGVTTAVWQQLINQNALPINFANLNSQANYLDASLNARAFVGSVFSAFNQSFLTS------SAQQSFRSPILHF30:HPF30_0306   FGGKNQPAFPSNYPNSIYTNCAQVPAGLIGVTTAVWQQLINQNALPINFANLNSQANYLDASLNASAFVGSVFNAFNQSFLTS------SAQQSFRSPILHF57:HPF57_1044   FGGKNQPAFPSNYPNSIYTNCAQVPAGLIGVTTAVWQQLINQNALPINFANLNSQANYLDASLNARAFVGSMFNAFNQSFLTS------STQQSFRSPILH51:KHP_0983      FGGKNQPAFPSNYPNSIYTNCAQVPAGLIGVTTAVWQQLINQNALPINFANLNSQANYLDASLNARAFVGSVFNAFNQSFLTS------SAQQSFRSPILHF16:HPF16_1024   FGGKNQPAFPSNYPNSIYTNCAQVPAGLIGVTTAVWQQLINQNALPINFANLNSQANYLDASLNASAFVGSMFNAFNQSFLTS------SISQSFRSPIL                  601       611       621       631       641       651       661       671       681       691                         |         |         |         |         |         |         |         |         |         |         HB8:HPB8_421      GANVKIGYQHYFNDYIGLAYYGIIKYNYAQANDEKIQQLSYGGGMDVLFDFITTYTNKKQNNPTKKVFASSFGVFGGLRGLYNSYYVFNQVKGSGNLDIVHSJM:HPSJM_05325  GVNVKIGYQHYFNDYIGLAYYGIIKYNYAQANDEKIQQLSYGGGMDVLFDFITTYTNKKQGNPTKKVFASSFGVFGGLRGLYNSYYVFNQVKGSGNLDIVHP12:HPP12_1048   GANVKIGYQHYFNDYIGLAYYGIIKYNYAKADDEKIQQLSYGGGMDVLFDFITTYANKKQGNPTKKVFASSFGVFGGLRGLYNSYYVFNQVKGSGNLDIVH266:HP0373       GVNVKIGYQHYFNDYIGLAYYGIIKYNYAKTNDEKIQQLSYGGGMDVLFDFITTYANKKQDNPTKKVFASSFGVFGGLRGLYNSYYVFNQVKGSGNLDIVHB38:HELPY_1052   GANVKIGYQHYFNDYIGLAYYGIIKYNYAQANDEKIQQLSYGGGMDVLFDFITTYTNKKQGNPTKKVFASSFGVFGGLRGLYNSYYVFNQVKGSGNLDIVHHPA:HPAG1_1019   GVNVKIGYQHYFNDYIGLAYYGIIKYNYAKTNDEKIQQLSYGGGMDVLFDFITTYANKKQGNPTKKVFASSFGVFGGLRGLYNSYYVFNQVKGSGNLDIVHG27:HPG27_1024   GVNVKIGYQHYFNDYIGLAYYGIIKYNYAQANDEKIQQLSYGGGMDVLFDFITTYTNKKQNNPTKKVFASSFGVFGGLRGLYNSYYVFNQVKGSGNLDIVH52:HPKB_1010     GVNVKIGYQHYFNDYIGLAYYGIIKYNYAQADDEKIQQLSYGGGMDVLFDFITTYANKKQGNLTKKVFASSFGVFGGLRGLYNSYYVFNQVKGSGNLDIVHF32:HPF32_1019   GVNVKIGYQHYFNDYIGLAYYGIIKYNYAQANDEKIQQLSYGGGMDVLFDFITTYTNKKQGNPTKKVFASSFGVFGGLRGLYNSYYVFSQVKGSGNLDIVHF30:HPF30_0306   GVNVKIGYQHYFNDYIGLAYYGIIKYNYAQANDEKIQQLSYGGGMDVLFDFITTYTNKKQDNPTKKVFTSSFGVFGGLRGLYNSYYVFNQVKGSGNLDIVHF57:HPF57_1044   GVNVKIGYQHYFNDYIGLAYYGIIKYNYAQANDEKIQQLSYGGGMDVLFDFITTYTNKKQDNPTKKVFASSFGVFGGLRGLYNSYYVFNQVKGSGNLDIVH51:KHP_0983      GVNVKIGYQHYFNDYIGLAYYGIIKYNYAQANDEKIQQLSYGGGMDVLFDFITTYTNKKQDNPTKKVFASSFGVFGGLRGLYNSYYVFNQVKGSGNLDIVHF16:HPF16_1024   GVNVKIGYQHYFNDYIGLAYYGIIKYNYAQANDEKIQQLSYGGGMDVLFDFITTYTNKKQDNPTKKIFASSFGVFGGLRGLYNSYYVFSQVKGSGNLDIV                  701       711       721       731       741       751                  |         |         |         |         |         |HB8:HPB8_421      TGFNYRYKHSKYSIGISVPLMQNDIKIASNNGIYANSVVLNEGGSHFKVFFNYGWIFHSJM:HPSJM_05325  TGFNYRYKHSKYSIGISVPLIQSGIKIASNNGIYANSVVLNERGSHFKVFFNYGWVFHP12:HPP12_1048   TGFNYRYKHSKYSIGISIPLIQSGIKIASNNGIYANSVVLNEGGSHFKVFFNYGWIFH266:HP0373       TGFNYRYKHSKYSVGISVPLIQSGIKIASNNGIYANSVVLNEGGSHFKVFFNYGWIFHB38:HELPY_1052   TGFNYRYKHSKYSIGISVPLIQSGIKIASNNGIYANSVVLNEGGSHFKVFFNYGWVFHHPA:HPAG1_1019   TGFNYRYKHSKYSIGISVPLIQSGIKIASNNGIYANSVVLNEGGSHFKVFFNYGWIFHG27:HPG27_1024   TGFNYRYKHSKYSVGISVPLIQSNIQIASSNGIYANSVVLNEGGSHFKVFFNYGWIFH52:HPKB_1010     TGFNYRYKHSKYSVGISVPLIQSGIKIASNNGIYANSVVLNEGGSHFKVFFNYGWIFHF32:HPF32_1019   TGFNYRYKHSKYSVGISVPLIQSDIKIASNNGIYADSVVLNEGGSHFKVFFNYGWIFHF30:HPF30_0306   TGFNYRYKHSKYSVGISVPLIQSGIKIASDNGIYADSVVLNEGGSHFKVFFNYGWIFHF57:HPF57_1044   TGFNYRYKHSKYSVGISVPLIQSDIEIASNNGVYADSVVLNEGGSHFKVFFNYGWIFH51:KHP_0983      TGFNYRYKHSKYSVGISVPLIQSDIEIASNNGIYADSVVLNEGGSHFKVFFNYGWIFHF16:HPF16_1024   TGFNYRYKHSKYSVGISVPLIQSGIKIASNNGIYADSVVLNEGGSHFKVFFNYGWIF
