# Supplementary material for: Evolution in an oncogenic bacterial species with extreme genome plasticity: Helicobacter pylori East Asian genomes
Source: BMC Microbiol. 2011 May 16;11:104. doi: 10.1186/1471-2180-11-104 (PMC3120642; doi:10.1186/1471-2180-11-104)
Supplement: Additional file 6 — Multiple sequence alignments of diverged genes. [file 1471-2180-11-104-S6.ZIP › Diverged_genes_multiple_seuence_alignments/HP0384.mfa.rtf]

                  1         11        21        31        41        51        61        71        81        91                          |         |         |         |         |         |         |         |         |         |         HB8:HPB8_433      MQKSILKMTLLLVFLFLRNAVGLEDEKA--DPKSVQNTPKNLPPIQLRLNQVHEELIEMLENMGKGTQYEFPKIKEILEQSEEEWLKVAHEECVALVMLIHB38:HELPY_1041   MQKNILKMTLLLVFLFLRSAVGLEDKKA--DPKSVQNTPKNLPPIQLRLNQVHEELIEMLENMGKGTQYEFPKIKEILEQSEEEWLKVAHEECVALVMLIHG27:HPG27_1013   MQKSILKMTLLLVFLFLRNAVGLEDKKA--DPKSVQNTPKNLPPIQLRLNQVHEELIEMLENMEKGTQYEFPKIKEILEQSEEEWLKVAHEECVALVMLIHHPA:HPAG1_1008   MQKSILKMTLLLVFLFLRNAVGLEDKKA--DLKSVQNTPKDLPPIQLRLNQVHEELIEMLENMGKGTQYEFPKIKEILEQSEEEWLKVAHEECVALVMLIHSJM:HPSJM_05265  MQKSILKMTLLLVFLFLRNAVGLEDKKA--DPKSVQNTPKNLPPIQLRLDQAHEDLIKMLDNMGKSTQYEFPKIKEILEQSEEEWLKVAHEECVALVMLIHP12:HPP12_1036   MQKSILKMTLLLVFLFLRNAVGLEDKKA--DLKSVQNTPKNLPPIQLRLDQAYEDLIKMLDNMGKSTQYEFPKIKEILEQSEEEWLKVAHEECVALVMLIH266:HP0384       MQKNILKMTLLLVFLFLRNAVGLEDKKATTQPESVQNTPKDLPPIQLRLNQVHEELIEMLENMGKGTQYEFPKIKEILEQSEEEWLKVAHEECVALVMLIHF32:HPF32_0334   MQKNILKMTLLLVFLFLRIAVGLEDKKA--DPKSVQNTPKNLPPIQLRLNQVHEELIEMLENMGKGTQYEFPKIKEILEESEEEWLKVAHEECVALVMLIHF57:HPF57_1033   MQKNILKMTLLLVFLFLKNAVGLEDKKA--DSKSVQNTPKNLPPIQLRLNQVHEELIEMLENMGKGTQYEFPKIKEILEQSEEEWLKVAHEECVVLVMLIHF30:HPF30_0317   MQKNILKMTLLLVFLFLKNAVGLENKKA--DPKSVQNTPKNLPPIQLRLNQVHEELIEMLENMGKGTQYEFPKIKEILEQSEEEWLKVAHEECVALVMLIHF16:HPF16_1012   MQKNILKMTLLLVFLFLKNAVGLEDKKA--DPKSVQNTPKNLPPIQLRLNQVHEELIEMLENMGKGTQYEFPKIKEILEQSEEEWLKVAHEECVALVMLIH51:KHP_0972      MQKNILKMTLLLVFLFLKNAVGLEDKKA--DPKSVQNTPKNLPPIQLRLNQVHEELIEMLENMGKGTQYEFPKIKEILEQSEEEWLKVAHEECVALVMLIH52:HPKB_0999     MQKNILKITLLLVFLFLRNAVGLEDKKA--DLKSVQNTPKNLPPIQLRLNQVHEELIEMLENMEKGTQYEFPKVKEILEQSEEEWLKVAHEECVALVMLI                  101       111       121       131       141       151       161       171       181       191                         |         |         |         |         |         |         |         |         |         |         HB8:HPB8_433      SPKASIENSPIYKNCYEAYVKQRIHDLYDFYIEGKKVKRKIKKAHKQEAAIKQSQPLTKESPKSENKKSLTKPSLKDAGIPKGYYLQIGAFLNAPSKDFLHB38:HELPY_1041   SPKASIENSPIYKNCYEAYVKQRIHDLYDFYIESKKVKRKIKKAHKQETAINQSQPLKKEPPKSENKKSLTKPSLKDASIPKGYYLQIGAFLNAPSKDFLHG27:HPG27_1013   SPKASIENSPIYKNCYEAYVKQRIHDLYDFYIESKKVKRKIKKAHKQETAINQSKPLTKEPPKNENKKSLVKPSLKDASIPKGYYLQIGAFLNAPSKDFLHHPA:HPAG1_1008   SPKASIENSPIYKNCYEAYVKQRIHDLYDFYIESKKVKRKIKKAHKQEVAIKQSQPLKKEPPKSENKKSLTKPSLKDTSIPKGYYLQIGAFLNAPSKDFLHSJM:HPSJM_05265  SPKASIENSPIYRNCYEAYVKQRIHDLYDFYIESKKVKRKIKKAHKQETAINQSQPLKKEPPKSENKKSLVKPSLKDASVPKGYYLQIGAFLNAPSKDFLHP12:HPP12_1036   SPKASIENSPIYRNCYEAYVKQRIHDLYDFYIEGKKVKRKIKKTHKQETAINQSQPLTKESPKNENKKSLVKPNLKDASIPKGYYLQIGAFLNAPSKDFLH266:HP0384       SPKASIENSPIYKNCYEAYVKQRIHDLYDFYIESKKVKRKIKKAHKQETAINQSQPLTKESPKNENKKNLVKPNLKDASIPKGYYLQIGXXLNAPSKDFLHF32:HPF32_0334   HPKASIENSPIYKNCYEAYVKQRIHDLYDFYVESKKVKRKIKKAHKHALIQNESKPLTKEPPKDEDKKSLTKPSLKDASIPKGYYLQIGAFLNAPSKDFLHF57:HPF57_1033   SPKASIKNSPIYKNCYEAYVKQRIHDLYDFYIESKKVKRKIKKAHKHALIQNESKPLTKESPKNEDKKSLTKPSLKDASIPKGYYLQIGAFLNVPSKDFLHF30:HPF30_0317   SPKASIKNSPIYKNCYEAYVKQRIHDLYDFYIESKKVKRKIKKAHKHALIQNESKPLTKEPPKNEDKKSLTKPSLKDASIPKGYYLQIGAFLNAPSKDFLHF16:HPF16_1012   SPKASIKNSPIYKNCYEAYVQQRIHDLYDFYIESKKVKRKIKKAHKHAVIQNESKPLTKEPPKSENKKSLIKPSLKDASIPKGYYLQIGAFLNAPSKGFLH51:KHP_0972      SPKASIKNSPIYKNCYEAYVQQRIHDLYDFYIESKKVKRKIKKAHKHALIQNESKPLTKEPPKDEDKKSLIKPSLKDASIPKGYYLQIGAFLNAPSKGFLH52:HPKB_0999     SPKASIENSPIYKNCYEAYVKQRIHDLYDFYIESKKVKRKIKKAHKHVLIQNESKPLTKEPPKDEDKKSLVKPSLKDANIPKGYYLQIGAFLNAPSKDFL                  201       211       221       231       241       251                  |         |         |         |         |         |HB8:HPB8_433      QTLKTFPYQIKKKDSLTHYLIGPYKTKEEALKQLENATKSFKNRPVLVEKHB38:HELPY_1041   QTLKTFPYQIKKKDSLTHYLIGPYKTKEEALKQLENATKSFKNNPVLVEKHG27:HPG27_1013   QTLKTFPYQIKKKDSLTHYLIGPYKTKEEALKQLENALKSFKNKPVLVEKHHPA:HPAG1_1008   QTLKTFPYQIKKKDSLTHYFIGPYKTKEEALKQLENALKSFKNKPVLVEKHSJM:HPSJM_05265  QTLKTFPYQIKKKDSLTHYFIGPYQTKEEALKQLENAAKSFKNKPVLVEKHP12:HPP12_1036   QTLKTFPYQIKKKDSLTHYFIGPYKTKEEALKQLENAAKSFKNKPVLVEKH266:HP0384       QTLKTFPYQIKKKDSLTHYFIGPYKTKEEALKQLENAIKSFKNKPVLVEKHF32:HPF32_0334   QTLKTFPYQIKKKDSLTHYFIGPYKTKEEALKQLENAAKNFKNKPVLVEKHF57:HPF57_1033   QTLKTFPYQMEKKDSLTHYFIGPYQTKEEALKQLENAAKNFKNKPVLVEKHF30:HPF30_0317   QTLKTFPYQMEKKDSLTHYFIGPYQTKEEALKQLENAAKNFKNKPVLVEKHF16:HPF16_1012   QTLKTFPYQMEKKDSLTHYFIGPYQTKEEALKQLENAAKNFKNKPVLVEKH51:KHP_0972      QTLKTFPYQMEKKDSLTHYFIGPYKTKEEALKQLENAAKSFKNKPVLVEKH52:HPKB_0999     QTLKTFPYQIKKKDSLTHYFIGPYQTKEEALKQLENAAKNFKNKPVLVEK
