# Supplementary material for: Evolution in an oncogenic bacterial species with extreme genome plasticity: Helicobacter pylori East Asian genomes
Source: BMC Microbiol. 2011 May 16;11:104. doi: 10.1186/1471-2180-11-104 (PMC3120642; doi:10.1186/1471-2180-11-104)
Supplement: Additional file 6 — Multiple sequence alignments of diverged genes. [file 1471-2180-11-104-S6.ZIP › Diverged_genes_multiple_seuence_alignments/HP0465.mfa.rtf]

                  1         11        21        31        41        51        61        71        81        91                          |         |         |         |         |         |         |         |         |         |         HB8:HPB8_1118     MPFLKALESFDAPFLEKEISKRFRDNLIFFKSYNPNLFNALNTPFKNYQLLFEKNHFNLLHTPTNALSYPKNQMIETAFNMASNPLNNPRYSLDNNHLSLHG27:HPG27_423    MPFLKALESFDAPFLEKEISKRFRDNLVFFKSYNPHLFNALNTPFKNYQLLFEKNHFNLLHTPTNALSYPKNQMIEIAFNMASNPLNNPRYSLDNNHLSLHB38:HELPY_0448   MPFLKALESFDAPFLEKEISKRFRDNLVFFKSYHPNLFNALNTPFKNYQLLFEKNHFNLLHTPTNALSYPKNQMIETAFNMASNPLNNPRWSLDNNHLSLHSJM:HPSJM_02330  MPFLKALESFDAPFLEKEISKRFRDNLVFFKSYNPNLFNALNTPFKNYQLLFEKNHFNLLHTPTNALSYPKNQMIETAFNMASNPLNNPRYSLDNNHLSLHP12:HPP12_0474   MPFLKALESFDTPFLEKEISKRFRDNLVFFKSYNPHLFNALNTPFKNYQLLFEKNHFNLLHTPTNALSYPKNQMIKIAFNMAKSPLNNPRYSLDNNHLSLHHPA:HPAG1_0441   MPFLKALESFDVPYLEKEISKRFRDNLVFFKSYHPNLFNALNTPFKNYQLLFEKNHLNLLHTPTNALSYPKNQMIETAFNMASNPLNNPRYSLDNNHLSLH266:HP0465       MPFLKALESFDAPFLEKEISKRFRDNLVFFKSYNPNLFNALNTPFKNYQLLFEKNHFNLLHTPTNALSYPKNQMIEIAFNMASNPLNNPKWSLDNNHLSLHF32:HPF32_0867   MPFLKALESFDAPFLEKEILKRFRDNLVFFKSYNPHLFNALNTPFKNYQLLFEKNHFNLLHTPTNALSYPKNQMIEIAFNMASNPLNNPRWSLDNNHLSLHF30:HPF30_0857   MPFLKALESFDVPFLEKEILKRFRDNLVFFKSYNPHLFNALNTPFKNYQLLFEKNHFNLLHTPTNALSYPKNQMVGIAFNMASNPLNNPRWSLDNNHLSLH52:HPKB_0447     MPFLKALESFDVPFLEKEFLKRFRDNLVFFKSYNPHLFNALNTPFKNYQLLFEKNHFNLLHTPTNALSYPKNQMVGIAFNMASNPLNNPRWSLDNNHLSLHF57:HPF57_0492   MPFLKALESFDAPFLEKEILKRFRDNLVFFKSYNPHLFNALNTPFKNYQLLFEKNHFNLLHTPTNALSYPKNQMVGIAFNIASNPLNNPRWSLDNNHLSLH51:KHP_0429      MPFLKALESFDAPFLEKEILKRFRDNLVFFKSYNPHLFNALNTPFKNYQLLFEKNHFNLLHTPTNALSYPKNQMVEIAFNMASNPLNNPRWSLDNNHLSLHF16:HPF16_0445   MPFLKALESFDAPFLEKEILKRFRDNLVFFKSCNPHLFNALNTPFKNYQLLFEKNHFNLLHTPTNALSYPKNQMVEIAFNMASNPLNNPRWSLDNNHLSL                  101       111       121       131       141       151       161       171       181       191                         |         |         |         |         |         |         |         |         |         |         HB8:HPB8_1118     HYLKSQNNPKLPLTLKATHAISNFLNNHQTPCSLEKFLPPTMIYGVLDGLFLAILQAQNYRFHSLYLFEENLDLFKISCYFARYEDLITKGAKLFIQGFFHG27:HPG27_423    HYLKTQNNPKLPLTLKATHAISNFLDNHQTPCSLEKFLPPTMIYGVLDGLFLAILQAQNYRFHSLYLFEENLDLFKISCYFARYEDLITKGAKLFIQGFFHB38:HELPY_0448   NYLKSQNNPKLPLTLKATHAISNFLDNHQTPCSLEKFLPPTMIYGVLDGLFLAILQAQNYRFHSLYLFEENLDLFKISCYFVRYEDLITKGAKLFIQGFFHSJM:HPSJM_02330  HYLKTQNNPKLPLTLKATHAISNFLNNHQTPCSLKKFLPPTMIYGVLDGLFLAILQAQNYRFHSLYLFEENLDLFKISCYFVRYEDLIIKGAKLFIQGFFHP12:HPP12_0474   HYLKTQNNPKLPLTLKATHAISNFLNNHQTPCSLKKFLPPTMIYGVLDGLFLAILQAQNYRFHSLYLFEENLDLFKISCYFARYEDLIIKGAKLFIQGFFHHPA:HPAG1_0441   HYLKSQNNPKLPLTLKATHAISNFLDNYQTPCSLKKFLPPTMIYGVLDGLFLAILQAQNYRFHSLYLFEENLDLFKISCYFARYEDLITKGAKLFIQGFFH266:HP0465       HYLKSQNNPKLPLTLKATHAISNFLNNHQTPCSLKKFLPPTMIYGVLDGLFLAILQAQNYRFHSLYLFEENLDLFKISCYFVRYEDLITKGAKLFIQGFFHF32:HPF32_0867   NYLKTQNNPKLPLTLKATHAISNFLDGYQTPCSLKKFLPPTMIYGVLDGLFLAILQAQNYRFHSLYLFEENLDLFKISCYFARYEDLITKGAKLFIEGFFHF30:HPF30_0857   NYLKTQNNPKLPLTLKATHAISNFLDGYQTPCSLKKFLPPTMIYGVLDGLFLAILQAQNYRFHSLYLFEENLDLFKISCYFARYEDLITKGAKLFIQGFFH52:HPKB_0447     NYLKTQNNPKLPLTLEATHAISNFLDGYQTPCSLKKFLPPTMIYGVLDGLFLAILQAQNYRFHSLYLFEENLDLFKISCYFVRYEDLITKGAKLFIEGFFHF57:HPF57_0492   NYLKTQNNPKLPLTLKATHAISNFLDGYQTPCSLKKFLPPTMIYGVLDGLFLAILQAQNYRFHSLYLFEENLDLFKISCYFARYEDLITKGAKLFIQGFFH51:KHP_0429      HYLKTQNNPKLPLTLEATHAISNFLDNYQTPCSLKKFLPPTMIYGVLDGLFLAILQAQNYRFHSLYLFEENLDLFKISCYFVRYEDLMIKGAKVFIQGFFHF16:HPF16_0445   NYLQTQNNPKLPLTLKATHAISNFLDDYQTPCSLKKFLPPTMIYGVLDGLFLAILQAQNYRFHSLYLFEENLDLFKISCYFVRYEDLIIKGAKLFIQGFF                  201       211       221       231       241       251       261       271       281       291                         |         |         |         |         |         |         |         |         |         |         HB8:HPB8_1118     NPNELKMDFLKRPVTHSFLKLEIMPYKSAFNSRMRENIQSYYKQALRGWGSFEDELLGLKNTLKNLPLYQTLKTKPKKINAPICVVGNGPSLDLLLDFLKHG27:HPG27_423    NPNELKMDFLKRPVTHSFLKLEIMPYKSAFNLRMRENIQSYYKQALRGWGSFEDELLGLKNTLKNLPLYQTLKTKPKKINAPICVVGNGPSLDLLLDFLKHB38:HELPY_0448   NPNELKMDFLKRPVTHSFLKLEIMPYKSAFNLHMRENIQSYYKQALRGWGSFEDELLGLKNTLKNLPLYQTLKTKPKKINAPICVVGNGPSLDLLLDFLKHSJM:HPSJM_02330  NPNELKMDFLKRPVTHSFLKLEIMPYKSAFNLRMRENIQSYYKQALRGWGSFEDELLGLKNTLKNLPLYQTLKTKPKKINAPICVVGNGPSLDLLLDFLKHP12:HPP12_0474   NPNELKMDFLKRPITHSFLKLEIMPYKSAFNSRMKENIQSYYKQALRGWGSFEDELLGLKNTLKNLPLYQTLKTKPQKINAPICVVGNGPSLDLLLDFLKHHPA:HPAG1_0441   NPNELKMDFLKRPITHSFLKLEIMPYKSAFNSCMRENIQSYYKQALRGWGSFEDELLGLKNTLKNLPLYQTLKIKPKKINAPICVVGNGPSLDLLLDFLKH266:HP0465       NPNELKMDFLKRPVTHSFLKLEIMPYKSAFNLRMRENIQSYYKQALRGWGSFEDELLGLKNTLKNLPLYQTLKIKPKKINAPICVVGNGPSLDLLLDFLKHF32:HPF32_0867   NPNELKMDFLKRPITHSFLKLEIMPYKSAFNSRMKENIQSYYKQALRGWGSFEDELLGLKNTLKNLPLYQTLKTKPEKINAPICVVGNGPSLDLLLDFLKHF30:HPF30_0857   NPNELKMDFLKRPITHSFLKLEIMPYKSAFNSRMKENIQSYYKQALRGWGSFEDELLGLKNTLKNLPLYQTLKTKPKKINAPICVVGNGPSLDLLLDFLKH52:HPKB_0447     NPNELKMDFLKRPITHSFLKLEIMPYKSAFNSRMKENIQSYYKQALRGWGSFEDELLGLKNTLKNLPLYQTLKTKPKKINAPICVVGNGPSLDLLLDFLKHF57:HPF57_0492   NPNELKMDFLKRPITHSFLKLEIMPYKSAFNSRMKENIQSYYKQALRGWGSFEDELLGLKNTLKNLPLYQTLKTKPKKINAPICVVGNGPSLDLLLDFLKH51:KHP_0429      NPNELKMDFLKRPITHSFLKLEIMPYKSTFNSRMKENIQSYYKQALRGWGSFEDELLGLKNTLKNLPLYQTLKTKPKKINAPICVVGNGPSLDLLLDFLKHF16:HPF16_0445   NPNELKMDFLKRPITHSFLKLEIMPYKSAFNSRMKENIQSYYKQALRGWGSFEDELLGLKNTLKNLPLYHTLKTKPKKINAPICVVGNGPSLDLLLDFLK                  301       311       321       331       341       351       361       371       381       391                         |         |         |         |         |         |         |         |         |         |         HB8:HPB8_1118     ENEDNFIIFSCGTALKPLKTHGIKVDFQIEVERIDYLKEVLEKAPLEDTPLIGANMLNPNAFNLAKEALMFMRGGSACAYISPLSVEYAAPFVGNAGVALHG27:HPG27_423    ENEDHFIIFSCGTALKPLKTHGVKVDFQIEVERIDYLKEVLEKAPLEDTPLMGANMLNPNAFNLAKEALMFMRGGSACAYISPLSIEYAAPFVGNAGVALHB38:HELPY_0448   ENEDNFIIFSCGTALKPLKTHGVKVDFQIEVERIDYLKEVLEKAPLEDTPLMGANMLNPNAFNLAKEALMFMRGGSACAYISPLSIEYAAPFVGNAGVALHSJM:HPSJM_02330  ENEDNFIIFSCGTALKPLKAHGVKVDFQIEVERIDYLKEVLEKAPLEDTPLMGANMLNPNAFNVAKEALMFMRGGSACAYISPLSIEYAAPFVGNAGVALHP12:HPP12_0474   ENEENCIIFSCGTALKPLKAHGVKVDFQIEVERIDYLKEVLEKAPLEDTPLMGANMLNPNAFNLAKEALMFMRGGSACAYISPLSIEYAAPFVGNAGVALHHPA:HPAG1_0441   ENEDNFIIFSCGTALKPLKTHGVKVDFQIEVERIDYLKEVLEKAPLEDTPLMGANMLNPNAFNLAKEALMFMRGGSACAYISPLSVEYAAPFVGNAGVALH266:HP0465       ENEKNCIIFSCGTALKPLKTHGVKVDFQIEVERIDYLKEVLENAPLEDTPLMGANMLNPNAFNVAKEALMFMRGGSACAYISPLSIEYAAPLVGNAGVALHF32:HPF32_0867   ENEDHFIIFSCGTALKPLKTHGIKVDFQIEVERIDYLKEVLEKAPLEDTPLIGANMLNPNAFNVAKEAFMFMRGGSACAYISPLSIEYAAPFVGNAGVALHF30:HPF30_0857   ENEDHFIIFSCGTALKPLKTHGIKVDFQIEVERIDYLKEVLEKAPLEDTPLIGANMLNPNAFNIAKEAFMFMRGGSACAYISPLSIEYAAPFVGNAGVALH52:HPKB_0447     ENEDHFIIFSCGTALKPLKTHGIKVDFQIEVERIDYLKEVLEKAPLEDTPLIGANMLNPNAFNVAKEAFMFMRGGSACAYINPLNIEYAAPFVGNAGVALHF57:HPF57_0492   ENEDHFIIFSCGTALKPLKTHGIKVDFQIEVERIDYLKEVLEKAPLEDTPLIGANMLNPNAFNVAKEAFMFMRGGSACAYISPLSIEYAAPFVGNAGVALH51:KHP_0429      ENEDHFIIFSCGTALKPLKTHGVKVDFQIEVERIDYLKEVLEKAPLEDTPLIGANMLNPNAFNIAKEAFMFMRGGSACAYISPLNIEYAAPFVGNAGVALHF16:HPF16_0445   ENEDHFIIFSCGTALKPLKTHGIKVDFQIEVERIDYLKEVLEKAPLEDTPLIGANMLNPNAFNIAKEAFMFMRGGSACAYISPLNIEYAAPFVGNAGVAL                  401       411       421       431       441       451       461       471       481       491                         |         |         |         |         |         |         |         |         |         |         HB8:HPB8_1118     AGLMSDEIYLCALDCAYIKGFKKHAQNSYYENEKEIDTSSLISVEGNFKGYETFSDSLFLLSKERIEEALHYYQPKKVYNLSYGAKIKHAVSLNHSQVKLHG27:HPG27_423    AGLMSDEIYLCALDCAYIKGFKKHAQNSYYENEKEIDTSSLISIEGNFKGYETFSDSLFLLSKERIEEALHYYQPKKVYNLSYGAKIKHAVSLNHSQVKLHB38:HELPY_0448   AGLMSDEIYLCALDCAYIKGFKKHAQNSYYGDEKEIDTSSLISVEGNVEGYETFSDSLFLLSKERIEEALHYYQPKKVYNLSYGAKIKHAVSLNRSQVKLHSJM:HPSJM_02330  AGLMSDEIYLCALDCAYIKGFKKHAQNSYYENEKEIDTSSLISVEGNVEGYETFSDSLFLLSKERIEEALHHYQPKKVYNLSYGAKIKHAVSLNHSQVKLHP12:HPP12_0474   AGLMSDEIYLCALDCAYIKGFKKHAKNSYYENEKEIDTSSLISVEGNVEGYETFSDSLFLLSKERIEEALHYYQPKKVYNLSYGAKIKHAVSLNYSQVKLHHPA:HPAG1_0441   AGLMSDEIYLCALDCAYIKGFKKHAQNSYYENEKEIDTSSLISIESNFKGYETFSDSLFLLSKERIEEALYYYQPKKVYNLSYGAKIKHAVSLNRSQVKLH266:HP0465       AGLMSDEIYLCALDCAYIKGFKKHAQNSYYGDEKEIDTSSLISVEGNFKGYETFSDSLFLLSKERIEEALNHYQPKKVYNLSYGAKIKHAVSLNYSQVKLHF32:HPF32_0867   ASLMSDEIYLCALDCAYIKGFKKHAQNSYYENEKEIDPSSLISVEGNFKGYETFGDSLFLLSKERIEEALNYYQPKKVYNLSYGAKIKHAVSLNRSQVKLHF30:HPF30_0857   ASLMSDEIYLCALDCAYIKGFKKHAQNSYYENEKEIDPSSLISVECNFKGYETFSDSLFLLSKERIEEALNYYQPKKVYNLSYGAKIKHAVSLNRSQVKLH52:HPKB_0447     ASLMSDEIYLCALDCAYIKGFKKHAQNSYYENEKEIDPSSLISVESNFKGYETFSDSLFLLSKERIEEALNYYQPKKVYNLSYGAKIKHAVSLNYSQVKLHF57:HPF57_0492   ASLMSDEIYLCALDCAYIKGFKKHAQNSYYENEKEIDPLSLISVEGNFKGYETFSDSLFLLSKERIEEALNYYQPKKVYNLSYGAKIKHAVSLNYSQVKLH51:KHP_0429      ASLMSDEIYLCALDCAYIKGFKKHAQNSYYENEKEIDPSSLISVEGNFKGYETFSDSLFLLSKERIEEALNYYQPKKVYNLSYGAKIRHAVSLNYSQVKLHF16:HPF16_0445   ASLMSDEIYLCALDCAYIKGFKKHAQNSYYENEKEIDPSSLISVEGNFKGYETFSDSLFLLSKERIEEALNYYQPKKVYNLSHGAKIKHAVSLNHSQVKL                  501       511       521       531       541       551       561       571       581       591                         |         |         |         |         |         |         |         |         |         |         HB8:HPB8_1118     KQINKQEAIARIKSMFSPKNNHAKDLNNLQKNLMSFKEDFFTHLNTPCKTKQEIFEWVDSLSGFCQTASAKTPTIGILFEGSIAHILQSVLIVSLHLNENHG27:HPG27_423    KQINKQDAIARIKSMFSPRSNHAKDLNNLQKNLIRFKEGFFTQLNTPHKTKQEIFEWVDSLSGFCQTASAKTPTIGILFEGSIAHILQSVLIVSLHLNENHB38:HELPY_0448   KQINKQDATARIKSMFNPPNNHAKDLNNLQKNLMSFKEDFFTHLNTPCKTKQEIFEWVDSLSGFCQTASAKTPTIGILFEGSIAHILQSVLIVSLHLNENHSJM:HPSJM_02330  KHSNKQDAIARIKSMFSPKNNHAKDLNNLQKNLIRFKEDFFTHLNTPCKTKQEIFEWVDNLSGFCQTASAKTPTIGILFEGSVAHILQSVLIVSLHLKENHP12:HPP12_0474   KQINKQEAIARIKSMFSPKNNHAKDLNNLQKNLIRFKEDFFTHLNTPCKTKQEIFEWVDSLSGFCQTASAKTPTIGILFEGSVAHILQSVLIVSLHLNENHHPA:HPAG1_0441   KHSNKQEAIARIKSMFSPRSNHAKDLNNLQKNLIRFKEDFFTQLNTPCKTKQEIFEWVDSLSGFCQTISAKTPTIGILFEGSIAHILQSVLIVSLHLKENH266:HP0465       KHSNKQEAIARIKSMFNPPNNHAKDLKNLQKNLMNFKESFFTHLNTPCKTKQEIFEWVDSLSGFCQTISAKTPTIGILFEGSVAHILQSVLIVSLHLNENHF32:HPF32_0867   KPSNKLEAIARIKSMFSPKSNHAKDLKNLQKNLIGFKENFFMRLNTPCKTKQEIFEWVDNLNGFCQTTSTKTPTIGILFEGSIAHILQSVLIVSLHLNENHF30:HPF30_0857   KPSNKQEAIARIKSMFSPKSNHAKDLKNLQKNLISFKEDFFMRLNTPCKTKQEIFEWVDNLNGFCQATSAKTPTIGILFEGSIAHILQSVLIVSLHLNENH52:HPKB_0447     KPSNKQEAITRIKSMFSPKSNHAKDLKNLQKNLISFKEDFFMRLNTPCKTKQEIFEWVDNLNGFCQTTSAKTPTIGILFEGSIAHILQSVLIVSLHLNENHF57:HPF57_0492   KPSNKQEAIARIKNMFSPRSNHAKDLKNLQKNLISFKEDFFTHLNTPCKTKQEIFEWVDSLSGFCQTTSAKTPTIGILFEGSIAHILQSVLIVSLHLNENH51:KHP_0429      KPSNKQEATARIKSMFSPKSNHAKDLNNLQKNLISFKEDFFMRLNTPCKTKQEIFEWVDNLNGFCQTTSAKTPTIGILFEGSIAHILQSVLIVSLHLNENHF16:HPF16_0445   KPSNKQEAIALIKSMFSPKSNHAKDLKNLQKNLISFKEDFFMRLNTPCNTKQEIFEWVDNLNGFCQTTSAKTPTIGILFEGSIAHILQSVLIVSLHLNEN                  601       611       621       631                  |         |         |         |HB8:HPB8_1118     ELTRFIKFSQNALKQFLKEACLLLQMQLKQPHG27:HPG27_423    ELTHFIKFSQNALKQFLKEACLLLQRQLKQPHB38:HELPY_0448   ELTHFINYSQNALKQFLKKACLLLQIQLKQPHSJM:HPSJM_02330  ELTHFINHSQNTLKQFLKKACLLLQRQLKQPHP12:HPP12_0474   ELTHFINHSQNTLKQFLKEACLLLQMQFKQPHHPA:HPAG1_0441   ELTHFIKFSQNALKQFLKEACLLLQMQLKQPH266:HP0465       ELTHFIKFSQNALKQFLKEACLLLQMQLKQPHF32:HPF32_0867   ELTHFTNHSQNTLKQFLKKACLLLQMRLKQPHF30:HPF30_0857   ELTHFTNHSQNTLKQFLKKACLLLQMRLKQPH52:HPKB_0447     DLTRFTSHSQNTLKQFLKKACLLLQMRLKQPHF57:HPF57_0492   ELTHFINHSQNTLKQFLKKACLLLQMRLKQPH51:KHP_0429      ELTRFTNHSQNTLKQFLKKACLLLQMRLKQPHF16:HPF16_0445   ELTHFTNHSQNTLKQFLKKACLLLQMRLKQP
