# Supplementary material for: Evolution in an oncogenic bacterial species with extreme genome plasticity: Helicobacter pylori East Asian genomes
Source: BMC Microbiol. 2011 May 16;11:104. doi: 10.1186/1471-2180-11-104 (PMC3120642; doi:10.1186/1471-2180-11-104)
Supplement: Additional file 6 — Multiple sequence alignments of diverged genes. [file 1471-2180-11-104-S6.ZIP › Diverged_genes_multiple_seuence_alignments/HP0477_HP0923_hopJ.mfa.rtf]

                  1         11        21        31        41        51        61        71        81        91                          |         |         |         |         |         |         |         |         |         |         HB8:HPB8_1104     MQFPKTLFSLS----L----LF--LSYCIAEENGAYASVGFEYSISHAVEHNNPFLNQERIQTISNAQNKIYKLNQVKNEITNMQNTFNYINNALKNNSKHB38:HELPY_0461   MQFQKTLLSLS----L----LF--LSYCIAEENGAYASVGFEYSISHAVEHNNPFLNQERIQIISNAQNQIYKLNQVKNEITSMPNTFNYINNALKNNSKHB38:HELPY_0907   --MQKILFPLP----L----LF--LSYCIAEENGAYASVGFEYSISHAVEHNNPFLNQERIQIISNAQNQIYKLNQVKNEITSMPNTFNYINNALKNNSKHB8:HPB8_626      --MPKTLLHSS----FFLP-LF--LSFCIAEENGAYASVGFEYSISHAVEHNNPFLNQERIQIISNAQNKIYKLHQVKNEITNMPKTFAYINNALKNNSKH266:HP0923       MQFQKALLHSS----FFLP-LF--LSFCIAEENGAYASVGFEYSISHAVEHNNPFLNQERIQIISNAQNKIYKLHQVKNEITSMPKTFAYINNALKNNSKH266:HP0477       --MQKALLHSS----FFLP-LF--LSFCIAEENGAYASVGFEYSISHAVEHNNPFLNQERIQIISNAQNKIYKLHQVKNEITSMPKTFAYINNALKNNSKHP12:HPP12_0487   MQFPKTLLHSS----FFLP-LL--LSFCIAEENGAYASVGFEYSISHAIQHNDPFLNQERIQIISNAQNKIYKLNQVKNEITSMPNTFNYINNALKNNSKHP12:HPP12_0920   MQFPKTLLHSS----FFLP-LL--LSFCIAEENGAYASVGFEYSISHAIQHNDPFLNQERIQIISNAQNKIYKLNQVKNEITSMPNTFNYINNALKNNSKHG27:HPG27_435    --MQKALLHSS----FFLP-LF--LSYCIAEENGAYASVGFEYSISHAVEHNDPFLNQERIQTISNAQNKIYKLNQVKNEITNMQKTFNYINNALKNNSKHG27:HPG27_872    --MQKALLHSS----FFLP-LF--LSYCIAEENGAYASVGFEYSISHAVEHNDPFLNQERIQTISNAQNKIYKLNQVKNEITNMQKTFNYINNALKNNSKHSJM:HPSJM_04700  MQFPKALLHSS----FFLP-LF--LSYCIAEENGAYASVGFEYSISHAVEHNNPFLNQERIQTISNAQNQIYKLNQVKNEITNMPNTFNYINNALKNNSKHSJM:HPSJM_02390  MQFPKALLHSS----FFLP-LF--LSYCIAEENGAYASVGFEYSISHAVEHNNPFLNQERIQTISNAQNQIYKLNQVKNEITNMPNTFNYINNALKNNSKHHPA:HPAG1_0904   --MQKALLHSS----FFLPLLF--LSFCIAEENGAYASVGFEYSISHAVEHNNPFLNQERIQIISNVQNKIYKLNQVKNEITNMPNTFNYINNALKNHAKHHPA:HPAG1_0454   --MQKALLHSS----FFLPLLF--LSFCIAEENGAYASVGFEYSISHAVEHNNPFLNQERIQIISNVQNKIYKLNQVKNEITNMPNTFNYINNALKNHAKHF32:HPF32_0433   --MQKTLFSF-----LFLP-LFLSLSFCIAEENGAYASVGFEYSISHAIQHNNPFLNQERIKTISNAQNKIYKLNQVKNEITSMPNTFAYINNNLKNHSQHF32:HPF32_0854   --MQKTLFSF-----LFLP-LFLSLSFCIAEENGAYASVGFEYSISHAIQHNNPFLNQERIKTISNAQNKIYKLNQVKNEITSMPNTFAYINNNLKNHSQHF57:HPF57_0506   --MQKTLFSLSLFLSLFLP-LF--LSFCIAEENGVYASVGFEYSISHAIQHNNPFLNQERIQIISNAQNKIYKLNQVKNEITSMPKTFAYINNNLKNNSKHF57:HPF57_0932   --MQKTLFSLSLFLSLFLP-LF--LSFCIAEENGVYASVGFEYSISHAIQHNNPFLNQERIQIISNAQNKIYKLNQVKNEITSMPKTFAYINNNLKNNSKHF30:HPF30_0418   --MQKTLFSF-----LSLS-LF--LSFCIAEENGAYASVGFEYSISHAIQHNNPFLNQERIQIISDAQNKIYKLNQVKNEITSMPNTFAYINNNLKNNSKHF30:HPF30_0844   --MQKTLFSF-----LSLS-LF--LSFCIAEENGAYASVGFEYSISHAIQHNNPFLNQERIQIISDAQNKIYKLNQVKNEITSMPNTFAYINNNLKNNSKHF16:HPF16_0902   --MQKTLFSLS----LPLF-LF--LSCCIAEENGAYASVGFEYSISHAIQHNNPFLNQERIQIISNAQNKIYKLNQVKNEITSMPNTFAYINNNLKNHSQHF16:HPF16_0458   --MQKTLFSLS----LPLF-LF--LSCCIAEENGAYASVGFEYSISHAIQHNNPFLNQERIQIISNAQNKIYKLNQVKNEITSMPNTFAYINNNLKNHSQH51:KHP_0441      --MQKTLFSF-----LSLP-LS--LSFCIAEENGAYASVGFEYSISHAIQHNNPFLNQERIQTISNAQNKIYKLNQVKNEITSMQNTFNYINNALKNNAKH51:KHP_0861      --MQKTLFSF-----LSLP-LF--LSFCIAEENGAYASVGFEYSISHAIQHNNPFLNQERIQTISNAQNKIYKLNQVKNEITSMQNTFNYINNALKNNAKH52:HPKB_0458     --MQKTLFS------LFLP-LF--LSFCIAEENGAYASVGFEYSISHAIQHNNPFLNQERIQIISNAQNKIYKLNQVKNEIISMPKTFAYINNNLKNHSKH52:HPKB_0891     --MQKTLFS------LFLP-LF--LSFCIAEENGAYASVGFEYSISHAIQHNNPFLNQERIQIISNAQNKIYKLNQVKNEIISMPKTFAYINNNLKNHSK                  101       111       121       131       141       151       161       171       181       191                         |         |         |         |         |         |         |         |         |         |         HB8:HPB8_1104     LTPTEMQAEQYYLQSTLQNIEKIVTLSGGVASNPQLAQALEKMQEPITNPLEFEENLKNLEVQFAQSQNRMLSSLSSQIAAISNSLNALDPSSYSKNISSHB38:HELPY_0461   LTPTEMQAEQYYLQSTLQNIEKIVMLSGGVASNPKLAQALEKMQEPITNPLELAENLRNLELQFAQSQNRMLSSLSSQIAQISNSLNVLDPSSYSKNISSHB38:HELPY_0907   LTPTEMQAEQYYLQSTLQNIEKIVMLSGGVASNPKLAQALEKMQEPITNPLELAENLRNLELQFAQSQNRMLSSLSSQIAQISNSLNVLDPSSYSKNISSHB8:HPB8_626      LTPTEIQAEKYYLQSTFENIEKIVTLSGGVASNPKLVQALEKIQEPITNPLEFEENLKNLELQFNQSQNRTLSSLSSQIAAISNSLNALDPTSYSKNISSH266:HP0923       LTPTEMQAEQYYLQSTFQNIEKIVMLSGGVSSNPQLVQALEKMQEPITNPLEFEENLRNLEVQFAQSQNRMLSSLSSQIAAISNSLNALDPNSYSKNISSH266:HP0477       LTPTEMQAEQYYLQSTFQNIEKIVMLSGGVSSNPQLVQALEKMQEPITNPLEFEENLRNLEVQFAQSQNRMLSSLSSQIAAISNSLNALDPNSYSKNISSHP12:HPP12_0487   LTPTEMQAEQYYLQSTLQNIEKIVMLSGGVASNPKLVQALEKMQEPTTNPLEFEENLRNLEVQFAQSQNRMLSSLSSQIAAISNSLNALDPNSYSKNVSSHP12:HPP12_0920   LTPTEMQAEQYYLQSTLQNIEKIVMLSGGVASNPKLVQALEKMQEPTTNPLEFEENLRNLEVQFAQSQNRMLSSLSSQIAAISNSLNALDPNSYSKNVSSHG27:HPG27_435    LTPTEMQAEQYYLQSTLQNIEKIVTLSGGVASNPQLAQALEKMQEPITNPLELAERLKNLELQFAQSQNRMLSSLSSQIAAISNSLNALDPNSYSKNVSSHG27:HPG27_872    LTPTEMQAEQYYLQSTLQNIEKIVTLSGGVASNPQLAQALEKMQEPITNPLELAERLKNLELQFAQSQNRMLSSLSSQIAAISNSLNALDPNSYSKNVSSHSJM:HPSJM_04700  LTPTEIQAEQYYLQSTLEGIEKIVALSGGVASNPQLAQALEKMQEPATNPLELVENLRNLELQFAQSQNRMLSSLSSQIAQISNSLNALDPNSYSKNVSSHSJM:HPSJM_02390  LTPTEIQAEQYYLQSTLEGIEKIVALSGGVASNPQLAQALEKMQEPATNPLELVENLRNLELQFAQSQNRMLSSLSSQIAQISNSLNALDPNSYSKNVSSHHPA:HPAG1_0904   LTPTEKQAEQYYLQSTLEGIEKIVALSGGVASQPKLVQALEKMQEPITNPLELAENLKNLELQFSQSQNRMLSSLSSQIAQISNSLNALDPTSYSKNVSSHHPA:HPAG1_0454   LTPTEKQAEQYYLQSTLEGIEKIVALSGGVASQPKLVQALEKMQEPITNPLELAENLKNLELQFSQSQNRMLSSLSSQIAQISNSLNALDPTSYSKNVSSHF32:HPF32_0433   LTPTEMQAEKYYLQSTLQNIEKIVMLSGGVASNPQLAQALEKMQEPITNPLELAENLKNLEAQFAQSQNRMLSSLSSQIAQISNSLNALDPSSYSKNISNHF32:HPF32_0854   LTPTEMQAEKYYLQSTLQNIEKIVMLSGGVASNPQLAQALEKMQEPITNPLELAENLKNLEAQFAQSQNRMLSSLSSQIAQISNSLNALDPSSYSKNISNHF57:HPF57_0506   PSATEMQAEKYYLQSTFQNIEKIVMLSGGVASNPQLVQALEKIQEPTTNPLEFEENLKNLEVQFNQSQNRMLSSLSSQIATISNSLNALDPSSYSKNISNHF57:HPF57_0932   PSATEMQAEKYYLQSTFQNIEKIVMLSGGVASNPQLVQALEKIQEPTTNPLEFEENLKNLEVQFNQSQNRMLSSLSSQIATISNSLNALDPSSYSKNISNHF30:HPF30_0418   PSATEMQAEKYYLQSTFQNIEKIVMLSGGVASNPQLVQALEKIQEPTTNPLEFEENLKNLEVQFNQSQNRMLSSLSSQIAQISNSLNALDPSSYSKNISNHF30:HPF30_0844   PSATEMQAEKYYLQSTFQNIEKIVMLSGGVASNPQLVQALEKIQEPTTNPLEFEENLKNLEVQFNQSQNRMLSSLSSQIAQISNSLNALDPSSYSKNISNHF16:HPF16_0902   LTPTEMQAEKYYLQSTFQNIEKIIALSGGVASNPQLVQALEKIQEPTTNPLEFEENLKNLEVQFNQSQNRMLSSLSSQIAQISNSLNAFDPNSYSKNISNHF16:HPF16_0458   LTPTEMQAEKYYLQSTFQNIEKIIALSGGVASNPQLVQALEKIQEPTTNPLEFEENLKNLEVQFNQSQNRMLSSLSSQIAQISNSLNAFDPNSYSKNISNH51:KHP_0441      LTPTEMQAEQYYLQSTFQNIEKIITLSGGAASNPQLAQALEKMQEPTNSPLEFEENLKNLEVQFNQSQNRMLSSLSSQIAQISNSLNALDPSSYSKNISSH51:KHP_0861      LTPTEMQAEQYYLQSTFQNIEKIITLSGGAASNPQLAQALEKMQEPTNSPLEFEENLKNLEVQFNQSQNRMLSSLSSQIAQISNSLNALDPSSYSKNISSH52:HPKB_0458     LTPTEMQAEKYYLQSSFQNIEKIVMLSGGVASNPQLVQALEGMQKPTNSPLELAERLKNLEVQFNQSQNRMLSSLSSQIAQISNSLNALDPNSYSKNISNH52:HPKB_0891     LTPTEMQAEKYYLQSSFQNIEKIVMLSGGVASNPQLVQALEGMQKPTNSPLELAERLKNLEVQFNQSQNRMLSSLSSQIAQISNSLNALDPNSYSKNISN                  201       211       221       231       241       251       261       271       281       291                         |         |         |         |         |         |         |         |         |         |         HB8:HPB8_1104     MYGVSLSVGYKHFFTKKKNQGFRYYLFYDYGYTNFGFVGNGFDGLGKMNNHLYGLGIDYLYNFIDNAKKHSSVGFYVGFALAGSSWVGSGLGMWVSQTDFHB38:HELPY_0461   MYGVSLSVGYKHFFTKKKNQGLRYYLFYDYGYTNFGFVGNGFDGLGKMNNHLYGLGIDYLYNFIDNAQKHSSVGFYVGFALAGSSWVGSGLGMWVSQTDFHB38:HELPY_0907   MYGVSLSVGYKHFFTKKKNQGLRYYLFYDYGYTNFGFVGNGFDGLGKMNNHLYGLGIDYLYNFIDNAQKHSSVGFYVGFALAGSSWVGSGLGMWVSQTDFHB8:HPB8_626      MYGVSLSVGYKHFFTKKKNQGFRYYLFYDYGYTNFGFVGNGFDGLGKMNNHLYGLGIDYLYNFIDNSQKHSSVGFYVGFALAGSSWVGSGLGMWISQTDFH266:HP0923       MYGVSLSVGYKHFFTKKKNQGLRYYLFYDYGYTNFGFVGNGFDGLGKMNNHLYGLGIDYLYNFIDNAKKHSSVGFYLGFALAGSSWVGSGLSMWVSQTDFH266:HP0477       MYGVSLSVGYKHFFTKKKNQGLRYYLFYDYGYTNFGFVGNGFDGLGKMNNHLYGLGIDYLYNFIDNAKKHSSVGFYLGFALAGSSWVGSGLSMWVSQTDFHP12:HPP12_0487   MYGVSLSVGYKHFFTKKKNQGLRYYLFYDYGYTNFGFVGNGFDGLGKMNNHLYGLGIDYLYNFIDNSQKHSSVGFYAGFALAGSSWVGSGLGMWVSQMDFHP12:HPP12_0920   MYGVSLSVGYKHFFTKKKNQGLRYYLFYDYGYTNFGFVGNGFDGLGKMNNHLYGLGIDYLYNFIDNSQKHSSVGFYAGFALAGSSWVGSGLGMWVSQMDFHG27:HPG27_435    MYGVSLSVGYKHFFTKKKNQGLRYYLFYDYGYTNFGFVGNGFDGLGKMNNHLYGLGIDYLYNFIDNAKKHSSVGFYAGFALAGSSWVGSGLGMWVSQMGFHG27:HPG27_872    MYGVSLSVGYKHFFTKKKNQGLRYYLFYDYGYTNFGFVGNGFDGLGKMNNHLYGLGIDYLYNFIDNAKKHSSVGFYAGFALAGSSWVGSGLGMWVSQMGFHSJM:HPSJM_04700  MYGVSLSVGYKHFFTKKKNQGFRYYLFYDYGYTNFGFVGNGFDGLGKMNNHLYGLGIDYLYNFIDNEQKHSSVGFYLGFALAGSSWVGSGLSMWVSQRGFHSJM:HPSJM_02390  MYGVSLSVGYKHFFTKKKNQGFRYYLFYDYGYTNFGFVGNGFDGLGKMNNHLYGLGIDYLYNFIDNEQKHSSVGFYLGFALAGSSWVGSGLSMWVSQRGFHHPA:HPAG1_0904   MYGVSLSVGYKHFFTKKKNQGLRYYLFYDYGYTNFGFVGNGFDGLGKMNNHLYGLGIDYLFNFIDNAKKHSSVGFYAGFALAGSSWVGSGLGMWVSQTDFHHPA:HPAG1_0454   MYGVSLSVGYKHFFTKKKNQGLRYYLFYDYGYTNFGFVGNGFDGLGKMNNHLYGLGIDYLFNFIDNAKKHSSVGFYAGFALAGSSWVGSGLGMWVSQTDFHF32:HPF32_0433   MYGVTLNVGYKHFFTKKKNQGFRYYLFYDYGYTNFGFVGNGFDGLGKMNNHLYGLGIDYLYNFIDNAQKHSSVGFYAGFALAGSSWVGSGLSMWVSETDFHF32:HPF32_0854   MYGVTLNVGYKHFFTKKKNQGFRYYLFYDYGYTNFGFVGNGFDGLGKMNNHLYGLGIDYLYNFIDNAQKHSSVGFYAGFALAGSSWVGSGLSMWVSETDFHF57:HPF57_0506   MYGVTLNVGYKHFFTKKKNQGFRYYLFYDYGYTNFGFVGNGFDGLGKMNNHLYGLGIDYLYNFIDNSQKHSSVGFYAGFALAGSSWVGSGLSMWVSETDFHF57:HPF57_0932   MYGVTLNVGYKHFFTKKKNQGFRYYLFYDYGYTNFGFVGNGFDGLGKMNNHLYGLGIDYLYNFIDNSQKHSSVGFYAGFALAGSSWVGSGLSMWVSETDFHF30:HPF30_0418   MYGVTLNVGYKHFFTKKKNQGFRYYLFYDYGYTNFGFVGNGFDGLGKMNNHLYGLGIDYLYNFIDNAQKHSSVGFYAGFALAGSSWVGSGLSMWVSETDFHF30:HPF30_0844   MYGVALNVGYKHFFTKKKNQGFRYYLFYDYGYTNFGFVGNGFDGLGKMNNHLYGLGIDYLYNFIDNAQKHSSVGFYAGFALAGSSWVGSGLSMWVSETDFHF16:HPF16_0902   MYGVTLNVGYKHFFTKKKNQGFRYYLFYDYGYTNFGFVGNGFDGLGKMNNHLYGLGIDYLYNFIDNAQKHSSVGFYAGFALAGSSWVGSGLSMWVSETDFHF16:HPF16_0458   MYGVTLNVGYKHFFTKKKNQGFRYYLFYDYGYTNFGFVGNGFDGLGKMNNHLYGLGIDYLYNFIDNAQKHSSVGFYAGFALAGSSWVGSGLSMWVSETDFH51:KHP_0441      MYGVTLNVGYKHFFTKKKNQGFRYYLFYDYGYTNFGFVGNGFDGLGKMNNHLYGLGIDYLYNFIDNSQKHSSVGFYAGFALAGSSWVGSGLSMWVSETDFH51:KHP_0861      MYGVTLNVGYKHFFTKKKNQGFRYYLFYDYGYTNFGFVGNGFDGLGKMNNHLYGLGIDYLYNFIDNSQKHSSVGFYAGFALAGSSWVGSGLSMWVSETDFH52:HPKB_0458     MYGVTLNVGYKHFFTKKKNQGFRYYLFYDYGYTNFGFVGNGFDGLGKMNNHLYGLGIDYLYNFIDNSQKHSSMGFYAGFALAGSSWVGSGLGMWVSETDFH52:HPKB_0891     MYGVTLNVGYKHFFTKKKNQGFRYYLFYDYGYTNFGFVGNGFDGLGKMNNHLYGLGIDYLYNFIDNSQKHSSMGFYAGFALAGSSWVGSGLGMWVSETDF                  301       311       321       331       341       351       361       371                  |         |         |         |         |         |         |         |HB8:HPB8_1104     INNYLTGYQAKMHTSFFQIPLNFGVRVNVNRHNGFEMGLKIPLAVNSFYETHGKGLNTSLFFKRLVVFNVSYVYSFHB38:HELPY_0461   INNYLTGYQAKMHTSFFQIPLNFGVRVNVNRHNGFEMGLKIPLAVNSFYETHGKGLNTSLFFKRLVVFNVSYVYNFHB38:HELPY_0907   INNYLTGYQAKMHTSFFQIPLNFGVRVNVNRHNGFEMGLKIPLAVNSFYETHGKGLNTSLFFKRLVVFNVSYVYSFHB8:HPB8_626      INNYLINYQAKMHTSFFQIPLNFGVRVNVNRHNGFEMGLKIPLAVNSFYETHGKGLNTSLFFKRLVVFNVSYVYSFH266:HP0923       INNYLTGYQAKMHTSFFQIPLNFGVRVNVNRHNGFEMGLKIPLAMNSFYETHGKGLNTSLFFKRLVMFNVSYVYSFH266:HP0477       INNYLTGYQAKMHTSFFQIPLNFGVRVNVNRHNGFEMGLKIPLAMNSFYETHGKGLNTSLFFKRLVMFNVSYVYSFHP12:HPP12_0487   INNYLTGYQAKMHTSFFQIPLNFGVRVNVNRHNGFEMGLKIPLAVNSFYETHGKGLNTSLFFKRLVVFNVSYVYSFHP12:HPP12_0920   INNYLTGYQAKMHTSFFQIPLNFGVRVNVNRHNGFEMGLKIPLAVNSFYETHGKGLNTSLFFKRLVVFNVSYVYSFHG27:HPG27_435    INNYLTDYQAKMHTSFFQIPLNFGVRVNVNRHNGFEMGLKIPLAVNSFYETHGKGLNASLFFKRLVMFNVSYVYSFHG27:HPG27_872    INNYLTDYQAKMHTSFFQIPLNFGVRVNVNRHNGFEMGLKIPLAVNSFYETHGKGLNASLFFKRLVMFNVSYVYSFHSJM:HPSJM_04700  INNYLTGYQAKMHTSFFQIPLNFGVRVNVNRHNGFEMGLKIPLAVNSFYETHGKGLNTSLFFKRLVVFNVSYVYSFHSJM:HPSJM_02390  INNYLTGYQAKMHTSFFQIPLNFGVRVNVNRHNGFEMGLKIPLAVNSFYETHGKGLNTSLFFKRLVVFNVSYVYSFHHPA:HPAG1_0904   INNYLINYQAKMHTSFFQIPLNFGVRVNVNRHNGFEMGLKIPLAVNSFYETHGKGLNTSLFFKRLVAFNVSYVYSFHHPA:HPAG1_0454   INNYLINYQAKMHTSFFQIPLNFGVRVNVNRHNGFEMGLKIPLAVNSFYETHGKGLNTSLFFKRLVAFNVSYVYSFHF32:HPF32_0433   INNYLTGYQAKMHTSFFQIPLNFGVRVNVNRHNGFEMGLKIPLAVNSFYETHGKGLNTSLFFKRLVVFNVSYVYSFHF32:HPF32_0854   INNYLTGYQAKMHTSFFQIPLNFGVRVNVNRHNGFEMGLKIPLAVNSFYETHGKGLNTSLFFKRLVVFNVSYVYSFHF57:HPF57_0506   INNYLTGYQAKMHTSFFQIPLNFGVRVNVNRHNGFEMGLKIPLAVDSFYETHGKGLNTSLFFKRLVVFNVSYVYSFHF57:HPF57_0932   INNYLTGYQAKMHTSFFQIPLNFGVRVNVNRHNGFEMGLKIPLAVDSFYETHGKGLNTSLFFKRLVVFNVSYVYSFHF30:HPF30_0418   INNYLTGYQAKMHTSFFQIPLNFGVRVNVNRHNGFEMGLKIPLAVNSFYETHGKGLNTSLFFKRLVVFNVSYVYSFHF30:HPF30_0844   INNYLTGYQAKMHTSFFQIPLNFGVRVNVNRHNGFEMGLKIPLAVNSFYETHGKGLNTSLFFKRLVVFNVSYVYSFHF16:HPF16_0902   INHYLTGYQAKMHTSFFQIPLNFGVRVNVNRHNGFEMGLKIPLAVNSFYETHGKGLNTSLFFKRLVVFNVSYVYSFHF16:HPF16_0458   INHYLTGYQAKMHTSFFQIPLNFGVRVNVNRHNGFEMGLKIPLAVNSFYETHGKGLNTSLFFKRLVVFNVSYVYSFH51:KHP_0441      INHYLMGYQAKMHTSFFQIPLNFGVRVNVDRHNGFEMGLKIPLAVNSFYETHGKGLNTSLFFKRLVVFNVSYVYSFH51:KHP_0861      INHYLTGYQAKMHTSFFQIPLNFGVRVNVDRHNGFEMGLKIPLAVNSFYETHGKGLNTSLFFKRLVVFNVSYVYSFH52:HPKB_0458     INHYLTGYQAKMHTSFFQIPLNFGVRVNVNRHNGFEMGLKIPLA--------GEFL-------------------LH52:HPKB_0891     INHYLTGYQAKMHTSFFQIPLNFGVRVNVNRHNGFEMGLKIPLAVNSFYETHGKGLNTSLFFKRLVVFNVSYVYSF
