# Supplementary material for: Evolution in an oncogenic bacterial species with extreme genome plasticity: Helicobacter pylori East Asian genomes
Source: BMC Microbiol. 2011 May 16;11:104. doi: 10.1186/1471-2180-11-104 (PMC3120642; doi:10.1186/1471-2180-11-104)
Supplement: Additional file 6 — Multiple sequence alignments of diverged genes. [file 1471-2180-11-104-S6.ZIP › Diverged_genes_multiple_seuence_alignments/HP0492_hpaA.mfa.rtf]

                  1         11        21        31        41        51        61        71        81        91                          |         |         |         |         |         |         |         |         |         |         HB8:HPB8_677      MERSL---IFKKVRIYSKMLVALGLSSVLIGCAMNPSAETKTPNNAKNQ--VPTHERIQTSSEHVTPLDFNYPVHIVQAPQNHHVVGILAPRIQVSDNLKHB38:HELPY_0861   MERSL---IFKKVRVYSKMLVALGLSSVLIGCAMNPSAETKTPNDAKNQ--VQTQERIQTSSEHVTPLDFNYPIHIAQAPQNHHVVGILAPRIQVSDNLKHHPA:HPAG1_0468   MERSL---IFKKVRVYSKMLVALGLSSVLIGCAMNPSAETKKPNDAKNQ--VQTHERIQTSSEHVTPLDFNYPIHIVQAPQNHHVVGILMPRIQVSDNLKHP12:HPP12_0500   MERSF---IFKKVRVYSKMLVALGLSGVLIGCAMNPSAETKTPNNAKNQ--VQTHERIQTSSEHVTPLDFNYPIHIAQAPQNHHIVGILAPRIQVSDNLKHG27:HPG27_452    MERSL---IFKKVRVYSKMLVALGLSSVLIGCAMNPSAETKTPNDAKNQ--VQTHERMKTSSEYVTPLDFNYPIHIAQAPQNHHIVGILAPRIQVSDNLKH266:HP0492       MERSL---IFKKVRIYSKMLVALGLSSVLIGCAMNPSAETKTPNDAKNQ--VQTHERMKTSSEHVTPLDFNYPIHIVQAPQNHHVVGILTPRIQVSDNLKHSJM:HPSJM_02465  MNCFFFKRLFFSQKLCFKTLIALGLSSVLVGCTMNPSAETKTQNDAKNQQPVQTHERMKTSSEHVMPLDFNYPIHIVQAPQNHHIVGILAPHIQVSDNLKHF32:HPF32_0473   MNCFFFKRLFFSQRLCFKTLIALGLSSVLVGCAIKPVAEVKPQNQQEKP--IQINEKIQT-TQKVTPFNFNYSLHVAQAPQNYRLIGILAPRIQVSDNLKHF30:HPF30_0829   MNCFFFKRLFFSQRLCFKTLISLGISSVLVGCTIKPVAEVKPQNQQEKP--IQVNEKIQT-TQKVTPFNFNYSLHVAQAAQNYRLIGILAPRIQVSDNLKHF16:HPF16_0861   MNCFFFKRLFFPQRLYFKTLIALGISSVLVGCAIKPVAEVKPQNQQEKP--IQVNEKIQT-TQKVTPFNFNYSLHVAQAAQNYRLIGILAPRIQVSDNLKH51:KHP_0824      MNCFFFKRLFFSQRLCFKTLISLGISSVLVGCAIKPVAEVKPQNQQKKP--IQVNEKIQT-TQKVTPFNFNYSLHVAQAPQNYRLIGILAPRIQVSDNLKH52:HPKB_0845     MNCFFFKRLFFSQRLCFKTLIALGLSSVLVGCAIKPVAEVKPQNQQEKP--IQVNEKIQT-TQKVTPFNFNYSLHVAQAPQNYRLIGILAPRIQVSDNLK                  101       111       121       131       141       151       161       171       181       191                         |         |         |         |         |         |         |         |         |         |         HB8:HPB8_677      PYIDKFQDALVNQIQTIFEKRGYQVLRFQDEKALNAQDKRKIFSVLDLKGWVGILEDLKMNLKDPNNPNLDTLVDQSSGSVWFNFYEPESNRVVHDFAVEHB38:HELPY_0861   PYIDKFQDALANQIQTIFEKRGYQVLRFQDEKALNEQDKKKIFSVLDLKGWVGILEDLKMNVKDPNNPNLDVLVDQSSGSVWFNFYEPESNRIVHDFAVEHHPA:HPAG1_0468   PYIDKFQDALANQIQTIFEKRGYQVLRFQDEKALSTQDKRKIFSVLDLKGWVGILEDLKMNVKDPNNPNLDTLVDQSSGSVWFNFYEPESNRVVHDFAVEHP12:HPP12_0500   PYIDKFQDALANQIQTIFEKRGYQVLRFQDEKALNAQDKRKIFCVLDLRGWVGILEDLKMNVKDPNDPNLDVLVDQSSGSVWFNFYDPESNRVVHDFAVEHG27:HPG27_452    PYIDKFQDALVNQIQTIFEKRGYQVLRFQDEKALNAQDKRKIFCVLDLKGWVGILEDLKMNLKDSNNPNLDTLVDQSSGSVWFNFYEPESNRVVHDFAVEH266:HP0492       PYIDKFQDALINQIQTIFEKRGYQVLRFQDEKALNAQDKRKIFSVLDLKGWVGILEDLKMNLKDPNNPNLDTLVDQSSGSVWFNFYEPESNRVVHDFAVEHSJM:HPSJM_02465  PYIDKFQDALANQIQTIFEKRGYQVLRFQDEKALSAQDKRKIFSVLDVKGWVGILEDLKMNLKDPNNPNLDALVDQSSGSVWFSFYEPESNRVVHDFAVEHF32:HPF32_0473   PYIDKFQDALINQIQTIFEKRGYQTLFFKDESALTLQDKRKLFAVLDVKGWVGVLEDLKMNLKDPNNPNLGTLVDQSSGSVWFSFYEPESNRVVHDFAVEHF30:HPF30_0829   PYIDKFQDALINQIQTIFEKRGYQTLFFKDESALTLQDKRKLFAVLDVKGWVGVLEDLKVNLKDPNNPNLGTLVDQSSGSVWFSFYEPESNRVVHDFAVEHF16:HPF16_0861   PYIDKFQDALINQIQTIFEKRGYQTLFFKDESALTLQDKRKLFAVLDVKGWVGVLEDLKMNLKDPNNPNLGTLVDQSSGSVWFSFYEPESNRVVHDFAVEH51:KHP_0824      PYIDKFQDALIDQIQTIFEKRGYQTLFFKDESALTLQDKRKLFAVLDVKGWVGVLEDLKMNLKDPNNPNLGTLVDQSSGSVWFSFYEPESNRVVHDFAVEH52:HPKB_0845     PYIDKFQNALINQIQTIFEKRGYQTLFFKDESALTLQDKRKLFAVLDVKGWVGVLEDLKMNLKDPNNPNLGTLVDQSSGSVWFSFYEPESNRVVHDFAVE                  201       211       221       231       241       251       261       271       281                  |         |         |         |         |         |         |         |         |HB8:HPB8_677      VGTFQAM--TYTYKHSNSGGLNSSNSIIHEDLEKNKEDAIHKILNRMYAVVMKKAVMELTEENIAKYRDAIDRMKGFKSSMPQKK--HB38:HELPY_0861   VGTFQAM--TYTYKHSNSGGFDSSNSIIHEDLEKNKEDAIHKILNRMYAVVMKKAVTELTEENIAKYRDAIDRMKGFKSSMPQKK--HHPA:HPAG1_0468   VGTFQAM--TYTYKHSNSGGFDSSNSIIHENLEKNKEDAIHKILNRMYAVVMKKAVTELTKENIAKYRDTIDRMKGFKSSMPQKK--HP12:HPP12_0500   VGTFQAM--TYTYKHSNSGGFDSSNSIIHEDLEKNKEDAIHKILNRMYAVVMKKAVTELTEENIAKYRDAIDRMKGFKSSMSQKK--HG27:HPG27_452    VGTFQAM--TYTYKHSNSGGFDSSNSIIHEDLEKNKEDAIHKILNRMYAVVMKKAVTELTEENIAKYRDAIDRMKGFKSSMPQKK--H266:HP0492       VGTFQAM--TYTYKHNNSGGLNSSNSIIHEYLEKNKEDAIHKILNRMYAVVMKKAVTELTKENIDKYREAIDRMKGFKSSMPQKK--HSJM:HPSJM_02465  VGTFQAM--TYTYKQSNSGGLNSSNSIIHEDLEKNKEDAIHQILNKIYALIMKKAVTELTEKNISQYKEAIDRMKGFKSSMPQKK--HF32:HPF32_0473   VGTFQAI--TYTYKQSNSGGFNSSNSIIHEDLEKNKEDAIHQILNKIYALIMKKAVTELTEKNISQYKEAIDRMKGFKSSMPQKK--HF30:HPF30_0829   VGTFQAI--TYTYKQSNSGGFNSSNSIIHEDLEKNKEDAIHQILNKIYALIMKKAVTELTEKNISQYKEAIDRMKGFKSSMPQKK--HF16:HPF16_0861   VGTFQAITYTYTYKQSNSGGFNSSNSIIHEDLEKNKEDAIHQILNKIYALIMKKAVTELTEKNISQYKEAFDRMKGFKSSMPQKK--H51:KHP_0824      VGTFQAI--THTYKQSNSGGFNSSNSIIHEDLEKNKQDAIHQILNKIYALIMKKAVTELTEKNISQYKETIDRMKGFKTPTPQKSSSH52:HPKB_0845     VGTFQAM--TYTYKQSNSGGFNSSNSIIHEDLEKNKEDAIHQILNKIYALIMKKAVTELTEKNISQHKETIDRMKGFKTPTPQKSSS
