# Supplementary material for: Evolution in an oncogenic bacterial species with extreme genome plasticity: Helicobacter pylori East Asian genomes
Source: BMC Microbiol. 2011 May 16;11:104. doi: 10.1186/1471-2180-11-104 (PMC3120642; doi:10.1186/1471-2180-11-104)
Supplement: Additional file 6 — Multiple sequence alignments of diverged genes. [file 1471-2180-11-104-S6.ZIP › Diverged_genes_multiple_seuence_alignments/HP0547_cagA.mfa.rtf]

                  1         11        21        31        41        51        61        71        81        91                          |         |         |         |         |         |         |         |         |         |         HB8:HPB8_741      MTNETINQ-----QPQTEAAFNPQQFINNLQVAFIKVDNAVASFDPDQKPIVDKNDRDNRQAFEKISQLREKFANKAIKNPTKKNQYFSNFISKSSDLINHSJM:HPSJM_02765  MTNETIDQ-----QPQTEAAFNPQQFINNLQVAFLKLDNAVASFDPDQKPIVDKNDRDNRQAFDGISQLREEYSNKAIKNPTKKNQYFSDFINKSNDLINHP12:HPP12_0555   MTNETINQ-----QPQTEAAFNPQQFINNLQVAFLKVNNAVASYDPDQKPIVDKNDRDNRQAFDGISQLKEEYSNKAIKNPTKKNQYFSDFINKSNDLINHG27:HPG27_507    MTNETINQ-----QPQTEAAFNPQQFINNLQVAFLKVDNAVASYDPDQKPIVDKNDRDNRQAFNGISQLREEYSNKAIKNPAKKNQYFSDFIDKSNNLINH266:HP0547       MTNETIDQTRTPDQTQSQTAFDPQQFINNLQVAFIKVDNVVASFDPDQKPIVDKNDRDNRQAFDGISQLREEYSNKAIKNPTKKNQYFSDFIDKSNDLINHHPA:HPAG1_0524   MTNETINQ-----QPQTEAAFNPQQFINNLQVAFLKVDNAVVSYDPDQKPIVDKNDRDNRQAFDGISQLREEYSNKAIKNPTKKNQYFSDFINKSNDLINHF32:HPF32_0523   MTNETIDQTT----TPDQTGFVPQRFINNLQVAFIKVDNAVASFDPDQKPIVDKNDKDNRQAFEKISQLREEYANKAIKNPAKKNQYFSDFINKSNDLINHF16:HPF16_0811   MTNETIDQTT----TPDQTGFVPQRFINNLQVAFIKVDNAVASFDPDQKSIVDKNDKDNRQAFEKISQLREEYANKAIKNPAKKNQYFSDFINKSNDLINHF57:HPF57_0574   MTNETIDQ------TPNQTDFVPQRFINNLQVAFIKVDNAVASFDPDQKPIVDKNDRDNRQAFERISQLREEYANKAIKNPAKKNQYFSDFINKSNDLINHF30:HPF30_0779   MTNETIDQTITPDQTPNQTDFVPQRFINNLQVAFIKVDNAVASFDPDQKPIVDKNDRDNRQAFEKISQLREEYANKAIKNPTKKNQYFSDFINKSNDLINH51:KHP_0771      MTNETIDQTTTPDQTLNQTDFVPQRFIHNLQVAFIKVDNAVASFDPDQKPIVDKNDRDNRQAFEKISQLREEYANKAIKNPAKKNQYFSDFINKSNDLINH52:HPKB_0795     MTNEIIDQTT----TPNQTDFVPQRFINNLQVAFLKVDNAVASFDPDQKPIVDKNDRDNRQAFEKISQLKEEYANKAIKNPTKKNQYFSDFINKSNDLIN                  101       111       121       131       141       151       161       171       181       191                         |         |         |         |         |         |         |         |         |         |         HB8:HPB8_741      KDGLIDTGSSIKSFQKFGTQRYQIFMNWVSHQKDPSQINTQKIRGFMENIIQPPISDDKEKAEFLRSAKQALAGIIIRNQIRSDQKFMGVFDESLKERQEHSJM:HPSJM_02765  KDALIDVESSTKSFQKFGDQRYRIFTSWVSHQNDPSKINTRSIRNFMEHTIQPPIPDDKEKAEFLKSAKQSFAGIIIGNQIRTDQKFMGVFDESLKERQEHP12:HPP12_0555   KDNLIDVESSTKSFQKFGDQRYQIFTSWVSHQNDPSKINTRSIRNFMENIIQPPIPDDKEKAEFLKSAKQSFAGIIIGNQIRTDQKFMGVFDESLKERQEHG27:HPG27_507    KDALIDVESSTKSFQKFGDQRYQIFTSWVSHQNDPSKINTRSIRNFMENIIQPPIPDDKEKAEFLKSAKQSFAGIIIGNQIRTDQKFMGVFDESLKERQEH266:HP0547       KDNLIDVESSTKSFQKFGDQRYQIFTSWVSHQKDPSKINTRSIRNFMENIIQPPIPDDKEKAEFLKSAKQSFAGIIIGNQIRTDQKFMGVFDESLKERQEHHPA:HPAG1_0524   KDNLIDVESSTKSFQKFGDQRYRIFTSWVSHQNDPSKINTRSIRNFMENIIQPPIPDDKEKAEFLKSAKQSFAGIIIGNQIRTDQKFMGVFDEFLKERQEHF32:HPF32_0523   KDNLIAVDSSVESFRKFGDQRYQIFTSWVSLQKDPSKINTQQIRNFMENVIQPPISDDKEKAEFLRSAKQSFAGIIIGNQIRSDEKFMGVFDESLKARQEHF16:HPF16_0811   KDNLIAVDSSVESFRKFGDQRYQIFTSWVSLQKDPSKINTQQIRNFMENVIQPPISDDKEKAEFLRSAKQSFAGIIIGNQIRSDEKFMGVFDESLKARQEHF57:HPF57_0574   KDNLIAVDSSVESFRKFGDQRYQIFTSWVSLQKDPSKINTQQIRNFMENIIQPPISDDKEKAEFLRSAKQSFAGIIIGNQIRSDEKFMGVFDESLKARQEHF30:HPF30_0779   KDNLIAVDSSVESFRKFGDQRYQIFTSWVSLQKDPSKINTQQIRNFMENIIQPPISDDKEKAEFLRSAKQSFAGIIIGNQIRSDEKFMGVFDESLKARQEH51:KHP_0771      KDNLIAVDSSVDSFRKFGDQRYQIFTSWVSLQKDPSKINTQQIRNFMENIIQPPISDDKEKAEFLRSAKQSFAGIIIGNQIRSDEKFMGVFDESLKARQEH52:HPKB_0795     KDNLIAVDSSVESFRKFGDQRYQIFMNWVSHQKDPSKINTQQIRNFMENIIQPPISDDKEKAEFLRSAKQSFAGIIIGNQIRSDQKFMGVFDESLKERQE                  201       211       221       231       241       251       261       271       281       291                         |         |         |         |         |         |         |         |         |         |         HB8:HPB8_741      AEKNGEPNGDPTGGDWFDIFLSFVFNKKQSSDLKETLNQEPVPHVQPDVATTTTDIQSLPPESRDLLDERGNFSKFTLGDMEMLDVEGVADIDPNYKFNQHSJM:HPSJM_02765  AEK----NGGPSGGDWLDIFLSFIFDKKQSSDVKEAINQEPVPHVQPDIATTTTDIQGLPPESRDLLDERGNFSKFTLGDMNMLDVEGVADIDPNYKFNQHP12:HPP12_0555   AEK----NGEPTGGDWLDIFLSFIFDKKQSSDVKEAINQEPVPHVQPDIATTTTDIQGLPPEARDLLDERGNFSKFTLGDMEMLDVEGVADIDPNYKFNQHG27:HPG27_507    AEK----NGGPTGGDWLDIFLSFIFDKKQSSDVKEAINQEPVPHVQPDIATTTTDIQGLPPEARDLLDERGNFSKFTLGDMEMLDVEGVADIDPNYKFNQH266:HP0547       AEK----NGGPTGGDWLDIFLSFIFNKKQSSDVKEAINQEPVPHVQPDIATTTTDIQGLPPEARDLLDERGNFSKFTLGDMEMLDVEGVADIDPNYKFNQHHPA:HPAG1_0524   AEK----NGGPTGGDWLDIFLSFIFDKKQSSDVKEAINQEPVPHVQPDIATTTTDIQGLPPESRDLLDERGNFSKFTLGDMEMLDVEGVADIDPNYKFNQHF32:HPF32_0523   AEK----NAEPAGGDWLDIFLSFVFNKKQSSDLKETLNQEPRPDFEQNLATTTTDIQGLPPEARDLLDERGNFFKFTLGDVEMLDVEGVADKDPNYKFNQHF16:HPF16_0811   AEK----NAEPAGGDWLDIFLSFVFNKKQSSDLKETLNQEPRPDFEQNLATTTTDIQGLPPEARDLLDERGNFFKFTLGDVEMLDVEGVADKDPNYKFNQHF57:HPF57_0574   AEK----NAEPAGGDWLDIFLSFVFNKKQSSDLKETLNQEPRPDFEQNLSTTTTDIQGLPPEARDLLDERGNFFKFTLGDVEMLDVEGVADKDPNYKFNQHF30:HPF30_0779   AEK----NAEPAGGDWLDIFLSFVFNKKQSSDLKETLNQEPRPDFEQNLATTTTDIQGLPPESRDLLDERGNFFKFTLGDMEMLDVEGVADKDPNYKFNQH51:KHP_0771      AEK----NAEPAGGDWLDIFLSFVFNKKQSSDLKETLNQEPRPDFEQNLATTTTDIQGLPPEARDLLDERGNFSKFTLGDMEMLDVEGVADKDPNYKFNQH52:HPKB_0795     AEK----NGEPAGGDWLDIFLSFVFNKKQSSDLKETLNQEPRPDFEQNLATTTTNIQGLPPESRDLLDERGNFFKFTLGDVEMLDVEGVADKDPNYKFNQ                  301       311       321       331       341       351       361       371       381       391                         |         |         |         |         |         |         |         |         |         |         HB8:HPB8_741      LLVHNNALSSVLMGSHDGIEPEKVSLLYGNNGGPEARHDWNATVGHKNQQGNNVATLINVHMKNGSGLVIAGGEKGVNNPSFYLYKEDQLTGLKQALSQKHSJM:HPSJM_02765  LLIHNNALSSVLMGSHNGIEPEKVSLLYAGNGGFGAKHDWNATVGYKDQQGNNVATIINVHMKNGSGLVIAGGEKGINNPSFYLYKEDQLTGSQRALSQEHP12:HPP12_0555   LLIHNNTLSSVLMGSHDGIEPEKVSLLYAGNGGFGAKHDWNATVGYKDQQGNNVATIINVHMKNGSGLVIAGGEKGINNPSFYLYKEDQLTGSQRALSQEHG27:HPG27_507    LLIHNNALSSVLMGSHNGIEPEKVSLLYGGNGGPKAKHDWNATVGYKDQQGNNVATIINVHMKNGSGLVIAGGEKGINNPSFYLYKEDQLTGSQRALSQEH266:HP0547       LLIHNNALSSVLMGSHNGIEPEKVSLLYAGNGGFGDKHDWNATVGYKDQQGNNVATLINVHMKNGSGLVIAGGEKGINNPSFYLYKEDQLTGSQRALSQEHHPA:HPAG1_0524   LLIHNNALSSVLMGSHDGIEPEKVSLLYGNNGGPEARHDWNATVGYKNQQGDNVATLINVHMKNGSGLVIAGGEKGVNNPSFYLYKEDQLTGLKQALSQEHF32:HPF32_0523   LLIHNNALSSMLMGSHSNIEPEKVSLLYGDNGGPEARHDWNATVGYKNQQGNNVATLINAHLNNGSGLIIAGNEDGIKNPSFYLYKEDQLTGLKQALSQEHF16:HPF16_0811   LLIHNNALSSMLMGSHSNIEPEKVSLLYGDNGGPEARHDWNATVGYKDQQGNNVATLINAHLNNDSGLIIAGNEDGIKNPSFYLYKEDQLTGLKQAMSQEHF57:HPF57_0574   LLIHNNALSSVLMGGHSSIEPEKVSLLYGDNGGPEARHDWNATVGYKNQQGSNVATLINAHLNNGSGLIIAGNENGIKNPSFYLYKEDQLTGLKQALSQEHF30:HPF30_0779   LLIHNNALSSVLMGGHSNIEPEKVSLLYGDNGGPEARHDWNATVGYKNQQGNNVATLINAHLRNGSGLVIVGNEDGIKNPSFYLYKEDQLTGLKQALSQEH51:KHP_0771      LLIHNNALSSVLMGGHSNIEPEKVSLLYGDNGGPEARHDWNATVGYKNQQGNNVATLINAHLNNGSGLIIAGNEDGIKNPSFYLYKEDQLTGLKQAMSQEH52:HPKB_0795     LLIHNNALSSVLMGGHSNIEPEKVSLLYGGNGGPEARHDWNATVGYKNQQGNNVATLINAHLNNGSGLIIAGNENGIKNPSFYLYKEDQLTGLKQALSQE                  401       411       421       431       441       451       461       471       481       491                         |         |         |         |         |         |         |         |         |         |         HB8:HPB8_741      EIQNKVDFMEFLAQNNAKLDNLSKKEKEKFQNEIEDFQKDHKAYLDALGNDHIAFVSKKDKKHLALVTEFGSGDLSYTLKDYGKKADKALDREIKTTLQGHSJM:HPSJM_02765  EIRNKIDFMEFLAQNNAKLDNLSEKEKEKFRNEIKDFQKDSKAYLDALGNDRIAFVSKKDPKHSALITEFNKGDLSYTLKDYGKKADKALDREKNVTLQGHP12:HPP12_0555   EIRNKIDFMELLAQNNAKLDNLSEKEKEKFQNEIKDFQKDSKAYLDALGNDRIAFVSKKDTKHSALITEFGNGDLSYTLKDYGKKADKALDREKNVTLQGHG27:HPG27_507    EIRNKVDFMEFLAQNNAKLDNLSEKEEEKFRNEIKDFQKDSKAYLDALGNDRIAFVSKKDTKHSALITEFGNGDLSYTLKDYGKKADKALDREKNVTLQGH266:HP0547       EIRNKVDFMEFLAQNNTKLDNLSEKEKEKFQNEIEDFQKDSKAYLDALGNDRIAFVSKKDTKHSALITEFNNGDLSYTLKDYGKKADKALDREKNVTLQGHHPA:HPAG1_0524   EIRNKVDFMEFLAQNNAKLDNLSEKEKEKFQTEIKDFQKDSKAYLDALGNDRIAFVSKKDTKHSALLTEFGNGDLSYTLKDYGKKADKALDREKNVTLQGHF32:HPF32_0523   EIQNKVDFMEFLAQNNAKLDNLSEKEKEKFQTEIENFQKDRKAYLDALGNDHIAFVSKKDPKHLALVTEFGNGELSYTLKDYGKKQDKALDGETKTTLQGHF16:HPF16_0811   EIQNKVDFMEFLAQNNAKLDNLSEKEKEKFQAEIENFQKDRKAYLDALGNDHIAFVSKKDPKHLALVTEFGNGEVSYTLKDYGKKQDKALDGETKTTLQGHF57:HPF57_0574   EIQNKVDFMEFLAQNNAKLDNLSEKEKEKFQAEIENFQKDRKAYLDALGNDHIAFVSKKDPKHLALVTEFGNGEVSYTLKDYGKKQDKALDGETKTTLQGHF30:HPF30_0779   EIQNKVDFMEFLAQNNAKLDNLSEKEKEKFQTEIEDFQKDRKAYLDALGNDRIAFVSKKDPKHLALVTEFGNGEVSYTLKDYGKKQDKALDGETKTTLQGH51:KHP_0771      EIQNKVDFMEFLAQNNAKLDNLSEKEKEKFQTEIENFQKDRKAYLDALGNDRIAFVSKKDPKHLALVTEFGNGEVSYTLKDYGKKQDKALDGETKTTLQGH52:HPKB_0795     EIQNKVDFMEFLVQNNAKLDNLSEKEKEKFQTEIENFQKDRKAYLDALGNDHIAFVSKKDPKHLALVTEFGNGEVSYTLKDYGKKQDKALDGETKTTLQG                  501       511       521       531       541       551       561       571       581       591                         |         |         |         |         |         |         |         |         |         |         HB8:HPB8_741      SLKHDGVMFVDYSNFKYTNASKSPDKGVGATNGVSHLEANLSKVAVFNLPNLNNLAITSYIRRDLEEKLGAKGLSPQEANKLIKDFLNSNKELVGKVLNLHSJM:HPSJM_02765  NLKHDGVMFVDYSNFKYTNASKNPNKGVGATNGVSHLDAGFSKVAVFNLPDLNNLAINSFVRRNLEDKLVAKGLPPQEANKLIKDFLSSNKELVGKALNFHP12:HPP12_0555   SLKHDGVMFVDYSNFKYTNASKNPNKGVGVTNGVSHLEAGFDKVAVFNLPDLNNLAITSFVRRNLEDKLVTKGLSIQEANKLIKDFLNSNKELVGKALNFHG27:HPG27_507    NLKHDGVMFVDYSNFKYTNASKNPNKGVGVTNGVSHLEAGFSKVAVFNLPDLNNLAITSLVRRDLEDKLIAKGLSPQETNKLVKDFLSSNKELVGKALNFH266:HP0547       SLKHDGVMFVDYSNFKYTNASKNPNKGVGATNGVSHLEAGFNKVAVFNLPDLNNLAITSFVRRNLENKLTAKGLSLQEANKLIKDFLSSNKELAGKALNFHHPA:HPAG1_0524   NLKHDGVMFVDYSNFKYTNASKNPNKGVGVTNGVSHLEANLSKVAVFNLPNLNNLAITSYVRRDLEDKLWAKGLSPQEANKLIKDFLNSNKELVGKTLNFHF32:HPF32_0523   SLKYDGVMFVNYSNFKYTNASKSPNKGLGTTNGVSHLEANFSKVAVFNLPNLNNLAITNYIRRDLEDKLWAKGLSPQEANKLIKDFLNSNKEMVGKVSNFHF16:HPF16_0811   NLKYDGVMFVDYSNFKYTNASKSPDKGVGATNGVSHLEANFSKVAVFNLPNLNNLAITNYIRRDLEDKLWAKGLSSQEANKLIKDFLNSNKEMVGKVLNFHF57:HPF57_0574   SLKYDGLMFVNYSNFKYTNASKSPDKGVGATNGVSHLEANFSKVAVFNLPNLNNLAITNYIRRDLEDKLWAKGLSSQEANKLIKDFLNSNKELLGKVSNFHF30:HPF30_0779   SLKYDGVMFVNYSNFKYTNASKSPDKGVGTTNGVSHLEANFSKVAVFNLPNLNNLAITNYIRRDLEDKLLAKGLSPQEANKLIKDFLNSNKEMVGKVSNFH51:KHP_0771      NLKYDGVMFVNYSNFKYTNASKSPDKGIGTTNGVSHLEANFSKVAVFNLPNLNNLAITNYIRRDLEEKLWAKGLSLQEANKLIKDFLNSNKEMVRKVSNFH52:HPKB_0795     NLKHDGVMFVNYSNFKYTNASKSPDKGVGATNGVSRLEANLSKVAVFNLPNLNNLAITNYIRRDLEDKLWAKGLSPQEANKLIKDFLNSNKELLGKVSNF                  601       611       621       631       641       651       661       671       681       691                         |         |         |         |         |         |         |         |         |         |         HB8:HPB8_741      NKAVAEAKNTGNYDEVKKAQKNLEKSLRKREHLEKEVVKKLENRNDNKNRMEAKAQANSQKDKIFAIINEEASKEARVAACVQKFKGIKMELSDKFENINHSJM:HPSJM_02765  NKAVADAKNTGNYDEVKKAQKDLEKSLRKREHLEKEVEKKLESKSGNKNKMEAKAQANSQKDEIFALINKEANRDARAITYSQNLKGIKRELSDKLESINHP12:HPP12_0555   NKAVADAKNTGNYDEVKKAQKDLEKSLKKREHLEKEVAKNLESKSGNKNKMEAKSQANSQKDEIFALINKEANRDARAIAYAQNLKGIKMELSDKLENVNHG27:HPG27_507    NKAVAEAKNTGNYDEVKQAQKDLEKSLKKRERLEKEVAKKLESKSGNKNKMEAKSQANSQKDEIFALINKEANREARAITYAQNLKGIKRELSDKLENVNH266:HP0547       NKAVAEAKSTGNYDEVKKAQKDLEKSLRKREHLEKEVEKKLESKSGNKNKMEAKAQANSQKDEIFALINKEANRDARAIAYTQNLKGIKRELSDKLEKISHHPA:HPAG1_0524   NKAVAEAKNTGDYDEVKKAHKDLEKSLRKREHLEKEVAKKLESKSGNKNKMEAKSQANSQKDEIFALINKEANRDARAIAYAQNLKGIKRELSDKLENINHF32:HPF32_0523   NKAVAEAKNTGNYDEVKKAQKDLEKSLRKREHLEKEVAKKLESRNDNKNRMEAKAQANSQKDKIFALISQEASKEARVATFDPNLKGVRSELSDKLENINHF16:HPF16_0811   NKAVAEAKNTGNYDEVKKAQKDLEKSLRKREHLEKEVAKKLESRNDNKNRMEAKAQANSQKDKIFALINQEASKEARAAVFDPNLKGIRSELSDKLENINHF57:HPF57_0574   NKAVAEAKNTGNYDEVKKAQKDLEKSLRKREHLEKEVAKKLESRNDNKNRMEAKAQANSQKDKIFALINQEASKEARAAAFDPNLKGIRSELSDKLENINHF30:HPF30_0779   NKAVAEAKNTGNYDEVKKAQKDLEKSLRKREHLEKEVAKKLESRNDNKNRMEAKAQANSQKDKIFALINQEASKEARAAAFDPNLKGIRSELSDKLENINH51:KHP_0771      NKAVAEAKNTGNYDGVKKAQKDLEKSLRKREHLEKEVAKKLESRNDNKNKMEAKAQANSQKDKIFALINKEASKEARAAAFDPNLKGIRSELSDKLENINH52:HPKB_0795     NKAVAEAKNTGNYDEVKKAQKDLEKSLRKREHLEKEVAKKLESRNDNKNRMEAKAQANSQKDKIFALINQEASKEARAVAFDPNLKGVRSELSDKLENIN                  701       711       721       731       741       751       761       771       781       791                         |         |         |         |         |         |         |         |         |         |         HB8:HPB8_741      KNLKDFDKSFDDFKNGKNKDFSKAEETLKALKGSVKDLGINPEWISKVENLNTALNDFKNGKNKDFSKVTQAKSDLENSIKDVIINQKITDKVDNLNQAVHSJM:HPSJM_02765  KDLKDFSKSFDEFKNGKNKDFSKAEETLKALKGSVKDLGINPEWISKVENLNAALNDFKNGKNKDFSKVTQAKIDLENSIKDTIINQKITDKVDDLNQAVHP12:HPP12_0555   KNLKDFSKSFDEFKNGKNKDFSKAEETLKALKGSVKDLGINPEWISKVENLNAALNEFKNGKNKDFSKVAQAKSDLENSVKDVIINQKITDKVDNLNQAVHG27:HPG27_507    KNLKDFSKSFDEFKNGKNKDFSKSEETLKALKGSVKDLGINPEWISKVENLNAALNEFKNGKNKDFSKVTQAKSDLENSVKDVIINQKVTDKVDNLNQAVH266:HP0547       KDLKDFSKSFDEFKNGKNKDFSKAEETLKALKGSVKDLGINPEWISKVENLNAALNEFKNGKNKDFSKVTQAKSDLENSVKDVIINQKVTDKVDNLNQAVHHPA:HPAG1_0524   KDLKDFSKSFDEFKNGKNKDFSKTEETLKALKGSVKDLGINPEWISKVENLNAALNEFKNGKNKDFSKVTQAKSDLENSIKDVIINQKITDKADNLNQAVHF32:HPF32_0523   KNLKDFGKSFDELKSGKNNDFSKAEETLKALKDSVKDLGINPEWISKIENLNAALNDFKNGKNKDFSKVTQAKSDLENSIKDVIINQKITDKVDNLNQAVHF16:HPF16_0811   KNLKDFGKSFDELKNGKNNDFSKAEETLKALKDSVKDLGINPEWISKIENLNVALNDFKNGKNKDFSKVTQAKSDLENSIKDVIINQKITDKVDNLNQAVHF57:HPF57_0574   KNLKDFGKSFDELKSGKNKDFSKAEETLKALKDSVKDLGINPEWISKIENLNTALNDFKNGKNKDFSKVTQAKSDLENSIKDVIINQKITDKVDNLNQAVHF30:HPF30_0779   KNLKDFGKSVDELKNGKNNDFSKAEETLKALKDSVKDLGINPEWISKIENLNAALNDFKNGKNKDFSKVTQAKSDLENSIKDVIINQKITDKVDNLNQAVH51:KHP_0771      KNLKDFGKSFDELKNGKNKDFSKAEETLKALKDSVKDLGINPEWISKIENLNAALNDFKNGKNKDFSKVTQAKSDLENSIKDVIINQKITDKVDNLNQAVH52:HPKB_0795     KNLKDFGKSFDELKSGKNNDFNKAEETLKALKDSVKDLGINPEWISKIENLNAALNDFKNGKNKDFSKVTQAKSDFENSIKDVIINQKITDKVDNLNQAV                  801       811       821       831       841       851       861       871       881       891                         |         |         |         |         |         |         |         |         |         |         HB8:HPB8_741      SVAKATGDFSGVEQALADLKNFSKGQLAQQAQKNEDFNTGKNSELYQSVKNGVNGTLVGNGLSGIEATALTKNFSDIKKELNEKF-KNF-NNNNNGLKNSHSJM:HPSJM_02765  SVAKATGDFSRVEQALADLKNFSKEQLAQQTQKNESFNVGKKSEIYQSVKNGVNGTLVGNGLSQVEATTLSKNFSDIKKELNAKLFGNFNNNNNNGLKN-HP12:HPP12_0555   SVAKATGDFSRVEQALADLKNFSKEQLAQQAQKNEDFNTGKNSALYQSVKNGVNGTLVGNGLSKAEATTLSKNFSDIKKELNAKL-GNFNNNNNNGLKN-HG27:HPG27_507    SVAKATGDFSRVEQALADLKNFSKEQLAQQAQKNEDFNTGKNSALYQSVKNGVNGTLVGNGLSKAEATTLSKNFSDIKKELNAKL-GNFNNNNNNGLKNSH266:HP0547       SVAKAMGDFSRVEQVLADLKNFSKEQLAQQAQKNEDFNTGKNSELYQSVKNSVNKTLVGNGLSGIEATALAKNFSDIKKELNEKF-KNF-NNNNNGLKNSHHPA:HPAG1_0524   SVAKTTGDFSRVEQALADLKNFSKGQLAQQAQKNEDFNTGKNSELYQSVKNGVNKTLVGNGLSGIEATALAKNFSDIKKELNEKF-KNFNNNNNNGLKNEHF32:HPF32_0523   SEIKLTGDFSKVEQALAELKNLS-------------LDLGKNSDLQKSVKNGVNGTLVSNGLSKTEATTLTKNFSDIRKELNEKLFGN-SNNNNNGLKNNHF16:HPF16_0811   SETKLTGDFSKVEQALAELKSLS-------------LDLGKNSDLQKSVKNGVNGTLVGNGLSKTEATTLTKNFSDIRKELNEKLFGN-SNNNNNGLKNSHF57:HPF57_0574   SETKLTGNFSKVEQALAELKSLS-------------LDLGKNSDLQKSVKNGVNGTLVGNGLSKTEATTLTKNFSDIRKELNEKLFGN-SNNNNNGLKNNHF30:HPF30_0779   SETKLTGDFSKVEQALAELKSLS-------------LDLGKNSDLQKSVKNGVNGTLVGNGLSKTEATTLTKNFSDIRKELNEKLFGN-SNNNNNGLKN-H51:KHP_0771      SETKLTGDFSKVEQALAELKSLS-------------LDLGKNSDLQKSVKNGVNGTLVGNGLSKTEATTLTKNFSDIRKELNEKLFGN-SNNNNNGLKNNH52:HPKB_0795     SETKLTGDFSKVEQALAELKNLS-------------LDLGKNSDLQKSVKNGVNGTLVGNGLSKTEATTLAKNFSDIRKELNEKLFGN-SNNNNNGLKNS                  901       911       921       931       941       951       961       971       981       991                         |         |         |         |         |         |         |         |         |         |         HB8:HPB8_741      -----------------GEPIYAQVNKKKT------------------------------------GQVASPEEPIYTQVAKKVKAKIDQFNQVASGLGGHSJM:HPSJM_02765  ------------------EPIYAKVNKKKT------------------------------------EQAASPEEPIYTQVAKKVTQKIDRLNQIASGLGGHP12:HPP12_0555   ------------------EPIYAKVNKKKT------------------------------------GQAASHEEPIYAQVAKKVNAKIDRLNQIASGLGGHG27:HPG27_507    -----------------TEPIYAKVNKKKA------------------------------------GQAASPEEPIYAQVAKKVNAKIDRLNQIASGLGVH266:HP0547       -----------------TEPIYAKVNKKKT------------------------------------GQVASPEEPIYTQVAKKVNAKIDRLNQIASGLGGHHPA:HPAG1_0524   PIYAKVNKKKTGQVASPEEPIYAKVNKKKT------------------------------------GQVASPEEPIYTQVAKKVNAKIDRLNQIASGLGGHF32:HPF32_0523   -----------------TEPIYAQVNKKKT------------------------------------GQATSPEEPIYAQVAKKVSAKIDQLNEATSAINRHF16:HPF16_0811   -----------------AEPIYAKVNKKKT------------------------------------GQATSPEEPIYAQVAKKVSAKIDQLNESTSAINRHF57:HPF57_0574   -----------------TEPIYAQVNKKKA------------------------------------GQAASPEEPIYAQVAKKVSAKIDQLNEATSAINRHF30:HPF30_0779   ------------------EPIYAQVNKKKA------------------------------------GQVASPEEPIYAQVAKKVSAKIDQLNEATSAINRH51:KHP_0771      -----------------TEPIYAQVNKKKT------------------------------------GQAASPEESIYAQVAKKVSAKIDQLNESASAINRH52:HPKB_0795     -----------------TEPIYAKVAKKVSVKIDQLNEATSAINRKIDRINKIASAGKGVGGFSGAGRSASP-EPIYAQVAKKVSAKIDQLNEATSAINR                  1001      1011      1021      1031      1041      1051      1061      1071      1081      1091                        |         |         |         |         |         |         |         |         |         |         HB8:HPB8_741      VGQ-AG----------------------------------FSLKGHTKVDDLSKVGRSVSPEPIYATID----DLGGPFPLKRHDKVDDLSKVGLSRNQEHSJM:HPSJM_02765  VGQAAG----------------------------------FPLKRHDKVEDLSKVGRSVSPEPIYATID----DLGGPFPLKRHDKVDDLSKVGLSREQQHP12:HPP12_0555   VGQAAGFPLKKHDKVEDLSKVGLSASPEPIYATIDDLGGPFPLKKHDKVEDLSKVGLSASPEPIYATID----DLGGPFPLKKHDKVEDLSKVGLSRNQKHG27:HPG27_507    VGQAVGFPLKRHDKVGDLSKVGQSVSPEPIYATIDDLGGPFPLKRHDKVGDLSKVGLSVSPEPIYATID----DLGGPFPLKRHDKVGDLSKVGLSREQQH266:HP0547       VGQAAG----------------------------------FPLKRHDKVDDLSKVGLSASPEPIYATID----DLGGPFPLKRHDKVDDLSKVGRSRNQEHHPA:HPAG1_0524   VGQAAG----------------------------------FPLKRHDKVDDLSKVGRSVSPEPIYATID----DLGGPFPLKRHDKVDDLSKVGLSRNQEHF32:HPF32_0523   KIDRIN----------------------------------KIASAGKGVGGFSGAGRSASPEPIYATIDFDEANQAG-FPLRRSAAVNDLSKVGLSREQEHF16:HPF16_0811   KIDRIN----------------------------------KIASAGKGVGGFSGAGRSASPEPIYATIDFDEANQAG-FPLRRSAAVNDLSKVGLSREQEHF57:HPF57_0574   KIDRIN----------------------------------KIASAGKGVGGFSGAGRSASPEPIYATIDFDEANQAG-FPLRRYAGFDDLSKVGLSREQEHF30:HPF30_0779   KIDRIN----------------------------------KIASAGKGVGGFSGAGQSASPEPIYATIDFDDANQAG-FPLRRSAAVNDLSKVGLSREQEH51:KHP_0771      KIDRIN----------------------------------KIASAGKGVGGFSGAGRSASPEPIYATIDFDEANQAG-FPLRRYAPVDDLSKVGLSREQEH52:HPKB_0795     KIDRIN----------------------------------KIASAGKGVGGFSGAGRSASPEPIYATIDFDEANQAG-FPLMRSAAVNDLSKVGLSREQE                  1101      1111      1121      1131      1141      1151      1161      1171      1181      1191                        |         |         |         |         |         |         |         |         |         |         HB8:HPB8_741      LAQKIDNLNQAVSEAKACHFDNLDQMIDKLKDSTKKNVMNLYVESAKKVPTSLSAKLDNYAINSHTRINSNVKNGTINEKVTGMLTQKNPEWLKLVNDKIHSJM:HPSJM_02765  LKQKIDNLSQAVSEAKAGFFGNLEQTIDKLKDSTKHNPMNLWVESAKKVPTSLSAKLDNYATNSHTRINSNIKNGAINEKATGMLTQKNPEWLKLVNDKIHP12:HPP12_0555   LAQKIDNLNQAVSEAKAGFFGNLEQTIDKLKDSTKYNPVNLWVESAKKVPASLSAKLDNYATNSHTRINSNIKNGAINEKATGMLAQKNPEWLKLVNDKIHG27:HPG27_507    LKQKIDNLSQAVSEAKAGFFGNLEQTIDNLKDSAKNNPVSLWAEGAKKVPASLSAKLDNYATNSHTRINSNIQSGAINEKATGMLTQKNPEWLKLVNDKIH266:HP0547       LAQKIDNLNQAVSEAKAGFFGNLEQTIDKLKDSTKKNVMNLYVESAKKVPASLSAKLDNYAINSHTRINSNIQNGAINEKATGMLTQKNPEWLKLVNDKIHHPA:HPAG1_0524   LAQKIDNLNQAVSEAKAGFFGNLEQAIDKLKDSTKHNPMNLWVESAKKVPASLSAKLDNYATNSHTRINSNIQNGAINEKVTGMLTQKNPEWLKLVNDKIHF32:HPF32_0523   LTRRIGDLSQAVSEAKTGHFGNLEQKIDELKDSTKKNALKLWVESAKQVPTSLQAKLDNYATNSHTRINSNVQSGTINEKATGMLTQKNPEWLKLVNDKIHF16:HPF16_0811   LTRRIGDLNQAVSEAKTGHFDNLEQKIDELKDSTKKNALKLWVESAKQVPTGLQAKLDNYATNSHTRINSNVHNGAINEKATGMLTQKNPEWLKLVNDKIHF57:HPF57_0574   LTRRIGDLNQAVSEAKTGRFDNLEQKIDELKDSTKKNALKLWVESTKQVPTSLQAKLDNYATNSHTRINSNVQSGTINEKATGMLTQKNPEWLKLVNDKIHF30:HPF30_0779   LTRRIGDLNQAVSEAKTGRFDNLEQKIDELKDSTKKNALKLWVESAKQVPIGLQAKLDNYATNSHTRINSNVQSGTINEKATGMLTQRNPEWLKLVNDKIH51:KHP_0771      LTRRIGDLNQAVSEAKIGHFDNLEQKIDELKDSTKKNALKLWVESAKQVPTGLQAKLDNYATNSHTRINSNVQTGAINEKATGMLTQKNPEWLKLVNDKIH52:HPKB_0795     LTRRIGDLNQAVSEAKTGHFGNLEQKIDELKDSTKKNALKLWVESAKQVPTGLQAKLDNYATNSHTRINSNVQSGAINEKATGMLMQKNPEWLKLVNDKI                  1201      1211      1221      1231      1241      1251      1261      1271      1281      1291                  |         |         |         |         |         |         |         |         |         |HB8:HPB8_741      VAHNVGSVPLSEYDKIGFNQKNMKDYSDSFKFSTRLSNAVKDIKSGFVQFLTNTFSM-GSYSLMKASVEHGVKNTNTKGGFQK--------------SHSJM:HPSJM_02765  VAHNVGSVPLSEYDKIGFNQKNMKDYSDSFKFSTKLNNAVKDVKSGFTQFLANAFST-GYYCLAGENAEHGIKNVNTKGGFQK--------------SHP12:HPP12_0555   VAHNVGSVPLLEYDKIGFNQKNMKDYSDSFKFSTNLNNAVKDIKSGFTQFLANAFST-GYYCLARENAEHGIKNVNTKGGFQK--------------SHG27:HPG27_507    VAHNVGSVPLLEYDKIGFNQKSMKDYSDSFKFSTELNNAVKDVKSGFTQFLANAFST-GYYRLAGENAEHGIKNVNTKGGSKNLKGLRNTKNAKTTPCH266:HP0547       VAHNVGSVSLSEYDKIGFNQKNMKDYSDSFKFSTKLNNAVKDIKSGFTHFLANAFST-GYYCLARENAEHGIKNVNTKGGFQK--------------SHHPA:HPAG1_0524   VAHNVGSVPLSEYDRIGFNQKNMKDYSDSFKFSTKLNNAVKDIKSGFVQFLTNTFSTASYYCLAEENAKHGIKNANTKGGFQK--------------SHF32:HPF32_0523   VAHNVGSAPLSAYDKIGFNQKNMKDYSDSFKFSTKLNNAVKDIKSSFVQFLTNTFST-GSYSLMKANVEHGVKNTNTKGGFQK--------------SHF16:HPF16_0811   VAHNVGSAHLSEYDKIGFNQKNMKDYSDSFKFSTKLNNAVKDIKSSFVQFLTNTFST-GSYSLTKANVEHGVKNT-TKSGFQK--------------SHF57:HPF57_0574   VAHNVGSAHLSEYDKIGFNQKNMKDYSDSFKFSTKLNNAVKDIKSSFVQFLTNTFST-GSYSLMKANAEHGVKNT-TKSGFQK--------------SHF30:HPF30_0779   VAHNVGSAHLSEYDKIGFNQKNMKDYSDSFKFSTKLNNAVKDIKSSFVQFLTNTFST-GSYSLMKANVEHGVKNT-TKSGFQK--------------SH51:KHP_0771      VAHNVGSAHLSEYDKIGFNQKNMKDYSDSFKFSTKLNNAVKDIKSSFVQFLTNTFST-GSYSLMKANAEHGVKNTNTKGGFQK--------------SH52:HPKB_0795     VAHNVGSTPLSEYDKIGFNQKNMKDYSDSFKFSTKLNNAVKDVKSDFVQFLTNAFST-GSYSLMKANAEHGVKNTNTKGGFQK--------------S
