# Supplementary material for: Evolution in an oncogenic bacterial species with extreme genome plasticity: Helicobacter pylori East Asian genomes
Source: BMC Microbiol. 2011 May 16;11:104. doi: 10.1186/1471-2180-11-104 (PMC3120642; doi:10.1186/1471-2180-11-104)
Supplement: Additional file 6 — Multiple sequence alignments of diverged genes. [file 1471-2180-11-104-S6.ZIP › Diverged_genes_multiple_seuence_alignments/HP0596.mfa.rtf]

                  1         11        21        31        41        51        61        71        81        91                          |         |         |         |         |         |         |         |         |         |         HB8:mHPB8_794     VLEKSFLKSKQLFLCGLGVLMLQACTCPNTSQRNSFLQDVPYWMLQNRSEYITQGVDSSHIVDGKKTEEIEKIATKRATIRVAQNIVHKLKEAYLSKSNRHG27:mHPG27_556   VLEKSFLKSKQLFLCGLGVLMLQACTCPNTSQRNSFLQDVPYWMLQNRSEYITQGVDSSHIVDGKKTEEIEKIATKRATIRVAQNIVHKLKEAYLSKSNRH266:HP0596       VLEKSFLKSKQLFLCGLGVLMLQACTCPNTSQRNSFLQDVPYWMLQNRSEYITQGVDSSHIVDGKKTEEIEKIATKRATIRVAQNIVHKLKEAYLSKTNRHB38:HELPY_0776   VLEKSFLKSKQLFLCGLGVLMLQACTCPNTSQRNSFLQDVPYWMLQNRSEYITQGVDSSHIVDGKKTEEIEKIATKRATIRVAQNIVHKLKEAYLSKTNRHP12:HPP12_0603   VLEKSFLKSKQLFLCGLGVLMLQACTCPNTSQRNSFLQDVPYWMLQNRSEYITQGVDSSHIVDGKKTEEIEKIATKRATIRVAQNIVHKLKEAYLSKSNRHSJM:HPSJM_03020  VLEKSFLKSKQLFLCGLGVLMLQACTCPNTSQRNSFLQDVPYWMLQNRSEYITQGVDSSHIVDGKKTEEIEKIATKRATIRVAQNIVHKLKEAYLSKSNRHHPA:HPAG1_0576   VLEKSFLKSKQLFLCGLGVLMLQACTCPNTSQRNSFLQDVPYWMLQNRSEYLTQGVDSSHIVDGKTTEEIEKIATKRATIRVAQNIVHKLKEAYLSKSNRHF32:HPF32_0571   VLEKSFLKSKQLVLCGLGVLMLQACTCPNTSQRNSFLQDVPYWMLQNRSEYITQGVDSSHIVDGKKTEEIEKIATKRATIRVAQNIVHKLKEAYLSKSNRHF57:HPF57_0621   VLEKSFLKSKQLVLCGLGVLMLQACTCPNTSQRNSFLQDVPYWMLQNRSEYITQGVDSSHIVDGKKTEEIEKIATKRATIRVAQNIVHKLKEAYLSKSNRHF16:HPF16_0763   VLEKSFLKSKQLVLCGLGVLMLQACTCPNTSQRNSFLQDVPYWMLQNRSEYITQGVDSSHIVDGKKTEEIEKIATKRATIRVAQNIVHKLKEAYLSKSNRHF30:HPF30_0731   VLEKSFLKSKQLVLCGLGVLMLQACTCPNTSQRNSFLQDVPYWMLQNRSEYITQGVDSSHIVDGKKTEEIEKIATKRATIRVAQNIVHKLKEAYLSKSNRH52:HPKB_0747     VLEKSFLKSKQLVLCGLGVLMLQACTCPNTSQRNSFLQDVPYWMLQNRSEYITQGVDSSHIVDGKKTEEIEKIATKRATIRVAQNIVHKLKEAYLSKSNRH51:KHP_0722      VLEKSFLKSKQLVLCGLGILMLQACTCPNTSQRNSFLQDVPYWMLQNRSEYITQGVDSSHIVDGKKTEEIEKIATKRATIRVAQNIVHKLKETYLSKSNR                  101       111       121       131       141       151       161       171       181       191                  |         |         |         |         |         |         |         |         |         |HB8:mHPB8_794     IKQKITNEMFVQMTQPIYDSLMNVDRLGIYINPNNEEVFALVRARGFDKDALSEGLHKMSLDNQAVSILVAKVEEIFKDSVNYGDVKVPIAMHG27:mHPG27_556   IKQKITNEMFIQMTQPIYDSLMNVDRLGIYINPNNEEVFALVRARGFDKDALSEGLHKMSLDNQAVSILVAKVEEIFKDSVNYGDIKVPIAMH266:HP0596       IKQKITNEMFIQMTQPIYDSLMNVDRLGIYINPNNEEVFALVRARGFDKDALSEGLHKMSLDNQAVSILVAKVEEIFKDSVNYGDVKVPIAMHB38:HELPY_0776   IKQKITNEMFIQMTQPIYDSLMNVDRLGIYINPNNEEVFALVRARGFDKDALSEGLHKMALDNQAVSILVAKVEEIFKDSINYGDIKVPIAMHP12:HPP12_0603   IKQKITNEMFIQMTQPIYDSLMNVDRLGIYINPNNEEVFALVRARGFDKDALSEGLHKMALDNQAVSILVAKVEEIFKDSINYGDIKVPIAMHSJM:HPSJM_03020  IKQKITNEMFIQMTKPIYDSLMNVDRLGIYINPNNEEVFALVRARGFDKDALSEGLHKMALDNQAVSILVAKVEEIFKDSVNYGDIKVPIAMHHPA:HPAG1_0576   IKQKITNEMFIQMTQPIYDSLMNVDRLGIYINPNNEEVFALVRARGFDKDALSEGLHKMSLDNQAVSILVAKVEEIFKDSVNYGDIKVPIAMHF32:HPF32_0571   IKQKITNEMFIQMTQPIFDSLMNVDRLGIYINPNNEEVFALVCARSFDKDVLSEGLHKMSLDNQAVSILISKVEEIFKDSINYSDVKVPIAMHF57:HPF57_0621   IKQKITNEMFVQMTQPIFDSLMNVDRLGIYINPNNEEVFALVRARSFDKDVLSEGLHKMSLDNQAVSILISKVEEIFKDSINYSDVKVPIAMHF16:HPF16_0763   IKQKITNEMFIQMTQPIFDSLMNVDRLGIYINPNNEEVFALVRARSFDKDVLSEGLHKMSLDNQAVSILISKVEEIFKDSINYSDVKVPIAMHF30:HPF30_0731   IKQKITNEMFIQMTQPIFDSLMNVDRLGIYINPNNEEVFALVRARSFDKDALSEGLHKMSLDNQAVSILISKVEEIFKDSINYSDVKVPIAMH52:HPKB_0747     IKQKITNEMFIQMTQPIFDSLMNVDRLGIYINPNNEEVFALVRARSFDKDVLSEGLHKMSLDNQAVSILVAKVEEIFKESINYSDVKVPIAMH51:KHP_0722      IKQKITNEMFIQMTQPIFDSLMNVDRLGIYINPNNEEVFALVRARGFDKDVLSEGLHKMSLDNQAVSILISKVEEIFKDSINYSDVKVPIAM
