# Supplementary material for: Evolution in an oncogenic bacterial species with extreme genome plasticity: Helicobacter pylori East Asian genomes
Source: BMC Microbiol. 2011 May 16;11:104. doi: 10.1186/1471-2180-11-104 (PMC3120642; doi:10.1186/1471-2180-11-104)
Supplement: Additional file 6 — Multiple sequence alignments of diverged genes. [file 1471-2180-11-104-S6.ZIP › Diverged_genes_multiple_seuence_alignments/HP0635_hydE.mfa.rtf]

                  1         11        21        31        41        51        61        71        81        91                          |         |         |         |         |         |         |         |         |         |         HB8:mHPB8_835     LAFVFLFKCVNEKTSLNFTPLLERMACNLQARFYSVYKDNTASFYLQASAETTLEFAQKLSEILPFSLDFSFLSLKEITEPLDENLFQTTSLSKPLFMNAHSJM:HPSJM_03225  LAFVFLFKCVNEKTSLNFTPLLEQMALHLQARFYSVYKDNTTSFYLQASAEITLEFAQKLSEILPFSLDFSFLSLKEITEPLDENLFQTKSLSKPLFMNAHB38:HELPY_0736   LAFVFLFKCVNEKTSLNFTPLLEQMVLHLQARFYSVYKDNTTSFYLQASAEITLEFAQKLSEILPFSLDFSFLSLKEITEPLDENLFQTTSLSKPLFMNAHG27:HPG27_596    LVFVFLFRCVNEKASLNFTPLLEQMALHLQARFYSVYKDNTTSFYLQASAEITLEFAQKLSEILPFSLDFSFLSLKEITEPLDENLFQTTSLSKPLFMNAHHPA:HPAG1_0618   LAFVFLFKCVNEKTSLNFMPLLEQIAFHLQARFYSVYKDNTTSFYLQASAEITLEFAQKLSEILPFSLDFSFLSLKEITEPLDENLFQTTSLSKPLFMNAH266:HP0635       LVFVFLFKCVNEETSLNFTPLLERMACNLQARFYSVYKDNTTSFYLQASAETTLEFAQKLSEILPFSLDFSFLSLKEITEPLDENLFQTASLSKPLFMNAHP12:HPP12_0647   LAFVFLFKCVNEKASLNFTPLLEQMALHLQARFYSVYKDNTTSFYLQASAEITLEFAQKLSEILPFSLDFSFLSLKEITEPLDENLFQTTSLSKPLFMNAHF32:HPF32_0612   LVFVFLFKCANEETSLNFTPLLEQMAFNLQAHFYSVYKDNTTSFYLQASAETTLEFAQKLSEILPFSLDFSFLSLKEITEPLDENLFQTTSLSKPLFMNAH51:KHP_0685      LVFVFLFKCTNEETSLNFTPLLEQMAFNLQARFYSVYKDNTTSFYLQASTETTLEFAQKLSEILPFSLDFSFLSLKEITEPLDENLFQTTSLSKPLFMNAHF16:HPF16_0725   LVFVFLFKCANEETSLNFTPLLEQMAFNLQARFYSVYKDNTTSFYLQASAETTLEFAQKLSEILPFSLDFSFLSLKEITEPLDENLFQTTSLLKPLFMNAHF30:HPF30_0692   LAFVFLFKCANEETSLNFTPLLEQMACNLQARFYSVYKDNTASFYLQASAETTLEFAQKLSEILPFSLDFSFLSLKEITEPLDENLFQTTSLSKPLFMNAHF57:HPF57_0659   LAFVFLFKCANEETSLNFTPLLEQMAFNLQACFYSVYKDNTASFYLQASAETTLEFAQKLSEILPFSLDFSFLSLKEITEPLDENLFQTTSLSKPLFMNTH52:HPKB_0708     LVFVFLFKCANEETSLNFTPLLEQMACHLQACFYSVYKDNTTSFYLQASAETTLEFAQKLSEILPFSLDFSFLSLKEITEPLDENLFQTTSLSKPLFMNA                  101       111       121       131       141       151       161       171       181       191                         |         |         |         |         |         |         |         |         |         |         HB8:mHPB8_835     KEHQDFLDKNSSLYANALGFVKNAAFKGAIIHSPKELIDCLTQLKGMLKTQDFIPIFTSRGALSLSLKKPSPSVIFSDLSSVLTCTKLPLEDAQYLASLEHSJM:HPSJM_03225  KEHQDFLDKNASLYANVLGFVKNTAFKGDIIHSPKELIDCLTQLKGMLKTQDFIPIFTSRGALSLSLKNPSPSVIFSDLSSVLTCTKLPLEDAKYLASLEHB38:HELPY_0736   KEHQDFLDKNASLYADALGFVKNTAFKGNIIHSPKELIDCLTQLKGMLKTQDFIPIFTSRGALSLSLKNPSPSVIFSDLSSVLSCTKLPLEDAKYLASLEHG27:HPG27_596    KEHQDFLDKNASLYADTLGLIENTAFKGDIIHSPKELIDCLTQLKGMLKTQDFTPIFTSRGALSLSLKNPSPSVIFSDLSSVLSCTKLPLEDAKYLASLEHHPA:HPAG1_0618   KEHQDFLDKNSSLYADTLGLIKNAAFKGAIIHSPKELIDCLTQLKGMLKTQDFIPIFTSREALSLSLKNPSPSAIFSDLSSVLSCTKLPLEDAKYLASLEH266:HP0635       KEHQDFLDKNSSLYADTLGLIKNTAFKGDIIHSPKELIDCLTQLKGMLKTQDFIPIFTSREALSLSLKNPSPSVIFSDLSSVLSCTKLPLEDAKYLASLEHP12:HPP12_0647   KEHQDFLDKNASLYANALGFVKNTAFKGAIIHSPKELIDCLTQLKEALKTQDCIPIHTSRGALSLSLKKPSPSVIFSDLSSVLSCTKLPLEDAQYLASLEHF32:HPF32_0612   KEHQDFLDKNSSLYANALGFVKNTAFKGAIIHSPKELIDCLTQLKEALKTQDFIPIHTSRGALSLSLKNPSPSVIFSDLSSVLTCTKLPLEDAKYLASLEH51:KHP_0685      KEHQDFLDKNSSLYANALGFVKNTAFKGATIHSPKELIDCLTQLKSMLKTQDFIPIHTSRGALSLSLKNPSPSVIFSDLSSVLTCTKLSLEDAKYLASLEHF16:HPF16_0725   KEHQDFLDKNSSLYANALGFVKNTAFKGAIIHSPKELIDCLTQLKSMLKTQDFIPIHTSRGALSLSLKNPSPSVIFSDLSSVLSCTKLSLEDAKYLASLEHF30:HPF30_0692   KEHQDFLDKNSSLYANALGFVKNTAFKGAIIHSPKELIDCLTQLKSMLKTQDFIPIHTSRGALSLSLKNPSPSVIFSDLSSVLTCTKLPLEYAKYLASLEHF57:HPF57_0659   KEHQDFLDKNSSLYANALGFVKNTAFKGAIIHSPKELIDCLTQLKGMLKTQDFIPIHTSRGALSLSLKNPSPSVIFSDLSSVLTCTKLPLEDAKYLASLEH52:HPKB_0708     KEHQDFLDKNSSLYANALGFVKNTAFKGAIIHSPKELIDCLTQLKSMLKTQDFIPIHTSRRALSLSLKNPSPSVIFSDLSSVLTCTKLPLEDAKYLASLE                  201       211       221       231       241       251       261       271       281       291                         |         |         |         |         |         |         |         |         |         |         HB8:mHPB8_835     KPCIKASLKSVFKDIFKNDEIIAQLPYDPILNLLCHILQDEGIEFVFTHESRSCEALLHYEALFKTPKRLITPTKKFVLENHLSTFPFKDELEFLSATPNHSJM:HPSJM_03225  KPSIKASLKSVFKDTFKNDEIIAQLPFDPILNLLCHILQDEGIEFVFMHESRSCEALLHYETLFKTPKRLITPTKKFVLENHLSTFPFKDELEFLSATPNHB38:HELPY_0736   KPSIKASLKSVFKDTFKNDEIIAQLPYDPILNLLCHILQDEGIEFVFMHESRSCEALLHYEALFKTPKRLITPTKKFVLENNLSTLPFKDELEFLSTTPNHG27:HPG27_596    KPSIKASLKSVFKDTFKNDEIIAQLPYDPILNLLCRILQDEGIEFVFMHESRSYEALLHYEALFKTPKRLITPTKKFVLENNFSTLPFKDELEFLSATPNHHPA:HPAG1_0618   KPSIKAPLKSVFKDTFKNDEIIAQLPYDPILDLLCHILQDEGIEFVFMHESRSCEALLHYEALFKTPKRLITPAKKFVLENNLSTLPFKDELEFLSATPNH266:HP0635       KPSIKAPLKSVFKDTFKNDEIIAQLPYDPILNLLCHILQDEGIEFVFMHESRSCEALLYYEALFKTPKRLITPTKKFVLENNFSTFPFKDELEFLSATPNHP12:HPP12_0647   KPSIKASLKSVFKDTFKNDEIIAQLPYDPILNLLCRILQDEGIEFVFIHANNPQEALLHYEALFKTPKRLITPTKKFVLENNLSTLPFKDELEFLSATPNHF32:HPF32_0612   KPCVKASLKSVFKDTFKNDEIIAQLPFDPILNLLCRILQDEGIEFVFTHANNSQEALLHYETLFKTPKRLITPTKKFVLENNLSTLAFKDELEFLKETPHH51:KHP_0685      KPSIKASLKSVFKDTFKNDEIIAQLPFDPILNLLCRILQDEGIEFVFTHANNSQEALLHYETLFKTPKRLITPTKKFVLENNLSTLAFKDELEFLKETPHHF16:HPF16_0725   KPSIKASLKSVFKDTFKNDGIIAQLPFDPILNLLCRILQDEGIEFVFTHANNPQEALLHYETLFKTPNRLITPTKKFVLENNLSTLAFKDELEFLKETPHHF30:HPF30_0692   KPSIKASLKSVFKDIFKNDEIIAQLPFDPILNLLCRILQDEGIEFVFTHANNPQEALLHYETLFKTPKRLITPTKKFVLENNLSTIAFKDELEFLKETPHHF57:HPF57_0659   KPCVKASLKSVFKDTFKNDEIIAQLPFDPILNLLCRILQDEGIEFVFIHANNPQEALLYYETLFKTPKRLITPTKKFVLENNLSAIIFKDELEFLKETPHH52:HPKB_0708     KPCVKASLKSVFKDTFKNDEIIAQLPFDPILNLLCRILQDEGIEFVFIHANNPQEALLHYETLFKTPNRLITPTKKFVLENNLSTLAFKDELEFLKETPH                  301       311       321       331       341       351       361       371       381       391                         |         |         |         |         |         |         |         |         |         |         HB8:mHPB8_835     SIVLYFSFKRPTRLLLHANGSLKTLLSVSFDFNQIFNLLKQDEKASRMLKNYAAKFPDFYARIAELSKHNLGGANVLDFFRILGFVLGYSEDFHSHSVISHSJM:HPSJM_03225  SIVLYFSFKRPTRLLLHANGSLKTLLSVSFDFNQMFNLLKQDEKASRMLQNYATKFPNFYARLLELSKYQLGGANLLDFFRILGFVLGYSEDFCAQSVISHB38:HELPY_0736   SIVLYFSFKHPTRLLLHANGSLKTLLSVSFDFNQIFNLLKQDEKASRMLQNYATKFPDFYARIVELSKYNLGGANLLDFFRILGFVLGYSEDFCTQSVISHG27:HPG27_596    SIVLYLSFKHPTRLLLHANGSLKTLLSVSFDFNKIFNALKQDEKASRMLKNYATKFPNFYARILELSKYQLGGANLLDFFQILGFVLGYSEDFCTQSVIPHHPA:HPAG1_0618   SIVLYFSFKRPTRLLLHANGSLKTLLSISFDFNQIFNTLKQDEKASRMLQNYATKFPNFYARVLELSKYQLGGANLLDFFQILGFVLGYSEDFCTQSVIPH266:HP0635       SIVLYLSFKRPTRLLLHANGSLKTLLSVSFDFNKMFNALKQDEKASRMLQNYATKFPDFYARIVELSKYDLGGANLLDFFCILGFVLGYSEDFCTQSVIPHP12:HPP12_0647   SIVLYFSFKRPTRLLLHANGSLKTLLSVSFDFNQIFNLLKQDEKASRMLQNYATKFPDFYARIVELSKYQLGGANLLDFFCILGFVLGYNEDFCAQSVISHF32:HPF32_0612   SIVLYLSFKRPTRLLLHANGSLKTLLSVSFDFNQSFNFLKQDEKASRMLQNYKAKFPNFYARILELSNYNLGGANLLDFFQILGFILGYSEDFCAQSVISH51:KHP_0685      SIVLYISFKRPTRLLLHANGSLKTLLSVSFDFNQSFNLLKQDEKASRMLKNYATKFPNFYARILELSNYNLGGANLLDFFQILGFVLGYSEDFCAQSVISHF16:HPF16_0725   SIVLYFSFKRPTRLLLHANGSLKTLLSVKFDFNQSFNLLKQDEKASKMLQNYATKFPNFYARILELSKYQLGGANLLDFFQILGFVLGYSEDFCTQNVISHF30:HPF30_0692   SIVLYFSFKRPTRLLLHANGSLKTLLSVKFDFNQSFNLLKQDEKASRMLKNYEAKFPNFYARILELSNHNLGGANLLDFFQILGFVLGYSEDFCAQSVISHF57:HPF57_0659   SIVLYLSFKRPTRLLLHANGSLKTLLSVNFDFNQSFNLLKQDEKASRMLKNYEAKFPNFYARILELSKYQLGGANLLDFFQILGFVLGYSEDFCAQSVISH52:HPKB_0708     SIVLYFSFKRPTRLLLHANGSLKTLLSVSFDFNQSFNLLKQDEKASRMLKNYEAKFPNFYARILELSKYQLGGANLLDFFQILGFILGYSEDFCAQSVIS                  401       411       421       431       441       451       461       471       481       491                         |         |         |         |         |         |         |         |         |         |         HB8:mHPB8_835     LAKECLRPKGPRIDYKILKDDSLKMALNFSKIMHSAMSFRLAGVENEILSLGILDSLAEFLGNFIWDNAQNFSVQEVTIAGDFFGEKVFLDLFVQYFPKTHSJM:HPSJM_03225  LAKECLRPKGPRIDYKILKDDSLKMALNFSKIMHSAMSFRLAGVENEILSLGILDSLAEFLGNFIWDNVQNFSVQEVTIAGDFFGEKVFLDLFVRYFPKTHB38:HELPY_0736   LARECLRPKGPRIDYKILKDDSLKMALNFSKIMHSAMSFRLAGVENEILSLGILDSLAEFLGNFIWDNAQNFSVQEVTIAGDFFGEKVFLDLFVQYFPKTHG27:HPG27_596    LAKECLRPKGPRIDYKILKDDSFKMALNFSKIMHSAMSFRLAGVENEILSLGILDSLAEFLGNFIWDNAQNFSVQEVTIAGDFFGEKVFLDLFVRYFPKTHHPA:HPAG1_0618   LAKECLRPKGPRIDYKILKGDSLKMALNFSKIMHSAMSFRLAGVENEILSLGILDSLAEFLGNFIWDNAQNFSVQEVTIAGDFFGEKVFLDLFVQYFPKTH266:HP0635       LAKECLRPKGPRIDYKILKDNSLKMALNFSKIMHSAMSFRLAGVENEILSLGILDSLAEFLGNFIWDNAQNFSVQEVTIAGDFFGEKVFLDLFVRYFPKTHP12:HPP12_0647   LAKECLRPKGPRIDYKILKDDSLKMALNFSKIMHSAMSFRLAGVENETLSLGILDSLAEFLGNFIWDNAQNFSIQEVTIAGDFFGEKVFLDLFVRYFPKTHF32:HPF32_0612   LAKECLRPKGPRIDYKILKDDSFKMALNFSKVMHSAMSFRLAGVENEILSLGILDSLAEFLGNFIWDNAQNFSVQEVTIAGDFFGEKVFLDLFVQYFPKTH51:KHP_0685      LAKECLRPKGPRIDYKILKDDSFKMALNFSKVMHSAMSFRLAGVENEILSLGILDSLAEFLGNFIWDNAQNFSVQEVTIAGDFFGEKVFLDLFVQYFPKTHF16:HPF16_0725   LAKECLRPKGPRIDYKILKDDSFKMALNFSKVMHSAMSFRLAGVENEILSLGILDSLAEFLGNFIWDNAQNFSVQEVTIAGDFFGEKVFLDLFVQYFPKTHF30:HPF30_0692   LAKECLRPKGPRIDYKILKDDSFKMALNFSKVMHSAMSFRLAGVENEILSLGILDSLAEFLGNFIWDNAQNFSVQEVTIAGDFFGEKVFLDLFVQYFPKTHF57:HPF57_0659   LAKECLRPKGPRIDYKILKDNSFKMALNFSKVMHSAMSFRLAGVENEILSLGILDSLAEFLGNFIWDNAQNFSVQEVTIAGDFFGEKVFLDLFVQYFPKTH52:HPKB_0708     LAKECLRTKGPRIDYKILKDDSFKMALNFSKIMHSAMSFRLAGVENEILSLGILDSLAEFLGNFIWDNAQNFSVQEVTIAGDFFGEKVFLDLFVQYFPKT                  501       511                  |         |HB8:mHPB8_835     LALKTHAFLDYEHSJM:HPSJM_03225  LALKVHAFLDYEHB38:HELPY_0736   LALKTHAFLDYEHG27:HPG27_596    LALKVHAFLDYEHHPA:HPAG1_0618   LTLKTHEFLDYEH266:HP0635       LALKTHAFLDYEHP12:HPP12_0647   LALKAHAFLDYEHF32:HPF32_0612   LTLKTHAFLDYEH51:KHP_0685      LTLKTHAFLDYEHF16:HPF16_0725   LTLKTHAFLDYKHF30:HPF30_0692   LTLKTHEFLDYEHF57:HPF57_0659   LTLKTHAFLDYEH52:HPKB_0708     LTLKTHAFLDYE
