# Supplementary material for: Evolution in an oncogenic bacterial species with extreme genome plasticity: Helicobacter pylori East Asian genomes
Source: BMC Microbiol. 2011 May 16;11:104. doi: 10.1186/1471-2180-11-104 (PMC3120642; doi:10.1186/1471-2180-11-104)
Supplement: Additional file 6 — Multiple sequence alignments of diverged genes. [file 1471-2180-11-104-S6.ZIP › Diverged_genes_multiple_seuence_alignments/HP0636.mfa.rtf]

                   1         11        21        31        41        51        61        71        81        91                           |         |         |         |         |         |         |         |         |         |         HB8:HPB8_836       -----------MRIVRNLFLVSFVAYSSAFATDLETETKSEKKSGKKFYKFHKNHGSETET----KNDKKLYDFTKNSGLEGVDLEKSPNLKSHKKSDKKHSJM:mHPSJM_03230  -----------MRIVRNLFLVSFVAYSSAFAVDLETETKSEKKSDKKFYKLHKNHGLKTEA----KNDKKLYDFTKNSALEGIDLEKSPNLKSHKKSDKKHHPA:HPAG1_0619    -----------MRIVRNLFFVSLMAYSSVFAADLETETKSEKKSSKKFYKLHKNHGLKTEA----KNDKKLYDFTKNSALEGIDLEKSPSLKSHKKSDKKHB38:HELPY_0735    -----------MRIVRNLFLVSLVAYSSAFATDLETETKNDKKSGKKFYKLHKNHGLKTEA----KNDKKLYDFTKNSALEGIDLEKSPKLKSHKKSDKKHP12:HPP12_0648    -----------MRIVRNLFLVSLVAYSSAFAADLETETKSDKKSSKKFYKLHKNHGLKTEA----KNDKKLYDFTKNSALEGIDLEKSPNLKSHKKSDKKHF32:HPF32_0613    -----------MRIVKNLFLVSFVAYSSMFAVDLETGTKSEKKSSKKFYELHKNHGLETEA----KSSKKLYDFTKNSALEGINLEKSPTLKSHKKSDKKHF30:HPF30_0691    -----------VRIVKNLFLVSFVAYSSAFAVDLETGTKSEKKSSKKFYELHKNHRLETEA----KNDKKLYDFTKNSALEGINLEKSPTLKSHKKSDKKHF16:HPF16_0724    -----------VHIVKNLFLVSFVAYSSAFAVDLETGTKSEKKSSKKFYELHKNHGLETEAKKDKKSNKKLYDFTKNSALEGINLEKSPTLKSHKKSDKKH51:KHP_0684       -----------VRIVKNLFLVSFVAYSSAFAVDLETETKSGKKSDKKFYELHKNHGLETEVKKDKKNDKKLYDFTKNSALEGINLEKSPTLKSHKKSNKKH52:HPKB_0707      -----------VRIVKNLFLVLFVAYSSAFAVDLEIETKSEKKSDKKFYKLHKNHGLETEAKKDKKSNKKLYYFTKNSALEGINLEKSPTLKSHKKSDKKHF57:HPF57_0660    MIEKDYIRGDLVRIVKNLFLVLFVAYSSAFAVDLEIEAKKDKKS-----------------------NKKLYYFTKNSALEGINLEKSPTLKSHKKSDKKHG27:HPG27_597     -----------MNIIKTLSLLFFVICNNVFATNLEAETKKDKKS-----------------------SKKLYYFTKNSALEGIDLEKSPKLKSHKKSDKK                   101       111       121       131       141       151                   |         |         |         |         |         |HB8:HPB8_836       FYKQLAKNNIAEGVSMPIVNFNKALSFGPYFERTKSKKTQYMDGGLMMHIRFHSJM:mHPSJM_03230  LYKQLPKNRISEGVSMPIVDFNKTLSFGPYFERTKSKKTQYMDGGLMIHIRFHHPA:HPAG1_0619    LYKQLPKNRISEGMSMPIVDFNKTLSFGPYFERTKSKKTQYMDGGLMMHIRFHB38:HELPY_0735    LYKQLPKNRISEGISMPIVEFNKTLSFGPYFERTKSKKTQYMDGGLMMHIRFHP12:HPP12_0648    LYKQLAKNNIAEGVSVPIVNFNKALSFGPYFERTKSKKTQYMDGGLMMHIRFHF32:HPF32_0613    FYKQLAKNNIAEGVSMPVVNFNKALSFGPYFERTKSKKTQYMDGGLMVHIRFHF30:HPF30_0691    FYKQLAKNNIAEGVSMPVVNFNKTLSFGPYFERTKSKKTQYMDGGLMMHIRFHF16:HPF16_0724    FYKQLAKNNIAEGVSMPVVNFNKALSFGPYFERTKSKKTQYMDGGLMIHIRFH51:KHP_0684       FYKQLAKNNIAEGVSMPVVNFNKALSFGPYFERTKSKKTQYMDGGLMIHIRFH52:HPKB_0707      FYKQLAKNNIAEGVSMPVVNFNKTLSFGPYFERTKSKKTQYMDGGLMIHIRFHF57:HPF57_0660    FYKQLPKNRISEGVSMPVVNFNKTLSFGPYFERTKSKKTQYMDGGLMMHIRFHG27:HPG27_597     LYKQLPKNRISEGMSMPIVDFNKTLSFGPYFERTKSKKTQYMDGGLMMHIRF
