# Supplementary material for: Evolution in an oncogenic bacterial species with extreme genome plasticity: Helicobacter pylori East Asian genomes
Source: BMC Microbiol. 2011 May 16;11:104. doi: 10.1186/1471-2180-11-104 (PMC3120642; doi:10.1186/1471-2180-11-104)
Supplement: Additional file 6 — Multiple sequence alignments of diverged genes. [file 1471-2180-11-104-S6.ZIP › Diverged_genes_multiple_seuence_alignments/HP0638_hopH.mfa.rtf]

                 1         11        21        31        41        51        61        71        81        91                         |         |         |         |         |         |         |         |         |         |         HB8:HPB8_838     M-----------------KKALLLTLSLSLSFWLHAERNGFYLGLNFAEGSYIQGQGSIGEKASAENALNEAINNAKNSLFP-EQNTKAIRDAQNALNEVHHPA:HPAG1_0621  M-----------------KKALL--LSLFLSFWLHAERNGFYLGLNFAEGSYIQGQGSIGEKASAENALNQAINNAKNSLFP-EQNTKAIRNAQNALNEVH266:HP0638      M-----------------KKALL--LTLSLSFWLHAERNGFYLGLNFLEGSYIKGQGSIGKKASAENALNEAINNAKNSLFP---NTKAIRDAQNALNAVHG27:HPG27_599   M-----------------KKALL--LTLSLSFWLHAERNGFYLGLNFLEGSYIKGQGSIGKKASAENALNEAINNAKNSLFP-EQNTKAIRDAQNALNAVHP12:HPP12_0650  M-----------------KKALL--LSLFLSFWLHAERNGFYLGLNFAEGSYIKGQGSIGKKASAENALNQAINNAKNSLFPTTQNTKAIRDAQNALNAVHF32:HPF32_0615  M-----------------KKALL--LTLFFSFWLHAERNGFYLGLNFAEGSYIKGQGSIGEKASAENALNEAINNAKNSVFP-EQNTKAIRDAQNALNKVHF16:HPF16_0648  M-----------------KKALL--LTLFFSFWLHAERNGFYLGLNFAEGSYIKGQGSIGEKASAENALNQAINNAKNSLFP-EQNTKAIRDAQNALNEVHF16:HPF16_0722  M-----------------KKALL--LTLFFSFWLHAERNGFYLGLNFAEGSYIKGQGSIGEKASAENALNQAINNAKNSLFP-EQNTKAIRDAQNALNEVH52:HPKB_0637    M-----------------KKTLL--LTLFFSFWLHAERNGFYLGLNFAEGSYIKGQGSIGEKASAENALNQAINNAKNSLFP-EQNTKAIRDAQNALNEVH52:HPKB_0705    M-----------------KKTLL--LTLFFSFWLHAERNGFYLGLNFAEGSYIKGQGSIGEKASAENALNQAINNAKNSLFP-EQNTKAIRDAQNALNEVHF57:HPF57_0735  M-----------------KKTLL--LFLSFSFWLHAERNGFYLGLNFAEGSYIKGQGSIGEKASAENALNQAINNAKNSLFP-EQNTKAIRDAQNALNEVHF57:HPF57_0662  M-----------------KKTLL--LFLSFSFWLHAERNGFYLGLNFAEGSYIKGQGSIGEKASAENALNQAINNAKNSLFP-EQNTKAIRDAQNALNEVHF30:HPF30_0689  M-----------------KKTLL--LFLSLSFWLHAERNGFYLGLNFAEGSYIKGQGSIGKKASAENALNQAINNAKNSLFP-EQNTKAIRDAQNALNEVHF30:HPF30_0622  M-----------------KKTLL--LFLSLSFWLHAERNGFYLGLNFAEGSYIKGQGSIGKKASAENALNQAINNAKNSLFP-EQNTKAIRDAQNALNEVH51:KHP_0682     M-----------------KKTLL--LFLSFSFWLHAERNGFYLGLNFLEGSYIKGQGSIGEKASAENALNQAINNAKNSLFP-EQNTKAIRDAQNALNEVH51:KHP_0612     M-----------------KKTLL--LFLSFSFWLHAERNGFYLGLNFLEGSYIKGQGSIGEKASAENALNQAINNAKNSLFP-EQNTKAIRDAQNALNEVHB38:HELPY_0733  MIKYLIIISNLKPKKNHEKSSLT--NSLSLSFWLHAERNGFYLGLNFAEGSYIKGQGSIGKKASAENALNEAINNAKNSLFP-EQNTKAIRNAQNALNEV                 101       111       121       131       141       151       161       171       181       191                        |         |         |         |         |         |         |         |         |         |         HB8:HPB8_838     KDSTKIANRFAGNGGSGGLFNELSFGYKYFLGKKRIIGFRHSLFFSYQLGGVGSVPGSGLIVFLPYGFNTDLLINWTNDKRASQKYVERRVKGLSIFYKDHHPA:HPAG1_0621  KDSTKIANRFAGNGGSGGLFNELSFGYKYFLGKKRIIGFRHSLFFGYQLGGVGSVPGSGLIVFLPYGFNTDLLINWTNDKRASQKYVERRVKGLSIFYKDH266:HP0638      KDSNKIASRFAGNGGSGGLFNELSFGYKYFLGKKRIIGFRHSLFFGYQLGGVGSVPGSGLIVFLPYGFNTDLLINWTNDKRASQKYVERRVKGLSIFYKDHG27:HPG27_599   KDSNKIANRFAGNGGSGGLFNELSFGYKYFLGKKRIIGFRHSLFFGYQLGGVGSVPGSGLIAFLPYGFNTDLLINWTNDKRASQKYVERRVKGLSIFYKDHP12:HPP12_0650  KDSNKIASRFAGNGGSGGIFNELSLGYKYFLGKKRIIGFRHSLFFGYQLGGVGSVPGSGLIVFLPYGFNTDLLINWTNDKRASQKYVERRVKGLSIFYKDHF32:HPF32_0615  KDSTKIANRFAGNGGSGGLFNELSFGYKYFLGKKRIIGFRHSLFFGYQLGGVGSVPGSGLIVFLPYGFNTDLLINWTNDKRASQEDVERRVKGLSIFYKDHF16:HPF16_0648  KDSTKIANRFAGNGGSGGLFNELSFGYKYFLGKKRIIGFRHSLFFGYQLGGVGSVPGSGLIVFLPYGFNTDLLINWTNDKRASQEYVERRVKGLSIFYKDHF16:HPF16_0722  KDSTKIANRFAGNGGSGGLFNELSFGYKYFLGKKRIIGFRHSLFFGYQLGGVGSVPGSGLIVFLPYGFNTDLLINWTNDKRASQEYVERRVKGLSIFYKDH52:HPKB_0637    KDSTKIANRFAGNGGSGGLFNELSFGYKYFLGKKRIIGFRHSLFFGYQLGGVGSVPGSGLIVFLPYGFNTDLLINWINDKRASQEYVERRVKGLSIFYKDH52:HPKB_0705    KDSTKIANRFAGNGGSGGLFNELSFGYKYFLGKKRIIGFRHSLFFGYQLGGVGSVPGSGLIVFLPYGFNTDLLINWINDKRASQEYVERRVKGLSIFYKDHF57:HPF57_0735  KDSTKIANRFAGNGGSGGLFNELSFGYKYFLGKKRIIGFRHSLFFGYQLGGVGSVPGSGLIVFLPYGFNTDLLINWTNDKRASQEYVERRVKGLSIFYKDHF57:HPF57_0662  KDSTKIANRFAGNGGSGGLFNELSFGYKYFLGKKRIIGFRHSLFFGYQLGGVGSVPGSGLIVFLPYGFNTDLLINWTNDKRASQEYVERRVKGLSIFYKDHF30:HPF30_0689  KDSTKIANRFAGNGGSGGLFNELSFGYKYFLGKKRIIGFRHSLFFGYQLGGVGSVPGSGLIVFLPYGFNTDLLINWTNDKRASQEYVERRVKGLSIFYKDHF30:HPF30_0622  KDSTKIANRFAGNGGSGGLFNELSFGYKYFLGKKRIIGFRHSLFFGYQLGGVGSVPGSGLIVFLPYGFNTDLLINWTNDKRASQEYVERRVKGLSIFYKDH51:KHP_0682     KDSTKIANRFAGNGGSGGLFNELSFGYKYFLGKKRIIGFRHSLFFGYQLGGVGSVPGSGLIVFLPYGFNTDLLINWTNDKRASQEDVERRVKGLSIFYKDH51:KHP_0612     KDSTKIANRFAGNGGSGGLFNELSFGYKYFLGKKRIIGFRHSLFFGYQLGGVGSVPGSGLIVFLPYGFNTDLLINWTNDKRASQEDVERRVKGLSIFYKDHB38:HELPY_0733  KDSNKIANRFAGNGGSGGLFNELSLGYKYFLGKKRIIGFRHSLFFGYQLGGVGSVPGSGLIVFLPYGFNTDLLINWTNDKRASQKYVERRVKGLSIFYKD                 201       211       221       231       241       251       261       271       281       291                        |         |         |         |         |         |         |         |         |         |         HB8:HPB8_838     MTGRTLDANTLKKASRHVFRKSSGLVIGMELGGSTWFASNNLTPFNQAKSHTIFQLQGKFGVRWNNDEYDIDRYGDENYLGGSSVELGVKVPAFKVNYYSHHPA:HPAG1_0621  MTGRTLDANTLKKASRHVFRKSSGLVIGMELGGSTWFASNNLTPFNQVKSRTIFQLQGKFGVRWNNDEYDIDRYGDENYLGGSSVELGVKVPAFKVNYYSH266:HP0638      MTGRTLDANTLKKASRHVFRKSSGLVIGMELGGSTWFASNNLTPFNQVKSRTIFQLQGKFGVRWNNDEYDIDRYGDEIYLGGSSVELGVKVPAFKVNYYSHG27:HPG27_599   MTGRTLDANTLKKASRHVFRKSSGLVIGMELGGSTWFASNNLTPFNQVKSRTIFQLQGKFGVRWNNDEYDIDRYGNEIYLGGSSVELGVKVPAFKVNYYSHP12:HPP12_0650  MTGRTLDANTLKKASRHVFRKSSGLVIGMELGASTWFASNNLTPFNQVKSRTIFQLQGKFGVRWNNDEYDIDRYGDEIYLGGSSVELGVKVPAFKVNYYSHF32:HPF32_0615  MTGRTLDANTLKKVSRHIFRKSSGLVIGMDIGASTWFASNNLTPFNQVKSHTIFQLQGKFGVRYNSDEYDIDRYGDEIYLGGSSVELGVKVPAFKVNYYSHF16:HPF16_0648  MTGRTLDANTLKKVSRHIFRKSSGLVIGMDIGASTWFASNNLTPFNQVKSHTIFQLQGKFGVRYNSDEYDIDRYGDEIYLGGSSVELGVKVPAFKVNYYSHF16:HPF16_0722  MTGRTLDANTLKKVSRHIFRKSSGLVIGMDIGASTWFASNNLTPFNQVKSHTIFQLQGKFGVRYNSDEYDIDRYGDEIYLGGSSVELGVKVPAFKVNYYSH52:HPKB_0637    MTGRTLDANTLKKVSRHIFRKSSGLVIGMDIGASTWFASNNLTPFNQVKSHTIFQLQGKFGVRYNSDEYDIDRYGDEIYLGGSSVELGVKVPAFKVNYYSH52:HPKB_0705    MTGRTLDANTLKKVSRHIFRKSSGLVIGMDIGASTWFASNNLTPFNQVKSHTIFQLQGKFGVRYNSDEYDIDRYGDEIYLGGSSVELGVKVPAFKVNYYSHF57:HPF57_0735  MTGRTLDANTLKKVSRHVFRKSSGLVIGMDIGASTWFASNNLTPFNQVKSHTIFQLQGKFGVRYNSDEYDIDRYGDEIYLGGSSVELGVKVPAFKVNYYSHF57:HPF57_0662  MTGRTLDANTLKKVSRHVFRKSSGLVIGMDIGASTWFASNNLTPFNQVKSHTIFQLQGKFGVRYNSDEYDIDRYGDEIYLGGSSVELGVKVPAFKVNYYSHF30:HPF30_0689  MTGRTLDANTLKKVSRHIFRKSSGLVIGMDIGASTWFASNNLTPFNQVKSHTIFQLQGKFGVRYNSDEYDIDRYGDEIYLGGSSVELGVKVPAFKVNYYSHF30:HPF30_0622  MTGRTLDANTLKKVSRHIFRKSSGLVIGMDIGASTWFASNNLTPFNQVKSHTIFQLQGKFGVRYNSDEYDIDRYGDEIYLGGSSVELGVKVPAFKVNYYSH51:KHP_0682     MTGRTLDANTLKKVSRHVFRKSSGLVIGMDIGASTWFASNNLTPFNQVKSHTIFQLQGKFGVRYNSDEYDIDRYGDEIYLGGSSVELGVKVPAFKVNYYSH51:KHP_0612     MTGRTLDANTLKKVSRHVFRKSSGLVIGMDIGASTWFASNNLTPFNQVKSHTIFQLQGKFGVRYNSDEYDIDRYGDEIYLGGSSVELGVKVPAFKVNYYSHB38:HELPY_0733  MTGRTLDANTLKKASRHVFRKSSGLVIGMELGGSTWFASNNLTPFNQVKSRTIFQLQGKFGVRWNNDEYDIDRYGDEIYLGGSSVELGVKVPAFKVNYYS                 301       311       321                 |         |         |HB8:HPB8_838     DNYGDKLDYKRVVSVYLNYTYNFKNKHHHPA:HPAG1_0621  DNYGDKLDYKRVVSVYLNYTYNFKNKHH266:HP0638      DDYGDKLDYKRVVSVYLNYTYNFKNKHHG27:HPG27_599   DDYGDKLDYKRVVSVYLNYTYNFKNKHHP12:HPP12_0650  DNYGDKLDYKRVVSVYLNYTYNFKH--HF32:HPF32_0615  DNYGDKLDYKRVVSVYLNYTYNFK---HF16:HPF16_0648  DNYGDKLDYKRVVSVYLNYTYNFK---HF16:HPF16_0722  DNYGDKLDYKRVVSVYLNYTYNFK---H52:HPKB_0637    DNYGDKLDYKRVVSVYLNYTYNFK---H52:HPKB_0705    DNYGDKLDYKRVVSVYLNYTYNFK---HF57:HPF57_0735  DNYGDKLDYKRVVSVYLNYTYNFK---HF57:HPF57_0662  DNYGDKLDYKRVVSVYLNYTYNFK---HF30:HPF30_0689  DNYGDKLDYKRVVSVYLNYTYNFK---HF30:HPF30_0622  DNYGDKLNYKRVVSVYLNYTYNFK---H51:KHP_0682     DNYGDKLDYKRVVSVYLNYTYNFK---H51:KHP_0612     DNYGDKLDYKRVVSVYLNYTYNFK---HB38:HELPY_0733  DNYGDKLDYKRVVSVYLNYTYTFRRKH
