# Supplementary material for: Evolution in an oncogenic bacterial species with extreme genome plasticity: Helicobacter pylori East Asian genomes
Source: BMC Microbiol. 2011 May 16;11:104. doi: 10.1186/1471-2180-11-104 (PMC3120642; doi:10.1186/1471-2180-11-104)
Supplement: Additional file 6 — Multiple sequence alignments of diverged genes. [file 1471-2180-11-104-S6.ZIP › Diverged_genes_multiple_seuence_alignments/HP0642_frxA.mfa.rtf]

                  1         11        21        31        41        51        61        71        81        91                          |         |         |         |         |         |         |         |         |         |         HB8:HPB8_844      MD-------REQVIALQHQRFAAKKYDPNRRISQKDWEALVEVGRLAPSSIGLEPWKMLLLKNERMKEDLKPMAWGALSSLEGASHFVIYLARKGVTYDSHSJM:HPSJM_03270  MD-------REQVVALQHQRFAAKKYDPNRRISQKDWEALVEVGRLAPSSIGLEPWKMLLLKNERMKEDLKPMAWGALFGLEGASHFVIYLARKGVTYDSH266:HP0642       MD-------REQVVALQHQRFAAKKYDPNRRISQKDWEALVEVGRLAPSSIGLEPWKMLLLKNERMKEDLKPMAWGALFGLEGASHFVIYLARKGVTYDSHP12:HPP12_0655   MD-------REQVVALQHQRFAAKKYDPNRRISQKDWEALVEVGRLAPSSIGLEPWKMLLLKNERMKEDLKPMAWGALFGLEGASHFVIYLARKGVTYDSHB38:HELPY_0729   MD-------REQVIALQHQRFAAKKYDPNRRISQKDWEALVEVGRLAPSSIGLEPWKMLLLKNERMKEDLKPMAWGALFGLEGASHFVIYLARKGVTYDSHHPA:HPAG1_0627   ---------------------------------------LVEVGRLAPSSIGLEPWKMLLLKNERMKEDLKPMAWGALFGLEGASHFVIYLARKGVTYDSHG27:HPG27_604    L--------------LYSTNDLLQKYDPNRRISQKDWEALVEVGRLAPSSIGLEPWKMLLLKNERMKEDLKPMAWGALFGLEGASHFVIYLARKGVTYDSHF32:HPF32_0677   MD-------REQVIALQHQRFATKKYDPNRRISQKDWEALVEVGRLAPSAFGLEPWKMLLLKNERMKEDLKPMAWGALSSLEGASHFVIYLARKGVTYDNHF57:HPF57_0665   MD-------REQVVALQHQRFATKKYDPNRRISQKDWEALVEVGRLAPSAFGLEPWKMLLLKNERMKEDLKPMAWGALSSLEGASHFVIYLARKGVTYDNH51:KHP_0679      MD-------REQVVALQHQRFATKKYDPNRRISQKDWEVLVEVGRLAPSAFGLEPWKMLLLKNERMKEDLKPMAWGALSSLEGASHFVIYLARKSVTYDNH52:HPKB_0702     MD-------REQVVALQHQRFATKKYDPNRRISQKDWEVLVEVGRLAPSAFGLEPWKMLLLKNERMKEDLKLMAWGALSSLEGASHFVIYLARKGVTYDSHF16:HPF16_0651   ---------------------------------------LVEVGRLAPSAFGLEPWKMLLLKNERMKEDLKPMAWGALSSLEGASHFVIYLARKGVTYDNHF30:HPF30_0686   MTTQNLDECHKSFILKQKIRFKCIGYG-SRLISQKDWEVLVEVGRLAPSAFGLEPWKMLLLKNERMKEDLKPMAWGALSSLEGASHFVIYLARKGVTYDN                  101       111       121       131       141       151       161       171       181       191                         |         |         |         |         |         |         |         |         |         |         HB8:HPB8_844      DYVKKVMHEVKKRDYDTNSRFAQIIKNFQESDMKLNSERSLFDWASKQTYIQMANMMMAAAMLGIDSCPIEGYDQEKVEAYLEEKGYLNMAEFGVSVMASHSJM:HPSJM_03270  DYVKKVMHEVKKRDYDTNSRFAQIIKNFQENDMKLNSERSLFDWASKQTYIQMANMMMAAAMLGIDSCPIEGYDQEKVEAYLEEKGYLNTAEFGVSVMACH266:HP0642       DYVKKVMHEVKKRDYDTNSRFAQIIKNFQENDMKLNSERSLFDWASKQTYIQMANMMMAAAMLGIDSCPIEGYDQEKVEAYLEEKGYLNTAEFGVSVMACHP12:HPP12_0655   DYVKKVMHEVKKRDYDTNSRFAQIIKNFQENDMKLNSERSLFDWASKQTYIQMANMMMAAAMLGIDSCPIEGYDQEKVEAYLEEKGYLNTAEFGVSVMACHB38:HELPY_0729   DYVKKVIHEVKKRDYDTNSRFAQIIKNFQENDMKLNSERSLFDWASKQTYIQMANMMMAAAMLGIDSCPIEGYDQEKVEAYLEEKGYLNTAEFGVSVMACHHPA:HPAG1_0627   DYVKKVMHEVKKRDYDTNSRFAQIIKNFQENDMKLNSERSLFDWASKQTYIQMANMMMAAAMLGIDSCPIEGYDQEKVEAYLEEKGYLNTAEFGVSVMACHG27:HPG27_604    DYVKKVMHEVKKRDYDTNSRFAQIIKNFQENDMKLNSERSLFDWASKQTYIQMANMMMAAAMLGIDSCPIEGYDQEKVEAYLEEKGYLNTVEFGVSVMACHF32:HPF32_0677   DYVKKVMHEVKKRDYDTDSRFAQMIKNFQESDMKLNSERSLFDWASKQTYIQMANMMMAAAMLGIDSCPIEGYDQEKVEAYLKEKGYLNTVEFGVSVMASHF57:HPF57_0665   DYVKKVMHEVKKRDYDTDSRFAQMIKNFQESDMKLNSERSLFDWASKQTYIQMANMMMAAAMLGIDSCPIEGYDQEKVEAYLKEKGYLNTAEFGVSVMASH51:KHP_0679      DYVKKVMHEVKKRDYDTDSRFAQMIKNFQESDMKLNSERSLFDWASKQTYIQMANMMMAAAMLGIDSCPIEGYDQEKVEAYLKEKGYLNTAEFGVSVMASH52:HPKB_0702     DYVKKVMHEVKKRDYDTDSRFAQMIKNFQESDMKLNSERSLFDWASKQTYIQMANMMMAAAMLGIDSCPIEGYDQEKVEAYLKEKGYLNTAEFGVSVMASHF16:HPF16_0651   DYVKKVMHEVKKRDYDTDSRFAQMIKNFQESDMKLNSERSLFDWASKQTYIQMANMMMAAAMLGIDSCPIEGYDQEKVEAYLKEKGYLNTAEFGVSVMASHF30:HPF30_0686   DYVKKVMHEIKKRDYDTDSRFAQMIKNFQESDMKLNSERSLFDWASKQTYIQMANMMMAAAMLGIDSCPIEGYDQEKVEAYLKEKGYLNTAEFGVSVMAS                  201       211       221                  |         |         |HB8:HPB8_844      FGYRNQEITPKTRWKTEVIYEVIEHSJM:HPSJM_03270  FGYRNQEITPKTHWKTEVIYEVIEH266:HP0642       FGYRNQEITPKTRWKTEVIYEVIEHP12:HPP12_0655   FGYRNQEITPKTRWKTEVIYEVIEHB38:HELPY_0729   FGYRNQEITPKTRWKTEVIYEVIEHHPA:HPAG1_0627   FGYRNQEITPKTRWKTEVIYEVIEHG27:HPG27_604    FGYRNQEITPKTRWKTEVIYEVIEHF32:HPF32_0677   FGYRNQEITPKTRWKTEVIYEVIEHF57:HPF57_0665   FGYRNQEITPKTRWKTEVIYEVIEH51:KHP_0679      FGYRNQEITPKTRWKTEVIYEVIEH52:HPKB_0702     FGYRNQEITPKTRWKTEVIYEVIEHF16:HPF16_0651   FGYRNQEITPKTRWKTEVIYEVIEHF30:HPF30_0686   FGYRNQEITPKTRWKTEVIYEVIE
