# Supplementary material for: Evolution in an oncogenic bacterial species with extreme genome plasticity: Helicobacter pylori East Asian genomes
Source: BMC Microbiol. 2011 May 16;11:104. doi: 10.1186/1471-2180-11-104 (PMC3120642; doi:10.1186/1471-2180-11-104)
Supplement: Additional file 6 — Multiple sequence alignments of diverged genes. [file 1471-2180-11-104-S6.ZIP › Diverged_genes_multiple_seuence_alignments/HP0651_HP0379_fucT.mfa.rtf]

                  1         11        21        31        41        51        61        71        81        91                          |         |         |         |         |         |         |         |         |         |         HF32:HPF32_0668   ----------------------------------VANWF----NGPKEF-KANILYFILKQRYKIILHNNPNEPSDLVFGNPLGQARKILSYQNTKRVFYHF16:HPF16_0660   ----------------------------------VANWF----GGVKEF-KASVLYFILKQRYKIILHSNPNEPSDLVFGNPLEQARKILSYQNTKRVFYHF57:HPF57_1038   MFQPLLDAFIESTSIKKKLPLISPP---PPLKIAVANWF----NGPKEF-KANILYFILKQRYKIILHSNPNESSDLVFGNPLEQARKILSYQNTKRVFYH51:KHP_0670      MFQPLLDAFIESASIKKKLPL-NLP---PPLKIAVANWF----NGPKEF-KANILYFILKQRYKIILHNNPNEPSDLVFGNPLEQARKILSYQNTKRVFYH52:HPKB_0693     ----------------------------------VANWF----NGPKEF-KANILYFILKQRYKIILHQNPNEPSDLVFGNPLEQARKILSYQNTKRVFYHF30:HPF30_0312   ----------------------------------VANWF----NGPKEF-KASVLYFILKQRYKIILHNNPNEPSDLVFGSPLGQARKILSYQNTKRVFYHF32:HPF32_0329   MFQPLLDAFIDSTHLDET----THK---PPLNIALANWWPLKNSEKKGF-RDFILYFILKQRYKIILHNNPNEPSDLVFGNPLGQARKILSYQNTKRVFYHF57:HPF57_0674   MFQPLLDAFIDSTHLDET----THK---PPLNIALANWWPLKNSEKKGF-RDSILHVILKQRYKIILHSNPNESSDLVFGNPLEQARKILSYQNTKRVFYHF16:HPF16_1018   MFQPLLDAFIDSTHLDGT----THK---PPLNIALANWWPLKNSEKKGF-RDFILHVILKQRYKIILHSNPNEPSDLVFGNPLEQARKILSYQNTKRVFYH52:HPKB_1004     MFQPLLDAYTDSTHLDET----THK---PPLNIALANWWPLKNSEKKGF-RDFILHVILKQRYKIILHQNPNEPSDLVFGNPLEQARKILSYQNTKRVFYHF30:HPF30_0677   MFQPLLDAYTDSTHLDGT----THK---PPLNIALANWWPLKNSEKKGF-RDFILHVILKQRYKIILHNNPNEPSDLVFGSPLGQARKILSYQNTKRVFYH51:KHP_0977      MFQPLLDAFIDSTYLDET----THK---PPLNIALANWWPLKNSEKKGF-RDFILHVILKQRYKIILHNNPNEPSDLVFGNPLEQARKILSYQNTKRVFYHSJM:HPSJM_05295  ----------------------------------VANWWGG----TKEF-KKSALYFILSQRYKITLHQNPNEPSDLVFGSPIGAARKILSYQNAKRVFYHSJM:HPSJM_03315  MFQPLLDAYIDSTRLDET----DYK---PPLNIALANWWPLDKRESKGFRKKFILHFILSQHYTITLHRNPDKPADIVFGNPLGSARKILSYQNAKRVFYHB38:HELPY_0720   MFQPLLDAYIDSTQIEET----THK---PPLNAALANWWPLDKRESKGFRKKFILYFILSQHYTITLHQNPNKPADLVFGNPLGSARKILSYQNTKRVFYHG27:HPG27_613    MFQPLLDAYIDSTQIEEI----THK---PPLNIALANWWPLDKRESKGFRKKFILHFILSQHYTITLHQNPNKPSDLVFSNPLGSARKILSYKNTKRVFYHP12:HPP12_0664   MFQPLLDAFIESTQIEET----THK---PPLNIALANWWPLKNSEKKGF-RDFILHVILKQRYKITLHQNPNELSDLVFGSPLGSARKILSYQNAKRVFYHHPA:HPAG1_1013   MFQPLLDAYIDSTRLDET----DYK---PPLNIALANWWPLDKRESKGFRKKFILHFILSQRYTITLHQNPNEPSDLVFGSPIGAARKILSYQNAKRVFYHB38:HELPY_1046   MFQPLLDAYIESAPIKKK----LSLNLPPPLKIAVANWWGG----AEEF-KKSTLYFILSQHYTITLHQNPNEPSDLVFGSPIGSARKILSYQNTKRVFYHP12:HPP12_1042   MFQPLLDAFIESASIEKM----ASKS-PPPLKIAVANWWGG----AEEF-KKSALYFILSQRYKITLHQNPNELSDLVFGSPLGSARKILSYQNAKRVFYHG27:HPG27_1018   MFQPLLDAFIESASIEKI----TSKS-PPPLKIAVANWWGG----AEEF-KKSALYFILSQRYKITLHQNPNEPSDLVFGSPIGAARKILSYKNTKRVFYHHPA:HPAG1_0636   ----------------------------------VANWW--GDLEVKEF-KKSVLYFILSQRYKIILHRNPDKPADIVFGSPFGSARKILSYQNAKRVFYH266:HP0651       MFQPLLDAFIESASIEKM----VSKSPPPPLKIAVANWW--GDEEIKEF-KKSVLYFILSQRYAITLHQNPNESSDLVFSNPLGAARKILSYQNTKRVFYH266:HP0379       MFQPLLDAFIESASIEKM----ASKSPPPPLKIAVANWW--GDEEIKEF-KKSVLYFILSQRYAITLHQNPNEFSDLVFSNPLGAARKILSYQNTKRVFY                  101       111       121       131       141       151       161       171       181       191                         |         |         |         |         |         |         |         |         |         |         HF32:HPF32_0668   TGENEAPNFNLFDYAIGFDELDFNDRYLRMPLYYAYLHYKAEIVNDTTSPYKLKADSLYTLKKPSHKFKENHPHLCALIHNESDPLKRGFVSFVASNPNAHF16:HPF16_0660   TGENEAPNFNLFDYAIGFDELDFNDRYLRMPLYYAYLHYKAEIVNDTTSPYKLKTDSLYTLKKPSHKFKENHPHLCALIHNESDPLKRGFASFVASNANAHF57:HPF57_1038   TGENEAPNFNLFDYAIGFDELDFNDRYLRMPLYYAYLHYKAMLVNDTTSPYKLKTDSLYTLKKPSHKFKENHPHLCALIHNESDPLKREFASFVASNANAH51:KHP_0670      TGENEAPNFNLFDYAIGFDELDFNDRYLRMPLYYAYLHYKAMLVNDTTSPYKLKTDSLYTLKKPSHKFKENHPHLCALIHNESDPLKRGFASFVASNANAH52:HPKB_0693     TGENEAPNFNLFDYAIGFDELEFNDRYLRMPLYYAYLHYKAEIVNDTTSPYKLKADSLYTLKKPSHKFKENHPHLCALIHNESDPLKRGFASFVASNANAHF30:HPF30_0312   TGENEAPNFNLFDYAIGFDELDFNDRYLRMPLYYAYLHYKAEIVNDTTSPYKLKTDSLYTLKKPSHKFKENHPNLCALINNESDPLKRGFASFVASNANAHF32:HPF32_0329   TGENEAPNFNLFDYAIGFDELDFNDRYLRMPLYYAYLHYKAEIVNDTTSPYKLKADSLYTLKKPSHKFKENHPHLCALIHNESDPLKRGFVSFVASNPNAHF57:HPF57_0674   TGENEAPNFNLFDYAIGFDELDFNDRYLRMPLYYAYLHYKAMLVNDTTSPYKLKTDSLYTLKKPSHKFKENHPHLCALIHNESDPLKREFASFVASNANAHF16:HPF16_1018   TGENEAPNFNLFDYAIGFDELDFNDRYLRMPLYYAYLHYKAEIVNDTTSPYKLKTDSLYTLKKPSHKFKENHPHLCALIHNESDPLKRGFASFVASNANAH52:HPKB_1004     TGENEAPNFNLFDYAIGFDELDFNDRYLRMPLYYAYLHYKAEIVNDTTSPYKLKADSLYTLKKPSHKFKENHPHLCALIHNESDPLKRGFASFVASNANAHF30:HPF30_0677   TGENEAPNFNLFDYAIGFDELDFNDRYLRMPLYYAYLHYKAEIVNDTTSPYKLKTDSLYTLKKPSHKFKENHPNLCALINNESDPLKRGFASFVASNANAH51:KHP_0977      TGENEAPNFNLFDYAIGFDELDFNDRYLRMPLYYAYLHYKAMLVNDTTSPYKLKTDSLYTLKKPSHKFRENHPHLCALIHNESDPLKRGFASFVASNANAHSJM:HPSJM_05295  TGENEVPNFNLFDYAIGFDELNFNDRYLRMPLYYAYLHYKAEIVNDTTSPYKLKADSLYALKKPSHKFKENHPHLCALINNEIDPLKRGFASFVASNANAHSJM:HPSJM_03315  TGENEVPNFNLFDYAIGFDELDFRDRYLRMPLYYDRLHHKAESVNDTTAPYKIKSDSLYTLKKPSHHFKENHPHLCAVVNDESDPLKRGFASFVASNANAHB38:HELPY_0720   TGENEVPNFNLFDYAIGFDELDFRDRYLRMPLYYDRLHHKAESVNDTTAPYKIKDNSLYALKKPSHHFKENHPHLCAVVNNESDPLKRGFASFVASNPNAHG27:HPG27_613    TGENEVPNFNLFDYAIGFDELDFNNRYLRMPLYYDRLHHKAESVNDTTSPYKLKDNSLYTLKKPTHHFKENHPNLCAVVNNESDPLKRGFASFVASNPNAHP12:HPP12_0664   TGENEVPNFNLFDYAIGFDELDFNDRYLRMPLYYASLHYKAESVNDTTAPYKIKDNSLYALKKPSHHFKENHPNLCAVVNNESDPLKRGFASFVASNPNAHHPA:HPAG1_1013   TGENEVPNFNLFDYAIGFDELDFNNRYLRMPLYYDRLHHKAESVNDTTSPYKLKADSLYALKKPSHHFKEKHPNLCAVVNDESDPLKRGFASFVASNPNAHB38:HELPY_1046   TGENEVPNFNLFDYAIGFDELDFRDRYLRMPLYYDRLHHKAESVNDTTAPYKLKDNSLYALKKPSHHFKENHPHLCAVVNNESDPLKRGFASFVASNPNAHP12:HPP12_1042   TGENEVPNFNLFDYAIGFDELDFNDRYLRMPLYYASLHYKAESVNDTTAPYKIKDNSLYALKKPSHHFKENHPNLCAVVNNESDPLKRGFASFVASNPNAHG27:HPG27_1018   TGENEVPNFNLFDYAIGFDELDFRDRYLRMPLYYASLHYKAESMNDTTVPYKLKDNSLYTLKKPSHHFKENHPNLYAVVNNEIDPLKRGFASFVASNPNAHHPA:HPAG1_0636   TGENEVPNFNLFDYAIGFDELDFNNRYLRMPLYYASLHYKAQGVNDTTSPYKLKADSLYALKKPSHCFKEKHPNLCAVVNDESDPLKRGFASFVASNPNAH266:HP0651       TGENESPNFNLFDYAIGFDELDFNDRYLRMPLYYAHLHYEAELVNDTTAPYKLKDNSLYALKKPSHHFKENHPNLCAVVNDESDLLKRGFASFVASNANAH266:HP0379       TGENESPNFNLFDYAIGFDELDFNDRYLRMPLYYAHLHYKAELVNDTTAPYKLKDNSLYALKKPSHHFKENHPNLCAVVNDESDLLKRGFASFVASNANA                  201       211       221       231       241       251       261       271       281       291                         |         |         |         |         |         |         |         |         |         |         HF32:HPF32_0668   PIRNAFYDALNSIEPVAGGGAVKNTLGYKVKNKNEFLSQYKFNLCFENSQGYGYVTEKILDAYFSHTIPIYWGSPSVAKDFNPKSFVNVHDFKNFDEAIDHF16:HPF16_0660   PIRNAFYDALNSIEPVAGGGSVKNTLGYKVKNKNEFLSQYKFNLCFENSQGYGYVTEKILDAYFSHTIPIYWGSPSVAKDFNPKSFVNVHDFKNFDEAIDHF57:HPF57_1038   PIRNAFYDALNAIEPVAGGGSVKNTLGYKVKNKNEFLSQYKFNLCFENSQGYGYVTEKILDAYFSHTIPIYWGSPSVAKDFNPKSFVNVHDFKNFDEAIDH51:KHP_0670      PIRNAFYDALNAIEPVAGGGAVKNTLGYKVKNKNEFLSQYKFNLCFENSQGYGYVTEKILDAYFSHTIPIYWGSPSVAKDFNPKSFVNVHDFKNFDEAIDH52:HPKB_0693     PVRNAFYEALNSIEPVAGGGSVKNTLGYNVKNKNEFLSQYKFNLCFENSQGYGYVTEKILDAYFSHTIPIYWGSPSVAKDFNPKSFVNVHDFKNFDEAIDHF30:HPF30_0312   PIRNAFYDALNSIEPVTGGGSVKNTLGYKVKNKNEFLSQYKFNLCFENSQGYGYVTEKILDAYFSHTIPIYWGSPSVAKDFNPKSFVNVHDFKNFDEAIDHF32:HPF32_0329   PIRNAFYDALNSIEPVAGGGAVKNTLGYKVKNKNEFLSQYKFNLCFENSQGYGYVTEKILDAYFSHTIPIYWGSPSVAKDFNPKSFVNVHDFKNFDEAIDHF57:HPF57_0674   PIRNAFYDALNAIEPVAGGGSVKNTLGYKVKNKNEFLSQYKFNLCFENSQGYGYVTEKILDAYFSHTIPIYWGSPSVAKDFNPKSFVNVHDFKNFDEAIDHF16:HPF16_1018   PIRNAFYDALNSIEPVAGGGSVKNTLGYKVKNKNEFLSQYKFNLCFENSQGYGYVTEKILDAYFSHTIPIYWGSPSVAKDFNPKSFVNVHDFKNFDEAIDH52:HPKB_1004     PVRNAFYEALNSIEPVAGGGSVKNTLGYNVKNKNEFLSQYKFNLCFENSQGYGYVTEKILDAYFSHTIPIYWGSPSVAKDFNPKSFVNVHDFKNFDEAIDHF30:HPF30_0677   PIRNAFYDALNSIEPVTGGGSVKNTLGYKVKNKNEFLSQYKFNLCFENSQGYGYVTEKILDAYFSHTIPIYWGSPSVAKDFNPKSFVNVHDFKNFDEAIDH51:KHP_0977      PIRNAFYDALNAIEPVTGGGSVKNTLGYKVKNKNEFLSQYKFNLCFENSQGYGYVTEKILDAYFSHTIPIYWGSPSVAKDFNPKSFVNVHDFKNFDEAIDHSJM:HPSJM_05295  PVRNAFYDALNSIEPVTGGGSVKNTLGYNIKNKNEFLSQYKFNLCFENTQGYGYVTEKIIDAYFSHTIPIYWGSPSVAKDFNPKSFVNVCDFKNFDEAIDHSJM:HPSJM_03315  PKRNAFYDALNSIEPVTGGGAVRNTLGYNVKNKSEFLSQYKFNLCFENTQGYGYVTEKIIDAYFSHTIPIYWGSPSVAKDFNPKSFVNVCDFKDFDEAIDHB38:HELPY_0720   PKRNAFYEALNSIEPVAGGGSVRNTLGYNVKNKSEFLSQYKFNLCFENTQGYGYVTEKIIDAYFSHTIPIYWGSPSVAKDFNPKSFVNVCDFKNFDEAIDHG27:HPG27_613    PKRNAFYEALNSIEPVTGGGSVKNTLGYNVKNKSEFLSQYKFNLCFENSQGYGYVTEKIIDAYFSHTIPIYWGSPSVAKDFNPKSFVNVCDFKNFDEAIDHP12:HPP12_0664   PKRNAFYDALNSIEPVTGGGSVRNTLGYNVKNKSEFLSQYKFNLCFENTQGYGYVTEKIIDAYFSHTIPIYWGSPSVAKDFNPKSFVNVCDFKNFDEAIDHHPA:HPAG1_1013   PKRNAFYDALNSIEPVIGGGSVRNTLGYNVKNKNEFLSQYKFNLCFENSQGYGYVTEKIIDAYFSHTIPIYWGSPSVAKDFNPKSFVNVHDFNNFDEAIDHB38:HELPY_1046   PKRNAFYEALNSIEPVAGGGSVRNTLGYNVKNKSEFLSQYKFNLCFENTQGYGYVTEKIIDAYFSHTIPIYWGSPSVAKDFNPKSFVNVCDFKNFDEAIDHP12:HPP12_1042   PKRNAFYDALNSIEPVTGGGSVRNTLGYNVKNKSEFLSQYKFNLCFENTQGYGYVTEKIIDAYFSHTIPIYWGSPSVAKDFNPKSFVNVCDFKNFDEAIDHG27:HPG27_1018   PKRNAFYDALNSIEPVTGGGSVKNTLGYNVKNKSEFLSQYKFNLCFENTQGYGYVTEKIIDAYFSHTIPIYWGSPSVAKDFNPKSFVNVCDFKNFDEAIDHHPA:HPAG1_0636   PKRNAFYDALNSIEPVTGGGSVRNTLGYNIKNKNEFLSQYKFNLCFENSQGYGYVTEKIIDAYFSHTIPIYWGSPSVAKDFNPKSFVNVHDFNNFDEAIDH266:HP0651       PMRNAFYDALNSIEPVTGGGSVRNTLGYKVGNKSEFLSQYKFNLCFENSQGYGYVTEKILDAYFSHTIPIYWGSPSVAKDFNPKSFVNVHDFNNFDEAIDH266:HP0379       PMRNAFYDALNSIEPVTGGGSVRNTLGYKVGNKSEFLSQYKFNLCFENSQGYGYVTEKILDAYFSHTIPIYWGSPSVAKDFNPKSFVNVHDFNNFDEAID                  301       311       321       331       341       351       361       371       381       391                         |         |         |         |         |         |         |         |         |         |         HF32:HPF32_0668   YIKYLHAHQNAYLDMLYENPLNTIDGKAGFYQDLSFEKILDFFKNILENDTIYH--CNDAHYSALHRDLNEPLVSV------------------------HF16:HPF16_0660   YIRYLHTHQNAYLDMLYENPLNTIDGKAGFYQDLSFEKILDFFKNILENDTIYH--CNDAHYSALHRDLNEPLVSV------------------------HF57:HPF57_1038   YIRYLHTHQNAYLDMLYENPLNTIDGKAGFYQDLSFEKILDFFKTILENDTIYH--CNDAHYSALHRDLNEPLVSV--------------DDLRRDH---H51:KHP_0670      YIRYLHTHQNAYLDMLYENPLNTIDGKAGFYQDLSFEKILDFFKSILENDTIYH--CNDAHYSALCRDLNEPLVSV--------------DDLRRDH---H52:HPKB_0693     YIRYLHAHQNAYLDMLYENPLNTIDGKAGFYQDLSFEKILDFFKNILENDTIYH------NSSALCRDLNEPLVSV------------------------HF30:HPF30_0312   YIRYLHTHKNAYLDMLYENPLNTIDGKAGFYQDLSFEKILDFFKNILENDTIYH------NPSALCRDLNEPLVSV------------------------HF32:HPF32_0329   YIKYLHAHQNAYLDMLYENPLNTIDGKAGFYQDLSFEKILDFFKNILENDTIYH--CNDAHYSALHRDLNEPLVSV--------------DGLRRDYDDLHF57:HPF57_0674   YIRYLHTHQNAYLDMLYENPLNTIDGKAGFYQDLSFEKILDFFKTILENDTIYH--CNDAHYSALHRDLNEPLVSV--------------DDLRRD----HF16:HPF16_1018   YIRYLHTHQNAYLDMLYENPLNTIDGKAGFYQDLSFEKILDFFKNILENDTIYH--CNDAHYSALHRDLNEPLVSV--------------DDLRVN----H52:HPKB_1004     YIRYLHAHPNAYLDMLYENPLNTIDGKAGFYQDLSFEKILDFFKNILENDTIYH--CNDSYYSALHRDLNEPLVSV--------------DDLRRD----HF30:HPF30_0677   YIRYLHTHQNAYLDMLYENPLNTIDGKAGFYQDLSFEKILDFFKNILENDTIYH--CNDAHYSALHRDLNEPLVSV--------------DGLRRDHDDLH51:KHP_0977      YIRYLHTHQNAYLDMLYENPLNTIDGKVGFYQDLSFEKILDFFKNILENDTIYH--CNDSCYSALCRDLNEPLVSV--------------DDLRRDH---HSJM:HPSJM_05295  YVRYLHTHPNAYLDMLYENPLNEIDGKAYFYQNLSFKKILDFFKTILENDTIYH--DNPFIF---YRDLNEPLISI------------------------HSJM:HPSJM_03315  YVRYLHAHQNAYLDMLYENPLNEIDGKAYFYQNLSFKKILDFFKTILENDTIYH--DNPFIF---YRDLNEPLISI--------------DDLRVNYDDLHB38:HELPY_0720   YIRYLHTHPNAYLDMLYENPLNTLDGKAYFYQDLSFKKILDFFKTILENDTIYH--NNPFIF---YRDLNEPLISI-------DDLRVNYDDLRVNYDDLHG27:HPG27_613    YVRYLHTHPNAYLDMLYENPLNTIDGKAGFYQDLSFKKILDFFKTILESDTIYH--DNPSTL---YRDLHEPLVSI--------------DDLRVNYDDLHP12:HPP12_0664   YVRYLHTHPNAYLDMLYENPLNTLDGKAYFYQDLSFKKILDFFKTILENDTIYH--DNPFIF---YRDLNEPLVTI--------------DDLRVNYDDLHHPA:HPAG1_1013   YVRYLHTHPNAYLDMLYENPLNTIDGKAYFYQDLSFKKILDFFKTILENDTIYH--DNPFIF---YRDLNEPLVSI---------------------DDLHB38:HELPY_1046   YIRYLHTHPNAYLDMLYENPLNTLDGKAYFYQNLSFKKILDFFKTILENDTIYH--NNPFIF---YRDLNEPLISI------------------------HP12:HPP12_1042   YVRYLHTHPNAYLDMLYENPLNTLDGKAYFYQDLSFKKILDFFKTILENDTIYH--DNPFIF---YRDLNEPLVTI------------------------HG27:HPG27_1018   YVRYLHTHPNAYLDMLYENPLNTLDGKAYFYQNLSFKKILDFFKTILENDTIYH--NNPFIF---YRDLNEPLATI------------------------HHPA:HPAG1_0636   YVRYLHTHPNAYLDMLYENPLNTIDGKAYFYQDLSFKKILDFFKTILENDTIYH--NNPFTL---YRDLNEPLVSI------------------------H266:HP0651       YIKYLHTHPNAYLDMLYENPLNTLDGKAYFYQDLSFKKILDFFKTILENDTIYH--NNPFIF---YRDLHEPLISIDDLRVNYDDLRVNYDDLRVNYDDLH266:HP0379       YIKYLHTHPNAYLDMLYENPLNTLDGKAYFYQDLSFKKILDFFKTILENDTIYHKFSTSFMW---EYDLHKPLVSI------------------------                  401       411       421       431       441       451       461       471       481       491                  |         |         |         |         |         |         |         |         |         |HF32:HPF32_0668   -------------------------DGLRRDYDDLRVNYDDLRRDHERLLSKATPLLELSQNTSFKIYRKAYQKSLPLLRAIRRWVKK---HF16:HPF16_0660   --------------------------------DDLRVNYDDLRRDHERLLSKATPLLELSQNISFKIYRKAYQKSLPLLRAIRRWVKK---HF57:HPF57_1038   -------------------------DDLRVNYDDLRVNYDDLRRDHERLLSKATPLLELSQNTSFKIYRKAYQKFLPLLRAIRRWVKK---H51:KHP_0670      -------------------------DDLRRDHDDLRRDHDDLRRDHERLLSKATPLLELSQNTSFKIYRKAYQKSLPLLRAIRRWVRK---H52:HPKB_0693     ---------------------------------------DDLRRDHERLLSKATPLLELSQNTSFKIYRKAYQKSLPLLRAIRRWVRK---HF30:HPF30_0312   --------------------------------DGLRRDHDDLRRDHERLLSKATPLLELSQNTSFKIYRKAYQKSLPLLRAIRRWVRK---HF32:HPF32_0329   RVNYDDLRVNYDDLRVNYDDLRVNYDDLRVNYDDLRVNYDDLRRDHEHLLSKATPLLELSQNTSFKIYRKAYQKSLPLLRAIRRWVKK---HF57:HPF57_0674   ----------HDDLRVNYDDLRVNYDDLRVNYDDLRVNYDDLRRDHERLLSKATPLLELSQNTSFKIYRKAYQKFLPLLRAIRRWVRK---HF16:HPF16_1018   ----------YDDLRVNYDDLRVNYDDLRVNYDDLRVNYDDLRRDHERLLSKATPLLELSQNISFKIYRKAYQKSLPLLRAIRRWVKK---H52:HPKB_1004     ----------HDDLRVNYDDLRVNYDDLRVNYDDLRVNYDDLRRDHERLLSKATPLLELSQNTSFKIYRKAYQKSLPLLRAIRRWVRK---HF30:HPF30_0677   RVNYDDLRVNYDDLRVNYDDLRVNYDDLRVNYDDLRVNYDDLRRDHERLLSKATPLLELSQNTSFKIYRKIYQKSLPLLRAIRGWVKK---H51:KHP_0977      ----DDLRRDHDDLRRDHDDLRRDHDDLRRDHDDLRRDHDDLRRDHERLLSKATPLLELSQNTSFKIYRKAYQKSLPLLRAIRRWVRK---HSJM:HPSJM_05295  -----------DNLRADYNNLRADY-------NNLRADYNNLRADYDRLLQNASPLLELSQNTSFKIYRKAYQKSLPLLHAIRRWVKKLGLHSJM:HPSJM_03315  RVNYDDLRVNYDDLRVNYDDLRVNYDDLRVNYDDLRVNY-------DRLLQNASPLLELSQNTTFKIYRKAYQKSLPLLRTIRRWVKK---HB38:HELPY_0720   RVNYDDLRVNYDDLRVNYDDLRVNYDDLRVNYDDLRVNYDDLRVNYERLLQNASPLLELSQNTTFKIYRKIYQKSLPLLRAIRRWVKK---HG27:HPG27_613    RVNYDDLRVNYDDLRVNYDDLRVNYDDLRVNYDDLRVNYDDLRVNYERLLQNASPLLELSQNTTFKIYRKAYQKSLPLLRTIRRWVKK---HP12:HPP12_0664   RVNYDDLRVNYDDLRVNYDDLRVNYDDLRVNYDDLRVNYDDLRVNYERLLQNASPLLELSQNTTFKIYRKIYQKSLPLLRVIRRWVNK---HHPA:HPAG1_1013   RVNYDDLRVNYDDLRVNYDDLRVNYDDLRVNYDDLRVNYDDLRINYERLLQNASPLLELSQNTSFKIYRKAYQKSLPLLCAVRKWVKKLGLHB38:HELPY_1046   -----------DDLRVNYDDLRVNYDDLRVNYDDLRVNYDDLRVNYERLLQNASPLLELSQNTTFKIYRKAYQKSLPLLRAIRKLIKKLGLHP12:HPP12_1042   ------------------DDLRVNYDDLRVNYDDLRVNYDDLRVNYERLLQNASPLLELSQNTTFKIYRKIYQKSLPLLRVIRRWVKKLGLHG27:HPG27_1018   ----DDLRVNYDDLRVNYDDLRVNYDDLRVNYDDLRVNYDDLRVNYERLLQNASPLLELSQNTTFKIYRKIYQKSLPLLRAIRRWVKK---HHPA:HPAG1_0636   -----------DDLRVNYDDLRVNYDDLRVNYDDLRVNYDDLRVNYERLLQNASPLLELSQNTSFKIYRKAYQKSLPLLRAIRRWVKK---H266:HP0651       RVNYDDLRVNYDDLRVNYDDLRVNYDDLRVNYDDLRVNYDDLRVNYDRLLQNASPLLELSQNTTFKIYRKAYQKSLPLLRTIRRWVKK---H266:HP0379       --------------------------------DDLRVNYDDLRVNYDRLLQNASPLLELSQNTTFKIYRKAYQKSLPLLRAVRKLVKKLGL
