# Supplementary material for: Evolution in an oncogenic bacterial species with extreme genome plasticity: Helicobacter pylori East Asian genomes
Source: BMC Microbiol. 2011 May 16;11:104. doi: 10.1186/1471-2180-11-104 (PMC3120642; doi:10.1186/1471-2180-11-104)
Supplement: Additional file 6 — Multiple sequence alignments of diverged genes. [file 1471-2180-11-104-S6.ZIP › Diverged_genes_multiple_seuence_alignments/HP0661_rnhA.mfa.rtf]

                  1         11        21        31        41        51        61        71        81        91                          |         |         |         |         |         |         |         |         |         |         HB8:HPB8_864      MQEIEIFCDGSSLGNPGPGGYAAILRYKDKEKIISGGENFTTNNRMELRALNEALKILKRPCHITLYSDSQYVCQAINVWLANWQKKNFSKVKNVDLWKEHB38:HELPY_0710   MQEIEIFCDGSSLGNPGPGGYAAILRYKDKEKIISGGEHFTTNNRMELRALNEALKVLKRPCHITLYSDSQYVCQAINVWLANWQKKNFSKVKNVDLWKEHG27:mHPG27_623   MQEIEIFCDGSSLGNPGPGGYAAILRYKDKEKIISGGEHFTTNNRMELRALNEALKILKRPCHITLYSDSQYVCQAINVWLANWQKKNFSKVKNVDLWKEHSJM:HPSJM_03365  MQEIEIFCDGSSLGNPGPGGYAAILRYKDKEKIISGGEHFTTNNRMELRALNEALKVLKRPCHITLYSDSQYVCQAINVWLINWQKKNFAKVKNVDLWKEH266:HP0661       MQEIEIFCDGSSLGNPGPGGYAAILRYKDKEKTISGGEEFTTNNRMELRALNEALKILKRPCRITLYSDSQYVCQAINVWLANWQKKNFSKVKNVDLWKEHHPA:HPAG1_0646   MQEIEIFCDGSSLGNPGPGGYAAILRYKDKEKTISGGEEFTTNNRMELRALNEALKILKRPCRITLYSDSQYVCQAINVWLVNWQKKNFSKVKNVDLWKEHF32:HPF32_0658   MQEIEIFCDGSSLGNPGPGGYAAILRYKDKEKIISGGESFTTNNRMELKALNEALKILKRPCHITLYSDSQYVCQAINVWLANWQKKNFSKVKNMDLWKEHF57:HPF57_0684   MQEIEIFCDGSSLGNPGPGGYAAILRYKDKEKIISGGESFTTNNRMELKALNEALKILKRPCHITLYSDSQYVCQAINVWLANWQKKNFSKVKNVDLWKEHF16:HPF16_0670   MQEIEIFCDGSSLGNPGPGGYAAILRYKDKEKVISGGESFTTNNRMELKALNEALKILKRPCHITLYSDSQYVCQAINVWLINWQKKNFSKVKNVDLWKEH51:KHP_0660      MQEIEIFCDGSSLGNPGPGGYAAILRYKDKEKIISGGESFTTNNRMELKALNEALKILKRPCRITLYSDSQYVCQAINVWLINWQKKNFSKVKNVDLWKEH52:HPKB_0683     MQEIEIFCDGSSLGNPGPGGYAAILRYKDKEKIISGGESFTTNNRMELRALNEALKILKRPCRITLYSDSQYVCQAINVWLVNWQKKNFSKVKNVDLWKEHF30:HPF30_0667   MQEIEIFCDGSSLGNPGPGGYAAILRYKDKEKIISGGESFTTNNRMELKALNEALKILKRPCRITLYSDSQYVCQAINVWLTNWQKKNFSKVKNVDLWKEHP12:mHPP12_0674  ---LKFFAMVLHLGNPGPGGYAAILRYKDKEKIISGGEHFTTNNRMELRALNEALKILKRPCHITLYSDSQYVCQAINVWLANWQKKNFSKVKNVDLWKE                  101       111       121       131       141       151                  |         |         |         |         |         |HB8:HPB8_864      FLKVSKGHLIVAVWIKGHNGHAENERCDSLAKLEAQKRVKTT-----------THB38:HELPY_0710   FVKVSKGHLIVAVWIKGHNGHAENERCDSLAKLEAQKRTKTT-----------THG27:mHPG27_623   FLKVSKGHLIVAVWIKGHNGHAENERCDSLAKLEAQKRTKTT-----------THSJM:HPSJM_03365  FLKVSKGHLIVAVWIKGHNGHAENERCDSLAKLEAQKRVKTT-----------TH266:HP0661       FLEVSKGHSIVAVWIKGHNGHAENERCDSLAKLEAQKRVKTT-----------THHPA:HPAG1_0646   FLKVSKGHLIMAVWIKGHNGHAENERCDSLAKLEAQKRTKTI-----------THF32:HPF32_0658   FLEVSKGHLIMAIWIKGHNGHAENERCDSLAKLEAQKRVKTIKGKNDEKQTLSKHF57:HPF57_0684   FLEVSKGHLIMAVWIKGHNGHAENERCDSLAKLEAQKRIKTTKGKNDEKQTLSKHF16:HPF16_0670   FLEVSKGHLIMAVWIKGHNGHAENERCDSLAKIEAQKRTKTTKGKNDEKQTLSKH51:KHP_0660      FLEVSKGHLIMAVWIKGHNGHAENERCDSLAKIEALKRTKTTKGKNDEKQTLSKH52:HPKB_0683     FLEVSKGHLIMAVWIKGHNGHAENERCDSLAKIEAQKRTKTTKGKNDEKQTLSKHF30:HPF30_0667   FLEVSKGHLIMAVWIKGHNGHVENERCDSLAKIEAQKRTKTTKGKNDEKQTLSKHP12:mHPP12_0674  FLKVSKGHLIVAVWIKGHNGHAENERCDNLAKLEAQKRVKTT-----------T
