# Supplementary material for: Evolution in an oncogenic bacterial species with extreme genome plasticity: Helicobacter pylori East Asian genomes
Source: BMC Microbiol. 2011 May 16;11:104. doi: 10.1186/1471-2180-11-104 (PMC3120642; doi:10.1186/1471-2180-11-104)
Supplement: Additional file 6 — Multiple sequence alignments of diverged genes. [file 1471-2180-11-104-S6.ZIP › Diverged_genes_multiple_seuence_alignments/HP0728_tilS.mfa.rtf]

                  1         11        21        31        41        51        61        71        81        91                          |         |         |         |         |         |         |         |         |         |         HB8:HPB8_933      -----------LTARDFKNYLEPLREGKNLLGFSGGLDSVCLFHLLVGENIAFDIALVDYNTQKQRLEIIQHAQTLAKTHHKKCHIHYAPKIARNFEMQAHSJM:HPSJM_03675  -----------LTARDFKNYLEPLREGKNLLGFSGGLDSTCLFHLLVGENIAFDIALVDYNTQKQRLEIIQHAQTLAKTHHKKCYIHYAPKIARNFEMQAH266:HP0728       -------------VQDFKTHLEPLKEGKNLLGFSGGLDSTCLFFLLVGENIVFDIALVDYNTQKQRLEIIQHAQKLAKTHHKKCYIHHAPKIAHNFEMQAHG27:HPG27_683    -----------LIARDFKNYLEPLREGKNLLGFSGGLDSTCLFYLLVGENIAFDIALVDYNTQKQRLKIIQHAQKLAKTHHKKCYIHYAPKIARNFEMQAHHPA:HPAG1_0712   -----------LTARDFKTYLEPLREGKNLLGFSGGLDSTCLFHLLVGKNIAFDIALVDYNTQKQRLEIIQHAQKLAQAHHKKCYIHYAPKIERNFEMQAHF32:HPF32_0693   ------MPNADLIVRDFKTYLEPLREGKNLLGFSGGLDSTCLFHLLVEENIAFDTALVDYNTQKQRLEIIQHAQKLAQAHHKKCYIHYAPKIERNFEMQAHF57:HPF57_0748   ------MLNADLIARDFKTYLEPLREGKNLLGFSGGLDSTCLFHLLVEENIAFDIALVDYNTQKQRLEIIQHAQKLAQAHHKKCYIHYAPKIERNFEMQAH51:mKHP_0599     ------MLNADLIARDFKTYLEPLREKKNLLGFSGGLDSICLFHLLVEENIAFDIALVDYNTQKQRLEIIQHAQKLAQAHHKKCYIHYAPKIERNFEMQAHF30:HPF30_0609   ------MLNADLIARDFKTYLEPLREGKNLLGFSGGLDSTCLFHLLVEESIAFDTALVDYNTQKQRLEIIQHAQKLAQAHHKKCYIHYAPKIERNFEMQAHF16:HPF16_0635   ------MLNADLIARDFKTYLEPLREGKNLLGFSGGLDSTCLFHLLVGENIVFDTALVDYNTQKQRLEIIQHAQKLAQVHHKKCYIHYAPKIECNFEMQAH52:mHPKB_0622    ------MLNADLIARDFKTYLEPLREKKNLLGFSGGLDSTCLFHLLVEENIAFDTALVDYNTQKQRLEIIQHAQKLAQVHHKKCYIYYAPKIECNFEMQAHB38:mHELPY_0639  VQPIQHAFSTDLTARDFKTYLEPLREGKNLLGFSGGLDSTCLFHLLVGENIAFDIALVDYNTQKQRLEIIQHAQTLAKTHHKKCYIHHAPKIARNFEMQAHP12:HPP12_0736   -----------LIARDFKPYLEPLREGKNLLGFSGGLDSTCLFHLLVGENIAFDIALVDYNTQKQRLEIIQHAQTLAKTHHKKCYIHYAPKIARNFEMQA                  101       111       121       131       141       151       161       171       181       191                         |         |         |         |         |         |         |         |         |         |         HB8:HPB8_933      RKIRYDFFEALTKEHSYKHLILAHHLNDRLEWFLMQLSKGAGLNTLLGFQAYEKRESYAIVRPLLYTPKDTLKTLAKDHKFFEDDSNSSLKFKRNFFRKNHSJM:HPSJM_03675  RKVRYDFFETLIKEHSYKHLILAHHLNDRLEWFLMQLSKGSGLNTLLSFQAYEKRESYAIVRPLLYTPKDTLKTLAKDLKFFEDDSNSSLKFKRNCFRKHH266:HP0728       RKIRYDFFETLIKEHSYKHLILAHHLNDRLEWFLMQLSKGAGLNTLLSFQAYEKRESYAIVRPLLYTPKDTLKTLAKDLKFFEDDSNSSLKFKRNCFRKNHG27:HPG27_683    RKIRYDFFETLTKEHSYKHLILAHHLNDRLEWFLMQLSKGAGLNTLLSFQAYEKRESYAIVRPLLYTPKDTLKTLAKDQKFFEDNSNSSLKFKRNFFRKNHHPA:HPAG1_0712   RKIRYDFFETLMREYSYKHLILAHHLNDRLEWFLMQLSKGAGLNTLLSFQAYEKRGFYAIVRPLLYTPKDTLKTLAKDQKFFEDDSNSSLKFKRNFFRKHHF32:HPF32_0693   RKIRYDFFETLIKEHSYKHLILAHHLNDRLEWFLMQLSKGAGLNTLLSFQAYEKRGFYAIIRPLLYTPKDTLKTLVKDREFFEDDSNSSLKFKRNFFRKNHF57:HPF57_0748   RKIRYDFFETLIKEHSYKHLILAHHLNDRLEWFLMQLSKGAGLNTLLSFQAYEKRGFYAIVRPLLYTPKDTLKTLAKDWEFFEDDSNSSLKFKRNFFRKNH51:mKHP_0599     RKIRYDFFETLIKEHSYKHLILAHHLNDRLEWFLMQLSKGAGLNTLLSFQAYEKRGFYAIVRPLLYTPKDTLKTLAKDWEFFEDDSNSSLKFKRNFFRKNHF30:HPF30_0609   RKIRYDFFETLIKEHSYKHLILAHHLNDRLEWFLMQLGKGAGLNTLLSFQAYEKRGFYAIVRPLLYTPKDTLKTLAKDWEFFEDDSNSSLKFKRNFFRKNHF16:HPF16_0635   RKIRYDFFETLIKEHSYKHLILAHHLNDRLEWFLMQLGKGAGLNTLLSFQAYEKRGFYAIVRPLLYTPKDTLKALVKDLKFFEDDSNSSLKFKRNFFRKNH52:mHPKB_0622    RKIRYDFFETLIKEHSYKHLILAHHLNDRLEWFLMQLSKGAGLNTLLSFQAYEKRGFYAIVRPLLYTPKETLKTLAKDRKFFEDDSNSSLKFKRNFFRKNHB38:mHELPY_0639  RQVRYDFFETLIKEHSYKHLILAHHLNDRLEWFLMQLSKGAGLNTLLSFQAYEKRGFYAIVRPLLYTPKDTLKTLAKDLKFFEDDSNSSLKFKRNFFRKHHP12:HPP12_0736   RKIRYDFFETLIKEHPYKHLILAHHLNDRLEWFLMQLSKGAGLNTLLSFQAYEKRGFYAIVRPLLYTPKDTLKTLAKNLKFFEDDSNSSLKFKRNCFRKH                  201       211       221       231       241       251       261       271       281       291                         |         |         |         |         |         |         |         |         |         |         HB8:HPB8_933      YANALMQDYSKGIIQSFKFLDQEKERLYPLMPVSQRHGITFFQYSQNALFMVDKILKQKGYVLSFSQKEEIKRHFFSLEIAQKFIIESDKEHVFIALKPPHSJM:HPSJM_03675  YANALMQDYSKGIIQSFKFLDQEKEWLYPLMVVSQRHGITFFKYSQNALFMVDKILKQKGYVLSFSQKEEIKRSFFSLEIAQKFIIESDKEHVFIALKPPH266:HP0728       YANSLMQDYSKGIIQSFKFLDQEKERLYPLTIVSQMHGITFFKYSQNALFMVDKILKQKGYVLSFSQKEEIKRSFFSLEIAQKFIIESDKEHVFIALKPPHG27:HPG27_683    YANALMQDYSKGIIQSFKFLDQEKERLYPLTIVSQMHGITFFKHSQNALFMVDKILKQKGYVLSFSQKEEIKRHFFSLEIAQKFIIESDKEHAFIALKPPHHPA:HPAG1_0712   YANALMQDYSKGIIQSFKFLDQEKERLYPLTPVSQMHGITFFKHSQNALFMVDKILKQKGYVLSFSQKEEIKRHFFSLEIAQKFIIESNKEHVFIALKPPHF32:HPF32_0693   YANSLMQHYSKGIIQSFKFLDKEKEQLYSLMPVSQMHGITFFKYSQNALFMVDKILKQKGYVLSFSQKEEIKRHFFSLEIAQKFIIEKDEEHAFIALKPQHF57:HPF57_0748   YANSLMQHYSKGIIQSFKFLDKEKERLYPLMPVSQMHGITFFKYSQNALFMVDKILKQKGYVLSFSQKEEIKRHFFSLEIAQKFIIEKDKEHAFIALKPQH51:mKHP_0599     YANSLMQHYSKGIIQSFKFLDKEKERLYSLMPVSQMHGITFFKYSQNALFMVDKILKQKGYVLSFSQKEEIKRHFFSLEIAQKFIIEKDKEHAFIALKPQHF30:HPF30_0609   YANSLMQHYSKGIIQSFKFLDKEKERLYPLILVSQMHGITFFKYSQNALFMVDKILKQKGYVLSFSQKEEIKRHFFSLEIAQKFIIEKDEEHAFIALKPQHF16:HPF16_0635   YANSLMQHYSKGIIQSFKFLDKEKERLYPLMPVSQMHGITFFKYSQNALFMVDKILKQKGYVLSFSQKEEIKRHFFSLEIAQKFIIEKDREHAFIALKPQH52:mHPKB_0622    YANSLMQHYSKGIIQSFKFLDKEKERLYPLIPVSQMHGITFFKYSQNALFMVDKILKQKGYVLSFSQKEEIKRHFFSLEIAQKFIIEKDREHAFIALKPQHB38:mHELPY_0639  YANALMQHYSKGIIQSFKFLDQEKERLYSLMIVSQMHGITFFKYSQNALFMVDKILKQKGYVLSFSQKEEIKRHFFSLEIAQKFIIESDKEHVFIAFKPPHP12:HPP12_0736   YANALMQHYSKGIIQSFKFLDKEKERLYPLIPVSQMHGITFFQRSQNALFMVDKILKQKGYVLSFPQKEEIKRHFFSLEIAQKFIIESDKEHVFIAFKPQ                  301       311       321       331       341                  |         |         |         |         |HB8:HPB8_933      KTLSMPKDFKDRARRLNIPKRLRPVLYAEFLKQPTHGFLTRFKQSFINLHSJM:HPSJM_03675  KTLSMPKDFKDRARRLNIPKRLRPVLYAEFLKQPTNDFLTRFKQSLINLH266:HP0728       KTLSMPKDFKDRARRLDIPKRLRPVLYAEFLKQPTHDFLTRFKQSLMDLHG27:HPG27_683    KTLSMPKDFKDRARRLDIPKRLRPVLYAEFLKQPTHDFLTRFKQGLMDLHHPA:HPAG1_0712   KTLSMPKDFKEKARRLDIPKRLRPVLYAEFLKQPTHDFLTRFKQSLTDLHF32:HPF32_0693   KTLSMPKDFKEKARRLNIPKRLRPVLYAEFLKQPTHDFLTRFKQSLTDLHF57:HPF57_0748   KTLSMPKDFKEKARRLNIPKRLRPVLYAEFLKQPTHDFLTRFKQSLTDLH51:mKHP_0599     KTLSMPKDFKDKARRLNIPKRLRPVLYAEFLKQPTHDFLTRFKQGLTDLHF30:HPF30_0609   KTLSMPKDFKDKARRLNIPKRLRPVLYAEFLKQPTHDFLTRFKQSLTDLHF16:HPF16_0635   KTLSMPKDFKDKARRLNIPKRLRPVLYAEFLKQPTHDFLTRFKQGLTDLH52:mHPKB_0622    KILSMPKDFKDKARRLNIPKRLRPVLYAEFLKQPTHDFLTRFKQSLTDLHB38:mHELPY_0639  KTLSMPKDFKDKARRLDIPKRLRPVLYAEFLKQPTHDFLTHFKQSLINLHP12:HPP12_0736   KTLSMPKDFKDRARRLDIPKRLRPVLYAEFLKQPTHDFLTRFKQSLINL
