# Supplementary material for: Evolution in an oncogenic bacterial species with extreme genome plasticity: Helicobacter pylori East Asian genomes
Source: BMC Microbiol. 2011 May 16;11:104. doi: 10.1186/1471-2180-11-104 (PMC3120642; doi:10.1186/1471-2180-11-104)
Supplement: Additional file 6 — Multiple sequence alignments of diverged genes. [file 1471-2180-11-104-S6.ZIP › Diverged_genes_multiple_seuence_alignments/HP0754.mfa.rtf]

                  1         11        21        31        41        51        61        71                  |         |         |         |         |         |         |         |HB8:HPB8_961      MNSPNALLDSFKIALVKKDSKQAFSLIERLSLEQIKNLDLNTLLSLKEMIAQSIELLEKEKEELQSQMHKAKKIQKFLSHF32:HPF32_0722   MNSPNMLLDSFKIALIKKDSKQAFSLIERLSLEQIKSLDLDTLLSLKEMIAQSIELLEKEKEELQSQMHKAKKIQKFLSHF57:HPF57_0775   MNSPDMLLDSFKIALIKKDSKQAFSLIERLSLEQIKSLDLDTLLSLKEMIAQSIELLEKEKEELQSQMHKAKKIQKFLSHF16:HPF16_0607   MNSPDMLLDSFKIALIKKDSKQAFSLIERLSLEQIKSLDLDTLLSLKEMIAQSIELLEKEKEELQSQMHKAKKIQKFLSH51:KHP_0572      MNSPDMLLDSFKVALIKKDSKQAFSLIERLSLEQIKSLDLDTLLSLKEMIAQSIELLEKEKEELQSQMHKAKKIQKFLSH52:HPKB_0593     MNSLNMLLDSFKIALIKKDSKQAFSLIERLSLEQIKSLDLDTLLSLKEMIAQSIELLEKEKEELQSQMHKAKKIQKFLSHHPA:HPAG1_0739   MNSPNILLDSFKIALVKKDSKQAFSLIERLSLEQIKNLDLDTLLSLKEMIAQSIELLEKEKEELQSQMHKAKKIQKFLSHF30:HPF30_0579   MNSPNMLLDSFKIALIKKDSKQAFSLIECLSLEQIKSLDLDTLLSLKEMIAQSIELLEKEKEELQSQMHKAKKIQKFLSHSJM:HPSJM_03825  MNSPNALLDSFKIALVKKDSKQAFSLIERLSLEQIKSLDLDALLSLKEMIAQSIELLEKEKEELQSQMHKAKKIQKFLSH266:HP0754       MNSPNILLDSFKIALVKKDSKQAFSLIERLSLEQIKSLDLDALLSLKEMIAQSIELLEKEKEELQLQMHKAKKIQKFLSHB38:HELPY_0611   MNSPNALLDSFKIALVKKDSKQAFSLIERLSLEQIKSLDLNTLLSLKEMIAQSIELLEKEKEELQLQMHKAKKIQKFLSHG27:mHPG27_711   MNSPNALLDSFKIALVKKDSKQAFSLIERLSLEQIKSLDLDTLLSLKEMIAQSIELLEKEKEELQLQMHKAKKIQKFLSHP12:HPP12_0764   MNSPDVLLDSFKIALVKKDSKQAFSLIERLSLEQIKNLDLDTLLSLKEMIAQSIELLEKEKEELQLQMHKAKKIQKFLS
