# Supplementary material for: Evolution in an oncogenic bacterial species with extreme genome plasticity: Helicobacter pylori East Asian genomes
Source: BMC Microbiol. 2011 May 16;11:104. doi: 10.1186/1471-2180-11-104 (PMC3120642; doi:10.1186/1471-2180-11-104)
Supplement: Additional file 6 — Multiple sequence alignments of diverged genes. [file 1471-2180-11-104-S6.ZIP › Diverged_genes_multiple_seuence_alignments/HP0793_def.mfa.rtf]

                  1         11        21        31        41        51        61        71        81        91                          |         |         |         |         |         |         |         |         |         |         HB8:HPB8_1001     MALLEIIHYPSKILRTISKEVVSFDAKLHQQLDDMHETMIASEGIGLAAIQVGLPLRMLIINLPREDGVQHKEDCLEIINPKWIETGGTIMYKEGCLSVPHSJM:HPSJM_04015  MALLEIIHYPSKILRTISKEVVSFDAKLHQQLDDMHETMIASEGIGLAAIQVGLPLRMLIINLPQEDGVQHKEDCLEIINPKFIEAGGSMMYREGCLSVPHB38:HELPY_0567   MALLEIIHYPSKILRTISKEVVSFDSKLHQQLDDMHETMIASEGIGLAAIQVGLPLRMLIINLPREDGVQHKEDCLEIINPKFIETGGSMMYREGCLSVPHG27:HPG27_749    MALLEIIHYPSKILRTISKEVVSFDAKLHQQLDDMHETMIASEGIGLAAIQVGLPLRMLIINLPREDGVQHKEDCLEIINPKFIETGGSMMYREGCLSVPHHPA:HPAG1_0778   MALLEIIHYPSKILRTISKEVVSFDAKLHQQLDDMHETMIASEGIGLAAIQVGLPLRMLIINLPREDGVQHKEDCLEIINPKFIETGGSMMYREGCLSVPH266:HP0793       MALLEIIHYPSKILRTISKEVVSFDSKLHQQLDDMHETMIASEGIGLAAIQVGLPLRMLIINLPQEDGVQHKEDCLEIINPKFIETGGSMMYREGCLSVPHP12:HPP12_0800   MALLEIIHYPSKILRTISKEVVSFDSKLHQQLDDMHETMIASEGIGLAAIQVGLPLRMLIINLPQEDGVQHKEDCLEIINPKWIETKGSMMYKEGCLSVPHF32:HPF32_0760   MALLEIIHYPSKILRTISKEVVSFDAKLHQQLDDMHETMIASEGIGLAAIQVGLPLRMLIINLPREDGVQHKEDCLEIINPKFIETKGTIMYKEGCLSVPHF16:HPF16_0569   MALLEIIHYPSKILRTISKEVVSFDAKLHQQLDDMHETMIASEGIGLAAIQVGLPLRMLIINLPREDGVQHKEDCLEIINPKFIETKGTIMYKEGCLSVPH51:KHP_0535      MALLEIIHYPSKILRTISKEVVSFDAKLHQQLDDMHETMIASEGIGLAAIQVGLPLRMLIINLPREDGVQHKEDCLEIINPKFIETKGTIMYKEGCLSVPHF57:HPF57_0813   MALLEIIHYPSKILRTISKEVVSFDSKLHQQLDDMHETMIASEGIGLAAIQVGLPLRMLIINLPREDGVQHKEDCLEIINPKFIETKGTIMYKEGCLSVPH52:HPKB_0557     MALLEIIHYPSKILRTISKEIVSFDSKLHQQLDDMHETMIASEGIGLAAIQVGLPLRMLIINLPREDGVQHKEDCLEIINPKFIETKGTIMYKEGCLSVPHF30:HPF30_0538   MALLEIIHYPSKILRTISKEVVSFDSKLHQQLDDMRETMIASEGIGLAAIQVGLPLRMLIINLPREDGVQHKEDCLEIINPKFIETKGTIMYKEGCLSVP                  101       111       121       131       141       151       161       171                  |         |         |         |         |         |         |         |HB8:HPB8_1001     GFYEEVERFEKVKIEYQNRFAEVKVLEASELLAVAIQHEIDHLNGVLFVDKLSILKRKKFEKELKELQKKQKHK-HSJM:HPSJM_04015  GFYEEVERFEKVKIEYQNRFAEVKVLEASELLAVAIQHEIDHLNGVLFVDKLSILKRKKFEKELKELQKKQKHK-HB38:HELPY_0567   GFYEEVERFEKVKIEYQNRFAEVKVLEASELLAVAIQHEIDHLNGVLFVDKLSILKRKKFEKELKELQKKQKHK-HG27:HPG27_749    GFYEEVERFEKVKIEYQNRFAEVKVLEASELLAVAIQHEIDHLNGVLFVDKLSILKRKKFEKELKELQKKQKHK-HHPA:HPAG1_0778   GFYEEVERFEKVKIEYQNRFAEVKVLEASELLAVAIQHEIDHLNGVLFVDKLSILKRKKFEKELKELQKQQKHK-H266:HP0793       GFYEEVERFEKVKIEYQNRFAEVKVLEASELLAVAIQHEIDHLNGVLFVDKLSILKRKKFEKELKELQKKQKHE-HP12:HPP12_0800   GFYEEVERFEKVKIEYQNRFAEVKILEASELLAVAIQHEIDHLNGVLFVDKLSILKRKKFEKELKELQKKQKRE-HF32:HPF32_0760   GFYEEVERFEKVKIEYQNRFAEVKILEASELLAVAIQHEIDHLNGVLFVDKLSILKRKKFEKELKELNKNPKNKSHF16:HPF16_0569   GFYEEVERFEKVKIEYQNRFAEVKILEASELLAVAIQHEIDHLNGVLFVDKLSILKRKKFEKELKELNKNPKNKSH51:KHP_0535      GFYEEVERFEKVKIEYQNRFAEVKILEASELLAVAIQHEIDHLNGVLFVDKLSILKRKKFEKELKELNKNPKNKSHF57:HPF57_0813   GFYEEVERFEKVKIEYQNRFAEVKILEASELLAVAIQHEIDHLNGVLFVDKLSILKRKKFEKELKELNKNPKNKSH52:HPKB_0557     GFYEEVERFEKVKIEYQNRFAEVKILEASELLAVAIQHEIDHLNGVLFVDKLSILKRKKFEKELKELNKNPKNKSHF30:HPF30_0538   GFYEEVERFEKVKIEYQNRFAEVKILEASELLAVAIQHEIDHLNGVLFVDKLSILKRKKFEKELKELNKNPRNKS
