# Supplementary material for: Evolution in an oncogenic bacterial species with extreme genome plasticity: Helicobacter pylori East Asian genomes
Source: BMC Microbiol. 2011 May 16;11:104. doi: 10.1186/1471-2180-11-104 (PMC3120642; doi:10.1186/1471-2180-11-104)
Supplement: Additional file 6 — Multiple sequence alignments of diverged genes. [file 1471-2180-11-104-S6.ZIP › Diverged_genes_multiple_seuence_alignments/HP0806.mfa.rtf]

                  1         11        21        31        41        51        61        71        81        91                          |         |         |         |         |         |         |         |         |         |         HB8:HPB8_1014     MLDNIPITIQKSKKIKTLSLNITPSLEVILKMPDSCPQARANAFLKEQETWLKKTFLAMQEKHSLLRVRLNTYKDKILVFDEVKNANDYTLTDLKKILKTHF32:HPF32_0773   MLDDIPITIQKSKKIKTLSLNITPSLEVILKMPDSCSQNRAHAFLKEQKAWLQKTLSAMQEKYSLLHS--QTYQNKILVFDEEKNANDYTLTELKKILKTHSJM:HPSJM_04085  MLDNVPITIQKSKKIKTLSLNITPSLEVILKMPYSCSQTRASAFLKEQEAWLKKTFLAMQEKYSLLRSRLETYQNKILVFDEVKNANDYTLTDLKKILKTHv22:HPV225_0820  MLDDIPITIQKSKKIKTLSLNITPSLEVILKMPDSCSQNRAHAFLKEQKAWLQKTLSAMQEKYSLLHSCLETYQNKILVFDEEKNANDYTLTELKKILKTHPeC:HPPC_04065   MLDDIPITIQKSKKIKTLSLNITPSLEVILKMPNSCSQNRAHAFLKEQKAWLQKTLSAMQEKYSLLHSRLETYQNKILVFDEEKNANDYTLTELKKILKTH266:HP0806       MLDDIPITIQKSKKIKTLSLNITPSLEVILKMPNSCSQTRASAFLKEQEAWLKKTFLSMQEKHSLLRTNLEKYQNKILVFDEVKNANDYTLTELKKILKTHF57:HPF57_0828   MLDDIPITIQKSEKIKTLSLNITPSLEVILKMPNSCSQARVHAFLKEQEAWLQKTLSAMQEKYSLLHS--QTYQNKILVFDEEKNANDYTLTELKKILKTHF16:HPF16_0556   MLDDIPITIQKSKKIKTLSLNITPSLEVILKMPDSCSQNRVHAFLKEQKAWLQKTLSAMQEKYSLLHS--QTYQNKILVFDEEKNANDYTLTELKKILKTHB38:HELPY_0553   MLDDIPITIQKSEKIKTLSLNITPSLEVILKMPNSCSQARASAFLKEQESWLKKTFLSMQEKHSLLRANLETYQNKILVFDEVKNANDYTLTDLKKILKTHCuz:HPCU_04270   MLDDIPITIQKSKRIKTLSLSITPSLEVILKMPNSCSQNRVHAFLKEQKAWLQKTLSAMQKKYSLLHSRLETYQNKILVFDEEKNANDYTLTELKKILKTHShi:HPSH_02785   MLDDIPITIQKSKKIKTLSLNITPSLEVILKMPDSCSQNRAHAFLKEQKAWLQKTLSAMQKKYSLLHSRLETYQNKILVFDEEKNANDYTLTELKKILKTH908:hp908_0820   MLDNVPITIQKSKKIKTLSLNVTPSLEVILKMPDSCPQARASAFLKEQEAWLKKTLLAMQEKHSLLHSRLETYQNKILVFDEVKNANDYTLTELKKILKTHHPA:HPAG1_0791   MLDDIPITIQKSEKIKTLSLNITPSLEVILKMPNSCSQARASAFLKEQEAWLKKTLSAMQEKYSLLRANLETYKNKILVFDEVRNANDYTLTELKKILKTHP12:HPP12_0812   MLDDIPITIQKSEKIKTLSLNITPSLEVILKMPNSCSQTRASAFLKEQEAWLKKTFLSMQEKHSLLHANLEKYKNKILVFDEIKNANDYTLTDLKKILKTH51:mKHP_0522     MLDDIPITIQKSKKIKNLSLNITPSLEVILKMPNSCSQNRAHAFLKEQKAWLQKTLSAMQKKYSLLHS--QTYQNKILVFDEEKNANDYTLTELKKILKTHSat:HPSAT_02745  MLDDIPITIQKSKKIKTLSLNITPSLEVILKMPDSCSQNRAHAFLKEQKAWLQKTLSAMQEKYSLLHSRLETYQNKILVFDEEKNANDYTLTELKKILKTHJ99:jhp0742      MLDNIPITIQKSKKIKTLSLNVTPSLEVILKMPDSCPQARANAFLKEQEAWLKKTLLAMQEKHSLLRTNLEKYKNKILVFDEMRNANDYTLTDLKKILKTH52:HPKB_0544     MLDDIPITIQKSKKIKTLSLNITPSLEVILKMPDSCSQNRAHAFLKEQKAWLKKTLSAMQEKYSLLHS--QTYQNKILVFDEVKNANDYTLTELKKILKTHG27:HPG27_762    MLDDIPITIQKSKKIKTLSLNITPSLEVILKMPDSCPQARASAFLKEQEAWLKKTLLAMQEKYSLLRSCLVTYQNKILVFDEVKNANDYTLTELKKILKTHF30:HPF30_0525   MLDDIPITIQKSKKIKTLSLNITPSLEVILKMPDSCSQTRVHAFLKEQKAWLQKTLSAMQKKYSLLHS--QTYQNKILVFDEEKNANDYTLTELKKILKT                  101       111       121       131       141       151       161       171       181       191                         |         |         |         |         |         |         |         |         |         |         HB8:HPB8_1014     YLEQKLPLISQKMQTSYTHFSIRNNAKVLGSCSYHNRLSFALLLVCAQKEAIDYVIIHELAHTIHKNHSKNFWRCVQIFCPNYRALRERLKQNIIFYAQLHF32:HPF32_0773   YLEQQLPLSAQKMQTSYTHFSVRNNAKVLGSCSYHNRLSFALLLVCAKKEAIDYVIIHELAHTIHKNHSKNFWRCVEIFCPNYRTLREHLKQRVVFYTQLHSJM:HPSJM_04085  YLEQQLPLIAQKMQTSYTHFSIRNNAKVLGSCSYHNRLSFALLLVCAQKEAIDYVIIHELAHTIHKNHSKNFWRCVQTFCPNYRALREHLKQRVVFYTQLHv22:HPV225_0820  YLEQQLSLSAQKMQTSYTHFSVRNNAKVLGSCSYHNRLSFALLLVCAKKEAIDYVIIHELAHTIHKNHSKNFWRCVEIFCPNYRTLREHLKQRVVFYTQLHPeC:HPPC_04065   YLEQKLPLSAQKMQTSYTHFSVRNNAKVLGSCSYHNRLSFALLLVCAKKEAIDYVIIHELAHTIHKNHSKNFWRCVEIFCPNYRALREHLKQRVVFYTQLH266:HP0806       YLEQKLPLIAQKMQTSYTHFSIRNNAKVLGSCSYHNRLSFALLLVCAQKEAIDYVIIHELAHTIHKNHSKNFWRCVQIFCPNYRALRERLKQNTIFYAQLHF57:HPF57_0828   YLEQKLPLSAQKMQTSYTHFSIRNNAKVLGSCSYHNRLSFALLLVCAKKEAIDYVIIHELAHTIHKNHSKNFWRCVEIFCPNYRALREHLKQRVVFYTQLHF16:HPF16_0556   YLEQKLPLSAQKMQTSYTHFSVRNNAKVLGSCSYHNRLSFALLLVCAKKEAIDYVIIHELAHTIHKNHSKNFWRCVEIFCPNYRTLREHLKQRVVFYTQLHB38:HELPY_0553   YLEQQLPLIAQKMQTSYTHFSIRNNAKVLGSCSYHNRLSFALLLVCAQKEAIDYVIIHELAHTIHKNHSKNFWRCVKIFCPNYRALRERLKQRVVFYTQLHCuz:HPCU_04270   YLEQKLPLSAQKMQTSYTHFSVRNNAKVLGSCSYHNRLSFALLLVCAKKEAIDYVIIHELAHTIHKNHSKNFWRCVEIFCPNYHALREHLKQRVVFYTQLHShi:HPSH_02785   YLEQQLPLSAQKMQTSYTHFSVRNNAKVLGSCSYHNRLSFALLLVCAKKEAIDYVIIHELAHTIHKNHSKNFWRCVEIFCPNYRALREHLKQRVVFYTQLH908:hp908_0820   YLERKLPSIAQKMQTSYTGFSVRNNAKVLGSCSYHNRLSFALLLVCAQKEAIDYVIIHELAHTIHKNHSKNFWRCVKTFCPNYRALREHLKQMVVFYTQLHHPA:HPAG1_0791   YLEQKLPLIAQKMQTSYTHFSVRNNAKVLGSCSYHNRLSFALLLVCAQKEAIDYVIIHELAHTIHKNHSKNFWRCVQIFCPNYRTLRERLKQNIIFYAQLHP12:HPP12_0812   YLERKLPLIAQKMQTSYTHFSIRNNAKVLGSCSYHNRLSFALLLVCAQKEAIDYVIIHELAHTIHKNHSKNFWRCVQIFCPNYRALREHLKQRVVFYTQLH51:mKHP_0522     YLEQQLPLSAQKMQTSYTHFSVRNNAKVLGSCSYHNRLSFALLLVCAKKEAIDYVIIHELAHTIHKNHSKNFWRCVEIFCPNYRALREHLKQRVVFYTQLHSat:HPSAT_02745  YLEQELPLSAQKMQTSYTHFSVRNNAKVLGSCSYHNRLSFALLLVCAKKEAIDYVIIHELAHTIHKNHSKNFWRCVEIFCPNYRALREHLKQRVVFYTQLHJ99:jhp0742      YLEQKLPLIAQKMQTSYTHFSIRNNAKVLGSCSYHNRLSFALLLVCAKKEAIDYVIIHELAHTIHKNHSKNFWRCVQIFCPNYRALRERLKQRVVFYTQLH52:HPKB_0544     YLEQKLPLIAQKMQTSYTGFNIRNNAKVLGSCSYHNRLSFALLLVCAQKEAIDYVIIHELAHTIHKNHSKNFWRCVEIFCPNYRTLRERLKQRVVFYTQLHG27:HPG27_762    YLEQKLPLIAQKMQTSYTGFNIRNNAKVLGSCSYHNRLSFALLLVCTQKEAIDYVIIHELAHTIHKNHSKNFWRCVQIFCPNYRALREHLKQRVVFYTQLHF30:HPF30_0525   YLEQKLPLSAQKMQTSYTHFSVRNNAKVLGSCSYHNRLSFALLLVCAKKEAIDYVIIHELAHTIHKNHSKNFWRCVEIFCPNYRALREHLKQRVVFYTQL                  201                  |HB8:HPB8_1014     LKTLQPHF32:HPF32_0773   LKTLQPHSJM:HPSJM_04085  LKQLQPHv22:HPV225_0820  LKPLQPHPeC:HPPC_04065   LKPLQPH266:HP0806       LKTLQPHF57:HPF57_0828   LKPLQPHF16:HPF16_0556   LKPLQPHB38:HELPY_0553   LKQLQPHCuz:HPCU_04270   LKQLQPHShi:HPSH_02785   LKPLQPH908:hp908_0820   LKPLQPHHPA:HPAG1_0791   LKTLQPHP12:HPP12_0812   LKQLEPH51:mKHP_0522     LKQLQPHSat:HPSAT_02745  LKPLQPHJ99:jhp0742      LKPLQPH52:HPKB_0544     LKPLQPHG27:HPG27_762    LKQLEPHF30:HPF30_0525   LKQLQP
