# Supplementary material for: Evolution in an oncogenic bacterial species with extreme genome plasticity: Helicobacter pylori East Asian genomes
Source: BMC Microbiol. 2011 May 16;11:104. doi: 10.1186/1471-2180-11-104 (PMC3120642; doi:10.1186/1471-2180-11-104)
Supplement: Additional file 6 — Multiple sequence alignments of diverged genes. [file 1471-2180-11-104-S6.ZIP › Diverged_genes_multiple_seuence_alignments/HP0861.mfa.rtf]

                  1         11        21        31        41        51        61        71        81        91                          |         |         |         |         |         |         |         |         |         |         HB8:HPB8_1070     MQMMQNLSFLGMFLAALSMSLGHCVGMCGGIVSAFSQIRFSKVTSFSYQLTCHALYNVGRISTYMLLGAITAGLGHSLSVSMGFRGVLLMSMGVVLILLAHF32:HPF32_0827   --MMQNLSFLGMFLAALSMSLGHCVGMCGGIVSAFSQIRFSKVTSFSYQLACHALYNVGRISSYMLLGAIAAGLGHSLSVSMGFRGVLFISMGIILIGLAHSJM:HPSJM_04370  MQMMQNLSFLGMFLAALSMSLGHCVGMCGGIVSAFSQIRFSKVTSFSYQLTCHVLYNVGRISTYMLLGAITAGLGHSLSVSMGFRGVLLMSMGIILICLAHv22:HPV225_0882  MQMMHNLSFLGMFLAALSMSLGHCVGMCGGIVSAFSQIRFSKVTSFSYQLACHALYNVGRISTYMLLGAIAAGLGHSLSVSMGFRGVLFISMGIILIGLAHPeC:HPPC_04350   MQMMHNLSFLGMFLAALSMSLGHCVGMCGGIVSAFSQIRFSKVTSFSYQLACHALYNVGRISTYMLLGAIAAGLGHSLSVSMGFRGVLFISMGIILICLAH266:HP0861       MQMMHNLSFLGMFLAALSMSLGHCVGMCGGIVSAFSQIRFSKVTSFSYQLTCHALYNVGRISTYMLLGAIAASLGHSLSVSMGFRGVLFISMGIILICLAHF57:HPF57_0879   MQMMHNLSFLGMFLAALSMSLGHCVGMCGGIVSAFSQIRFSKVTNFSYQLACHALYNLGRISTYMLLGAIAAGLGHSLSVSMGFRGVLFISMGIILIGLAHF16:HPF16_0503   MQMMHNLSFLGMFLAALSMSLGHCVGMCGGIVSAFSQIKFSKVTSFSYQLACHALYNVGRISTYMLLGAIAASLGHSLSVSMGFRGVLFISMGIVLILLAHB38:mHELPY_0491  MQMMQNLSFLGMFLAALSMSLGHCVGMCGGIVSAFSQIRFSKVTSFSYQIACHALYNVGRISTYMLLGAITAGLGHSLSVSMGFRGVLLMSMGVVLILLAHCuz:HPCU_04575   MQMMHNLSFLGMFLAALSMSLGHCVGMCGGIVSAFSQIRFSKVTSFSYQLACHALYNVGRISTYMLLGAIAAGLGHSLSVSMGFRGVLFISMGIILIGLAHShi:HPSH_02505   MQMMHNLSFLGMFLAALSMSLGHCVGMCGGIVSAFSQIRFSKVTSFSYQLACHALYNVGRISTYMLLGAIAAGLGHSLSVSMGFRGVLFISMGIVLICLAH908:hp908_0872   MQMMQNLSFLGMFLAALSMSLGHCVGMCGGIVSAFSQIKFSKVTNFSYQLTCHALYNVGRISTYMLLGAITAGLGHSLSVSMGFRGVLLMSMGVVLILLAHHPA:HPAG1_0844   MQMMHHLSFLGMFLAALSMSLGHCVGMCGGIVSAFSQIRFSKVTSFSYQLTCHALYNVGRISTYMLLGAITAGLGHSLSVSMGFRGVLLMSMGIILIGLAHP12:HPP12_0861   MQMMQNLSFLGMFLAALSMSLGHCVGMCGGIVSAFSQIRFSKVTSFSYQITCHALYNIGRISTYMLLGAITAGLGHSLSVSMGFRGVLLIGMGVVLILLAH51:KHP_0471      MQMMHNLSFLGMFLAALSMSLGHCVGMCGGIVSAFSQIRFSKVTSFSYQLACHALYNLGRISTYMLLGAIAAGLGHSLSVSMGFRGVLFISMGIILIGLAHSat:HPSAT_02425  MQMMHNLSFLGMFLAALSMSLGHCVGMCGGIVSAFSQIRFSKVTSFSYQLACHALYNVGRISAYMFLGAIAAGLGHSLSVSMGFRGVLFISMGIVLICLAHJ99:jhp0795      MQMMQNLSFLGMFLAALSMSLGHCVGMCGGIVSAFSQIRFSKVTSFSYQIACHALYNVGRISTYMLLGAITAGLGNSLSVSMGFRGVLLMSMGIVLILLAH52:mHPKB_0488    MQMMHNLSFLGMFLAALSMSLGHCVGMCGGIVSAFSQIRFSKVTSFSYQLACHALYNVGRISTYMLLGAIAAGLGHSLSVSMGFRGVLFISMGIILIGLVHG27:mHPG27_815   MQMIHHLSFLGMFLAALSMSLGHCVGMCGGIVSAFSQIRFSKVTSFSYQLTCHALYNVGRISTYMLLGAITAGLGHSLSVSMGFRGVLLMSMGIILICLAHF30:HPF30_0472   MQMMHNLSFLGMFLAALSMSLGHCVGMCGGIVSAFSQIRFSKVTSFSYQLACHALYNVGRISTYMLLGAIAAGLGHSLSVSMGFRGVLFISMGIILIGLA                  101       111       121       131       141       151       161       171       181       191                         |         |         |         |         |         |         |         |         |         |         HB8:HPB8_1070     LLGSKAEKLSFQIPFISFLMKKTLQSQNILGLYFLGVLNGFLPCMMVYSFLASVILSHSAFMGAMLGLSFGLGTSVPLFLMGIFLSKISISYRKFFNLLSHF32:HPF32_0827   LLGAKVEKLSFQIPFISFLMKKTLQSQNILGLYFLGVLNGFLPCMMVYSFLASVILSHSAFMGAMLGLSFGLGTSVPLFLMGVFLSKISISYRKFFNLLSHSJM:HPSJM_04370  LLGARMEKLSFQIPFISFLMKKTLQSQNILGLYFLGVLNGFLPCMMVYSFLASVILSHSAFMGAMLGLSFGLGTSVPLFLMGIFLSKISVSYRKFFNLLSHv22:HPV225_0882  LLGAKVEKLSFQIPFISFLMKKTLQSQNILGLYFLGVLNGFLPCMMVYSFLASVILSHSAFMGAMLGLSFGLGTSVPLFLMGFFLSKISISYRKFFNLLSHPeC:HPPC_04350   LLGAKVEKLSFQIPFISFLMKKTLQSQNILGLYFLGVLNGFLPCMMVYSFLASVILSHSAFMGAMLGLSFGLGTSVPLFLMGVFLSKISISYRKFFNLLSH266:HP0861       LLGARMEKLSFQIPFISFLMKKTLQSQNILGLYFLGVLNGFLPCMMVYSFLASVILSHSAFMGAMLGLSFGLGTSMPLFLMGIFLSKISVSYRKFFNLLSHF57:HPF57_0879   LLGARMEKLSFQIPFISFLMKKTLQSQNILGLYFLGVLNGFLPCMMVYSFLASVILSHSAFMGAMLGLSFGLGTSVPLFLMGVFLSKISISYRKFFNLLSHF16:HPF16_0503   LLGARMEKLSFQIPFISFLMKKTLQSQNILGLYFLGVLNGFLPCMMVYSFLASVILSHSAFMGAMLGLSFGLGTSVPLFLMGVFLSKISVSYRKFFNLLSHB38:mHELPY_0491  LLGAKAEKLSFQIPFISFLMKKTLQSQNILGLYFLGVLNGFLPCMMVYSFLASVILSHSAFMGAMLGLSFGLGTSVPLFLMGIFLSKISISYRKFFNLLSHCuz:HPCU_04575   LLGAKVEKLSFQIPFISFLMKKTLQSQNILGLYFLGVLNGFLPCMMVYSFLASVILSHSAFMGAMLGLSFGLGTSVPLFLMGFFLSKISVSYRKFFNLLSHShi:HPSH_02505   LLGAKVEKLSFQIPFISFLMKKTLQSQNILGLYFLGVLNGFLPCMMVYSFLASVILSHSAFMGAMLGLSFGLGTSVPLFLMGVFLSKISISYRKFFNLLSH908:hp908_0872   LLGAKAEKLSFSIPFISFLMKKTLQSQNVLGLYFLGVLNGFLPCMMVYSFLASVILSHSMFMGAMLGLSFGLGTSVPLFLMGVFLSKISTSYRKFFNLLSHHPA:HPAG1_0844   LLGARMEKLSFQIPFISFLMKKTLQSQNILGLYFLGVLNGFLPCMMVYSFLASVILSHSAFMGAMLGLSFGLGTSVPLFLMGIFLSKISISYRKFFNLLSHP12:HPP12_0861   LLGSKAEKLSFQIPFISFLMKKTLQSQNILGLYFLGVLNGFLPCMMVYSFLASVILSHSAFMGAMLGLSFGLGTSVPLFLMGIFLSKISVSYRKFFNLLSH51:KHP_0471      LLGARMEKLSFQIPFISFLMKKTLQSQNILGLYFLGVLNGFLPCMMVYSFLASVILSHSAFMGAMLGLSFGLGTSVPLFLMGVFLSKISVSYRKFFNLLSHSat:HPSAT_02425  LLGAKVEKLSFQIPFISFLMKKTLQSQNILGLYFLGVLNGFLPCMMVYSFLASVILSHSAFMGAMLGLSFGLGTSVPLFLMGVFLSKISVSYRKFFNLLSHJ99:jhp0795      LLGAKAEKLSFQIPFISFLMKKTLQSQNVLGLYFLGVLNGFLPCMMVYSFLASVILSHSTFMGAMLGLSFGLGTSVPLFLMGIFLSKISISYRKFFNLLSH52:mHPKB_0488    LLGAKVEKLSFQIPFISFLMKKTLQSQNILGLYFLGVLNGFLPCMMVYSFLASVILSHSAFMGAMLGLSFGLGTSVPLFLMGVFLSKISVSYRKFFNLLSHG27:mHPG27_815   LLGARMEKLSFQIPFISFLMKKTLQSQNILGLYFLGVLNGFLPCMMVYSFLASVILSHSAFMGAMLGLSFGLGTSMPLFLMGIFLSKISISYRKFFNLLSHF30:HPF30_0472   LLGARMEKLSFQIPFISFLMKKTLQSQNILGLYFLGVLNGFLPCMMVYSFLASVILSHSAFMGAMLGLSFGLGTSVPLFLMGVFLSKISISYRKFFNLLS                  201       211       221       231       241       251                  |         |         |         |         |         |HB8:HPB8_1070     KGLMGVFGLYVLYMGIMLINHQTPHAMHHQNSTTQHDHKGA-------HSHEHHF32:HPF32_0827   KGLMGVFGLYVLYMGIMLINHQNPHAMHHSNETTKHDHKRM-------HERH-HSJM:HPSJM_04370  KGLMGVFGLYVLYMGIMLISHQTPHAMHHQSDMMHHQNNATPQQESHFHDHH-Hv22:HPV225_0882  KGLMGVFGLYILYMGIMLINHQNPHAMHHSSETTQHDHKGM-------HERH-HPeC:HPPC_04350   KGLMGVFGLYILYMGIMLINHQNPHAMHYQSDMMHHQNNATPQQESH-HDH--H266:HP0861       KILMGVFGLYILYMGIMLINHKMPHAMHHQNNTTQHDHKGV-------HSHEHHF57:HPF57_0879   KGLMGVFGLYILYMGIMLINHQNPHAMHHSSETTQHDHKRM-------HERH-HF16:HPF16_0503   KGLMGVFGLYILYMGIMLINHQNPHAMHHSSETTQHDHKGM-------HERH-HB38:mHELPY_0491  KGLMGVFGLYVLYMGIMLINHQTPHAMHHQNNTTQHDHKGA-------HSHEHHCuz:HPCU_04575   KGLMGVFGLYILYMGIMLINHQNPHAMHHSSETTQHDHKGM-------HERH-HShi:HPSH_02505   KGLMGVFGLYILYMGIMLINHQNPHAMHHSSETTQHDHKGM-------HERH-H908:hp908_0872   KGLMGVFGLYVLYMGIMLINHKMPHAMHHQSDMMCHQNNATPQQESHFHDHH-HHPA:HPAG1_0844   KGLMGVFGLYVFYMGIMLINHKMPHAMHHQNSTTQHDHKGV-------HSHEHHP12:HPP12_0861   KILMGVFGLYVLYMGIMLINHKMPHAMHHQNSTTQHDYKGM-------HSHEHH51:KHP_0471      KGLMGVFGLYILYMGIMLINHQNPHAMHHSNETTQHDHKRM-------HERH-HSat:HPSAT_02425  KGLMGVFGLYILYMGIMLINHQNPHAMHHSSETTQHDHKGM-------HERH-HJ99:jhp0795      KGLMGVFGLYVLYMGIMLISHQTPHAMRHQ-------NNATPQQEGHFHDHH-H52:mHPKB_0488    KGLMGVFGLYILYMGIMLINHQNPHAMHHSSETTQHDHKRM-------HEHH-HG27:mHPG27_815   KILMGVFGLYVLYMGIMLINHKMPHAMHHQNNTTQHDHKGA-------HSHEHHF30:HPF30_0472   KGLMGVFGLYILYMGIMLINHQNPHAMHHLGETTQHDHKRM-------HERH-
