# Supplementary material for: Evolution in an oncogenic bacterial species with extreme genome plasticity: Helicobacter pylori East Asian genomes
Source: BMC Microbiol. 2011 May 16;11:104. doi: 10.1186/1471-2180-11-104 (PMC3120642; doi:10.1186/1471-2180-11-104)
Supplement: Additional file 6 — Multiple sequence alignments of diverged genes. [file 1471-2180-11-104-S6.ZIP › Diverged_genes_multiple_seuence_alignments/HP0887_vacA.mfa.rtf]

                  1         11        21        31        41        51        61        71        81        91                          |         |         |         |         |         |         |         |         |         |         HB8:HPB8_666      MEIQQTHRKINRPLVSLALVGALVSI-----TP----QQSHAAFFTTVIIPAIVGGIATGTAVGTVSGLLSWGLKQAEEANKTPDKPDKVWRIQAGRGFNHB38:HELPY_0872   MEIQQTHRKINRPIISLALVGVLMGTELGANTPNDPIHSESRAFFTTVIIPAIVGGIATGAAVGTVSGLLSWGLKQAEQANKAPDKPDKVWRIQAGRGFDHF32:HPF32_0462   MELQQTHRKINRPLVSLALVGLLVSI-----TP----QKSHAAFFTTVIIPAIVGGIATGAAVGTVSGLLGWGLKQAEEANKTPDKPDKVWRIQAGRGFNHF16:HPF16_0872   MELQQTHRKINRPLVSLALVGLLVSI-----TP----QKSHAAFFTTVIIPAIVGGIATGAAVGTVSGLLGWGLKQAEEANKTPDKPDKVWRIQAGRGFNH51:KHP_0833      MELQQTHRKINRPLVSLALVGLLVSI-----TP----QKSHAAFFTTVIIPAIVGGIATGAAVGTVSGLLGWGLKQAEEANKTPDKPDKVWRIQAGRGFNHF30:HPF30_0448   MELQQTHRKINRPLVSLALVGLLVSI-----TP----QKSHAAFFTTVIIPAIVGGIATGAAVGTVSGLLGWGLKQAEEANKTPDKPDKVWRIQAGRGFNHF57:HPF57_0902   MEIQQTHRKINRPLVSLALVGALVSI-----TP----QQSHAAFFTTVIIPAIVGGIATGAAVGTVSGLLGWGLKQAEEANKTPDKPDKVWRIQAGRGFNH52:aH52_1_061    ----------------------------------------------------------------------------------------------------HSJM:HPSJM_04485  MEIQQT-QKMNRPLVSLVLAGALISA-----IP----QESHAAFFTTVIIPAIVGGIATGTAVGTVSGLLSWGLKQAEEANKTPDKPDKVWRIQAGNGFNHG27:HPG27_840    MEIQQTHRKMNRPLVSLVLAGALISA-----IP----QESHAAFFTTVIIPAIVGGIATGTAVGTVSGLLSWGLKQAEEANKNPDKPDKVWRIQAGKGFNH266:HP0887       MEIQQTHRKINRPLVSLALVGALVSI-----TP----QQSHAAFFTTVIIPAIVGGIATGAAVGTVSGLLGWGLKQAEEANKTPDKPDKVWRIQAGKGFNHHPA:HPAG1_0867   MEIQQTHRKMNRPLVSLVLAGALISA-----IP----QQSHAAFFTTVIIPAIVGGIATGTAVGTVSGLLGWELKQAEEANKTPDKPDKVWRIQAGKGFNHP12:HPP12_0884   MEIQQTHRKINRPLVSLALVGALVSI-----TP----QQSHAAFFTTVIIPAIVGGIASGAAVGTVSGLLGWGLKQAEEANKTPDKPDKVWRIQAGKGFN                  101       111       121       131       141       151       161       171       181       191                         |         |         |         |         |         |         |         |         |         |         HB8:HPB8_666      NFPNKEYDLYKSLLSSKIDGGWDWGNAARHYWVKDGQWNKLEVDMQNAVGTYNLSGLINFTGGDLDVNMQKATLRLGQFNGNSFTSFKDGANRTTRVDFNHB38:HELPY_0872   NFPHKQYDLYKSLLSSKIDGGWDWGNAARHYWVKDGQWNKLEVDMQNAVGTYNLSGLINFTGGDLDVNMQKATLRLGQFNGNSFTSFKDGANRTTRVDFNHF32:HPF32_0462   EFPNKEYDLYKSLLSSKIDGGWDWGNAARHYWVKGGQWNKLEVDMKDAVGTYKLSGLRNYTGGDLDVNMQKATLRLGQFNGNSFTSFKDSADRTTRVDFNHF16:HPF16_0872   EFPNKEYDLYKSLLSSKIDGGWDWGNAARHYWVKGGQWNKLEVDMKDAVGTYKLSGLRNFTGGDLDVNMQKATLRLGQFNGNSFTSFKDSADRTTRVDFNH51:KHP_0833      EFPNKEYDLYKSLLSSKIDGGWDWGNAARHYWVKGGQWNKLEVDMKDAVGTYKLSGLRNYTGGDLDVNMQKATLRLGQFNGNSFTSFKDSADRTTRVDFNHF30:HPF30_0448   EFPNKEYDLYKSLLSSKIDGGWDWGNAARHYWVKGGQWNKLEVDMKDAVGTYKLSGLRNYTGGDLDVNMQKATLRLGQFNGNSFTSFKDSADRTTRVDFNHF57:HPF57_0902   EFPNKEYDLYKSLLSSKIDGGWDWGNAARHYWVKGGQWNKLEVDMKDAVGTYKLSGLINYTGGDLDVNMQKATLRLGQFNGNSFTSFKDNADRTTRVDFNH52:aH52_1_061    --------------------------------------------------------------------MQKATLRLGQFNGNSFTSYKDSADRTTRVDFNHSJM:HPSJM_04485  EFPNKEYDLYQSLLSSKIDGGWDWGNAARHYWVKGGQQNKLEVDMKDAVGTYKLSGLRNFTGGDLDVNMQKATLRLGQFNGNSFTSYKDSADRTTRVNFNHG27:HPG27_840    EFPNKEYDLYKSLLSSKIDGGWDWGNAARHYWVKGGQWNKLEVDMKDAVGTYKLSGLRNFTGGDLDVNMQKATLRLGQFNGNSFTSYKDAADRTTRVNFNH266:HP0887       EFPNKEYDLYRSLLSSKIDGGWDWGNAATHYWVKGGQWNKLEVDMKDAVGTYNLSGLRNFTGGDLDVNMQKATLRLGQFNGNSFTSYKDSADRTTRVDFNHHPA:HPAG1_0867   EFPNKQYDLYKSLLSSKIDGGWDWGNAARHYWVKGGQWNKLEVDMKDAVGTYTLSGLRNFTGGDLDVNMQKATLRLGQFNGNSFTSYKDSADRTTRVDFNHP12:HPP12_0884   EFPNKEYDLYRSLLSSKIDGGWDWGNAATHYWVKGGQWNKLEVDMKDAVGTYNLSGLRNFTGGDLDVNMQKATLRLGQFNGNSFTSYKDSADRTTRVDFN                  201       211       221       231       241       251       261       271       281       291                         |         |         |         |         |         |         |         |         |         |         HB8:HPB8_666      AKNILIDNFVEINNRVGSGAGRKASSTVLTLQASEKITSRENAEISLYDGATLNLVSSSNQSVDLYGKVWMGRLQYVGAYLAPSYSTINTSKVQGEMNFRHB38:HELPY_0872   AKNILIDNFVEINNRVGSGAGRKASSTVLTLQASEKITSRENAEISLYDGATLNLVSSSNHSVDLYGKVWMGRLQYVGAYLAPSYSTIDTSKVTGEMNFRHF32:HPF32_0462   AKNISIDNFLEINNRVGSGAGRKASSTVLTLQASEGITSGKNAEISLYDGATLNLASN---SVKLMGNVWMGRLQYVGAYLAPSYSTINTSKVTGEVNFNHF16:HPF16_0872   AKNILIDNFLEINNRVGSGAGRKASSTVLTLQASEGITSGKNAEISLYDGATLNLASN---SVKLMGNVWMGRLQYVGAYLAPSYSTINTSKVVGEVNFNH51:KHP_0833      AKNISIDNFLEINNRVGSGAGRKASSTVLTLQASEGITSGKNAEISLYDGATLNLASN---SVKLMGNVWMGRLQYVGAYLAPSYSTINTSKVTGEVNFNHF30:HPF30_0448   AKNISIDNFLEINNRVGSGAGRKASSTVLTLQASEGITSGKNAEISLYDGATLNLASN---SVKLMGNVWMGRLQYVGAYLAPSYSTINTSKVVGEVNFNHF57:HPF57_0902   AKNISIDNFIEINNRVGSGAGRKASSTVLTLLASEGITSGKNAEISLYDGATLNLASN---SVKLMGNVWMGRLQYVGAYLAPSYSTINTSKVTGEVNFNH52:aH52_1_061    AKNILIDNFLEINNRVGSGAGRKASSTVLTLQASEGITSGKNAEISLYDGATLNLASN---SVKLMGNVWMGRLQYVGAYLAPSYSTINTSKVAGEVNFNHSJM:HPSJM_04485  AKNISIDNFVEINNRVGSGAGRKASSTVLTLQASEGITSSKNAEISLYDGATLNLASN---SVKLMGNVWMGRLQYVGAYLAPSYSTINTSKVTGEVDFNHG27:HPG27_840    AKNISIDNFVEINNRVGSGAGRKASSTVLTLQASEGITSDKNAEISLYDGATLNLASS---SVKLMGNVWMGRLQYVGAYLAPSYSTINTSKVTGEVNFNH266:HP0887       AKNILIDNFLEINNRVGSGAGRKASSTVLTLQASEGITSSKNAEISLYDGATLNLASN---SVKLMGNVWMGRLQYVGAYLAPSYSTINTSKVTGEVNFNHHPA:HPAG1_0867   AKNISIDNFLEINNRVGSGAGRKASSTVLTLQASEKITSRENAEISLYDGATLNLASN---SVKLMGNVWMGRLQYVGAYLAPSYSTINTSKVTGEVDFNHP12:HPP12_0884   AKNISIDNFLEINNRVGSGAGRKASSTVLTLQASEGITSSKNAEISLYDGATLNLASS---SVKLMGNVWMGRLQYVGAYLAPSYSTINTSKVTGEVNFN                  301       311       321       331       341       351       361       371       381       391                         |         |         |         |         |         |         |         |         |         |         HB8:HPB8_666      HLAVGDHNAAQAGIIANKKTNIGVLDLWQSAGLNIIAPPEGGYKDKPSNTTQN---------NANNNQQNSAQNNSNTQVINPPNSAQKTEIQPTQVIDGHB38:HELPY_0872   HLAVGDQNAAQAGIIANKKTNIGVLDLWQSAGLSIITPPEGGYESKTKDTPQN----------------------------NPKNDAQKTEIQPTQVIDGHF32:HPF32_0462   HLTVGDRNAAQAGIIASKKTYIGTLDLWQSAGLNIIAPPEGGYKDKPNNTNSQSGAKNDKNESAKNDKQ-----DSNTQVINPPNSGQKTEIQPTQVIDGHF16:HPF16_0872   HLTVGDRNAAQAGIIASKKTYIGTLDLWQSAGLNIIAPPEGGYKDKPNNTNSQSGAKNDKNESAKNDKQ-----DSNTQVINPPNSGQKTEIQPTQVIDGH51:KHP_0833      HLTVGDRNAAQAGIIASKKTYIGTLDLWQSAGLNIIAPPEGGYKDKPNNTNSQSGAKNDKNESAKNDKQ-----DSNTQVINPPNSGQKTEIQPTQVIDGHF30:HPF30_0448   HLTVGDRNAAQAGIIASKKTYIGTLDLWQSAGLNIIAPPEGGYKDKPNNTNSQSGAKNDKNESAKNDKQESSQNNSNTQVINPPNSGQKTEIQPTQVIDGHF57:HPF57_0902   HLTVGDRNAAQAGIIASKKTYIGTLDLWQSAGLNIITPPEGGYKDKPNNTNSQSGAKNDKNESAKNDKQESSQNNSNTQVINPPNSGQKTEIQPTQVIDGH52:aH52_1_061    HLTVGDHNAAQAGIIASKKTYIGTLDLWQSAGLNIIAPPEGGYKDKPNNTNSQSG--------AKNDKQESSQNNSNTQVINPPNSGQKTEIQPTQVIDGHSJM:HPSJM_04485  HLTVGDHNAAQAGIIASNKTHIGTLDLWQSAGLNIIAPPEGGYKDKPNNTPSQSGTKNDKNESAKNDKQESSQNNSNTQVINPPNNTQKTEIQPTQVIDGHG27:HPG27_840    HLTVGDKNAAQAGIIASNKTHIGTLDLWQSAGLNIIAPPEGGYKDKPNNTPSQSGTKNDKNESAKNDKQESSQNNSNTQVINPPNSTQKTEIQPTQVIDGH266:HP0887       HLTVGDHNAAQAGIIASNKTHIGTLDLWQSAGLNIIAPPEGGYKDKPKDKPSNTTQNN-----ANNNQQNSAQNNSNTQVINPPNSAQKTEIQPTQVIDGHHPA:HPAG1_0867   HLTVGDKNAAQAGIIASNKTHIGTLDLWQSAGLNIIAPPEGGYKNQTNNTPSQSGAKNDKNESAKNDKQESSQNNSNTQVINPPNSTQKTEIQPTQVIDGHP12:HPP12_0884   HLTVGDRNAAQAGIIASNKTHIGTLDLWQSAGLNIIAPPEGGYKDKPNNTPSQSGAKNDKNESAKNDKQESSQNNSNTQVINPPNSAQKTEVQPTQVIDG                  401       411       421       431       441       451       461       471       481       491                         |         |         |         |         |         |         |         |         |         |         HB8:HPB8_666      PFAGGKDTVVNIFHLNTKADGTLRAGGFKASLSTNAAHLHIGEGGVNLSNQASGRTLLVENLTGNITVEGTLRVNNQVGGAAVAGSSANFEFKAGEDTNNHB38:HELPY_0872   PFAGGKDTVVNIFHLNTKADGTIKAGGFKASLSTNAAHLHIGEGGVNLSNQASGRSLLVENLTGNITVEGTLRVNNQVGGAAVAGSSANFEFKAGEDTNNHF32:HPF32_0462   PFAGAKDTVVNINRINTNADGTIKVGGYTASLTTNAANLNIGKGGVNLSNQASGRSLLVENLTGNITVDGALMVNNQVGGYALAGSSANFEFKAGVDTKNHF16:HPF16_0872   PFAGAKDTVVNINRINTNADGTIKVGGYTASLTTNAAHLNIGKGGVNLSNQASGRSLLVENLTGNITVDGALRVNNQVGGYALAGSSANFEFKAGVDTKNH51:KHP_0833      PFAGAKDTVVNINRINTNADGTIKVGGYTASLTTNAAHLNIGKGGVNLSNQASGRSLLVENLTGNITVDGALMVNNQVGGYALAGSSANFEFKAGVDTKNHF30:HPF30_0448   PFAGAKDTVVNINRINTNADGTIKVGGYTASLTTNAANLNIGKGGVNLSNQASGRSLLVENLTGNITVDGALMVNNQVGGYALAGSSANFEFKAGVDTKNHF57:HPF57_0902   PFAGAKDTVVNINRINTNADGTIKVGGYTASLTTNAAHLNIGKGGVNLSNQASGRSLLVENLTGNITVDGALMVNNQVGGYALAGSSANFEFKAGVDTKNH52:aH52_1_061    PFAGAKDTVVNINRINTNADGTIKVGGYTASLTTNAANLNIGKGGVNLSNQASGRSLLVENLTGNITVDGALMVNNQVGGYALAGSSANFEFKAGVDTKNHSJM:HPSJM_04485  PFAGGKDTVVNIDRINTNADGTIRVGGYKASLTTNAAHLHIGKGGVNLSNQASGRSLLVENLTGNITVDGPLRVNNQVGGYALAGSSANFEFKAGTDTKNHG27:HPG27_840    PFAGGKDTVVNINRINTNADGTIRVGGFKASLTTNAAHLHIGKGGVNLSNQASGRTLLVENLTGNITVDGPLRVNNQVGGYALAGSSANFEFKAGVDTKNH266:HP0887       PFAGGKDTVVNIDRINTNADGTIKVGGYKASLTTNAAHLHIGKGGINLSNQASGRTLLVENLTGNITVDGPLRVNNQVGGYALAGSSANFEFKAGTDTKNHHPA:HPAG1_0867   PFAGGKDTVVNINRINTNADGTIKVGGFKASLTTNAAHLHIGKGGVNLSNQASGRTLLVENLTGNITVDGPLRVNNQVGGYALAGSSANFEFKAGTDTKNHP12:HPP12_0884   PFAGGKDTVVNINRINTNADGTIRVGGYKASLTTNAAHLHIGKGGVNLSNQASGRTLLVENLTGNITVDGPLRVNNQVGGYALAGSSANFEFKAGTDTKN                  501       511       521       531       541       551       561       571       581       591                         |         |         |         |         |         |         |         |         |         |         HB8:HPB8_666      ATATFNNDIHLGKAVNLRVDAHTAYFNGNIYLGKSTNLRVNGHSAHFKNIDASKSDNGLNTSTLDFSGVTDKVNINKLTTSATNVNIKNFDIKELVVTTRHB38:HELPY_0872   ATATFNNDIHLGKAVNLRVDAHTANFNGNIYLGKSTNLRVNGHSAHFKNIDATKSDNGLNTSALDFSGVTDKVNINKLTTSATNVNIKNFDIKELVVTTRHF32:HPF32_0462   GTIAFNNNISLGRFVNLKASAHTVN---------------------FKDIDT--GNGGFNT--LDFSGVTNKVNINKLITASTNVAIKNFNINELLVKTNHF16:HPF16_0872   GTIAFNNNISLGRFVNLKASAHTVN---------------------FKDIDT--GNGGFNT--LDFSGVTNKVNINKLITASTNVAVKNFNINELLVKTNH51:KHP_0833      GTIAFNNNISLGRFVNLKASAHTVN---------------------FKDIDT--GNGGFNT--LDFSGVTNKVNINKLITASTNVAVKNFNINELLVKTNHF30:HPF30_0448   GTIAFNNNISLGRFVNLKASAHTVN---------------------FKDIDT--GNGGFNT--LDFSGVTNKVNINKLITASTNVAIKNFNINELLVKTNHF57:HPF57_0902   GTIAFNNNISLGRFVNLKASAHTVN---------------------FKDIDT--GNGGFNT--LDFSGVTNKVNINKLITASTNVAIKNFNINELLVKTNH52:aH52_1_061    GTIAFNNNISLGRFVNLKASAHTVN---------------------FKDIDT--GNGGFNT--LDFSGVTNKVNINKLITASTNVAIKNFNINELLVKTNHSJM:HPSJM_04485  GTATFNNDINLGRFVNLKVDAHTAN---------------------FKGIDT--GNGGFNT--LDFSGVTNKVNINKLITASTNVAVKNFNINELIVKTNHG27:HPG27_840    GTATFNNDISLGRFVNLKVDAHTAN---------------------FKGIDT--GNGGFNT--LDFSGVTDKVNINKLITASTNVAVKNFNINELIVKTNH266:HP0887       GTATFNNDISLGRFVNLKVDAHTAN---------------------FKGIDT--GNGGFNT--LDFSGVTGKVNINKLITASTNVAVKNFNINELVVKTNHHPA:HPAG1_0867   GTATFNNDISLGRFVNLKVDAHTAN---------------------FKGIDT--GNGGFNT--LDFSGVTDKVNINKLITASTNVAIKNFNINELLVKTNHP12:HPP12_0884   GTATFNNDISLGRFVNLKVDAHTAN---------------------FKGIDT--GNGGFNT--LDFSGVTDKVNINKLITASTNVAIKNFNINELLVKTN                  601       611       621       631       641       651       661       671       681       691                         |         |         |         |         |         |         |         |         |         |         HB8:HPB8_666      VQSFGQYTIFGEIIGDKSRIGVVSLQTGYNRAYSGGVTFKSGKKLVIDEIYHAPWNYFDARNVTDVEVNKRILFGAPGNIAGKTGLMFNNLTLNSNASMDHB38:HELPY_0872   VQSFGQYTIFGENIGDKSHIGVVSLQTGYSPAYSGGVTFKSGKKLVIDEIYHAPWNYFDARNVTDVEINKRILFGAPGNIAGKTGLMFNNLTLNSNASMDHF32:HPF32_0462   GISVGEYTNFSEDIGNQSRINTVRLETGTRSIYSGGVKFKGGEKLVINDFYYAPWNYFDARNIKNVEITNKLAFGPQGSPWGTAKLMFNNLTLGPNAVMDHF16:HPF16_0872   GISVGEYTNFSEDIGNQSRINTVRLETGTRSIYSGGVKFKGGEKLVINDFYYAPWNYFDARNIKNVEITNKLAFGPQGSPYGTAKLMFNNLTLGPNAVMDH51:KHP_0833      GISVGEYTNFSEDIGNQSRINTVRLETGTRSIYSGGVKFKGGEKLVINDFYYAPWNYFDARNIKNVEITNKLAFGPQGSPWGTAKLMFNNLTLGPNAVMDHF30:HPF30_0448   GISVGEYTNFSEDIGNQSRINTVRLETGTRSIYSGGVKFKSGEKLVINDFYYAPWNYFDARNIKNVEITNKLAFGPQGSPWGTAKLMFNNLTLGPNAVMDHF57:HPF57_0902   GISVGEYTNFSEDIGNQSRINTVRLETGTRSIYSGGVKFKSGEKLVINDFYYAPWNYFDARNIKNVEITNKLAFGPQGSPWGTAKLMFNNLTLGPNAVMDH52:aH52_1_061    GISVGEYTNFSEDIGNQSRINTVRLETGTRSIYSGGVKFKGGEKLVINDFYYAPWNYFDARNIKNVEITNKLAFGPQGSPWGTAKLMFNNLTLGPNAVMDHSJM:HPSJM_04485  GISVGEYTHFSEDIGSQSRINTVRLETGTRSIFSGGVKFKGGEKLVIDEFYYSPWNYFDARNIKNVEITRKLASSTPENPWGTSKLMFNNLTLGQNAVMDHG27:HPG27_840    GISVGEYTHFSEDIGSQSRINTVRLETGTRSIFSGGVKFKSGEKLVIDEFYYSPWNYFDARNVKNVEITRKFASSTPENPWGTSKLMFNNLTLGQNAVMDH266:HP0887       GVSVGEYTHFSEDIGSQSRINTVRLETGTRSIFSGGVKFKSGEKLVIDEFYYSPWNYFDARNIKNVEITRKFASSTPENPWGTSKLMFNNLTLGQNAVMDHHPA:HPAG1_0867   GVSVGEYTYFSEDIGSQSRINTVRLETGTRSIFSGGVKFKGGEKLVINDFYYAPWNYFDARNIKNVEITNKLAFGPQGSPWGTAKLMFNNLTLGQNAVMDHP12:HPP12_0884   GVSVGEYTHFSEDIGSQSRINTVRLETGTRSIFSGGVKFKGGEKLVINDFYYAPWNYFDARNIKNVEITNKLAFGPQGSPWGTSKLMFNNLTLGQNAVMD                  701       711       721       731       741       751       761       771       781       791                         |         |         |         |         |         |         |         |         |         |         HB8:HPB8_666      YGKDLDLTIQGHFTNNQGTMNLFVQDGRVATLNAGHQASMIFNNLVDSATGFYKPLIKINNAQNLTKNKEHVLVKARNIDYNLVGVQGASYDNISASNTNHB38:HELPY_0872   YGKDLDLTIQGHFTNNQGTMNLFVQDGRVATLNAGHQASMIFNNVVDSATGFYKPLIKINNAQNLTKNKEHVLVRARNIDYNLVGVQGASYDNISASNTNHF32:HPF32_0462   YSQFSNVTIQGNFINNQGTINYLVRGGNIETLNVGNAAVMSFNNDIDSATGFYKPLIKINSAQDLIKNKEHVLLKAKIIGYENA-----SLGTNSISNANHF16:HPF16_0872   YSQFSNVTIQGNFINNQGTINYLVRGGNIETLNVGNAAVMSFNNDIDSATGFYKPLVKINSAQDLIKNKEHVLLKAKIIGYENA-----SLGTNSISNANH51:KHP_0833      YSQFSNVTIQGNFVNNQGTINYLVRGGNIETLSIGNAAVMSFNNDIDSATGFYKPLIKINSAQDLIKNKEHVLLKAKIIGYENA-----SLGTNSISNANHF30:HPF30_0448   YSQFSNVTIQGNFINNQGTINYLVRGGNIETLSVGNAAVMSFNNDIDSATGFYKPLIKINSAQDLIKNKEHVLLKAKIIGYENA-----SLGTNSISNANHF57:HPF57_0902   YSQFSNVTIQGNFVNNQGTINYLVRGGNIETLSVGNAAVMSFNNDIDSATGFYKPLIKINSAQDLIKNKEHVLLKAKIIGYENA-----SLGTNSISNANH52:aH52_1_061    YSQFSNVTIQGNFINNQGTINYLVRGGNIETLSVGNAAVMSFNNDIDSATGFYKPLIKINSAQDLIKNKEHVLLKAKIIGYENA-----SLGTNSISNANHSJM:HPSJM_04485  YSQFSNLTIQGNFINNQGTINYLVRDGKVATLSVGNAAAMMFNNDIDSATGFYKPLIKINSAQDLIKNTEHVLLKAKIIGYGNV-----STGTNGISNVNHG27:HPG27_840    YSQFSNLTIQGDFINNQGTINYLVRGGKVATLSVGNAAAMMFNNDIDSATGFYKPLIKINSAQDLIKNTEHVLLKAKIIGYGNV-----STGTNSISNVNH266:HP0887       YSQFSNLTIQGDFINNQGTINYLVRGGQVATLNVGNAAAMFFSNNVDSATGFYQPLMKINSAQDLIKNKEHVLLKAKIIGYGNV-----SLGTNSISNVNHHPA:HPAG1_0867   YSQFSNLTIQGDFINNQGTINYLVRGGQVATLNVGNAAAMMFNNDIDSATGFYKPLIKINSAQDLIKNTEHVLLKAKIIGYGNV-----STGTNGISNVNHP12:HPP12_0884   YSQFSNLTIQGDFVNNQGTINYLVRGGQVATLNVGNAAAMFFNNNVDSATGFYQPLMKINSAQDLIKNKEHVLLKAKIIGYGNV-----SAGTNSISNVN                  801       811       821       831       841       851       861       871       881       891                         |         |         |         |         |         |         |         |         |         |         HB8:HPB8_666      LQEQFKERLALYNNNNRMDICVVRKDNLNDIKACGMAIGNQSMVNNPNNYKYLEGKAWKNTGINKTANNTTIAVNLGNNSTPTENGGNTTNLPTNTTNKAHB38:HELPY_0872   LQESFKERLALYNNNNRMDICVVRKDNLNDIKACGMAIGNQSMVNNPENYKYLEGKAWKNTGINKTANNTTIAVNLGNNSAPTSSESNTTNLPTNTTNKAHF32:HPF32_0462   LIEQFNERLALYNNNNRMDTCVVR--NTDDIKACGMAIGDQAMVNNPDNYKYLIGKAWKNIGISKTANGSKISVRYLGNATPAENGGNTTNLPTNTTNNAHF16:HPF16_0872   LIEQFNERLALYNNNNRMDTCVVR--NTDDIKACGMAIGDQAMVNNPDNYKYLIGKAWKNIGISKTANGSKISVRYLGNSTPTENGGNTTNLPTNTTNNAH51:KHP_0833      LIEQFNERLALYNNNNRMDTCVVR--NTDDIKACGMAIGDQAMVNNPDNYKYLIGKAWKNIGISKTANSSKISVRYLGNATPTENGGNTTNLPTNTTNNAHF30:HPF30_0448   LIEQFNERLALYNNNNRMDTCVVR--NTDDIKACGMAIGDQAMVNNPDNYKYLIGKAWKNIGISKTANGSKISVRYLGNATPAENGGNTTNLPTNTTNNAHF57:HPF57_0902   LIEQFNERLALYNNNNRMDTCVVR--NTDDIKACGMAIGDQAMVNNPDNYKYLIGKAWKNIGISKTANGSKISVRYLGNATPAENGGNTTNLPTNTTNNAH52:aH52_1_061    LIEQFNERLALYNNNNRMDTCVVR--NTDDIKACGMAISDQAMVNNPDNYKYLIGKAWKNIGISKTANGSKISVRYLGNATPAENGGNTTNLPTNATNNAHSJM:HPSJM_04485  LEEQFKERLALYNNNNRMDTCVVR--NTDDIKACGMAIGNQSMVNNPDNYKYLIGKAWKNIGISKTANGSKISVYYLGNSTPSENGGNTTNLPTNTTNNAHG27:HPG27_840    LEEQFKERLALYNNNNRMDTCVVR--NTDDIKACGMAIGNQSMVNNPDNYKYLIGKAWKNIGISKTANGSKISVYYLGNSTPTENGGNTTNLPTNTTNNAH266:HP0887       LIEQFKERLALYNNNNRMDICVVR--NTDDIKACGTAIGNQSMVNNPDNYKYLIGKAWKNIGISKTANGSKISVYYLGNSTPTEKGGNTTNLPTNTTSNVHHPA:HPAG1_0867   LEEQFKERLALYNNNNRMDTCVVR--NTDDIKTCGMAIGNQSMVNNPDNYKYLIGKAWKNIGISKTANGSKISVYYLGNSTPTENSGNTTNLPTNTTSNAHP12:HPP12_0884   LIEQFKERLALYEHNNRMDICVVR--NTDDIKACGTAIGNQSMVNNPDNYKYLIGKAWKNIGISKTANGSKISVHYLGNSTPTENSGNTTNLPTNTTSNA                  901       911       921       931       941       951       961       971       981       991                         |         |         |         |         |         |         |         |         |         |         HB8:HPB8_666      RFASYALIKNAPFAH-SATPNLVAINQHDFGTIESVFELADRSKDIDTLYTHSGAQGRDLLQTLLIDSHDAGYARTMIDATNANEITQQLNAATTTLNNIHB38:HELPY_0872   RFASYALIKNAPFAHYNATPNLVAINQHDFGTIESVFELANRSKDIDTLYANSGAQGRDLLQTLLIDSHDAGYARTMIDATSANEITKQLNAATTTLNNIHF32:HPF32_0462   RFASYALIKNAPFAQTSATPNLVAINKHDFGTIESVFELANRSKDIDTLYANSGAQGRDLLQTLLIDSHNAGYARTMIDATSANEITKQLNTATDALNNIHF16:HPF16_0872   RFARYALIKNAPFAQTSATPNLVAINKHDFGTIESVFELANRSKDIHALYAHSGAQGRDLLQTLLIDSHNAGYARTMIDATSANEITKQLNEANSALNNIH51:KHP_0833      RFARYALIKNAPFAQTNATPNLVAINKHNFGTIESVFELANRSEDIDTLYAHSGTQGRDLLQTLLIDSHNAGYARTMINATSANEITKQLNEANSALNNIHF30:HPF30_0448   RFASYALIKNAPFAQTNATPSLVAINKHNFGTIESVFELANRSEDIDTLYANSGAQGRDLLQTLLIDSHDAGYARTMIDATSTNEITKQLNEANSALNNIHF57:HPF57_0902   RFASYALIKNAPFAQTSATPSLVAINKHNFGTIESVFELANRSEDIDTLYANSGAQGRDLLQTLLIDSHDAGYARTMIDATSANEITKQLNTATDALNNIH52:aH52_1_061    RFARYALIKNAPFAQTNATPNLVAINKHNFGTIESVFELANRSDDIDTLYAHSGVQGRDLLQTLLIDSHNAGYARTMIDATSANEITKQLNEANSALNNIHSJM:HPSJM_04485  RSANYALVKNAPFAH-SATPNLVAINQHDFGTIESVFELANRSKDIDTLYTHSGAKGRDLLQTLLIDSHDAGYARQMIDNTSTGEITKQLNAATTTLNNIHG27:HPG27_840    RSANYALVKNAPFAH-SATPNLVAINQHDFGTIESVFELANRSKDIDTLYTHSGVQGRDLLQTLLIDSHDAGYARQMIDNTSTGEITKQLNAATDALNNIH266:HP0887       RSANNALAQNAPFAQPSATPNLVAINQHDFGTIESVFELANRSKDIDTLYANSGAQGRDLLQTLLIDSHDAGYARQMIDNTSTGEITKQLNAATTTLNNIHHPA:HPAG1_0867   RSANNALAQNAPFAQPSATPNLVAINQHDFGTIESVFELANRSSDIDTLYTHSGAQGRDLLQTLLIDSHDAGYARKMIDNTSTGEITKQLNTATTTLNNVHP12:HPP12_0884   RSAKNALAQNAPFAQPSATPSLVAINQHDFGTIESVFELANRSKDIDTLYTHSGAQGRNLLQTLLIDSHDAGYARQMIDNTSTGEIIKQLNAATTTLNNV                  1001      1011      1021      1031      1041      1051      1061      1071      1081      1091                        |         |         |         |         |         |         |         |         |         |         HB8:HPB8_666      ASLEHKTSGLQTLSLSNAMILNSRLVNLSRRHTNHIDSFAKRLQALKDQRFASLESAAEVLYQFAPKYEKPTNVWATLLG--------------------HB38:HELPY_0872   ASLEHKTSGLQTLSLSNAMILNSRLVNLSRRHTNHIDSFAKRLQALKDQRFASLESAAEVLYQFAPKYEKPTNVWANAIGGTSLNNGSNASLYGTSAGVDHF32:HPF32_0462   ASLEHKTSGLQTLSLSNAMILNSRLVNLSRRHTNNIDSFAKRLQALKDQKFASLESAAEVLYQFAPKYEKPTNVWANAIGGASLNSGGNTSLYGTSAGVDHF16:HPF16_0872   ASLEHKTSGLQTLSLSNAMILNSRLVNLSRRHTNNIDSFARRLQALKDQRFASLESAAEVLYQFAPKYEKPTNVWANAIGGASLNSGGNTSLYGTSAGVDH51:KHP_0833      ASLEHKTNGLQTLSLSNAMILNSRLVNLSRRHTNNIDSFAKRLQALKDQRFASLESAAEVLYQFAPKYEKPTNVWANAIGGASLNSGGNTSLYGTSAGVDHF30:HPF30_0448   ASLDHKTSGLQTLSLSNAMILNSRLVNLSRRHTNNIDSFAKRLQALKDQKFASLESAAEVLYQFAPKYEKPTNVWANAIGGASLNSGGNTSLYGTSAGVDHF57:HPF57_0902   ASLEHKTSGLQTLSLSNAMILNSRLVNLSRRHTNNIDSFAKRLQALKDQRFASLESAAEVLYQFAPKYEKPTNVWANAIGGASLNSGGNTSLYGTSAGVDH52:aH52_1_061    ASLEHKTSGLQTLSLSNAMILNSRLVNLSRRHTNNIDSFAQRLQALKGQRFASLESAAEVLYQFAPKYEKPTNVWANAIGGASLNSGGNTSLYGTSAGVDHSJM:HPSJM_04485  ASLEHKTSGLQTLSLSNAMILNSRLVNLSRRHTNNIDSFAQRLQALKDQRFASLESAAEVLYQFAPKYEKPTNVWANAIGGTSLNNGSNASLYGTSAGVDHG27:HPG27_840    ASLEHKTSGLQTLSLSNAMILNSRLVNLSRKHTNHIDSFAQRLQALKGQRFASLESAAEVLYQFAPKYEKPTNVWANAIGGASLNNGGNASLYGTSAGVDH266:HP0887       ASLEHKTSSLQTLSLSNAMILNSRLVNLSRRHTNNIDSFAQRLQALKDQKFASLESAAEVLYQFAPKYEKPTNVWANAIGGTSLNNGGNASLYGTSAGVDHHPA:HPAG1_0867   ASLEHKTSGLQTLSLSNAMILNSRLVNLSRRHTNNIDSFAKRLQALKDQRFASLESAAEVLYQFAPKYEKPTNVWANAIGGTSLNNGGNASLYGTSAGVDHP12:HPP12_0884   ASLEHKQSGLQTLSLSNAMILNSRLVNLSRRHTNNIDSFAQRLQALKDQKFASLESAAEVLYQFAPKYEKPTNVWANAIGGTSLNNGGNASLYGTSAGVD                  1101      1111      1121      1131      1141      1151      1161      1171      1181      1191                        |         |         |         |         |         |         |         |         |         |         HB8:HPB8_666      ----------------------------------------------------------------------------------------ERA---------HB38:HELPY_0872   AYLNGEVEAIVGGFGSYGYSSFSNQANSLNSGANNTNFGVYSRLFANQHEFDFEAQGALGSDQSSLNFKSALLQDLNQSYNYLAYSAATRASYGYDFAFFHF32:HPF32_0462   AYLNGEVEAIVGGFGSYGYSSFSNQANSLNSGANNANFGVYSRIFANKHEFDFEAQGALGSDQSSLNFKSALLRDLNQSYNYLAYGAATRASYGYDFAFFHF16:HPF16_0872   AYLNEKVEAIVGGFGSYGYSSFNNQANSLNSGANNANFGVYSRIFANRHEFDFEAQGAVGSDQSSLNFKSALLRDLNQSYNYLAYGAATRASYGYDFAFFH51:KHP_0833      AYLNEKVEAIVGGFGSYGYSSFSNQANSLNSGANNANFGVYSRIFANRHEFDFEAQGAVGSDQSSLNFKGALLRDLNQSYNYLAYGASTRASYGYDFAFFHF30:HPF30_0448   AYLNGEVEAIVGGFGSYGYSSFSNQANSLNSGANNANFGVYSRIFANQHEFDFEAQGAVGSDQSSLNFKGALLRDLNQSYNYLAYGASARASYGYDFAFFHF57:HPF57_0902   AYLNGEVEAIVGGFGSYGYSSFNNQANSLNSGANNANFGAYSRIFANRHEFDFEAQGAVGSDQSSLNFKSALLRDLNQSYNYLAYGASTRASYGYDFAFFH52:aH52_1_061    AYLNEKVEAIVGGFGSYGYSSFNNQANSLNSGANNANFGVYSRIFANRHEFDFEAQGALGSDQSSLNFKSALLRDLNQSYNYLAYGAATRASYGYDFAFFHSJM:HPSJM_04485  AYLNGEVEAIVGGFGSYGYSSFNNRANSLNSGANNTNFGVYSRLFANQHEFDFEAQGALGSDQSSLNFKSTLLQDLNQSYNYLAYSAATRASYGYDFAFFHG27:HPG27_840    AYLNGEVEAIVGGFGSYGYSSFSNRANSLNSGANNANFGVYSRIFANQHEFDFEAQGALGSDQSSLNFKSALLQDLNQSYHYLAYSAATRASYGYDFAFFH266:HP0887       AYLNGEVEAIVGGFGSYGYSSFNNQANSLNSGANNTNFGVYSRIFANQHEFDFEAQGALGSDQSSLNFKSALLRDLNQSYNYLAYSAATRASYGYDFAFFHHPA:HPAG1_0867   AYLNGEVEAIVGGFGSYGYSSFSNQANSLNSGANNTNFGVYSRIFANQHEFDFEAQGALGSDQSSLNFKSALLQDLNQSYHYLAYSAATRASYGYDFAFFHP12:HPP12_0884   AYLNGEVEAIVGGFGSYGYSSFSNQANSLNSGANNTNFGVYSRLFANQHEFDFEAQGALGSDQSSLNFKSALLRDLNQSYNYLAYSAATRASYGYDFAFF                  1201      1211      1221      1231      1241      1251      1261      1271      1281      1291                        |         |         |         |         |         |         |         |         |         |         HB8:HPB8_666      ----------------------------------------------------------------------------------------------------HB38:HELPY_0872   RNALVLKPSVGVSYNHLGSTNFKSNSNQKVALKNGASSQHLFNASANVEARYYYGDTSYFYMNAGVLQEFAHVGSNNAASLNTFKVNAARNPLNTHARVMHF32:HPF32_0462   RNALVLKPSVGVSYNHLGSTNFESNSTNKTALKNGASSQHLFNASANVEARYYYGDTSYFYMNAGVLQEFANFGSSNAVSLNTFKVNAARNPLNTHARVMHF16:HPF16_0872   RNALVLKPSVGVSYNHLGSTNFESNSTNKTALKNGASSQHLFNASANVEARYYYGDTSYFYMNAGVLQEFANFGSSNAVSLNTFKVNAARNPLNTHARVMH51:KHP_0833      RNALVLKPSVGVSYNHLGSTNFESNSTNKTALKNGASSQHLFNASANVEARYYYGDTSYFYMNAGVLQEFANFGSSNAVSLNTFKVNAARNPLNTHARVMHF30:HPF30_0448   RNALVLKPSVGVNYNHLGSTNFESNSTHKAALKNGASSQHLFNASANVEARYYYGDTSYFYMNAGVLQEFTNFGSSNAVSLNTFKVNAARNPLNTHARVMHF57:HPF57_0902   RNALVLKPSVGVSYNHLGSTNFESNSTHKTALKNGASSQHLFNASANVEARYYYGDTSYFYMNAGVLQEFANFGSSNALSLNTFKVNTARNPLNTHARVMH52:aH52_1_061    RNALVLKPSVGVSYNHLGSTNFESNSTNKTALKNGANSQHLFNASANVEARYYYGDTSYFYMNAGVLQEFANFGSSNALSLNTFKVNAARNPLNTHARVMHSJM:HPSJM_04485  RNALVLKPSVGVSYNHLGSTNFKSNSTNKVALKNGSSSQHLFNANANVEARYYYGDTSYFYLHAGVLQEFAHFGSNDVASLNTFKINAARSPLSTYARAMHG27:HPG27_840    RNALVLKPSVGVSYNHLGSTNFKS-SSNQVALKNGSSSQHLFNANANVEARYYYGDTSYFYMNAGVLQEFARFGSNNAASLNTFKVNTARNPLNTHARVMH266:HP0887       RNALVLKPSVGVSYNHLGSTNFKSNSNQ-VALKNGSSSQHLFNASANVEARYYYGDTSYFYMNAGVLQEFANFGSSNAVSLNTFKVNAAHNPLSTHARVMHHPA:HPAG1_0867   RNALVLKPSVGVSYNHLGSTNFKSNSNQKVALKNGSSSQHLFNASANVEARYYYGDTSYFYMNAGVLQEFANFGSSNAVSLNTFKVNAARNPLNTHARVMHP12:HPP12_0884   RNALVLKPSVGVSYNHLGSTNFKSNSTNQVALKNGSSSQHLFNASANVEARYYYGDTSYFYMNAGVLQEFANFGSSNAVSLNTFKVNAARNPLNTHARVM                  1301      1311      1321      1331      1341                  |         |         |         |         |HB8:HPB8_666      ----------------------------------------HB38:HELPY_0872   MGGELQLAKEVFLNLGVVYLHNLISNIGHFASNLGMRYSFHF32:HPF32_0462   MGGELQLAKEVFLNLGFIYLHNLISNAGHFASNLGMRYSFHF16:HPF16_0872   MGGELKLAKEVFLNLGFVYLHNLISNIGHFASNLGMRYSFH51:KHP_0833      MGGELKLAKEVFLNLGFIYLHNLISNAGHFASNLGMRYSFHF30:HPF30_0448   MGGELKLAKEVFLNLGFIYLHNLISNIGHFASNLGMRYSFHF57:HPF57_0902   MGGELKLAKEVFLNLGFIYLHNLISNAGYFASNLGMRYSFH52:aH52_1_061    MGGELKLAKEVFLNLGFIYLHNLISNAGHFASNLGMRYSFHSJM:HPSJM_04485  MGGELRLAKEVFLNLGVVYLHNLISNASHFASNLGMRYSFHG27:HPG27_840    MGGELQLAKEVFLNLGVVYLHNLISNIGHFASNLGMRYSFH266:HP0887       MGGELKLAKEVFLNLGFVYLHNLISNIGHFASNLGMRYSFHHPA:HPAG1_0867   MGGELKLAKEVFLNLGFVYLHNLISNIGHFASNLGMRYSFHP12:HPP12_0884   MGGELKLAKEVFLNLGFVYLHNLISNIGHFASNLGMRYSF
