# Supplementary material for: Evolution in an oncogenic bacterial species with extreme genome plasticity: Helicobacter pylori East Asian genomes
Source: BMC Microbiol. 2011 May 16;11:104. doi: 10.1186/1471-2180-11-104 (PMC3120642; doi:10.1186/1471-2180-11-104)
Supplement: Additional file 6 — Multiple sequence alignments of diverged genes. [file 1471-2180-11-104-S6.ZIP › Diverged_genes_multiple_seuence_alignments/HP0898_hypD.mfa.rtf]

                  1         11        21        31        41        51        61        71        81        91                          |         |         |         |         |         |         |         |         |         |         HB8:HPB8_654      MSVDHLISPFRDKRTLLALSDAIKKLAFKLEKKLVIMEVCGGHTHSLMKYGLLDLMPKNLEFVHGPGCPVCVMPRARLDEAYELATIKDSIILSLGDMMKH266:HP0898       MSVDHLISPFRDKRTLLALSNAIKKLAFKLEKKLVIMEVCGGHTHSIMKYGLLDLMPNNLEFVHGPGCPVCVMPRARLDEAYELATIKDSIVLSLGDMMRHB38:HELPY_0882   MSVDHLISPFRDKRVILALSNEIKKLAFKLTKKLVIMEVCGGHTHSIMKYGLLDLMPNNLEFVHGPGCPVCVMPRARLDEAYELATIKDSIVLSLGDMMKHHPA:HPAG1_0878   MSVDHLISPFRDKRIILALSNAIKKLAFKLEKKLVIMEVCGGHTHSIMKYGLLDLMPNNLEFVHGPGCPVCVMPRARLDEAYELASMKDSIVLSLGDMMKHP12:HPP12_0894   MSVDHLISPFRDKKTLLALSNAIKKLAFKLEKKLVIMEVCGGHTHSLMKYGLLDLMPKNLEFVHGPGCPVCVMPRARLDEAYELATIKDSIILSLGDMMKHSJM:HPSJM_04560  MSVDHLISPFRDKQTLLALSNAIKKLAFKLEKKLVIMEVCGGHTHSLMKYGLLDLMPKNLEFAHGPGCPVCVMPRARLDEAYELAMIKDSIILSLGDMMKHG27:HPG27_852    MSVDHLISPFRDKQTLLALSNAIKKLAFKLEKKLVIMEVCGGHTHSIMKYGLLDLMPNNLEFVHGPGCPVCVMPRARLDEAYELASMKDSIVLSLGDMMRHF32:HPF32_0453   MSVNHLIAPFRDKQTILALSNEIKKLAFKLEKKLVIMEVCGGHTHSIMKYGLLDLMPKNLEFVHGPGCPVCVMPRARLDEAYELATIKDSIVLSLGDMMKH52:HPKB_0868     MSVNHLIAPFRDKQTILALSNEIKKLAFKLEKKLVIMEVCGGHTHSIMKYGLLDLMPKNLEFVHGPGCPVCVMPRARLDEAYELATIKDSIILSLGDMMKHF57:HPF57_0910   MSVNHLIAPFRDKQTILALSNGIKKLAFKLEKKLVIMEVCGGHTHSIMKYGLLDLMPKNLEFVHGPGCPVCVMPRARLDEAYELAAIKDSIVLSLGDMMKH51:KHP_0841      MSVNHLIAPFRDKQTILALSNEIKKLASKLEKKLVIMEVCGGHTHSIMKYGLLDLMPKNLEFVHGPGCPVCVMPRARLDEAYELATIKNSIILSLGDMMKHF30:HPF30_0439   MSVNHLIAPFRDKQTILALSNGIKKLASKLEKKLVIMEVCGGHTHSIMKYGLLDLMPKNLEFVHGPGCPVCVMPRARLDEAYELATIKDSIILSLGDMMKHF16:HPF16_0881   MSVNHLIAPFRDKQTILALSNGIKKLASKLEKKLVIMEVCGGHTHSIMKYGLLDLMPKNLEFVHGPGCPVCVMPRARLDEAYELAAIKDSIVLSLGDMMK                  101       111       121       131       141       151       161       171       181       191                         |         |         |         |         |         |         |         |         |         |         HB8:HPB8_654      VPGSYGSLIQAREKGLDARFLYSPMQALEIAKENPTKKVIYIAIGFETTTPMSASVLLNAKKEKLKNLFFHINHILVPPSVSAILKDPACQINALLAPSHH266:HP0898       VPGSYGSLIQAREKGLDARFLYSPMQALEIAKENPTKKVIYIAIGFETTTPMSASVLWSAKKEKINNLFFHINHILVPPSVSAILKDPACQINALLAPSHHB38:HELPY_0882   VPGSYGSLIQAREKGLDARFLYSPMQALEIAKENPTKKVIYIAIGFETTTPMTASVLLNAKKEKINNLFFHINHILVPPSVSAILKDPACQINALLAPSHHHPA:HPAG1_0878   VPGSYGSLIQAREKGLDARFLYSPMQALEIAKENPHKKVIYIAIGFETTTPMTASVLLNAKKEKINNLFFHTNHLLVPPSVSAILKDPACQINALLAPSHHP12:HPP12_0894   VPGSYGSLIQAREKGLDARFLYSPMQALEIAKENPHKKVIYIAIGFETTTPMSASVLLNAKKEKINNLFFHTNHILVPPSVSAILKDPACQINALLAPSHHSJM:HPSJM_04560  VPGSYGSLIQAREKGLDARFLYSPMQALEIAKENPTKKVIYIAIGFETTTPMTASVLNNAKKEKLKNLFFHINHILVPPSVSAILKDPACQINALLAPSHHG27:HPG27_852    VPGSYGSLIQAREKGLDARFLYSPMQALEIAKENPTKKVIYIAIGFETTTPMTASVLKNAQKEKLKNLFFHINHILVPQSVSAILEDPACQINALLAPSHHF32:HPF32_0453   VPGSYGSLIQAREKGLDARFLYSPMQALEIAKENPHKKVIYIAIGFETTTPMSASVLLNAKKEKIRNLFFHINHLLVPPSVSAILNDPACQINALLAPSHH52:HPKB_0868     VPGSYGSLIQAREKGLDVRFLYSPMQALEIAKENPHKKVIYIAIGFETTTPMSASVLLNAKKEKIRNLFFHINHILVPPSVSAILKDPACQINALLAPSHHF57:HPF57_0910   VPGSYGSLIQAREKGLDARFLYSPMQALETAKENPHKKVIYIAIGFETTTPMSASVLLNAKKEKIRNLFFHINHLLVPPSVSAILQDPACQINALLAPSHH51:KHP_0841      VPGSYGSLIQAREKGLDARFLYSPMQALEIAKENPHKKVIYIAIGFETTTPMSASVLLNAKKEKIHNLFFHINHLLVPPSVSAILNDPACRINALLAPSHHF30:HPF30_0439   VPGSYGSLIQAREKGLDARFLYSPMQALEIAKENPHKKVIYIAIGFETTTPMSASVLLNAKKEKIRNLFFHINHILVPPSVSAILHDPACQINALLAPSHHF16:HPF16_0881   VPGSYGNLIQAREKGLDARFLYSPMQALEIAKENPHKKVIYIAIGFETTTPMSASVLLNAKKEKIRNLFFHINHILVPPSVSAILQDPACQINALLAPSH                  201       211       221       231       241       251       261       271       281       291                         |         |         |         |         |         |         |         |         |         |         HB8:HPB8_654      VSVISGAQIYSPLVDRFKLPIIVSGFEPVDILESVLMLLKQALNKEAKLEIQYKRAVSFEGNVKAQALVNACMEVRENFEWRGLGNIKRSALKLKEAFASH266:HP0898       VSVISGAQIYAPLVDRFKIPIIVSGFEPVDILESVLMLIKQALNKEAKLEIQYKRAVSFEGNVKAQELVNACMEVRENFEWRGLGNIKRSALKLKEAFASHB38:HELPY_0882   VSVISGAQIYAPLVDRFKIPIIVSGFEPVDILESVLMLIKQALNKEAKLEIQYKRAVSFEGNTKAQELVNACMEVRENFEWRGLGNIKRSALKLKEIFASHHPA:HPAG1_0878   VSVISGAQIYAPLVDRFKLPIIVSGFEPVDILESVLMLLKQALNKEAKLEIQYKRAVSYEGNVKAQELVNACMEVRENFEWRGLGNIKHSALKLKEAFASHP12:HPP12_0894   VSVISGAQIYAPLVDRFKIPIIVSGFEPVDILESVLMLLKQALNKEAKLEIQYKRAVSYEGNVKAQELVNACMEVRENFEWRGLGNIKHSALKLKETFASHSJM:HPSJM_04560  VSVISGAQIYAPLVDRFKLPIIVSGFEPVDILESVLMLLKQALNKEAKLEIQYKRAVSYEGNTKAQELVNACMEVRENFEWRGLGNIKHSALKLKEIFASHG27:HPG27_852    VSVISGAQIYSPLVDRFKLPIIVSGFEPVDILESVLMLLKQALNKEAKLEIQYKRAVSFEGNVKAQELVNACMEVRENFEWRGLGNIKRSALKLKEAFASHF32:HPF32_0453   VSVISGASIYAPLIHRFKLPIVVSGFEPVDILESVLMLIKQALKKEAKLEIQYKRAVSYEGNMKAQELVNACMEVRENFEWRGLGNIKRSALKLKETFASH52:HPKB_0868     VSVISGAQIYAPLIHRFKLPIVVSGFEPVDILESVLMLLKQALKKEAKLEIQYKRAVSYEGNVKAQELVNACMEVRENFEWRGLGNIKHSALKLKETFASHF57:HPF57_0910   VSVISGAQIYAPLTERFKLPIVVSGFEPVDILESVLMLLKQALKKEAKLEIQYKRAVSYEGNVKAQELVNACMEVRENFEWRGLGNIKHSALKLKETFASH51:KHP_0841      VSVISGAQIYAPLVDRFKLPIVVSGFEPVDILESVLMLIKQALKKEAKLEIQYKRAVSYEGNVKAQELVNACMEVRENFEWRGLGNIKHSALKLKETFASHF30:HPF30_0439   VSVISGAQIYAPLIDRFKLPIVVSGFEPVDILESVLMLIKQALKKEAKLEIQYKRAVSYEGNVKAQELVNACMEVRENFEWRGLGNIKHSALKLKETFASHF16:HPF16_0881   VSVISGAQIYAPLIDRFKLPIVVSGFEPVDILESVLMLIKQALKKEAKLEIQYKRAVSYEGNVKAQELVNACMEVRENFEWRGLGNIKRSALKLRETFVS                  301       311       321       331       341       351       361       371                  |         |         |         |         |         |         |         |HB8:HPB8_654      YDAEKVFKEYLSHKIIKENKACKCGEILKGIAKPLDCSLFATTCTPQNPIGSCMVSSEGACAAYYRYKRVH266:HP0898       YDAEEVFKEYLSHKTSKENKACKCGEILKGIAKPLDCSLFATTCTPQNPIGSCMVSSEGACAAYYRYKRVHB38:HELPY_0882   YDAEKVFKAYLSHKTSKENKACKCGEILKGIAKPLDCSLFATTCTPQNPIGSCMVSSEGACAAYYRYKRVHHPA:HPAG1_0878   YDAEKVFKEYLSHKTSKENKACKCGEILKGIAKPLDCSLFATICTPQNPIGSCMVSSEGACAAYYRYKRVHP12:HPP12_0894   YDAEKVFKEYLSHKISKENKACKCAEILKGIAKPLDCSLFATTCTPQNPIGSCMVSSEGACAAYYRYKRVHSJM:HPSJM_04560  YDAEKVFKAYLSHKTSKENKACKCGEILKGIAKPLDCSLFATTCTPQNPIGSCMVSSEGACAAYYRYKRVHG27:HPG27_852    YDAEKVFKEHLTHKTSKENKACKCGEILKGIAKPLDCSLFATTCTPQNPIGSCMVSSEGACAAYYRYKRVHF32:HPF32_0453   YDAEKVFKEHLSHKTSKENKACKCGEILKGIAKPLDCALFAKTCTPQNPIGSCMVSSEGACAAYYRYKRVH52:HPKB_0868     YDAEKVFKEHLSHKTSKENKACKCGEILKGIAKPLDCALFAKTCTPQNPIGSCMVSSEGACAAYYRYKRVHF57:HPF57_0910   YDAEKVFKEHLSHKTSKENKACKCAEILKGIAKPLDCALFAKTCTPQNPIGSCMVSSEGACAAYYRYKRVH51:KHP_0841      YDAEKVFKEHLSHKTSKENKACKCGEILKGTAKPLDCALFAKTCTPQNPIGSCMVSSEGACAAYYRYKRVHF30:HPF30_0439   YDAEKVFQEHLSHKTSKENKACKCAEILKGTAKPLDCALFAKTCTPQNPIGSCMVSSEGACAAYYRYKRVHF16:HPF16_0881   YDAEKVFKEHLSHKTPKENKACKCGEILKGIAKPLDCSLFAKTCTPQNPIGSCMVSSEGACAAYYRYKRV
