# Supplementary material for: Evolution in an oncogenic bacterial species with extreme genome plasticity: Helicobacter pylori East Asian genomes
Source: BMC Microbiol. 2011 May 16;11:104. doi: 10.1186/1471-2180-11-104 (PMC3120642; doi:10.1186/1471-2180-11-104)
Supplement: Additional file 6 — Multiple sequence alignments of diverged genes. [file 1471-2180-11-104-S6.ZIP › Diverged_genes_multiple_seuence_alignments/HP0906_fliK.mfa.rtf]

                  1         11        21        31        41        51        61        71        81        91                          |         |         |         |         |         |         |         |         |         |         HB8:HPB8_646      MPSPINPIHTNANANANALI--NSGAKNE--DTKNAPKSASKDFSKILNQKISKDKTAPKE--DSN--ASKVTPKD----AKEDAKALEKTPTLPHQHAQHHPA:HPAG1_0888   MPSPINPIHTNANANASTLI--NSGAKNE--DTKNAPKSASKDFSKILNQKISKDKTAPKE--NPN--ALKATPKDAKEGAKEDAKTLEKTPTPHHQHAQHP12:HPP12_0904   MPSPVNPIYTNANA-----L--NSGAKNE--DTKNAPKSASKDFSKILNQKISKDKTASKE--NPN--ALKATPK--------DSKVLEKTPTPHPQHAQHG27:HPG27_857    MPSPINPIHTNASANASTLI--NSGAKNK--DTKNAPKSASKDFSKILNQKISKDKTAPKE--NPN--ALKATPKN----TKEDAKVLEKTPTLQPQHAQHB38:HELPY_0890   MPSPVNPIHTNANA-----L--NSGAKNE--DTKNAPKSASKDFSKILNQKISKDKTTPKE--DPN--ALKATPKD----AKEDAKALEKAPTPHHQHAQH266:HP0906       MPSPINPIHTNASANANA-L--NSGAKNE--DAKNAPKSASKDFSKILNQKISKDKTAPKE--NPN--ALKTTPQNSKEGAKEDAKTLEKTPTLPHQHAQHF32:HPF32_0448   MPSPINPIHTNASTNANA-L--NNGAKNGVKDTKNAPKSASKDFSKILNQKISKDKTAPKE--SPS--ALKALPKD----AKKDAKELEKTPTPHHQHAQH51:KHP_0846      MPSPINPIHTNAS--ANA-L--NSGAKNEVKDTKNAPKSTSKDFSKILNQKISKDKTAPKE--NPN--ALKATPKD----AKKDAKELEKTPTPHHQHAQH52:HPKB_0873     MPSPINPIHTNAN--ASA-L--NSGAKNE--DTKNAPKSASKDFSKILNQKISKDKTAPKE--NPN--ALKATPKD----AKKDAKELEKTPTPHHQHAQHF57:HPF57_0916   MPSFVNPIHTNASANASA-L--NSGAKNE--DTKNAPKSASKDFSKILNQKISKDKTAPKE--NPN--ALKAVPKD----AKQNAKEPEKTPTPHHQHAQHF30:HPF30_0433   MPSPINPIHTNASANANA-L--NSGAKNEVKDTKNAPKSASKDFSKILNQKISKDKTAPKE--SPNSSALKAVPKD----AKQNAKELEKTPTPHHQHAQHF16:HPF16_0887   MPSPINPIHTNASANANA-LIKNSGAKNEVKDTKNAPKSASKDFSKILNQKISKDKTAPKE--NPS--ALKATPKD----AKQNAKELEKNPTPHHQHAQHSJM:HPSJM_04620  MPSPINPIHTNANA-----L--NSGAKNE--DAKNAPKSASKDFSKILNQKISKDKTAPKESLNHN--ALKATPKD----AKEDAKALEKTPTPNHQHAQ                  101       111       121       131       141       151       161       171       181       191                         |         |         |         |         |         |         |         |         |         |         HB8:HPB8_646      NPAKDQQAPTLKDWLNHKKT--TVSHEAQHETHEANETNPKTPNETLNKNEKKPNGVTSNAHQTNLASKNPITP-NHANNAIKNPTTPTHSAKEPKTLKDHHPA:HPAG1_0888   NLAKDQQAPTLKDWLNHKKT--TASHEAQHEIHENHETNPKTPNETLNKNEKKSNGVTSNAHQANLTNKNPLTPTNHANHAIKTPTTPTHNAKEPKTLKDHP12:HPP12_0904   NPAKDQQAPTLKDLLNRPKTHPTAPHETQHE---NHETNPKTPNETLNKNEKKPNGVTSNAHQANLTNKNPLTPTNH---AIKNPTTPTHNAKEPKTLKDHG27:HPG27_857    NPAKDQQAPTLKDLLNH-KT--TAPHEAQHENHE-HETNPKTPNETLNKNEKEPNGVTSNDHQANLTNKNPLTPTNH---AIKNPTAPTHNAKEPKTLKDHB38:HELPY_0890   NPAKDQQAPTLKDWLNHKKT--TASHEAQHE--KNHETNPKTPNETLNKNEKKPNGVTSNAHQTNLPNKNPITPTNHANNAIKNPTAPTHNAKESKTLKDH266:HP0906       NPAKDQQAPTLKDWLNHKKT--TTPHETQHETHEANETNPKTPNETLNKNEKKPNGVTSSVHQTNLTNKNPITPTNHANNAIKNPTAPTDTKKEPKTLKDHF32:HPF32_0448   NLAKDQQAPTLKDLLNHKKT--TASHEAQHEIHKNHETNPKTPNETLNKNEKKPNGVISSAHQANLTNKNPLAPTNHANNAIKNPTAPTHNAKDPKTLKDH51:KHP_0846      NLAKDQQAPTLKDLLNHKKT--TASHEAQHEIHKNHETNPKTPNETLNKNEKKPNGVTSSAHQANLTNKNPLTPTNHANNAIKNPTAPTHNAKESKTLKDH52:HPKB_0873     NLAKDQQAPTLKDLLNHKKT--TASHEAQHEMHENHETNPKTPNETSNKNEKKSNGVVSNAHQANLTNKNPLTPTNHANNSIKNPTAPTDTKKDPKTLKDHF57:HPF57_0916   NLAKDQQAPTLKDLLNHKKT--TASHEAQHEIHKNHETNPKTPNKTLNKNEKKPNEVASSAHQANLTNKNPLTSTNHANNAIKNPAAPTHNAKEPKTLKDHF30:HPF30_0433   NLAKDQQAPTLKDLLNHKKT--TASHEAQHEIHKNHETNPKTPNETLNKNEKKPNGVTSNAHQASLTSKNPLTPTNHAHNAIKNPTAPTHNAKDPKTLKDHF16:HPF16_0887   NLAKDQQAPTLKDLLNHKKT--TASHETQHETHKNHETNPKTPNETLNKNEKKPNGVASGAHQANLTHKNPLTPTNH---AIKNPTAPTHNAKESKTLKDHSJM:HPSJM_04620  NLAKNQQAPTLKDWLNHPKTHPTAKHEAQHETHEANETNPKTPNETLSKNEKKPNEVTSNAHQINLPNKNPITP-NHAN---KTPTTPTHSAKEPKTLKD                  201       211       221       231       241       251       261       271       281       291                         |         |         |         |         |         |         |         |         |         |         HB8:HPB8_646      IQTLSQKHDLNASNIQATTTPENK-NPLNASDHLALKTTQTPTNHTLAKNDAKNTANLSSVLQSLEKKEPHNKEHANPQNNEKKTPPLKEALPMNAIKRDHHPA:HPAG1_0888   IQTLSQKHDLNANNIQATTTPENK-TPLNAGDQFALKTTQTPTNHTLAKNDAKNTANLSSVLQSLEKKESHNKEHANLSNNEKKTPPLKEALQMNAIKRDHP12:HPP12_0904   IQTLSQKHDLNASNIQATTTPENK-TPLNASDQLALKTTQAPINNTLAKNDAKNTANLSSVLQSLEKKESHNKERTTPPNNEKKTPPLREALQMNAIKRDHG27:HPG27_857    IQTLSQKHDLNANNIQAATIPENK-TPLNASDHLALKTTQTPINHTLAKNDAKNTANLSSVLQSLEKKESHNKEHANPPNNEKKTPPLKEALQMNAIKRDHB38:HELPY_0890   IQTLSQKHDLNASNIQAATTPENK-TPLNASDQLALKTTQTPTNHTLAKNDAKNTANLSSVLQSLEKKESHNKEHANPQNNEKKTPPLKEALQMNAIKRDH266:HP0906       IQTLSQKHDLNASNIQAATTPENK-NPLNASDQLALKTTQTPTNHTLAKNDAKNTANLSSVLQSLEKKEPQNKEHANPLNNEKKTPPLKEALEMNAIKRDHF32:HPF32_0448   IQTLSQKHDLNASNIQVGTPLEKKETPLNASDQLALKTTQTPINHTLAKNDAKNTANLSSVLQSLEKKESHNKERATPPSNEKKTPPLREALQMNAIKRDH51:KHP_0846      IQTLSQKHDLNASNIQVGTPLEKKETPLNANDQLALKTTQTSINHTLAKNDTKNTANLSSVLQSLEKKESQNKERTTPPSNEKKTPPLKEALQMNAIKRDH52:HPKB_0873     IHALSQKHDLNASNIQVGTPLEKKETPLNASDQLALKTTQTSINHTLAKNGTKNTANLSSVLQSLEKKESHNKERTTLPHNEKKTPPLREALQMNAIKRDHF57:HPF57_0916   IQTLSQKHDLNASNIQVGTPLEKKETPLNASDQLALKTTQTSINHTLAKNDAKNTANLSSVLQSLEKKESHNKDHATPPSNEKKTPPLREALQMNAIKRDHF30:HPF30_0433   IQTLSQKHDLNASNIQATAPLEKKETPLNASDQLALKTTQTSINHTLAKNDSKNTANLSSVLQSLEKKESQNKEHATPPSNEKKTPPLREALQMNAIKRDHF16:HPF16_0887   IQTLSQKHDLNASNIQVGTPLEKKETPLKASDQLALKTTQTPINHTLAKNGAKNTANLSSVLQSLEKKESHNKKHTTPPSNEKKTPPLREALQMNAIKRDHSJM:HPSJM_04620  IQTLSQKHDLNASNIQATAPLEKKETPLSASDQLALKTTQTPTSHTLAKNDAKNTANLSSVLQSLEKKESQNKEHANPPNNEKKTPPLKEALQMNAIKRD                  301       311       321       331       341       351       361       371       381       391                         |         |         |         |         |         |         |         |         |         |         HB8:HPB8_646      KTLSKKKSEKTQ--TKAQTTAPSIATENAPKIPLKTPPLMPLTGANPPPNNNAPTLLEKEETTKEASDNKEKTKESSNSAQSAQNAQSSDKTSENKSAAPHHPA:HPAG1_0888   KTLSKKKSEKTP--TKAQTTAPSITPENAPKIPLKTPPLMPLIGANPPLNNNAPTPLEKEETTKEISDNKEKAKETNNSAQNAQNAQASDKTNENKSIAPHP12:HPP12_0904   KTLSKKKSKKTP--TKAQTTAPSIAPENAPKIPLKTPPLMPLIGANPP-NDNPPTPLEKEETTKEASDNKEKTKEANNSAQSAQNAQASDKTSENKSVTPHG27:HPG27_857    KTLSKKKSEKTP--TKTQTTAPSIAPENAPKIPLKTPPLMPLIGANPP-NDNPPTPLEKEETTKEASDNKEKTKESSNSAQNAQNAQSSDKTSENKSVTPHB38:HELPY_0890   KTLSKKKPEKTQ--TKTQTTAPSIALENAPKIPLKTPPLMPLIGANPP-NDNPPTLLEKEETTKEVSDNKEKAKETSSSAQSAQNTQASDKTSDNKSIAPH266:HP0906       KTLSKKKSEKTPIHAKTQTTAPSATPENAPKIPLKTPPLMPLIGANPPPNDNIPTPLEKEEKAKEASDNKEKTKETSNSAQNAQNTQASDKTSDNKSTAPHF32:HPF32_0448   KTLSKKKPEKTP--TKTQTTAPSTTPENAPKIPLKTPPLMPLIGANPP-NDNAPTLLEKEEKAKEVSENKEKTKESTNSAQSAQNAQASDKTSENKSTAPH51:KHP_0846      KTLSKKKSEKTP--TKTQTTAPSAMPENAPKIPLKTPPLMPLIGANPP-NDNPPTLLEKEETTKEVSDNKEKTKETSNSAQSAQNAQASDKTSENKSAAPH52:HPKB_0873     KTLSKKKPEKTP--TKTQTTAQAATPENAPKIPLKTPPLMPLIGANPP-NDNAPTPLEKEEKAKEASDNKEKTKESTNSAQSAQNAQASDKTSENKSAAPHF57:HPF57_0916   KTLSKKKPEKTPIHAKTQTTAQAATPENAPKIPLKTPPLMPLIGANPP-NDNAPTPLEKEEKTKEVSENKEKTKESTNSAQNAQNAQASDKTSENKSVTSHF30:HPF30_0433   KTLSKKKSEKTPIHAKAQTTAQAATPENAPKIPLKTPPLMPLIGANPP-NDNAPTPLEKEEKTKEASDNKEKTKESTNSAQNAQNAQASDKTSENKSAAPHF16:HPF16_0887   KTLSKKKPEKTPIHAKTQTTAQAATPENAPKIPLKTPPLMPLIGANPP-NDNAPTPLEKEEKTKEVSENKEKTKESTNSTQNAQNAQASDKTSENKSTTPHSJM:HPSJM_04620  KTLSKKKPEKTPIHAKTQTTAPSATPENAPKLALKTPPLMPLIGANPP-NDNIPTPLEKEEKTKEVSDNKEKTKESSNSAQSAQNTQASDKTSDNKSIAP                  401       411       421       431       441       451       461       471       481       491                         |         |         |         |         |         |         |         |         |         |         HB8:HPB8_646      KETIKHFTQQLKQEIQEYKPPMSRISMDLFPKELGKVEVVIQKVGKNLKVSVISHNNSLQTFLDNQQDLKNSLNALGFEGVDLSFSQDSSKEQ--PKEQLHHPA:HPAG1_0888   KETIKHFTQQLKQEIQEYKPPMSRISMDLFPKELGKVEVIIQKVGKNLKVSVISHNNSLQTFLDNQQDLKNSLNALGFEGVDLSFSQDSSKEQ--PKEQLHP12:HPP12_0904   KETIKHFTQQLKQEIQEYKPPMSRISMDLFPKELGKVEVIIQKVGKNLKVSVISHNNSLQTFLDNQQDLKNNLNALGFEGVDLSFSQDSSKEQ--PKEQLHG27:HPG27_857    KETIKHFTQQLKQEIQEYKPPMSRISMDLFPKELGKVEVIIQKVGKNLKVSVISHNNSLQTFLDNQQDLKNSLNALGFEGVDLSFSQDSSKEQ--PKEQLHB38:HELPY_0890   KETIKHFTQQLKQEIQEYKPPMSKISMDLFPKELGKVEITIQKMGKNLKVSVISHNNSLQTFLDNQQDLKNSLNALGFEGVDLSFSQDSSKEQ--EKEPFH266:HP0906       KETIKHFTQQLKQEIQEYKPPMSRISMDLFPKELGKVEVIIQKVGKNLKVSVISHNNSLQTFLDNQQDLKNSLNALGFEGVDLSFSQDSSKEQQAPKDQPHF32:HPF32_0448   KETIKHFTQQLKQEIQEYKPPMSRISMDLFPKELGKVEVTIQKVGKNLKVSVISHNNSLQTFLDNQQDLKNSLNALGFEGVDLSFSQDSSKEQ--PKEQLH51:KHP_0846      KETIKHFTQQLKQEIQEYKPPMSRISMDLFPKELGKVEVTIQKVGKNLKVSVISHNNSLQTFLDNQQDLKNSLNALGFEGVDLSFSQDSSKEQ--PKEQLH52:HPKB_0873     KETIKHFTQQLKQEIQEYKPPMSRISMDLFPKELGKVEVTIQKVGKNLKVSVISHNNSLQTFLDNQQDLKNSLNALGFEGVDLSFSQDSSKEQ--EKEQLHF57:HPF57_0916   KETIKHFTQQLKQEIQEYKPPMSRISMDLFPKELGKVEVVIQKVGKNLKVSVISHNNSLQTFLDNQQDLKNSLNALGFEGVDLSFSQDSSKEQ--PKEPFHF30:HPF30_0433   KETIKHFTQQLKQEIQEYKPPMSRISMDLFPKELGKVEVTIQKVGKNLKVSVISHNNSLQTFLDNQQDLKNSLNALGFEGVDLSFSQDSSKEQ--EKESLHF16:HPF16_0887   KETIKHFTQQLKQEIQEYKPPMSRISMDLFPKELGKVEVIIQKVGKNLKVSVISHNNSLQTFLDNQQDLKNSLNALGFEGVDLSFSQDSSKEQ--EKEPLHSJM:HPSJM_04620  KETIKHFTQQLKQEIQEYKPPMSKISMDLFPKELGKVEVTIQKVGKNLKVSVISHNNSLQTFLDNQQDLKNSLNALGFEGVDLSFSQDSSKEQ--PKEQL                  501       511       521       531                  |         |         |         |HB8:HPB8_646      RELFKEQESSPLKENALKSYQENTDHENQETSMQITLYAHHPA:HPAG1_0888   RELFKEQESSPLKENALKSYQENTDHENQETSMQITLYAHP12:HPP12_0904   RELFKEQESSPLKENALKSYQENTDNEHKETSMQITLYAHG27:HPG27_857    KEPFKEQELTPLKENALKSYQENTDHENKETSMQITLYAHB38:HELPY_0890   KEPFKEQELTPLKENALKSYQENTDHENKETSMQITLYAH266:HP0906       KEPFKEQELTPLKENALKSYQENTDNENQETSMQITLYAHF32:HPF32_0448   REPFKEQESTPLKENALKSYQENTDNENKETSMQITLYAH51:KHP_0846      REPFKEQESTPLKENALKSYQENTDNENKETSMQITLYAH52:HPKB_0873     REPFKEQESTPLKENALKSYQENTDNENKETSMQITLYAHF57:HPF57_0916   KEPFKEQELTPLKENALKSYQENTDNENKETSMQITLYAHF30:HPF30_0433   REPFKEQELTPLKENALKSYQENTDNENKETSMQITLYAHF16:HPF16_0887   REPFKEQESTPLKENALKSYQENTDNENKETSMQITLYAHSJM:HPSJM_04620  RELFKEQESSPLKENALKSYQENTNHENQETSMQITLYA
