# Supplementary material for: Evolution in an oncogenic bacterial species with extreme genome plasticity: Helicobacter pylori East Asian genomes
Source: BMC Microbiol. 2011 May 16;11:104. doi: 10.1186/1471-2180-11-104 (PMC3120642; doi:10.1186/1471-2180-11-104)
Supplement: Additional file 6 — Multiple sequence alignments of diverged genes. [file 1471-2180-11-104-S6.ZIP › Diverged_genes_multiple_seuence_alignments/HP0922_vacA4.mfa.rtf]

                  1         11        21        31        41        51        61        71        81        91                          |         |         |         |         |         |         |         |         |         |         HB8:HPB8_627      MAFKKARLISKLISKGSFKLSKISKKIFTLNQILKREKPLKRHKKALKPIKKLSNRNKSFLKASVLLIGALGGLSHLRANECRYWSWSSWSYQDNIESGSHSJM:HPSJM_04695  MAFKKARLISRFISKGSFKLNKISKKIFTLNQILKREKPLKCHKKT-KSIKKLSNRNKSFLKASILLIGALGGLSHLRANECRYWSWSSWGYQDNIESGPHP12:HPP12_0919   VAFKKAKLISNLISKGSFKLNKISKKIFKLNQILKCEKPLKRHKKT-KSIKKPFNKNKSFLKASILLIGALGGLSHLRANECRYWSWSSWSYQDNIESGPHHPA:HPAG1_0903   MAFKKARLISRFISKGSFKLSKISKKIFTLNQILKCEKPLKCHKKT-KSIKKLSNRNKSFLKASILLIGALGGLSHLRANECRYWSWSSWGYQDNIESGPH266:HP0922       MAFKKARLISKFISKGSFKLNKISKKIFTLNQILKCEKPLKRHKKALKPIKKLSNRNKSFLKASVLLIGALGGLSHLRANECRYWSWSSWSYQDNIESGPHG27:HPG27_871    VAFKKARLISRFISKGSFKLNKISKKIFKLNQILKREKPLKRHKKALKPIKKLSDRNKSFLKVSVLLIGALGGLSHLRANECRYWSWSSWSYQDNIESGPHB38:mHELPY_0906  -------------------LNKISKKIFTLNQILKREKPLKRHKKALKPIKKLSDRNKSFLKASVLLIGALGGLSHLRANECRYWSWPSWSYQDNIESGPHF32:HPF32_0434   MAFKKAGLISKFISKGSFKLNKISKKIFKLNLILKHEKPLS-HKKT-KSVKKPFNQNKSFLKASVLLIGALGGLSHLRASECRYWSWSSWSYHDNIESGSHF57:HPF57_0931   MAFEKARLISKFILKGSFKLNKISKKIFKLNLILKREKPLS-HKKT-KSVKKPFNKNRSFLKASVLLIGALGGLSHLRASECRYWSWSSWGYHDNIESGSH51:KHP_0860      MAFKKVGLISKFISKGSFKLNKISKKIFKLNQILKRENPLS-HKKT-KSIKKPFNKSKSFLKASVLLIGALGGLSHLRASECRYWSWSSWSYHDNIESGSHF16:HPF16_0901   MAFKKAGLISKFILKGSFKLNKISKKIFKLNLILKREKPLS-HKKT-KSIKKPFNKNKSFLKASVLLIGALGGLSHLRASECRYWSWSSWNYQDNIESGPH52:HPKB_0890     MAFKKARLISKFILKGSFKLNKISKKIFKLNQILKYEKPLS-HKKT-KSVKKPFNKNKSFLKASVLLIGALGGLSHLRASECRYWSWSSWSYHDNIESGPHF30:HPF30_0419   MAFKKVGLISKFILKGSF-LNKISKKIFKLNPILKREKPLS-HKKT-KSVKKPFNKNKSFLKASVLLIGALGGLSHLRASECRYWSWSSWGYHDNIESGS                  101       111       121       131       141       151       161       171       181       191                         |         |         |         |         |         |         |         |         |         |         HB8:HPB8_627      NSPTHNSYCLFSSTQGSGTYYLNTLTTYSAGGASFTQKFNNGTLNVGGNIRFGGTGINGGDVGYITGTYDAQTINFNSSHLTTGNSYADGGGATLNFNATHSJM:HPSJM_04695  NSPTHNSYCLFSSAQGSGTYYLNTLTTYSAGGASFTQKFNGGTLDIGGNIRFGGTGINGGDVGYITGTYDAQTINFDSSHLTTGNSYSDGGGATLNFNAAHP12:HPP12_0919   NSPTHNSYCLFSSTQGSGTYYLNTLTTYSAGGASFTQKFNGGTLNVGGNIRFGGTGINGGDVGYITGAYDAQTINFNSSHLTTGNSYSDGGGATLNFNATHHPA:HPAG1_0903   NSPTHNSYCLFSSTQGSGTYYLNTLTTYSAGGASFTQKFNGGTLNVGGNIRFGGTGINGGDVGYITGTYDAQTINFNSSHLTTGNSYADGGGATLNFNAAH266:HP0922       NSPTHNSYCLFSSTQGSGTYYLNTLTTYSAGGASFTQKFNNGTLNVGENIRFGGTGINGGDVGYITGTYDAQTINFNSSHLTTGNSYADGGGATLNFNAAHG27:HPG27_871    NSPTHNSYCLFSSTQGSGTYYLNTLTTYSAGGASFTQKFNGGTLNVGGNIRFGGAGINGGDVGYITGTYDAQTINFNSSHLTTGNSYADGGGATLNFNATHB38:mHELPY_0906  NSPTHNSYCLFSSTQGSGTYYLNTLTTYSAGGASFTQKFNGGTLNVGGNIRFGGTGINGGDVGYITGTYDAANI-YLTSHLTTGNSYADGGGATLNFNATHF32:HPF32_0434   NSPTHNSYCLFNSAQGSGTYYLNTLTTYSAGGASFTQKFNNGTLDVGGNIRFGGTGVNGGDVGYITGTYDSQTINFNSSRITTGNSFSTGGGTTLNFNATHF57:HPF57_0931   NSPTHNSYCLFNSAQGSGTYYLNTLTTYSAGGASFTQKFNNGTLNVGGNIRFGGTGVNGGNLGYITGTYDAQTINFNSSRITTGNSFSTGGGATLNFNATH51:KHP_0860      NSPTHNSYCLFNSAQGSGTYYLNTLTTYSAGGASFTQKFNNGTLNVGGNIRFGGTGVNGGNLGYITGTYDAQTINFNSSRITTGNSYSTGGGATLNFNATHF16:HPF16_0901   NSPTHNSYCLFSSTQGSGTYYLNTLTTYSAGGASFTQKFNNGTLNVGGNIRFGGTGVNGGNLGYITGTYDAQTINFNSSRITTGNSFSTGGGATLNFNATH52:HPKB_0890     NSPTHNSYCLFSSAQGSGTYYLNTLTTYSAGGASFTQKFNGGTLNVGGNIRFGGTGINGGNLGYITGTYDAQTINFNSSRITTGNSFSTGGGATLNFNATHF30:HPF30_0419   NSPTHNSYCLFSSAQGSGTYYLNTLTTYSPGGASFTQKFNNGTLNVGGNIRFGGTGVNGGDVGYITGTYDAQTINFNSSRITTGNSFSTGGGATLNFNAT                  201       211       221       231       241       251       261       271       281       291                         |         |         |         |         |         |         |         |         |         |         HB8:HPB8_627      NHITINQASFDNSDAGTQKSYMNFKGSNIKVSGSSFKDDTDGGFSFSGNSNNSTISFNQTSFNQGTYHFSNSATLSFDNSS-------------------HSJM:HPSJM_04695  NNITINQASFDNSDAGTQHSYMNFKGSNIKVSGSSFKDDTDGGFSFSGNSNNSTISFNQTNFNQGTYHFSNSASSSFDNSS-------------------HP12:HPP12_0919   NNITINQASLDNSDAGAQKSYMNFKGSNINVSGSSFTDDTDGGFSFSGNNNHSAISFNQTNFNQGT----------------------------------HHPA:HPAG1_0903   NNITINQASFDNSDAGAQKSYMNFKGSNIKVSGSSFKDDTDGGFSFSGNSNNSTISFNQTNFNQGTYNFSNSANLSFTNSAFNQGTYNFNSAQSVFENSSH266:HP0922       NNITINQASFDNSHAGTQKSYMNFKGSNIKVSGSSFTDDTDGGFSFSGNSNNSTISFNQTSFNQGTYHFSNSATLSFNHSA-------------------HG27:HPG27_871    NNLTINQASFDNSDAGTQHSYMNFKGSNIKVSGSRFKDDTNGGFNFSGNNNNSAISFNQTNFNQGT----------------------------------HB38:mHELPY_0906  NNLTINQASFDNNDAGTQKSYMNFKGSNIKVSGSSFKDDTDGGFSFSGNNNNSTISFNQTNFNQGTYHFSNSASSSFDNSS-------------------HF32:HPF32_0434   NHITINQASFDNGDAGTQHSYMNFSGSNINVSDSSFTDDTNGGFSFSGNGTNSNLSFDKTNFNQGTYKFTNSANLNFNNSA-------------------HF57:HPF57_0931   NHITINQASFDNSDAGTQRSYMNFSGSNINVISSSFTDDTNGGFSFSANGANSNLSFNKTNFNQGTYKFTNSANLNFNNSAFNQGTYNFNSAQSVFENSNH51:KHP_0860      NHITIDQASFDNSDAGTQHSYMNFSGSSINVSASSFTDDTNGGFSFSGNGANSNLSFDKTNFNQGTYKFTNSANLNFNNSA-------------------HF16:HPF16_0901   NHITINQASFDNSDAGTQRSYMNFSGSNINVSASIFTDGTNGGFSFSGNGTNSNLSFNKTNFNQGTYKFTNSANLNFNNSA-------------------H52:HPKB_0890     NHITINQASFDNSDAGTQHSYMNFSGSNINVSGSSFTDDTNGGFSFSGNGTNSNLSFDKTNFNQGTYKFTNSANLNFN----------------------HF30:HPF30_0419   NHNTINQASFDNGDAGTQHSYMNFSGSNINVSDSSFTDDTNGGFSFSGNNNNSTISFNQTKFNQGTYKFTNSANLSFNNSA-------------------                  301       311       321       331       341       351       361       371       381       391                         |         |         |         |         |         |         |         |         |         |         HB8:HPB8_627      FNQGTYHFNSAQSVFENSNFNQGTYNFNDNT--SFNNDTFNQGTYSFNTSKVSFSGANTLNSSSPFASLKGSVSFGSGAVFNLNQTLNSNQTYDILTTNGHSJM:HPSJM_04695  FNQGSYHFNSAQSTFENSNFNQGTYNFNDNT--SFNNDTFNQGTYNFNTSKVSFLGINTLNSSSPFASLKGSVSFNSGAIFNLNQTLNSNQTYDILTTNGHP12:HPP12_0919   -----YHFNSTQSTFENSNFNQGTYNFNDNT--SFNNDTFNQGTYSFNTSKVSFSGINTLNSSSPFASLKGSVSFGSDAVFNLNQTLNSNQTYDILTTNGHHPA:HPAG1_0903   FNQGTYNFNSAQSVFENSAFNQGTYNFNGNA--SFDNDIFNQGTYNFNTSKVSFSGANTLNSSSPFASLKGSVSFGSDAVFNLNQTLNSNQTYDILTTNGH266:HP0922       FNQGTYNFNSTQSAFNNSAFNQGTYHFNGNA--SFDNDTFNQGTYSFNTSKVSFSGINTLNSSSPFASLKGSVSFGSDAIFNLNQTLN-NQTYDILTTNGHG27:HPG27_871    -----YHFNSAQSTFENSNFNQGTYNFNGNA--SFDNDTFNQGTYNFNTSKVSFSGINTLNSSSPFASLKGSVSFNSNAVFNLNQTLNNNQTYDILTTNGHB38:mHELPY_0906  FNQGSYHFNSAQSTFENSNFNQGTYNFNDNT--SFNNDTFNQGTYSFNTSKVSFSGINTLNSSSPFASLKGSVSFNSGAVFNLNQTLNNNQTYDILTTNGHF32:HPF32_0434   FNQGSYHFNSAQSVFENSNFNQGTYNFNDNT--SFNNDTFNQGTYSFNTNKVSFSGINTLNSSSPFANLKGSVSFGSDAIFNLNQTLNTNQTYDILTTNGHF57:HPF57_0931   FNQGTYNFNSAQSVFENSNFNQGTYNFTDNA--SFNNDTFNQGTYNFNASKVSFSGANTLNSSSPFASLKGSVSFGSGAIFNLNQTLNSNQTYDILTTNKH51:KHP_0860      FNQGTYNFNSAQSTFNNSNFNQGTYHFTDNA--SFNNDTFNQGTYNFNASKVSFSGANTLNSSSPFASLKGSVSFGSGAIFNLNQTFNSNQTYDILTTNKHF16:HPF16_0901   FNQGTYNFNSAQSVFENSNFNQGTYSFTDNTGLNFNNDTFNQGTYNFNTSKVSFSGANTLNSSSPFASLKGSVSFGSGAIFNLNQTLNSNQTYDILTTNKH52:HPKB_0890     ----------------NSNFNQGTYSFTDNTGLNFNNDTFNQGTYNFNASKVSFSGANTLNSSSPFASLKGSVSFGSGAIFNLNQTLNSNQTYDILTTNKHF30:HPF30_0419   -------------------FNQGTYNFTDNA--SFNNDTFNQGTYNFNVSKVSFSGTNTLNSSSPFASLKGSVSFGSDAIFNLNQTLNTNQTYDILTTNK                  401       411       421       431       441       451       461       471       481       491                         |         |         |         |         |         |         |         |         |         |         HB8:HPB8_627      TIQYGVYQSYLWDLINYKGDKAISHVGVGNNTYDVTFDINGQDETLQETFNNQSIITQFLGDDLQAKAQKTYQQDLSNSQSALNNAASDSKIANSDTSYTHSJM:HPSJM_04695  AIQYGVYQSYLWDLINYKGDKAISHVEVGNNTYDVTFDINGQDETLQETFNNQSIITQFLGDDLQQQAQKTYQQDLSNSQSALNNAASDSKIANSDTDYTHP12:HPP12_0919   AIQYGVYQSYLWDLINYKGDKAISHVGVGNNTYDVTFDINGQDETLQETFNKQSIITQFLGDDLQQQAQKTYQEDVAHSQNALNNVTSDNKIANSDTDYTHHPA:HPAG1_0903   TIQYGVYQSYLWDLINYKGDKAISHVEVGNNTYDVTFDINGQDETLQETFNKQSIITQFLGDDLQQQAQKTYQQDLSNSQSALNNAASDSKVANNDTGYTH266:HP0922       AIQYGVYQSYLWDLINYKGDKAISHVEVGNNTYDVTFDINGQDETLQETFNKQSIITQFLGDDLQQQAQKTYQQDLSNSQSALNNAASDNKIANSDTDYTHG27:HPG27_871    AIQYGVYQSYLWDLINYKGDKAISHVGVGNNTYDVTFDINGQDETLQETFNKQSIITQFLGDDLQQQAQKTYQEDLTHSQNALNNVTSDNTIANNDTSYTHB38:mHELPY_0906  AIQYGVYQSYLWDLINYKGDKAISHVGVGNNTYDVTFNINGQDETLQETFNKQSIITQFLGDDLQQQAQQTYQEDLTHSQNALNNVTSDNTIANNDTSYTHF32:HPF32_0434   TIQYGVYQSYLWDLINYKGDKAISHVEVGSNTYDVTFDINGQDETLQETFNKQAITTQFLGDDLQAKAQATYQQDLSNSQTALNNATSDNKIASSDTGYTHF57:HPF57_0931   TIQYGVYQSYLWDLINYKGDKAISHVEVGSNTYDVTFDINGQDETLQETFNNQSITTQFLGDDLQAKAQATYQQDLSNSQSALNNATSDNKIASNDTGYTH51:KHP_0860      TIQYGVYQSYLWDLINYKDDKAISHVEVGSNTYDVTFDINGQDETLQETFNNQSITTQFLGDDLQAKAQATYQQDLSNSQTALNNATSDNKIASSDTGYTHF16:HPF16_0901   TIQYGVYQSYLWDLINYKGDKAISHVEVGSNTYDVTFDINGQDETLQETFSNQSITTQFLGDDLQAKAQAIYQQDLSNSQTALNNATSDNKIASNDTGYTH52:HPKB_0890     TIQYGVYQSYLWDLINYKGDKAISHVEVGSNTYDVTFDINGQDETLQETFSNQSITTQFLGDDLQAKAQATYQQDLSNSQSALNNATSDNKIASNDTSYTHF30:HPF30_0419   TIQYGVYQSYLWDLINYKGDKAISHVEVGSNTYDVTFDINGQDETLQETFNNQAITTQFLGDDLQAKAKATYQQDLSNSQTALNNATSDNKIASSDTSYT                  501       511       521       531       541       551       561       571       581       591                         |         |         |         |         |         |         |         |         |         |         HB8:HPB8_627      QSKNTTVAKDAQNLEHTNQQIAQDEQALQGDLDKLKQLANSPTGFNEQAFNQAQKQEQQDEQTLQNDENAFNTEQEG-----------------------HSJM:HPSJM_04695  NNKNTTIKEDTKNLEHTNQQIAQDEQALQGDLDKLKQLANSTTGFNEQAFNQAQSKEQQDEQTLQNDENAFNAEQDS-----------------------HP12:HPP12_0919   KSSNPTINKDAQNLEHTNQQIAQDEQALQQDLDKLKQLANSTTGFNEQAFNQAQKQEQQDEQTLQNEENAFNAEQEG-----------------------HHPA:HPAG1_0903   QSKNATVAKDAQGLENTNQKIQQDEQALEKDLAQIKQLANSTTGFNQQAFNQAQSTEQQDEQTLQNEENTFNIEQES-----------------------H266:HP0922       KNKNATIKKDAQGLENTNQQIAQDEQALQGDLDKLKQLANSPTGFSEQAFNQAQKQEQQDEQTLQNEEKTFNSEQEGLKQAIQQAQAQQQKQQQKQEQQQHG27:HPG27_871    QSKNATIAKDAQGLENTNQKIAQDEQALEKDLAQIKQLANSTTGFSEQAFTQAQKQEQQDEQTLQNEEKTFNSEQEGLKQAIQQAQA----QQQKQEQQQHB38:mHELPY_0906  QSKNTTVAKDAQGLENTNQKIQQDEQALEKDLAQIKQLANSPTGFSEQAFNQAQKQEQQDEQTLQNDENAFNTKQEGLKQAIQQAQA----QQQKQEQQQHF32:HPF32_0434   NNQNTTIKKDAQSLENTSQQIVKDQRALQQDLDNLKQLANSPTGFNQQAFKNTQSTEQQDLQTLQENENTFNSEQEG-----------------------HF57:HPF57_0931   NNQNTTIKKDAQNLENTDQTIQQDKQALEKDLANVKQLANAPTGFNQQAFNQAQSTEQQDLQTLQKNESTFSSEQEG-----------------------H51:KHP_0860      NNQNTTIKKDAQNLENTDQTIQQDKQALEKDLANVKQLANAPTGFNQQAFKNAQSTEQQDLQTLQGEEKAFSSEQEG-----------------------HF16:HPF16_0901   NNQNTTIKKDAQSLENTDQTIQQDEQALKGDLDKLQQLANATTGFNEQAFNQAQSTEQQDLQTLQENENTFNSEQEG-----------------------H52:HPKB_0890     NNQNTTIKKDAQSLENTSQQIAQDQKDLEQDLDNLKQLANAPTGFNQQAFKNAQSTEQQDLQTLQENEKTFSSEQEG-----------------------HF30:HPF30_0419   NNQNTTIKKDAQSLENTSQQIAQDKQALQGDLDKLQQLANAPTGFNQQAFKNAQSTEQQDLQTLQGEEKAFSSEQEG-----------------------                  601       611       621       631       641       651       661       671       681       691                         |         |         |         |         |         |         |         |         |         |         HB8:HPB8_627      ----------------------------------------------------------------------------------------------------HSJM:HPSJM_04695  ----------------------------------------------------------------------------------------------------HP12:HPP12_0919   ----------------------------------------------------------------------------------------------------HHPA:HPAG1_0903   ----------------------------------------------------------------------------------------------------H266:HP0922       AQQTYQEDLTHSQSALNDVASDNTIASNDTNYTNNQNTAIKEDAQGLENTNQQIAQDEQALQGDLDKLKQLANSPTGFSEQAFNQAQKQEQQDEQTLQNEHG27:HPG27_871    AQQTYQQDLSNSQSALNNAASDSKIANSDTDYTKNKNTAIATDAQNLENTNQQIAQDEQALEKDLAQIKQLANSTTGFSEQAFNQAQSTEQQDEQTLQNEHB38:mHELPY_0906  AQQTYQEDLTHSQNALNNVTSDNTIANNDTSYTQSKNTTVAKDAQGLENTNQKIQQDEQALEKDLAQIKQLANSPTGFSEQAFNQAQKQEQQDEQTLQNDHF32:HPF32_0434   ----------------------------------------------------------------------------------------------------HF57:HPF57_0931   ----------------------------------------------------------------------------------------------------H51:KHP_0860      ----------------------------------------------------------------------------------------------------HF16:HPF16_0901   ----------------------------------------------------------------------------------------------------H52:HPKB_0890     ----------------------------------------------------------------------------------------------------HF30:HPF30_0419   ----------------------------------------------------------------------------------------------------                  701       711       721       731       741       751       761       771       781       791                         |         |         |         |         |         |         |         |         |         |         HB8:HPB8_627      ----------LKQAIANAK---PT---------SPTPSPTPTPTKHTAQNTPPNKVPPTPTPPTQNLPTTNVWDGVYNLQNQTYSQKGIYYIDPNLSGQSHSJM:HPSJM_04695  ----------LNKAIANAKHANPTPNPTPSPTPNPTPSPTPTPTKHTVQNTPPNKVP----PPTQNLPTTNVWNGVYNLQNQTYSNKGIYYIDPNLSGQSHP12:HPP12_0919   ----------LKQAI--AK---PT---------SPTPSPTPTPTKHTAQNTPPNKVP----PPAQNLPTTNVWNGVYNLQNQTYSNKGVYYIDPNLSGQSHHPA:HPAG1_0903   ----------LDKAIANAK---HT---------SPTPSPTPTPTKHTAQNTPPNKVSPTPTPPTQNLPTTNVWNGVYWLQNQTYSKQGVYYIDPNLSGQSH266:HP0922       EKTFNSEQERLKQAIANAK---PT---------SPTPSHAPTPTKHTAPNTPPNKVP--PTPPTQNPPAESVWSGVYWLQNKTYSNKGIYYIDPNLSGQSHG27:HPG27_871    EKTFNAEQEGLKQALANAK---PT---------SPTPSPTPTPTKHTAPNTPPNKVS--PTPPTQNLPTTNVWNGVYNLQNQTYSNKGIYYIDPNLSGQSHB38:mHELPY_0906  EKTFNAEQEGLKQAIANAK---PA---------SPTPSPTPTPTKHTVQNTPPSQVP--PT-PTQNPPAESVWSGVYWLQNKTYSNKGIYYIDPNLSGQSHF32:HPF32_0434   ----------LKQAIANAN---PA---------SPTPSPTPTPTKHTAPNTPPNKVP--PTPPTQNLPTTNVWNGVYNLQNQTYSKQGVYYIDPNLSGQSHF57:HPF57_0931   ----------LKQAIANAK---PT---------SPTPSPTPTPTKHTVPNTPPNKVP--PTPPTQNLPTTNVWSGVYNLQNQTYSQKGVYYIDPNLSGQSH51:KHP_0860      ----------LKQAIANAK---PA---------SPTPSPTPTPTKHTVPNTPPNKVP--PTPPTQNLPTTNVWSGVYWLQNQTYSKQGVYYIDPNLSGQSHF16:HPF16_0901   ----------LKQAIANAK---PT-------------SPTPTPTKHTAPNTPPNKVP--PTPPTQNLPTTNVWNGVYNLQNQTYSQKGIYYIDPNLSGQSH52:HPKB_0890     ----------LEKAIANTK---PA-------------NPTPTPTKHTAPNTPPNKVP--PTPPTQNLPTTNVWSGVYWLQNQTYSKQGIYYIDPNLSGQSHF30:HPF30_0419   ----------LEKAIANAK---PT---------SPTPSPTPTPTKHTAPNTPPNKVP--PTPPTQNLPTTNVWNGVYNLQNQTYSQKGIYYIDPNLSGQN                  801       811       821       831       841       851       861       871       881       891                         |         |         |         |         |         |         |         |         |         |         HB8:HPB8_627      GQSANTLSTYTANLFGRSFGVNIQNGTLIIGNNTESVNDNGLIWIGHGGFGYITGTFNAANIYLTNNFKTGEGVSNSDGGGANITFKASDNITMDGLNYNHSJM:HPSJM_04695  GQSGNTLSTYTANLFGRSFGVNIQNGALIIGNNTESVNDNGLIWIGHGGFGYITGTFNAANIYLTNNFKTGGGVSNSDGGGANITFKASDNITMDGLNYNHP12:HPP12_0919   GQSGNTLSTYTANLFGRSFGVNIQNGTLIIGNNTESVNDNGLIWIGHGGFGYITGTFSAANIYLTNNFKTGEGVSNSDGGGANITFKASDNITMDGLNYNHHPA:HPAG1_0903   GQSANTLSTYTANLFGRSFGVNIQNGTLIIGNNTESVNDNGLIWIGHGGFGYITGTFSAANIYLTNNFKTGEGVSNSDGGGANITFKASDNITMDGLNYNH266:HP0922       GQSGNTLSTYTANLFGRSFSVNIQNGTLIIGNNTESVNSNGLIWIGHGGFGYITGTFSAANIYLTNNFKTGEGVSNSDGGGANITFKASDNITMDGLNYNHG27:HPG27_871    GQSGNTLSTYTANLFGRSFSVNIQNGTLIIGNNTESVNDNGLIWIGHGGFGYITGTFNAANIYLTNNFKTGEGVSNSDGGGANITFKASDNITMDGLNYNHB38:mHELPY_0906  GQSGNTLSTYTANLFGRSFGVNIQNGTLIIGNNTESVNDNGLIWIGHGGFGYITGTFSAANIYLTNNFKTGEGVSNSDGGGANITFKASDNITMDGLNYNHF32:HPF32_0434   GQSGNTLSTYTANLLGRSFGMNIQNGTLIIGNNTESVNDNGLIWIGHGGFGYIIGTFNAANIYLTNNFKTGEGVSNSDGGGANITFKASDNITMDGLDYNHF57:HPF57_0931   GQSGNTLSTYTANLLGRSFSVNANNGTLIIGNNTESVNDNGLIWIGHGGFGYITGTFNAANIYLTNNFKTGEGVSGSDGGGANITFKASDNITMDGLNYNH51:KHP_0860      GQSANTLSTYTANLLGRSFGVNIQNGTLIIGNNTESVNDNGLIWIGHGGFGYIIGTFNAANIYLTNNFKTGEGVSGSDGGGANITFKASDNITMDGLNYNHF16:HPF16_0901   GQSGNTLSTYTANLLGRSFGVNIQNGTLIIGNNTESVNDNGLIWIGHGGFGYITGTFNAANIYLTNNFKTGEGVSGSDGGGANITFKASDNITMDGLNYNH52:HPKB_0890     GQSGNTLNTYTANLLGRSFGVNIQNGTLIIGNNTESVNDNGLIWIGHGGFGYITGTFNAANIYLTNNFKTGEGVSGSDGGGANITFKASDNITMDGLNYNHF30:HPF30_0419   GQSANTLSTYTANLFGRSFGVNIQNGTLIIGNNTESVNDNGLIWIGHGGFGYITGTFNAANIYLTNNFKTGEGVSGSDGGGANITFKASDNITMDGLNYN                  901       911       921       931       941       951       961       971       981       991                         |         |         |         |         |         |         |         |         |         |         HB8:HPB8_627      DAETVTKMIQTGTSQHSYAAFDALNNISVTNSSFSDMTWGKFSFSAKNISFSNASFSGFTNPGGSSTISANAANSLSFVNSRLNGGAIYNLQANSLIFNNHSJM:HPSJM_04695  DAETVTKMIQTGASQHSYATFDALNNISVTNSSFGDMTWGKFSFSAKNISFSNASFSGFTNPGGSSTISANASNSLSFINSRLNGGAIYNLQANSLIFNNHP12:HPP12_0919   DAETVTKMIQTGASQHSYAAFDATNNISVTNSSFSDMTWGKFSFSAKNISFSNASFSGFTNPGGSSTISANAANSLSFVNSRLNGGAIYNLQANSLIFNNHHPA:HPAG1_0903   DAETVTKMIQTGASQHSYAAFDALNNISVTNSSFSDMTWGKFSFSAKNISFSNASFSGFTNPGGSSVISANASNSLSFINSRLNGGAVYNLWANSLIFNNH266:HP0922       DAETVTKMIQTGASQHSYATFDALNNISVTNSSFSDMTWGKFSFSAKNISFSNASFSGFTNPGGSSVISANATNSLSFINSRLNGGAVYNLQANSLIFNNHG27:HPG27_871    DAETVTKMIQTRASQHSYATFDALNNISVTNSSFSDMTWGKFSFSAKNISFSNASFSGFTNPGGSSVISANAANSLSFINSRLNGGAIYNLQANSLIFNNHB38:mHELPY_0906  DAETVTKMIQTGASQHSYATFDALNNISVTNSSFSDMTWGKFSFSAKNISFSNASFSGFTNPGGSSTISANASNSLSFVNSRLNGGAIYNLWANSLIFNNHF32:HPF32_0434   DAETVTKMIQTGASQHSYAAFDATNNISVTNSSFSDMTWGEFSFSAKNISFSNASFSGFTNPGGSSVISANAANSLSFNNSRLNGGAVYNLWANSLIFNNHF57:HPF57_0931   DAETVTKMIQTGASQHSYAAFDATNNISVTNSSFSDMTWGKFSFNAKNISFSNASFSGFTNPGGSSVISANAANSLSFNNSRLNGGAVYNLWAKSLIFNNH51:KHP_0860      DAETVTKMIQTGASQHSYAAFDATNNISVTNSSFSDMTWGKFSFNAKNISFSNALFSGFTNPGGSSVISANAANSLSFNNSRLNGGAVYNLWANSLIFNNHF16:HPF16_0901   DAETVTKMIQTGASQHSYAAFDATNNISVTNSSFSDMTWGKFSFNAKNISFSNALFSGFTNPGGSSVISANAANSLSFNNSRLNGGAAYNLWANSLIFNNH52:HPKB_0890     DAETVTKMIQTEASQHSYAAFDATNNISVTNSSFSDMTWGKFSFNAKNISFSNASFSGFTNPGGSSVISANAANSLSFNNSRLNGGAIYNLQANSLIFNNHF30:HPF30_0419   DAETVTKMIQTGASQHSYAAFDATNNISVTNSSFSDMTWGKFSFSAKNISFSNASFSGFTNPGGSSVISANAANSLSFNNSRLNGGAVYNLWANSLIFNN                  1001      1011      1021      1031      1041      1051      1061      1071      1081      1091                        |         |         |         |         |         |         |         |         |         |         HB8:HPB8_627      TQAVFNVLYSRGTSNFNATTQLLGNTNFTLSSQSLLNFNGDTTLQNNANITLGNKSQAAFKNSLTLDNNSNLSLDNQSVLNASGTSAFNNQASLNIYNGSHSJM:HPSJM_04695  TQAVFNVLYSRGTSNFNATTQLLGNTNFTLSSQSLLNFNGDTTLQNNANITLGNKSQAAFKNSLTLDNNSNLSLDNQSVLNANGASAFNNQASLNIYNGSHP12:HPP12_0919   TQAVFNVLYSRGTSNFNATTQLLGNTNFTLSSQSLLNFNGDTTLQNNANITLGNKSQADFKNSLTLNNNSNLSLDNQSVLNANGTSAFNNQASLNIYNGSHHPA:HPAG1_0903   TQAVFNVLHSRGTSNFNATTQLLGNTNFTLSSQSLLNFNGDTTLQNNANITLGNKSQAAFKNSLTLDNDSNLSLDNQSVLNANGASAFNNQASLNIYNGSH266:HP0922       TQAVFNVLYSRGTSNFNATTQLLGNTNFTLSSQSLLNFNGDTTLQNNANITLGNKSQAAFKNSLTLDNNSNLSLDNQSVLNANNTSAFNNQASLNIYNGSHG27:HPG27_871    TQAVFNVLYSRGTSNFNATTQLLGNTNFTLSSQSLLNFNGDTTLQNNANITLGNKSQADFKNSLTLDNNSNLSLDNQSVLNANNTSAFNNQASLNIYNGSHB38:mHELPY_0906  TQAVFNVLYSRGTSNFNATTQLLGNTNFTLSSQSLLNFNGDTTLQDNANITLGNKSQAAFKNSLTLDNDSNLSLDNQSVLNANGASAFNNQASLNIYNGSHF32:HPF32_0434   TQAVFNVLYSRGTSNFNATTQLLGNTNFTLSSQSLLNFNGDTTLQNNANITLGNKSQAAFKNSLTLDNNSNLSLDNQSVLNANGASAFNNQASLNIYNGSHF57:HPF57_0931   TQAVFNVLYSRGASNFNATTQLLGNTSFTLSSQSLLNFNGDTTLQNNANITLGNKSQAAFKNSLTLDNNSNLSLDNQSVLNANGASAFNNQASLNIYNGSH51:KHP_0860      TQAVFNVLYSRGTSNFNATTQLLGNTSFTLSSQSLLNFNSDTTLQNNANITLGNKSQAAFKNSLTLDNNSNLSLDNQSVLNANGSSAFNNQASLNIYNGSHF16:HPF16_0901   TQAVFNVLYSRGTSNFNATTQLLGNTSFTLSSQSLLNFNGDTTLQNNANITLGNKSQAAFKNSLTLDNNSNLSLDNQSVLNANGASAFNNQASLNIYNGSH52:HPKB_0890     TQAVFNVLYSRGTSNFNATTQLLGNTNFTLSSQSLLNFNGDTTLQNNANITLGNKSQAAFKNSLTLDNNSNLSLDNQSVLNANNTSAFNNQASLNIYNGSHF30:HPF30_0419   TQAVFNVLYSRGTSNFNATTQLLGNTSFTLSSQSLLNFNGDTTLQNNANITLGNKSQAAFKNSLTLDNNSNLSLDNQSVLNANGASAFNNQASLNIYNRS                  1101      1111      1121      1131      1141      1151      1161      1171      1181      1191                        |         |         |         |         |         |         |         |         |         |         HB8:HPB8_627      QAAFNSLFFNGGTLSLNASSKLNASNASFSSNTTINLDDSVLSASNTSSLNANINFQGASQADFGGNTTIDTASFNFDSASSLSFNNLTANGALNFNGYAHSJM:HPSJM_04695  QAAFSSLFFNGGTLSLNANSKLNTSNASFSNNTTINLDDSVLNANNTSSLNANINFQGTSQADFGGNTTIDTASFNFDSASSLSFNNLTANGALNFNGYAHP12:HPP12_0919   QATFSSLFFNGGTLSLNASSKLNASSASFSNNTTINLDDSVLSANNTSSLNANINFQGASQADFGGNTTIDTASFNFDSASSLNFNNLTANGALNFNGYAHHPA:HPAG1_0903   QATFKSLFFNGGTLSLNASSKLNASSASFSNNTTINLDDSVLSASNTSSLNANINFQGASQADFGGNTTINTASFNFDSASSLSFNNLTANGALNFNGYTH266:HP0922       QATFNSLFFNGGTLSLNASSKLNASNASFSNNTTINLDDSVLSASNTSSLNANINFQGASQADFGGNTIIDTASFNFDSASSLNFNNLTANGALNFNGYTHG27:HPG27_871    QATFSSLFFNGGTLSLNASSKLNASNASFSNNTTINLDDSVLSANNTSSLNANINFQGASQADFGGNTTIDTASFNFDSASSLNFNNLTANGALNFNGYAHB38:mHELPY_0906  QAAFSSLFFNGGTLSLNASSKLNASSASFSNNTTINLDDSVLSASNTSSLNANINFQGASQADFGGNTTIDTASFNFDSTSSLSFNNLTANGALNFNGYAHF32:HPF32_0434   QATFKSLFFNGGTLSLNASSKLNASNASFSNNTTINLDDSVLSASNTSSLNANINFQGASQANFGGNTTIDTASFNFDSASSLSFNNLTANGALNFNGYAHF57:HPF57_0931   QATFNSLFFNGGILSLNANSKLNASSASFSNNTTINLDDSVLSVSNASSLNANINFQGASQADFGGNTTINTASFNFDSASSLSFNNLTANGALNFNGYAH51:KHP_0860      QATFKSLFFNGGTLSLNASSKLNASSASFSNNTTINLDDSVLSANNTSSLNANINFQGTSQANFGGNTTINTASFNFDSASSLSFNNLTANGALNFNGYAHF16:HPF16_0901   QATFNSLFFNGGILSLNANSKLNASSASFSNNTTINLDDSVLSASNTSSLNANINFQGTSQANFGGNTTINTASFNFDSASSLSFNNLTANGALNFNGYAH52:HPKB_0890     QATFKSLFFNGGTLSLNASSKLNASRASFSNNTTINLDDSVLSASNTSSLNANINFQGASQADFGGNTTIDTASFNFDSASSLNFNNLTANGALNFNGYAHF30:HPF30_0419   QATFNSLFFNGGIISLNANSKLNASSASFSNNTTINLDDSVLSASNTSSLNANINFQGASQANFGGNTTIDTASFNFDSTSSLSFNNLTANGALNFNGYA                  1201      1211      1221      1231      1241      1251      1261      1271      1281      1291                        |         |         |         |         |         |         |         |         |         |         HB8:HPB8_627      PSLTKALMSVSGQFVLGNNGDINLSDINIFDNITKSVTYNILNAQKGITGISGANGYEKILFYGMKIQNATYSDNNNIQTWSFINPLNSSQIIQESIKNGHSJM:HPSJM_04695  PSLTKALMSVSGQFVLGNNGDINLSDINIFDNITKSVTYNILNAQKGITGISGANGYEKILFYGMKIQNATYSDNNNIQTWSFINPLNSSQIIQESIKNGHP12:HPP12_0919   LSLTKALISVSGQFVLGNNGDINLSDINIFDNITKSVTYNILNAQKGITGISGANGYEKILFYGMKIQNAIYSDNNNIQTWSFINPLNSSQIIQESIKNGHHPA:HPAG1_0903   PSLTKALMSVSGQFVLGNNGDINLSDINIFDNITKSVTYNILNAQKGITGISGANGYEKILFYGMKIQNATYSGNNNIQTWSFINPLNSSQIIQESIKNGH266:HP0922       PSLTKALMSVSGQFVLGNNGDINLSDINIFDNITKSVTYNILNAQKGITGISGANGYEKILFYGMKIQNATYSDNNNIQTWSFINPLNSSQIIQESIKNGHG27:HPG27_871    PSLAKALMSVSGQFVLGNNGDINLSDINIFDNITKSVTYNILNAQKGITGISGANGYEKILFYGMKIQNATYSDNNNIQTWSFINPLNSSQIIQESIKNGHB38:mHELPY_0906  PSLTKALMSVSGQFVLGNNGDINLSDINIFDNITKSVTYNILNAQKGITGISGANGYEKILFYGMKIQNATYSDNNNIQTWSFINPLNSSQIIQESIKNGHF32:HPF32_0434   PSLSKALMSVSGQFVLGNNGDINLSDINIFDNITKSVTYSILNAQKGITGISGANGYEKILFYGMKIQNATYSDNNNIQTWSFINPLNSSQIIQESIKNGHF57:HPF57_0931   PSLTKALMSVSGQFVLGNNGDINLSDINIFDNITKSVTYNILNAQKGITGISGANGYEKILFYGMKIQNATYSDNNNIQTWSFINPLNSSQIIQESIKNGH51:KHP_0860      PSLTKALMSVSGQFVLGNNGDINLSDINIFDNITKSVTYNILNAQKGITGISGANGYEKILFYGMKIQNATYSGNNNIQTWSFINPLNSSQIIQESIKNGHF16:HPF16_0901   PSLTKALMSVSGQFVLGNNGDINLSDINIFGNITRSVTYNILNVQKGITGISGANGYEKILFYGMKIQNATYSDNNNIQTWSFINPLNSSQIIQESIKNGH52:HPKB_0890     PSLTKALMSVSGQFVLGNNGDINLSDINIFDNITKSVTYNILNAQKGITGISGANGYEKILFYGMKIQNATYSGNNNIQTWSFINPLNSSQIIQESIKNGHF30:HPF30_0419   PSLSKALMSVSGQFVLGNNGDINLSDINIFDNITKSVTYNILNAQKGITGISGANGYEKILFYGMKIQNATYSDNNNIQTWSFINPLNSSQIIQESIKNG                  1301      1311      1321      1331      1341      1351      1361      1371      1381      1391                        |         |         |         |         |         |         |         |         |         |         HB8:HPB8_627      DLTIEVLNNPNSASNTIFNIAPELYNYQASKQNPTGYSYDYSDDQAGTYYLTSNIKGLFTPKGSQTPQAPGTYSPFNQPLNSLNIYNKGFSSENLKTLLGHSJM:HPSJM_04695  DLTIEVLNNPNSASNTIFNIAPELYNYQASKQNLTGYSYDYSDNQAGTYYLTSNIKGLFTPKGSQTPQTPGTYSPFNQPLNSLNIYNKGFSSENLKTLLGHP12:HPP12_0919   DLTIEVLNNPNSASNTIFNIAPELYNYQASKQNPTGYSYDYSDNQAGTYYLTSNIKGLFTPKGSQTPQTPGTYSPFNQPLNSLNIYNKGFSSENLKTLLGHHPA:HPAG1_0903   DLTIEILNNPNSASNTIFNIAPELYNYQASKQNPTGYSYDYSDNQAGTYYLTSNIKGLFTPKGSQTPQTPGTYSPFNQPLNSLNIYNKGFSSENLKTLLGH266:HP0922       DLTIEVLNNPNSASNTIFNIAPELYNYQASKQNPTGYSYDYSDNQAGTYYLTSNIKGLFTPKGSQTPQAPGTYSPFNQPLSSLNIYNKGFSSENLKTLLGHG27:HPG27_871    DLTIEVLNNPNSASNTIFNIAPELYNYQASKQNPTGYSYDYSDNQAGTYYLTSNIKGLFTPKGSQTPQTPGTYSPFNQPLNSLNIYNKGFSSENLKTLLGHB38:mHELPY_0906  DLTIEVLNNPNSASNTIFNIAPELYNYQASKQNPTGYSYDYSDNQAGTYYLTSNIKGLFTPKGSQTPQAPGTYSPFNQPLNSLNIYNKGFSSENLKTLLGHF32:HPF32_0434   DLTIEVLNNPNSASNTIFNIAPELYNYQASKQNPTGYSYDYSDNQAGTYYLTSNIKGLFTPKGSQTPQTPGTYSPFNQPLNSLNIYNKGFSSGNLKTLLGHF57:HPF57_0931   DLTIEVLNNPNSASNTIFNIAPELYNYQASKQNPTGYSYDYNDNQAGTYYLTSNIKGLFTPKGSQTPQTPGTYSPFNQPLNSLNIYNKGFSSGNLKTLLGH51:KHP_0860      DLTIEVLNNPNSASNTIFNIAPELYNYQASKQNPTGYSYDYSDNQAGTYYLTSNIKGLFTPKGSQTPQTPGTYSPFNQPLNSLNIYNKGFSSGNLKTLLGHF16:HPF16_0901   DLTIEVLNNPNSASNTIFNIAPELYNYQASKQNPTGYSYDYSDNQVGTYYLTSNIKGLFTPKGSQTPQIPGTYSPFNQPLNSLNICNKGFSSGNLKTLLGH52:HPKB_0890     DLTIEVLNNPNSASNTIFNIAPELYNYQASKQNPTGYSYDYSDNQAGTYYLTSNIKGLFTPKGSQTPQTPGTYSPFNQPLNSLNIYNKGFSSGNLKTLLGHF30:HPF30_0419   DLTIEVLNNPNSASNTIFNIAPELYNYQASKQNPTGYSYDYSDNQVGTYYLTSNIKGLFTPKGSQTPQTPGTYSPFNQPLNSLNIYNKGFSSGNLKTLLG                  1401      1411      1421      1431      1441      1451      1461      1471      1481      1491                        |         |         |         |         |         |         |         |         |         |         HB8:HPB8_627      ILSQNSATLKEMIESNQLDNITNINEVLQLLDRIKITPAQKQALLETINHLTDNINQTFNNGNLIIGATQDNVTNSTSSIWFGGNGYSSPCALDSATCSSHSJM:HPSJM_04695  ILSQNSAALKEMIESNQLDNITNINEVLQLLDKIKITQTQKQALLETINHLTDNINQTFNNGNLIIGATQDNVTNSTSSIWFGGNGYSSPCALDSATCSSHP12:HPP12_0919   FLSQNSATLKEMIESNQLDNITNINEVLQLLDKIKITQTQKQALLDIINHLTDNINQTFSNGNLIIGATQDNVTNSTSSIWFGGNGYSSPCTLDSATCSSHHPA:HPAG1_0903   ILSQNSATLKEMIESNQLDNITNINEVLQLLDKIKITQTQKQALLETINHLTDNINQTFNNGNLIIGATQDNVTNSTSSIWFGGNGYSSPCALDSATCSSH266:HP0922       ILSQNSATLKEMIESNQLDNITNINEVLQLLDKIKITQVQKQALLETINHLTDNINQTFNNGNLIIGATQDNVTNSTSSIWFGGNGYSSPCTLDSATCSSHG27:HPG27_871    ILSQNSVALKEMIESNQLDNITNINEVLQLLDKIKITQTQKQVLLETINHLTDNINQTFNNGNLIIGATQDNVTNSTSSIWFGGNGYSSPCALDSATCSSHB38:mHELPY_0906  ILSQNSATLKEMIESNQLDNITNINEVLQLLDKIKITPAQKQALLETINHLTDNINQTFSNGNLIIGATQDNVTNSTSSIWFGGNGYSSPCMLDSATCSSHF32:HPF32_0434   ILSQNSATLKEMIESNQLDNITSINEVLQLLDKIKITPAQKQALLETINHLTDNINQTFSNGNLVIGATQDNVTNSTSSIWFGGNGYSSPCALDSATCSSHF57:HPF57_0931   ILSQNSATLKETIESNQLDNITNINEVLQLLDKIKITPAQKQALLETINHLTDNINQTFSNGNLVIGATQDNVTNSTSSIWFGGNGYSSPCALDSATCSSH51:KHP_0860      ILSQNSATLKEMIESNQLDNITSINEVLQLLDKIKITPTQKQALLETINHLTDNINQTFSNGNLVIGATQDNVTNSTSSIWFGGNGYSSPCVLDSATCSSHF16:HPF16_0901   ILSQNSATLKEMIESNQLDNITSINEVLQLLDEIKITPAQKQALLETINHLTDNINQTFNNGNLVIGTTQDNVTNSTSSIWFGGNGYSSPCVLDSATCSSH52:HPKB_0890     ILSQNSATLKEMIESNQLDNITSINEVLQLLDKIKITPAQKQELLETINHLTDNINQTFSNGNLVIGSTQDNATNSTSSIWFGGNGYSSPCALDSATCSSHF30:HPF30_0419   ILSQNSATLKEMIESNQLDNITSINEVLQLLDKIKITPAQKQALLETINHLTDNINQTFSNGNLVIGATQDNVTNSTSSIWFGGNGYSSPCTLDSATCSS                  1501      1511      1521      1531      1541      1551      1561      1571      1581      1591                        |         |         |         |         |         |         |         |         |         |         HB8:HPB8_627      FRNTYLGQLLGSTSPYLGYINADFKAKSIYITGTMGSGNAFESGGSADVTFQSANNLVLNKANIEAQATDNIFNLLGQKGIEKIFNQGNLANVLSQVAMEHSJM:HPSJM_04695  FRNTYLGQLLGSTSPYLGYINADFKAKSIYITGTIGSGNAFESGGSADVTFQSANNLVLNKANIEAQATDNIFNLLGQEGIDKIFNQGNLANVLSQVAMEHP12:HPP12_0919   FRNTYLGQLLGSTSPYLGYINADFKAKSIYITGTIGSGNAFESGGSADVTFQSANNLVLNKANIEAQATDNIFNLLGQKGINEIFNQGNLANVLSQMAMEHHPA:HPAG1_0903   FRNTYLGQLLGSTSPYLGYINADFKAKSIYITGTIGSGNAFESGGSADVTFQSTNNLVLNKANIEAQATDNIFNLLGQEGIDKIFNQGNLANVLSQMAMEH266:HP0922       FRNTYLGQLLGSTSPYLGYINADFKAKSIYITGTIGSGNAFESGGSADVTFQSANNLVLNKANIEAQATDNIFNLLGQKGIEKIFNQGNLANVLSQVAMEHG27:HPG27_871    FRNTYLGQLLGSTSPYLGYINADFKAKSIYITGTLGSGNAFESGGSADVTFQSANNLVLNKANIEAQATDNIFNLLGQEGIDKIFNQGNLANVLSQVAMEHB38:mHELPY_0906  FRNTYLGQLLGSTSPYLGYINADFKAKSIYITGTLGSGNAFESGGSADVTFQSANNLVLNKANIEAQATDNIFNLLGQEGIDKIFNQGNLANVLSQVAMEHF32:HPF32_0434   FRNTYLGQLLGSTSPYLGYINADFKAKSIYITGTLGSGNAFESGGSADVTFQSANNLVLNKANIEAQATDNIFNLLGQEGIDKIFNQGNLANVLSQVAMEHF57:HPF57_0931   FRNTYLGQLLGSTSPYLGYINADFKAKSIYITGTLGSGNAFESGGSADVTFQSANNLVLDKANIEAQATDNIFNLLGQEGIDKIFNQGNLANVLSQVAMEH51:KHP_0860      FRNTYLGQLLGSTSPYLGYINADFKAKSIYITGTLGSGNAFESGGSADVTFQSANNLVLNKANIEAQATDNIFNLLGQEGIDKIFNQGNLANVLSQVAMEHF16:HPF16_0901   FRNTYLGQLLGSTSPYLGYINADFKAKSIYITGTLGSGNAFESGGSADVTFQSANNLVLNKANIEAQATDNIFNLLGQEGIDKIFNQGNLANVLSQVAMEH52:HPKB_0890     FRNTYLGQLLGSTSPYLGYINADFKAKSIYITGTLGSANAFESGGSADVTFQSANNLVLDKANIEAQATDNIFNLLGQEGIDKIFNQGNLANVLSQVAMEHF30:HPF30_0419   FRNTYLGQLLGSTSPYLGYINADFKAKSIYITGTLGSANAFESGGSADVTFQSANNLVLDKANIEAQATDNIFNLLGQEGIDKIFNQGNLANVLSQVAME                  1601      1611      1621      1631      1641      1651      1661      1671      1681      1691                        |         |         |         |         |         |         |         |         |         |         HB8:HPB8_627      KIKQAGGLGNFIENALSPLSKELPASLQNETLGQLIGPNNLDDLLNNSGVMNAIQNIISKKLSIFGNFVTPSIIENYLAKQSLKSMLDDKGLLNFIGGYMHSJM:HPSJM_04695  KIKQAGGLGNFIENALSPLSKELPASLQNETLGQLIGQNNLDDLLNNSGVMNAIQNIISKKLSIFGNFVTPSIIENYLAKQSLKSMLDDKGLLNFIGGYIHP12:HPP12_0919   KIKQAGGLGNFIENALSPLSKELPSSLQSETLGQLIGQNNLDDLLNNSGVMNAIQNIISKKLSIFGNFVTPSIIENYLAKQSLKSMLDDKGLLNFIGGYIHHPA:HPAG1_0903   KIKQAGGLGNFIENALSPLSKELPASLQDETLGQLIGQNNLDDLLNNSGVMNAIQNIISKKLSIFGNFVTPSIIENYLAKQSLKSMLDDKGLLNFIGGYIH266:HP0922       KIKQAGGLGNFIENALSPLSKELPASLQNETLGQLIGQNNLDDLLNNSGVMNAIQNIISKKLSIFGNFVTPSIIENYLAKQSLKSMLDDKGLLNFIGGYMHG27:HPG27_871    KIKQAGGLGNFIENALSPLSKELPASLQDETLGQLIGQNNLDDLLNNSGIMNAIQNIISKKLSIFGNFVTPSIIENYLAKQSLKSMLDDKGLLNFIGGYIHB38:mHELPY_0906  KIKQAGGLGNFVENALSPLSKELPASLQSETLGQLIGQNNLDDLLNNSGVMNAIQNIISKKLSIFGNFVTPSIIENYLAKQSLKSMLDDKGLLNFIGGYIHF32:HPF32_0434   KIKQAGGLGNFVENALSPLSKELPASLQNETLGQLIGQNNLDDLLNNSGVMNAIQNIISKKLSIFGNFVTPSIIENYLAKQSLKSMLDDKGLLNFIGGYMHF57:HPF57_0931   KIKQAGGLGNFVENALSPLSKELPTSLQNETLGQLIGQNNLDNLLNNSGVMNEIQNIISKKLSIFGNFVTPSIIENYLAKQSLKSMLDDKGLLNFIGGYIH51:KHP_0860      KIKQAGGLGNFIENALSPLSKELPTSLQNETLGQLIGQNNLDNLLNNSGVMNAIQNIISKKLSIFGNFVTPSIIENYLAKQSLKSMLDDKGLLNFIGGYIHF16:HPF16_0901   KIKQAGGLGNFVENALSPLSKELPTSLQNETLGQLIGQNNLDNLLNNSGVMNEIQNIISKKLSIFGNFVTPSIIENYLAKQSLKSMLDDKGLLNFIGGYIH52:HPKB_0890     KIKQAGGLGNFVENALIPLSKELPASLQDETLGQLIGQNNLDNLLNNSGVMNEIQNIISKKLSIFGNFVTPSIIENYLAKQSLKSMLDDKGLLNFIGGYIHF30:HPF30_0419   KIKQAGGLGNFVENALSPLSKELPTSLQNETLGQLIGQNNLDNLLNNSGVMNAIQNIISKKLSIFGNFVTPSIIENYLAKQSLKSMLDDKGLLNFIGGYI                  1701      1711      1721      1731      1741      1751      1761      1771      1781      1791                        |         |         |         |         |         |         |         |         |         |         HB8:HPB8_627      DASELSSILSVVLKDITNPPTSLQKDIGVVANDLLNEFLGQDVIKKLENQGLVSNIINNIISQGGLSGVYNQGLGSVLPPSLQNALKENDLGALLSPRGLHSJM:HPSJM_04695  DASELSSILSVVLKDITNPPASLQKDIGVVANDLLNEFLGQDVVKKLESQGLVNNIINNIISQGGLSGVYNQGLGSVLPPSLQNALKENDLGALLSPRGLHP12:HPP12_0919   DASELSSILGVILKDITNPPTSLQKDIGVVANDLLNEFLGQDVVEKLESQGLVNNIINNIISQGGLSGVYNQGLGSVLPPSLQNALKENDLGALLSPRGLHHPA:HPAG1_0903   DASELSSILSVILKDITNPPTSLQKDIGVVANDLLNEFLGQDVIKKLESQELVNNIINNIISQGGLSGVYNQGLGSVLPPSLQNALKENDLGALLSPRGLH266:HP0922       NASELSSILSVVLKDITNPPTSLQKDIGVVANDLLNEFLGQDVIKKLESQGLVSNIINNIISQGGLSGVYNQGLGSVLPPSLQNALKENDLGTLLSPRGLHG27:HPG27_871    DASELSSILSVILKDITNPPTSLQKDIGVVANDLLNEFLGQDVVKKLESQGLVNSIINNIISQGGLSGVYNQGLGSVLPPSLQNALKGNDLGALLSPRGLHB38:mHELPY_0906  DASELSSILSVILKDITNPPASLQKDIGVVANDLLKEFLGQDVVKKLESQGLVSNIINNIISQGGLSDVYNQGLGSVLPPSLQNALKENDLGALLSPRGLHF32:HPF32_0434   DASELSSILSVILKDITNPPTSLQKDIGVVANDLLNEFLGQDVVKKLESQGLVSNIINNIISQGGLSGIYNQGLGSVLPLSLQNALKENDLGALLSPRGLHF57:HPF57_0931   DASELSSILSVILKDITNPPTSLQKDIGVVANDLLNEFLGQDVVKKLESQGLVSNIINNIISQGGLSGVYNQGLGSVLPPSLQNALKENDLGALLSPRGLH51:KHP_0860      DASELSSILSVILKDITNPPTSLQKDIGVVANDLLNEFLGQDVVKKLESQGLVSNIINNIISQGGLSGIYNQGLGSVLPLSLQNALKENDLGALLSPRGLHF16:HPF16_0901   DASELSSILSVILKDITNPPTSLQKDIGVVANDLLNEFLGQDVVKKLESQGLVSNIINNIISQGGLSGVYNQGLGSMLPPSLQNALKENDLGALLSPRGLH52:HPKB_0890     DASELSSILSVVLKDIINPPTSLQKDIGVVANDLLNEFLGQDVVKKLESQGLVSNIINNIISQGGLSGVYNQGLGSVLPPSLQNALKENDLGALLSPRGLHF30:HPF30_0419   DASELSSILSVILKDITNPPTSLQKDIGVVANDLLNEFLGQDIVKKLESQGLVSNIINNIISQGGLSGVYNQGLGSVLPPSLQNALKENDLGALLSPRGL                  1801      1811      1821      1831      1841      1851      1861      1871      1881      1891                        |         |         |         |         |         |         |         |         |         |         HB8:HPB8_627      HDFWQKGYFNFLSNSYVFVNNSSFSNATGGSLNFVANKSIIFNGDNTIDFSKYQGALIFASNDVSNINITTLNATNGLSLNAGLNNVSVQKGEICVNLANHSJM:HPSJM_04695  HDFWQKGYFNFLSNGYVFVNNSSFSNATGGSLNFVANKSIIFNGDNTIDFSKYQGALIFASNGVSNINITTLNATNGLSLNAGLNNVSVQKGEICVNLANHP12:HPP12_0919   HDFWQKGYFNFLSNGYVFVNNSSFSNATGGSLNFVANKSIIFNGDNTIDFSKYQGALIFASNGVSNINITTLNATNGLSLNAGLNNVSVQKGEICVNLANHHPA:HPAG1_0903   HDFWQKGYFNFLSNGYVFVNNSSFSNATGGSLNFVANKSIIFDGDNTIDFSKYQGALIFASNDVSNINITTLNATNGLSLNAGLNNVSVQKGEICVNLANH266:HP0922       HDFWQKGYFNFLSNGYVFVNNSSFSNATGGSLNFVANKSIIFNGDNTIDFSKYQGALIFASNDVSNINITTLNATNGLSLNAGLNNVSVQKGEICVNLANHG27:HPG27_871    HDFWQKGYFNFLSNGYVFVNNSSFSNATGGSLNFVANKSIIFNGDNTIDFSKYQGALIFASNGVSNINITTLNATNGLSLNAGLNNVSVQKGEICVNLANHB38:mHELPY_0906  HDFWQKGYFNFLSNGYVFVNNSSFSNATGGSLNFVANKSIIFNGDNTIDFSKYQGVLIFASNGVSNINITTLNATNGLSLNAGLNNVSVQKGEICVNLANHF32:HPF32_0434   HDFWQKGYFNFLSNGYVFVNNSSFSNATGGSLNFVANKSIIFNGDNTIDFSKYQGALIFASNGVSNINITTLNATNGLSLNAGLNNVSVQKGEICINLANHF57:HPF57_0931   HDFWQKGYFNFLSNGYVFVNNSSFSNATGGSLNFVANKSIIFNGDNTIDFSKYQGALIFASNGVSNINITTLNATNGLSLNAGLNNVSVQKGEICINLANH51:KHP_0860      HDFWQKGYFNFLSNGYVFVNNSSFSNATGGSLNFVANKSIIFNGDNTIDFSKYQGALIFASNGVSNINITTLNATNGLSLNAGLNNVSVQKGEICINLANHF16:HPF16_0901   HDFWQKGYFNFLSNGYVFVNNSSFSNATGGSLNFVANKSIIFNGDNTIDFSKYQGALIFASNGVSNINITTLNVTNGLSLNAGLNNVSVQKGEICINLANH52:HPKB_0890     HDFWQKGYFNFLSNGYVFVNNSSFSNATGGSLNFVANKSIIFNGDNTIDFSKYQGALIFASNGVSNINITTLNATNGLSLNAGLNNVSVQKGEICVNLANHF30:HPF30_0419   HDFWQKGYFNFLSNGYVFVNNSSFSNATGGSLNFVANKSIIFDGDNTIDFSKYQGALIFASNGVSNINITTLNATNGLSLNAGLNNVSVQKGEICINLAN                  1901      1911      1921      1931      1941      1951      1961      1971      1981      1991                        |         |         |         |         |         |         |         |         |         |         HB8:HPB8_627      CPTTKNSSSTNSSVTPTNESLSVRANNFTFLGVIASNGAIDLSQVKNNSVIGTLNLNENAALQANNLTITNAFNNTSNSTANINGNFTLNQQATLSTNASHSJM:HPSJM_04695  CPTTKNSSSTNSSVTPTNESLSVRANNFTFLGAITSNGAIDLSQVTNNSVIGTLNLNENATLQANNLTITNAFNNASNSTANINGNFTLNQQATLSTNANHP12:HPP12_0919   CPTTKNSSSTNSSVTPTNESLSVRANNFTFLGAITSNGAIDLSQVTNNSVIGTLNLNENATLQANNLTITNAFNNASNSTANINGNFTLNQQATLSTNASHHPA:HPAG1_0903   CPTTKNSSSTNSSVTPTNESLSVHANSFTFLGVIASNGAIDLSQVKNNSVIGTLNLNENATLQANNLTIANAFNNASNSTANINGDFTLNQQATLSTNASH266:HP0922       CPTTKNSSSTNSSVTPTNESLSVRANNFTFLGAIASNGAIDLSQVKNNSVIDTLNLNENAALQANNLTITNAFNNASNSTANINGNFTLNQQATLSTNASHG27:HPG27_871    CPTTKNSSSTNSSVTPTNESLSVHANNFTFLGAITSNGAIDLSQVKNNSVIGTLNLNENATLQANNLTITNAFNNASNSTANINGNFTLNQQATLSTNASHB38:mHELPY_0906  CPTTKNNSSTNSSVTPTNESLSVRANNFTFLGAITSNGAIDLSQVTNNSVIGTLNLNENATLQANNLTITNAFNNASNSTANINGNFTLNQQATLSTNASHF32:HPF32_0434   CPTTKNSSSTNSSVTPTNESLSVRANNFTFLGVIASNGAIDLSQVKNNSVIGTLNLNENATLQANNLTITNAFNNASNSTANINGNFTLNQQATLSTNASHF57:HPF57_0931   CPTTKNSSSTNSSVTPTNESLSVRANNFTFLGTIASNGAIDLSQVKNNSVIGTLNLNENATLQANNLTITNAFNNASNSTANINGNFTLNQQATLSTNASH51:KHP_0860      CPTTKNSSSTNSSVTPTDESLSVRANNFTFLGTIASNGAIDLSQVKNNSVIGTLNLNENATLQANNLTITNAFNNASNSTANINGNFTLNQQATLSTNASHF16:HPF16_0901   CPTTKNSSSTNSSVTPTNESLSVRANNFTFLGTIASNGAIDLSQVKDNSVIGTLNLNENATLQANNLTITNAFNNASNSTANINGSFTLNQQATLSTNASH52:HPKB_0890     CPTTKNSSSTNSSVTPTNESLSVRANNFTFLGVIASNGAIDLSQVKNNSVIGTLNLNENATLQANNLTITNAFNNASNSTANINGDFTLNQQATLSTNASHF30:HPF30_0419   CPTTKNSSSTNSSVTPTDESLSVRANNFTFLGTIASNGAIDLSQVKNNSVIGTLNLNENATLQANNLTITNAFNNASNSTANINGNFTLNQQAALSTNAS                  2001      2011      2021      2031      2041      2051      2061      2071      2081      2091                        |         |         |         |         |         |         |         |         |         |         HB8:HPB8_627      GLNVMGNFNSYGDLVFNLSHSVSHAIINAQGSATIMANNNNPLIQFNTSSKETGTYTLIDSVKAIYYGYNDQITGGSSLDNYLKLYTLIDINGKHMVMTDHSJM:HPSJM_04695  GLNVMGNFNSYGDLVFNLSHSVSHAIINAQGNATIMANDNNPLIQFNTSSKETGTYTLIDSAKAIYYGYNNQITGGSSLDNYLKLYALIDINGKHMVMTDHP12:HPP12_0919   GLNVMGNFNSYGDLVFNLSHSVSHAIINAQGAATIMANDNNPLIQFNTSSKEAGTYTLIDSAKAIYYGYNDQITGGSSLDNYLKLYVLIDINGKHMVMTDHHPA:HPAG1_0903   GLNVMGNFNSYGDLVFNLSHSVSHAIINAQGSATIMANNNNPLIQFNTSSKETGTYTLINSAKAIYYGYNDQITGGSSLDNYLKLYALIDINGKHMVMTDH266:HP0922       GLNVMGNFNSYGDLVFNLSHSVSHAIINAQGSATIMANNNNPLIQFNTSSKEVGTYTLIDSAKAIYYGYNNQITGGSSLDNYLKLYTLIDINGKHMVMTDHG27:HPG27_871    GLNVMGNFNSYGDLVFNLIHSVSHAIINAQGNATIMANNNNPLIQFNTSSKEVGTYTLIDSAKAIYYGYNDQITGGSSLDNYLKLYVLIDINGKHMVMTDHB38:mHELPY_0906  GLNVMGNFNSYGDLVFNLSHSVSHAIINAQGNATIMANDNNPLIQFNTSSKEVGTYTLIDSAKAIYYGYNDQITGGSSLDNYLKLYTLIDINGKHMVMTGHF32:HPF32_0434   GLNVMGNFNSYGDLVFNLSHSASHAIINAQGTATIMANNNSPLIQFNTSSKETGAYTLIDSAKAIYYGYNDQITGGNSLADYLKLYTLIDINGKRMVMSNHF57:HPF57_0931   GLNVMGNFNSYGDLVFNLSHSASHAIINAQGVATIMANNNNPLIQFNTSSKEAGAYTLIDSAKAIYYGYNDQITGGNSLADYLKLYTLIDINGKRMAMTDH51:KHP_0860      GLNVMGNFNSYGDLVFNLSHSASHAIINAQGIATIMANNNNPLIQFNTSSKETGAYTLIDSTKAIYYGYNDQITGGNSLADYLKLYTLIDINGKHMVMTDHF16:HPF16_0901   GLNVMGNFNSYGDLVFNLSHSASHAIISAQGVATIMANNNNPLIQFNTSSKETNTYTLIDSAKAIYYGYNDQITGGNSLADYLKLYTLIDINGKRMVMSNH52:HPKB_0890     GLNVMGNFNSYGDLVFNLSHSVSHAIINAQGTATIMANNNNPLIQFNTSSKETGAYTLIDSAKAIYYGYNDQITGGNSLADYLKLYTLIDINGKRMVMTDHF30:HPF30_0419   GLNVMGNFNSYGDLVFNLSHSASHAIISAQGVATIMANNNNPLIQFNTSSKETNTYTLIDSAKAIYYGYNDQITGGNSLDNYLKLYTLIDINGKHMVMSN                  2101      2111      2121      2131      2141      2151      2161      2171      2181      2191                        |         |         |         |         |         |         |         |         |         |         HB8:HPB8_627      NGLTYNGQAVNIKDGGLVVGFKDSQNQYIYTSILYNKVKIAVSNDPINNLQAPTLKQYIAQIQGTQSVDSIDQAGGSQAINWLNKIFETKGSPLFAPYYLHSJM:HPSJM_04695  NGLTYNGQAVSVKDGGLVVGFKDSQNQYIYTSILYNKVKIAVSNDPINNLQAPTLKQYIAQIQGVQSVDSIDQSGGNQAIDWLNKIFETKGSPLFAPYYLHP12:HPP12_0919   NGLTYNGQAVNIKDGGLVVGFKDSQNQYIYTSILYNKVKIAVSNDPINNLQAPTLKQYIAQIQGTQGVDSIDQAGGNQAINWLNKIFETKGSPLFAPYYLHHPA:HPAG1_0903   NGLTYNGQAVNIKDGGLVVGFKDSQNQYIYTSILYNKVKIAVSNDPINNLQAPTLKQYIAQIQGVQSVDSIDQVGGNQAINWLNKIFETKGSPLFAPYYLH266:HP0922       NGLTYNGQAVSVKDGGLVVGFKDSQNQYIYTSILYNKVKIAVSNDPINNLQAPTLKQYIAQIQGTQGVDSIDQAGGSQAINWLNKIFETKGSPLFAPYYLHG27:HPG27_871    NGLTYNGQAVNIKDGGLVVGFKDSQNQYIYTSILYNKVKIAVSNDPINNLQAPTLKQYIAQIQGVQSVDSIDQVGGNQAINWLNKIFETKGSPLFAPYYLHB38:mHELPY_0906  NGLTYNGQAVNIKDGGLVVGFKDSQNQYIYTSILYNKVKIAVSNDPINNLQAPTLKQYIAQIQGVQSVDSIDQVGGNQAINWLNKIFETKGSPLFAPYYLHF32:HPF32_0434   NGLTYNGQAVNIKDGGLIVGFKDSQNQYIYTSILYNKVKIAVSNDPISNLQAPTLKQYIAQIQGAQGVDSIEQVGGTQAINWLNKIFETKGSPLFAPYYLHF57:HPF57_0931   NGLTYNGQAVNIKDGGLIVGFKDSQNQYIYTSILYNKVKIAVSNDPINNLQAPTLKQYIAQIQGTQSVDSIDQAGGAQAINWLNKIFETKGSPLFAPYYLH51:KHP_0860      NGLTYNGQAVNIKDGGLVVGFKDSQNQYIYTSILYNKVKIAVSNDPINNLQAPTLKQYIAQIQGTQGVDSIDQAGGNQAISWLNKIFETKGSPLFAPYYLHF16:HPF16_0901   NGLTYNGQAVNIKDGGLIVGFKDSQNQYIYTSILYNKVKIAVSNDPINNLQAPTLKQYIAQIQGAQGVDSIDQAGGTQAINWLNKIFETKGSPLFAPYYLH52:HPKB_0890     NGLTYNGQAVSIKDGGLIVGFKDSQNQYIYTSILYNKVKIAVSNDPINNLQAPTLKQYIAQIQGTQGVDSIEQAGGTQAINWLNKIFETKGSPLFAPYYLHF30:HPF30_0419   NGLTYNDQAVNIKDGGLIVGFKDSQNQYIYTSILYNKVKIAVSNDPINNLQAPTLKQYIAQIQGAQGVDSIEQAGGTQAINWLNKIFETKGSPLFAPYYL                  2201      2211      2221      2231      2241      2251      2261      2271      2281      2291                        |         |         |         |         |         |         |         |         |         |         HB8:HPB8_627      ESHSTKDLTTIAGDIANTLEVIANPNFKNDATNILQINTYTQQMSRLAKLSDTSTFARSDFLERLEALKNKRFADAIPNAMDVILKYSQRNRVKNNVWATHSJM:HPSJM_04695  ESHSVKDLTTIAGDIANTLEVIANPDFKNDATNILQINTYTQQMSRLAKLSDTSTFARSDFLERLEALKNKRFADAIPNAMDVILKYSQRNRVKNNVWATHP12:HPP12_0919   ESHSVKDLTTIAGDIANTLEVIANPDFKNDATNILQINTYTQQMSRLAKLSDTSTFARSDFLERLEALKNKRFADAIPNAMDVILKYSQRNRVKNNVWATHHPA:HPAG1_0903   ESHSTKDLTTIAGDIANTLEVIANPNFKNDATNILQINTYTQQMSRLAKLSDTSTFASADFHERLEALKNKRFADAIPNAMDVILKYSQRNRVKNNVWATH266:HP0922       ESHSTKDLTTIAGDIANTLEVIANPNFKNDATNILQINTYTQQMSRLAKLSDTSTFASADFHERLEALKNKRFADAIPNAMDVILKYSQRNRVKNNVWATHG27:HPG27_871    ESHSAKDLTTIAGDIANTLEVIANPDFKNDATNILQINTYTQQMSRLAKLSDTSTFASADFHERLEALKNKRFADAIPNAMDVILKYSQRNRVKNNVWATHB38:mHELPY_0906  ESHSTKDLTTIAGDIANTLEVIANPDFKNDATNILQINTYTQQMSRLAKLSDTSTFASADFHERLEALKNKRFADAIPNAMDVILKYSQRNRVKNNVWATHF32:HPF32_0434   ESHSTKDLTTIAGDIANTLEVIANPDFKNDATNILQINTYTQQMSRLAKLSDTSTFASADFHERLEALKNKRFADAIPNAMDVILKYSQRNRVKNNVWATHF57:HPF57_0931   ESHSTKDLTTIAGDIANTLEVIANPNFKNDATNILQINTYTQQMSRLAKLSDTSTFASADFHERLEALKNKRFADAIPNAMDVILKYSQRNRVKNNVWATH51:KHP_0860      ESHSTKDLTTIAGDIANTLEVIANPNFKNDATNILQINTYTQQMSRLAKLSDTSTFASADFHERLEALKNKRFADAIPNAMDVILKYSQRNRVKNNVWATHF16:HPF16_0901   ESHSTKDLTTIAGDIANTLEVIANPDFKNDATNILQINTYTQQMSRLAKLSDTSTFASADFHERLEALKNKRFADAIPNAMDVILKYSQRNRVKNNVWATH52:HPKB_0890     ESHSTKDLTTIAGDIANTLEVIANPDFKNDATNILQINTYTQQMSRLAKLSDTSTFASADFHERLEALKNKRFADAIPNAMDVILKYSQRNRVKNNVWATHF30:HPF30_0419   ESHSTKDLTTIAGDIANTLEVIANPNFKNDAANILQINTYTQQMSRLAKLSDTSTFASADFHERLEALKNKRFADAIPNAMDVILKYSQRNRVKNNVWAT                  2301      2311      2321      2331      2341      2351      2361      2371      2381      2391                        |         |         |         |         |         |         |         |         |         |         HB8:HPB8_627      GVGGASFINGGTGTLYGINVGYDRFIKGVIVGGYAAYGYSGFHANITQSGSSNVNMGVYSRAFIKRSELTMSLNETWGYNKTFINSYDPLLSIINQSYRYHSJM:HPSJM_04695  GVGGASFINGGTGTLYGINVGYDRFIKGVIVGGYAAYGYSGFHANITQSGSSNVNMGVYSRAFIKRSELTMSLNETWGYNKTFINSYDPLLSIINQSYKYHP12:HPP12_0919   GVGGASFINGGTGTLYGINVGYDRFIKGVIVGGYAAYGYSGFHGNITQSGSSNVNMGVYSRAFIKRSELTMSLNETWGYNKTFINSYDPLLSIINQSYRYHHPA:HPAG1_0903   GVGGASFINGGTGTLYGINVGYDRFIKGVIVGGYAAYGYSGFHGNITQSGSSNVNMGVYSRAFIKRSELTMSLNETWGYNKTFINSYDPLLSIINQSYRYH266:HP0922       GVGGASFINGGTGTLYGINVGYDRFIKGVIVGGYAAYGYSGFHANITQSGSSNVNMGVYSRAFIKRSELTMSLNETWGYNKTFINSYDPLLSIINQSYKYHG27:HPG27_871    GVGGASFINGGTGTLYGINVGYDRFIKGVIVGGYAAYGYSGFHGNITQSGSSNVNMGVYSRAFIKRSELTMSLNETWGYNKTFINSYDPLLSIINQSYKYHB38:mHELPY_0906  GVGGASFINGGTGTLYGINVGYDRFIKGVIVGGYAAYGYSGFHGNITQSGSSNVNIGVYSRAFIKRSELTMSLNETWGYNKTFINSYDPLLSIINQSYRYHF32:HPF32_0434   GVGGASFINGGTGTLYGINIGYDRFIKGVIVGGYAAYGYSGFHANITQSGSSNVNMGVYSRAFIKRSELTMSLNETWGYNKTFINSYDPLLSIINQSYKYHF57:HPF57_0931   GVGGASFINGGTGTLYGINIGYDRFIKGVIVGGYAAYGYSGFHANITQSGSSNVNMGVYSRAFIKRSELTMSLNETWGYNKTFINSYDPLLSIINQSYKYH51:KHP_0860      GVGGASFINGGTGTLYGINIGYDRFIKGVIVGGYAAYGYSGFHANITQSGSSNVNMGVYSRAFIKRSELTMSLNETWGYNKTFINSYDPLLSIINQSYKYHF16:HPF16_0901   GVGGASFINGGTGTLYGINIGYDRFIKGVIVGGYAAYGYSGFHANITQSGSNNVNMGVYSRAFIKRSELTMSLNETWGYNKTFINSYDPLLSIINQSYKYH52:HPKB_0890     GVGGASFINGGTGTLYGINIGYDRFIKGVIVGGYAAYGYSGFHANITQSGSSNVNMGVYSRAFIKRSELTMSLNETWGYNKTFINSYDPLLSIINQSYKYHF30:HPF30_0419   GVGGASFINGGTGTLYGINIGYDRFIKGVIVGGYAAYGYSGFHANITQSGSSNVNMGVYSRAFIKRSELTMSLNETWGYNKTFINSYDPLLSIINQSYKY                  2401      2411      2421      2431      2441      2451      2461      2471      2481      2491                        |         |         |         |         |         |         |         |         |         |         HB8:HPB8_627      DTWTTDAKINYGYDFMFKDKSVIFKPQVGLAYYYIGLSGLRGIMDDPIYNQFRANADPNKKSVLTINFALESRHYFNKNSYYFVIADVGRDLFINSMGDKHSJM:HPSJM_04695  DTWTTDAKINYGYDFMFKDKSVIFKPQVGLAYYYIGLSGLRGIMDDPIYNQFRANADPNKKSVLTINFALESRHYFNKNSYYFVIADVGRDLFINSMGDKHP12:HPP12_0919   DTWTTDAKINYGYDFMFKDKSVIFKPQVGLAYYYIGLSGLRGIMDDPIYNQFRANADPNKKSVLTINFALESRHYFNKNSYYFVIADVGRDLFINSMGDKHHPA:HPAG1_0903   DTWTTNAKINYGYDFMFKDKSVIFKPQVGLAYYYIGLSGLRGIMDDPIYNQFRANADPNKKSVLTINFALESRHYFNKNSYYFVIADVGRDLFINSMGDKH266:HP0922       DTWTTDAKINYGYDFMFKDKSVIFKPQIGLAYYYIGLSGLRGIMDDPIYNQFRANADPNKKSVLTINFALESRHYFNKNSYYFVIADVGRDLFINSMGDKHG27:HPG27_871    DTWTTDAKINYGYDFMFKDKSVIFKPQIGLAYYYIGLSGLRGIMDDPIYNQFRANADPNKKSVLTINFALESRHYFNKNSYYFVIADVGRDLFINSMGDKHB38:mHELPY_0906  DTWTTDAKINYGYDFMFKDKSVIFKPQVGLAYYYIGLSGLRGIMDDPIYNQFRANADPNKKSVLTINFALESRHYFNKNSYYFVIADVGRDLFINSMGDKHF32:HPF32_0434   DTWTTDAKINYGYDFMFKDKSVIFKPQIGLAYYYIGLSGLRGIMDDPIYNQFRANADPNKKSVLTINFALESRHYFNKNSYYFVIADVGRDLFINSMGDKHF57:HPF57_0931   NTWTTDAKINYGYDFMFKDKSVIFKPQIGLAYYYIGLSGLRGIMDDPIYNQFRANADPNKKSVLTINFALESRHYFNKNSYYFVIADVGRDLFINSMGDKH51:KHP_0860      NTWTTDAKINYGYDFMFKDKSVIFKPQIGLAYYYIGLSGLRGIMDDPIYNQFRANADPNKKSVLTINFALESRHYFNKNSYYFVIADVGRDLFINSMGDKHF16:HPF16_0901   DTWTTDAKINYGYDFMFKDKSVIFKPQIGLAYYYIGLSGLRGIMDDPTYNQFRANADPNKKSVLTINFALESRHYFNKNSYYFVIADVGRDLFINSMGDKH52:HPKB_0890     NTWTTDAKINYGYDFMFKDKSVIFKPQIGLAYYYIGLSGLRGIMDDPIYNQFRANADPNKKSVLTINFALESRHYFNKNSYYFVIADVGRDLFINSMGDKHF30:HPF30_0419   NTWTTDAKINYGYDFMFKDKSVIFKPQIGLAYYYIGLSGLRGIMDDPIYNQFRANADPNKKSVLTINFALESRHYFNKNSYYFVIADVGRDLFINSMGDK                  2501      2511      2521      2531      2541      2551      2561                  |         |         |         |         |         |         |HB8:HPB8_627      MVRFIGNNTLSYRDGGRYNTFASIITGGEIRLFKTFYVNAGIGARFGLDYKDINITGNIGMRYAFHSJM:HPSJM_04695  MVRFIGNNTLSYRDGGRYNTFASIITGGELRLFKTFYVNAGIGARFGLDYKDINITGNIGMRYAFHP12:HPP12_0919   MVRFIGNNTLSYRDGGRYNTFASIITGGEIRLFKTFYVNAGIGARFGLDYKDINITGNIGMRYAFHHPA:HPAG1_0903   MVRFIGNNTLSYRDGGRYNTFASIITGGEIRLFKTFYVNAGIGARFGLDYKDINITGNIGMRYAFH266:HP0922       MVRFIGNNTLSYRDGGRYNTFASIITGGEIRLFKTFYVNAGIGARFGLDYKDINITGNIGMRYAFHG27:HPG27_871    MVRFIGNNTLSYRDGGRYNTFASIITGGEIRLFKTFYVNAGIGARFGLDYKDINITGNIGMRYAFHB38:mHELPY_0906  MVRFIGNNTLSYRDGGRYNTFASIITGGEIRLFKTFYVNAGIGARFGLDYKDINITGNIGMRYAFHF32:HPF32_0434   MVRFIGNNTLSYRDGGRYNTFASIITGGEIRLFKTFYVNAGIGARFGLDYKDINITGNIGMRYAFHF57:HPF57_0931   MVRFIGNNTLSYRDGGRYNTFASIITGGEIRLFKTFYVNAGIGARFGLDYKDINITGNIGMRYAFH51:KHP_0860      MVRFIGNNTLSYRDGGRYNTFASIITGGEIRLFKTFYVNAGIGARFGLDYKDVNITGNIGMRYAFHF16:HPF16_0901   MVRFIGNNTLSYRDGGRYNTFASIITGGEIRLFKTFYVNAGIGARFGLDYKDINITGNIGMRYAFH52:HPKB_0890     MVRFIGNNTLSYRDGGRYNTFASIITGGEIRLFKTFYVNAGIGARFGLDYKDINITGNIGMRYAFHF30:HPF30_0419   MVRFIGNNTLSYRDGGRYNTFASIITGGEIRLFKTFYVNAGIGARFGLDYKDINITGNIGMRYAF
