# Supplementary material for: Evolution in an oncogenic bacterial species with extreme genome plasticity: Helicobacter pylori East Asian genomes
Source: BMC Microbiol. 2011 May 16;11:104. doi: 10.1186/1471-2180-11-104 (PMC3120642; doi:10.1186/1471-2180-11-104)
Supplement: Additional file 6 — Multiple sequence alignments of diverged genes. [file 1471-2180-11-104-S6.ZIP › Diverged_genes_multiple_seuence_alignments/HP0978_ftsA.mfa.rtf]

                  1         11        21        31        41        51        61        71        81        91                          |         |         |         |         |         |         |         |         |         |         HB8:HPB8_567      MEHKEIVIGVDIGSRKICAIVAEFKEGILRIIGTAHQDSKEINSKAIKRGRINSLAHASNAIKEVINSAKKMAGLNADEDRNNPISSFRESYYPKTKAIVHSJM:HPSJM_04995  MEHKEIVIGVDIGSRKICAIVAEFKEGILRIIGTAHQDSKEINSKAIKRGRINSLAHASNAIKEVINSAKKMAGLNADEDRNNPISSFRESYYPKTKAIVHB38:HELPY_0965   MEHKEIVIGVDIGSRKICAIVAEFKEGILRIIGTAHQDSKEINSKAIKRGRINSLAHASNAIKEVINSAKKMAGLNADEDRNNPISSFRESYYPKTKAIVHG27:mHPG27_925   MEHKEIVIGVDIGSRKICAIVAEFKEGILRIIGTAHQDSKEINSKAIKRGRINSLAHASNAIKEVINSAKKMAGLNADEDRNNPISSFRESYYPKTKAIVH266:HP0978       MEHKEIVIGVDLGSRKICAIVAEFKEGILRIIGTAHQDSKEINSKAIKRGRINSLAHASNAIKEVINSAKKMAGLNADEDRNNPMPHFGE-YHPKTKAIVHHPA:HPAG1_0959   MEHKEIVIGVDIGSRKICAIVAEFKEGILRIIGTAHQDSKEINSKAIKRGRINSLAHASNAIKEVINSAKKMAGLNADEDRNNPMPHFGE-YRPKTKAIVHP12:HPP12_0974   MEHKEIVIGVDLGSRKICAIVAEFKEGILRIIGTAHQDSKEINSKAIKRGRINSLAHASNAIKEVINSAKKMAGLNADEDRNNPMPHFGE-YHPKTKAIVHF32:HPF32_0383   MENKEIVIGVDLGSRKICAIVAEFKEGILRIIGTAHQDSKEINSKAIKRGRINSLAHASNAIKEVINSAKKMAGLNADEDRNNPISSFRESYYPKTKAIVHF57:HPF57_0987   MEHKEIVIGVDIGSRKICAIVAEFKEGILRIIGTAHQDSKEINSKAIKRGRINSLAHASNAIKEVINSAKKMAGLNADEDRNNPISSFRESYYPKTKAIVH52:HPKB_0947     MEHKEIVIGVDIGSRKICAIVAEFKEGILRIIGTAHQDSKEINSKAIKRGRINSLAHASNAIKEVINSAKKMAGLNADEDRNNPISSFRESYYPKTKAIVHF30:HPF30_0363   MEHKEIVIGVDIGSRKICAIVAEFKEGILRIIGTAHQDSKEINSKAIKRGRINSLAHASNAIKEVINSAKKMAGLNADEDRNNPISSFRESYYPKTKAIVH51:KHP_0911      MEHKEIVIGVDIGSRKICAIVAEFKEGILRIIGTAHQDSKEINSKAIKRGRINSLAHASNAIKEVINSAKKMAGLNADEDRNNPISSFRESYYPKTKAIVHF16:HPF16_0961   MEHKEIVIGVDIGSRKICAIVAEFKEGILRIIGTAHQDSKEINSKAIKRGRINSLAHASNAIKEVINSAKKMAGLNADEDRNNPISSFRESYYPKTKAIV                  101       111       121       131       141       151       161       171       181       191                         |         |         |         |         |         |         |         |         |         |         HB8:HPB8_567      SFSGAYTESIRDITGVASTKDNVVTIDEINRAINNACAKAGLDNDKHILHALPYRFTLDKQEVNDPLGMSGTRLEVFIHIVYTEKNNIENLEKIMIQSGVHSJM:HPSJM_04995  SFSGAYTESIRDVTGVASTKDNVVTIDEINRAINNACAKAGLDNDKHILHALPYRFTLDKQEVNDPLGMSGTRLEVFIHIVYTEKNNIENLEKIMIQSGVHB38:HELPY_0965   SFSGAYTESIRDVTGVASTKDNVVTIDEINRAINNACAKAGLDNDKHILHALPYRFTLDKQEVNDPLGMSGTRLEVFIHIVYTEKNNIENLEKIMIQSGVHG27:mHPG27_925   SFSGAYTESIRDVTGVASTKDNVVTIDEINRAINNACAKAGLDNDKHILHALPYRFTLDKQEVNDPLGMSGTRLEVFIHIVYTEKNNIENLEKIMIQSGVH266:HP0978       SFSGAYTESIRDVTGVASTKDNVVTIDEINRAINSACAKAGLDNDKHILHALPYRFTLDKQEVNDPLGMSGTRLEVFIHIVYTEKNNIENLEKIMIQSGVHHPA:HPAG1_0959   SFSGAYTESIRDVTGVASTKDNVVTIDEINRAINNACAKAGLDNDKHILHALPYRFTLDKQEVNDPLGMSGTRLEVFIHIVYTEKNNIENLEKIMIQSGVHP12:HPP12_0974   SFSGAYTESIRDVTGVASTKDNVVTIDEINRAINNACAKAGLDNDKHILHALPYRFTLDKQEVNDPLGMSGTRLEVFIHIVYTEKNNIENLEKIMIQSGVHF32:HPF32_0383   SFSGAYTESIRDVTGVASTKDNVVTIDEINRAINNACAKAGLDNDKHILHALPYRFTLDKQEVNDPLGMSGTRLEVFIHIVYTEKNNIENLEKIMIQSGVHF57:HPF57_0987   SFSGAYTESIRDVTGVASTKDNVVTIDEINRAINNACAKAGLDNDKHILHALPYRFTLDKQEVNDPLGMSGTRLEVFIHIVYTEKNNIENLEKIMIQSGVH52:HPKB_0947     SFSGAYTESIRDVTGVASTKDNVVTIDEINRAINNACAKAGLDNDKHILHALPYRFTLDKQEVNDPLGMSGTRLEVFIHIVYTEKNNIENLEKIMIQSGVHF30:HPF30_0363   SFSGAYTESIRDVTGVASTKDNVVTIDEINRAINNACAKAGLDNDKHILHALPYRFTLDKQEVNDPLGMSGTRLEVFIHIVYTEKNNIENLEKIMIQSGVH51:KHP_0911      SFSGAYTESIRDVTGVASTKDNVVTIDEINRAINNACAKAGLDNDKHILHALPYRFTLDKQEVNDPLGMSGTRLEVFIHIVYTEKNNIENLEKIMIQSGVHF16:HPF16_0961   SFSGAYTESIRDVTGVASTKDNVVTIDEINRAINNACAKAGLDNDKHILHALPYRFTLDKQEVNDPLGMSGTRLEVFIHIVYTEKNNIENLEKIMIQSGV                  201       211       221       231       241       251       261       271       281       291                         |         |         |         |         |         |         |         |         |         |         HB8:HPB8_567      EIENIVINSYAASIATLSNDERELGVACVDMGGETCNLTIYSGNSIRYNKYLPVGSHHLTTDLSHMLNIPFPYAEEVKIKYGDLSFKYDTETPSQNVQIPHSJM:HPSJM_04995  EIENIVINSYAASIATLSNDERELGVACVDMGGETCNLTIYSGNSIRYNKYLPVGSHHLTTDLSHMLNTPFPYAEEVKIKYGDLSFESGAETPSQSVQIPHB38:HELPY_0965   EIENIVINSYAASIATLSNDERELGVACVDMGGETCNLTIYSGNSIRYNKYLPVGSHHLTTDLSHMLNTPFPYAEEVKIKYGDLSFEGGTETPSQSVQIPHG27:mHPG27_925   EIENIVINSYAASIATLSNDERELGVACVDMGGETCNLTIYSGNSIRYNKYLPVGSHHLTTDLSHMLNTPFPYAEEVKIKYGDLSFEGGTETPSQNVQIPH266:HP0978       EIENIVINSYAASIATLSNDERELGVACVDMGGETCNLTIYSGNSIRYNKYLPVGSHHLTTDLSHMLNTPFPYAEEVKIKYGDLSFEGGEETPSQNVQIPHHPA:HPAG1_0959   EIENIVINSYAASIATLSNDERELGVACVDMGGETCNLTIYSGNSIRYNKYLPVGSHHLTTDLSHMLNTPFPYAEEVKIKYGDLSFEGGTETPSQNVQIPHP12:HPP12_0974   EIENIVINSYAASIATLSNDERELGVACVDMGGETCNLTIYSGNSIRYNKYLPVGSHHLSTDLSHMLNTPFPYAEEVKIKYGDLSFESGEETPSQNVQMPHF32:HPF32_0383   EIENIVINSYAASIATLSNDERELGVACVDIGGETCNLTIYSGNSIRYNKYLPIGSHHLSTDLSSMLNTPFPYAEEVKIKYGDLSFESGEETPSQSVQIPHF57:HPF57_0987   EIENIVINSYAASIATLSNDERELGVACVDIGGETCNLTIYSGNSIRYNKYLPIGSHHLSTDLSSMLNTPFPYAEEVKIKYGDLSFESGEETPSQSVQIPH52:HPKB_0947     EIENIVINSYAASIATLSNDERELGVACVDIGGETCNLTIYSGNSIRYNKYLPIGSHHLSTDLSSMLNTPFPYAEEVKIKYGDLSFESGEETPSQSVQIPHF30:HPF30_0363   EIENIVINSYAASIATLSNDERELGVACVDIGGETCNLTIYSGNSIRYNKYLPIGSHHLSTDLSSMLNTPFPYAEEVKIKYGDLSFESGEETPSQSVQIPH51:KHP_0911      EIENIVINSYAASIATLSNDERELGVACVDIGGETCNLTIYSGNSIRYNKYLPIGSHHLSTDLSSMLNTPFPYAEEVKIKYGDLSFESGEETPSQSVQIPHF16:HPF16_0961   EIENIVINSYAASIATLSNDERELGVACVDIGGETCNLTIYSGNSIRYNKYLPIGSHHLSTDLSSMLNTPFPYAEEVKIKYGDLSFESGEETPSQSVQIP                  301       311       321       331       341       351       361       371       381       391                         |         |         |         |         |         |         |         |         |         |         HB8:HPB8_567      TTGSDGHESHIVPLSEIQTIMRERALETFKIIHRSIQDSGLEEHLGGGVVLTGGMALMKGIKELARTHFTNYPVRLAAPVEKYNIMGMFEDLKDPRFSVVHSJM:HPSJM_04995  TTGSDGHESHIVPLSEIQTIMRERALETFKIIHRSIQDSGLEEHLGGGVVLTGGMALMKGIKELARTHFTNYPVRLATPVEKYNIMGMFEDLKDPRFSVVHB38:HELPY_0965   TTGSDGHESHIVPLSEIQTIMRERALETFKIIHRSIQDSGLEEHLGGGVVLTGGMALMKGIKELARTHFTNYPVRLAAPVEKYNIMGMFEDLKDPRFSVVHG27:mHPG27_925   TTGSDGHESHIVPLSEIQTIMRERALETFKIIHRSIQDSGLEEHLGGGVVLTGGMALMKGIKELARTHFTNYPVRLAAPVEKYNIMGMFEDLKDPRFSVVH266:HP0978       TTGSDGHESHIVPLSEIQTIMRERALETFKIIHRSIQDSGLEEHLGGGVVLTGGMALMKGIKELARTHFTNYPVRLAAPVEKYNIMGMFEDLKDPRFSVVHHPA:HPAG1_0959   TTGSDGHESHIVPLSEIQTIMRERALETFEIIHRSIQDSGLEEHLGGGVVLTGGMALMKGIKELAKAHFTNYPVRLAAPMEKYNIMGMFEDLKDPRFSVVHP12:HPP12_0974   TTGSDGHESHIVPLNKIQTIMRERALETFEIIHRSIQDSGLEEHLGGGVVLTGGMALMKGIKELAKAHFTNYPVRLAAPMEKYNIMGMFEDLKDPRFSVVHF32:HPF32_0383   TTGSDGNESHVVPLSKIQNIMRDRALETFQIIHRSIQDSGLEEHLGGGVVLTGGMALMKGIKELAKAHFTNYPVRLAAPMEKYNIMGMFEDLKDPRFSVVHF57:HPF57_0987   TTGSDGNESHVVPLSKIQNIMRDRALETFQIIHRSIQDSGLEEHLGGGVVLTGGMALMKGIKELAKAHFTNYPVRLAAPMEKYNIMGMFEDLKDPRFSVVH52:HPKB_0947     TTGSDGNESHVVPLSRIQNIMRDRALETFQIIHRSIQDSGLEEHLGGGVVLTGGMALMKGIKELAKAHFTNYPVRLAAPMEKYNIMGMFEDLKDPRFSVVHF30:HPF30_0363   TTGSDGNESHVVPLSKIQNIMRDRALETFQIIHRSIQDSGLEEHLGGGVVLTGGMALMKGIKELAKAHFTNYPVRLAAPMEKYNIMGMFEDLKDPRFSVVH51:KHP_0911      TTGSDGNESHVVPLSRIQNIMRDRALETFQIIHRSIQDSGLEEHLGGGVVLTGGMALMKGIKELAKAHFTNYPVRLAAPMEKYNIMGMFEDLKDPRFSVVHF16:HPF16_0961   TTGSDGNESHVVPLSRIQNIMRDRALETFQIIHRSIQDSGLEEHLGGGVVLTGGMTLMKGIKELAKAHFTNYPVRLAAPMEKYNIMGMFEDLKDPRFSVV                  401       411       421       431       441       451       461       471       481       491                  |         |         |         |         |         |         |         |         |         |HB8:HPB8_567      VGLILYKAGGHTNYERDSKGVIRYHESDDYTRTAHQSSPTPHIHSSPTERNLSDLKTPNTPLNTAKNDDFLPIKPTEQKGFFKSFLDKISKIFHSJM:HPSJM_04995  VGLILYKAGGHTNYERDSKGIIRYHESDDYTRTAHQSSPTPHIHSSPTERNLSDLKTPNAPLNTAKNDDFLPIKPTEQKGFFQNLLDKISKIFHB38:HELPY_0965   VGLILYKAGGHTNYERDSKGVIRYHESDDYTRTAHQSSPTPHIHSSPTERNLSDLKTPNAPLNTAKNDDFLPIKPTEQKGFFKSFLDKISKLFHG27:mHPG27_925   VGLILYKAGGHTNYERDSKGVIRYHESDDYTRTAHQSSPTPHIHSSPTERNLSDLKAPSTPLNTAKNDDFLPIKPTEQKGFFKSFLDKISKIFH266:HP0978       VGLILYKAGGHTNYERDSKGVIRYHESDDYTRTAHQSSPTPHIHSSPTERNLSDLKAPSAPLNTAKNDDFLPIKPTEQKGFFKSFLDKISKFFHHPA:HPAG1_0959   VGLILYKAGGHTNYERDSKGIIRYHESDDYTRTAHQSSPTPHIHSSPTERNLSDLKAPSAPLNTAKNDDFLPIKPTEQKGFFKSFLDKISKIFHP12:HPP12_0974   VGLILYKAGGHTNYERDSKGIIRYHESDDYTRTTHQSSPTPHIHSSPTERNLSDLKTPSTPLNTAKNDDFLPIKPTEQKGFFKSFLDKISKIFHF32:HPF32_0383   VGLILYKAGGHTNYERDSKGIIRYHEIDDYTRTAHQSSPTPHIHSSPTERNLSDLKTPSAPSNTAKNDDFLPIKPTEQKGFFKSFLDKISKIFHF57:HPF57_0987   VGLILYKAGGHTNYERDSKGIIRYHEIDDYTRTAHQSSPTPHIHSSPTERNLSDLKAPSAPSNTAKNDDFLPVKPTEQKGFFKSFFDKISKIFH52:HPKB_0947     VGLILYKAGGHTNYERDSKGIIRYHEIDDYTRIAHQSSPTPHIHSSPTERNLSDLKAPSAPLNTAKNDDFLPVKPTEQKGFFKSFLDKISKIFHF30:HPF30_0363   VGLILYKAGGHTNYERDSKGIIRYHEIDDYTRTAHQSSPTPHIHSSPTERNLSDLKAPSTPLNTAKNDDFLPVKPTEQKGFFKSFLDKISKIFH51:KHP_0911      VGLILYKAGGHTNYERDSKGIIRYHEIDDYTRTAHQSSPTPHIHSSPTERNLSDLKTPSVPSNTAKNDDFLPVKPTEQKGFFKSFLDKISRIFHF16:HPF16_0961   VGLILYKAGGHTNYERDSKGIIRYHKIDDYTRTAYQSSPTPHIHSSPTERNLSDLKALSAPSNTAKNDDFLPIKPTEQKGFFKSFLDKISKIF
