# Supplementary material for: Evolution in an oncogenic bacterial species with extreme genome plasticity: Helicobacter pylori East Asian genomes
Source: BMC Microbiol. 2011 May 16;11:104. doi: 10.1186/1471-2180-11-104 (PMC3120642; doi:10.1186/1471-2180-11-104)
Supplement: Additional file 6 — Multiple sequence alignments of diverged genes. [file 1471-2180-11-104-S6.ZIP › Diverged_genes_multiple_seuence_alignments/HP1067_cheY.mfa.rtf]

                  1         11        21        31        41        51        61        71        81        91                          |         |         |         |         |         |         |         |         |         |         HB8:HPB8_1186     LKLLVVDDSSTMRRIIKNTLSRLGYEDVLEAEHGVEAWEKLDANADTKVLITDWNMPEMNGLDLVKKVRSDSRFKEIPIIMITTEGGKAEVITALKAGVNHSJM:HPSJM_02010  LKLLVVDDSSTMRRIIKNTLSRLGYEDVLEAEHGVEAWEKLDANADTKVLITDWNMPEMNGLDLVKKVRSDSRFKEIPIIMITTEGGKAEVITALKAGVNH266:HP1067       LKLLVVDDSSTMRRIIKNTLSRLGYEDVLEAEHGVEAWEKLDANADTKVLITDWNMPEMNGLDLVKKVRSDSRFKEIPIIMITTEGGKAEVITALKAGVNHB38:HELPY_0383   LKLLVVDDSSTMRRIIKNTLSRLGYEDVLEAEHGVEAWEKLDANADTKVLITDWNMPEMNGLDLVKKVRSDSRFKEIPIIMITTEGGKAEVITALKAGVNHHPA:HPAG1_0380   LKLLVVDDSSTMRRIIKNTLSRLGYEDVLEAEHGVEAWEKLDANADTKVLITDWNMPEMNGLDLVKKVRSDSRFKEIPIIMITTEGGKAEVITALKAGVNHG27:HPG27_361    LKLLVVDDSSTMRRIIKNTLSRLGYEDVLEAEHGVEAWEKLDANADTKVLITDWNMPEMNGLDLVKKVRSDSRFKEIPIIMITTEGGKAEVITALKAGVNHP12:HPP12_0377   LKLLVVDDSSTMRRIIKNTLSRLGYEDVLEAEHGVEAWEKLDANADTKVLITDWNMPEMNGLDLVKKVRADSRFKEIPIIMITTEGGKAEVITALKAGVNHF32:HPF32_0928   LKLLVVDDSSTMRRIIRNTLSRLGYEDVLEAEHGVEAWEKLNANADTKVLITDWNMPEMNGLDLVIKVRADERFKEIPIIMITTEGGKAEVITALKAGVNHF57:HPF57_0432   LKLLVVDDSSTMRRIIKNTLSRLGYEDVLEAEHGVEAWEKLNANADTKVLITDWNMPEMNGLDLVIKVRADERFKEIPIIMITTEGGKAEVITALKAGVNHF16:HPF16_0384   LKLLVVDDSSTMRRIIKNTLSRLGYEDVLEAEHGVEAWEKLNANADTKVLITDWNMPEMNGLDLVIKVRADERFKEIPIIMITTEGGKAEVITALKAGVNH51:KHP_0368      LKLLVVDDSSTMRRIIKNTLSRLGYEDVLEAEHGVEAWEKLNANADTKVLITDWNMPEMNGLDLVIKVRADERFKEIPIIMITTEGGKAEVITALKAGVNH52:HPKB_0384     LKLLVVDDSSTMRRIIKNTLSRLGYEDVLEAEHGVEAWEKLNANADTKVLITDWNMPEMNGLDLVIKVRADERFKEIPIIMITTEGGKAEVITALKAGVNHF30:HPF30_0917   LKLLVVDDSSTMRRIIKNTLSRLGYEDVLEAEHGVEAWEKLNANADTKVLITDWNMPEMNGLDLVIKVRADERFKEIPIIMITTEGGKAEVITALKAGVN                  101       111       121                  |         |         |HB8:HPB8_1186     NYIVKPFTPQVLKEKLEVVLGTNDHSJM:HPSJM_02010  NYIVKPFTPQVLKEKLEVVLGTNDH266:HP1067       NYIVKPFTPQVLKEKLEVVLGTNDHB38:HELPY_0383   NYIVKPFTPQVLKEKLEVVLGTNDHHPA:HPAG1_0380   NYIVKPFTPQVLKEKLEVVLGTNDHG27:HPG27_361    NYIVKPFTPQVLKEKLEVVLGTNDHP12:HPP12_0377   NYIVKPFTPQVLKEKLEVVLGTNDHF32:HPF32_0928   NYIVKPFTPQVLKEKLEVVLGTNDHF57:HPF57_0432   NYIVKPFTPQVLKEKLEVVLGTNDHF16:HPF16_0384   NYIVKPFTPQVLKEKLEVVLGTNDH51:KHP_0368      NYIVKPFTPQVLKEKLEVVLGTNDH52:HPKB_0384     NYIVKPFTPQVLKEKLEVVLGTNDHF30:HPF30_0917   NYIVKPFTPQVLKEKLEVVLGTND
