# Supplementary material for: Evolution in an oncogenic bacterial species with extreme genome plasticity: Helicobacter pylori East Asian genomes
Source: BMC Microbiol. 2011 May 16;11:104. doi: 10.1186/1471-2180-11-104 (PMC3120642; doi:10.1186/1471-2180-11-104)
Supplement: Additional file 6 — Multiple sequence alignments of diverged genes. [file 1471-2180-11-104-S6.ZIP › Diverged_genes_multiple_seuence_alignments/HP1068_prmA.mfa.rtf]

                   1         11        21        31        41        51        61        71        81        91                           |         |         |         |         |         |         |         |         |         |         HB8:HPB8_1187      VLKPMYYEFFFIFPKERELFESFLLDTTHLALEESSLEDLKAFDDKETIEFISQSSWHYFATHDPLKKDLKEHLKEKPPYLKNFVILRSEENLNNSLILAHHPA:HPAG1_0379    VLKPMYYEFFFIFPKERELFESFLLDTTHLALEESSLENLKAFDDKETIEFISQSSWHYFATHDPLKEH----LKEKPQHLKNFVILRSQKDLNNSLILAH266:HP1068        VLKPMYYEFFFIFPKERELFESFLLDATHLALEESSLENLKAFDDKETIGFISQSNWHYFATHDPLKKDLKENLKEKPPHLKNFVILRSQKDLNNSLIPAHSJM:mHPSJM_02005  ----MYYEFFFIFPKERELFESFLLDTTHLALEESSLENLKAFDDKETIEFISQSSWRYFATHDPLKED----LKEKPPYLKNFVILRSEKNLSDSLFPAHG27:HPG27_360     ----MYYEFFFIFPKERELFESFLLDATHLALEESSLESLKAFDDKETIEFISQSSWHHFATHDPLKKD----LKEKPPYLKNFVILRSEKNLSDSLFPAHB38:mHELPY_0382   ----MYYEFFFIFPKERELFESFLLDATHLALEESSLENLKAFDDKETIEFISQSNWHYFATHDPLKKD----LKEKPPHLKNFVILRSEEDLNNSLIPAHF32:HPF32_0929    VLKPMYYEFFFIFPKERELFESFLLDTTHLALEESSLENLKAFDDKETIEFISQSGWHYFTTHDPLKEN----LKEKPPHLKNFVILRSQKDLNDSLISAHF57:HPF57_0431    VLKPMYYEFFFIFPKEQELFESFLLDTTHLALEESSLENLKAFDDKETIEFISQSSWHYFTTHDPLKEN----LKEKPPHLKNFVILRSQKDLNDSLIPAHF16:HPF16_0383    VLKPMYYEFFFIFPKERELFESFLLDTTRLALEESSLENLKAFDDKETIEFISQSSWHYFTTHDPLKEN----LKEKPPHLKNFIILRSQKDLNDSLIPAHF30:HPF30_0918    VLKPMYYEFFFIFPKERELFESFLLDTTHLALEESSLENLKAFDDKETIEFISQSSWHYFTTHDPLKEN----LKEKPPHLKNFVILRSQKDLNDSLIPAH51:mKHP_0367      ----MYYEFFFIFPKERELFESFLLDTTHLALEESSLENLKAFDDKETIEFISQSSWHYFTTHDPLKEN----LKEKPPHLKNFIILRSQKDLNDSLIPAH52:mHPKB_0383     ----MYYEFFFIFPKERELFESFLLDTTHLALEESSLENLKAFDDKETIEFISQSSWHYFTTHDPLKEN----LKEKPPHLKNFIILRSQKDLNDSLIPTHP12:HPP12_0376    VLKPMYYEFFFIFPKKRELFESFLLDTTHLALEESSLENLKAFDDEETIDFISQSSWRYFATHDLLKEH----LKEKSPHLKNFVILRSEKDLNDSLIPA                   101       111       121       131       141       151       161       171       181       191                          |         |         |         |         |         |         |         |         |         |         HB8:HPB8_1187      LEAFCLNLKQNLQNEFDFFYLSRNLASKDWLEAYKQAILPVQCAKFYIHPSWHQKPSHVVTNDCIMIDPALAFGSGHHESTSMCLELLSNLDLKRKNALDHHPA:HPAG1_0379    LEAFCLNLKQNLQSEFDFFYLSRNLASKDWLEAYKQAILPVQCAKFYIHPSWHQKPSHISTDDCIMIDPALAFGSGHHESTSMCLELLSDLDLKRKNALDH266:HP1068        LEAFCLNLKQNLQSEFDFFYLSRNLASKDWLEAYKQAILPVQCTKFYIHPSWHQKPSHVVTNDCIMIDPALAFGSGHHESTSMCLELLSDIDLKRKNALDHSJM:mHPSJM_02005  LEAFCLNLKQNLQSEFDFFYLSRNLASKDWLEAYKQAILPVQCAKFYIHPSWHQKPSHVVTNDCIMIDPALAFGSGHHESTSMCLELLSDLDLKRKNALDHG27:HPG27_360     LEAFCLNLKQNLQSEFDFFYLSRNLASKDWLEAYKQAILPVQCAKFYIHPSWHQKPSHVATNDSIMIDPALAFGSGHHESTSMCLELLSNLDLKRKNALDHB38:mHELPY_0382   LEAFCLSLKQNLQSEFGFFYLSRNLASKDWLEAYKQAILPVQCAKFYIHPSWHQKPSHVVTNDCIMIDPALAFGSGHHESTSMCLELLSNLDLKRKNALDHF32:HPF32_0929    LEAFCLSLQQNLQSGFDFFYLSRNLASKDWLEAYKQAILPVQCAKFYIHPSWHQKPSHIAIDDSIMIDPALAFGSGHHESTSMCLELLSNLDLKRKNALDHF57:HPF57_0431    LEAFCLSLQQNLQSGFDFFYLSRNLASKNWLEAYKQAILPVQCAKFYIHPSWHQKPSHIATDDSIMIDPALAFGSGHHESTSMCLELLSNLDLKRKNALDHF16:HPF16_0383    LEAFCLSLQQNLQSGFDFFYLSRNLASKDWLEAYKQAILPVQCTKFYIHPSWHQKPSHVAIDDSIMIDPALAFGSGHHESTSMCLELLSNLDLKRKNALDHF30:HPF30_0918    LEAFCLSLQQNLQSGFDFFYLSRNLASKDWLEAYKQAILPVQCTKFYIHPSWHQKPSHVATDDSIMIDPALAFGSGHHESTSMCLELLSNLDLKRKNALDH51:mKHP_0367      LEAFCLSLQQNLQSGFDFFYLSRNLASKDWLEAYKQAILPVQCAKFYIHPSWHQKPSHVATDDSIMINPALAFGSGHHESTSMCLELLSNLDLKRKNALDH52:mHPKB_0383     LEAFCLSLQQNLQSGFDFFYLSRNLASKDWLEAYKQAILPVQCAKFYIHPSWHQKPSHVAIDDSIMIDPALAFGSGHHESTSMCLELLSNLDLKHKNALDHP12:HPP12_0376    LEAFCLSLQQNLQSEFDFFYLSRNLASKDWLEAYKQAILPVQCTKFYIHPSWHQKPSHVATNDCIMIDPALAFGSGHHESTSMCLELLSNLDLKRKNALD                   201       211       221       231       241       251       261       271       281       291                          |         |         |         |         |         |         |         |         |         |         HB8:HPB8_1187      VGCGSGILSIALKKQGVSALVACDTDSLAVEETLKNFSLNQIPLLAQDKVIYGSTQKIEGRFDVIVANLVADVIKSLYSEFVRLCNHTLILSGILETHLNHHPA:HPAG1_0379    VGCGSGILSIALKKQGVSALVACDTDSLAVEETLKNFSLNQIPLLAQDKVIYGSTQKIEGRFDIIVANLVADVVKSLYSEFVRLCNHTLILSGILETHLNH266:HP1068        VGCGSGILSIALKKQGVSALVACDTDSLAVEETLKNFSLNQIPLLVQDKVIYGSTQKIEGRFDVIVANLVADVIKSLYSEFVRLCNHTLILSGILETHLNHSJM:mHPSJM_02005  VGCGSGILSIALKKQGVSALVACDTDSLAVEETLKNFSLNQIPLLAQDKVIYGSTQKIEGRFDVIVANLVADVIKSLYSEFVRLCNHTLILSGILETHLNHG27:HPG27_360     VGCGSGILSIALKKQGVSALVACDTDSLAVEETLKNFSLNQIPLLAQDKVIYGSTQKIEGRFDVIVANLVADVIKSLYSEFVRLCNHTLILSGILETHLNHB38:mHELPY_0382   VGCGSGILSIALKKQGVSALAACDTDSLAVEETLKNFSLNQIPLLAQDKVIHGSTQKIEGHFDIIVANLVADVIKSLYSEFVRLCNHTLILSGILETHLNHF32:HPF32_0929    VGCGSGILSIALKKQGVSTLNACDTDSLAVEETLKNFSLNQIPLLAQDKVIHGSTQKIKGRFDIIVANIVADVIKSLYSEFVRLCNHTLILSGILETHLNHF57:HPF57_0431    VGCGSGILSIALKKQGVSALSACDTDSLAVEETLKNFVLNQIPLLVQDKVIHGSTQKIKGRFDIIVANIVADVIKSLYSEFVRLCNHTLILSGILETHLNHF16:HPF16_0383    VGCGSGILSIALKKQGVSALSACDTDSLAVEETLKNFSLNQIPLLVQDKVIHGSTQKIKGRFDIIVANIVADVIKSLYSEFVRLCNHTLILSGILETHLNHF30:HPF30_0918    VGCGSGILSIALKKQGVSALSACDTDSLAVEETLKNFSLNQIPLLAQDKVIHGSTQKIKGRFDIIVANIVADVIKSLYSEFVRLCNHTLILSGILETHLNH51:mKHP_0367      VGCGSGILSIALKKQGVSALSACDTDSLAVEETLKNFSLNQIPLLAQDKVIHGSTQKIKGRFDIIVANIVADVIKSLYSEFVRLCNHTLILSGILETHLNH52:mHPKB_0383     VGCGSGILSIALKKQGVSALSACDTDSLAVEETLKNFSLNQIPLLAQDKVIHGSTQKIKGRFDIIVANIVADVIKSLYSEFVRLCNHTLILSGILETHLNHP12:HPP12_0376    VGCGSGILSIALKKQGVSALVACDTDSLAVEETLKNFSLNQIPLLAQDKVICGSTQKIEGRFDIIVANLVADVIKSLYSEFVRLCNHTLILSGILETHLN                   301       311       321       331                   |         |         |         |HB8:HPB8_1187      SVLQIYYNGFEILEQRQRNEWVALKLLKKQPINHHPA:HPAG1_0379    SVLQIYYNGFEILEQRQRNEWVALKLLKKQPINH266:HP1068        SVLQIYYNGFEVLEQRQRNEWVALKLLKKQPINHSJM:mHPSJM_02005  SVLQIYYNGFEVLEQRQRNEWVALKLLRKQPINHG27:HPG27_360     SVLQIYYNGFEVLEQRQRNEWVALKLLKKQPINHB38:mHELPY_0382   SVLQIYYNGFEVLEQRQRNEWVALKLLKKQSINHF32:HPF32_0929    SVLQIYYNGFEVLEQRQRNEWIALKLLKKQSINHF57:HPF57_0431    SVLQIYYNGFEVLEQRQRNEWVALKLLKKQSINHF16:HPF16_0383    SVLQIYYNGFEVLEQRQRNEWVALKLLKKQSINHF30:HPF30_0918    SVLQIYYNGFEVLEQRQRNEWVALKLLKKQSINH51:mKHP_0367      SVLQIYYNGFEVLEQRQRNEWVALKLLKKQSINH52:mHPKB_0383     SVLQIYYNGFEVLEQRQRNEWVALKLLKKQSINHP12:HPP12_0376    SVLQIYYNGFEVLEQRQRNEWVALKLLKKQPIN
