# Supplementary material for: Evolution in an oncogenic bacterial species with extreme genome plasticity: Helicobacter pylori East Asian genomes
Source: BMC Microbiol. 2011 May 16;11:104. doi: 10.1186/1471-2180-11-104 (PMC3120642; doi:10.1186/1471-2180-11-104)
Supplement: Additional file 6 — Multiple sequence alignments of diverged genes. [file 1471-2180-11-104-S6.ZIP › Diverged_genes_multiple_seuence_alignments/HP1102_pgl.mfa.rtf]

                  1         11        21        31        41        51        61        71        81        91                          |         |         |         |         |         |         |         |         |         |         HB8:HPB8_402      MGYQLFEFESLKDCHKALIERFKEFFNAALKKHHQVSIAFSGGRSPIGLLQKLSVLDLKWHECLISLVDERIVDTSHKDSNTKLLHDYLLQNNALKASFTHB38:HELPY_1071   MGYQLFEFESLKDCHKALTERFKEFFNTALKKHHQVSIAFSGGRSPIGLLQKLSVLDLKWHECLISLVDERIIDTSHDDSNTKLLHDYLLQNNALKASFTHHPA:HPAG1_1040   MGYQLFEFENLKDCHKALIERFKEFFNTALKKHHQVSIAFSGGRSPISLLQKLSVLDLKWHECLISLVDERIIDTSHKDSNTKLLHDYLLQNNALKASFTHP12:HPP12_1067   MGYQLFEFENLKDCHKALTERFKEFFNTALKKHHQVSIAFSGGRSPIGLLQKLSVLDLKWHECLISLVDERIIDTSHKDSNTKLLHDYLLQNNALKASFIHG27:HPG27_1043   MGYQLFEFENLKDCHKALIERFKEFFNATLKKHHQVSIAFSGGRSPIGLLQKLSVLDLKWHECLISLVDERIIDTSHDDSNTKLLHDYLLQNNALKASFTHF32:HPF32_1038   MGYQLFEFENLENCHKALTERFKEFFNAALKKHHQVSIAFSGGRSPIGLLQKLSVLDLKWHACLVSLVDERIIDTNHDDSNTKLLHDYLLQNNALKASFIHF16:HPF16_1044   MGYQLFEFENLENCHKALTERFKEFFNAALKKHHQVSIAFSGGRSPISLLQKLSVLDLKWHACLVSLVDERIIDTNHDDSNTKLLHDYLLQNNALKASFIH52:HPKB_1032     MGYQLFEFENLENCHKALTERFKEFFNAALKKHHQVSIAFSGGRSPISLLQKLSVLDLKWHACLVSLVDERIIDTNHDDSNTKLLHDYLLQNNALKASFIHF57:HPF57_1064   MGYQLFEFENLKDCHKALTERFKEFFNVALKKHHQVSIAFSGGRSPISLLQKLSVLDLKWHACLVSLVDERIIDTNHDDSNTKLLHDYLLQNNALKASFIHF30:HPF30_0287   MGYQLFEFENLKDCHKALTERFKEFFNAALKKHHQVSIAFSGGRSPISLLQKLSVLDLKWHACLVSLVDERIIDTNHDDSNTKLLHDYLLQNNALKASFIH51:KHP_1002      MGYQLFEFENLKDCHKALTERFKEFFNTALKKHHQVSIAFSGGRSPISLLQKLSVLDLKWHACLVSLVDERIIDTNHDDSNTKLLHDYLLQNNALKASFIH266:HP1102       MGYQLFEFENLKDCHKALTERFKEFFNTALKKHHQISIAFSGGRSPISLLQKLSVLNLKWHECLISLVDERIIDTSHDDSNTKLLHDYLLQNNALKASFIHSJM:HPSJM_05440  MGYQLFEFESLEDCHRALTERFKEFFNATLKKHHQVSVAFSGGRSPISLLQKLSVLDLNWHECLISLVDERIIDTSHKDSNTKLLHDYLLQNNALNASFT                  101       111       121       131       141       151       161       171       181       191                         |         |         |         |         |         |         |         |         |         |         HB8:HPB8_402      PLLPKKISNDTNALFNFANQHFKQPHLAILGMGTDGHTASLFPETSAFLNEEKENIVLTKPTNAPYERLSMSVNALENCERLFLSISGVEKRGVLEKALKHB38:HELPY_1071   PLLPEKISSDTNALFNFANQHFKQPHLAILGMGTDGHTASLFPETSAFLNEEKENIVLTKPANAPYERLSMSVHALENCEKLFLSISGVEKREVLEKALKHHPA:HPAG1_1040   PLLPKKISSDTNALFHFANQHFKQPHLAILGMGTDGHTASLFPETSAFLNEEKENIVLTKPANAPYERLSMSVNALENCEKLFLSISGVEKREVLEKALKHP12:HPP12_1067   PLLPKKISGDTNALFHFANKHFKQPHLAILGMGTDGHTASLFPETSAFLNEEKENIVLTKPANAPYERLSMSINALENCEKLFLSISGVEKREVLEKALKHG27:HPG27_1043   PLLPKKISNDTNALFHFANQHFKQPHLAILGMGTDGHTASLFPETSAFLNEEKENIVLTKPINAPYERLSMSINALENCEKLFLSISGVEKREILEKALKHF32:HPF32_1038   PLLPEKISSDTNALFNFANQHFKQPHLAILGMGTDGHTASLFPETSAFLNEEKENIVLTKPANAPYERLSMSINALENCEKLFLSISGVQKRGVLEKALKHF16:HPF16_1044   PLLPEKISSDTNALFNFANQHFKQPHLAILGMGTDGHTASLFPETSAFLNEEKENIVLTKPANAPYERLSMSINALENCEKLFLSISGAQKRGVLEKALKH52:HPKB_1032     PLLPEKISNDTNALFNFANQHFKQPHLAILGMGTDGHTASLFPETSAFLNEEKENIVLTKPANAPYERLSMSINALENCEKLFLSISGAQKRGVLEKALEHF57:HPF57_1064   PILPEKISSDTNALFNFANQHFKQPHLAILGMGTDGHTASLFPETSAFLNEEKENIVLTKPANAPYERLSMSINALENCEKLFLSISGAQKRGVLEKALKHF30:HPF30_0287   PILPEKISSDTNALFNFANQHFKQPHLAILGMGTDGHTASLFPETSAFLNEEKENIVLTKPANTPYERLSMSINALENCEKLFLSISGAQKRGVLEKALKH51:KHP_1002      PILPEKISSDTNALFNFANQHFKQPHLAILGMGTDGHTASLFPETSAFLNEEKENIVLTKPANAPYERLSMSINALENCEKLFLSISEAQKRGVLEKALKH266:HP1102       PLLPEKISSDTNALFNFANQHFKQPHLAILGMGTDGHTASLFPETSAFLNEEKENIVLTKPINAPYERLSMSVNALENCEKLFLSISGVEKRGVLEKALKHSJM:HPSJM_05440  PLLPKKISGDTNELLDFANQHFKQPHLAILGMGTDGHTASLFPETSAFLNEEKENIVLTKPINAPYERLSMSINALENCEKLFLSISGVEKRGVLEKALK                  201       211       221                  |         |         |HB8:HPB8_402      ENAPYSLPIARILHSQKVTTEVFYAKNHB38:HELPY_1071   ENAPYSLPIARILHSKKVTTEVFYAKNHHPA:HPAG1_1040   ENAPYSLPIARILHSKKVTTEVFYAKNHP12:HPP12_1067   ENAPYSLPIARILHSKKVTTEVFYAKNHG27:HPG27_1043   ENAPYSLPIARILHSQKVTTEVFYAKNHF32:HPF32_1038   ENAPYSLPIARILHSQKVTTEVFYAKNHF16:HPF16_1044   ENAPYSLPIARILHSQKVTTEVFYAKNH52:HPKB_1032     ENAPYSLPIARILHSQKVTTEVFYAKNHF57:HPF57_1064   ENAPYSLPIARILHSQKVTTEVFYAKNHF30:HPF30_0287   ENAPYSLPIARILHSQKVTTEVFYAKNH51:KHP_1002      ENAPYSLPIARILHSQKVTTEVFYAKNH266:HP1102       ENAPYSLPIARILHSQKVTTEVFYAKNHSJM:HPSJM_05440  ENAPYSLPIARILHSQKVTTEVFYAKN
