# Supplementary material for: Evolution in an oncogenic bacterial species with extreme genome plasticity: Helicobacter pylori East Asian genomes
Source: BMC Microbiol. 2011 May 16;11:104. doi: 10.1186/1471-2180-11-104 (PMC3120642; doi:10.1186/1471-2180-11-104)
Supplement: Additional file 6 — Multiple sequence alignments of diverged genes. [file 1471-2180-11-104-S6.ZIP › Diverged_genes_multiple_seuence_alignments/HP1105.mfa.rtf]

                  1         11        21        31        41        51        61        71        81        91                          |         |         |         |         |         |         |         |         |         |         HB8:HPB8_399      MTSASSHSFKEQDFHIPIAFAFDKNYLIPAGACIYSLLESIAKANKKIRYTLHALVVGLNEEDRAKLNQITEPFKEFAVLEVKDIEPFLDAIPNPFDEDFH266:HP1105       MTSASSHSFKEQDFHIPIAFAFDKNYLIPAGACLYSLLESIAKANKKIRYTLHALVVGLNEEDKAKLNQITEPFKEFAALEVRDIESFLDTIPNPFDEDFHB38:HELPY_1074   MTSASNHSFKEQDFHIPIAFAFDKNYLIPAGACIYSLLESIAKANKKIRYTLHALVVGLNEEDKAKLNQIAEPFKEFAVLEVKDIEPFLDTIPNPFDEDFHG27:HPG27_1046   MTSASNHSFKEQDFHIPIAFAFDKNYLIPAGACLYSLLESIAKANKKIRYTLHALVVGLNEEDKAKLNQIAEPFKEFAVLEVKDIEPFLDTIPNPFDEDFHF32:HPF32_1042   MTSASSHSLKEQDFHIPIAFAFDKNYLIAASACIYSLLESIAKANKKIRYTLYALVVGLDEEDKAKLHQITEPFKEFAALEVKDIEPFLDTIPNPFDEDFHF57:HPF57_1068   MTSASSHSLKEQDFHIPIAFAFDKNYLIAASACIYSLLESIAKANKKIRYTLYALVVGLDEEDKAKLHQITEPFKEFAVLEVKDIESFLDTIPNPFDEDFHF16:HPF16_1048   MTSASSHSLKEQDFHIPIAFAFDKNYLIAAGACIYSLLESIAKANKKIRYTLYALVVGLDEEDKAKLHQITEPFKEFAALEVKDIEPFLDTIPNPFDEDFHF16:HPF16_1047   MTSASSHSLKEQDFHIPIAFAFDKNYLIAAGACIYSLLESIAKANKKIRYTLYALVVGLDEEDKAKLHQITEPFKEFAALEVKDIEPFLDTIPNPFDEDFH51:KHP_1006      MTSASSHSLKEQDFHIPIAFAFDKNYLIAASACIYSLLESIAKANKKIRYTLYALVVGLDEEDEAKLHQIAEPFKEFAALEVKDIEPFLDTIPNPFDEDFHF30:HPF30_0283   MTSGSSHSLKEQDFHIPIAFAFDKNYLIAAGACIYSLLESIAKANKKIRYTLYALVVGLDEEDKAKLHQITEPFKEFAALEVKDIEPFLDTIPNPFDEDFH52:HPKB_1036     MTSASSHSLKEQDFHIPIAFAFDKNYLIAAGACIYSLLESIAKANKKIRYTLYALVVGLDEEDKAKLHQITEPFKGFTALEVKDIEPFLDTIPNPFDEDFHHPA:HPAG1_1044   MTSSSSHSFKEQDFHIPIAFAFDKNYLIPAGACLYSLLESIAKANKKIRYTLHALVVGLNEEDKAKLHQITEPFKEFAALEVRDIEPFLDAIPNPFDEDFHP12:HPP12_1070   MTSASSHSFKEQDFHIPIAFAFDKNYLIPAGACLYSLLESIAKANKKIRYTLHALVVGLNEEDKAKLNQITEPFKEFAVLEVKDIEPFLDTIPNPFDEDFHSJM:HPSJM_05465  MTSASSHSLKEQDFHIPIAFAFDKNYLIPAGACIYSLLESIAKANKKIRYTLYALAVGLNGEDKAKLNQIAEPFKEFAALEVRDIESFLDTIPNPFDEDF                  101       111       121       131       141       151       161       171       181       191                         |         |         |         |         |         |         |         |         |         |         HB8:HPB8_399      TKRFSKMVLVKYFLADLFPKYSKMVWSDVDVIFCNEFSADFLNIKENDENYFYGVLEVEKHHMMEGFLFCNLDYQRKKNFTLRMHDLLKGNEAKGELDFTH266:HP1105       TKRFSKMVLVKYFLADLFPKYSKMVWSDVDVIFCNEFSADFLNLEENDENYFYGVLEVEKHHMMEGFLFCNLDYQRKKNFTLRMHELLRGNEAKGELDFTHB38:HELPY_1074   TKRFSKMVLVKYFLADLFPKYSKMVWSDVDVIFCNEFSADFLNIKENDENYFYGVLEVEKHHMLEGFLFCNLDYQRKKNFTLRMHDLLKGNGAKEELDFTHG27:HPG27_1046   TKRFSKMVLVKYFLADLFPKYSKMVWSDVDVIFCNEFSADFLNIKENDENYFYGVLEVEKHHMMEGFLFCNLDYQRKKNFTLRMHEILKGNEAKGELDFTHF32:HPF32_1042   TKRFSKMVLVKYFLADLFPKYSKMVWSDVDVIFCNEFSADFLNIKEDDENYFYGVLEVEKHHIMEGFLFCNLDYQRKKNFTLRMHDLLKGNETKEELDFTHF57:HPF57_1068   TKRFSKMVLVKYFLADLFPKYSKMVWSDVDVIFCNEFSADFLNIKEDDENYFYGVLEVEKHHMMEGFLFCHLDYQRKKNFTLRMHDLLRGNEAKEELDFTHF16:HPF16_1048   TKRFSKMVLVKYFLADLFPKYSKMVWSDVDVIFCNEFSADFLNIKEDDENYFYGVLEVEKHHMMEGFLFCHLDYQRKKNFTLRMHDLLKGNEAKEELDFTHF16:HPF16_1047   TKRFSKMVLVKYFLADLFPKYSKMVWSDVDVIFCNEFSADFLNIKEDDENYFYGVLEVEKHHMMEGFLFCHLDYQRKKNFTLRMHDLLKGNEAKEELDFTH51:KHP_1006      TKRFSKMVLVKYFLADLFPQYSKMVWSDVDVIFCNEFSADFLNIKEDDENYFYGVLEVEKHHMMEGFLFCHLDYQRKKNFTLRMHDLLKGNEATEELDFTHF30:HPF30_0283   TKRFSKMVLVKYFLADLFPKYSKMVWSDVDVIFCNEFSADFLNIKEDDENYFYGVLEVEKHHMMEGFLFCHLDYQRKKNFTLRIHDLLKGNEAKGELDFTH52:HPKB_1036     TKRFSKMVLVKYFLADLFPKYSKMVWSDVDVIFCNEFSADFLNIKEDDENYFYGVLEVEKHHMMEGFLFCNLDYQRKKNFTLRMHDLLKGNEAKEELDFTHHPA:HPAG1_1044   TKRFSKMVLVKYFLADLFPKYSKMVWSDVDVIFCNEFSADFLNIKENDENYFYGVLEVEKHHMMEGFLFCNLDYQRKKNFTLRMHDLLKGNEAKGELDFTHP12:HPP12_1070   TKRFSKMVLVKYFLADLFPKYSKMVWSDVDVIFCNEFSADFLNIKENDENYFYGVLEVEKHHMMEGFLFCNLDYQRKKNFTLRMHDLLKGNEAKGELDFTHSJM:HPSJM_05465  TKRFSKMVLVKYFLADLFPKYSKMVWSDVDVIFCNEFSADFLNIKEDDENYFYGVLEVEKHHMMEGFLFCNLDYQRKKNFTLRMHELLRGNEAKGELDFT                  201       211       221       231       241       251       261       271       281       291                         |         |         |         |         |         |         |         |         |         |         HB8:HPB8_399      KWCWPNMKALGIEYCVFPYYYTIKDFSNAYLNENYKKTILEARENPTIIHYDAWWGAVKPWDYPFGLKADLWLNALAKTPFMSDWSYLITGGGEIGGEKWH266:HP1105       KWCWPNMKALGIEYCVFPYYYTIKDFSNAYLNENYKKTILEARENPTIIHYDAWWGAVKPWDYPFGLKADLWLNALAKTPFMSDWIDSI-ARVEIGSEKWHB38:HELPY_1074   KWCWPNMKALGIEYCVFPYYYTIKDFSNAHLNENYKKTILEARENPTIIHYDAWWGAVKPWDYPFGLKADLWLNALAKTPFMSDWIDSI-ARVEIGSEKWHG27:HPG27_1046   KWCWPNMKALGIEYCVFPYYYTIKDFSNAYLNENYKKTILEVRENPTIIHYDAWWGAVKPWDYPFGLKADLWLNALAKTPFMSDWSYLITGGGEIGGEKWHF32:HPF32_1042   KWCWPNMKALGIEYCVFPLYYTIKDFSNVYLNENYKKTILEALKNPIIIHYDAWWGAVKPWDYPFGLKADLWLNALSKTPFMSDYTKKM-HTNE------HF57:HPF57_1068   KWCWPNMKALGIEYCVFPLYYTIKDFSNVYLNENYKKTILEALKNPIIIHYDAWWGAVKPWDYPFGLKADLWLNALSKTPFMSDYTKKM-HTNE------HF16:HPF16_1048   KWCWPNMKALGIEYCVFPLYYTIKDFSNVYLNENYKKTILEALKNPIIIHYDAWWGAVKPWDYPFGLKADLWLNALSKTPFMSDYTKKM-HTNE------HF16:HPF16_1047   KWCWPNMKALGIEYCVFPLYYTIKDFSNVYLNENYKKTILEALKNPIIIHYDAWWGAVKPWDYPFGLKADLWLNALSKTPFMSDYTKKM-HTNE------H51:KHP_1006      KWCWPNMKALGIEYCVFPLYYTIKDFSNVYLNENYKKTILEALKNPIIIHYDAWWGAVKPWDYPFGLKADLWLNALSKTPFMSDYTKKM-HTNE------HF30:HPF30_0283   KWCWPNMKALEIEYCIFPLYYTIKDFSNAYLNENYKKTILEALKNPTIIHYDAWWGAVKPWDYPFGLKADLWLNALSKTPFMSDYTKKM-HTNE------H52:HPKB_1036     KWCWPNMKALGIEYCVFPYYYTIKDFSNAYLNENYKKTILEARENPTIIHYDAWWGAVKPWDYPFGLKADLWLNALSKTPFMSDYTKKM-HTNE------HHPA:HPAG1_1044   KWCWPNMKALGIEYCVFPYYYTIKDFSNAYLNENYKKTILEALKNPTIIHYDAWWGAVKPWDYPFGLKADLWLNALAKTPFMSDYTKKM-HTNE------HP12:HPP12_1070   KWCWPNMKALGIEYCVFPYYYTIKDFSNAYLNENYKKTILEARENPTIIHYDAWWGAVKPWDYPFGLKADLWLNALAKTPFMSDYTKKM-HTNE------HSJM:HPSJM_05465  KWCWPNMKALGIEYCVFPYYYTIKDFSNAYLNENYKKTILEARENPTIIHYDAWWGAVKPWDYPFGLKADLWLNALAKTPFMSDYTKKM-HTNE------                  301       311       321       331       341       351       361       371       381       391                         |         |         |         |         |         |         |         |         |         |         HB8:HPB8_399      HHYHSIAAYHYYFPLWKAEEQIAHDAFKTFLKHYFL----------------------------------------HIHEIPQNARRRLF----KYCISIH266:HP1105       HRYHSIVAYHYYFPLWKTEEQIAHDALKTFLDHYFSCIHAAIKQENLGMFLNHYFSHAHAEIKENSLEMFLNHYFSHVYRLPKKARKRLFRVFVKHCILIHB38:HELPY_1074   HRYHSIVAYHYYFPLWKTEEQIAHDALKTFLDHYFSHIHATIKQESLGMFLNHYFSHAHAEIKENSLEAFLNHYFSHVYRLPKNARKKLLRVFVKHCILIHG27:HPG27_1046   HHYHSIAAYHYYFPLWKAEEQIAHDALKIFLDHYFSCIHAAIKQENLGMFLNHYFSHAHAEIKENSLEAFLNHYFSHVYKLPKNARKRLFRVFVKHCILIHF32:HPF32_1042   SFYTTKMAQQHYFSSTAPSKEILFKAPYLFFKSYLFVV-----------------------FKERKIH-------SRVFELTCVLVKKFFNKLIYFGFFIHF57:HPF57_1068   SFYTTKMAQQYYFSSTAPSKEILFKAPYLFFKSYLFVV-----------------------FKERKIH-------SRVFELTCGLVKKFFNKLIYFGFFMHF16:HPF16_1048   SFYTTKMAQQHYFSSTAPSKEILFKAPYLFFKSYLFVV-----------------------FKERKIH-------SRVFELTCGLVKKIFNKLIYFGFFMHF16:HPF16_1047   SFYTTKMAQQHYFSSTAPSKEILFKAPYLFFKSYLFVV-----------------------FKERKIH-------SRVFELTCGLVKKIFNKLIYFGFFMH51:KHP_1006      SFYTTKMAQQHYFSSTAPSKEILFKAPYLFFKSYLFVV-----------------------FKERKIH-------SRVFELTCGLVKKFFNKLIYFGFFMHF30:HPF30_0283   SFYTTKMAQQHYFSSTAPSKEILFKAPYLFFKSYLFVV-----------------------FKERKIH-------SRVFELTCGLVKKFFNKLIYFGFFMH52:HPKB_1036     SFYTTKMAQQHYFSSTAPSKEILFKAPYLFFKSYLFVV-----------------------FKERKIH-------SRVFELTCGLVKKFFNRLIYFGFFMHHPA:HPAG1_1044   SFYTTKMAEQHYFSSVKSSKEIVFKAPYLFFKSYLFVV-----------------------FKERKIH-------SRVFELTCNLVKKFFNKLIYFGFLMHP12:HPP12_1070   SFYTTKMAEQHYFSSVKSSKEIVFKAPYLFFKSYLFVV-----------------------FKERKIH-------SRVFELTCNLVKKFFNKLIYFGFLMHSJM:HPSJM_05465  SFYTTKMAEQHYFSSIKSSKEILFKTPYLFFKSYLFVV-----------------------FKERKIH-------LRIFALMGGLVKKFFNKLIHFVFLM                  401       411       421       431                  |         |         |         |HB8:HPB8_399      P---LKSFISKTLKILKLHALVKKILIQLKLLKKSH266:HP1105       P---LKSLVGKTLRLLKLHALAKKILIQLKLLKKSHB38:HELPY_1074   P---LKSLISKTLKILGLHVLAKKILIQLKLLRKSHG27:HPG27_1046   P---LKSLISKTLKILKLHALAKNTLIQLKLLKKSHF32:HPF32_1042   SKSLAKRVVSKTLKILGLHGIVKKILIKLKLLKKSHF57:HPF57_1068   PKSLAKRVVSKTLKILGLHGIVKKIL--LKLLKKSHF16:HPF16_1048   PKSLAKRVVSKILRFLGLHGIVKKIL--LKLLKKGHF16:HPF16_1047   PKSLAKRVVSKILRFLGLHGIVKKIL--LKLLKKGH51:KHP_1006      PKSLAKRVVSKILRVLGLHGIAKKILIKLKLLKKSHF30:HPF30_0283   PKSLAKRVVSKTLKILGLHGIAKKILIKLKLLKKSH52:HPKB_1036     PKSLAKRVVSKILRVLGLHGIAKKILIKLKILKKSHHPA:HPAG1_1044   PKALAKRVVSKILRVLGLHGIVKKILIKLKLLRKGHP12:HPP12_1070   PKALAKRVVSKILRILGLHGIVKKILIQLKLLKKSHSJM:HPSJM_05465  PKALVKRAVSKILRVLGLHGIVKKILIQLKLLRKG
