# Supplementary material for: Evolution in an oncogenic bacterial species with extreme genome plasticity: Helicobacter pylori East Asian genomes
Source: BMC Microbiol. 2011 May 16;11:104. doi: 10.1186/1471-2180-11-104 (PMC3120642; doi:10.1186/1471-2180-11-104)
Supplement: Additional file 6 — Multiple sequence alignments of diverged genes. [file 1471-2180-11-104-S6.ZIP › Diverged_genes_multiple_seuence_alignments/HP1113_horI.mfa.rtf]

                  1         11        21        31        41        51        61        71        81        91                          |         |         |         |         |         |         |         |         |         |         HB8:HPB8_391      LKRALCLVLGLFCALNAKGFKDVLTKGDYTFFNKKVVSPIKRYADQSAFYLGLGYQLGSIQHNSSNLNLSQQFNKSQIVFSDGLSPVFKNSYVSNGLGVQHSJM:HPSJM_05505  LKRALCLILGLSCVLNAKGFKDVLTKGDYTFFNKKVVSPIKRYADRSAFYLGLGYQLGSIQHNYSNLNLSQQFNKSQIIFSDGLSPVFKNSYVSNGLGVQHB38:HELPY_1083   LKRALYLILGLSYALHADSFKDVLTKGDYTFFNKKVVSPIKRYADRSAFYLGLGYQLGSIQHNYSNLNLSQQFNKSQIIFSDGLSPVFKNSYVSNGLGVQHP12:HPP12_1078   LKRALYLVLGLSYALHADSFKDVLTKGDYTFFNKKVVSPIKRYADQSAFYLGLGYQLGSIQHNYSNLNLSQQFNKSQIVFSDGLSPVFKNSYVSNGLGVQHG27:HPG27_1055   LKRALYLVLGLSYALHADSFKDVLTKGDYTFFNKKVVSPIKRYADQSAFYLGFGYQLGSIQHNYSNLNLSQQFNKSQIIFSDGLSPVFKNSYVSNGFGVQHHPA:HPAG1_1052   LKRALYLILGLFCTLNAESFKDVLTKGDYTFFNQKVVSPIKRYADRSAFYLGLGYQLGSIQHNYSNLNLSQQFNKSQIIFSDGLSPVFKNSYVSNGFGVQH266:HP1113       LKRALYLILGLFYTLNAESFKDVLTKVDYTFFNKKVVSPIKRYADRSAFYLGLGYQLGSIQHNSSNLNLSQQFNKSQIIFSDSLSPVFKNSYVSNGLGVQHF32:HPF32_1050   LKRALCLILGLSCALNAKGFKDVLTKGDYTFFNKKVVSPIKRYADRSAFYLGLGYQLGSIQRNSSNLNLFQNFTRTQILFSDGLSPVFKNSYVSNGLGVQHF16:HPF16_1056   LKRALCLILGLFCVLNAKGFKDVLTKGDYTFFNKKVVSPIKRYADRSAFYLGLGYQLGSIQRNSSNLNLFQNFTRTQILFSDGLSPVFKNSYVSNGLGVQHF57:HPF57_1076   LKRALCLILGLSCALNAKGFKDVLTKGDYTFFNKKVVSPIKRYADKSAFYLGLGYQLGSIQHNSSNLNLFQRFTRTQIIFSDGLSPVFKNSYVSNGLGVQHF30:HPF30_0275   LKRALCLILGLFCTLNAKGFKDVLTKGDYTFFNRKVVSPIKRHADKSAFYLGLGYQLGSIQHNSSNLNLFQRFTRTQIIFSDGLSPVFKNSYVSNGLGVQH52:HPKB_1044     LKRALCLILGLFCTLNAKGFKDVLTKGDYTFFNKKVVSPIKRYADRSAFYLGLGYQLGSIQHNSSNLNLFQNFTRTQILFSDGLSPVFKNSYVSNGLGVQ                  101       111       121       131       141       151       161       171       181       191                         |         |         |         |         |         |         |         |         |         |         HB8:HPB8_391      AGYKWVGKHEETKWFGFRWGLFYDLSASLYGQKESQSVIISTYGTYMDLLLNAYNGDKFFAGFNLGIAFAGVYDRLSDALLYQALLQNTFGGKVDPNGFQHSJM:HPSJM_05505  AGYKWVGKHEETKWFGFRWGLFYDLSASLYGQKESQSIIISTYGTYMDLLLNAYNGDKFFAGFNLGIAFAGVYDKLSDALLYQALLLDTFGGKVDPNGFQHB38:HELPY_1083   VGYKWVGKHEEMKWFGFRWGLFYDLSASLYGQKESQSVIISTYGTYMDLLLNAYNGDKFFAGFNLGIAFAGVYDKLSDALLYQALLLDTFGGKVDPNGFQHP12:HPP12_1078   VGYKWVGKHEETKWFGFRWGLFYDLSASLYGAQESQSVIISTYGTYMDLLFNAYNGDKFFAGFNLGIAFAGVYDKLSDALLYQALLLDTFGGKVNPNGFQHG27:HPG27_1055   VGYKWVGKHEETKWFGFRWGLFYDLSASLYGQKESQSIIISTYGTYMDLLFNAYNGDKFFAGFNLGIAFAGVYDKLSDELLYQALLLDTFGGKVNLNGFQHHPA:HPAG1_1052   VGYKWVGKHEETKWFGFRWGLFYDLSASLYGQKESQSIIISTYGTYMDLLLNAYNGGKFFAGFNLGIAFAGVYDKLSDALLYQALLLDTFGGKVDPNGFQH266:HP1113       VGYKWVGKHEETKWFGFRWGLFYDLSASLYGQKESQSVIISTYGTYMDLLLNAYNGDKFFAGFNLGIAFAGVYDKVSDALLYQALLLDTFGGKVDPNGFQHF32:HPF32_1050   VGYKWVGKHEETKWFGFRWGLFYDLSASLYGLKESQSVIISTYGTYMDLLFNAYNGDKFFAGFNLGIAFAGVYNKLSDVLLYQTLLQDTFGGKVDPNGFQHF16:HPF16_1056   VGYKWVGKHEETKWFGFRWGLFYDLSASLYGLKESQSVLISTYGTYMDLLLNAYNGDKFFAGFNLGIAFAGVYNKLSDALLYQTLIQDTFGGKVDPNGFQHF57:HPF57_1076   VGYKWVGKHEETKWFGFRWGLFYDLSASLYGPQESQSVIISTYGTYMDLLFNAYNGDKFFAGFNLGIAFAGVYNKLSDALLYQTLLQDTFGGKVDPNGFQHF30:HPF30_0275   VGYKWVGKHEETKWFGFRWGLFYDLSASLYGLKESQSVIISTYGTYMDLLFNAYNGDKFFAGFNLGIAFAGVYNKLSDALLYQTLLQDTFGGKVDPNGFQH52:HPKB_1044     VGYKWVGKHEETKWFGFRWGLFYDLSASLYGAQESQSVIISTYGTYMDLLFNAYNGDKFFAGFNLGIAFAGVYDKVSDELLYQALLLDTFGGKVNPNGFQ                  201       211       221       231       241       251       261       271                  |         |         |         |         |         |         |         |HB8:HPB8_391      FLVDLGVRLGSKRNQFGFGIKIPTYYFNHYYSMNNISNNSGDVLKVLRFLEYGINSLLYQVDFRRNYSVYFNYTYSFHSJM:HPSJM_05505  FLVDLGVRLGNKRNQFGFGIKIPTYYFNHYYSMNNISNNSEDILKVLRFLEYGINSLLYQVDFRRNYSVYFNYTYSFHB38:HELPY_1083   FLVDLGVRLGNKRNQFGFGIKIPTYYFNHYYSMNNISNNSEDVLKVLRFLEYGINSLLYQVDFRRNYSVYFNYTYSFHP12:HPP12_1078   FLVNLGVRLGSKRNQFGFGIKIPTYYFNHYYSMNNISNNSGDVLKVLRFLEYGINSLLYQVDFRRNYSVYFNYTYSFHG27:HPG27_1055   FLVDLGVRLGNEHNQFGFGIKVPTYYFNHYYSMNNISNNSEDVLKVLRFLEYGINSLLYQVDFRHNYSVYFNYTYSFHHPA:HPAG1_1052   FLVNLGVRLGNKRNQFGFGIKIPTYYFNHYYSMNNISNNSGDVLKVLRFLEYGINSLLYQVDFRRNYSVYFNYTYSFH266:HP1113       FLVNLGVRLGNKHNQFGFGIKIPTYYFNHYYSMNNISNNSEDVLKVLRFLEYGINSLLYQVDFRRNYSVYFNYTYIFHF32:HPF32_1050   FLVDLGVRLGNKHNQFGFGIKVPTYYFNHYYSMNNISNNSGDVLKVLRFLEYGINSLLYQVDFRRNYSVYFNYTYSFHF16:HPF16_1056   FLVDLGVRLGNEHNQFGFGIKVPTYYFNHYYSMNNISNNSGDVLKVLRFLEYGIRSVLYRVDFRRNYSVYFNYTYSFHF57:HPF57_1076   FLVDLGVRLGNERNQFGFGIKVPTYYFNHYYSMNNISNNSGNVLKVLRFLEYGINSVLYRVDFRRNYSVYFNYTYSFHF30:HPF30_0275   FLVDLGVRLGNERNQFGFGIKVPTYYFNHYYSMNNISNNSGDVLKVLRFLEYGINSLLYRVDFRRNYSVYFNYTYSFH52:HPKB_1044     FLVDLGVRLGSKRNQFGFGIKVPTYYFNHYYSMNNISNNSKDVLKVLRFLEYGINSLLYQVDFRRNYSVYFNYTYSF
