# Supplementary material for: Evolution in an oncogenic bacterial species with extreme genome plasticity: Helicobacter pylori East Asian genomes
Source: BMC Microbiol. 2011 May 16;11:104. doi: 10.1186/1471-2180-11-104 (PMC3120642; doi:10.1186/1471-2180-11-104)
Supplement: Additional file 6 — Multiple sequence alignments of diverged genes. [file 1471-2180-11-104-S6.ZIP › Diverged_genes_multiple_seuence_alignments/HP1143.mfa.rtf]

                  1         11        21        31        41        51        61        71        81        91                          |         |         |         |         |         |         |         |         |         |         HB8:HPB8_359      MQENQTRPFICPKCQEPINVNEALYKQIEQENQSRFLAQQKAFEKEVNEKRAQYQSHFKALEQKEEALKEREREQKAQFDDAVKQASALALQDERAKIIEHG27:HPG27_1088   MQENQTRPFICPNCQAPINVNEALYKQIEQENQSRFLAQQKAFEKEVNEKRAQYQSHFKALEQKEEALKEREREQKAQFDDAVKQASVLALQDERAKIIEH266:HP1143       MQENQTRPFICPKCQEPIDVNEALYKQIEQENQNKFLAQQKEFEKEVNEKRAQYLSYFKNLEQKEETLKEREKEQQAKFDEAVKQASALALQDERAKIIEHB38:HELPY_1115   MQENQTRPFICPKCQEPINVNEVLYKQIELENQNKFLAQQKEFEKEVNEKRAQYLSYFKNLEQKEEALKEREKEQKAKFDDAVKQASALALQDERAKIIEHP12:HPP12_1109   MQENQTRPFICPNCQVPIDVNEALYKQIEQENQSRFLAQQKAFEKEMNEKRAQYQSHSKALKQKEEALKEREREQKAQFDDAVKQASALALQDERAKIIEHSJM:HPSJM_05665  MQENQTRPFICPKCQEPIDVNEALYKQIEQENQNKFLAQQKAFEKEVNEKRAQYHTHLKMLEQKEEALKERAKEQQAQFDDAVKQASVLALQDERAKIIEHHPA:HPAG1_1081   MQENQTIPFICPKCQEPIDVNEALYKQIEQENQSRFLAQQKAFEKEVKEKRAQYQSHFKALEQKEEALKERAKEQQAKFDEAVKHASVLALQDERAKIIEHF32:HPF32_1080   MQENQTHSFKCPNCQVVIDVNEALYKQIELENQSRFLAQQKEFEKEVKEKRAQYQSHFKMLEQKEEALKEQEREQKAKFDDAVKQASTLALQDERAKIIEHF16:HPF16_1085   MQENQTHSFKCPNCQVVIDVNEALYKQIELENQSRFLAQQKEFEKEVKEKRAQYQSHFKILEQKEEALKEREREQKAKFDDAVKQASALALQDERAKIIEHF57:HPF57_1106   MQENQTHSFKCPNCQVVIDVNEALYKQIELENQSRFLAQQKEFEKEVKEKRAQYQSHFKMLEQKEEALKEQEREQKAKFDDAVKQASALALQDERAKIIEH52:HPKB_1073     MQENQTHSFKCPNCQVVIDVNEALYKQIELENQSRFLAQQKEFEKEVKEKRAQYQSHFKMLEQKEEALKEREREQKAKFDDAVKQASALALQDERAKIIEH51:KHP_1041      MQENQTHSFKCPNCQVVIDVNEALYKQIELENQSRFLAQQKEFEKEVKEKRA-------MLEQKEEALKEQEREQKAKFDDAVKQASALALQDERAKIIEHF30:HPF30_0246   MQENQTHSFKCPNCQAPIDVNEALYKQIELENQSRFLAQQKEFEKEVKEKRVQYQSHFKMLEQKEEALKEQEREQKAKFDDAVKQASVLALQDERAKIIE                  101       111       121       131       141       151       161       171       181       191                         |         |         |         |         |         |         |         |         |         |         HB8:HPB8_359      EARKNAFLEQQKGLELLQKELEEKSKQVQELHQKEAEIERLKRENNEAESRLKAENEKKLNEKLEMERERIEKALHEKNELKFKQQEEQLEMLRNELKNAHG27:HPG27_1088   EARKNAFLEQQKGLELLQKELEEKSKQVQELHQKEAEIERLKRENNEAESRLKAENEKKLNEKLDLEREKIEKALHEKNELKFKQQEEQLEMLRNELKNAH266:HP1143       EARKNAFLEQQKGLELLQKELDEKSKQVQELHQKEAEIERLKRENNEAESRLKAENEKKLNEKLDLEREKIEKALHEKNELKFKQQEEQLEMLRNELKNAHB38:HELPY_1115   EARKNAFLEQQKGLELLQKELEEKSKQVQELHQKEAEIERLKRENNEAESRLKAENEKKLNEKLDLEREKIEKALHEKNELKFKQQEEQLEMLRNELKNAHP12:HPP12_1109   EARKNAFLEQQKGLELLQKELDEKSKQVQQLHQKEAEIERLKRENNEAESRLKAENEKKLNEKLDLERERIEKALHEKNELKFKQQEEQLEMLRNELKNAHSJM:HPSJM_05665  EARKNAFLEQQKGLELLQKELDEKSKQVQELHQKEAEIERLKRENNEAESRLKAENEKKLNEKLDLEREKIEKALHEKNELKFKQQEEQLEMLRNELKNAHHPA:HPAG1_1081   EARKNAFLEQQKGLELLQKELDEKSKQVQELHQKEAEIERLKRENNEAESRLKAENEKKLNEKLDLEREKIEKALHEKNELKFKQQEEQLEMLRNELKNAHF32:HPF32_1080   EARKNAFLEQQKGLELLQKELDEKSKQVQELHQKEAEIERLKRENNEAESRLKAENEKKLNEKLETEREKIEKALHEKNELKFKQQEEQLEMLRNELKNAHF16:HPF16_1085   EARKNAFLEQQKGLELLQKELDEKSKQVRELHQKEAEIERLKRENNEAESRLKAENEKKLNEKLETERERIEKALHEKNELKFKQQEEQLEMLRNELKNAHF57:HPF57_1106   EARKNAFLEQQKGLELLQKELDEKSKQVQELHQKEAEIERLKRENNEAESRLKAENEKKLNEKLDLERERIEKALHEKNELKFKQQEEQLEMLRNELKNAH52:HPKB_1073     EARKNAFLEQQKGLELLQKELDEKSKQVQELHQKEAEIERLKRENNEAESRLKAENEKKLNEKLDLERERIEKALHEKNELKFKQQEEQLEMLRNELKNAH51:KHP_1041      EARKNAFLEQQKGLELLQKELDEKSKQVQELHQKEAEIERLKRENNEAESRLKAENEKKLNEKLDLERERIEKALHEKNELKFKQQEEQLEMLRNELKNAHF30:HPF30_0246   EARKNAFLEQQKGLELLQKELDEKSKQVQELHQKEAEIERLKRENNEAESRLKAENEKKLNEKLETEREKIEKALHEKNELKFKQQEEQLEMLRNELKNA                  201       211       221       231       241       251       261       271       281       291                         |         |         |         |         |         |         |         |         |         |         HB8:HPB8_359      QRKAELSSQQLQGEVQELAIEEFLKQKFPLDSVEEIKKGQRGGDCIQVVHTREFQNCGKIYYESKRTKEFQKAWVEKLKSDMREIGADVGVIVSEALPKEHG27:HPG27_1088   QRKAELSSQQLQGEVQELAIEEFLRQKFPLDSIEEIKKGQRGGDCIQVVHTREFQNCGKIYYESKRTKEFQKAWVEKLKSDMREIGADVGVIVSEALPKEH266:HP1143       QRKAELSSQQFQGEVQELAIEEFLRQKFPLDCIEEIKKGQRGGDCIQVVHTREFQNCGKIYYESKRTKEFQKAWVEKLKSDMREIGADVGVIVSEALPKEHB38:HELPY_1115   QRKAELSSQQFQGEVQELAIEEFLRQKFPLDSVEEIKKGQRGGDCIQVVHTREFQNCGKIYYESKRTKEFQKAWVEKLKSDMREIGADVGVIVSEALPKEHP12:HPP12_1109   QRKAELSSQQFQGEVQELAIEEFLRQKFPLDSVEEIKKGQRGGDCIQVVHTREFQNCGKIYYESKRTKEFQKAWVEKLKSDMREIGADVGVIVSEALPKEHSJM:HPSJM_05665  QRKAELSSQQFQGEVQELAIEEFLRQKFPLDSVEEIKKGQRGGDCIQVVHTREFQNCGKIYYESKRTKEFQKAWVEKLKSDMREIGADVGVIVSEALPKEHHPA:HPAG1_1081   QRKAELSSQQFQGEVQELAIEEFLRQKFPLDSIEEIKKGQRGGDCIQVVHTREFQNCGKIYYESKRTKEFQKAWVEKLKSDMREIGADVGVIVSEALPKEHF32:HPF32_1080   QRKAELSSQQFQGEVQELAIEEFLRQKFPLDCVEEIKKGQRGGDCIQVVHTREFQNCGKIYYESKRTKEFQKAWVEKLKSDMREIGADVGVIVSEALPKEHF16:HPF16_1085   QRKAELSSQQFQGEVQELAIEEFLRQKFPLDCIEEIKKGQRGGDCIQVVHTREFQNCGKIYYESKRTKEFQKAWVEKLKSDMREIGADVGVIVSEALPKEHF57:HPF57_1106   QRKAELSSQQFQGEVQELAIEEFLRQKFPLDSVEEIKKGQRGGDCIQVVHTREFQNCGKIYYESKRTKEFQKAWIEKLKSDMREIGADVGVIVSEVLPKEH52:HPKB_1073     QRKAELSSQQFQGEVQELAIEEFLRQKFPLDCIEEIKKGQRGGDCIQVVHTREFQNCGKIYYESKRTKEFQKAWIEKFKSDMREIGADVGVIVSEALPKEH51:KHP_1041      QRKAELSSQQFQGEVQELAIEEFLRQKFPLDCIEEIKKGQRGGDCIQVVHTREFQNCGKIYYESKRTKEFQKAWVEKLKSDMREIGADVGVIVSEALPKEHF30:HPF30_0246   QRKAELSSQQFQGEVQELAIEEFLRQRFPLDCVEEIKKGQRGGDCIQVVHTREFQNCGKIYYESKRTKEFQKAWVEKLKSDMREIGADVGVIVSEALPKE                  301       311       321       331       341       351       361       371       381       391                         |         |         |         |         |         |         |         |         |         |         HB8:HPB8_359      MERMGLFEGVWVCSFEEFKGLSAVLREGVIQVGLAKKSQENKGDKVNLLYHYLTSSEFSMQVNAIIEGFEQLRADLESEKRAMARIWKSREKQMEKVFEGHG27:HPG27_1088   MERMGLFEGVWVCSFEEFKGLSAVLREGVIQVSLAKKSQENKGDKVDLLYHYLTSSEFSMQVNAIIEGFERLRVDLESEKRAMNRIWKSREKQMEKVFEGH266:HP1143       MERMGLFEGVWVCSFEEFKGLSAVLREGVIQVSLAKKSQENKGDKVNLLYHYLTSSEFSMQVNVIIEGFEQLRADLESEKRAMARIWKSREKQIDKVFEGHB38:HELPY_1115   MERMGLFEGVWVCSFEEFKGLSAVLREGVIQVGLAKKSQENKGDKVNLLYHYLTSSEFSMQVNAIIEGFEQLRADLESEKRAMNRIWKSREKQIDKVFEGHP12:HPP12_1109   MERMGLFEGVWVCSFEEFKGLSAVLREGVIQVGLAKKSQENKGDKMDLLYHYLTSSEFSMQVNAIIEGFEQLRADLESEKRAMARIWKSREKQIDKVFEGHSJM:HPSJM_05665  MERMGLFEGVWVCSFEEFKGLSAVLREGVIQVGLAKKSQENKGDKMDLLYHYLTSSEFSMQVNAIIEGFEQLRADLEGEKRAMARIWKSREKQIDKVFEGHHPA:HPAG1_1081   MERMGLFEGVWVCSFEEFKGLSAVLREGVIQVSLAKKSQENKGDKVNLLYHYLTSSEFSMQVNAIIEGFEQLRADLEKEKNAMARIWKSREKQIEKVFEGHF32:HPF32_1080   MERMGLFEGVWVCSFEEFKGLSAVLREGVIQVSLAKKSQENKGDKVNLLYHYLTSSEFSMQVSAIIEGFEQLKADLEKEKNAMARIWKSREKQMEKVFEGHF16:HPF16_1085   MERMGLFEGVWVCSFEEFKGLSAVLREGVIQVSLAKKSQENKGDKVDLLYHYLTSSEFSMQVSAIIEGFEQLRAELEKEKNAMARIWKSREKQMEKVFEGHF57:HPF57_1106   MERMGLFEGVWVCSFEEFKGLSAVLREGVIQVGLAKKSQENKGDKVNLLYHYLTSSEFSMQVSAIIEGFEQLRADLEKEKNAMARIWKSREKQIEKVFESH52:HPKB_1073     MERMGLFEGVWVCSFEEFKGLSAVLREGVIQVSLAKKSQENKGDKVNLLYHYLTSSEFSMQVSAIIEGFEQLRAELEKEKNAMARIWKSREKQMEKVFEGH51:KHP_1041      MERMGLFEGVWVCSFEEFKGLSAVLREGVIQVSLAKKSQENKGDKVNLLYHYLTSSEFSMQVSAIIEGFEQLRAELEKEKNAMARIWKSREKQIEKVFEGHF30:HPF30_0246   MERMGLFEGVWVCSFEEFKGLSAVLREGVIQVSLAKKSQENKGDKVDLLYHYLTSSEFSMQVSAIIEGFEQLRAELENEKRAMARIWKSREKQIEKVFEG                  401       411       421       431                  |         |         |         |HB8:HPB8_359      TINMYGSIKGIAGNAIGQVKALELGYDGEDLEDHG27:HPG27_1088   TINMYGSIKGIAGNAIGQVKALELGYDGEDLE-H266:HP1143       TINMYGSIKGIAGNAIGQVKALELGYDGEDLEDHB38:HELPY_1115   TINMYGSIKGIAGNAIGQVKALELGYDGEDLEDHP12:HPP12_1109   TINMYGSIKGIVGNTIGQVKALELGYDEEDLEDHSJM:HPSJM_05665  TINMYGSIKGIAGNAIGQVKALELGYDERDLEDHHPA:HPAG1_1081   TINMYGSIKGIAGNAIGQVKALELGYDEEDLE-HF32:HPF32_1080   TINMYGSIKGIMGNAIGQVKALELGYDGEDLEDHF16:HPF16_1085   TINMYGSIKGIMGNAIGQVKALELGYDGEDLEDHF57:HPF57_1106   TINMYGSIKGIMGNAIGQVKALELGYDGEDLE-H52:HPKB_1073     TINMYGSIKGIMGNAIGQVRALELGYDGEDLE-H51:KHP_1041      TINMYGSIKGIMGNAIGQVKALELGYDGEDLEEHF30:HPF30_0246   TINMYGSIKGIMGNAIGQVKALELGYDGEDLED
