# Supplementary material for: Evolution in an oncogenic bacterial species with extreme genome plasticity: Helicobacter pylori East Asian genomes
Source: BMC Microbiol. 2011 May 16;11:104. doi: 10.1186/1471-2180-11-104 (PMC3120642; doi:10.1186/1471-2180-11-104)
Supplement: Additional file 6 — Multiple sequence alignments of diverged genes. [file 1471-2180-11-104-S6.ZIP › Diverged_genes_multiple_seuence_alignments/HP1163_fixS.mfa.rtf]

                  1         11        21        31        41        51        61                  |         |         |         |         |         |         |HB8:HPB8_334      MNTEILTIMLVVSVLMGLVGLIAFLWGVKSGQFDDEKRMLESVLYDSASDLNEAILQEKRQDPKNHSJM:HPSJM_05780  MNTEILTIMLVVSVLMGLVGLIAFLWGVKSGQFDDEKRMLESVLYDSASDLNEAILQEKRQDPKNHG27:HPG27_1107   MNTEILTIMLVVSVLMGLVGLIAFLWGVKSGQFDDEKRMLESVLYDSASDLNEAILQEKRQDPKNHF32:HPF32_1098   MNTEILTIMLVVSVLMGLIGLIAFLWGVKSGQFDDEKRMLESVLYDSTSDLNEAILQEKRQE--NH51:KHP_1059      MNTEILTIMLVVSVLMGLIGLIAFLWGVKSGQFDDEKRMLESVLYDSTSDLNEAVLQEKRQE--NHF30:HPF30_0227   MNTEILTIMLVVSVLMGLIGLIAFLWGVKSGQFDDEKRMLESVLYDSTSDLNEAVLQEKRQE--NHF57:HPF57_1125   MNTEILTIMLVVSVLMGLIGLIAFLWGVKSGQFDDEKRMLESVLYDSASDLNEAVLQEKRQE--NHF16:HPF16_1103   MNTEILTIMLVVSVLMGLIGLIAFLWGVKSGQFDDEKRMLESVLYDSASDLNEAVLQEKRQE--NH52:HPKB_1093     MNTEILTIMLVVSVLMGLIGLIAFLWGVKSGQFDDEKRMLESVLYDSASDLNEAILQEKRQE--NH266:HP1163       MNTEILTIMLVVSVLMGLVGLIAFLWGVKSGQFDDEKRMLESVLYDSASDLNEAILQEKRQK--NHHPA:HPAG1_1102   MNTEILTIMLVVSVLMGLVGLIAFLWGVKSGQFDDEKRMLESVLYDSASDLNEAILQEKRQK--NHP12:HPP12_1129   MNTEILTIMLVVSVLMGLVGLIAFLWGVKSGQFDDEKRMLESVLYDSASDLNEAILQEKRQK--NHB38:HELPY_1137   MNTEILTIMLVVSVLMGLVGLIAFLWGVKSGQFDDEKRMLESVLYDSASDLNEAISQEKRQK--N
