# Supplementary material for: Evolution in an oncogenic bacterial species with extreme genome plasticity: Helicobacter pylori East Asian genomes
Source: BMC Microbiol. 2011 May 16;11:104. doi: 10.1186/1471-2180-11-104 (PMC3120642; doi:10.1186/1471-2180-11-104)
Supplement: Additional file 6 — Multiple sequence alignments of diverged genes. [file 1471-2180-11-104-S6.ZIP › Diverged_genes_multiple_seuence_alignments/HP1185_sotB.mfa.rtf]

                   1         11        21        31        41        51        61        71        81        91                           |         |         |         |         |         |         |         |         |         |         HB8:mHPB8_306      -------------MRVFVFSLSAFIFNTTEFVPVALLSDIAKSFEMESATVGLMITAYAWVVSLGSLPLMLLSAKMERKRLLLFLFALFIASHILSALAWHSJM:mHPSJM_05920  -------------MRVFVFSLSAFIFNTTEFVPVALLSDIAKSFEMESATVGLMITAYAWVVSLGSLPLMLLSAKVERKRLLLFLFALFILSHILSALAWHB38:mHELPY_1158   -------------MRVFVFSLSAFIFNTTEFVPVALLSDIAKSFEMESATVGLMITAYAWVVSLGSLPLMLLSAKIERKRLLLFLFALFIASHILSALAWHP12:mHPP12_1151   -------------MRVFVFSLSAFIFNTTEFVPVALLSDIAKSFEMESATVGLMITAYAWVVSLGSLPLMLLSAKIERKRLLLFLFALFILSHILSALAWHHPA:mHPAG1_1125   -------------MRVFVFSLSAFIFNTTEFVPVALLSDIAKSFEMESATVGLMITAYAWVVSLGSLPLMLLSAKIERKRLLLFLFALFILSHILSALAWH266:HP1185        MMITKQSYQKFALMRVFVFSLSAFIFNTTEFVPVALLSDIAKSFEMESATVGLMITAYAWVVSLGSLPLMLLSAKIERKRLLLFLFALFILSHILSALAWHG27:HPG27_1128    MMITKQSYQKFALMRVFVFSLSAFIFNTTEFVPVALLSDIAKSFEMESATVGLMITAYAWVVSLGSLPLMLLSAKIERKRLLLFLFALFIASHILSALAWHF32:HPF32_1119    MMITKQSYKKLALMRVFVFSLSAFIFNTTEFVPVALLSDIAKSFEMESASVGLMITLYAWLVSLGSLPLMLFSAKIERKRLLLFLFALFILSHILSALAWH52:mHPKB_1117     MMITKQSYQKFALMRVFVFSLSAFIFNTTEFVPVALLSDIAKSFEMESASVGLMITLYAWLVSLGSLPLMLFSAKIERKRLLLFLFALFILSHILSALAWHF30:HPF30_0206    MMITKQSYQKLALMRVFVFSLSAFIFNTTEFVPVALLSDIAKSFEMESASVGLMITLYAWLVSLGSLPLMLFSAKIERKRLLLFLFALFILSHILSALAWHF16:HPF16_1124    MMMTKQSYQKFALMRVFVFSLSAFIFNTTEFVPVALLSDIAKSFEMESASVGLMITLYAWLVSLGSLPLMLLSAKIERKRLLLFLFALFILSHILSALAWHF57:HPF57_1148    MMITKQSYQKFALMRVFVFSLSAFIFNTTEFVPVALLSDIAKSFEMESASVGLMITLYAWLVSLGSLPLMLLSAKIERKRLLLFLFALFILSHILSALAWH51:mKHP_1082      -MMTKQSYQKFALMRVFVFSLSAFIFNTTEFVPVALLSDIAKSFEMESASVGLMITLYAWLVSLGSLPLMLFSAKIERKRLLLFLFALFILSHILSALAW                   101       111       121       131       141       151       161       171       181       191                          |         |         |         |         |         |         |         |         |         |         HB8:mHPB8_306      NFWVLLLSRMGIAFAHSIFWSITASLVIRVAPRNKKQQALGLLALGSSLAMILGLPLGRIIGQILDWRSTFGVIGGVATLIMLLMWKLLPHLPSKNAGTLHSJM:mHPSJM_05920  NFWVLLISRMGIAFAHSIFWSITASLVIRVAPRNKKQQALGLLALGSSLAMILGLPLGRIIGQMLDWRSTFGVIGGVATLIMLLMWKLLPHLPSRNAGTLHB38:mHELPY_1158   NFWVLLLSRMGIAFAHSIFWSITASLVIRVAPRNKKQQALGLLALGSSLAMILGLPLGRIIGQILDWRSTFGVIGGVATLIMLLMWKLLPHLPSRNAGTLHP12:mHPP12_1151   NFWVLLISRMGIAFAHSIFWSITASLVIRVAPRNKKQQALGLLALGSSLAMILGLPLGRIIGQILDWRSTFGVIGGVATLIMLLMWKLLPHLPSRNAGTLHHPA:mHPAG1_1125   NFWVLLLSRMGIAFTHSIFWSITASLVIRVAPRNKKQQALGLLALGSSLAMILGLPLGRIIGQMLDWRSTFGVIGGVATLIALLMWKLLPHLPSRNAGTLH266:HP1185        NFWVLLLSRMGIAFAHSIFWSITASLVIRVAPRNKKQQALGLLALGSSLAMILGLPLGRIIGQILDWRSTFGVIGGVATLIALLMWKLLPHLPSRNAGTLHG27:HPG27_1128    NFWVLLISRIGIAFAHSIFWSITASLVIRVAPRNKKQQALGLLALGSSLAMILGLPLGRIIGQMLDWRSTFGVIGGVATLIALLMWKLLPPLPSRNAGTLHF32:HPF32_1119    NFWVLLISRTGIALAHSVFWSITASLVIRVAPIGRKQQALGLLALGSSLAMILGLPLGRIIGQMLDWRSTFGVIGGVATLIALLMYKLLPPLPSKNAGTLH52:mHPKB_1117     NFWVLLISRAGIALAHSVFWSITASLVIRVAPIGRKQQALGLLALGSSLAMILGLPLGRIIGQMLDWRSTFGVIGGVATLIALLMYKLLPPLPSKNAGTLHF30:HPF30_0206    NFWVLLISRAGIALAHSVFWSITASLVIRVAPIGRKQQALGLLALGSSLAMILGLPLGRIIGQILDWRSTFGVIGGVATLIALLMYKLLPPLPSKNAGTLHF16:HPF16_1124    NFWVLLISRAGIALAHSVFWSITASLVIRVAPIGRKQQALGLLALGSSLAMILGLPLGRIIGQMLDWRSTFGVIGGVATLIALLMYKLLPPLPSKNAGTLHF57:HPF57_1148    NFWVLLISRIGIALAHSIFWSITASLVIRVAPIGRKQQALGLLALGSSLAMILGLPLGRIIGQILDWRSTFGVIGGVATLIALLMYKLLPPLPSKNAGTLH51:mKHP_1082      NFWVLLISRSGIALAHSIFWSITASLVIRVAPIGKKQQALGLLALGSSLAMILGLPLGRIIGQMLDWRSTFGVIGGVATLIALLMYKLLPPLPSKNAGTL                   201       211       221       231       241       251       261       271       281       291                          |         |         |         |         |         |         |         |         |         |         HB8:mHPB8_306      ASVPVLMKRPLLMGIYLLVIMVISGHFTTYSYIEPFIIQISQFSPDITTLMLFVFGLAGVVGSFLFGRLYAKNSRKFIAFAMILVICPQLLLFVFKNLEWHSJM:mHPSJM_05920  ASVPILMKRPLLVGIYLLVIMVISGHFTTYSYIEPFIIQISQFSPDITTLMLFVFGLAGVAGSFLFGRLYAKNSRKFIAFAMILVICPQLLLFVFKNLEWHB38:mHELPY_1158   ASVPILMKRPLLVGIYLLVIMVISGHFTTYSYIEPFIIQISQFSPDITTLMLFVFGLAGVAGSFLFGRLYAKNSRKFIAFAMVLVICPQLLLFVFKNLEWHP12:mHPP12_1151   ASVPVLMKRPLLVGIYLLVIMVISGHFTTYSYIEPFIIQISQFSPDITTLMLFVFGLAGVAGSFLFGRLYAKNSRKFIAFAMVLVICPQLLLFVFKNLEWHHPA:mHPAG1_1125   ASVPILMKRPLLMGIYLLVIMVISGHFTTYSYIEPFIIQISQFSPDITTLMLFVFGLAGVAGSFLFSRLYAKNSRKFIAFAMVLVICPQLLLFVFKNLEWH266:HP1185        ASVPVLMKRPLLMGIYLLVIMVISGHFTTYSYIEPFIIQISQFSPDITTLMLFVFGLAGVVGSFLFGRLYAKNSRKFIAFAMVLVICPQLLLFVFKNLEWHG27:HPG27_1128    ASVPILMKRPLLMGIYLLVIMVISGHFTTYSYIEPFIIQISQFSPDITTLMLFVFGLAGVAGSFLFGRLYAKNSRKFIAFAMILVICPQLLLFVFKNLEWHF32:HPF32_1119    SSLPVLVKRPLLMGIYLLVIMVISGHFTTYSYIEPFIIQISQFSPDITTLMLFVFGLAGVVGSFLFGRLYAKNSRKFIAFAMVLVICPQLLLFVFKNSEWH52:mHPKB_1117     SSLPVLVKRPLLMGIYLLVIMVISGHFTTYSYIEPFIIQISQFSPDITTLMLFVFGLAGVVGSFLFGRLYAKNSRKFIAFAMVLVICPQLLLFVFKNSEWHF30:HPF30_0206    SSLPVLVKRPLLMGIYLLVIMVISGHFTTYSYIEPFIIQISQFSPDITTLMLFVFGLAGVVGSFLFGRLYAKNSRKFIAFAMVLVICPQLLLFVFKNSEWHF16:HPF16_1124    SSLPVLVKRPLLMGIYLLVIMVISGHFTTYSYIEPFIIQISQFSPDITTLMLFVFGLAGVVGSFLFGRLYAKNSRKFIAFAMILVICPQLLLFVFKNSEWHF57:HPF57_1148    SSLPVLVKRPLLMGIYLLVIMVISGHFTTYSYIEPFIIQISQFSPDITTLMLFVFGLAGVVGSFLFGRLYAKNSRKFIAFAMVLVICPQLLLFVFKNSEWH51:mKHP_1082      SSLPVLVKRPLLMGIYLLVIMVISGHFTTYSYIEPFIIQISQFSPDITTLMLFVFGLAGVVGSFLFGRLYAKNSRKFIAFAMVLVICPQLLLFVFKNSEW                   301       311       321       331       341       351       361       371       381       391                   |         |         |         |         |         |         |         |         |         |HB8:mHPB8_306      VVFLQIFLWGIGITSLGISLQMRVLQLAPDATDVASAIFSGSYNVGIGSGALFGSIVIHQLGLGYIGFVGGALGLLALFWLRFITIKFKKTHSJM:mHPSJM_05920  VIFLQIFLWGIGITSLTIALQMRVLQLAPDATDVASAIFSGSYNVGIGSGALFGSIVIHQLGLGYIGFVGGALGLLALFWLRFITIKFKKTHB38:mHELPY_1158   VIFLQIFLWGIGITSLTIALQMRVLQLAPDATDVASAIFSGSYNVGIGSGALFGSIVIHQLGLGYIGFVGGALGLLALFWLRFITIKFKKTHP12:mHPP12_1151   VIFLQIFLWGIGITSLTIALQMRVLQLAPDATDVASAIFSGSYNVGIGSGALFGSIVIHQLGLEYIGFVGGALGLLALFWLRFITIKFKKTHHPA:mHPAG1_1125   VIFLQIFLWGIGITSLTIALQMRVLQLAPDATDVASAIFSGSYNVGIGSGALFGSIVIHQLGLEYIGFVGGALGLLALFWLRFITIKFKKTH266:HP1185        VVFLQIFLWGIGITSLGISLQMRVLQLAPDATDVASAIYSGSYNVGIGSGALFGSIVIHQLGLGYIGFVGGALGLLALFWLRFITIKFKKTHG27:HPG27_1128    VIFLQIFLWGIGITSLTIALQMRVLQLAPDATDVASAIFSGSYNVGIGSGALFGSIVIHQLGLGYIGFVGGALGLLALFWLRFITIKFKKTHF32:HPF32_1119    VVFLQIFLWGIGITSLGISLQMRVLQLAPDATDVASAIYSGSYNVGIGAGALFGSIVIHQLGLGYIGFVGGALGLLALFWLRFITIKFKKTH52:mHPKB_1117     VVFLQIFLWGIGITSLGISLQMRVLQLAPDATDVASAIYSGSYNVGIGAGALFGSIVIHQLGLGYIGFVGGALGLLALFWLRFITIKFKKTHF30:HPF30_0206    VVFLQIFLWGIGITSLGISLQMRVLQLAPDATDVASAIYSGSYNVGIGAGALFGSIVIHQLGLGYIGFVGGALGLLALFWLRFITIKFKKTHF16:HPF16_1124    VVFLQIFLWGVGITSLGISLQMRVLQLAPDATDVASAIYSGSYNVGIGAGALFGSIVIHQLGLGYIGFVGGALGLLALFWLRFITIKFKKTHF57:HPF57_1148    VVFLQIFLWGIGITSLGISLQMRVLQLAPDATDVASAIYSGSYNVGIGAGALFGSIVIHQLGLGYIGFVGGALGLLALFWLRFITIKFKKTH51:mKHP_1082      VVFLQIFLWGIGITSLGISLQMRVLQLAPDATDVASAIYSGSYNVGIGAGALFGSIVIHQLGLGYIGFVGGALGLLALFWLRFITIKFKKT
