# Supplementary material for: Evolution in an oncogenic bacterial species with extreme genome plasticity: Helicobacter pylori East Asian genomes
Source: BMC Microbiol. 2011 May 16;11:104. doi: 10.1186/1471-2180-11-104 (PMC3120642; doi:10.1186/1471-2180-11-104)
Supplement: Additional file 6 — Multiple sequence alignments of diverged genes. [file 1471-2180-11-104-S6.ZIP › Diverged_genes_multiple_seuence_alignments/HP1250.mfa.rtf]

                  1         11        21        31        41        51        61        71        81        91                          |         |         |         |         |         |         |         |         |         |         HB8:HPB8_230      MKTEMKSSLKLFMRPLLVVLAFMLLYALVHAALGFYVKKDSTPINPNIEKTETERQNSALSPKQEEANTTTTATEENPTKDTVPPLDTAAQ----KQETKHHPA:HPAG1_1193   MKTEMKYSLKLFAQPLLVVLAFMLLYALAHAALGFYVKKDSAPINPNVEKTETERQNSALSPKQEEANTATTATEENPTKDTAPPLETAAQ----EKETKH266:HP1250       MKTEMKSSLKLFMRPLLVVLAFMLLYALVHAALGFYVKKDSAPISPNVEKTETERQNGVLSPKQEEANATTTATEESPTKDTAPPLDTAAQ--------KHB38:HELPY_1226   MKTEMKSSLKLFMRPLLVVLAFMLLYALVHAVLGFYVKKDSAPISPNAEKTETERQNSALPPKQEEANTATTATEENPTKDTAPPLETTAQ----EKETKHG27:HPG27_1195   MKTEMKSSLKLFMRPSLVVLAFMLLYALAHAALGFYVKKDSAPISPNAEKTEIERQNSALSPKQEEANTTTTATEESPTKDTAPPLETTAQ----EKETKHP12:HPP12_1216   MKTEMKSSLKLFMRPLLVVLAFMLLYALAHAALGFYAKKDSAPISPNAEKTEMERQNSALPLKQEESNTTTTATEENPTKDTAPPLETAVQ----EKEAKHSJM:HPSJM_06250  ----MKSFLKLFAQPLLVVLAFMLLYALAHAALGFYVKKDSAPISPNVEKSETEHQNSVLSPK-EEANTTTTATEESPTKDTAPPLDTAAQ----EKETKHF32:HPF32_1180   ----MKSSLKLFMRPFLVVLGFMLLYALAHAALGFYGEKDSASISQNLEKSEIKRQSSALLPKQEEANTTTTATEENPTKDPPLPLETATQ----KQETKH51:mKHP_1146     ----MKSSLKLFVRPFLVVLGFMLLYALVHAVLGFYGEKDSTSISQNLEETEIERQNSALSPKQEEANTATTATEENPTKDPLLPLETATQKQENKQETKHF57:HPF57_1209   MKTEMKSSLKLFVRPFLVVLGFMLLYALAHAALGFYGEKDSASINQNLEKTKIERQNSTLSPKQEETNTTTTATEENPTKDSPLPLETPTQEKENKQENKHF16:HPF16_1184   MKTEMKSSLKLFVRPFLVVLGFMLLYALAHAALGFYGEKDSASISQNLEKTEIERQNSVLSPKQEETNMTTTATEENPTKDSPLPLETPTQ----KQETKHF30:HPF30_0147   MKTEMKSSLKLFMRPFLVVLGFMLLYALAHATLGFYGEKDSASISQNLEKTEIERQNSVLSPKQEEANTTTTATEENPTKDSPLPLETATQ--------KH52:HPKB_1185     MKTEMKSSLKLFMRSFLVVLGFMLLYALVHAALGFYGEKDSTSISQNLEKTEIERPNSALSPKQEEANTTTTIAEENPTKDSPLPLETPTQ----ENEPK                  101       111       121       131       141       151       161       171       181       191                         |         |         |         |         |         |         |         |         |         |         HB8:HPB8_230      QETKQEQEKENEPKQDSVSPIQNNQKTPTTPLMGKKPLEYKVAVSGVNVRAFPSTKGKIIGSLTKDKSVKVLEIQNDWAKIEFSNKTKGYVFLKLLKKAEHHPA:HPAG1_1193   QETKQEQEKENESKQDSVSPVQNNQKTLTTPTMGQKPLEYKVAVSGVNVRAFPSTKGKIIGSLAKDKSVKVLEIQNDWAKIEFSNKTKGYVFLKLLKKAEH266:HP1250       QETKQEQEKENEPKQDSVPPVQNNQKTPTTPLMGKKPLEYKVAVSGVNVRAFPSTKGKILGLLLKNKSVKVLEIQNDWAEIEFSHETKGYVFLKLLKKAEHB38:HELPY_1226   QETKQEQEKENEPKQNSVPPVQNNQKTPTTPLMGKKPLEYKVAVSGVNVRAFPSTKGKILGLFLKNKSVKVLEIQNGWAEIEFSNKTKGYVFLKLLKKAEHG27:HPG27_1195   QETKQEQEKESEPKQNSVPPVQNNQKAPTISTMGKKPLEYKVAVSGVNVRAFPSTKGKILGLLLKNKSVKVLEIQNDWAEIEFSNKTKGYVFLKLLKKAEHP12:HPP12_1216   QEIKQEQEKENEPKQDSVSPVQNNQKTPTTPLIGKKPLEYKVAVSGVNVRAFPSTKGKILGLLAKNKSVKVLEIQNDWAKIEFSNKTKGYVFLKLLKKAEHSJM:HPSJM_06250  QETKQEQEKENEPKQNSVSPVQNNQKAPTTPLIGKKPLEYKVAVSGVNVRAFPSTKGKILGLLLKNKGVKVLEIQNDWAEIEFSNKTKGYVFLKLLKKAEHF32:HPF32_1180   QETKQEQEKENESKQNSASPAQNNQKTLSSPTIGKKPLEYKVAVSGVNVRAFPSTKGKILGSLAKNKSVKVLEIQNDWAKIEFSNETKGYVFLKLLKKAEH51:mKHP_1146     QETKQEQEKENESKQNSASPIQNHQKTLSTPTIGKKPLEYKAAVNSVNVRAFPSTKGKILGSLAKNKSVKVLEIQNDWAKIEFSNETKGYVFLKLLKKAEHF57:HPF57_1209   QETKQEQEKENEPKQNSASPIQNHQKTLSTSTIGKKPLEYKAAVNSVNVRAFPSTKGKILGSLAKNKSVKVLEIQNDWAKIEFSNETKGYVFLKLLKKAEHF16:HPF16_1184   QETKQEQEKENEPKQNSASPIQNHQKTLSTSTIGKKPLEYKAAVNSVNVRAFPSTKGKILGSLAKNKSVKVLEIQNDWAKIEFSNETKGYVFLKLLKKAEHF30:HPF30_0147   QENKQEQEKENESKQNSASPTQNHQKTLSTPTIGKKPLEYKAAVNSVNVRAFPSTKGKILGSLAKNKSVKVLEIQNDWAKIEFSNETKGYVFLKLLKKAEH52:HPKB_1185     QENKQEQEKETKPKQNSASPVQNHQKTLSTPTMGKKPLEYKVAVNSVNVRAFPSTKGKILGSLAKNKSVKVLEIQNDWAKIEFSNETKGYVFLKLLKKAE                  201                  |HB8:HPB8_230      HHPA:HPAG1_1193   H266:HP1250       HB38:HELPY_1226   HG27:HPG27_1195   HP12:HPP12_1216   HSJM:HPSJM_06250  HF32:HPF32_1180   H51:mKHP_1146     HF57:HPF57_1209   HF16:HPF16_1184   HF30:HPF30_0147   H52:HPKB_1185     
