# Supplementary material for: Evolution in an oncogenic bacterial species with extreme genome plasticity: Helicobacter pylori East Asian genomes
Source: BMC Microbiol. 2011 May 16;11:104. doi: 10.1186/1471-2180-11-104 (PMC3120642; doi:10.1186/1471-2180-11-104)
Supplement: Additional file 6 — Multiple sequence alignments of diverged genes. [file 1471-2180-11-104-S6.ZIP › Diverged_genes_multiple_seuence_alignments/HP1265_nuoF.mfa.rtf]

                   1         11        21        31        41        51        61        71        81        91                           |         |         |         |         |         |         |         |         |         |         HB8:mHPB8_215      --LNGFNPLNSPLIASSSLSLKEAYYLEKLSLQKGFKIHYKMTKDSLSLLEKSDLCVLFGGFSNACLNENERLILENINQLKRPYALLRPLQDTRDLQENHF32:HPF32_1195    --LSGFNPLNSPLIASSSLSLKEAYCLEKLSFKKGFKINYKMTKDSLNLLEKSDLCVLFGGFSNACLNENERLILENINQLKLPYALLRPLQDTRDLQENHSJM:HPSJM_06325   --LSGFNPLNSPLSASSSLSLKEAYCLEKLSLQKGFKIHYKMTKDSLSLLEKSDLCVLFGGFSNACLNENERWILESINQSKRPYALLRPLQDTRDLQENHv22:mHPV225_1299  LILSGFNPLNSPLTASSSLSLKEAYCLEKLSLKKGFKINYKMTKDSLNLLEKSDLCVLFGGFSNACLNENERLILESINQLKLPYALLRPLQDTRDLQENHPeC:HPPC_06220    --LSGFNPLNSPLVASSSLSLKEAYCLEKLSLKKGFKINYKMTKDSLNLLEKSDLCVLFGGFSNACLNENERLILESINQLKLPYALLRPLQDTRDLQENH266:HP1265        --LSGFNPLNSPLVASSSLSLKEAYYLEKLSLKKGFKIHYKMTKDSLNLLEKSDLCVLFGGFSNACLNENERWILESISHSKRPYALLRPLQDTRDLQENHF57:HPF57_1224    LILSGFNPLNSPLIASSSLSLKEAYCLEKLSLKKGFKINYKMAKDSLSLLEKSDLCVLFGGFSNACLNENERLILENINQLKLPYALLRPLQDTRDLQENHF16:HPF16_1200    --LSGFNPLNSPLIASSSLSLKEAYCLEKLSLKKGFKINYKMAKDSLNLLEKSDLCVLFGGFSNACLNENERLILENINQLKLPYALLRPLQDTRDLQENHB38:HELPY_1241    --LSGFNPLNSPLIASSSLSLKEAYYLEKLSLKKGFKINYKMTKDSLNLLEKSDLCVLFGGFSNACLNENERLILENINQLKLPYALLRPLQDTRDLQENHCuz:HPCU_06465    --LSGFNPLNSPLIASSSLSLKEAYCLEKLSLKKGFKINYKMTKDSLNLLEKSDLCVLFGGFSNACLNENERLVLENINQLKLPYALLRPLQDTRDLQENHShi:mHPSH_06555   --LSGFNPLNSPLIASSSLSLKEAYCLEKLSLKKGFKINYKMTKDSLSLLEKSDLCVLFGGFSNACLNENERLILESINQLKLPYALLRPLQDTRDLQENH908:hp908_1265    --LSGFNPLNSPLSASSSISLKEAYCLEKLSLQKGFKINYKLSEDSLNLLKKSDLCVLFGGFSNACLNENERWVLGSINQSKRPYALLRPLQDTRDLQENHHPA:HPAG1_1209    --LSGFNPLNSPLIASSSISLKEAYYLEKLSLKKGFKINYKMTKDSLNLLEKSDLCVLFGGFSNACLNENERLILENINQSKRPYALLRSLQDTRDLQENHP12:HPP12_1231    --LSGFNPFNSPLIASSSLSLKEAYYLEKLSLKKGFKISYKMTKDSLNLLEKSDLCVLFGGFSNACLNENERLILGSINQSKHPYALLRPLQDTRDLQENH51:KHP_1161       --LSGFNPLNSPLIASSSLSLKEAYCLEKLSLKKGFKINYKMAKDSLSLLEKSDLCVLFGGFSNACLNENERLILENINQLKLPYALLRPLQDTRDLQENHSat:HPSAT_06115   --LSGFNPLNSPLIASSSLSLKEAYCLEKLSLKKGFKINYKMTKDSLSLLEKSDLCVLFGGFSNACLNENERLILESINQLKLPYALLRPLQDTRDLQENHJ99:jhp1186       --LSGFNPLNSPLSASSSISLKEAYCLEKLSLQKGFKINYKLSEDSLNLLEKSDLCVLFGGFSNACLNENERWILESINQSKRPYALLRPLQDTRDLQENH52:HPKB_1201      --LSGFNPLNSPLIASSSLSLKEAYCLEKLSLKKGFKINYKMTKDSLNLLEKSDLCVLFGGFSNACLNENERLILESINQLKLPYALLRPLQDTRDLQENHG27:HPG27_1210    --LSGFNPLNSPLSASSSISLKEAYCLEKLSLKKGFEINYKLSEDSLNLLKKSDLCVLFGGFSNACLNENERLILENINQLKLPYALLRPLQDTRDLQENHF30:HPF30_0132    LILSGFNPLNSPLIASSSLSLKEAYCLEKLSLKKGFKINYKMTKDSLSLLEKSDLCVLFGGFSNACLNENERLILENINQLKLPYALLRPLQDTRDLQEN                   101       111       121       131       141       151       161       171       181       191                          |         |         |         |         |         |         |         |         |         |         HB8:mHPB8_215      CLFASYEIHTEAAILALILRGILEKTSQLKGHVLEKVDVGYLSSEANMSEEELQELIALIIKAKKRALVLNREITKHADSAFLYTLLSELQNYLEILHIPHF32:HPF32_1195    CLFASYEINTEAAVLALILRGILEKTSRLKGHVLEDVDVGYLSSEANMSEEELQELIALIIKAKKRVLVLNREITKHADSAFLYTLLSELQNHLEILHIPHSJM:HPSJM_06325   CLFASYEIHTEAAILALILRGILEKTSQLKGHVLEKIDVGYLSSEANMSEEELQELIALIVKAKKRALVLNREITKHAHSTFLYTLLSGLQNYLEILHIPHv22:mHPV225_1299  CLFASYEINTEAAVLALILRGILEKTSQLKGHVLEDVDVGYLSSEANMSEEELQELIALIIKAKKRVLVLNREITKHADSAFLYTLLSELQNHLEILHIPHPeC:HPPC_06220    CLFASYEINTEAAVLALILRGILEKTSQLKGHVLEDVDVGYLSSEANMSEEELQELIALIIKAKKKVLVLNREITKHADSAFLYTLLSELQNHLEILHIPH266:HP1265        CLFASYEIHTEAAILALILRGILEQTSQLKGHVLEKIDVGYLSSEANMSEEELQELIALIVKAKKRALVLNREITKHANNAFLYTLLSELQNYLEILHIPHF57:HPF57_1224    CLFASYEINTEVAILALILRGILEKTSRLKGHVLEDVDVGYLSSEANMSEEELQELIALIIKAKKRVLVLNREITKHADSAFLYTLLSELQNHLEILHIPHF16:HPF16_1200    CLFASYEINTEAAILALILRGILEKTSRLKGHVLEDVDVGYLSSEANMSEEELQELIALIIKAKKRVLVLNREITKHADSAFLYTLLSELQNHLEILHIPHB38:HELPY_1241    CLFASYEINTEAAILALILRGILEKTSQLKGHVLEKIDVGYLSSEANMSEEELQDLIALIVKAKKRALVLNREITKHAHSAFLYTLLSELQNYLEILHIPHCuz:HPCU_06465    CLFASYEINTEAAVLALILRGILEKTSQLKGHVLEDVDVGYLSSEANMSEEELQELIALIIKAKKKVLVLNREITKHADSAFLYTLLSELQNHLEILHIPHShi:mHPSH_06555   CLFASYEINTEAAVLALILRGILEKTSQLKGHVLEDVDVGYLSSEANMSEEELQELIALIIKAKKKVLVLNREITKHADSAFLYTLLSELQNHLEILHIPH908:hp908_1265    CLFASYEIHTEAAILALILRGILEQTSQLKGHVLENVDVGYLSSEANMSEEELQELIVLIVKAKKGALVLNREITKHVDSTFLYTLLSGLQNYLEILHIPHHPA:HPAG1_1209    CLFASYEIHTEAAILALILRGILEKTSQLKGHVLEKVDVGYLSSEANMSEEELQELIALIIKAKKRALVLNREITKHADNAFLYTLLNELQNYLEILHIPHP12:HPP12_1231    CLFASYEIHTEAAVLALILRGILEQTSRLKGHVLEKIDVGYLSSEANMSEEELQELIALIIKAKKRALVLNREITKHADSTFLYTLLSELQNYLEILHIPH51:KHP_1161       CLFASYEINTEAAILALILRGILEKTSQLKGHVLEGVDVGYLSSEANMSEEELQELIALIIKAKKRVLVLNREITKHADSAFLYTLLSELQNHLEILHIPHSat:HPSAT_06115   CLFASYEINTEAAVLALILRGILEKTSQLKGHALEDIDVGYLSSEANMSEEELQELIALIIKAKKRVLVLNREITKHADSAFLYTLLSELQNHLEILHIPHJ99:jhp1186       CLFASYEIHTEAAILALILRGILEQTSQLKGHVLEKIDVGYLSSEANMSEEELQELIALIVKAKKRVLVLNREITKHANNAFLYTLLSGLQNYLEILHIPH52:HPKB_1201      CLFASYEINTEAAILALILRGILEKTSRLKGHVLENVDVGYLSSEANMSEEELQELIALIIKAKKRALVLNREITKHADSAFLYTLLSELQNHLEILHIPHG27:HPG27_1210    CLFASYEIHTEAAILALILRGILEKTSQLKGHILEKIDVGYLSSEANMSEEELQELIALIIKAKKRALVLNREITKHADSAFLYTLLSGLQNYLEILHIPHF30:HPF30_0132    CLFASYEINTEAAILALILRGILEKTSQLKGHVLEDIDVGYLSSEANMSEEELQELIALIIKAKKKVLVLNREITKHADSAFLYTLLSELQNHLEILHIP                   201       211       221       231       241       251       261       271       281       291                          |         |         |         |         |         |         |         |         |         |         HB8:mHPB8_215      CNDSNATTAFYDFKDQEWLLETAFKEGILPFESQLKSKDLELLERMGEANGSFVYVSYKSLKTPKLSFSKQFKIANKIEHSKAGFQISNKTLECELEESPHF32:HPF32_1195    CNNSNAMAAFYDSKDQEWLLETALKEGILPFESQLQSKDLELLERISEANGSFVYVSYKSLETPRLSFSKQFKIANKIEHSKAVFQISNKTLECELEESPHSJM:HPSJM_06325   CNDSNATTAFYDSKDQEWLLETAFKEGILPFESELQSKDLELLERMGEANGSFVYVSYKSLETPRLSFSKQFKIANRIQHSKAKFQISNQTLECELEESPHv22:mHPV225_1299  CNNSNATPTFYDFKDQEWLLETTLKEGILPFESQLKSKDLELLERMSEANGSFVYVSYKSLEPPRLSFSKQFKIANKIQHSKAVFQISNKALECELEESPHPeC:HPPC_06220    CNHSNATPAFYDSKDQEWLLETALKEGILPFESQLKSKDLELLERMSEANGSFVYVSYKSLEPPRLSFSKQFKIANKIEHSKAKFQISNKALECELEESPH266:HP1265        CYDSSATTAFYDFKDQEWLLETAFKEGILPFKSQLQSKDLELLERISEANGSFVYVSYKSLETPKLSFSKQFKIANKIEHSKAGFQISNQTLECELEENPHF57:HPF57_1224    CKDSNATPTFYDFKDQEWLLETALKEGILPFESQLQSKDLELLERMSEANGSFAYISYKSLETPRLSFSKQFKIANKIQHSKAGFQISNKTLECELEESPHF16:HPF16_1200    CNNSNATAAFYDSKNQEWLLETALKEGILPFESQLQSKDLELLERMSEANGSFVYVSYKSLETPRLSFSKQFKIANKIQHSKAGFQISNKTLECELEESPHB38:HELPY_1241    CYDSSATTAFYDFKDQEWLLETAFKEGILPFESELQSKDLELLERMGEANGSFVYVSYKSLETPKLSFSKQFKIANKIKHSKAKFQILDKTLECELEESPHCuz:HPCU_06465    CNHSNATPTFYDFKDQEWLLETALKEGILPFESQLKSKDLELLERMSEANGSFVYVSYKSLETPRLSFSKQFKIANKIEHSKAGFQISNKTLECELEESPHShi:mHPSH_06555   CNNSNATPTFYDSKDQEWLLETALKEGILPFESQLKSKDLELLERMGEANGSFVYVSYKSLETPRLSFSKQFKIANKIQHSKAGFQISNKTLECELEESPH908:hp908_1265    CNDSSATTAFYDSKDQEWLLETALKEGVLPFESEL--KDLESLEQISEANGSFVYVSYKSLKTPKLSFSKQFKIANKIKHSKAVFQISNQTLECELEESPHHPA:HPAG1_1209    CNDSNATTAFYDFKDQEWLLETAFKEGILPFKSQLQSKDLEFLERMGEANGSFVYVSYKSLETPKLSFSKQFKIANKIQHSKAVFQILNKTLECELEESPHP12:HPP12_1231    CNDSSATTAFYDSKDQEWLLETAFKEGILPFESEL--KDLESLEQISEANGSFVYVSYKSLKTPKLSFSKQFKIANKIKHSKAKFQISNQTLECELEESPH51:KHP_1161       CNNSNAMAAFYDSKDQEWLLETALKEGILPFESQLQSKDLELLERISEANGSFVYASYKSLETPRLSFSKQFKIANKIQHSKAEFQISNKTLECELEESPHSat:HPSAT_06115   CNNSNATPTFYDSKDQEWLLETALKEGILPFESQLKSKDLELLERMGEANGSFVYVSYKSLETPRLSFSKQFKIANKIEHSKAGFQISNKALECELEESPHJ99:jhp1186       CNDSNPTVAFYDSKDQEWLLETAFKEGILPFESQLQSKDLELLERMGEANGSFVYVSYKSLKTPKLSFSKQFKIANKIKHSKAKFQILNQTLECELEESPH52:HPKB_1201      CNNSNATPTFYDSKDQEWLLETALKESILPFESQLQSKDLELLERISEANGSFVYVSYKSLETPRLSFSKQFKIANKIQHSKAEFQISNKTLECELEESPHG27:HPG27_1210    CNDSGATAAFYDSKDQEWLLETALKEGILPFESQLKSKDLELLERMGEANGSFVYVSYKSLETPKLSFSKQFKIANKIQHSKAGFQISNQTLECELEESPHF30:HPF30_0132    CNNSNATAAFYDSKDQEWLLETALKEGILPFESQLQSKDLELLERMSEANGSFVYISYKSLETPRLSFSKQFKIANKIEHSKAVFQISNKTLECDLEESP                   301       311       321       331                   |         |         |         |HB8:mHPB8_215      HLKGLIAILEGAFFDAYPYIPILSHSQGISHF32:HPF32_1195    HLKGLIAILEGAFFDAYPYIPILSHSQGISHSJM:HPSJM_06325   HLKGLIAILEGAFFDAYPYIPILSHSQGISHv22:mHPV225_1299  HLKGLIAILEGAFFDAYPYIPILSHSQGISHPeC:HPPC_06220    HLKGLIAILEGAFFDAYPYIPILSHSQGISH266:HP1265        HLKGLIAILEGAFFDAYPYIPILSHSQGISHF57:HPF57_1224    HLKGLIAILEGAFFDAYPYIPILSHSQGISHF16:HPF16_1200    HLKGLVAILEGAFFDAYPYIPILSHSQGISHB38:HELPY_1241    HLKGLIAILEGAFFDAYPYIPILSHSQGISHCuz:HPCU_06465    HLKGLIAILEGAFFDAYPYIPILSHSQGISHShi:mHPSH_06555   HLKGLIAILEGAFFDAYPYIPILSHSQGISH908:hp908_1265    HLKGLIAILEGAFFDTYPYIPILSHSQGISHHPA:HPAG1_1209    NLKGLIAILEGAFFDAYPYIPILSHSQGISHP12:HPP12_1231    NLKGLIAILEGAFFDTYPYIPILSHSQGISH51:KHP_1161       HLKGLIAILEGAFFDAYPYIPILSHSQGISHSat:HPSAT_06115   HLKGLIAILEGAFFDAYPYIPILSHSQGISHJ99:jhp1186       HLKGLIAILEGAFFDTYPYIPILSHSQGISH52:HPKB_1201      HLKGLIAILEGAFFDAYPYIPILSHSQGISHG27:HPG27_1210    YLKGLIAILEGAFFDAYPYIPILSHSQGISHF30:HPF30_0132    HLKGLIAILEGAFFDAYPYIPILSHSQGIS
