# Supplementary material for: Evolution in an oncogenic bacterial species with extreme genome plasticity: Helicobacter pylori East Asian genomes
Source: BMC Microbiol. 2011 May 16;11:104. doi: 10.1186/1471-2180-11-104 (PMC3120642; doi:10.1186/1471-2180-11-104)
Supplement: Additional file 6 — Multiple sequence alignments of diverged genes. [file 1471-2180-11-104-S6.ZIP › Diverged_genes_multiple_seuence_alignments/HP1468_ilvE.mfa.rtf]

                  1         11        21        31        41        51        61        71        81        91                          |         |         |         |         |         |         |         |         |         |         HB8:HPB8_63       MANLENLDWKNLGFSYIKTDFRFIATYKNGSWSQGELVSENVLQLSEGSPVLHYGQACFEGLKAYRSQKGKALLFRPLENAKRLQTSCERLLMPKVSEELHG27:HPG27_1391   MANLENLDWKNLGFSYIKTDFRFIASYKNGSWSQGELVSENVLQLSEGSPVLHYGQACFEGLKAYRSQKGKALLFRPLENAKRLQTSCERLLMPKVSEELHSJM:HPSJM_07485  MANLENLDWKNLGFSYIKTDFRFIATYKNGSWSQGELVSENVLQLSEGSPVLHYGQACFEGLKAYRSQKGKALLFRPLENAKRLQTSCERLLMPKVSEELH266:HP1468       MANLENLDWKNLGFSYIKTDFRFIATYKNGSWSQGGLVSENMLQLSEGSPVLHYGQACFEGLKAYRSQKGKALLFRPLENAKRLQTSCERLLMPKVSEELHB38:HELPY_1440   MANLENLDWKNLGFSYIKTDFRFIATYKNGSWSHGGLVSENVLQLSEGSPVLHYGQACFEGLKAYRSQKGKALLFRPLENAKRLQTSCERLLMPKVSEELHP12:HPP12_1446   MANLENLDWKNLGFSYIKTDFRFIATYKTGSWSHGELVSENVLQISEGSPVLHYGQACFEGLKAYRSQKGEALLFRPLENAKRLQTSCERLLMPKVSEELHHPA:HPAG1_1447   MANLENLDWKNLGFSYIKTDFRFIATYKNGSWSHGELVSENVLQLSEGSPVLHYGQACFEGLKAYRSQKGKALLFRPLENAKRLQTSCERLLMPKVSEELHF32:HPF32_1355   MANSGNLDWKNLGFSYIKTDFRFIAAYKNGSWSHGELVSENALQISEGSPVLHYGQACFEGLKAYRSQKGKALLFRPLENAKRLQTSCERLLMPKVSEELHF57:HPF57_1383   MANSGNLDWKNLGFSYIKTDFRFIASYKNGSWSQGELVSENALQISEGSPVLHYGQACFEGLKAYRSQKGKALLFRPLENAKRLQTSCERLLMPKVSEELHF16:HPF16_1364   MANSENLDWKNLGFSYIKTDFRFIASYKNGSWSQGELVSENALQISEGSPVLHYGQACFEGLKAYRSQNGKALLFRPLENAKRLQTSCERLLMPKVSEELHF30:HPF30_1335   MANLENLDWKNLGFSYIKTDFRFIASYKNGSWSQGELVSENALQISEGSPVLHYGQACFEGLKAYRSQNGKALLFRPLENAKRLQTSCERLLMPKVSEELH52:HPKB_1370     MANSGNLDWKNLGFSYIKTDFRFIASYKNGSWSQGELVSKNALQISEGSPVLHYGQACFEGLKAYRSQKGKALLFRPLENAKRLQTSCERLLMPKVSEELH51:KHP_1319      MANLENLDWKNLGFSYIKTDFRFIASYKNGSWSQGELVGENALQISEGSPVLHYGQACFEGLKAYRSQNGKALLFRPLENAKRLQTSCERLLMPKVSEEL                  101       111       121       131       141       151       161       171       181       191                         |         |         |         |         |         |         |         |         |         |         HB8:HPB8_63       FLRACAEVVKANQKWLAPYKSGASLYLRPFVIGVGDNLGVKPANEYLFIVFCAPVGAYFKGGIEKGGARFITTIFDRAAPKGTGGVKVGGNYAASLLAHKHG27:HPG27_1391   FLRACTEVVKANQKWLAPYKSGASLYLRPFVIGVGDNLGVKPANEYLFIVFCAPVGAYFKGGIEKGGARFITTAFDRAAPKGTGGVKVGGNYAASLLAHKHSJM:HPSJM_07485  FLRACAEVVKANQKWLAPYKSGASLYLRPFVIGVGDNLGVKPASEYLFIVFCAPVGAYFKGGIEKGGARFITTAFDRAAPKGTGGVKVGGNYAASLLAHKH266:HP1468       FLRACAEVVKANQKWLAPYKSGASLYLRPFVIGVGDNLGVKPANEYLFIVFCAPVGAYFKGGIEKGGARFITTIFDRAAPKGTGGVKVGGNYAASLLAHKHB38:HELPY_1440   FLKACAEVIKANQKWLAPYKSGASLYLRPFVIGVGDNLGVKPANEYLFIVFCAPVGAYFKGGIEKGGARFITTIFDRAAPKGTGGVKVGGNYAASLLAHKHP12:HPP12_1446   FLRACAEVVKANQKWLAPYKSGASLYLRPFVIGVGDNLGVKPASEYLFIVFCAPVGAYFKGGIEKGGARFITTIFDRAAPKGTGGVKVGGNYAASLLAHKHHPA:HPAG1_1447   FLRACAEVVKANQKWLAPYKSGASLYLRPFVIGVGDNLGVKPANEYLFIVFCAPVGAYFKGGIEKRGARFITTAFDRAAPKGTGGVKVGGNYAASLLAHKHF32:HPF32_1355   FLRACAEVVKANQKWLAPYKSGASLYLRPFVIGVGDNLGVKPASEYLFIVFCVPVGAYFKGGIEKGGARFITTAFDRAAPKGTGGVKVGGNYAASLLAHKHF57:HPF57_1383   FLRACAEVVKANQKWLAPYKSGASLYLRPFVIGVGDNLGVKPASEYLFIVFCAPVGAYFKGGIEKGGARFITTAFDRAAPKGTGGVKVGGNYAASLLAHKHF16:HPF16_1364   FLRACAEVVKANQKWLAPYKSGASLYLRPFVIGVGDNLGVKPASEYLFIVFCAPVGAYFKGGIEKGGARFITTAFDRAAPKGTGGVKVGGNYAASLLAHKHF30:HPF30_1335   FLRACTEVVKANQKWLAPYKSGASLYLRPFVIGVGDNLGVKPASEYLFIVFCAPVGAYFKGGIEKGGARFITTAFDRAAPKGTGGVKVGGNYAASLLAHKH52:HPKB_1370     FLRACAEVVKANQKWLAPYKSGASLYLRPFVIGVGDNLGVKPASEYLFIVFCAPVGAYFKGGIEKGGATFITTAFDRAAPKGTGGVKVGGNYAASLLAHKH51:KHP_1319      FLRACAEVVKANQKWLAPYKSGASLYLRPFVIGVGDNLGVKPASEYLFIVFCAPVGAYFKGGIEKGGARFITTAFDRAAPKGTGGVKVGGNYAASLLAHK                  201       211       221       231       241       251       261       271       281       291                         |         |         |         |         |         |         |         |         |         |         HB8:HPB8_63       MATDQGYDDCIYLDPATHTKIEEVGAANFFGITHDNAFITPHSPSILPSVTRKSLMVLAKEYLKLNIEEREILMDELGAFREAGACGTAAIITPIKEIAHHG27:HPG27_1391   MAVEQGYDDCIYLDPATHTKIEEVGAANFFGITHDNAFITPYSPSILPSVTRKSLMALAKEYLKLKVEEREILMDELGAFKEAGACGTAAIITPIKEIAHHSJM:HPSJM_07485  MATEQGYDDCIYLDPTTHTKIEEVGAANFFGITHDNAFITPYSPSILPSVTRKSLMVLAKEHLKLKVEEREILMDELDAFKEAGACGTAAIITPIKEIAHH266:HP1468       MATEQGYDDCIYLDPTTHTKIEEVGAANFFGITHDDAFITPHSPSILPSITKKSLMVLAKEYLNLKVEEREILMDELDAFKEAGACGTAAIITPIKEIVHHB38:HELPY_1440   MATEQGYDDCIYLDPTTHTKIEEVGAANFFGITHDNAFITPHSPSILPSVTRKSLMALAKEYLNLKVEEREILMDELDAFKEAGACGTAAIITPIKEITHHP12:HPP12_1446   MAVEQGYDDCIYLDPATHTKIEEVGAANFFGITRDNAFITPHSPSILPSVTRKSLMVLAKEYLNLKVEEREILMDELDAFKEAGACGTAAIITPIKEITHHHPA:HPAG1_1447   MAVEQGYDDCIYLDPATHTKIEEVGAANFFGITHDNAFITPHSPSILPSITKKSLMVLAKEYLNLKVEEREILMDELDAFKEAGACGTAAIITPIKEIAHHF32:HPF32_1355   IATEQGYDDCIYLDPATHTKIEEVGAANFFGITHDNAFITPHSPSILPSITKKSLMVLAKEYLNLKVEEREILMDELGAFREAGACGTAAIITPIKEIAHHF57:HPF57_1383   IATEQGYDDCIYLDPATHTKIEEVGAANFFGITHDNAFITPHSPSILPSITKKSLMVLAKECLNLKVEEREILMDELGAFKEAGACGTAAIITPIKEIVHHF16:HPF16_1364   IATEQGYDDCIYLDPATHTKIEEVGAANFFGITHDNAFITPHSPSILPSITKKSLMVLAKECLNLKVEEREILMDELGAFKEAGACGTAAIITPIKEIAHHF30:HPF30_1335   IATEQGYDDCIYLDPATHTKIEEVGAANFFGITHDNAFITPHSPSILPSITKKSLMVLAKECLNLKVEEREILMDELGAFREAGACGTAAIITPIKEITHH52:HPKB_1370     IATEQGYDDCIYLDPTTHTKIEEVGAANFFGITHDNAFITPHSPSILPSITKKSLMVLAKECLNLKVEEREILMDELGAFKEAGACGTAAIITPIKEIAHH51:KHP_1319      IATEQGYDDCIYLDPTTHTKIEEVGAANFFGITHDNAFITPHSPSILPSITKKSLMVLAKEYLNLKVEEREILMDELGAFKEAGACGTAAIITPIKEIAH                  301       311       321       331       341                  |         |         |         |         |HB8:HPB8_63       NNKSYFFEAPGHITKQLYDLLLSIQQGEQEAPKDWIFEVGHG27:HPG27_1391   NNKSYFFEAPGHITKQLYDLLLSIQQGEQEAPKDWIFEVGHSJM:HPSJM_07485  NNKSYFFETPGHITKRLYDLLLSIQQGEQEAPKDWIFEVGH266:HP1468       NNKSYFFEAPGHITKRLYDLLLSIQQGEQEAPKDWIFEVGHB38:HELPY_1440   NNKSYFFEAPGHITKQLYDLLLSIQQGEQEAPKDWIFEVGHP12:HPP12_1446   NNKSYFFEAPGHITKQLYDLLLSIQQGEQEAPKDWIFEVGHHPA:HPAG1_1447   NNKSYFFETPGHITKQLYDLLLSIQQGEQKAPKDWIFEVGHF32:HPF32_1355   NNKSYFFEAPGNTTKQLYDLLLSIQQGEQEAPKDWIFEVCHF57:HPF57_1383   NNKSYFFEAPGHTTKQLYDLLLSIQQGEQEAPKDWIFEVCHF16:HPF16_1364   NNKSYFFEAPGHTTKQLYDLLLSIQQGEQEAPKDWIFEVCHF30:HPF30_1335   HNKSYFFEAPGHTTKQLYDLLLSIQQGEQEAPKDWIFEVCH52:HPKB_1370     NNKSYFFEAPGNTTKQLYDLLLSIQQGEQEAPKDWIFEVCH51:KHP_1319      NNKSYFFEAPGHTTKQLYDLLLSIQQGEQEAPKDWIFEVC
