# Supplementary material for: Evolution in an oncogenic bacterial species with extreme genome plasticity: Helicobacter pylori East Asian genomes
Source: BMC Microbiol. 2011 May 16;11:104. doi: 10.1186/1471-2180-11-104 (PMC3120642; doi:10.1186/1471-2180-11-104)
Supplement: Additional file 6 — Multiple sequence alignments of diverged genes. [file 1471-2180-11-104-S6.ZIP › Diverged_genes_multiple_seuence_alignments/HP1527_comH.mfa.rtf]

                  1         11        21        31        41        51        61        71        81        91                          |         |         |         |         |         |         |         |         |         |         HB8:HPB8_2        MKKTLCLSFFLTFSNPLQALVIELLEEIKTSPHKGTFKAKVLDSKEPRQVLGVYNISPHKKLTLTITHISTAIVYQPLDEKLSLETTLNPNRPTIPRNTQHSJM:HPSJM_07795  MKKSLCLSFFLTFSNPLQALVIELLEEIKTSPHKGTFKAKVLDSKEPRQVLGVYNISPHKKLTLTITHISTAIVYQPLDEKLSLETTLNPNRPTIPRNTQHB38:HELPY_1499   MKKSLCLSFFLTFSNPLQALVIELLEEIKTSPHKGTFKAKVLDSKEPRQVLGVYNISPHKKLTLTITHISTAIVYQPLDEKLSLETTLNPNRPTIPRNTQHG27:HPG27_1449   MKKSLCLSFFLTFSNPLQALVIELLEEIKTSPHKGTFKAKVLDSKEPRQVLGVYNISPHKKLTLTITHISTAIVYQPLDEKLSLETTLNPNRPTIPRNTQH266:HP1527       MKKSLCLSFFLTFSNPLQALVIELLEEIKTSPHKGTFKAKVLDSKKPRQVLGVYNISPHKKLTLTITHISTAIVYQPLDEKLSLETTLNPNRPTIPRNTQHHPA:HPAG1_1388   MKKSLCLSFFLTFSNPLQALVIELLEEIKTSPHKGTFKAKVLDSKEPRQVLGVYNISPHKKLTLTITHISTAIVYQPLDEKLSLETTLNPSRPAIPRNTQHP12:HPP12_1502   MKKTLCLSFFLTFSNPLQALVIELLEEIKTSPHKGTFKAKVLDSKEPKQVLGVYNISPHKKLTLTITHISTAIVYQPLDEKLSLETTLNPNRPTIPRNTQHF32:HPF32_1413   MKKTLCLSFFLTFSNPLQALVIELLEEIKTSPHKGTFKAKILDSKEPRQVLGVYNISPHKKLTLTITHISTAIVYQPLDEKLSLETTLNPNRPTIPRNTQH52:HPKB_1429     MKKTLCLSFFLTFSNPLQALVIELLEEIKTSPHKGTFKAKVLDSKEPRQVLGVYNISPHKKLTLTITHISTAIVYQPLDEKLSLETTLNPNRPTIPRNTQHF57:HPF57_1440   MKKTLCLSFFLTFSNPLQALVIELLEEIKTSPHKGTFKAKVLDSKEPRQVLGVYNISPHKKLTLTITHISTAIVYQPLDEKFSLETTLNPNRPTIPRNTQH51:KHP_1379      MKKTLCLSFFLTFSNPLQALVIELLEEVKTSPHKGTFKAKVLDSKEPRQVLGVYNISPHKKLTLTITHISTAIVYQPLDEKLSLEMILNPNRPTIPRNTQHF30:HPF30_1399   MKKTLCLSFFLTFSNPLQALVIELLEEIKTSPHKGTFKAKVLDSKEPRQVLGVYNISPHKKLTLTITHISTAIVYQPLDEKLSLETTLNPNRPTIPRNTQHF16:HPF16_1422   MKKTLFLSFFLTFSNPLQALVIELLEEIKTSPHKGTFKAKVLDSKEPRQILGVYNISPHKKLTLTITHISTAIVYQPLDEKLSLETTLNPNRPTIPRNTQ                  101       111       121       131       141       151       161       171       181       191                         |         |         |         |         |         |         |         |         |         |         HB8:HPB8_2        IVFSSKELKEAHAHQMPSLNAPMQKPQNKPSSSQQPSQNFSYPEPKLGSKNSKNSLLQPLATPSKISPTNEVKTPTNDTKPPLKHSSEDQENNLFITPPTHSJM:HPSJM_07795  IVFSSKELKEAHPHQMPSLNAPMQKPQNKPSSSQQSPQNFSYPEPKLGSKNSKNSLLQPLAIPSKISPTNEVKTPTTDTKPPLKHSSEDQENNLFITPPTHB38:HELPY_1499   IVFSSKELKEAHAHQMPSLNAPMQKPQNKPSSSQQSPQNFSYPEPKLGSKNSKNSLLQPLAIPSKISPTNETQTPTNDTKPPLKHSSEDQENNLFITPPTHG27:HPG27_1449   IVFSSKELKEAHAHQMPSLNAPMQKPQNKPSSSQQPPQNFSYPESKLGSKNSKNSLLQPLAIPSKISPTNETQTPTNDTKPPLKHSSEDQENNLFITPPTH266:HP1527       IVFSSKELKESHPHQMPSLNAPMQKPQNKPHSSQQPSQNFSYPEPKLGSKNSKNSLLQPLAIPSKISPTNETQTPTNDTKPPLKHSSEDQESNLFITPPTHHPA:HPAG1_1388   IVFSSKELKELHPHQMPSLNAPMQKPQNKPHSSQQPPQNFSYPEPKLGSKNSKNSLLQPLAIPSKISPTNETQTPTNDTKPPLKHSSEDQENNLFITPPTHP12:HPP12_1502   IVFSSKELKESHAHQMLSLNAPMQKPQNKPSSSQQPPQNFSYPEPKLGSKNSKNSLLQPLAIPSKISPTNKTQTPTNDTKPPLKHSSEDQENNLFITPPTHF32:HPF32_1413   IVFSSKELKESHPHQMPSLNAPIQKPQNKPTSSQQSLQNFSYPESKLGSKNPKNSLLQPLATPNKISSANEVKTPTNDTKPPLKHSSEDQENNLFVAPPTH52:HPKB_1429     IVFSSKELKESHPHQMPSLNAPIQKPQNKPTPSQQSLQNFSYTESKLGSKNPKNSLLQPLATPNKISSANEVKTPTNDTKPPLKHSSEDQENNLFITPPTHF57:HPF57_1440   IVFSSKELKEPHSHQMPSLNAPIQKPQNKPTSSQQSLQNFPYTESKLGSKNPKNSLLQPLATPNKMSSANEVKTPTNDTKPPLKHSSEDQENNLFIAPPTH51:KHP_1379      IVFSSKELKE--PHQMPSLNAPIQKPQNKPTSSQQSLQNFPYTESKLGSKNPKNSLLQPLATPNKISSANEVKTPTNDTKPPLKHSSEDQENNLFVAPPTHF30:HPF30_1399   IVFSSKELKESHPHQMPSLNAPMQKPQNKPNSSQQSLQNFSYPESKLGSKNPKNSLLQPLATPNKISSANEVKAPTNDTKPPLKHFSEDQENNLFITPPTHF16:HPF16_1422   IVFSSKELKEPHPHQMPSLNAPIQKPQNKPNSSQQSIQNFPYTESKLGSKNPKNSLLQPLATPNKISSTNEVKTPTNDTKPPLKHSSEDQENNLFITPPT                  201       211       221       231       241       251       261       271       281       291                         |         |         |         |         |         |         |         |         |         |         HB8:HPB8_2        EKTLPNDTSNADINESNESNENRDNVEKQAVRDANVKEFACGKWVYDDENLQAYRPSILKRVDEDKQTTTDITPCDYSTAENKSGKITTPYTKISVHKTEHSJM:HPSJM_07795  EKTLPNNTSNADINENNESNENRDNVEKQAVRDANVKEFACGKWVYDDENLQAYRPSILKRVDEDRQTATDITPCDYSTAENKSGKIITPYTKISIHKTEHB38:HELPY_1499   EKTLPNDTSNADINENNESNENRDNVEKQAIRDANVKEFACGKWVYDDENLQAYRPSILKRVDEDKQTATDITPCDYSTAENKSGKIITPYTKISVHKTEHG27:HPG27_1449   EKTLPNNTSNADSNENNENNENRDNVEKQAIRDANIKEFACGKWVYDDENLQAYRPSILKRVDADKQTATDITSCDYSTAENKSGKIITPYTKISVHKTEH266:HP1527       EKTLPNNTSNADISENNESNENKDNVEKQAIRDANIKEFACGKWVYDDENLQAYRPSILKRVDEDKQTATDITPCDYSTAENKSGKIITPYTKISVHKTEHHPA:HPAG1_1388   EKTLPNNTPNADISENNESNENKDNVEKQAIRDPNIKEFACGKWVYDDENLQAYRPSILKRVDEDKQTATDITPCDYSHAENKSGKITTPYTKISVHKTEHP12:HPP12_1502   EKTLPNNTPNADI---NESNENKDNVEKQAIRDPNIKEFACGKWVYDDENLQAYRPSILKRVDEDKQTATDITPCDYSHAENKSGKIITPYTKISVHKTEHF32:HPF32_1413   EKTLPNNNTNANINEHHESNENRDNGEKQAIRDPNIKEFACGKWVYDDENLQAYRPSILKRVDEDKQTATDITPCDYSNAENKSGKITTPYTKISVHKTEH52:HPKB_1429     EKTLPNNTPNANINEHDESNENRDNGEKQAIRDPNIKEFACGKWVYDDENLQAYRPSILKRIDEDKQTATDITPCDYSNAENKSGKITTPYTKISVHKTEHF57:HPF57_1440   EKTLPNNTPNANINEHNESNENRDNVEKQAIRDPNVKEFACGKWVYDDENLQAYRPSILKRIDEDKQTATDITPCDYSNAENKSGKITTPYTKISVHKTEH51:KHP_1379      EKTLPNNTPNANINEHNESNENRDSVEKQAIRDPNVKEFACGKWVYDDENLQAYRPSILKRVDEDKQTATDITPCDYSNAENKSGKITTPYTKISVHKTEHF30:HPF30_1399   EKTLPNNTPNANINEHNESNENRDSVEKQAIRDPNVKEFACGKWVYDDENLQAYRPSILKRIDEDKQTATDITPCDYSNAENKSGKITTPYTKISVHKTEHF16:HPF16_1422   EKTLPNNTPNANINEHNESNENRDSVEKQAIRDPNVKEFACGKWVYDDENLQAYRPSILKRIDEDKQTATDITPCDYSNAENKSGKITTPYTKISVHKTE                  301       311       321       331       341       351       361       371       381       391                         |         |         |         |         |         |         |         |         |         |         HB8:HPB8_2        PLEEPQTFEAKNNFAILQARSSTEKCKRARVRKDGTTRQCYLIEEPLKQAWESEYEITTQLVKAIYERPKQDDQIEPTFYETSELAYSSTRKSEITQNELHSJM:HPSJM_07795  PLEEPQTFEAKNNFAILQARSSTEKCKRARARKDGTTRQCYLIEEPLKQAWESEYEITTQLVKAVYERPKQDDQVEPTFYETSELAYSSTRKSEITRNELHB38:HELPY_1499   PLEEPQTFEAKNNFTILQARSSTEKCKRARARKDGTTRQCYLIEEPLKQAWESEYEITTQLVKAVYERPKQDDQAEPTFYETNELAYSSTRKSEITQNELHG27:HPG27_1449   PLEEPQTFEAKNNFTILQARSSTEKCKRARARKDGTTRQCYLIEEPLKQAWESEYEITTQLVKAIYERPKQDDQVEPTFYETSELAYSSTRKSEITRNELH266:HP1527       PLEEPQTFEAKNNFAILQARSSTEKCKRARARKDGTTRQCYLIEEPLKQAWESEYEITTQLVKAIYERPKQDDQVEPTFYETSELAYSSTRKSEITHNELHHPA:HPAG1_1388   PLEEPQTFEAKNNFAILQARSSTEKCKRARARKDGTTRQCYLIEEPLKQAWESEYEITTQLVKAIYERPKQDDQVEPTFYETSELAYSSTRKSEITQNELHP12:HPP12_1502   PLEEPQTFEAKNNFTILQARSSTEKCKRARARKDGTTRQCYLIEEPLKQAWESEYEITTQLVKAVYERPKQDDQVEPTFYETSELAYSSTRKSEITHNELHF32:HPF32_1413   PLEEPQTFEAKNNFTILQARSSTEKCKRARARKDGTIRQCYLIEEPLKQAWESEYQITTQLVKATYERPKQDDQTEPTFYETSELAYSSTRKSEITRNELH52:HPKB_1429     PLEEPQTFEAKNNFTILQARSSTEKCKRARARKDGTIRQCYLIEEPLKQAWESEYEITTQLVKATYERPKQDDQTEPTFYETSELAYSSTRKSEITHNELHF57:HPF57_1440   PLEEPQTFEAKNNFTILQARSSTEKCKRARARKDGTTRQCYLIEEPLKQAWESEYEITTQLVKATYERPKQDDQTEPTFYETSELAYSSTRKSEITHNELH51:KHP_1379      PLEEPQTFEAKNNFTILQARSSTEKCKRARARKDGTTRQCYLIEEPLKQAWESEYEITTQLVKATYERPKQDDQTEPTFYETSELAYSSTRKSEITHNELHF30:HPF30_1399   PLEEPQTFEAKNNFTILQARSSTEKCKRARARKDGTTRQCYLIEEPLKQAWESKYEITTQLVKATYERPKQDDQIEPTFYETSELAYSSTRKSEIMHNELHF16:HPF16_1422   PLEEPQTFEAKNNFTILQARSSTEKCKRARARKDGTTRQCYLIEEPLKQAWESQYQITTQLVKAIYERPKQDDQIEPTFYETSELAYSSTRKSEITHNEL                  401       411       421       431       441       451       461       471                  |         |         |         |         |         |         |         |HB8:HPB8_2        NLNEKFMEFVEVYEGHYLNDIVKESSEYKEWVKNHVRFKEGVCMVLEIEEQPRAKSTPLSIENSKVVCVKKGNYLFNEVHSJM:HPSJM_07795  NLNEKFMEFVEVYEGHYLNDIIKESSEYKEWVKNHVRFKEGVCMALEIEEQPRAKSTPLSIENSRVVCVKKGNYLFNEVHB38:HELPY_1499   NLNEKFMEFVEVYEGHYLNDIIKESSEYKEWVKNHVHFKEGVCMVLEIEEQPRAKSTPLSIENSRVVCVKKGNYLFNEVHG27:HPG27_1449   NLNEKFMEFVEVYEGHYLNDIIKESSEYKEWVKNHVRFKEGVCMVLEIEEQPRAKSTPLSIENSRVVCVKKGNYLFNEVH266:HP1527       NLNEKFMEFVEVYEGHYLNDIIKESSEYKEWVKNHVRFKEGVCMALEIEEQPRAKSTPLSIENSRVVCVKKGNYLFNEVHHPA:HPAG1_1388   NLNEKFMEFVEVYEGHYLNDIVKESSEYKEWVKNHVRFKEGVCMALEIEEQPRAKSTPLSIENSRVVCVKKGNYLFNEVHP12:HPP12_1502   NLNEKFMEFVEVYEGHYLNDIIKESSEYKEWVKNHVRFKEGVCMVLEIEEQPRAKSTPLSIENSRVVCVKKGNYLFNEVHF32:HPF32_1413   NLNEKFMEFVEVYEGHYLNDIVKESSEYKEWVKNHVRFKEGVCMVLEIEEQPRAKSTPLSIENSRVVCVKKGNYLFNEVH52:HPKB_1429     NLNEKFMEFVEVYEGHYLNDIVKESSEYKEWVKNHVRFKEGVCMVLEIEEQPRAKSTPLSIENSRVVCVKKGNYLFNEVHF57:HPF57_1440   NLNEKFMEFVEVYEGHYLNDIVKESSEYKEWVKNHVRFKEGVCMVLEIEEQPRAKSTPLSIENSRVVCVKKGNYLFNEVH51:KHP_1379      NLNEKFMEFVEVYERHYLNDIVKESSEYKEWVKNHVRFKEGVCMVLEIEEQPRAKSTPLSIENSRVVCVKKGNYLFNEVHF30:HPF30_1399   NLNEKFMEFVEVYEGHYLNDIVKESSEYKEWVKNHVRFKEGVCMVLEIEEQPRAKSTPLSIENSRVVCVKKGNYLFNEVHF16:HPF16_1422   DLNEKFMEFVEVYEGHYLNDIIKESSEYKEWVKNHVRFKEGVCIVLEIEEQPRAKSTPLSIENSKVVCVKKGNYLFNEV
