# Supplementary material for: Evolution in an oncogenic bacterial species with extreme genome plasticity: Helicobacter pylori East Asian genomes
Source: BMC Microbiol. 2011 May 16;11:104. doi: 10.1186/1471-2180-11-104 (PMC3120642; doi:10.1186/1471-2180-11-104)
Supplement: Additional file 6 — Multiple sequence alignments of diverged genes. [file 1471-2180-11-104-S6.ZIP › Diverged_genes_multiple_seuence_alignments/HP1551_yajC.mfa.rtf]

                  1         11        21        31        41        51        61        71        81        91                          |         |         |         |         |         |         |         |         |         |         HB8:HPB8_1679     MGQIKDILTTLLPLVVLFLIFYFLIVRPQRQQQKKHKEMIEGLTKGDKIVTQGGLIVEVLKAEANFFSVKLNDDTTAKLSKNYVAFKLDELDQFGLAEPIH266:HP1551       MGQIKDILTTLLPLVVLFLIFYFLIVRPQRQQQKKHKEMIESLTKGDKIVTQGGLIVEVLKAEANFFSVKLNDDTTAKLSKNYVAFKLDELDQFGLAEPIHSJM:HPSJM_08020  MGQIRDILTTLLPFVVLFLIFYFLIVRPQRQQQKKHKEMIEGLTKGDKIVTQGGFIVEVLKAEANFFSVKLNDDTTAKLSKNYVAFKLDELDQFGLAEPIHB38:HELPY_1554   MGQIKDILTTLLPLVVLFLIFYFLIVRPQRQQQKKHKEMIEGLTKGDKIVTQGGLIVEVLKAEANFFSVKLNDDTTAKLSKNYVAFKLDELDQFGLAEPIHP12:HPP12_1533   MGQIRDILTTLLPFVVLFLIFYFLIVRPQRQQQKKHKEMIEGLTKGDKIVTQGGLIVEVLKAEANFFSVKLNDDTTAKLSKNYVAFKLDELDQFGLAEPIHF32:HPF32_1447   MGQTKDILTTLLPLVVLFLIFYFLIVRPQRQQQKKHKEMIEGLSKGDKVVTQGGLIVEVLKAEANFFSVKLNDDTTAKLSKNYIAFKLDE----------HF30:HPF30_1432   MGQTKDILTTLLPLVVLFLIFYFLIVRPQRQQQKKHKEMIEGLSKGDKVVTQGGLIVEVLKAEANFFSVKLNDDTTAKLSKNYIAFKLDE----------H51:KHP_1411      MGQTKDILTTLLPLVVLFLIFYFLIVRPQRQQQKKHKEMIEGLTKGDKVVTQGGLIVEVLKAEANFFSVKLNDYTTAKLSKNYIAFKLDE----------HF57:HPF57_1474   MGQTKDILTTLLPLVVLFLIFYFLIVRPQRQQQKKHKEMIEGLTRGDKVVTQGGLIVEVLKAEANFFSVKLNDDTTAKLFKNYIAFKLDE----------HF16:HPF16_1454   MGQTRDILTTLLPLVVLFLIFYFLIVRPQRQQQKKHKEMIEGLTRGDKVVTQGGLIVEVLKAEANFFSVKLNDDTTAKLFKNYIAFKLDE----------H52:HPKB_1463     MGQTKEILTTLLPFVVLFLIFYFLIVRPQRQQQKKHKEMIESLTKGDKVVTQGGLIVEVLKAEANFFSVKLNDDTTAKLSKNYIAFKLDE----------HHPA:HPAG1_1500   MGQIRDILTTLLPLVVLFLIFYFLIVRPQRQQQKKHKEMIEGLTKGDKIVTQGGLIVEVLKAEANFFSVKLNDDTTAKLSKNYIAFKLDE----------                  101       111       121                  |         |         |HB8:HPB8_1679     VIQQGREEISAKLSGAKTLKQRQITTKH266:HP1551       VIQQGREEISAKLSGAKTLKQRQITTEHSJM:HPSJM_08020  VIQQGREEISAKLSGAKTLKQRQITTEHB38:HELPY_1554   VIQQGREEISAKLSGTKTLKQRQITTEHP12:HPP12_1533   VIQQGREEISAKLSGTKTLKQRQITTEHF32:HPF32_1447   --------------------EAAQNNNHF30:HPF30_1432   --------------------EAAQNNNH51:KHP_1411      --------------------EAAQNNNHF57:HPF57_1474   --------------------EAVQNNNHF16:HPF16_1454   --------------------EAVQNNNH52:HPKB_1463     --------------------EVVQNNNHHPA:HPAG1_1500   --------------------ETTPNNN
