# Supplementary material for: Evolution in an oncogenic bacterial species with extreme genome plasticity: Helicobacter pylori East Asian genomes
Source: BMC Microbiol. 2011 May 16;11:104. doi: 10.1186/1471-2180-11-104 (PMC3120642; doi:10.1186/1471-2180-11-104)
Supplement: Additional file 6 — Multiple sequence alignments of diverged genes. [file 1471-2180-11-104-S6.ZIP › Diverged_genes_multiple_seuence_alignments/HP1553_pcrA.mfa.rtf]

                  1         11        21        31        41        51        61        71        81        91                          |         |         |         |         |         |         |         |         |         |         HB8:HPB8_1681     MDTKRQCMALKASAGSGKTFALSVRFLALLFKGANPSEILTLTFTKKATAEMKERILDYLKILQKENLEN--EKEKSQNILKELEEKYHLDPSLVRNSAPH266:HP1553       MDTKRQCMALKASAGSGKTFALSVRFLALLFKGANPSEILTLTFTKKATAEMKERILDYLKILQQENLEN--EKEKSQNILKELEEKYHLDPSLVQNSAPHP12:HPP12_1531   MDTKRQCMALKASAGSGKTFALSVRFLALLFKGANPSEILTLTFTKKATAEMKERILDYLKILQKENLEN--EKEKSQNILKELEEKYHLDPSLVRNSAPHSJM:HPSJM_08030  MDTKRQCMALKASAGSGKTFALSVRFLALLFKGANPSEILTLTFTKKATAEMKERILDYLKILQKENLES--GKEKSQNILKELEEKYHLDPSLVRNSAQHG27:mHPG27_1491  MDTKRQCMALKASAGSGKTFALSVRFLALLFKGANPSEILTLTFTKKATAEMKERILDYLKILQQENLEN--EKEKSQNILKELEEKYHLDPDLVQNSAQHB38:HELPY_1556   MDTKRQCMALKASAGSGKTFALSVRFLALLFKGANPSEILTLTFTKKATAEMKERILDYLKILQKENLES--GKEKSQNILKELEEKYHLDPSLVRNSAQHHPA:HPAG1_1502   MDTKRQCMALKASAGSGKTFALSVRFLALLFKGANPSEILTLTFTKKATAEMKERILDYLKILQKENLENENKKEKSQNILKELEEKYHLDPSLVRNSAPHF32:HPF32_1445   MDTKRQCMALKASAGSGKTFALSVRFLALLFKGANPSEILTLTFTKKATAEMKERILDYLKILQKENLEDEKEKEKSQNILKELEEKYRLDPSFVQNRAQHF57:HPF57_1472   MDTKRQCMALKASAGSGKTFALSVRFLALLFKGANPSEILTLTFTKKATAEMKERILDYLKILQKENLENEKEKEKSQNILKELEEKYRLDPSFVQNSAQHF16:HPF16_1452   MDTKRQCMALKASAGSGKTFALSVRFLALLFKGANPSEILTLTFTKKATAEMKERILDYLKILQKENLEN--EKEKSQNILKELEEKYRLDPSFVQNNAQH51:KHP_1409      MDTKRQCMALKASAGSGKTFALSVRFLALLFKGANPSEILTLTFTKKATAEMKERILDYLKILQKENLENEKEKEKSQNILKELEEKYRLDPSFVQNSAQH52:HPKB_1461     MDTKRQCMALKASAGSGKTFALSVRFLALLFKGANPSEILTLTFTKKATAEMKERILDYLKILQKENLENEKEKEKSQNILKELEEKYRLDPSFVQNSAQHF30:HPF30_1430   MDTKRQCMALKASAGSGKTFALSVRFLALLFKGANPSEILTLTFTKKATAEMKERILDYLKILQKENLEDEKEKEKSQNILKELEEKYRLNPSFVQNRAQ                  101       111       121       131       141       151       161       171       181       191                         |         |         |         |         |         |         |         |         |         |         HB8:HPB8_1681     KIYQRFLNAEVRISTIDAFFQSILRKFCWFVGLSANFEVNEDTKVHQRQLNEGFLSALNNEQLEELSAFIVQCLSYDSYTSDSILERLRFLKNKLYLFDPH266:HP1553       KIYQRFLNAEIRISTIDAFFQSILRKFCWFVGLSANFEVNEDTKAHQQQLNEGFLSALNGEQLEALSVFIAQCLSYDSYTSDSILERLRFLKNKLYLFDPHP12:HPP12_1531   KIYQRFLNAEIRISTIDAFFQSILRKFCWFVGLSANFEVNEDTKAHQQQLNEGFLSALNGEQLEELSVFIAQCLSYDSYTSDSVLERLRFLKNKLYLFDPHSJM:HPSJM_08030  KIYQRFLNAEIRISTIDAFFQSILRKFCWFVGLSANFEVNEDTKAHQQQLNEGFLSALNNEQLEELSVFITQCLSYDNYTSDSILERLRFLKNKLYLFDPHG27:mHPG27_1491  KIYQRFLNAEIRISTIDAFFQSILRKFCWFVGLSANFEVNEDTKAHQRQLNESFLSALNGEQLEELSVFIAQCLSYDSYTSDSILERLRFLKNKLYLFDPHB38:HELPY_1556   KIYQRFLNAEIRISTIDAFFQSILRKFCWFVGLSANFEVNEDTKAHQQQLNEGFLSALNNEQLEELSVFITQCLSYDNYTSDSILERLRFLKNKLYLFDPHHPA:HPAG1_1502   KIYQRFLNAEIRISTIDAFFQSILRKFCWFVGLSANFEVNEDTKAHQQQLNDGFLSALNNEQLEELSVFIVQCLSYDSYTSDSILERLRFLKNKLYLFDSHF32:HPF32_1445   EIYQRFLNAEIRISTIDAFFQSILRKFCWFVGLRANFEVNEDTKAHQQQLNESFLSALDKEQLEELSVFITQCLSYDSYTSDSVLERLRFLKNKLYLFDPHF57:HPF57_1472   KIYQRFLNAEIRISTIDAFFQSILRKFCWFVGLSANFEVNEDTKAHQQQLNESFLSALNNKQLEELSVFIAQCLSYDSYTSDSILEWLRFLKNKLYLFDPHF16:HPF16_1452   KIYQRFLNAEIRISTIDAFFQSILRKFCWFVGLSANFEVNEDTKAYQQQLNESFLSALNNKQLEELSVFIAQCLSYDSYTSDSILEWLRFLKNKLYLFDPH51:KHP_1409      KIYQRFLNAEIRISTIDAFFQSILRKFCWFVGLSANFEVNEDTKAHQQQLNASFLSALDNKQLEKLSVFITQCLSYDSYTSDSILERLRFLKNKLYLFDPH52:HPKB_1461     KIYQRFLNAEIRISTIDAFFQSILRKFCWFVGLSANFEVNEDTKAHQQQLNESFLSALNNKQLEELSVFIAQCLSYDSYTSDSVLERLRFLKNKLYLFDPHF30:HPF30_1430   KIYQRFLNAEIRISTIDAFFQSILRKFCWFVGLSANFEVNEDTKAHQQQLNESFLSALNGEQLEELSVFIAQCLSYDSYTSDSILERLRFLKNKLYLFDS                  201       211       221       231       241       251       261       271       281       291                         |         |         |         |         |         |         |         |         |         |         HB8:HPB8_1681     NKKEPVFDEESFLEKLRSLNNQIQSIETASDRAKEAIKCDSFRGFLNSSLTWLEKKSEYIYFKKLKDEIPTLESECEEIENDLKRYYEARETALFKKFPKH266:HP1553       NKKEPAFDEKDFLEKLRSLNEQIQSIETASDRAKTAIKCDDFRGFLNSSLTWLEKKSEYQSFKKLKSEIPTLESECEEIENDLKRYYEAKETAIFKKFPKHP12:HPP12_1531   NKKEPAFDEEGFLEKLRNLNQQIQSVETASDTAKKAIKCDDFRGFLNSSLTWLKKKGEYRDFKKLKDEIPTLESECEEIENDLKRYYEAREIALFKKFPKHSJM:HPSJM_08030  NKKEPAFDEEGFLEKLRSLNNQIQSIETASNEAKKAIKCDSFRGFLNSSLTWLEKKSEYLYFKKLKNEIPTLESECEEIENDLKRYYEAKETAIFKKFPKHG27:mHPG27_1491  NKKEPAFDEKGFLEKLRSLNEQIQNIETASDRAKTAIKCDSFRGFLNSSLTWLKKKSEYQFFKKLKSEIPTLESECKEIENDLKRYYEAKETAIFKKFPKHB38:HELPY_1556   NKKEPAFDEKGFLEKLRSLNEQIQSIETASDRAKTAIKCDSFRGFLNSSLTWLEKKSEYQSFKKLKSEIPTLESECEEIENDLKRYYEAKETAIFKKFPKHHPA:HPAG1_1502   NKQEPVFDEEGFLEKLKSLNNQIQNIETASDRAKTAIKCDSFRGFLNSSLTWLEKKSEYQSFKKLKSEIPTLESECEEIENDLKRYYEAKETAIFKKFPKHF32:HPF32_1445   NKKDPIFNEEGFLEKLRSLNQQIQSVETASNEAKKAIKCDDFRGFLNSSLTWLEKKGEYRYFKKFKDEIPTLESECEEIENDLKRYYEAKESALFKKFPKHF57:HPF57_1472   NKKEPAFDEEGFLEKLRSLNRQIQSVETASNEAKKAIKCDDFRGFLNSSLTWLEKKSEYRYFKKFKDEIPTLESECEEIENDLRCYYEARESALFKKFPKHF16:HPF16_1452   NKKEPAFDEEGFLEKLRSLNRQIQSVETASNEAKKAIKCDDFRGFLNSSLTWLEKKSEYRYFKKFKDEIPTLESECEEIENDLRRYYEARESALFKKFPKH51:KHP_1409      NKKEPIFDEEGFLDKLRSLNRQIQSVETASDTAKKAIKCDDFRGFLNSSLTWLKKKGEYRDFKKIKDEIPTLESECEEIENDLKRYYEARESALFKKFPKH52:HPKB_1461     NKKDLAFDEEGFLEKLRNLNKQIQSVETASDTAKKAIKCDDFRGFLNSSLTWLKKKGEYRDFKKIKDEIPTLESECEEIENDLKRYYEARESALFKKFPKHF30:HPF30_1430   NEKEPAFDEEGFLEKLRSLNQQIQSIETASDRAKTAIKCDDFRGFLNSSLTWLEKKSEYIYFKKLKNEIPTLESECEEIENDLKRYYEARETAIFKKFPK                  301       311       321       331       341       351       361       371       381       391                         |         |         |         |         |         |         |         |         |         |         HB8:HPB8_1681     FIQLYDKATSKIQALDFDAIKDKVHVLLNGYEEMPAEFFYFRLDSKIAHILIDEFQDTSLNDYKILAPFIDEIKAGIGQAKWHRSVFFVGDVKQSIYAFRH266:HP1553       FIQLYDNATSKIQALDFDAIKDKVHVLLNGYEEMPAEFFYFRLDSKIAHILIDEFQDTSLNDYKILAPFIDEIKAGIGQAKWHRSVFFVGDVKQSIYAFRHP12:HPP12_1531   FIQLYNKATSKIQALDFDAIKDKVHVLLNGYEEMPAEFFYFRLDSKIAHILIDEFQDTSLNDYKILAPFIDEIKAGIGQAKWHRSVFFVGDVKQSIYAFRHSJM:HPSJM_08030  FIQLYDNATSKIQTLDFDAIKDKVHVLLNGYEEMPAEFFYFRLDSKIAHILIDEFQDTSLNDYKILAPFIDEIKAGIGQAKWHRSVFFVGDVKQSIYAFRHG27:mHPG27_1491  FIQLYDNATSKIQALDFDAIKDKVHVLLNGYEEMPAEFFYFRLDSKIAHILIDEFQDTSLNDYKILAPFIDEIKAGIGQAKWHRSVFFVGDVKQSIYAFRHB38:HELPY_1556   FIQLYDNATSKIQALDFDAIKDKVHVLLNGYEEMPAEFFYFRLDSKIAHILIDEFQDTSLNDYKILAPFIDEIKAGIGQAKWHRSVFFVGDVKQSIYAFRHHPA:HPAG1_1502   FIQLYDKATSKIQALDFDAIKDKVHVLLNGYEEMPAEFFYFRLDSKIAHILIDEFQDTSLNDYKILAPFIDEIKAGIGQAKWQRSVFFVGDVKQSIYAFRHF32:HPF32_1445   FIQLYDKATSKIQALDFDAIKDKVHALLNGYEEMPAEFFYFRLDSRIAHILIDEFQDTSLNDYKILAPFIDEIKAGIGQAKWHRSVFFVGDVKQSIYGFRHF57:HPF57_1472   FIQLYDKATSKIQALDFDAIKDKVHALLNGYEEMPAEFFYFRLDSRIVHILIDEFQDTSLNDYKILAPFIDEIKAGIGQAKWHRSVFFVGDVKQSIYGFRHF16:HPF16_1452   FIQLYDKATSKIQALDFDAIKDKVHALLNGYEEMPAEFFYFRLDSRIAHILIDEFQDTSLNDYKILAPFIDEIKAGIGQAKWHRSVFFVGDVKQSIYGFRH51:KHP_1409      FIQLYDKATSKIQALDFDAIKDKVHALLNGYEEMPAEFFYFRLDSRIAHILIDEFQDTSLNDYKILAPFIDEIKAGIGQAKWHRSVFFVGDVKQSIYGFRH52:HPKB_1461     FIQLYDKATSKIQALDFDAIKDKVHALLNGYEEMPAEFFYFRLDSRIVHILIDEFQDTSLNDYKILAPFIDEIKAGIGQAKWHRSVFFVGDVKQSIYGFRHF30:HPF30_1430   FIQLYDKATSKIQALDFDAIKDKVHALLNGYEEMPAEFFYFRLDSKIAHILIDEFQDTSLNDYKILAPFIDEIKAGIGQAKWQRSVFFVGDVKQSIYGFR                  401       411       421       431       441       451       461       471       481       491                         |         |         |         |         |         |         |         |         |         |         HB8:HPB8_1681     GSFSSLFESVSKDFYHDNLQFNHRSAPLIINYVNTIFKKAYQNSPTAYLEQKYPKTSQNKHVTEGYVKVSLVADERELLLDQVLQEAQNLLDHHIDPKDIH266:HP1553       GSFSSLFESVSKDFYHDNLEFNHRSAPLIINYVNTIFKKAYQNSPTAYLEQKYPKTSQNKHVTDGYVKVSLVADERELLLDQVLQEAQNLLEHRIEPKDIHP12:HPP12_1531   GSFSSLFESVSKDFYHDNLEFNHRSAPLIINYVNTIFKKAYQNSPTAYLEQKYPKTSQNKHATEGYVKVSLVADERELLLEQILQEAQNLLEHHIDPKDIHSJM:HPSJM_08030  GSFSSLFESVSKDFYHDNLEFNHRSAPLIINYVNTIFKKAYQDSPTAYLEQKYPKTSQNKHVTEGYVKVSLVADERELLLDQVLQEAQNLLDHRIDPKDIHG27:mHPG27_1491  GSFSSLFESVSKDFYHDNLQFNHRSAPLIINYVNTIFKKAYQNSPTAYLEQKYPKASSNKHATDGYVKVSLVADDRELLLDQILQEVQNLLEHRIDPKDIHB38:HELPY_1556   GSFSSLFESVSKDFYHDNLQFNHRSAPLIINYVNTIFKKAYQNFPTAYLEQKYPKASSNKHATDGYVKVSLVADERELLLEQILQEAQNLLEHRIDPKDIHHPA:HPAG1_1502   GSFSSLFESVSKDFYHDNLQFNHRSAPLIINYVNTIFKKAYQNSPTAYLEQKYPKASNNKHVTEGYVKVSLVADERELLLEQILQEAQNLLDHHIDPKDIHF32:HPF32_1445   GSFSSLFESVSKDFYHDNLEFNHRSSPLIINYVNTIFKKAYQNSPTAYLEQKYPKASNNKHATDGYVKVSLVADERELLLEQILQEAKNLLEHRIDPKDIHF57:HPF57_1472   GSFSSLFESVSKDFYHDNLPFNHRSSPLIINYVNTIFKKAYQNSPTAYLEQKYPKASSNKHARDGYVKVSLVADERELLLEQILQEAKNLLEHRLDPKDIHF16:HPF16_1452   GSFSSLFESVSKDFYHDNLPFNHRSSPLIINYVNTIFKKAYQNSPTAYWEQKYPKASSNKHARDGYVKVSLVADERELLLEQILQEAKNLLEHRIDPKDIH51:KHP_1409      GSFSSLFESVSKDFYHDNLEFNHRSSPLIINYVNAIFKKAYQNSPTAYLEQKYPKASSNNHARDGYVKVSLVADERELLLEQILQEAKNLLEHRIEPKDIH52:HPKB_1461     GSFSSLFESVSKDFYHDNLPFNHRSSPLIINYVNTIFKKAYQNSPTAYLEQKYPKASSNKHAKDGYVKVSLVADERELLLKQILQEAKNLLEHRIEPKDIHF30:HPF30_1430   GSFSSLFESVSKDFYHDNLQFNHRSSPLIINYVNTIFKKAYQNSPTAYLEQKYPKASSNKHAKDGYVKVSLVADERELLLKQILQEAKNLLEHRIDPKDI                  501       511       521       531       541       551       561       571       581       591                         |         |         |         |         |         |         |         |         |         |         HB8:HPB8_1681     TILCATNKDALEIKNYLQENLSAIRPSTESSAKLSQFVESKIIKNALRYALAEEPYKPFYKHSVLKLAGYLHDDVIALAGFNPKKESVAGFVWKIMELFEH266:HP1553       TILCATNDDALEIKNYLQERLSAIRPSTESSAKLSQFVESKIIKNALEYALAEEPYKPFYKHSVLKLAGYLHDDAIALAGFNPKKESVAGFVWKVMEQFEHP12:HPP12_1531   TILCATNKDALEIKNYLQERLSAIRPSTESSAKLSQFVESKIIKNALEYALAEEPYKPFYKHSVLKLAGYLHDDAIALPGFNPKKESVAGFVWKVMELFEHSJM:HPSJM_08030  TILCATNKDALEIKNYLQERLSAIRPSTESSAKLSQLVESKIIKNALEYALAEEPYKPFYKHSVLKLAGYLHDDAIALPGFNPKKESVASFVWKVMELFEHG27:mHPG27_1491  TILCTTNEDALEIKNYLQENLSTIRPSTESSANLSQFVESKIIKNALEYALAEEPYKPFYKHSVLKLAGYLHDDVIALPGFNPKKESVAGFVWKVMELFEHB38:HELPY_1556   TILCATNKDALEIKNYLQEYLSDIRPSTESSTNLSQFVESKIIKNALRYALAEEPYKPFYKHSVLKLAGYLHDDAIVLPSFNPKKESVAGFVWKVMELFEHHPA:HPAG1_1502   TILCATNDDALEVKNYLQEYLSEICPSTESSAKLSQFVESKIIKNALKYALAEEPYKPFYKHSVLKLAGYLHDDAIALPSFNPKKESVAGFVWKIMEQFKHF32:HPF32_1445   TILCATNDDALEIKNYLQENLSAIRPSTESSTNLSQFVESKIIKNALKYALAEEPYKPFYKHSVLKLAGYLHDDVIALPGFNPKKESVAGFVWKVMEWFEHF57:HPF57_1472   TLLCATNDDALEIKNYLQKNLSAIRPSTESSAKLSQFVESKIIKNALEYALAEEPYKPFYKHSVLKLAGYLHDDAIALAGFNPKKESVAGFVWKVMELFEHF16:HPF16_1452   TLLCATNDDALEIKNYLQKNLSAIRPSTESSAKLSQFVESKIIKNALEYALAEEPYKPFYKHSVLKLAGYLHDDAIALAGFNPKKESVAGFVWKVIELFEH51:KHP_1409      TLLCATNDDALEITNYLQKNLSAIRPSTESSAKLSQFVESKIIKNALEYALAEEPYKPFYKHSVLKLAGYLHDDAIALAGFNPKKESVAGFVWKVMELFEH52:HPKB_1461     TLLCATNDDALEIKNYLQENLSAIRPSTESSAKLSQFVESKIIKNALEYALAEEPYKPFYKHSVLKLAGYLHDDAIALVGFNPKKESVAGFVWKVMEWFEHF30:HPF30_1430   TLLCARNKDALEIKNYLQENLSAIRPSTESSAKLSQFVESKIIKNALEYALAEEPYKPFYKHSVLKLAGYLHDDAIALVGFNPKKESVAGFVWKVMELFE                  601       611       621       631       641       651       661       671       681       691                         |         |         |         |         |         |         |         |         |         |         HB8:HPB8_1681     LYGEPAQICLELAVGCEDADGFLEKLEAKSIASFNAKGAQIMTIHKSKGMQFPYVIVCERLGKPNSSHSNQLLEEYDGAELLRLYYRMKNREVVDKDYARH266:HP1553       LYGEPAQSCLELAVGCEDADGFLEKLETKAIASSHSKGAQIMTIHKSKGMQFPYVIVCERLGKPNSSHSNQLLEEYDGTELLRLYYRMKNREVVDKDYARHP12:HPP12_1531   LYGEPAQICLELAVGCEDANDFLEKLEAKAIASFNAKGAQIMTIHKSKGMQFPYVIVCERLGNPKSNHSNQLLEEYNGAELMHLYYRMKNREVVDKDYARHSJM:HPSJM_08030  LYGEPAQICLELAVGCEDADGFLEKLEAKSIASSHSKGAQIMTIHKSKGMQFPYVIVCERLGKPNSSHSNQLLEEYNGTELARLYYRMKNREVVDKDYARHG27:mHPG27_1491  LYGECAQICLELAVGCEDADGFLEKLETKSIASFNAKGAQIMTIHKSKGMQFPYVIVCERLGKPNSSHSNQLLEEYNGAELLRLYYRMKNREVVDKDYARHB38:HELPY_1556   LYGEPAQSCLELAVGCEDADGFLEKLEAKKIASFNAKGAQIMTIHKSKGMQFPYVIVCERLGNPNSSHSNQLLEEYNGAELVCLYYRMKNREVVDKDYARHHPA:HPAG1_1502   LYGEPAQSCLELAVGCEDANGFLEKLEAKSIASFNLKGAQIMTIHKSKGMQFPYVIVCERLGKPNSSHANQLLEEYNGAELLRLYYRMKNREVVDKDYARHF32:HPF32_1445   LYGECAQICLELAVGCKDANEFLEKLEAKEIASFKIEGAQIMTIHKSKGMQFPYVIVCERLGKPKTNNSNQFLEEYSGTELTRLYYRMKNREVVDKDYARHF57:HPF57_1472   LYGECAQICLELAVGCEDADEFLKKLEAKEIASFKIGGAQIMTIHKSKGMQFPYVIVCERLGKPKTNNSNQFLEEYSNTELTRLYYRMKNREVVDKDYARHF16:HPF16_1452   LYGECAQICLELAVGCEDANEFLEKLEAKEIASFKAEGAQVMTIHKSKGMQFPYVIVCERLGKPKTNNSNQFLEEYSGTELTRLYYRMKNREVVDKDYARH51:KHP_1409      LYTECAQICLELAVGCEDANEFLKKLEAKEIASFKAEGAQIMTIHKSKGMQFPYVIVCERLGKPKTNNSNQFLEEYSGTELTRLYYRMKNREVVDKDYARH52:HPKB_1461     LYGECAQICLELAVGCEDANEFLEKLEAKEIASFKAEGTQIMTIHKSKGMQFPYVIVCERLGKPKSNNSNQFLEEYSGTELTRLYYRMKNREVVDKDYARHF30:HPF30_1430   LYGECAQICLELAVGCEDADEFLKKLEAKEIASFKIEGAQIMTIHKSKGMQFPYVIVCERLGKPKTNNSNQFLEEYSGTELTCLYYRMKNREVVDKDYAR                  701       711       721       731       741       751       761       771       781       791                         |         |         |         |         |         |         |         |         |         |         HB8:HPB8_1681     ALDKEEAAKDHEETNVYYVAFTRAELGLIVVAKD------------------------K--KESKKESKNKTMREQLDLVPLEEGEIMPVISPQKEPLITH266:HP1553       ALDKEEAAKDHEEINVYYVAFTRAELGLIVVAKD------------------------K--KESKKESKNKTMREKLDLVPLEEGEIAPVISPQKEPLITHP12:HPP12_1531   ALDKEEAAKDHEETNVYYVAFTRAELGLIVVAKD------------------------K--KESKKENKNKTMHEKLDLALLEEGEIMPVISPQKEPLITHSJM:HPSJM_08030  ALDKEEAAKDHEETNVYYVAFTRAELGLIVVAKD----------------KKESKKESK--KESKKESKNKTMHEKLDLAPLEEGEIMPVISPQKEPLIAHG27:mHPG27_1491  ALDKEEAAKDHEEINVYYVAFTRAELGLIVVAKDKKESKKESKKESKKESKKESKKESK--KESKKESKNKTMREQLDLVPLEEGEIAPVISPQKEPLITHB38:HELPY_1556   ALEKEEEAKDHEETNVYYVAFTRAELGLIVVAKD------------------------K--KESKKESKNKTMREQLDLTPLEEGEIAPVVSHRKEPLITHHPA:HPAG1_1502   ALNKEEAAKDHEETNVYYVAFTRAELGLIVVAKD------------------KDQKKDK--KESKKESKNKGMREKLDLFPLEEGTIAPVISPQKEPLIAHF32:HPF32_1445   ALDKEEAAKNHEETNVYYVAFTRAELGLIVVAKD------------------------K-----------KGMREKLDLAPLEEGEIAPVISSQKEPLIPHF57:HPF57_1472   ALDKEEVAKDHEETNVYYVAFTRAELGLIVVAKD------------------------K-----------KGMHEKLDLAPLEEGEIATVISSQKEPSSKHF16:HPF16_1452   ALDKEEAAKDHEETNVYYVAFTRAELGLIVVAKD------------------------KDQKKDKKESKSKGMREKLDLAPLEEGEIAPVISSQKEPSPEH51:KHP_1409      ALDKEEAAKDHEETNVYYVAFTRAELGLIVVAKD------------------------KDQKKDKKESKNKGMREKLDLAPLEEGEIAPVISSQKEPSPEH52:HPKB_1461     ALDKEEAAKDHEETNVYYVAFTRAELGLIVVAKD------------------------KNQKKDKKESKNKGMREKLDLAPLEEGEIAPVISSQEEPLSAHF30:HPF30_1430   ALDKEEAAKDHEETNVYYVAFTRAELGLIVVAKD------------------------KDQKKDKKESKNKGMREKLDLAPLEEGEIAPVIYSQKEPSIP                  801       811       821       831       841       851       861       871       881       891                         |         |         |         |         |         |         |         |         |         |         HB8:HPB8_1681     SALIKPHAYGEQVQEIEEEP-SDYEKNNDQEAINFGIALHKGLEYQYAYNIPKKSVLEYLNYHHGFYGLDYQALEESLELFENDAEIQALFKNHALKGEAH266:HP1553       STLIKPHAYGEQVQEIEEEP-SDYEKNNDQEAINFGIALHKGLEYQYAYNIPKQSVLEYLNYHHGFYGLDYQALEESLELFENDAEIQALFKNLPLKGEAHP12:HPP12_1531   STLIKPHAYGEQVQEIEEEPESDYEKNNDQEAINFGIALHKGLEYQYAYNIPKQSVLEYLNYHHGFYGLDHQALEESLELFENDMEIQTLFKNHALKGEAHSJM:HPSJM_08030  SVVIKPHAYGEQVQEIEEEP-SDYEKNNDQEAINFGIALHKGLEYQYAYNIPKKSVLEYLNYHHGFYGLDYQALEESLELFENDAKIQALFKNLALRGEVHG27:mHPG27_1491  SALIKPHAYGEQVQEIEEEPDSDYEKNNDQEAINFGIALHKGLEYQYAYNVPKQSVLEYLNYHHGFYGLDYQALEESLELFENDAEIQTLFKNYFLKGEAHB38:HELPY_1556   SVVIKPHAYGEQVQEIEEEP-SDYEKNNDQEAINFGIALHKGLEYQYAYNIPKKSVLEYLNYHHGFYGLDHQALEESLELFENDTEIQALFKNYALKGEVHHPA:HPAG1_1502   SVLIKPHAYGEQVQEIEEEP-SDYEKNNDQEAINFGIALHKGLEYQYAYNIPKKSVLEYLNYHHGFYGLDYQALEESLELFENDAEIQALFKNLPLKGEAHF32:HPF32_1445   SVVIKPHAYGEQVQEIEEEP-SDYEKNNDQEAINFGIALHKGLEYQYAYNIPKKSVLEYLNYHHGFYGLDYQALEESLELFENDAKIQALFKNLALRGEVHF57:HPF57_1472   SVLIKPHAYGEQVQEIEEEP-SDYEKNNDQEAINFGIALHKGLEYQYAYNIPKKSVLEYLNYHHGFYGLDYQALEESLELFENDAKIQALFKNLALRGEVHF16:HPF16_1452   SVVIKPHAYGEQVQEMEEEP-SDYEKNNDQEAINFGIALHKGLEYQYAYNIPKKSVLEYLNYHHGFYGLDYQALEESLELFENDAKIQALFKNLALKGEVH51:KHP_1409      SVLIKPHAYGEQVQEIEEEP-SDYEKNNDQEAINFGIALHKGLEYQYAYRIPKKSVLEYLNYHHGFYGLDYQALEESLELFENDAKIQALFKNLALRGEVH52:HPKB_1461     SVVIKPHAYGEQVQEIEEEP-SDYEKNNDQEAINFGIALHKGLEYQYAYRIPKKSVLEYLNYHHGFYGLDYQALEESLELFENDAKIQALFKNLALRGEVHF30:HPF30_1430   SVLIKPHAYGEQVQEIEEEP-SDYEKNNDQEAINFGIALHKGLEYQYAYNIPKESVLEYLNYHHGFYGLDYQALEESLELFENDAKIQALFKNLALRGEV                  901       911       921       931       941       951       961       971                  |         |         |         |         |         |         |         |HB8:HPB8_1681     AFLFEGVVSRIDVLLWDKGQNLCVLDYKSSQNYQQSHKAQVSHYAEFLKTQAPHFKIQAGIIYAHKRLLEKLWVH266:HP1553       AFLFQGVVSRIDVLLWDRGQNLYVLDYKSSQNYQQSHKAQVSHYAEFLKTQAPHFKIQAGIIYAHKRLLEKLWVHP12:HPP12_1531   AFLFQGVVSRIDVLLWDKGQNLCVLDYKSSQNYQQSHKAQVSHYAEFLKTQAPHFKIQAGIIYAHKRLLEKLWVHSJM:HPSJM_08030  AFLFEGVVSRIDVLLWDKGQNLYVLDYKSSQNYQQSHKAQVSHYAVFLKTQAPHFKIQAGIIYAHKRLLEKLWVHG27:mHPG27_1491  AFLFEGVVSRIDVLLWDRGQNLYVLDYKSSQNYQQSHKAQVSHYAAFLKTQAPHFKIQAGIIYAHKRLLEKLWVHB38:HELPY_1556   AFLFEGVVSRIDVLLWDKGQNLCVLDYKSSQNYQQSHKAQVSHYAAFLKTQAPHFKIQAGIIYAHKRLLEKLWVHHPA:HPAG1_1502   AFLFEGVVSRIDVLLWDRGQNLYVLDYKSSQNYQQSHKAQVSHYAEFLKTQAPHFKIQAGIIYAHKRLLEKLWVHF32:HPF32_1445   AFLFEGVVSRIDVLLWDKGQNLYVLDYKSSQNYQQSHKAQVSHYAAFLQTQAPHFKIQAGIIYAHKRLLEKLWVHF57:HPF57_1472   AFLFEGVVSRIDVLLWDKRQNLYVLDYKSSQNYQQSHKAQVSHYAAFLQTQAPHFKIQAGIIYAHKRLLEKLWVHF16:HPF16_1452   AFLFEGVVSRIDVLLWDKGQNLYVLDYKSSQNYQQSHKAQVSHYAAFLQTQAPHFKIQAGIIYAHKRLLEKLWVH51:KHP_1409      AFLFEGVVSRIDVLLWDKGQNLYVLDYKSSQNYQQSHKVQVSHYAAFLQTQAPHFKIQAGIIYAHKRLLEKLWVH52:HPKB_1461     AFLFEGVVSRIDVLLWDKGQNLYVLDYKSSQNYQQSHKVQVSHYTAFLQTQAPHFKIQAGIIYAHKRLLEKLWVHF30:HPF30_1430   AFLFEGVVSRIDVLLWDKGQNLYVLDYKSSQNYQQSHKAQVSHYAAFLQTQAPYFKIQAGIIYAHKRLLEKLWV
