# Supplementary material for: Evolution in an oncogenic bacterial species with extreme genome plasticity: Helicobacter pylori East Asian genomes
Source: BMC Microbiol. 2011 May 16;11:104. doi: 10.1186/1471-2180-11-104 (PMC3120642; doi:10.1186/1471-2180-11-104)
Supplement: Additional file 6 — Multiple sequence alignments of diverged genes. [file 1471-2180-11-104-S6.ZIP › Diverged_genes_multiple_seuence_alignments/mHP0146_fixQ.mfa.rtf]

                  1         11        21        31        41        51        61        71                  |         |         |         |         |         |         |         |HB8:HPB8_1419     MDLESLRGFAYAFFTILFTLFLYAYIFSMYRKQKKGVVDYERYGYLALNDALEDELIEPRHKKVHDNGIKENH266:mHP0146      MDLESLRGFAYAFFTILFTLFLYAYIFSMYRKQKKGIMDYERYGYLALNDALEDELIEPRHKKVHDNGIKESHHPA:mHPAG1_0144  MDLESLRGFAYAFFTILFTLFLYAYIFSMYRKQKKGIVDYERYGYLALNDALEDELIEPRHKKVHDNGIKESHB38:mHELPY_0150  MDLESLRGFAYAFFTILFTLFLYAYIFSMYRKQKKGVVDYERYGYLALNDALEDELIEPRHKEVHDNGIKESHP12:HPP12_0145   MDLESLRGFAYAFFTILFTLFLYAYIFSMYRKQKKGVVDYERYGYLALNDALEDELIEPRHKKVHDKGIKESHSJM:HPSJM_00795  MDLESLRGFAYAFFTILFTLFLYAYIFSMYRKQKKGIMDYERYGYLALNDALEDELIEPRHKEVHDKGIKESHG27:HPG27_133    MDLESLRGFAYAFFTVLFTLFLYAYIFSMYRKQKKGIVDYERYGYLALNDALEDELIEPRHKEVHDKGIKESHF32:HPF32_0155   MDLESLRGFAYAFFTILFTLFLYAYIFSMYRKQKKGIVDYERYGYLALNDALEDELIEPRHKNAHDNGIKESHF16:HPF16_0155   MDLESLRGFAYAFFTILFTLFLYAYIFSMYRKQKKGIVDYERYGYLALNDALEDELIEPRHKNAHDNGIKESH51:mKHP_0145     MDLESLRGFAYAFFTILFTLFLYAYIFSMYRKQKKGIVDYERYGYLALNDALEDELIEPRHKNAHDNGIKESH52:HPKB_0154     MDLESLRGFAYAFFTILFTLFLYAYIFSMYRKQKKGIVDYERYGYLALNDALEDELIEPRHKNAHDNGIKESHF30:HPF30_1149   MDLESLRGFAYAFFTILFTLFLYAYIFSMYRKQKKGIVDYERYGYLALNDALEDELIEPRHKNAHDNGIKESHF57:HPF57_0166   MDLESLRGFAYAFFTILFTLFLYAYIFSMYRKQKKGIVDYERYGYLALNDALEDELIEPRYKNAHDNGIKES
