# Supplementary material for: Evolution in an oncogenic bacterial species with extreme genome plasticity: Helicobacter pylori East Asian genomes
Source: BMC Microbiol. 2011 May 16;11:104. doi: 10.1186/1471-2180-11-104 (PMC3120642; doi:10.1186/1471-2180-11-104)
Supplement: Additional file 6 — Multiple sequence alignments of diverged genes. [file 1471-2180-11-104-S6.ZIP › Diverged_genes_multiple_seuence_alignments/mHP0174.mfa.rtf]

                   1         11        21        31        41        51        61        71        81        91                           |         |         |         |         |         |         |         |         |         |         HB8:HPB8_1392      MVLFLSIFKKSFNDFLSARMLLINLGPILLSLAFFGAVFYYNGGSIVGYCQTLLPQSLNDYSHSQGFFAGVFAWVFKALVYFLIFWIVILLSLVINIFASH266:mHP0174       MVLSLSILKKSFNDFLSTRMLLINLGPILLSLAFFGAVFYYNGGNIVGYCQTLLPQSLNDYSHSQGFFAGVFAWVFKALVYFLIFWIVILLSLVINIFASHG27:HPG27_159     MVLSLSILKKSFNDFLSARMLLINLGPILLSLAFFGAVFYYNGGSIVGYCQTLLPQSLNDYSHSQGFFVGVFAWVFKALVYFLIFWIVILLSLVINIFASHHPA:HPAG1_0170    MVLSLSILKKSFNDFLSVRMLLINLGPILLSLAFFGAIFYYNGGSIVGYCQTLLPQSLSDYSHSQGFFAGVFTWVFKALVYFLIFWIVILLSLVINIFASHB38:HELPY_0177    MVLSLSILKKSFNDFLGARMLLINLGPILLSLAFFGAIFYYNGENIVNYCQALLPQSLSDYSHSQGFFAGVFAWVFKALVYFLIFWIAILLSLVINIFASHF32:HPF32_0182    MVLSLSILKKSFNDFLSARMLLINLGPILLSLAFFGAVFYYNGANIVGYCQTLLPQSLNGYSHSQGFFAGVFAWVFKALVYFLVFWIVILLSLVINIFASHF57:HPF57_0193    MVLSLSILKKSFNDFLSARMLLINLGPILLSLAFFGAVFYYNGANIVGYCQTLLPQSLNGYSHSQGFFAGVFAWVFKALVYFLVFWIVILLSLVINIFVSH52:HPKB_0181      MVLSLSILKKSFNDFLSARMLLINLGPVLLSLAFFGAVFYYNGANIVGYCQTLLPQSLNGYSHSQGFFASMFAWVFKALVYFLVFWIVILLSLVINIFASHF30:HPF30_1122    MVLFLSILKKSFNDFLSARMLLINLGPILLSLAFFGAVFYHNGANIVGYCQTLLPQSLNGYSHSQGFFAGVFAWVFKALVYFLVFWIVILLSLIINIFASHF16:HPF16_0182    MVLFLSILKKSFNDFLSARMLLINLGPILLSLAFFGAVFYYNGASIVGYCQTLLPQSLNGYSHSQGFFTGVFAWVFKALVYFLVFWIVILLSLVINIFVSH51:KHP_0171       MVLSLSILKKSFNDFLSARMLLINLGPILLSLVFFGAVFYYNGASIVGYCQTLLPQSLNGYSYSQGFFAGVFAWVFKALVYFLVFWIVILLSLIINIFASHP12:HPP12_0171    MVLFLSIFKKSFNDFLSARMLLINLGPILLSLAFFGAVFHYNGENIVNYCQTLLPQSLSDYSHSQGFFSGVFVWVFKALVYFLIFWIAILLSLVINIFASHSJM:mHPSJM_00935  --LFLSIFKKSFNDFLSARMILINLGPILLSLAFFGAIFYYNGENIVNYCQTLLPQSLSDYSHSQGFFSGVFTWVFKALVYFLIFWIAILLSLVINIFAS                   101       111       121       131       141       151       161       171       181       191                          |         |         |         |         |         |         |         |         |         |         HB8:HPB8_1392      IFYTPLVVSYLHQKYYPHVVLEEFGSILFSIKYFLKSLAFMLLFLAVLTPFYFIPFIGVFGVFFSIIPHFLFFKNTMSWDIASMIFNYQSYQNLLKQHR-H266:mHP0174       IFYTPLVVSYLHQKYYPHVVLEEFGSILFSIKYFLKSLAFMLLFLAVLTPFYFIPFIGVFGVFFSIVPHFLFFKNTMSLDIASMIFNHQSYQNLLKQHR-HG27:HPG27_159     IFYTPLVVSYLHQKYYPHVVLEEFGSILFSIKYFLKSLAFMLLFLAVLTPLYFIPFIGVFGVFFSIIPHFLFFKNTMSLDIASMIFNHQSYQNLLKQHR-HHPA:HPAG1_0170    IFYTPLVVSYLHQKYYPHVVLEEFGSILFSIKYFLKSLIFMLLFLAVLTPLYFIPFIGVFGVFFSIIVHFLFFKNTMSLDIASMIFNYQSYQNLLKQHR-HB38:HELPY_0177    IFYTPLVVSYLHQKYYPHVVLEEFGSILFSIKYFLKSLAFMLLFLAVLTPFYFIPFIGVFGVFFSIIPHFLFFKNTMSLDIASMIFNHQSYQNLLKQHR-HF32:HPF32_0182    VFYTPLVVSYLHQKYYPHVVLEEFGSVLFSIKYFLKSLIFMLLWMALLMLFYFIPFIGVFGVFFSIIPHFLFFKNTMSLDIASMIFNHQSYQNLLKQHR-HF57:HPF57_0193    VFYTPLVVSYLHQKHYPHVVLEEFGSIFFSIKYFLKSLIFMLLWMAVLTPFYFIPFIGVFGVFFSIIPHFLFFKNTMSLDIASMIFNHQSYQNLLKQHR-H52:HPKB_0181      VFYTPLVVSYLHQKHYPHVVLEEFGSILFSIKYFLKSLIFMLLWMAVLTPFYFIPFIGVFGVFFSIVPHFLFFKNTMSLDIASMIFNHQSYQNLLKQHR-HF30:HPF30_1122    VFYTPLVVSYLHQKHYPHVVLEEFGSILFSIKYFLKSLTFMLLWMAVLTPFYFIPFIGVFGVFFSIIPHFLFFKNTMSLDIASMIFNHQSYQNLLKQHR-HF16:HPF16_0182    VFYTPLVVSYLHQKYYPHVVLEEFGSILFSIKYFLKSLIFMLLWMAVLTPFYFIPFIGVFGVFFSIIPHFLFFKNTMSLDIASMIFNHQSYQNLLKQHR-H51:KHP_0171       VFYTPLVVSYLHQKYYPHVVLEEFGSIFFSIKYFLKSLIFMLLWMAVLTPFYFIPFIGVFGVFFSIIPHFLFFKNTMSLDIASMIFNHQNYQNLLKQHRLHP12:HPP12_0171    IFYTPLVVSYLHQKYYPHVVLEEFGSILFSIKYFLKSLIFMLLFLAVLTPLYFIPFIGVFGVFFSIIVHFLFFKNTMSLDIASAIFNYQSYQNLLKQHR-HSJM:mHPSJM_00935  IFYTPLVVSYLHQKYYPHVVLEEFGSIFFSIKYFLKSLLFMLLFLAVLTPFYFIPFIGVFGVFFSIVPHFLFFKNTMSLDIASMIFNHQSYQNLLKQHR-                   201       211       221       231       241                   |         |         |         |         |HB8:HPB8_1392      -LKHYRFSFFCYLFSLIPFFNFFATLLQTLMLTHYFFILKEKECH266:mHP0174       -LKHYRFSFFCYLFSLIPFFNFFATLLQTLMLTHYFFIFKEKECHG27:HPG27_159     -LKHYRFSFFCYLFSLIPFFNFFATLLQTLMLTHYFFILKEKECHHPA:HPAG1_0170    -LKHYRFSFFCYLFSLIPFFNFFATLLQTLMLAHYFFILKEKECHB38:HELPY_0177    -LKHYRFSFFCYLFSLIPFFNFFATLLQTLMLTHYFFILKEKECHF32:HPF32_0182    -LKHYRFSFFCYLFSLIPFFNFFATLLQTLMLTHYFFILKEKECHF57:HPF57_0193    -LKHYRFSFFCYLFSLIPFFNFFATLLQTLMLTHYFFIFKEKECH52:HPKB_0181      -LKHYRFSFFCYLFSLIPFFNFFATLLQTLMLTHYFFILKEKECHF30:HPF30_1122    -LKHYRFSFFCYLFSLIPFFNFFATLLQTLMLTHYFFILKEKECHF16:HPF16_0182    -LKHHRFSFFCYLFSLIPFFNFFATLLQTLMLTHYFFILKEKECH51:KHP_0171       KLKHYRFSFFCYLFSLIPFFNFFATLLQTLMLTHYFFILKEKECHP12:HPP12_0171    -LKHYRFSFFCYLFSLIPFFNFFATLLQTLMLAHYFFILKEKECHSJM:mHPSJM_00935  -LKHYRFSFFCYLFSLIPFFNFFATLLQTLMLAHYFFIFKEKEC
