# Supplementary material for: Evolution in an oncogenic bacterial species with extreme genome plasticity: Helicobacter pylori East Asian genomes
Source: BMC Microbiol. 2011 May 16;11:104. doi: 10.1186/1471-2180-11-104 (PMC3120642; doi:10.1186/1471-2180-11-104)
Supplement: Additional file 6 — Multiple sequence alignments of diverged genes. [file 1471-2180-11-104-S6.ZIP › Diverged_genes_multiple_seuence_alignments/mHP0181_cvpA.mfa.rtf]

                  1         11        21        31        41        51        61        71        81        91                          |         |         |         |         |         |         |         |         |         |         HB8:HPB8_1385     LNYIDLALLVVVVAFGIRGFYHGFVSEIAGTLGIVLGVYLASRYSVAVGNLFSEHLYDLRNETMTNLIGFLLVLASIWVFFLAFGVLLGKVLVFSGLGIIH266:mHP0181      LNYIDLALLVVVVAFGIRGFYHGFVSEVAGTLGIVLGVYLASRYSVAVGNLFSEHLYDLRNETMTNLIGFLLVLASIWVFFLAFGVLLGKVLVFSGLGIIHB38:HELPY_0185   LNYIDLALLVVVVAFGIRGFYHGFVSEVAGTLGIVLGVYLASRYSVAVGNLFSEHLYDLRNETMTNLIGFLLVLASIWVFFLAFGVLLGKVLVFSGLGIIHHPA:HPAG1_0178   LNYIDLALLVVVVAFGIRGFYHGFVSEVAGTLGIVLGVYLASRYSVAVGNLFSEHLYDLRNETMTNLIGFLLVLASIWVFFLAFGVLLGKVLVFSGLGIIHG27:HPG27_167    LNYIDLALLVVVVAFGIRGFYHGFVSEVAGILGIVLGVYLASRYSVAVGHLFSQHLYDLKNETMMNLVGFLLVLASIWVFFLAFGVLLGKVLVFSGLGIIHP12:HPP12_0181   LNYIDLALLVVVVAFGIRGFYHGFVSEVAGTLGIVLGVYLASRYSVAVGHLFSQHLYDLKNETMMNLVGFLLVLASIWVFFLAFGVLLGKVLVFSGLGIIHF32:HPF32_0191   LNYIDLALLVVVVAFGIRGFYHGFVSEVAGTLGIVLGVYLASRYSVAVGHLFSQHLYDLKNETMMNLVGFLLVLASIWVFFLAFGVLLGKVLVFSGLGIIHF16:HPF16_0189   LNYIDLALLVVVVAFGIRGFYHGFVSEVAGTLGIVLGVYLASRYSVAVGHLFSQHLYDLKNETMMNLVGFLLVLASIWVFFLAFGVLLGKVLVFSGLGIIH52:HPKB_0191     LNYIDLALLVVVVAFGIRGFYHGFVSEVAGTLGIVLGVYLASRYSVAVGHLFSQHLYDLKNETMMNLVGFLLVLASIWVFFLAFGVLLGKVLVFSGLGIIHF30:HPF30_1113   LNYIDLALLVVVVAFGIRGFYHGFVSEVAGILGIVLGVYLASRYSVAVGHLFSQHLYDLKNETMMNLVGFLLVLASIWVFFLAFGVLLGKVLVFSGLGIIH51:KHP_0180      LNYIDLALLVVVVAFGIRGFYHGFVSEVVGILGIVLGVYLASRYSVAVGHLFSQHLYDLKNETMMNLVGFLLVLASIWVFFLAFGVLLGKVLVFSGLGIIHF57:HPF57_0200   LNYIDLALLVVVVAFGIRGFYHGFVSEVAGILGIVLGVYLASRYSVAVGHLFSQHLYDLKNETMMNLVGFLLVLASIWVFFLAFGVLLGKVLVFSGLGIIHSJM:HPSJM_00995  LNYIDLALLVVVVAFGIRGFYHGFVSEVAGTLGIVLGVYLASRYSVAVGNLFSEHLYDLRNETMTNLIGFLLVLASIWVFFLAFGVLLGKVLVFSGLGII                  101       111       121       131       141       151       161       171       181       191                         |         |         |         |         |         |         |         |         |         |         HB8:HPB8_1385     DKALGFIFSCLKTFLVLSFILYALSKMEVMKDANAYLQEKSTFFSTMKSVASKIMRLDGVKHVEQNLKDNLEEMSDEVKNKESFNKN-------KQSFN-H266:mHP0181      DKALGFIFSCLKTFLVLSFILYALSKMEVMKDANAYLQEKSAFFSTMKSVASKIMRLDGVKHVEQNLKDNLEEMSDEVKNKESFNKN-------KESFN-HB38:HELPY_0185   DKALGFIFSCLKTFLVLSFILYALSKMEVMKDANAYLQEKSAFFSTMKSVASKIMRLDGVKHVEQNLKDNLEEMSDEVKNKESFNKN-------KESFN-HHPA:HPAG1_0178   DKALGFIFSCLKTFLVLSFILYALSKMEVMKDANAYLQEKSAFFSTMKSIASKIMRLDGVKHVEQNLKGNLEEMSDEVKNKESFNKN-------KESFN-HG27:HPG27_167    DKALGFIFSCLKTFLVLSFILYALSKMEVMKDANAYLQEKSAFFSTMKSVASKIMRLDGVKHVEQNLKDNLEEMSDEVKNKESFNKN-------KESFN-HP12:HPP12_0181   DKALGFIFSCLKTFLVLSFILYALSKMEVMKDANAYLQEKSAFFSTMKSVASKIMRLDGVKHVEQNLKGNLEEMSDEVKNKESFNKN-------KESFNKHF32:HPF32_0191   DRALGFIFSCLKTFLVLSFILYALSKMEVMKDANAYLQGKSAFFPTMKSVASKIMRLDGVKHVEQNLKNNFEEMSDEVKNKESSNKA-------KESFD-HF16:HPF16_0189   DRALGFIFSCLKTFLVLSFILYALSKMEVMKDANAYLQGKSAFFPTMKSVASKIMRLDGVKHVEQNLKNNLEEMSDEVKNKESSNKA-------KESFD-H52:HPKB_0191     DRALGFIFSCLKTFLVLSFILYALSKMEVMKDANAYLQGKSAFFPTMKSVASKIMRLDGVKHVEQNLKDNLEEMSDEVKNKESLNKT-------KESLD-HF30:HPF30_1113   DRALGFIFSCLKTFLVLSFILYALSKMEVMKDANAYLQEKSAFFPTMKSVASKIMRLDGVKHVEQNLKDNLEEMSDEVKNKESFNKN-------KESFD-H51:KHP_0180      DRALGFIFSCLKTFLVLSFILYALSKMEVMKDANAYLQGKSAFFPTMKSVASKIMRLDGVKHVEQNLKDNLEEMSDEVKNKESSNKA-------KESFD-HF57:HPF57_0200   DRALGFIFSCLKTFLVLSFILYALSKMEVMKDANTYLQGKSAFFPTMKSVASKIMRLDGVKHVEQNLKDNLEEMSDEVKNKESFNKNKESSNKAKESFD-HSJM:HPSJM_00995  DKALGFIFSCLKTFLVLSFILYALSKMEVMKDANAYLQEKSAIFPTMKSIASKIMRLDGVKHVEKNLKDNLEEMSDEVKNKESIDNA-------KESFN-                  201       211       221       231       241       251                  |         |         |         |         |         |HB8:HPB8_1385     ------KAMDKGMESLKEKAKDLPKNMLDPKA-----NQTPPNPTPSNKEPLH266:mHP0181      ------KAMDKGVESLKEKAKDLPKNMLDPKA-----NQTPPNPTPSNKEPLHB38:HELPY_0185   ------KAMDKGVESLKEKAKDLPKNMLDPKA-----NQTPPNPTPSNKEPLHHPA:HPAG1_0178   ------KTMDKGVESLKEKAKDLPKNMLDPKA-----NQTPPNPTPSNKEPLHG27:HPG27_167    ------KAMDKGVESLKEKAKDLPKNMLDPKA-----NQTPPNPTPSNKEPLHP12:HPP12_0181   NKQSFDKAMDKGVESLKEKAKDLPKNMLDPKA-----NQTPPNPTPSNKEPLHF32:HPF32_0191   ------KAMDKGVEALKEKAKDLPKNMRDPKA-----NHTPPNHTPSNKEPLHF16:HPF16_0189   ------KAMDKGMEALKEKAKDLPKNMRDPKA-----NHTPPNHTPSNKEPLH52:HPKB_0191     ------KAMDKGVEALKEKTKDLPKNMRDPKA-----NHTPPNHTPSNKEPLHF30:HPF30_1113   ------KAMDKGMEALKEKAKDLPKNMRDPKA-----NHTPPNHTPSNKEPLH51:KHP_0180      ------KAMDKGVEALKEKAKDLPKNMLDPKA-----NQTPPSHTPSNKEPLHF57:HPF57_0200   ------KAMDKGMEALKEKAKDLPKNMRDPKANHTPPNHTPPNHTPSNKEPLHSJM:HPSJM_00995  ------KAMDKGMESLKEKAKDLPKNMLDPKA-----NQTPPNPTPSNKEPL
