# Supplementary material for: Evolution in an oncogenic bacterial species with extreme genome plasticity: Helicobacter pylori East Asian genomes
Source: BMC Microbiol. 2011 May 16;11:104. doi: 10.1186/1471-2180-11-104 (PMC3120642; doi:10.1186/1471-2180-11-104)
Supplement: Additional file 6 — Multiple sequence alignments of diverged genes. [file 1471-2180-11-104-S6.ZIP › Diverged_genes_multiple_seuence_alignments/mHP1255_secG.mfa.rtf]

                  1         11        21        31        41        51        61        71        81        91                          |         |         |         |         |         |         |         |         |         |         HB8:HPB8_225      MTSALLGLQIVLAVLIVVVVLLQKSSSIGLGAYSGSNDSLFGAKGPASFMAKLTMFLGLLFVINTIALGYFYNKEYGKSILDETKTNKDLSPLVPATG--HSJM:HPSJM_06275  MTSALLGLQIVLAVLIVVVVLLQKSSSIGLGAYSGSNDSLFGAKGPASFMAKLTMFLGLLFVINTIALGYFYNKEYGKSILDETKTNKELSPLVPATG--H266:mHP1255      MTSALLGLQIVLAVLIVVVVLLQKSSSIGLGAYSGSNESLFGAKGPASFMAKLTMFLGLLFVINTIALGYFYNKEYGKSVLDETKTNKELSPLVPATG--HB38:mHELPY_1231  MTSALLGLQIVLAVLIVVVVLLQKSSSIGLGAYSGSNESLFGAKGPASFMAKLTMFLGLLFVINTIALGYFYNKEYGKSILDETKTNKDLSPLVPATG--HHPA:mHPAG1_1199  MTSALLGLQIVLAVLIVVVVLLQKSSSIGLGAYSGSNDSLFGAKGPASFMAKLTMFLGLLFVINTIALGYFYNKEYGKSILDETKTN--LSPLVPTTG--HP12:HPP12_1221   MTSALLGLQIVLAVLIVVVVLLQKSSSIGLGAYSGSNESLFGAKGPASFMAKLTMFLGLLFVINTIALGYFYNKEYGKSILDETKTNKDLSPLVPATG--HG27:HPG27_1200   MTSALLGLQIVLAVLIVVVVLLQKSSSIGLGAYSGSNDSLFGAKGPASFMAKLTMFLGLLFVINTIALGYFYNKEYGKSILDETKTNKDLSPLVPATG--HF32:HPF32_1185   MTSALLGLQIVLAVLIVVVVLLQKSSSIGLGAYSGSNESLFGAKGPASFMAKLTMFLGLLFVANTIALGYFYNKEYGKSILDETKTNKELSPLVPATG--HF30:HPF30_0142   MTSALLGLQIVLAVLIVVVVLLQKSSSIGLGAYSGSNESLFGAKGPASFMAKLTMFLGLLFVINTIALGYFYNKEYSKSILDETKTNKELSPLVPATG--H51:mKHP_1151     MTSALLGLQIVLAVLIVVVVLLQKSSSIGLGAYSGSNESLFGAKGPASFMAKLTMFLGLLFVINTIALGYFYNKEYGKSILDETKTNKELSPLVPATGTLHF16:HPF16_1189   MTSALLGLQIVLAVLIVVVVLLQKSSSIGLGAYSGSNESLFGAKGPASFMAKLTMFLGLLFVINTIALGYFYNKEYGKSILDETKTNKELSPLVPATG--HF57:HPF57_1214   MTSALLGLQIVLAVLIVVVVLLQKSSSIGLGAYSGSNESLFGAKGPASFMAKLTMFLGLLFVVNTIALGYFYNKEYGKSILDETKTNKELSPLVPATG--H52:HPKB_1190     MTSALLGLQIVLAVLIVVVVLLQKSSSIGLGAYSGSNESLFGAKGPASFMAKLTMFLGLLFVANTIALGYFYNKEYGKSILDETKTNKELSPLVPATG--                  101       111       121       131       141       151       161       171       181       191                         |         |         |         |         |         |         |         |         |         |         HB8:HPB8_225      --TLNPTLNPTLNPTLNPLEQAPTNPLMPTQTPNELPKEPAKAPSVESPKQNEKNE--KNDAK------E-NGIKGVEKTKENAKTPPTTHQKPKTHAT-HSJM:HPSJM_06275  --TLNPTLNPTLNPTLNPLEQAPTNPLMPQQTPNELPKEPAKAPSVESPKQNEKNE--KNDAK------E-NGIKGVEKTKENAKTPPTTHQKPKTHATQH266:mHP1255      --TLNPALNPTLNPTLNPLEQAPTNPLMPQQTPNELPKEPAKTPSVESPKQNEKNE--KNDAK------E-NGIKGVEKTKENAKTPPTTHQKPKTHATQHB38:mHELPY_1231  --TLNPTLNPTLNPTLNPLEQAPTNPLMPQQTPNELPKEPAKASSVESPKQNEKNE--KNDAK------E-NSIKGVEKTKENAKTPPTTHQKPKTHATQHHPA:mHPAG1_1199  --TLNPTLNPTLNPTLNPLEQAPTNPLMPKQTPNELPKEPAKAPSVESPKQNEKNDAK----K------E-NNIKGVEKTKESAKTPPTTHQKPKTHAT-HP12:HPP12_1221   --TLNPTLNPTLNPTLNPLEQAPTNPLMPKQTPDGLPKEPAKGLSVESPKQNEKNDAKENNIK------K-NSIKGVEKTKENAKTPPTTHQKPKTHATQHG27:HPG27_1200   ------TLNPTLNPTLNPLEQAPTNPLMPKQTPDELPKEPAKAPSVESPKQNEKNDAKENNIK------K-NSIKGVEKTKESAKTPPTTHQKPKTHATQHF32:HPF32_1185   --TLNPMLNPTLNPTLNPLEQVPTNPLMPKQTPNEFPKEPAKAPFIESPKQNEKNDAKKEN-K------E-NNIKGVEKTKEDTKTPPTAHQKPKTH-TQHF30:HPF30_0142   --TLNPTLNPTLNPTLNPLEQAPTNPLMPKQTPNEFPKEPAKAPFIESPKQNEKNDAKKEN-K------E-NNIKGVEKTKEDTKTPPTAHQKPKTH-TQH51:mKHP_1151     NPTLNPTLNPTLNPTLNPLEQAPTNPLMPKQTPNEFPKEPAKAPFIESPKQNEKNDAKKEN-K------E-NNIKGVEKTKEDTKTPPTAHQKPKTH-TQHF16:HPF16_1189   --TLNPTLNPTLNPTLNPLEQVPTNPLMPKQTPNELPKEPAKAPFIESPKQNEKNDAKKEN-KENKENRE-NNIKGVEKTKEDTKTPPTAHQKPKTH-TQHF57:HPF57_1214   --TLNPTLNPTLNPTLNPLEQVPTNPLIPKQTPNELPKEPAKAPFIESPKQNEKNGVKKEN-K------ENNNIKGVEKTKEDTKTPPTAHQKPKTH-TQH52:HPKB_1190     ----------TLNPTLNPLEQAPTNPLMPKQTPNELPKEPAKAPFIENPKQNEKNDTKKEN-K------E-NNIKGVEKTKEDAKTPPTTHQKPKTHATQ                  201       211                  |         |HB8:HPB8_225      TNAHTNQKKDEKHSJM:HPSJM_06275  TNAHTNQKKDEKH266:mHP1255      TNAHTNQKKDEKHB38:mHELPY_1231  TNAHTNQKKDEKHHPA:mHPAG1_1199  TNAHTNQKKDEKHP12:HPP12_1221   TNAHTNQKKDEKHG27:HPG27_1200   TNAHTNQKKDEKHF32:HPF32_1185   TNAHTNQKKDEKHF30:HPF30_0142   TNAHINQKKDEKH51:mKHP_1151     TNAHTNQKKDEKHF16:HPF16_1189   TNAHINQKKDEKHF57:HPF57_1214   TNVHTNQKKDEKH52:HPKB_1190     TNAHTNQKKDEK
