# Supplementary material for: Evolution in an oncogenic bacterial species with extreme genome plasticity: Helicobacter pylori East Asian genomes
Source: BMC Microbiol. 2011 May 16;11:104. doi: 10.1186/1471-2180-11-104 (PMC3120642; doi:10.1186/1471-2180-11-104)
Supplement: Additional file 6 — Multiple sequence alignments of diverged genes. [file 1471-2180-11-104-S6.ZIP › Diverged_genes_multiple_seuence_alignments/mHP1323_rnhB.mfa.rtf]

                  1         11        21        31        41        51        61        71        81        91                          |         |         |         |         |         |         |         |         |         |         HB8:HPB8_156      MVLGIDEAGRGCLAGSLFVAGVACDEKTALEFLKMGLKDSKKLSPKKRFFLEDKIKTHGEVGFFVVKKSANEIDSLGLGACLRLSIEEILENGCSLANEIHG27:HPG27_1272   MILGIDEAGRGCLAGSLFVAGVACDDQTALEFLKMGLKDSKKLSPKKRFFLEDKIKTHGEVGFFVVKKSANEIDSLGLGACLKLAVQEILENGCSLANEIHSJM:HPSJM_06635  MILGIDEAGRGCLAGSLFVAGVVCSEKTALEFLEMGLKDSKKLSPKKRFFLEDKIKTHGEVGFFVVKKSAEAIDSLGLGACLKLAIQEILENGCSLANEIHHPA:HPAG1_1268   MTLGIDEAGRGCLAGSLFVAGVACSEKTALEFLKMGLKDSKKLSQKKRFFLEDKIKTHGEVGFFVVKKSAEAIDSLGLGACLKLAIEEILENGCSLANEIHB38:HELPY_1299   MVLGIDEAGRGCLAGSLFVAGVVCSEKTALEFLKMGLKDSKKLSPKKRFFLEDKIKTHGEVGFFVVKKSANEIDSLGLGACLKLAIQEILENGCSLANEIH266:mHP1323      MTLGIDEAGRGCLAGSLFVAGVACNEKTALEFLKMGLKDSKKLSLKKRFFLEYKIKTHGEVGFFVVKKSANEIDSLGLGACLKLAVQEILENGCSLVDEIHP12:HPP12_1287   MILGIDEAGRGCLAGSLFVAGVACNDQTALELLKMGLKDSKKLSLKKRFFLEDKIKTHGKVKFFVVKKSANEIDNLGLGACLKLAIQEILENNRSLANEIHF32:HPF32_1249   MTLGIDEAGRGCLAGSLFVAGVVCSEKTALEFLEMGLKDSKKLSPKKRFFLEDKIKTHGEIKFWVVKKSANEIDNLGLGACLKLAIQEILENDRSLANQIH51:KHP_1216      MTLGIDEAGRGCLAGSLFVAGVVCNEKTALEFLSMGLKDSKKLSPKKRFFLEDKIKTHGEVKFWVVKKSANEIDSLGLGVCLKLAIQEILENGRSLANKIH52:HPKB_1256     MILGIDEAGRGCLAGSLFVAGVVCSEKTALEFLEMGLKDSKKLSPKKRFFLEDKIKTHGEVEFWVVKKSANEIDRLGLGACLKLAIQEILENNRSLANGIHF30:HPF30_0077   MILGIDEAGRGCLAGSLFVAGVVCGEKTASEFLSMGLKDSKKLSPKKRFFLEDKIKTHGEMKFWVVKKSANEIDGLGLGACLKLAIQEILENGRSLANKIHF16:HPF16_1254   MILGIDEAGRGCLAGSLFVAGVVCSEKTASEFLSMGLKDSKKLSPKKRFFLEDKIKTHGEVEFWVVKKSANEIDSLGLGACLKLAIQEILENGRSLANGIHF57:HPF57_1279   MTLGIDEAGRGCLAGSLFVAGVVCSEKTALEFLEMGLKDSKKLSPKKRFFLEDKIKTHGEIKFWVVKKSANEIDNLGLGACLKLAIQEILENLSASPHAI                  101       111       121       131       141       151       161       171       181       191                         |         |         |         |         |         |         |         |         |         |         HB8:HPB8_156      KIDGNTAFGLNKRYPHIQTIIKGDEKIAQIAMASVLAKASKDREMRQLHALFKEYGWDKNCGYGTKQHIEAIIKLGATPFHRRSFTLKNRIFNPKLLEVEHG27:HPG27_1272   KIDGNTAFGLNKRYPNIQTIIKGDEKIAQIAMASVLAKAFKDREMRQLHALFKEYGWDKNCGYGTKQHIEAISKLGATPFHRHSFTLKNRILNPKLLEVEHSJM:HPSJM_06635  KIDGNTAFGLDKRYPNIQTIIKGDETIAQIAMASVLAKTAKDREMLELHALFKEYGWDKNCGYGTKQHIEAIIKLGATPFHRHSFTLKNRILNPKLLDVEHHPA:HPAG1_1268   KIDGNTAFGLNKRYPNIQTIIKGDETIAQIAMASVLAKAAKDREMLELHALFKEYGWDKNCGYGTKQHIEAIIKLGATPFHRHSFTLKNRILNPKLLEVEHB38:HELPY_1299   KIDGNTAFGLNKRYSNIQTIIKGDETIAQIAMASVLAKAAKDREMLELHALFKEYGWDKNCGYGTKQHIEAMSKLGATPFHRHSFTLKNRTLNPKLLEVEH266:mHP1323      KIDGNTAFGLNKRYPHIQTIIKGDETIAQIAMASVLAKAFKDREMLELHALFKEYGWDKNCGYGTKQHIEAIIKLGATPFHRHSFTLKNRILNPKLLEVEHP12:HPP12_1287   KIDGNTAFGLNKRYPNIQTIIKGDETIAQIAMASVLAKAFKDREMLELHALFKEYGWDKNCGYGTKQHIEAITKLGATPFHRHSFTLKNCILNPKLLEVEHF32:HPF32_1249   KIDGNTAFGLNKRYSNIQTIIKGDEKIAQIAMASVLAKAFKDREMRQLHALFKEYGWDKNCGYGTKQHIEAMIKLGATPFHRHSFTLKNRLFNPKLLEVEH51:KHP_1216      KIDGNTAFGLNKRYSNIETIIKGDEKIAQIAMASVIAKAFKDREMRQLHALFKEYGWDKNCGYGTKQHIEAMIKLGATPFHRHSFTLKNRLFNPKLLDVEH52:HPKB_1256     KIDGNTAFGLNKRYPNIQTIIKGDEKIAQIAMASVLAKAFKDREMRQLHALFKEYGWDKNCGYGTKQHIEAMIKLGATPFHRHSFTLKNRLLNPKLLDVEHF30:HPF30_0077   KIDGNTAFGLNKRYSNIETIIKGDEKIAQIAMASVLAKAFKDREMLKLHALFKEYGWDKNCGYGTKQHIEAMIKLGATPFHRHSFTLKNRIFNPKLLDVEHF16:HPF16_1254   KIDGNTAFGLNKRHSNIQTIIKGDEKIAQIAMASVLAKAFKDREMRQLHALFEEYGWDKNCGYGTKQHIEAMIKLGATPFHRHSFTLKNRIFNPKLLEVEHF57:HPF57_1279   KIDGNTAFGLNKRYSNIETIIKGDEKIAQIAMASVLAKAFKDREMRQLHALFKEYGWDKNCGYGTKQHIEAMIKLGATPFHRHSFTLKNRILNPKLLDVE                  201                  |HB8:HPB8_156      QRLIHG27:HPG27_1272   QRLIHSJM:HPSJM_06635  QRLVHHPA:HPAG1_1268   QRLVHB38:HELPY_1299   QRLIH266:mHP1323      QRLIHP12:HPP12_1287   QRLIHF32:HPF32_1249   QRLIH51:KHP_1216      QRLIH52:HPKB_1256     QRLIHF30:HPF30_0077   QRLIHF16:HPF16_1254   QHLIHF57:HPF57_1279   QHLI
