# Supplementary material for: Evolution in an oncogenic bacterial species with extreme genome plasticity: Helicobacter pylori East Asian genomes
Source: BMC Microbiol. 2011 May 16;11:104. doi: 10.1186/1471-2180-11-104 (PMC3120642; doi:10.1186/1471-2180-11-104)
Supplement: Additional file 6 — Multiple sequence alignments of diverged genes. [file 1471-2180-11-104-S6.ZIP › Diverged_genes_multiple_seuence_alignments/mHP1402_hsdR.mfa.rtf]

                  1         11        21        31        41        51        61        71        81        91                          |         |         |         |         |         |         |         |         |         |         HB8:HPB8_1704     -MKTEKEVQKQVIETFKSMGYAYLGDLTKSDNKNINKESLKAWLIKNQKINDERWQRIEQKINNALKNDLYEANQKFYELLIYGVKTKISQ-NEN-TQTTHSJM:HPSJM_07845  -MKTEKEVQKQVIETFKAMGYAYLGDLTKSDNENINKESLKAWLIKNQKISDERWHKIEQKIHDALKNDLYEANQKFYDLLIYGVKTKISQ-NEN-FQTTHG27:HPG27_1457   -MKTEREVQKQVIETFKSMGYAYLGDLTKSDNENINKESLKAWLIKNQKINNERWHKIEQKIHNALKNDLYEANQKFYELLIHGVQTKISQ-NEN-AQTHHB38:HELPY_1508   MVKTEKEVQKQVIETFKSMGYAYLGDLTKSDNENINKESLKAWLIKNQKINDERWHKIEQKIHNALKNDLYEANQTFYELLIYGVKTKISQ-NEN-TQTTH266:mHP1402      -MKTEKEVQKQVIETFKSMGYAYLGDLTKSDNENINKESLKAWLIKNQKIEPERWQRIEHKIHNALKNDLYEANQTFYELLIYGVKTKISQKNEN-TQTTHHPA:mHPAG1_1466  -MKTEKEVQKQVIETFKAMGYAYLGDLTKSDNENINRESLKAWLIKNQKINDERWHKIEHEIHNALKNDLYEANQKFYELLIYGVKTKISQ-NEN-TQTTHP12:aHPP12_045   ----------------------------------------------------------------------------------------------------HF32:HPF32_1421   -MKTEKEVQKQVIETFKSMGYAYLGDLTKSDNENINKESLKAWLVKNQKINHERWQRIEHEINNALKNDLYEANQKFYELLIYGVKTKISQ-NEP-TQTSHF57:HPF57_1449   -MKTEKEVQKQVIETFKSMGYAYLGDLTKSDNENINKESLKAWLVKNQKINDKRWQRIEHEINKALENDLYEANQKFYELLIYGVKTRISQ-NEP-TQTSH51:KHP_1387      -MKTEKEVQKQVIETFKSMGYAYLGDLTKSDNENINKESLKAWLVKNQKINDERWQRIEYKINDALNNDLYEANQKFYELLIYGVKTKISQ-NEP-TQTSH52:HPKB_1438     -MKTEKEVQKQVIETFKSMGYAYLGDLTKSDNENINKESLKAWLVKNQKINDKRWQRIEHEIHKALENDLYEANQKFYELLIYGVKTKTSQ-NEP-TQTSHF16:HPF16_1431   -MKTEKEVQKQVIETLKSMGYAYLGDLTKSDNENINKESLKAWLVKNQKINDERWQRIEHKINEALKNDLYEANQKFYELLIYGVKTTISQ-NEL-TQTSHF30:HPF30_1407   MVKTEKEVQKQVIETFKSMGYAYLGDLTKSDNENIDKESLKAWLVKNQKINDKRWQRIEHEINKALTNDLYEANQEFYELLIYGVKTKISQ-NEPTTQTS                  101       111       121       131       141       151       161       171       181       191                         |         |         |         |         |         |         |         |         |         |         HB8:HPB8_1704     YFIDWKDVSKNEFSVAEEVSVKGPNTKRPDIVLYVNGIALGVLELKKSSVSVESGIRQNLDNQKKEFIRDFFKTIQLVMAGNESQGLKYGVIETEEKYYLHSJM:HPSJM_07845  WLIDWKDVSKNEFSVAEEVSVKGPNAKRPDIVLYVNGIALGVLELKKSSVSVESGIRQNLDNQKKKFIRDFFKTIQLVMAGNESQGLKYGVIETKEKYYLHG27:HPG27_1457   WLIDWKDISKNEFSVAEEVSVKGPNAKRPDIVLYVNGIALGVLELKKSSVSVESGIRQNLDNQKKEFIRDFFKTIQLVMAGNESQGLKYGVIETKEKYYLHB38:HELPY_1508   YLIDWKDVSKNEFSVAEEVSVKGPNMKRPDIVLYVNGIALGVLELKKSSVSVESGIRQNLDNQKKEFIRDFFKTIQLVMAGNESQGLRYGVIETKEKYYLH266:mHP1402      WLIDWKDISENEFSVAEEVSVKGPNAKRPDVVLYVNGIALGVLELKKSSVSVESAIRQNLDNQKKEFIRDFFKTIQLVMAGNESQGLRYGVIETEEKYYLHHPA:mHPAG1_1466  WLIDWKDVSKNEFSVAEEVSVKGPNAKRPDVVLYVNGIALGVLELKKSSVSVESAIRQNLDNQKKKFIRDFFKTIQLVMAGNESQGLKYGVIETEEKYYLHP12:aHPP12_045   ----------------------------------------------------------------------------------------------------HF32:HPF32_1421   WLIDWKDVSKNEFSVAEEVSVKGANTKRPDIVLYVNGIALGVLELKNSSVSVESAIRQNLDNQKKEFIRDFFKTIQLVMAGNESQGLRYGVIETKEKHYLHF57:HPF57_1449   WLIDWKDVSKNEFSVAEEVSVKGPNMKRPDMVLYVNGIALGVLELKNSSVSVESAIRQNLDNQKKEFIRDFFKTIQLVMVGNESQGLRYGVIETKEKHYLH51:KHP_1387      WLIDWKDVFKNEFSVAEEVSVKGANTKRPDIVLYVNGIALGVLELKNSSVSVESAIRQNLDNQKKEFIRDFFKTIQLVMAGNESQGLRYGVIETKEKHYLH52:HPKB_1438     WLIDWKDVFKNEFSVAEEVSVKGSNTKRPDIVLYVNGIALGVLELKNSSVSVESAIRQNLDNQKKEFIRDFFKTIQLVMAGNESQGLRYGVIETKEKYYLHF16:HPF16_1431   WLIDWEDVFKNEFSVAEEVSVKGLNTKRPDMVLYVNGIALGVLELKNSSVSVESAIRQNLDNQKKEFIRDFFKTIQLVMAGNESQGLRYGVIETKEKHYLHF30:HPF30_1407   WLIDWKDVSKNEFSVAEEVSVKGQNMKRPDVVLYVNGIALGVLELKNSSVSVESAIRQNLDNQKKEFIRDFFKTIQLVMAGNESQGLRYGVIETKEKHYL                  201       211       221       231       241       251       261       271       281       291                         |         |         |         |         |         |         |         |         |         |         HB8:HPB8_1704     SWKEEGVLKNLFETIERFLKKERFLEFIHDFLIFDKGKKKCARFHQYFAIKKTQEFIQRKEGGIIWHTQGSGKSLTMVWLTRWLRRNKERARVLIVTDRRHSJM:HPSJM_07845  SWKEEGVLKNLFETIECFLKKERFLEFIHDFLIFDKGQKKCARFHQYFAIKKTQEFIKRKEGGIIWHTQGSGKSLTMVWLTQWLRKNRKQARVLIVTDRRHG27:HPG27_1457   SWKEGGVLKNLFETIECFLKKERFLEFIHDFLIFDKGQKKCARFHQYFAIKKTQEFIKRKEGGIIWHTQGSGKSLTMVWLTRWLRRNIKQARVLIVVDRRHB38:HELPY_1508   SWKEEGVQKNLFETIECFLDKKRFLEFIHDFLIFDKGQKKCARFHQYFAIKKTQEFIKRKEGGIIWHTQGSGKSLTMVWLAQWLRINTTQARVLIVTDRRH266:mHP1402      SWKEEGVLKNLFETIECFLKKERFLEFIHDFLIFDKGKKKCARFHQYFAIKKTQEFIQRKEGGIIWHTQGSGKSLTMVWLTRWLRRNIKQARVLIVIDRRHHPA:mHPAG1_1466  SWKEEGVLKNLFETIERFLKKERFLEFIHDFLIFDKGKKKCARFHQYFAIKKTQEFIQRKEGGIIWHTQGSGKSLTMVWLTQWLRRNRKQARVLIVTDRRHP12:aHPP12_045   -LERRGRLKNLFETIECFLKKERFLEFIHDFLIFDKGQKKCARFHQYFAIKKTQEFIKRKEGGIIWHTQGSGKSLTMVWLAQWLKMNIQQPRILIVTDRRHF32:HPF32_1421   SWKEEGVQKNLFETIECFLEKERFLEFIHDFLIFDKGQKKCARFHQYFAVKKTQEFIHKKEGGIIWHTQGSGKSLTMVWLTKWLRSNKEQARILIVTDRRHF57:HPF57_1449   SWKEEGVQKNLFETIECFLKKERFLEFIHDFLIFDKGQKKCARFHQYFAIKKTQEFIRRKEGGIIWHTQGSGKSLTMVWLTQWLRKNIKQARILIVTDRRH51:KHP_1387      SWKEEGVQKNLFETIECFLKKERFLEFIHDFLIFDKGQKKCARFHQYFAIKKTQEFIQRKEGGIIWHTQGSGKSLTMVWLTKWLRKNIKQARILIVTDRRH52:HPKB_1438     SWKEEGVQKNLFETIECFLKKERFLEFIHDFLIFDKGQKKCARFHQYFAVKKTQEFIHKKEGGIIWHTQGSGKSLTMVWLTQWLRKNIKQARILIVTDRRHF16:HPF16_1431   SWKEEGTQKNLFETIECFLQKERFLEFIHDFLIFDKGQKKCARFHQYFAIKKTQEFIHKKEGGIIWHTQGSGKSLTMVWLTKWLRSNIKQARILIVTDRRHF30:HPF30_1407   SWKEEGVQKNLFETIECFLKKERFLEFIHDFLIFDKGQKKCARFHQYFAIKKMQEFIHKKEGGIIWHTQGSGKSLTMVWLTQWLRKNIKQARILIVTDRR                  301       311       321       331       341       351       361       371       381       391                         |         |         |         |         |         |         |         |         |         |         HB8:HPB8_1704     ELDAQIQGVFEGIGEDLYRADSKKDLLSVLFENKEFLVSSLVHKFDDNDLEDLKKQPVLKEWVVLVDECHRTQSGKLHKAMKSLLPNAIFIAFSGTPLLKHSJM:HPSJM_07845  ELDAQIQGVFQGIGEKIYRADSKKDLLSVLFENKEFLVGSLVHKFDDNDLEDLKKQPVLKEWVVLVDECHRTQGGKLHKAMKSLLPNAIFIAFSGTPLLKHG27:HPG27_1457   ELDAQIQGVFLGIGEAIHRADSKKDLLSVLFENKEFLVGTLVHKFDDNDLEDLKKQPILKEWIVLVDECHRTQSAKLHKAMKSLLPNAIFIAFSGTPLLKHB38:HELPY_1508   ELDAQIQGVFEGIGEAIYRADSKKDLLSVLFENKKFLVGSLVHKFDDNDL----KQPVLKEWIVLVDECHRTQSGKLHKAMKSLLPNAIFIAFSGTPLLKH266:mHP1402      ELDAQIKGVFQGIGEDLYRADSKKDLLSALFENKEFLVGSLVHKFDDNDLEDLKKQPVLKEWVVLVDECHRTQSAKLHKAMKSLLPNAIFIAFSGTPLLKHHPA:mHPAG1_1466  ELDAQIQGVFEGIGEDLYRADSKKDLLSVLFENKEFLVGSLVHKFDDNDLEDLKKQPVLKEWIVLVDECHRTQGAKLHNAMKSLLPNAIFIAFSGTPLLKHP12:aHPP12_045   ELDAQIQGVFSGIGEDLYRADSKKDLLSVLFENKEFLVGSLVHKFDDNDLEDLKKQPVLKEWVVLVDECHRTQGGKLHKAMKSLLPNAIFIAFSGTPLLKHF32:HPF32_1421   ELDAQIQGVFDGIGEPIYRADSKKDLLSVLFENKEFLVGSLVHKFDDNDLEDLKKQPILKEWIVLVDECHRTQGGKLHNAMKSLLPNAIFIAFSGTPLLKHF57:HPF57_1449   ELDAQIQGVFEGIGEAIYRADSKKDLLSVLFENKEFLVGSLVHKFDDNDLEDLKKQPVLKEWIVLVDECHRTQSGKLHNAMKSLLPNAIFIAFSGTPLLKH51:KHP_1387      ELDAQIQGVFDGIGEPIYRADSKKDLLSVLFENKEFLVGSLVHKFDDNDLEDLKKQSVLKEWIVLVDECHRTQSGKLHNAMKSLLPNAIFIAFSGTPLLKH52:HPKB_1438     ELDAQIQGVFEGIGESICRADSKKDLLSVLFENKEFLVGSLVHKFDDNDLEDLKKQPVLKEWIVLVDECHRTQSGKLHNAMKSLLPNAIFIAFSGTPLLQHF16:HPF16_1431   ELDAQIQGVFLGIGEAIYRADSKKDLLSVLFENKEFLVGSLVHKFDDNDLEDLKKQPVLKEWIVLVDECHRTQSGKLHNAMKSLLPNAIFIAFSGTPLLKHF30:HPF30_1407   ELDAQIQGVFERMGEAIYRADSKKDLLSVLFENKEFLVGSLVHKFDDNDLGDLKKQPVLKEWIVLVDECHRTQSGKLHNAMKSLLPNAIFIAFSGTPLLK                  401       411       421       431       441       451       461       471       481       491                         |         |         |         |         |         |         |         |         |         |         HB8:HPB8_1704     QDKKTSQEVFGDYIHCYKFNEAVSDKVVLDLNYEARSVDQYVSSPEKLDEYFELKTQGLNDIAKAELKKKWVNLQKVFSTKDRLEHIAQDIVLDMAKLPRHSJM:HPSJM_07845  QDKKTSQEVFGNYIHCYKFNEAVSDKVVLDLNYEARSVDQYVSSPEKLDEYFELKTQGLNEAAKTELKKKWVNLQKVFSTKDRLARIVQDIVLDMAKLPRHG27:HPG27_1457   QDKKTSQEVFGNYIHCYKFNEAVSDKVVLDLNYEARSVDQYVSSSEKLDEYFELKTQGLNEAAKTELKKKWVNLQKVFSTKDRLARIVQDIVLDMAKLPRHB38:HELPY_1508   QDKKTSQEVFGNYIHCYKFNEAVSDKVVLDLNYEARSVDQYVSSSEKLDEYFELKTQGLNEAAKTELKKKWVNLQKVFSTKDRLARIVQDIVLDMAKLPRH266:mHP1402      QDKKTSQEVFGNYIHCYKFNEAVSDRVVLDLNYEARSVDQYVSSPEKLDEYFELKTQCLNDIAKTELKKKWVNLQKVFSTKDRLARIVQDIVLDMAKLPRHHPA:mHPAG1_1466  QDKKTSQEVFGDYIHCYKFNEAVSDKVVLDLSYEARSVEQYVSSPEKLDEYFELKTQNLNDAAKTELKKKWVNLQKVFSTKDRLARIVQDIVLDMAKLPRHP12:aHPP12_045   QDKKTSQEVFGDYIHCYKFNEAVSDRVVLDLNYEARSVDQYVSSPIKLDEYFELKTQGLNETAKIELKKKWANLQKVFSTKDRLARIVQDIVLDMAKLPRHF32:HPF32_1421   QEKKTSQEVFGDYIHCYKFNEAVSDKVVLDLNYEARSVDQYVSSPEKLDEYFELKTQNLNDAAKTELKKKWANLQKVFSTKNRLEHIVQDIVLDMAKLPRHF57:HPF57_1449   QDKKTSQEVFGDYIHCYKFNEAVSDKVVLDLNYEARSVDQYVSSPEKLDEYFELKTQNLNDTAKTELKKKWANLQKVFSTKNRLEHIVQDIVLDMAKLPRH51:KHP_1387      QDKKTSQEVFGDYIHCYKFNEAVSDKVVLDLNYEARSVDQYVSSPEKLDEYFELKTQNLNDTAKTELKKKWANLQKVFSTKNRLEHIVQDIVLDMAKLPRH52:HPKB_1438     QDKKTSQEVFGNYIHCYKFNEAVSDKVVLDLNYEARSVDQYVSSPEKLDEYFELKTQNLNDTAKTELKKKWANLQKVFSTKNRLEHIVQDIVLDMAKLPRHF16:HPF16_1431   QDKKTSQEVFGDYIHCYKFNEAVSDKVVLDLNYEARSVDQYVSSPEKLDEYFELKTQNLNDAAKTELKKKWANLQKVFSTKNRLEHIVQDIVLDMAKLPRHF30:HPF30_1407   QDKKTSQEVFGNYIHCYKFNEAVSDKVVLDLNYEARSVDQYVSSPEKLDEYFELKTQNLNDAAKTELKKKWANLQKVFSTKNRLEHIVQDIVLDMVKLPR                  501       511       521       531       541       551       561       571       581       591                         |         |         |         |         |         |         |         |         |         |         HB8:HPB8_1704     LRSEKGNAMLVAESVYNACRYFELFLETELKDKVAVITSYEPNIADLKDCGSNESEESYKYRIYRKMLQNFFNEKDEKKALNQIKEFEEKVKERFINEPNHSJM:HPSJM_07845  LRSEKGNAMLVAESVYNACRYFELFLETELKDKVAVITSYEPNIADLKDCGSDESEESYQYRAYCKMLQNFFDEKDEKKALNKIKEFEEKVKERFINEPNHG27:HPG27_1457   LRSEKGNAMLVAESVYNACRYFELFLETELKDKVAVITSYEPNIADLKDCGSDESEESYKYRAYCKMLQNFFNEKDEKKALNKIKEFEEKVKERFINEPAHB38:HELPY_1508   LRSEKGNAMLVAESVYNACRYFELFLETELKDKVAVITSYEPNIADLKDCGSDESEESYKYRAYCKMLQNFFDEKDEKKALNKIKEFEEKVKERFINEPNH266:mHP1402      LRSEKGNAMLVAESVYNACQYFELFLETELKDKVAVITSYEPNIADLKDCGSDESEESYKYRAYCKMLQNFFDEKDEKKALNKIKEFEEKVKDRFINEPNHHPA:mHPAG1_1466  LKNGKGNAMLVAESVYNACRYFELFLETELKDKVAVITSYEPNIADLKDCGSDESEESYKYRTYCTMLQNFFNEKDEKKALNKIKEFEEEVKKRFINEPDHP12:aHPP12_045   LRSGKGNAMLVAESVYNACRYFELFLETELKDKVAVITSYEPNIADLKDCGSDESEESYQYRAYCKMLQNFFNEKDEKKALNKIKEFEEKVKERFINEPNHF32:HPF32_1421   LSNGKGNAMLVAESVYNACRYFELFLETELKDKVAVITSYEPNITDLKDCGSNESEESYKYRAYCKMLQNFFNEKDEKKALNKTKEFEEEVKKRFINEPAHF57:HPF57_1449   LSNGKGNAMLVAESVYNACRYFELFLETELKDKVAVITSYEPNIADLKDCGSNESEESYKYRAYCKMLQNFFNEKDEKKALNKTKEFEEEVKKRFINEPAH51:KHP_1387      LSNGKGNAMLVAESVDNACRYFELFLETELKDKVAVITSYESNITDLKDCGSNESEESYKYRTYCKMLQNFFNEKDEKKALNKTKEFEEEVKKRFINEPAH52:HPKB_1438     LSNGKGNAMLVAESVYNACRYFELFLETELKDKVAVITSYEPNITDLKDCGSNESEESYKYRTYCKMLQNFFNEKDEKKALNKTKEFEEEVKKRFINEPAHF16:HPF16_1431   LSNGKGNAMLVAESVYNACRYFELFLETELKDKVAVITSYEPNITDLKDCGGNESEESYKYRAYCKMLQNFFNEKDEKKVLNKTKEFEEEVKKRFINEPSHF30:HPF30_1407   LKNGKGNAMLVAESVYNACRYFELFLETELKDKVAVITSYEPNITDLKDCGSNESEESYKYRAYCKMLQNFFNEKDEKKALNKTKEFEEEVKKRFINEPS                  601       611       621       631       641       651       661       671       681       691                         |         |         |         |         |         |         |         |         |         |         HB8:HPB8_1704     RMKLLIVVYKLLTGFDAPSLTYLYIDKKMQDHKLFQAVCRVNRLDSEDKDFGCIIDYSDLFDSLQEAHSDYTNGAFENYEREDIQGLISDKAQKIKKKLEHSJM:HPSJM_07845  RMKLLIVVDKLLTGFDAPSLTYLYMDKKMQDHGLFQAVCRVNRLDSEDKDFGCIIDYKDLFDSLQEAHSDYTNKAFENYEREDIQGLISDKSQKIKKKLEHG27:HPG27_1457   RMKLLIVVDKLLTGFDAPSLTYLYIDKKMQDHGLFQAVCRVNRLDGEDKDFGCIIDYKDLFDSLQEVHSDYTNKAFENYEREDIQGLISDKAQKIKKKLEHB38:HELPY_1508   RMKLLIVVDKLLTGFDAPSLTYLYMDKKMQDHELFQAVCRVNRLDSEDKDFGCIIDYKDLFDSLQEAHSDYTNKAFENYEREDIQGLISDKSQKIKKRLEH266:mHP1402      RMKLLIVVDKLLTGFDAPSLTYLYIDKKMQDHGLFQAVCRVNRLDGEDKDFGCIIDYSDLFDSLQEVHSDYTNKAFENYEREDIQGLISDKAQKIKKKLEHHPA:mHPAG1_1466  RMKLLIVVDKLLTGFDVPSLTYLYMDKKMQDHGLFQAVCRVNRLDSEDKDFGCIIDYSDLFDSLQEAHSDYTNGAFENYEREDIQGLISDKAQKIKKKLEHP12:aHPP12_045   RMKLLIVVDKLLTGFDAPSLTYLYMDKKMQDHGLFQAVCRVNRLDSEDKDFGCIIDYKDLFDSLQEAHSDYTNKAFENYEREDIQGLISDKSQKIKKRLEHF32:HPF32_1421   RMKLLIVVDKLLTGFDAPSLTYLYIDKKMQDHGLFQAVCRVNRLDGEDKDFGCIIDYSDLFESLQEAHSDYTNGAFENYEREDIQGFISNKAQKIKKKLEHF57:HPF57_1449   RMKLLIVVDKLLTGFDAPSLTYLYIDKKMKDHELFQAVCRVNRLDGEDKDFGCIIDYRDLFDSLQEVHSDYTNGAFENYEREDIQGLISDKAQKIKKKLEH51:KHP_1387      RMKLLIVVDKLLTGFDAPSLTYLYIDKKMQDHGLFQAVCRVNRLDGEDKDFGCIIDYSDLFDSLQEAHSDYTNKAFENYEREDIQGLISNKAQKIKKKLEH52:HPKB_1438     RMKLLIVVDKLLTGFDAPSLTYLYIDKKMQDHGLFQAVCRVNRLDGEDKDFGCIIDYSDLFDSLQEAHNDYTNGAFENYEKEDIQGLISSKAQKIKKKLEHF16:HPF16_1431   RIKLLIVVDKLLTGFDAPSLTYLYIDKKMQDHELFQAVCRVNRLDGEDKDFGCIIDYSDLFDSLQEAHSDYTNGAFENYEKEDIQGLISNKAQKIKKKLEHF30:HPF30_1407   RMKLLIVVEKLLTGFDAPSLTYLYIDKKMKDHELFQAVCRVNRLDGEDKDFGCIIDYSDLFDSLQEVHSDYTNGAFENYEREDIQGLISNKAQKIKKKLE                  701       711       721       731       741       751       761       771       781       791                         |         |         |         |         |         |         |         |         |         |         HB8:HPB8_1704     EARDQLRSLGESVKEPKDEMDYIAYFCGNDLEKNAQKRRLFYQLVGAFLRMFVELNNAEKPVYSKEEMQQIKQEAQFYRHLQKAVSLSSGDSVDLKSYSEHSJM:HPSJM_07845  ETRDQLKSLCESVKEPKDEEDYIAYFCGSDLEKNAQKRRLFYQLVGAFLRMFVELNNLEKPIYSKEETQKIKQEAEFYRHLQKAVGLSSGDSVDLKSYSEHG27:HPG27_1457   ETRDQLKSLCESVKEPKDEEDYIAYFCGSDLEKNAQKRRLFYQLVGAFLRMFVELNHLEKPIYSKEETQKIKQEAEFYRHLQKAIGLSSGDSVDLKSYSEHB38:HELPY_1508   ETRDQLKSLCESVKEPKDEMDYIAYFCGSDLEKNAQKRRLFYQLVGAFLRMFVELNNLEKPIYSKEETQKIKQEVEFYRHLQKVIGLSSGDSVDLKSYSEH266:mHP1402      EARDQLRSLGESVKEPKDEMDYIAYFCGNDLEKNAQKRRLFYQLVGAFLRMFVELNHLEKPVYSKEEMQQIKQEAEFYRHLQKVVSLSSGDSVDLKSYSEHHPA:mHPAG1_1466  EVREQLRSLGESVKEPKDEMDYIAYFCGSDLEKNAQKRRLFYQLVGAFLRMFVELNNLEKPIYSKEETQQIKQEAEFYRHLQKAVSLSSGDSVDLKSYSEHP12:aHPP12_045   ETRDQLKSLCESVKEPKDEEDYIAYFCGNDLEKNAQKRRLFYQLVGAFLRMFVELNNLEKPIHSKEETQQIKQEAEFYRHLQKVIGLSSGDSVDLKSYNEHF32:HPF32_1421   ETRDQLRSLCESVKEPKDETGYIAYFCGSDLEKNAQKRRLFYQLVGAFLRMFVELNNLEKPVYSKEEMQQIKQEAQFYRHLQKAVSLNSGDSVDLKSYSEHF57:HPF57_1449   EARDQLRSLCESVKEPKDETGYIAYFCGSDLEKNAQKRRLFYQLVGAFLRMFVELNNIEKPIYSKEEMQKIKQEAQFYRHLQKMIGLNSGDSVDLKSYSEH51:KHP_1387      EARDQLRSLCESVKEPKDETDYIAYFCGSDLEKNAQKRRLFYQLVGAFLRMFVELNHLEKPIYSQEEMQKIKQEAQFYRHLQKMIGLNSGDSVDLKSYSEH52:HPKB_1438     EARDQLSSLCESVKEPKDETGYIAYFCGSDLKKNAQKRRLFYQLVGAFLRMFVELNHLEKPIYSQEEMQKIKQEAQFYRHLQKMIGLNSGDSVDLKSYSEHF16:HPF16_1431   EARGQLRSLCESVKEPKDETGYIAYFCGNDLEKNAQKRRLFYQLVGAFLRMFVELNHLEKPIYSQEEMQKIKQEAQFYRHLQKMIGLNSGDSVDLKSYSEHF30:HPF30_1407   EARDQLRSLCESVKEPKDETGYIAYFCGSDLEKNAQKRRLFYQLVGAFLRMFVELNNLEKPIYSKEEMQKIKQEAQFYRHLQKMIGLNSGDSVDLKSYSE                  801       811       821       831       841       851       861       871       881       891                         |         |         |         |         |         |         |         |         |         |         HB8:HPB8_1704     DMRRILDAYIKATDSKTLIKIEDQGLCEVLAQMDINDFNKELSQAFKNESSMAESIANNTKKRIIEKEASDPKYYEKLSSLLNDLINQFREKKLTYLEYLHSJM:HPSJM_07845  DMRRILDAYIKTTDSEVLFQIEDQGLCEVLAQMDIDDFNKALSQVFKNKSSMAESIANNTKKRIVEKEASDPKYYGELSSLLNDLINQFREKKLTYLEYLHG27:HPG27_1457   DMRRILDAYIKATDSETLIKIEDQGLCEVLAQMDINDFNKALSQVFKNESSMAESIANNTKKRIVEKEASDPKYYEKLSSLLNDLILQFREKKLTYLEYLHB38:HELPY_1508   DMRRILDAYIKATDSEVLFQIEDQGLCEVLAQMDIDDFNKALSQVFKNESSMAESIANNTRKRIIEKEASDPKYYEKLSSLLNDLILQFREKKLTYLEYLH266:mHP1402      EMRRILDAYIKATDSKTLIKIEDQGLCEVLAQMDINDFNKELSQAFKNESSMAESIANNTKKRIIEKEASDPKYYEKLSSLLNDLIFQFREKKLTYLEYLHHPA:mHPAG1_1466  EMRRILDAYIKTTDSETLIKIEDQGLCEVLAQMDIDDFNKALSQAFKNESSMAESIANNTKKRIIEKEASDPKYYEKLSSLLNDLILQFREKKLTYLEYLHP12:aHPP12_045   DMRRILDAYIKTTDSEVLFKIEDQGLCEVLAQMDINDFNKTLSQAFKNESSMAESIANNTRKRIIEKEASDPKYYGELSSLLNDLILQFREKKLTYLEYLHF32:HPF32_1421   DMRRILDAYIKATDSKTLIKIEDQGLCEVLAQMDINDFNKELSEVFKNESSMAESIANNTRKRIIEKEASDPKYYEKLSSLLNDLINQFREKKLTYLEYLHF57:HPF57_1449   DMRRILDAYIKATDSKTLIKIEDQGLCEVLAQMDINDFHKELSEVFKNKSSMAESIANNIRKRIIEKEASDPKYYEKLSSLLNDLINQFREKKLTYLEYLH51:KHP_1387      DMRRILDAYIKATDSKMLIKIEDQGLCEVLAQMDINDFNKELSQVFKNESSMAESIANNTRKRIIEKEASDPKYYEKLSSLLNDLINQFREKKLTYLEYLH52:HPKB_1438     DMRRILDAYIKATDSKTLIKIEDQGLCEVLAQMDINDFNKELSEVFKNESSMAESIANNTRKRIIEKEASDPKYYEKLSSLLNDLINQFREKKLTYLEYLHF16:HPF16_1431   DMRRILDAYIKATDSKTLIKIEDQGLCEVLAQMDINDFHKELSQVFKNESSMAESIANNTRKRIIEKEASDPKYYEKLSSLLNDLINQFREKKLTYLEYLHF30:HPF30_1407   DMCRILDAYIKATDSKTLIKIEDQGLCEVLAQMDINDFHKELSQVFKNESSMAESIANNTRKRIIEKEASDPKYYKKLSSLLNDLINQFREKKLTYLEYL                  901       911       921       931       941       951       961       971       981       991                         |         |         |         |         |         |         |         |         |         |         HB8:HPB8_1704     QQIQHLAKQVIHKEDRNYPKKINTNALKTLYDNLDENDNLDKKEALALEIDACIRGNKKDGWVGHNQKEKNLKIALRKIINDEGLLENVFNLAKNIEEYRHSJM:HPSJM_07845  QQIHDLAKKVIDKEDRNYPKKINTKALKTLYDNLDEN------EALALEIDACIRGNKADDWVGHLMSERILKIALRKIINDEVLLENVFNLAKHIEEYHHG27:HPG27_1457   QQIRDLAKKVIDKEDRNYPKKINTNALKTLYDNLDEN------EALALEIDACIRGNKKDGWVGHNQKEKNLKIALRKIINDEGLLENIFNLAKHIDEYRHB38:HELPY_1508   QQIQNLAKKVIDKEDRNYPKKINTNALKTLYDNLDQN------EALALEIDACIRGDKKDGWVGHNQKEKNLKIALRKIINDEGLLENAFNLAKRIDEYRH266:mHP1402      QQIQHLAKKVIHKEDRNYPKKINTNALKTLYDNLDGN------EALALETDACIRGNKKDGWVGHNQKEKNLKIALRKIINDEGLLENTFNLAKHIDEYHHHPA:mHPAG1_1466  QQIHDLAKKVIDKEDKNYPKKINTKALKTLYDNLDEN------EALALDIDACIRDNKKDGWVGHNQKEKNLKIALRKIINDEGLLENTFNLAKRIDEYRHP12:aHPP12_045   QQIHDLAKKVIDKEDRNYPKKINTNALKTLYDNLDEN------EALTLEIDACIRGNKKGDWVGHNQKEKNLKIALRKIINDEVLLENAFNLAKHIKEYHHF32:HPF32_1421   QQIHNLAKQVIHKEDKNYPKKINTNALKTLYDNLDQN------EALALEIDACIRDNKKDGWVGHNQKEKNLKIALRKTINDEGLLENTFNLAKHIDEYHHF57:HPF57_1449   QQIHNLAKQVIHKEDKNYPKKINTNALKTLYDNLNQN------EALALEIDACIRDNKKDGWVGHNQKEKNLKIALRKTINDEGLLENTFNLAKHIGEYHH51:KHP_1387      QQIRNLAKQVIHKEDKNYPKKINTNALKTLYDNLDQN------EALALEIDACIRDNKKDGWVGHNQKEKNLKIALRKIINDEGLLENIFNLAKHINEYHH52:HPKB_1438     QQIHNLAKQVIHKEDKNYPKRINTNALKTLYDNLDQN------EALSLEIDACIRDNKKDGWVGHNQKEKNLKIALRKIINDEGLLENIFNLAKHIDEYHHF16:HPF16_1431   QQIRNLAKQVIHKEDKNYPKKINTNALKTLYDNLDQN------EALALEIDACIRDNKKDGWVGHHQKEKNLKIALRKTINDEGLLENTFNLAKHIDEYHHF30:HPF30_1407   QQIRNLAKQVIHKEDKDYPKKINTNALKTLYNNLDQN------EALALEIDACIRANKKDGWVGHNQKEKNLKIALRKIINDEGLLENIFNLAKHIDEYY                  1001                  |HB8:HPB8_1704     HSJM:HPSJM_07845  HG27:HPG27_1457   HB38:HELPY_1508   H266:mHP1402      HHPA:mHPAG1_1466  HP12:aHPP12_045   HF32:HPF32_1421   HF57:HPF57_1449   H51:KHP_1387      H52:HPKB_1438     HF16:HPF16_1431   HF30:HPF30_1407   
