# Supplementary material for: Evolution in an oncogenic bacterial species with extreme genome plasticity: Helicobacter pylori East Asian genomes
Source: BMC Microbiol. 2011 May 16;11:104. doi: 10.1186/1471-2180-11-104 (PMC3120642; doi:10.1186/1471-2180-11-104)
Supplement: Additional file 6 — Multiple sequence alignments of diverged genes. [file 1471-2180-11-104-S6.ZIP › Diverged_genes_multiple_seuence_alignments/mHP1415_miaA.mfa.rtf]

                  1         11        21        31        41        51        61        71        81        91                          |         |         |         |         |         |         |         |         |         |         HB8:HPB8_1572     ------------------------------------LDAEIFSLDSLSIYKDINIASAKPSLKERKDIKHYALDCLSIDEKNNAQLFKTLLEDAMRVSSKH266:mHP1415      ------------------------------------LDAEIFSLDSLSIYKDINIASAKPSLKERKNIKHYALDHLNIDEKNNAPLFKTLLEDAMRVSSKHG27:HPG27_1338   ------------------------------------LDAEIFSLDSLSIYKDINIASAKPSLKERKNIKHYALDYLNIDEKNNAQLFKTLLEDAMRVSSKHHPA:HPAG1_1341   ------------------------------------LDAEIFSLDSLSIYKDINIASAKPSLKERKNIKHYALDHLNIDEKNNAQLFKTLLEDAMRVSSKHSJM:HPSJM_07160  --------------------------------LAQELDAEIFSLDSLSIYKDINIASAKPSLKERKNIKHYALDHLNIDEKNNASLFKTLLEDAMRVSSKHB38:mHELPY_1383  ------------------------------------LDAEIFSLDSLSIYKDINIASAKPSLKERKNIKHYALDHLNIDEKNNAPLFKTLLEDAMRMSSKHF32:HPF32_1301   --------------------------------LAQELDAEIFSLDSLSIYKDINIASAKPSLKERKNIKHYALDYLNIDEKNNASLFKTLLEDAMRVSSKHF16:HPF16_1312   MPTKGRAFIKTPKKLIALLGPSGSGKSALSIELAQELDAEIFSLDSLSIYKDINIASAKPSLKERKNIKHYALDYLNIDEKNNASLFKTLLEDAMRVSSKH51:KHP_1266      ------------------------------------LDAEIFSLDSLSIYKDINIASAKPSLKERKNIKHYALDYLNIDEKNNASLFKTLLEDAMRVSSKH52:HPKB_1313     MPTKGRAFIKTPKKLIALLGPSGSGKSALSIELAQELDAEIFSLDSLSIYKDINIASAKLSLKERKNIKHYALDYLNIDEKNNASLFKTLLEDAMRVSSKHF30:HPF30_1283   MPTKGRASIKTPKKLIALLGPSGSGKSTLSIELAQELDAEIFSLDSLSIYKDINIASAKPSLKERKNIKHYALDYLNIDEKNNASLFKTLLEDAMRVSSKHF57:HPF57_1330   ------------------------------------LDAEIFSLDSLSIYKDINIASAKPSLKERKNIKHYALDYLNIDEKNNASLFKTLLEDAMRVSSKHP12:HPP12_1387   ------------------------------------LDAEIFSLDSLSIYKDINIASAKPSLKERKNIKHYALDHLNIDEKNNAQLFKTLLEDAMRVSSK                  101       111       121       131       141       151       161       171       181       191                         |         |         |         |         |         |         |         |         |         |         HB8:HPB8_1572     EILLIVGGSSFYLKSILEGLSRMPKLSNEEVVKIEREIATLSDPYIFLKSIDPNMAFKIHPNDTYRIHKALEIFYATHTPPSKYFKANPKKPFEHAISLFH266:mHP1415      EILLIVGGSSFYLKSILEGLSRMPKLSGEEVVKIEREIATLSNPYIFLKSIDPNMAFKIHPNDTYRTHKALEIFYATCTPPSEYFKANPKKPFEHAISLFHG27:HPG27_1338   EILLIVGGSSFYLKSILEGLSDTPKISGEEVVKIEREIATLSNPYIFLKSIDPNMAFKIHSNDTYRIHKALEIFYATHTPPSEYFKANPKKPFAHAISLFHHPA:HPAG1_1341   EILLIVGGSSFYLKSILEGLSRMPKISGEEVVKIEREISTLADPYAFLKSIDPTIAFKIHPNDTYRIHKALEIFYATHTPPSEYFKANPKKPFEHAISLFHSJM:HPSJM_07160  EILLIVGGSSFYLKSILEGLSDTPKLSGEEVVKIEREIAALTNPYAFLKSIDPNMAFKIHPNDTYRTHKALEIFYATHTPPSEYFKANPKKPFEHAISLFHB38:mHELPY_1383  EILLIVGGSSFYLKSILEGLSDTPKLSNEEVVKIEREIAALSNPYIFLKSIDPNMAFKIHPNDTYRIHKALEIFYATRTPPSEYFKANPKKPFEHAISLFHF32:HPF32_1301   EILLIVGGSSFYLKSILEGLSDMPKISGEEVVKIEREISSLANPYAFLKSIDPTSAFKIHPNDTYRIHKALEIFYSTHTPPSEYFKTNPKKPFEHAISLFHF16:HPF16_1312   EILLIVGGSSFYLKSILEGLSSMPKISGEEAAKIEREISSLANPYAFLKSIDPTSAFKIHPNDTYRTHKALEIFYSTHTPPSEYFKTNPKKPFEHAISLFH51:KHP_1266      KILLIVGGSSFYLKSILEGLSSMPKISGEEAVKIEREINSLANPYAFLKSIDPTSAFKIHPNDTYRTHKALEIFYSTHTPPSEYFKTNPKKPFEHAISLFH52:HPKB_1313     EILLIVGGSSFYLKSILEGLSSMPRISGEEVVKIEREISSLANPYAFLKSIDPTSAFKIHPNDTYRIHKALEIFYSTHTPPSEYFKTNPKKPFEHAISLFHF30:HPF30_1283   EVLLIVGGSSFYLKSILEGLSSMPKISGEEVVKIEREVSSLANPYAFLKSIDPTSAFKIHPNDTYRIHKALEIFYSTHTPPSEYFKTNPKKPFEHAISLFHF57:HPF57_1330   EILLIVGGSSFYLKSILEGLSSMPRISGEEVAKIEREINSLANPYAFLKSIDPTSTFKIHPNDTYRIHKALEIFYSTHTPPSEYFKTNPKKPFEHAISLFHP12:HPP12_1387   EILLIVGGSSFYLKSILEGLSDTPKISGEEVVKIEREISALANPYAFLKSIDPTIAFKIHPNDTYRIHKALEIFYLTHMPPSEYFKANPKKPFEHAISLF                  201       211       221       231       241       251       261       271       281       291                         |         |         |         |         |         |         |         |         |         |         HB8:HPB8_1572     ALSVEKNALHSNIKQRTKNMLHSGLVEEIKALYAKYPKDSQPFKAIGVKESVLFLEKQLTLKELEETITSNTIKLAKRQNTFNKTQFNNLYAGSVKEVRHH266:mHP1415      ALSIEKSALHNNIKRRTKNMLHSGLVEEIKALYTQYPKDSQPFKAIGVKESVLFLEKRLTLKELEEAITSNTMKLAKRQNTFNKTQFNNLYVGSAEEVRHHG27:HPG27_1338   ALSVEKNALHNNIKQRTKNMLHSGLIEEIKALYTQYPKDSQPFKAIGVKESILFLEKRLTLKELEETITSNTIKLAKRQNTFNKTQFNNLYTGSVKEVRHHHPA:HPAG1_1341   ALSIEKSTLHSNIKQRTKNMLHSGLVEEIKALYTQYPKDSQPFKAIGVKESILFLEKQLTLKELEEAIISNTMKLAKCQNTFNKTQFNNLYVGSVKEVRHHSJM:HPSJM_07160  ALSVEKSALHNNIKQRTKSMLDCGLIEEIKALYTQYPKDSQPFKAIGVKESVLFLEKRLTLKELEEAIVSNTMKLAKRQNTFNKTQFNNLYMGGVEEIRHHB38:mHELPY_1383  ALHIEKSALHNNIKQRTKSMLDCGLIEEIKALYTQYPKDSQPFKAIGVKESILFLEKQLTLKELEEAITSNTMKLAKRQNTFNKTQFNNLYAGSAEEVRHHF32:HPF32_1301   ALSIEKSALANNIKQRTKNMLDCGLIEEIKALYAKYPKNSQPFKAIGVKESILYLEKQLTLKELEEAIISNTIQLAKRQNTFNKTQFNNLYTGSVKEVRHHF16:HPF16_1312   ALSIEKSALANNIKQRTKNMLDCGLIEEIKALYAQYPKDSQSFKAIGVKESILYLEKQLTLKELEEAIVSNTIQLAKRQNTFNKTQFNNLYTGSVKEVRHH51:KHP_1266      ALSIEKNALANNIKQRTKNMLDCGLIEEIKALYAKYPKDSQPFKAIGVKESILYLEKQLTLKELEEAIVSNTIQLAKRQNTFNKTQFNNLYTGSVKEVRHH52:HPKB_1313     ALSIEKNALANNIKQRTKNMLDCGLIEEIKALYAQYPKDSQPFKAIGVKESILYLEKQLTLKELEEAIVSNTIQLAKRQNTFNKTQFNNLYTGSVGEVRHHF30:HPF30_1283   ALSIEKNALANNIKQRTKNMINCGLIEEIKALYAQYPKDSQPFKAIGVKESILYLEKQLTLKELEETIVSNTIKLAKRQNTFNKTQFNNLYMGSVGEVRHHF57:HPF57_1330   ALSIEKSTLANNIKQRTKNMLDCGLIEEIKALYAQYPKDSQPFKAIGVKESILYLEKQLTLKELEETIISNTIQLAKRQNTFNKTQFNNLYTGSVKEVRHHP12:HPP12_1387   ALHIEKNALHNNIKQRTKNMLHSGLVEEIKALYAKYPKDSQPFKAIGVKESVLFLEKQLTLKELEGAITFNPIKLAKRQNTFNKTQFNILYTGSVKEVRH                  301       311                  |         |HB8:HPB8_1572     AILKHSKSAIK-GH266:mHP1415      AILKHSKSGIK-GHG27:HPG27_1338   AILKHSKSGIK-GHHPA:HPAG1_1341   AILNHSKSAIK-GHSJM:HPSJM_07160  AILKHSKSDTRERHB38:mHELPY_1383  AILKHSKSGTKERHF32:HPF32_1301   AILNHSKSAIK-GHF16:HPF16_1312   AILNHSKSTIK-GH51:KHP_1266      AILNHSKSTIK-GH52:HPKB_1313     AILKHSKSAIK-GHF30:HPF30_1283   AILNHSKSAIK-GHF57:HPF57_1330   AILNHSKNAIK-GHP12:HPP12_1387   AILKHSKSA---Y
